# CD36 is involved in oleic acid detection by the murine olfactory system

**DOI:** 10.3389/fncel.2015.00366

**Published:** 2015-09-16

**Authors:** Sonja Oberland, Tobias Ackels, Stefanie Gaab, Thomas Pelz, Jennifer Spehr, Marc Spehr, Eva M. Neuhaus

**Affiliations:** ^1^Pharmacology and Toxicology, University Hospital Jena, Friedrich-Schiller-University JenaJena, Germany; ^2^Cluster of Excellence NeuroCure, Charité-Universitätsmedizin BerlinBerlin, Germany; ^3^Freie Universität-Berlin, Fachbereich Biologie, Chemie und PharmazieBerlin, Germany; ^4^Department of Chemosensation, Institute for Biology II, RWTH Aachen UniversityAachen, Germany

**Keywords:** olfactory receptor, CD36, Ca^2+^ imaging, STED, signal transduction, microscopy, fatty acid, olfaction

## Abstract

Olfactory signals influence food intake in a variety of species. To maximize the chances of finding a source of calories, an animal’s preference for fatty foods and triglycerides already becomes apparent during olfactory food search behavior. However, the molecular identity of both receptors and ligands mediating olfactory-dependent fatty acid recognition are, so far, undescribed. We here describe that a subset of olfactory sensory neurons expresses the fatty acid receptor CD36 and demonstrate a receptor-like localization of CD36 in olfactory cilia by STED microscopy. CD36-positive olfactory neurons share olfaction-specific transduction elements and project to numerous glomeruli in the ventral olfactory bulb. In accordance with the described roles of CD36 as fatty acid receptor or co-receptor in other sensory systems, the number of olfactory neurons responding to oleic acid, a major milk component, in Ca^2+^ imaging experiments is drastically reduced in young CD36 knock-out mice. Strikingly, we also observe marked age-dependent changes in CD36 localization, which is prominently present in the ciliary compartment only during the suckling period. Our results support the involvement of CD36 in fatty acid detection by the mammalian olfactory system.

## Introduction

Mammals have a demand to consume food with high fat content, possibly due to an evolutionary pressure-derived propensity to hoard energy. Behavioral studies in rodents have provided evidence for a role of olfaction in preference for fatty foods and triglycerides (Ramirez, 1993; Kinney and Antill, 1996; Takeda et al., 2001). Humans are also capable of detecting fatty acids by their odor (Bolton and Halpern, 2010; Wajid and Halpern, 2012; Chukir et al., 2013). Even in combination with other odorants present in food, humans can detect fat content by the sense of smell alone (Boesveldt and Lundstrom, 2014). The molecular basis of fat detection by olfactory sensory neurons is unknown so far.

Canonical sensory neurons in the main olfactory epithelium express one allele out of ~1200 olfactory receptor genes (Buck and Axel, 1991). Upon stimulation, olfactory receptors activate a specific Gαs isoform (Gα_olf_; Belluscio et al., 1998), leading to cAMP increase by activation of type III adenylyl cyclase (ACIII; Wong et al., 2000). The membrane depolarizes through Na^+^ and Ca^2+^ influx due to opening of cyclic nucleotide-gated (CNG) channels (Brunet et al., 1996) and subsequent activation of calcium-activated chloride channels (Reisert et al., 2005). Additional subpopulations of olfactory neurons are defined by specific expression of non-canonical receptors and/or distinct signaling mechanisms. Neurons expressing trace amine-associated receptors (TAARs) lack olfactory receptors, but share a Gα_olf_-dependent signaling cascade. The axons of TAAR neurons project to a specific set of glomeruli in the dorsal region of the olfactory bulb (Johnson et al., 2012; Pacifico et al., 2012). Another neuronal subpopulation expresses the receptor guanylyl cyclase GC-D and recognizes natriuretic peptides and small gaseous molecules (CO_2_ and CS_2_). These neurons project their axons to a specific region of the caudal olfactory bulb, and form approximately 12 “necklace glomeruli” (Hu et al., 2007; Leinders-Zufall et al., 2007; Munger et al., 2010).

In our study, we found that up to ~8% of mature olfactory neurons express the transmembrane glycoprotein cluster of differentiation 36 (CD36). CD36 is a well described receptor for fatty acids (Ibrahimi et al., 1996) that was so far unidentified in the peripheral mammalian olfactory system, although it has already been detected in central parts (e.g., piriform cortex, nucleus of the lateral olfactory tract; Glezer et al., 2009). A CD36 homolog is expressed in a subpopulation of insect olfactory neurons and plays an essential role in the detection of the fatty acid-derived pheromone *cis*-vaccenyl acetate (cVA; Benton et al., 2007). We here describe that CD36 is localized in cilia of olfactory neurons during the first weeks of life. Mice lacking CD36 display reduced response rates when challenged with oleic acid, a well described CD36 ligand.

## Materials and methods

### Animals

All animal procedures were in compliance with the European Union legislation (Directive 86/609/EEC) and FELASA recommendations. The study was approved by the Lageso (Landesamtes für Gesundheit und Soziales Berlin, T0264/11). C57BL/6 (Charles River Laboratories, Sulzfeld, Germany) and CD36 knockout mice (Febbraio et al., 1999) were housed at RT in a 12:12 h light dark cycle with food and water *ad libitum*. Mice were used for analysis at ages between P6 and P14, unless indicated otherwise.

### Antibodies

Primary antibodies: rat anti-CD36 (AbD Serotec, MCA2748, MCA2748A488, dilution 1.200); goat anti-CNGA2 (Santa Cruz, M-20, dilution 1:200); rabbit anti-ACIII (Santa Cruz, C-20, 1:200); rabbit anti-ANO2, dilution 1:100 (Rasche et al., 2010), rabbit anti-mOR-EG, dilution 1:200 (Baumgart et al., 2014); rabbit anti-Gα_olf_ (Santa Cruz, C18, dilution 1:200), goat anti-OMP (Wako, 019-22291, dilution 1:1000). Secondary antibodies (Life Technologies) were coupled to Alexa Fluor dyes (Alexa Fluor® 568 donkey anti-rabbit A10042, Alexa Fluor® 633 goat anti-rabbit A21070, Alexa Fluor® 488 donkey anti-rabbit, A21206, Alexa Fluor® 488 donkey anti-goat A11055, Alexa Fluor® 568 donkey anti-goat A11057, Alexa Fluor® 488 donkey anti-rat A21208, Alexa Fluor® 568 goat anti-rat A11077, Alexa Fluor® 633 goat anti-rat A21094), all were used in a dilution of 1:500. Additional counterstaining was performed with TO-PRO®-3 (1:2000, Life Technologies, T3605).

### Solutions

PBS^−/−^: 2.68 mM KCl, 1.47 mM KH_2_PO_4_, 136 mM NaCl, 8.1 mM Na_2_HPO_4_, pH 7.4; PBS^+/+^: 1x PBS^−/−^, 0.9 mM CaCl_2_, 0.48 mM MgCl_2_, pH 7.4; 2x Laemmli buffer: 20% glycerol, 4% SDS, 125 mM Tris, 0.02% bromophenol blue, 200 mM DTT; Homogenization buffer: 300 mM sucrose, 5 mM Tris-Cl, 0.1 mM EDTA, pH 7.4+1 fresh cOmplete Mini Protease Inhibitor Cocktail tablet (Roche); Solubilization buffer: 10% glycerol, 150 mM NaCl, 20 mM Tris-Cl, 1% CHAPSO, 1 cOmplete Mini Protease Inhibitor Cocktail tablet (Roche); Fixative solution: 2.68 mM KCl, 1.47 mM KH_2_PO_4_, 0.136 mM NaCl, 8.1 mM Na_2_HPO_4_, 0.2 mM CaCl_2_, 4% sucrose, 4% paraformaldehyde; ACSF (artificial cerebrospinal fluid): 119 mM NaCl, 26 mM NaHCO_3_, 2.5 mM KCl, 1 mM NaH_2_PO_4_, 10 mM glucose, 2.5 mM CaCl_2_, 1.3 mM MgCl_2_, pH 7.4.

### Standard immunostaining protocol

Tissue was rinsed two times in PBS^+/+^ followed by incubation in fresh blocking solution [1x PBS^+/+^, 0.1% Triton X-100, 1% gelatin (Sigma, G7765)] for at least 1 h. Primary antibodies were diluted in blocking solution and put on the tissue over night at 4°C. After five times rinsing in PBS^+/+^ for 5 min each, the tissue was incubated in secondary antibody (1:500) solution for 1 h. The tissue was rinsed three times for 5 min each and mounted in ProLong® Gold antifade reagent (Life Technologies).

### Quantitative real-time PCR

Total RNA from olfactory epithelium (septum und turbinates) was isolated with RNeasy Mini Kit (Qiagen) including DNaseI digestion. cDNA was prepared using iScript cDNA Synthesis Kit (Bio-Rad). PCR reactions were performed with an iQ5 thermal cycler (Bio-Rad) using iQ SYBR Green Supermix. Expression levels were calculated using the ddCt method. Primers for housekeeping gene α1b-tubulin were used as described (Dubacq et al., 2009). Predesigned QuantiTect primers for CD36 (QT01058253), mOR-EG (QT00278789) and Olfr124 (QT00304794) were purchased from Qiagen. PCR conditions were 30 s, 94°C; 30 s, 55°C; 30 s, 72°C; 35 cycles.

### OE membrane preparation and western blotting

Membrane preparation was performed on ice to avoid protein degradation. Olfactory epithelium was collected, homogenized, and centrifuged for 30 min at 20,000 g at 4°C. After solubilization, protein samples were mixed with Laemmli buffer and separated in a 10–15% SDS gel. Proteins were transferred to nitrocellulose membrane. Membranes were blocked in 5% dry milk (Bio-Rad) in TBST and incubated with the antibodies that were detected using ECL Select™ and ECL Hyperfilm (Amersham).

### Olfactory neuron dissociation

Dissociation of olfactory sensory neurons was performed using the Miltenyi neural tissue dissociation kit for postnatal neurons. Main olfactory epithelium was collected in pre-heated enzyme mix 1 (10 μl solution 1+1950 μl solution 2) and cut into smaller pieces with spring scissors. After 15 min incubation at 37°C on the MACSmix™ Tube Rotator (Miltenyi), enzyme mix 2 (30 μl solution 3+15μl solution 4) was added and incubated for 10 min at 37°C on the Tube Rotator.

For immunohistochemistry, samples were centrifuged for 2 min at 800 rpm and resuspended in 400 μl fixative solution. The cells were dispersed on adhesion slides (Polysine® slides, Thermo Scientific) on which an area was encircled with a hydrophobic marker pen (PAP pen, Sigma-Aldrich). The cells were incubated in fixative solution for 10 min at room temperature without moving the slides to let the OSNs settle down on the glass. All washing and staining steps were performed very carefully, as OSNs and their connection to the glass slide were very fragile. Solutions were sucked off by pipetting and new solutions were applied by slowly pipetting them in one corner of the encircled area. Primary antibody solution was applied for 1–3 h, secondary antibody solution for 45 min.

### Transcriptome sequencing

For transcriptome sequencing, olfactory epithelium from wild type P8 pups was collected and dissociated as described above. CD36 expressing cells were separated using the magnetic cell separation system from Miltenyi Biotec (MiniMACS™ Separator with Anti-Rat IgG MicroBeads). RNA from the CD36-enriched fraction and from the cells passing the column was isolated using the RNeasy Micro Kit (Qiagen). Sequencing libraries were generated by Eurofins MWG Operon (Ebersberg, Germany). Briefly, a 3′-fragment cDNA library with fragment sizes of 200–450 bp was created for every sample. Libraries were sequenced on Illumina HiSeq 2000 with chemistry v3.0 and with 1 × 100 bp single read module. Reads were mapped on a “protein-coding unspliced-genes” reference from Ensemble (GRCm38.p1, created by Eurofins MWG Operon) using the software BWA-backtrack. 55.06 and 53.97% reads were mapped of 25607716 and 28205993 total reads for the CD36-enriched fraction and the CD36-depleted fraction, respectively. The mean read coverage (covered reference bases/sum of all reference bases) accounted for 148.5 for the CD36-enriched and 185.7 for the CD36-depleted cell fraction. Samples were normalized using the Trimmed Mean of *M*-values (TMM) method (Robinson and Oshlack, 2010), and analyzed using the Integrative Genomics Viewer (Robinson et al., 2011). Mapped reads were termed by gene accession numbers. To assign gene names to provided accession numbers, we used a self-made “WebRequest” tool, designed and kindly provided by Sven Fabricius (http://www.designerco.de/). Gene names were automatically requested from the NCBI database (http://www.ncbi.nlm.nih.gov/), exported to an overview table and manually imported in Excel. Gene function was evaluated manually by PubMed searches. Olfactory receptor transcripts were counted when detected by at least 10 raw counts.

### Cryosections

The head was fixed for 2 h at 4°C in fixative solution, cryoprotected in 30% sucrose, and embedded in Leica tissue freezing medium (Leica Microsystems). Sections of 12 μm thickness were cut on a Leica CM 1900 UV cryostat (Leica Microsystems), adhered to Super Frost® Plus cryoslides (Thermo Scientific) and stored at −25°C.

### Vibratome sections

Pups were decapitated and the skin, lower jaw, palate, and front teeth were removed from the head. The back part of the head was removed and the front part was glued to the specimen plate of a VT1200S microtome (Leica Microsystems) and embedded in 2% low-gelling temperature agarose. Sections of 100–200 μm thickness were cut and collected in ice-cold ACSF in a 24 well plate. Sections were fixed for 20 min at room temperature and blocked for 1 h in blocking solution. Antibody staining was either performed for 3 h with a CD36 antibody conjugated to Alexa Fluor 488 or following the standard immunostaining protocol.

### Whole-mount and *En Face* preparation

Mice were decapitated and skin and bones removed. The head was split with a razor blade 1 mm beside the sagittal midline to expose one nasal cavity. Turbinates, nasal septum or bulbs were carefully removed, and fixed for 10–30 min at room temperature. The tissue was collected in Ringer’s solution without touching the epithelial surface. Epithelium sheets were carefully transferred to an adhesion slide (Polysine® slides, Thermo Scientific) by moving it with fine forceps and fixed for 10 min at room temperature. Top view preparations were handled very cautiously to prevent damage of fine cilia structures. All solutions were applied by pipetting next to the tissue (Oberland and Neuhaus, 2014). Antibody staining was performed for 3 h or o.n.

### Cilia characteristics

*En face* preparations were used to characterize cilium characteristics of CD36+ and mOR EG+ OSNs. Analyzed regions were randomly chosen with bright field microscopy, confocal images of both fluorescence stainings were taken. ImageJ (Schneider et al., 2012) was used to mark all cilia of one OSN. A freehand line was drawn for every cilium from its base at the knob to its end. Selections were collected in the ROI manager (regions of interest) and the length was measured by ImageJ. Data was transferred to Excel and further processed.

### Microscopy

Widefield fluorescence microscopy of cryosections and for olfactory neuron counting was documented using bright field microscopy on a Leica DMI6000 B system (Leica microsystems). Whole-mount stainings of turbinates and olfactory bulbs and vibratome sections were analyzed with the fluorescence stereo microscope M205FA (Leica Microsystems). Documentation of stainings in cryosections, *en face* preparations, and vibratome sections was predominantly performed using confocal microscopy with TCS SPE and TCS SP5 systems (Leica microsystems). For Ca^2+^ imaging in main olfactory epithelium slices, we used an upright confocal microscope DM6000CFS (Leica Microsystems) equipped with a 20x 1.0 NA objective. Images were further processed using LAS AF, ImageJ, and Photoshop. To analyze protein distribution in more detail we used stimulated emission depletion (STED) microscopy (Dyba et al., 2003). We used the TCS STED system on a confocal TCS SP5 II platform with STED CW laser (Leica microsystems), as described before (Henkel et al., 2015).

Cryosections from the age stages E18, P0, P1, P8, P14, P21, P28, P60, P90, and P180 were co-immunostained with CD36 and OMP antibody, only areas with OMP staining were analyzed. The LOCI plugin for ImageJ was used for cell counting. Every neuron was marked by hand and counted by the software. For statistical analysis, we chose defined stretches of 600 μm olfactory epithelium lining the septum and counted CD36 and OMP expressing cells for P0, P1, P8, P14, P21, P28, P60, P90, and P180.

### RNA *In situ* hybridization

RNA *in situ* hybridization was performed using non-fluorescent digoxigenin labeled probes. CD36 riboprobes cover around 1400 bp and were prepared as described (Glezer et al., 2009). RNA *in situ* primers (sequence 5′ to 3′):

CD36_IS_Rive_fw GTAAAGCTTATGGGCTGTGATCGGAACTGTGGGCG

CD36_IS_Rive_rv GTAGAATTCTTATTTTCCATTCTTGGATTTGCAAGCAC

PCR products were cloned into the pcDNA3 vector. The vector was linearized according to the manufacturer instructions (Roche) at the 3′ or the 5′ end of the insert, to start *in vitro* transcription with T7 or SP6 RNA Polymerase to get the negative sense probe or the positive antisense probe, respectively (DIG RNA Labeling Mix (11277073910), transcription buffer (11465384001), Protector RNase Inhibitor (03335399001), T7 or SP6 RNA polymerase (10881767001, 10810274001). After 2 h incubation at 37°C, the reaction was inactivated by adding 0.2 M EDTA (pH 8.0). RNA was cleaned up using the RNeasy Mini Kit and stored at −80°C.

Stored unfixed cryosections were thawed and dried on a 37°C heating plate and fixed for 20 min at 4°C with PFA-solution. Cells were lysed with RIPA buffer (0.25% SDS, 150 mM NaCl, 1% NP40, 0.5% sodium deoxycholate, 1 mM EDTA, 50 mM Tris-Cl) for 10 min, washed with 2x SSC (20x SSC stock: 3 M NaCl, 0.3 M Na_3_C_6_H_5_O_7_), dehydrated in a graded ethanol series (50, 75, 95, 100% ethanol, 5 min each) and air dried. After washing the sections for 10 min with 2x SSC, they were incubated in 250 ml TEA (0.1 M triethanolamine, pH 8.0) on a magnetic stirrer for 5 min. To reduce non-specific probe binding, slices were acetylated by adding dropwise 600 μl acetic anhydride following incubation for 15 min. Sections were washed two times in 2x SSC and incubated in prehybridization buffer (hybridization buffer without probe) for 1 h at 55°C in a humid chamber. Finally, the hybridization buffer (10% dextran sulfate, 4x SSC, 2.5 mM EDTA, 50% formamide, 1x Denhardts solution (1% BSA fraction V, 1% Ficoll PM400, 1% PVP), 250 μg/ml yeast tRNA, 50 μg/ml heparin, 0.1% Tween-20, 500 μg/ml salmon sperm DNA) containing the riboprobe (15–30 ng/100 μl) was added to the slices for 16–20 h at 55°C in a humid chamber. Sections were washed in 2x SSC, incubated in 0.2x SSC for 1 h at 55°C to remove unspecific probe binding and washed again in 2x SSC and 0.2x SSC. To aid antibody penetration, the slices were incubated in PBST (1x PBS^−/−^, 0.1% Tween-20) for 10 min following blocking solution (1x PBS^−/−^, 0.1% Triton X-100, 10% normal goat serum) for 1 h. Anti-digoxigenin-AP Fab fragments (Roche, 11093274910) were used in a 1:100 dilution in blocking solution for 2 h at room temperature and additional 16–20 h at 4°C.

Sections were rinsed with PBST three times for 20 min each and equilibrated in buffer B3 (0.1 M Tris-Cl, 0.1 M NaCl, 50 mM MgCl2, 0.1% Tween-20) for 10 min. The final developing step was performed using freshly prepared buffer B4 (NBT/BCIP ready-to-use tablets, Roche 11697471001) for at least 30 min up to 3 days, depending on the staining intensity. Reaction was stopped by washing the sections with PBST for 5 min. After rinsing the slices in H_2_O, they were mounted in ProLong® Gold antifade reagent (Life Technologies, P36930).

### Electro-olfactogram recordings

Mice were decapitated and skin and lower jaw removed. The head was split with a razor blade 1 mm beside the sagittal midline to expose one nasal cavity. Turbinates were removed to uncover the olfactory epithelium of the nasal septum. The head was fixed in a recording chamber half-filled with agarose and connected to an indifferent electrode. The electro-olfactogram (EOG) recording setup consists of a glass pipette electrode holder fixed to a probe that is connected to an IDAC-4 data-acquisition amplifier (Ockenfels Syntech GmbH). The glass pipette is filled with Ringer’s solution and gets in contact to the probe by an inserted tungsten wire. The stimulus controller (CS-55, Ockenfels Syntech GmbH) generates a continuous humidified air flow onto the epithelium with triggered stimulus pulses, and was connected to the amplifier. Odors were loaded on a filter paper, inserted in a modified syringe and introduced in the stimulus air stream. We used a mixture of 100 odorants (Henkel 100, 1:200 in H_2_O, Henkel KGaA) for stimulation (Wetzel et al., 1999). Stimulus air flow was applied for 0.1 s. Signals were recorded and analyzed with the software AutoSpike (Ockenfels Syntech GmbH).

### Ca^2+^ imaging

The following solutions were used for Ca^2+^ imaging experiments: (a) Extracellular solution (S1): 145 mM NaCl, 5 mM KCl, 1 mM CaCl_2_, 1 mM MgCl_2_, 10 mM HEPES; pH 7.3 (adjusted with NaOH); osmolarity = 300 mOsm (adjusted with glucose). (b) Oxygenated extracellular solution (S2): 120 mM NaCl, 25 mM NaHCO_3_, 5 mM KCl, 1 mM CaCl_2_, 1 mM MgSO_4_, 5 mM BES; pH 7.3; osmolarity = 300 mOsm. (c) Elevated potassium solution (S3): 100 mM NaCl, 50 mM KCl, 1 mM CaCl_2_, 1 mM MgCl_2_, 10 mM HEPES; pH 7.3 (adjusted with NaOH); osmolarity = 300 mOsm. Oleic acid was diluted in S1 to final concentrations and solved immediately before use using a vortex and sonication for at least 2 min. A mixture of 10 odorants (Acetophenone, Benzylacetate, Cineol, Cyclohexanone, Eugenol, Geraniol, Isoamyacetate, Isovaleric acid, Octanal, Phenylbutyric acid; final concentrations 10 μM each) was used to increase response probability. Final DMSO concentrations were ≤ 0.1%. Stimuli and pharmacological agents were applied manually via a 5-in-1 multi-barrel “perfusion pencil” (Veitinger et al., 2011).

Acute main olfactory epithelium slices were prepared from young mice of either sex between postnatal day 6 and 9. Mice were sacrificed by decapitation. Acute coronal main olfactory epithelium tissue slices were prepared as described (Fluegge et al., 2012). Briefly, the skin, lower jaw, teeth and palate were removed. The anterior aspect of the head was dissected, embedded in 4% low-gelling temperature agarose, placed in ice-cold oxygenated S2, and coronal slices (250 μm) were cut on a VT1000S vibratome (Leica Microsystems, Nussloch, Germany). Slices were transferred to a submerged, chilled, and oxygenated (S2) storage chamber until use. Slices were incubated in fluo4/AM-containing S1 (10 μM; 30 min; RT; Molecular Probes), washed, transferred to a Slice Mini Chamber (Luigs and Neumann, Ratingen, Germany), anchored (0.1 mm thick lycra threads), and superfused with oxygenated S2 (~3 ml/min; gravity flow). Fluo-4 was excited at 488 nm and images were acquired at 1.0 Hz (~10 μm optical thickness).

Time-lapse experiments were analyzed using LAS-AF and MM-AF software (Leica Microsystems). All cells in a field of view were marked as ROIs and relative fluorescence intensities were calculated as arbitrary units. Data are reported as means from at least three independent experiments and statistical analyses were performed using a chi-squared (Fisher’s exact) as dictated by data distribution and experimental design. *p*-values that report statistical significance (≤ 0.05) are individually specified in figure legends. In fluorescence imaging experiments, ROIs were defined to encompass single cells based on visually identified morphological features of dye-loaded cells at rest. An increase in fluorescence intensity was judged as a stimulus-dependent response if the following two criteria were both fulfilled: (a) the peak intensity value exceeded a given threshold that was calculated as the average baseline intensity before stimulation plus an intensity value corresponding to twice the baseline intensity standard deviation (I_resp_ > I_baseline_+2 × SD(I_baseline_) (Riviére et al., 2009; Ferrero et al., 2011; Cichy et al., 2015); (b) the increase in light intensity was observed within 10 s after stimulus application.

## Results

### CD36 is expressed in a subset of neurons in the olfactory epithelium

**Figure 1:**
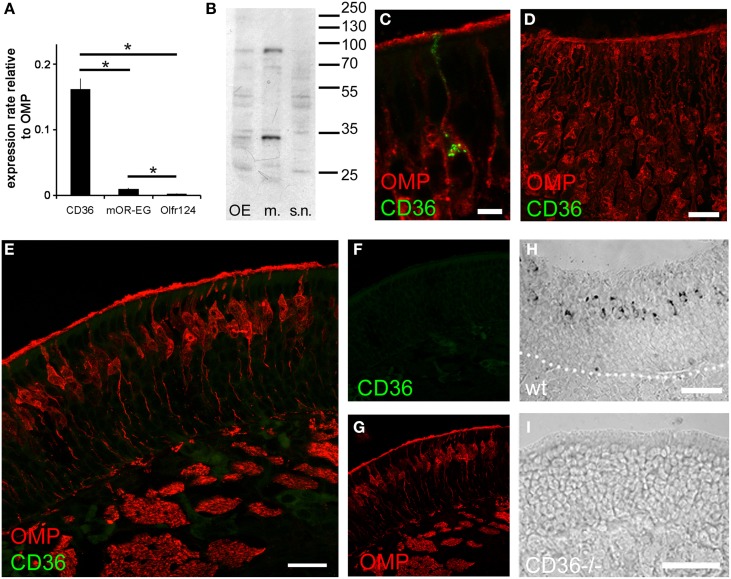
**CD36 is expressed in the olfactory epithelium. (A)** Quantitative real-time PCR showing expression of CD36 in adult mice compared to two olfactory receptors (mOR-EG and Olfr124). Data are the mean of five mice (*n* = 5) and three technical replicates each. Significance was calculated using two sample *t*-test. Error bars represent SEM (^*^p ≤ 0.05). **(B)** Western blot analysis of whole olfactory epithelium preparation, membrane preparation and supernatant of membrane preparation of an adult mouse showing CD36 protein expression. **(C)** Confocal image of a cryosection (P8) immunostained for CD36 (green) and the mature olfactory neuron marker OMP (red). CD36 is present in soma, dendrite and knob. **(D)** Confocal image (maximum projection) of an adult vomeronasal organ cryosection immunostained for OMP (red) and CD36 (green) showing densely packed vomeronasal sensory neurons but no CD36 presence in these neurons. **(E)** Confocal image of a cryosection (P8) immunostained for CD36 (green) and the mature olfactory neuron marker OMP (red) in CD36^−/−^ mice, showing absence of labeling. **(F,G)** Single channels of the picture shown in **(E)**. **(H)** RNA *in situ* hybridization of wild-type P8 cryosection showing CD36 mRNA expression in OSNs. **(I)** RNA *in situ* hybridization of cryosection from CD36^−/−^ mouse showing the absence of CD36 mRNA. Scale bars: 5 μm **(C)**, 20 μm **(D)**, 20 μm **(E)**, 50 μm **(H)**, 50 μm **(I)**.

We detected CD36 in olfactory epithelia of mice by previous proteomic analysis (Rasche et al., 2010), CD36 protein has also been found to be highly abundant in the proteome of rat cilia (Mayer et al., 2009). We therefore analyzed the expression pattern of CD36 in the olfactory system. Quantitative real-time PCR revealed that the expression level of CD36 was significantly higher compared to olfactory receptors Olfr73 (mOR-EG) and Olfr124 (CD36: 0.161, mOR-EG: 0.009, Olfr124: 0.003; Figure 1A), similar as the protein amounts detected in previous proteomic approaches. We also detected the described 88 kD glycosylated form of CD36 protein (Talle et al., 1983; Abumrad et al., 1993; Han et al., 1997) by western blot analysis of whole olfactory epithelium and of olfactory epithelium membrane preparations, but not in the supernatant fraction containing soluble cytosolic proteins (Figure 1B).

Immunostaining of coronal olfactory epithelia cryosections revealed CD36 localization in a subset of mature sensory neurons, identified by expression of the olfactory marker protein (OMP; Figure 1C). By contrast, we did not find CD36 expression in the accessory olfactory system (Figure 1D). We then used a previously generated CD36 knockout mouse strain (CD36^−/−^; Febbraio et al., 1999) to confirm antibody specificity. Using OMP for counterstainings to visualize mature olfactory sensory neurons we found CD36 staining to be completely absent from the olfactory epithelium of CD36^−/−^ mice (Figures 1E–G). Using RNA *in situ* hybridization we moreover detected CD36 mRNA in sensory neurons of P8 wild-type mice (Figure 1H), but not in olfactory epithelium from CD36^−/−^ animals (Figure 1I).

### CD36 does not show zonal expression pattern

**Figure 2:**
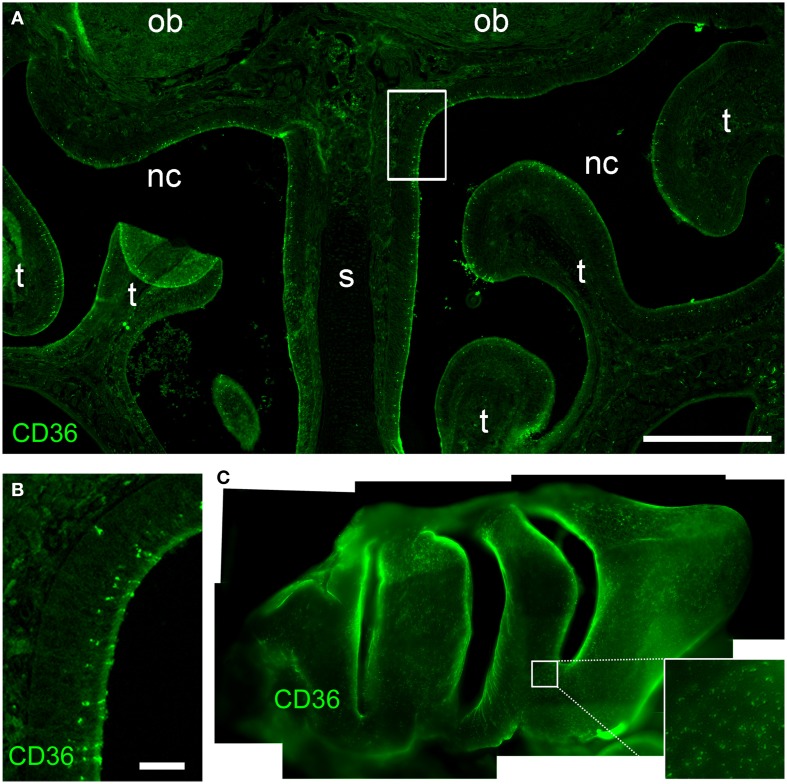
**CD36 distribution in the olfactory epithelium. (A)** Widefield microscopy image of a cryosection (P8) immunostained for CD36. CD36 expressing neurons are scattered along the olfactory epithelium. (nc, nasal cavity; s, septum; t, turbinate; ob, olfactory bulb). **(B)** Magnification of the boxed area shown in **(A)**. Immunostaining for CD36 shows expression in a subset of olfactory neurons. **(C)** Stitching of fluorescence stereo microscopy images of a whole-mount turbinate preparation immunostained for CD36 (P8). Olfactory knobs of CD36 expressing neurons are detected as small dots all along the surface of the epithelium lining the turbinates Scale bar: 500 μm **(A)**, 50 μm **(B)**.

Most olfactory sensory neurons that express a specific odorant receptor are restricted to one of four zones of the olfactory epithelium in the nasal cavity in the mouse (Ressler et al., 1993; Vassar et al., 1993). Also TAAR genes are expressed in unique populations of sensory neurons spread in single zones of the olfactory epithelium (Liberles and Buck, 2006). By contrast, CD36 expressing cells were not confined to a specific epithelial region (Figure 2). Analysis of cryosections from both rostral and caudal areas of the olfactory epithelium (Figure 2A) and whole-mount turbinate preparations (Figure 2C) revealed non-zonal CD36 expression along the septum and on all turbinates.

### CD36-positive glomeruli in the olfactory bulb

**Figure 3:**
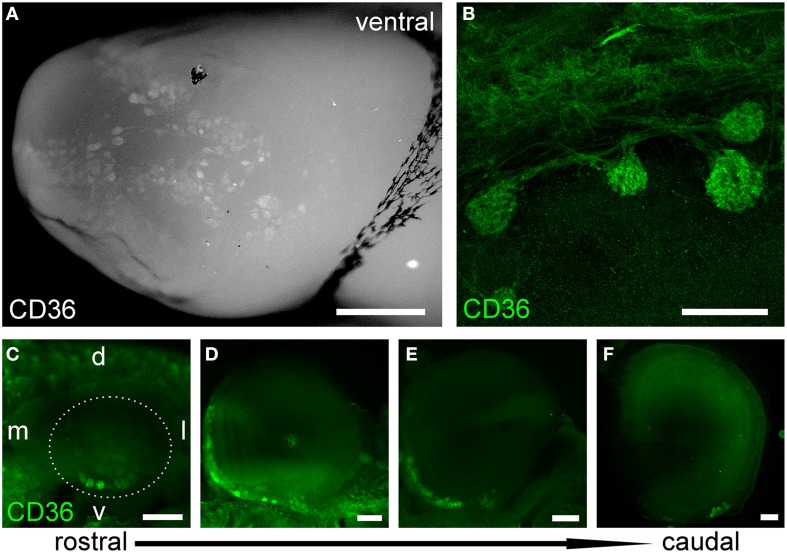
**CD36-positive glomeruli in the olfactory bulb. (A)** Fluorescence stereo microscopy image (maximum projection) of an olfactory bulb preparation (P9) immunostained for CD36. Ventral view onto the right bulb. Medial side: top; lateral side: bottom. **(B)** Confocal image (maximum projection) of a vibratome section immunostained for CD36 demonstrating CD36 staining in glomeruli and nerve fibers (P9). **(C–F)** Fluorescence stereo microscopy images of P8 vibratome sections from rostral to caudal regions of the olfactory bulb immunostained for CD36. Glomeruli are located in ventromedial OB regions. Dotted line encircles the OB in the first image. (d, dorsal; l, lateral; v, ventral; m, medial, consistent for **C–F**). Scale bars: 100 μm **(B)**, 200 μm **(C–F)**, 500 μm **(A)**.

Next, we immunostained whole-mount olfactory bulb preparations, and screened the surface for CD36-positive glomeruli. A large subset of glomeruli was labeled at different staining intensities (Figure 3A), clearly outnumbering two glomeruli per bulb typically observed for canonical olfactory neurons with defined receptor identity (Vassar et al., 1994). CD36-positive glomeruli were analyzed in more detail using vibratome slices (Figures 3B–F). CD36 was detected in nerve fibers and axon termini (Figure 3B). We analyzed the distribution of CD36-positive glomeruli from rostral to more caudal parts of the olfactory bulb and found CD36-positive glomeruli predominantly in the ventral and ventromedial region. CD36-positive glomeruli were essentially absent in the most caudal sections of the olfactory bulb (Figures 3C–F).

### CD36 neurons express olfactory signaling proteins

**Figure 4:**
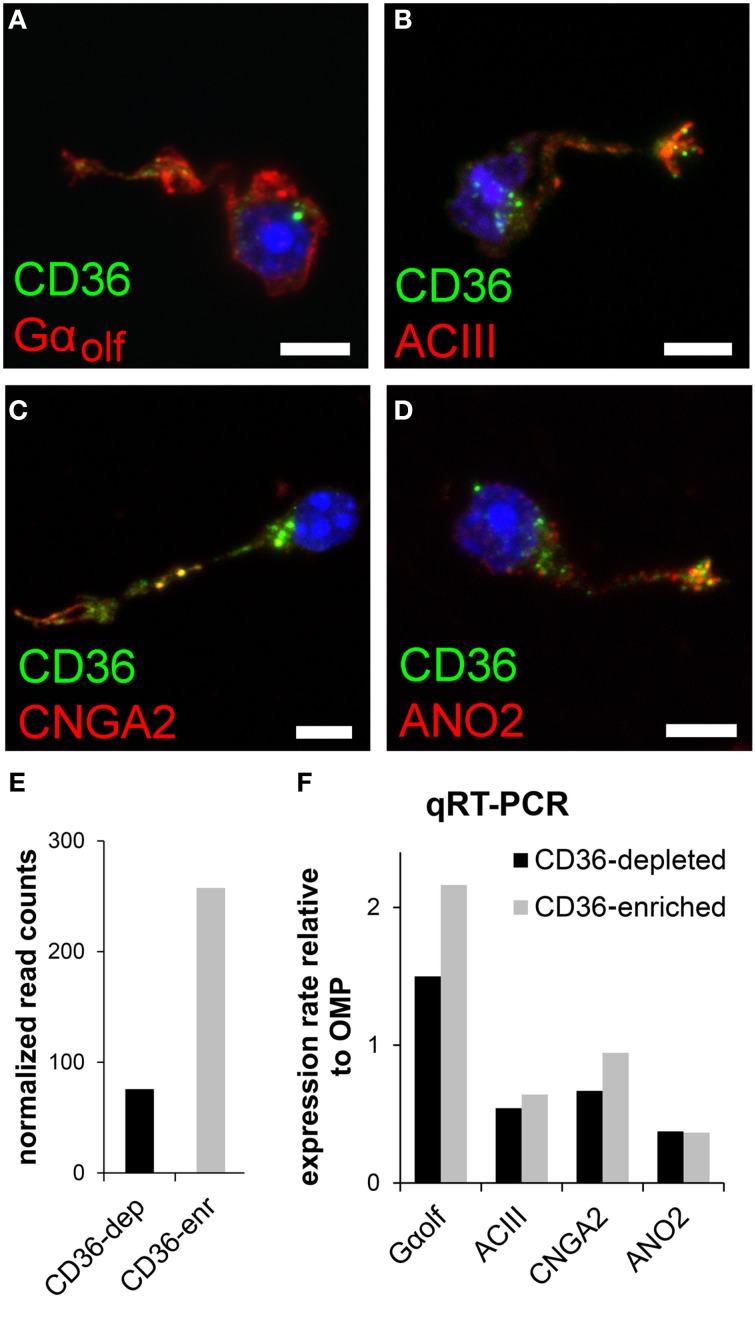
**CD36 neurons express olfactory signal transduction proteins. (A–D)** Confocal images of dissociated olfactory sensory neurons from P8 animals. Immunostainings show co-expression of CD36 (green) and Gα_olf_ **(A)**, ACIII **(B)**, CNGA2 **(C)**, and ANO2 **(D)** (red). **(E)** Normalized CD36 read counts (Trimmed Mean of *M*-values (TMM) method) for both cell fractions CD36-depleted (CD36-dep) and CD36-enriched (CD36-enr) used for transcriptome sequencing. Diagram illustrates the enhancement of CD36 (3.4 fold) in the CD36-enriched fraction (257.5) compared to the CD36-depleted fraction (75.7). **(F)** Quantitative real-time PCR results for Gα_olf_, ACIII, CNGA2, and ANO2 comparing CD36-enriched and CD36-depleted cell fraction from magnetic CD36 cell sorting in P8 animals. Data was normalized to OMP. Scale bars: 5 μm **(A–D)**.

We tested whether and, if so, which members of the olfactory transduction machinery are expressed in CD36-positive sensory neurons. Immunostaining of four canonical olfactory transduction proteins, Gα_olf_, ACIII, CNGA2, and ANO2, in dissociated olfactory neurons demonstrated co-expression in CD36-positive cells (Figures 4A–D). To further uncover the expression of signaling proteins in CD36-positive neurons, we performed quantitative real-time PCR and transcriptome sequencing. Dissociated neurons were sorted via a magnetic cell separation system, yielding in a fraction enriched in CD36 expressing neurons (Figure 4E). Quantitative real-time PCR of the sorted neurons confirmed the expression of Gα_olf_, ACIII, CNGA2, and ANO2 (Figure 4F). Also transcriptome sequencing of the CD36-enriched fraction showed transcripts for Gα_olf_, ACIII, CNGA2, and ANO2 together with mRNAs from other elements of the canonical olfactory signaling cascade (Gβ1, Gγ13, CNGA4, CNGB1, PDE1C, PDE4A, NKCC1, NCKX4, RTP1, RTP2, REEP1; Supplementary Table 1). To exclude proteins with low abundance, analysis was restricted to transcripts that showed both an expression level of at least 0.1% of ACIII. The transcriptome dataset from CD36 expressing neurons further showed expression of ORs, but not of TAARs or GUCY2D, the particulate guanylate cyclase expressed in CO_2_ responsive olfactory neurons.

### CD36 neurons express ORs

**Figure 5:**
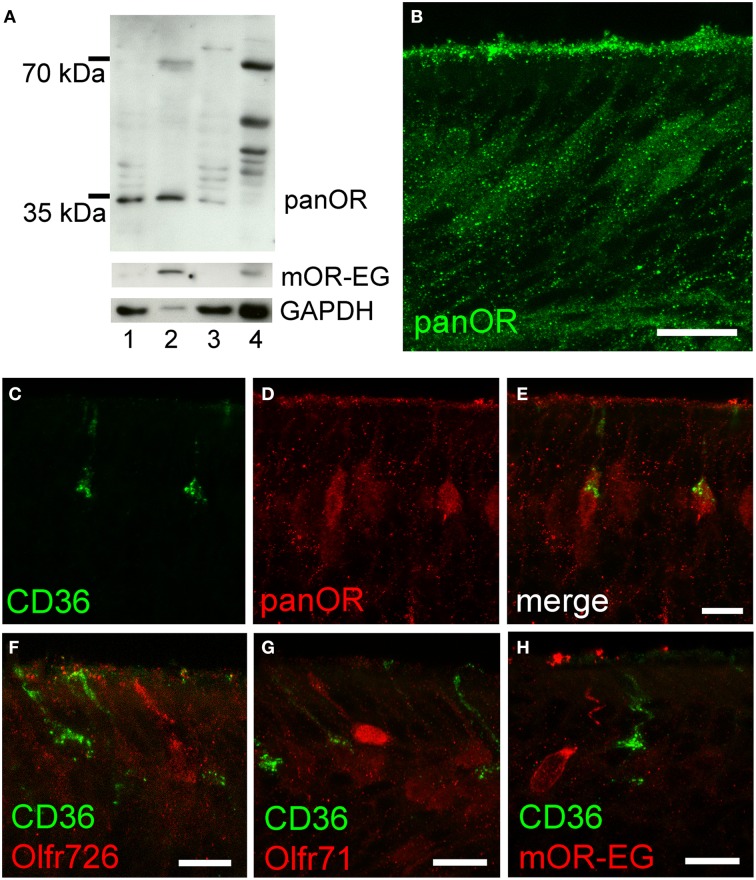
**CD36-positive neurons express olfactory receptors. (A)** Western blot analysis of olfactory epithelium (1), membrane preparation (2), and supernatant (3) of three P60 mice showing panOR specificity to olfactory epithelium. Adult liver (4) was used as negative control; absence of GAPDH shows successful membrane preparation; mOR-EG demonstrates an olfactory receptor-specific band of same size like the panOR band. **(B)** Confocal image (maximum projection) of a cryosection immunostained with the panOR antibody (P8). Scale bar: 20 μm. **(C–E)** Confocal images (maximum projections) of a P8 cryosection immunostained for CD36 (green) and panOR (red). **(F–H)** Confocal images of P8 cryosections immunostained for CD36 (green) and specific olfactory receptors Olfr726 **(F)**, Olfr71 **(G)**, and mOR-EG **(H)** (red). Scale bars: 20 μm **(B)**, 10 μm **(C–H)**.

To further analyze potential co-expression of ORs and CD36, we raised an antibody (panOR) against an amino acid sequence motif that is shared by most ORs. Similar to previous results, the non-transmembrane motif with the highest degree of conservation is MAYDRYVAIC at the transition between transmembrane domain 3 and intracellular loop 2. Although many class A GPCRs share similarity in the DRY motif, the MAY and VAIC sequences were specific for ORs and are not found in other GPCRs. Western blot analysis revealed a strong band in olfactory epithelium membrane preparations, but not in liver (Figure 5A). Immunostaining in cryosections showed labeling of the ciliary layer and many mature olfactory neurons (Figure 5B). The immunostaining partially overlapped with CD36 immunostaining: some neurons revealed strong OR and CD36 staining (Figures 5C–E). We then investigated expression of mOR-EG, Olfr71, and Olfr726 with specific antibodies, but never observed co-localization with CD36 (Figures 5F–H), indicating that individual ORs may be specifically co-expressed in CD36-positive neurons.

### CD36 is localized in olfactory cilia

**Figure 6:**
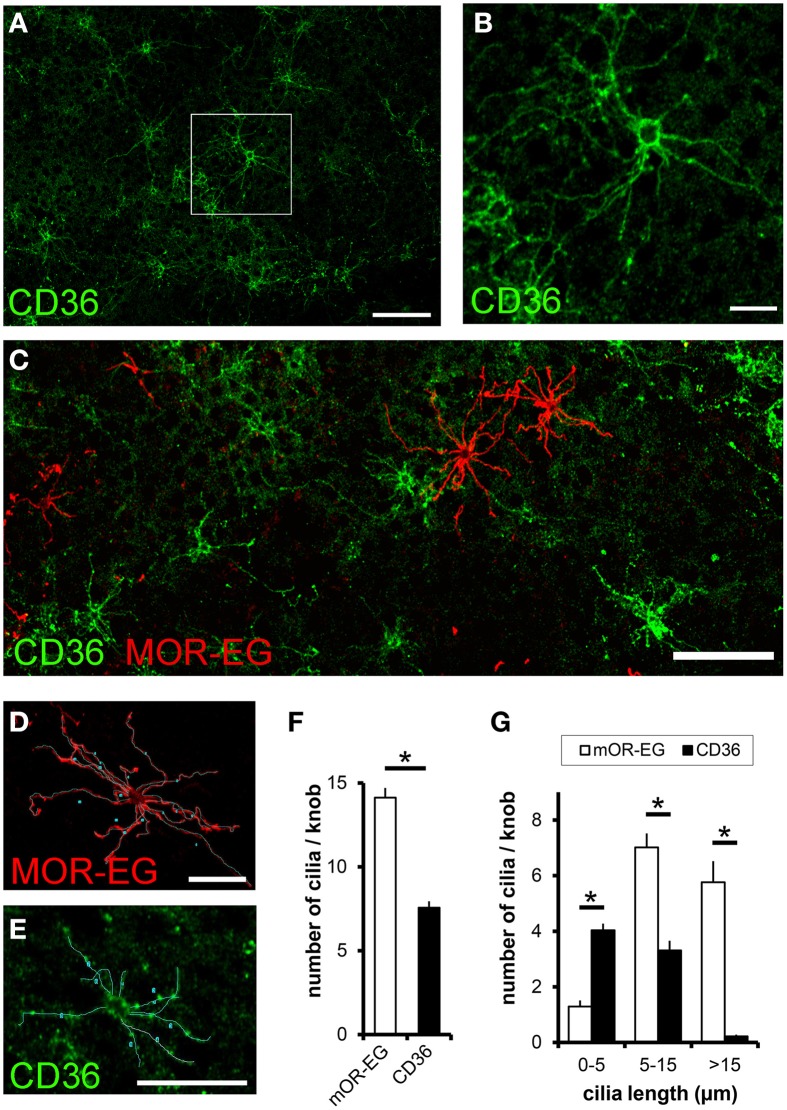
**CD36 is localized in olfactory cilia**. **(A)** Confocal image of a *en face* preparation immunostained for CD36 showing CD36 localization in olfactory cilia (P8). **(B)** Higher magnification of the boxed area in **(A)**. CD36 staining is prominent around the knob and along olfactory cilia. **(C)** Confocal image of an *en face* preparation immunostained for CD36 (green) and mOR-EG (red) showing differences in cilia length in both differently stained cell types, both proteins showed no co-expression. **(D)** Olfactory cilia counting: Confocal image of a top view preparation immunostained for CD36. Cilia were marked as demonstrated using a freehand tool from ImageJ. **(E)** Olfactory cilia counting: Confocal image of the same *en face* preparation shown in **(C)**, co-immunostained for mOR-EG. **(F)** Total number of cilia per knob for mOR-EG-positive and CD36-positive neurons. CD36-positive cells possess half as much cilia compared to mOR-EG expressing cells. Data are shown as mean ± SEM of 55 knobs in 3 individual preparations. Significance was calculated using two sample *t*-test (^*^p ≤ 0.05). **(G)** Number of cilia with different length of mOR-EG-positive and CD36-positive neurons. CD36-positive cilia are shorter compared to mOR-EG-positive cilia. Data are shown as mean ± SEM of 55 knobs in 3 individual preparations. Significance was calculated using Mann–Whitney-*U*-Test (^*^p < 0.01).

Since CD36 was co-expressed with canonical signal transduction proteins, we investigated whether CD36 was also localized in olfactory cilia. We used a preparation that allows an *en face* view onto the epithelial surface (Oberland and Neuhaus, 2014). CD36 was localized in a subset of olfactory knobs and cilia spreading from these (Figures 6A,B), but did not co-express with the olfactory receptor mOR-EG (Figure 6C). Moreover, labeling of CD36 and mOR-EG on the same preparation revealed morphological differences between both stained cell populations. To examine these differences, we analyzed 55 CD36-positive and 55 mOR-EG-positive olfactory neurons in co-stainings (from 3 animals) by counting cilia and measuring cilia length (Figures 6D,E). We counted 14 cilia per mOR-EG expressing neuron, which is comparable to earlier scanning electron microscopy studies showing an average of 17 cilia, each up to 60 μm long (Menco et al., 1997). CD36 expressing neurons extend only half as many cilia (CD36: 7.6 ± 0.4 m, Figure 6F). Moreover, mOR-EG-positive cilia were significantly longer (Figure 6G). CD36 expressing neurons therefore seem to differ from other olfactory neurons by morphological characteristics.

### CD36 showed a receptor-like expression pattern in cilia from olfactory neurons

**Figure 7:**
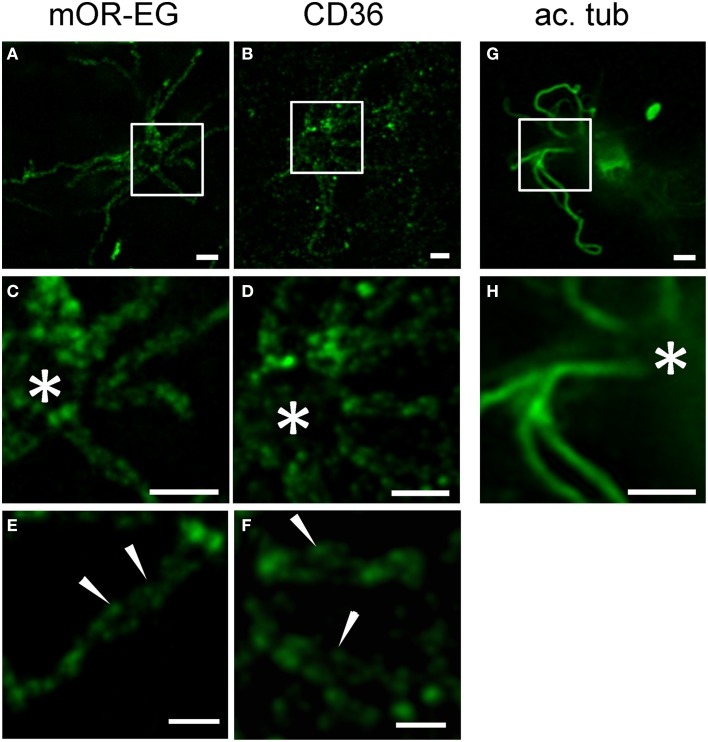
**STED microscopy revealed receptor like localization of CD36 in olfactory cilia. (A,B)** Shown are STED images of *en face* preparations immunostained for mOR-EG and CD36 (P8). **(C,D)** Higher magnifications of the cilia shown in **(A,B)**, respectively. The olfactory receptor mOR-EG and CD36 show a similar staining pattern. **(E,F)** High magnification pictures showing localization of CD36 and mOR-EG in discrete spots (examples are indicated by arrowheads). **(G,H)** STED images of dissociated adult neurons immunostained for acetylated tubulin (ac. tub). Scale bars: 1 μm **(A–D,G,H)**, 500 nm **(E,F)**. ^*^Marks the dendritic knob **(C,D,H)**.

Little is known about the spatial distribution of receptor proteins within olfactory cilia. Using stimulated emission depletion (STED) super-resolution microscopy, we recently showed that CNG channels and calcium-activated chloride channels of the Anoctamin family are localized to discrete microdomains in the ciliary membrane (Henkel et al., 2015). We here used STED microscopy to analyze mOR-EG and CD36 expression patterns in top view *en face* preparations (Figure 7). Similar to the ion channels, mOR-EG dissolved into distinct foci along the olfactory cilia under STED conditions (Figures 7A,C,E), while acetylated tubulin showed homogenous protein distribution (Figures 7G,H). This localization pattern provided first optical evidence for distinct spatial organization of olfactory receptors in cilia of sensory neurons. When analyzing CD36 staining pattern with STED microscopy, we found it also to be localized to discrete foci in the ciliary membrane (Figures 7B,D,F). Together, STED microscopy clearly provided morphological evidence for the organization of olfactory receptors in distinct domains along olfactory cilia. Since also the ion channels involved in odorant detection showed clustered localization pattern (Henkel et al., 2015), the presence in such microdomains seems to be a common feature of signaling proteins in olfactory cilia. The similarity in localization of CD36 is an indicator for a potential role of this fatty acid receptor in olfactory signal transduction.

### Absence of CD36 has no effect on general morphology of the olfactory system

**Figure 8:**
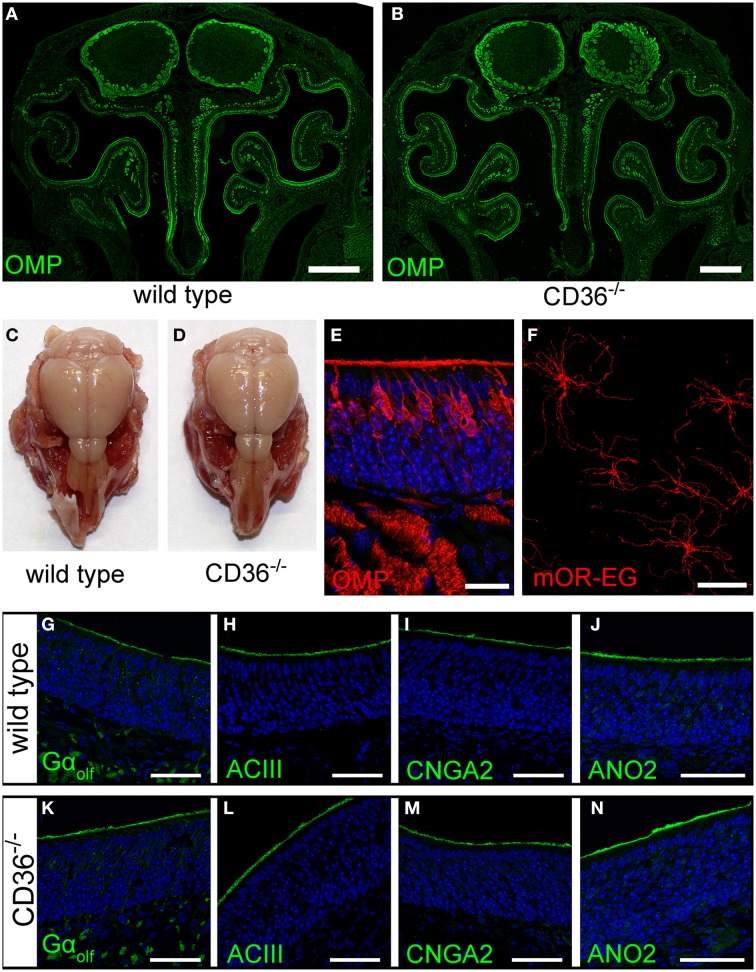
**Olfactory system of CD36 knockout mice. (A,B)** Widefield fluorescence microscopy images of P8 wild type **(A)** and CD36^−/−^ **(B)** cryosections immunostained for the mature olfactory neuron marker OMP. Olfactory bulb and turbinates show no morphological abnormalities. **(C,D)** Head preparation of wild type **(C)** and CD36^−/−^ **(D)** P90 animals showing no differences in OB and OE size and shape. **(E)** Confocal image (maximum projection) of a P8 CD36^−/−^ cryosection immunostained for OMP (red), CD36 (green) and counterstained with TO-PRO (blue) to visualize cell nuclei. CD36 protein is absent in the olfactory epithelium. **(F)** Confocal image of a P14 CD36^−/−^ *en face* preparation immunostained for the olfactory receptor mOR-EG. Immunostaining shows normal mOR-EG localization to olfactory knobs and cilia and unaltered ciliary length. **(G–N)** Confocal images (maximum projections) of wild type **(G–J)** and CD36^−/−^ **(K–N)** cryosections immunostained for classical olfactory transduction proteins Gα_olf_, ACIII, CNGA2, and ANO2 (green) and counterstained with TO-PRO (blue). Signal cascade proteins are mainly restricted to the ciliary layer in both wild type and CD36^−/−^ P8 animals and no abnormal protein distributions are observed. Scale bars: 20 μm **(E,F)**, 50 μm **(G–N)**, 500 μm **(A,B)**.

We further analyzed the potential role of CD36 in olfactory signaling in CD36 knockout (CD36^−/−^) mice (Febbraio et al., 1999). The gross anatomy of the nasal cavity and the olfactory bulb was unaltered (Figures 8A–D). Moreover, the sensory neuron morphology showed no differences compared to wild type mice (Figure 8E). We counted all OMP-positive sensory neurons in a 600 μm region of septal epithelium and detected no differences between wild type (224 neurons; *n* = 10 septum regions from 5 animals) and CD36^−/−^ mice (222 neurons; *n* = 8 septum regions from 4 animals). Ciliary morphology of mOR-EG expressing neurons was not altered in knockout animals (Figure 8F). To determine whether the amount or the localization of signaling proteins is changed in the CD36^−/−^ mice, we stained the olfactory epithelium for the olfactory signal transduction proteins G_olf_, ACIII, CNGA2, and ANO2 (Figures 8G–N) and performed quantitative PCR (data not shown). Both experiments revealed no changes in CD36^−/−^ mice compared to wild type mice.

### Responses to oleic acid are reduced in CD36^−/−^ animals

**Figure 9:**
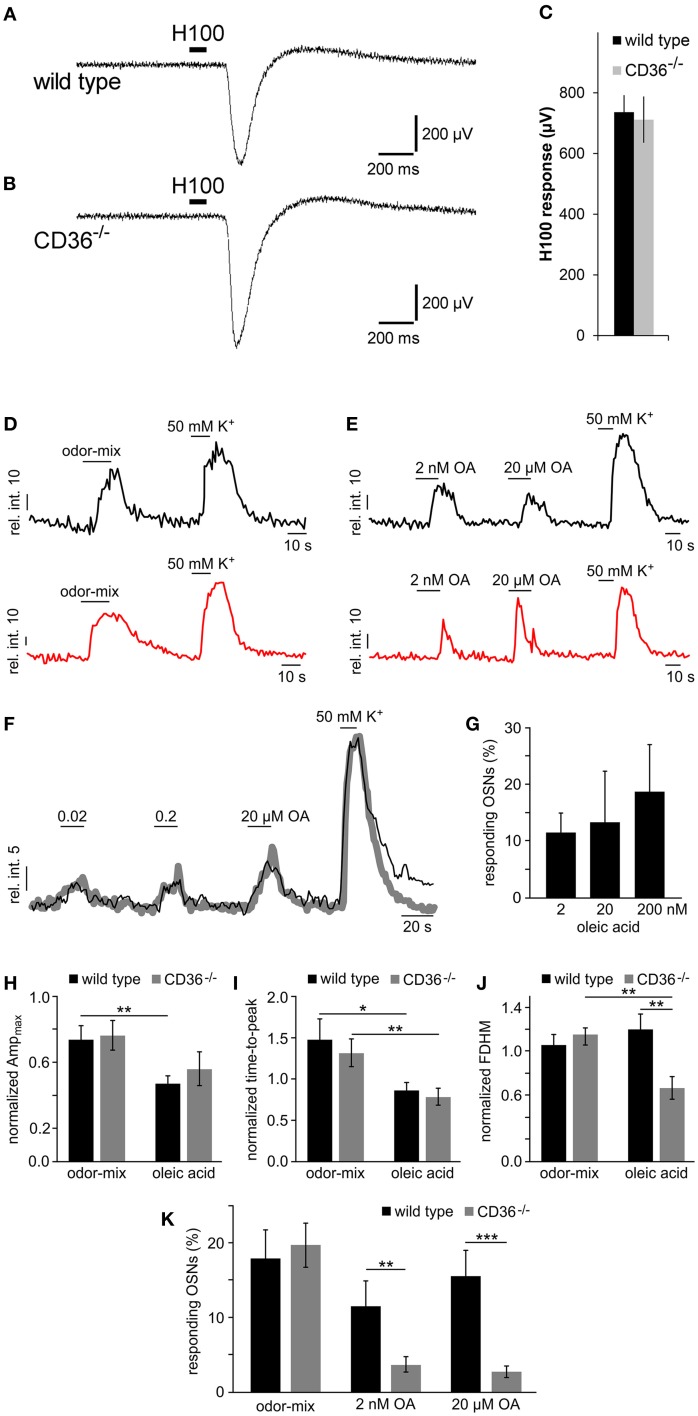
**Functional investigation of CD36 knockout mice. (A,B)** Representative traces of local field potentials (electro-olfactogram, EOG) generated in the main olfactory epithelium of wild type **(A)** and CD36^−/−^ **(B)** mice upon stimulation with a mixture of 100 odorants (Henkel 100). **(C)** Mean EOG responses to the odor mixture Henkel 100 from the main olfactory epithelium of wild type and CD36^−/−^ mice showing no significant differences. CD36^−/−^ mice possess normal ability to detect odorants. Data are shown as mean ± SEM of *n* = 6–8 mice at postnatal day 14 (3 individual recordings each). Significance was calculated using two sample *t*-test. Data are shown as mean ± SEM. **(D,E)** Representative traces (relative intensity vs. time) illustrating odor-, OA- and K^+^-dependent Ca^2+^ elevations in olfactory neurons from a wild type (black) and a CD36^−/−^ mouse (red). Horizontal bars indicate stimulus application. OA concentrations as indicated. **(F,G)** Dose-dependent Ca^2+^ elevations recorded from fluo-4/AM loaded OSNs in acute tissue sections from wild type mice. **(F)** Overlay of average Ca^2+^ signals (gray curve; 11 randomly selected neurons) and an original trace from a single OSN (black) in response to increasing oleic acid (OA) concentrations (0.02, 0.2, and 20 μM). Relative fluorescence intensity is plotted as a function of time. Horizontal bars indicate duration of stimulus application. **(G)** Bar graph illustrating the relative proportion of OA-sensitive neurons as a function of stimulus concentration [*n* = 27 (2 nM); *n* = 11 (20 nM); *n* = 11 (200 nM)]. Data are shown as mean ± SEM. **(H–J)** Bar charts comparing different response characteristics as a function of genotype [wild type (black) vs. CD36^−/−^ (gray)] and stimulus (odor-mix vs. OA). Signal parameters in response to odor (10 μM each) and OA (2 nM) are normalized to depolarization-dependent Ca^2+^ signals (K^+^; *n* = 11 randomly selected neurons). Maximum response amplitude [Ampmax (H)], rising speed [time-to-peak **(I)** and half-width; full duration at half maximum, FDHM **(J)**] are plotted. Asterisks denote statistical significance (unpaired, two-sided *t*-test; ^*^p ≤ 0.05; ^**^p ≤ 0.01; age P6–P9). Data are shown as mean ± SEM. **(K)** Bar diagram illustrating the portion of neurons responding to the odor-mix and to oleic acid, respectively, for wild type (black) and CD36^−/−^ (gray) animals. Cell count was normalized to K^+^-sensitive neurons in each experiment [wild type: *n* = 40 (odor-mix), 27 (OA, 2 nM), 37 (OA, 20 μM); CD36^−/−^: *n* = 26 (odor-mix), 40 (OA, 2 nM and 20 μM)]. Similar portions of neurons responded to the odor mix. By contrast, the number of OA sensing neurons was significantly reduced in CD36^−/−^ mice. Significance was calculated using unpaired, two-sided *t*-tests (^**^p ≤ 0.01; ^***^p < 0.005; age P6–P9). Data are shown as mean ± SEM.

The olfactory function in wild type and CD36^−/−^ mice was first analyzed by recording epithelial field potentials (electro-olfactogram, EOG), elicited by a complex odorant mixture. The signals were indistinguishable between wild type and CD36^−/−^ mice (Figures 9A–C), indicating that CD36^−/−^ mice are not generally anosmic.

By using confocal imaging in acute slices (Fluegge et al., 2012), we then recorded Ca^2+^ signals in response to odorant mixtures (Figure 9D). Brief odor stimulation triggered Ca^2+^ transients in a subpopulation of olfactory neurons in both, wild type and CD36^−/−^ mice, whereas K^+^-mediated depolarization activated most neurons. No difference in the number of responding cells was observed between wild type and CD36^−/−^ mice (Figure 9K). This observation confirms field potential recordings, which did not reveal a general olfactory deficit of CD36^−/−^ mice.

Together, our results show that loss of CD36 does not impair the development or the overall function of the olfactory system, but instead indicates a specific role for the CD36 expressing population of olfactory neurons. Given that CD36 has been described as a receptor for fatty acids (Baillie et al., 1996; Ibrahimi et al., 1996) we investigated responses to the known CD36 ligand oleic acid. Since oleic acid and other free fatty acids are rapidly oxidized, we kept the substances under inert gas to prevent formation of oxidation products. First, we analyzed whether olfactory neurons respond to fatty acids, which has not been shown before. We stimulated olfactory epithelium slices with oleic acid (2 nM and 20 μM) and recorded Ca^2+^ signals by confocal imaging (Figure 9E). A subset of ~10–15% of neurons from wild type mice responded to the ligand (Figure 9K), showing that oleic acid can indeed cause Ca^2+^ increases in olfactory neurons of young mice. These responses appear to be dose-dependent (Figures 9F,G). To analyze the role of CD36 in oleic acid-induced Ca2+ elevations we also recorded signals in neurons from CD36^−/−^ animals (Figure 9E) and compared various signal characteristics as well as the proportion of oleic acid-sensitive neurons in CD36^−/−^ to data from wild-type mice (Figures 9H–K). Oleic acid-induced Ca^2+^ responses not only displayed several differences in signal shape (amplitude, rise, half-width) as compared with odor-dependent signals (Figures 9H–J). Some oleic acid-specific signal parameters also changed with genotype (Figure 9J). Moreover, we found that the number of responding cells was significantly reduced in CD36^−/−^ mice (Figure 9K), indicating that CD36 plays a role for the detection of oleic acid by the main olfactory epithelium.

### Plasticity of CD36 expression during postnatal development

**Figure 10:**
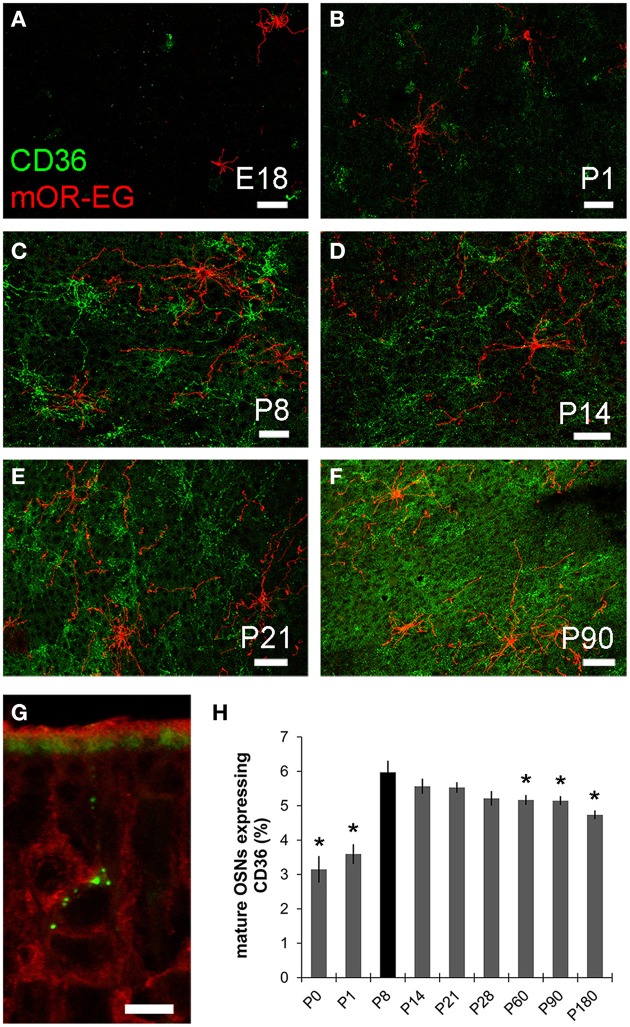
**CD36 localization is regulated. (A–F)** Confocal images of *en face* olfactory epithelium preparations for age stages E18-P90 immunostained for CD36 (green) and mOR-EG (red) showing localization changes of CD36 in olfactory cilia. mOR-EG immunostainings validate successful sample preparation and show clear ciliary localization at all age stages. CD36 ciliary localization is restricted to P8 **(C)** and P14 **(D)**. Before, it is found in single olfactory knobs, but barely in cilia (E18: **A**; P1: **B**) and later, ciliary localization decreases (P21: **E**) and is absent in adult animals (P90: **F**). **(G)** Confocal image (maximum projection) of a P90 cryosection immunostained for CD36 (green) and OMP (red). CD36 is still localized in OSN soma and denrite in adult animals. Additionally, CD36 is expressed in the microvilli layer of sustentacular cells. **(H)** Cell counting of CD36 and OMP expressing neurons in a stretch of 600 μm epithelium lining the septum. Cell numbers for CD36 were normalized to OMP expressing cells. If possible, both sides of every septum were counted (*n* = 2) to get a total number of 7–10. Significance was calculated in comparison to P8 using two sample *t*-test. Error bars represent SEM (^*^p ≤ 0.05). Scale bars, 10 μm **(A–F)**.

As oleic acid is a major milk component, we next analyzed ciliary localization of CD36 from prenatal to adult age (Figures 10A–F). Preparations were co-immunostained for mOR-EG to ensure integrity of the tissue. CD36 was localized to a subset of olfactory knobs and cilia spreading from these. While mOR-EG was localized to cilia at all ages, clear ciliary localization of CD36 was only observed from P8-P21 (Figures 10C–E). At P21, punctate CD36 staining appeared around several olfactory knobs, indicating that CD36 labeled cilia were disintegrated. The cilia of co-stained mOR-EG expressing neurons appeared unchanged, ruling out a general disassembly or reorganization of cilia at this age (higher magnification in Supplementary Figure 1). Some regions in the epithelium even completely lacked CD36 ciliary staining at P21. Around P28, punctate labeling declined strongly as well (data not shown), and completely disappeared in older animals. Ciliary CD36 staining was lacking in P90 animals (Figure 10F). A strong, relatively uniform staining with CD36 antibodies in adult animals, however, was derived from labeled microvilli of sustentacular cells (Figure 10G and RNA *in situ* hybridization, data not shown). Although ciliary CD36 labeling was progressively lost between P21 and P28, CD36 was still present within somata and dendrites of single neurons until adulthood (Figure 10G). We counted the relative amount of CD36 expressing neurons among mature OMP-positive neurons from E18 to P180. Analyzing whole cryosections, we found on average ~1.6% CD36 expressing neurons (11 ± 1/677 ± 44 per section) in E18 animals, ~8.0% at P8–P14 (328 ± 29/4098 ± 436), and gradually decreasing numbers during and post-puberty (Table 1). Although we counted 161,353 sensory neurons in total (11,304 of them CD36-positive), it was not feasible to perform statistical analyses with whole slice countings, as the total number of analyzed cryosections was too low with 1–3 sections per age. For statistical analysis, we therefore defined a 600 μm epithelial stretch along the septum and quantified CD36-positive relative to OMP-positive neurons (Figure 10H). Together, the percentage of CD36 expressing neurons significantly increases after birth until P8, and decreases moderately during further aging. Nevertheless, CD36 completely disappeared from the cilia when mice were weaned. We therefore hypothesize that the presence of CD36 in cilia of olfactory neurons during the suckling period plays an important role for adaptation to the particular needs during this phase of life.

**Table 1:** **Number of CD36 expressing cells in whole slices of age stages E18-P180**.

**Age**	**OMP + neurons**	**CD36 + neurons**	**CD36 + neurons/OMP + neurons**	**% CD36 + neurons (mean)**
E18	708	10	0.0141	1.64
E18	646	12	0.0186	
P0	1381	54	0,0391	3.73
P0	1122	39	0.0348	
P0	919	35	0.0381	
P1	1323	66	0.0499	4.90
P1	1050	55	0.0524	
P1	1182	53	0.0448	
P8	3556	285	0.0801	8.31
P8	3187	254	0.0797	
P8	3683	329	0.0893	
P14	5216	453	0.0868	7.92
P14	5672	356	0.0628	
P14	3276	288	0.0879	
P21	7460	618	0.0828	7.62
P21	9832	799	0.0813	
P21	10242	660	0.0644	
P28	9713	709	0.0730	7.49
P28	6190	474	0.0766	
P28	11202	841	0.0751	
P60	16022	1092	0.0682	7.50
P60	13568	1111	0.0819	
P90	12042	787	0.0654	6.45
P90	15427	981	0.0636	
P180	16734	943	0.0564	

*Data from single cryosections are presented. Cell counting in whole cryosections was performed using a cell counting plugin for ImageJ. OMP- and CD36-expressing neurons were manually marked and counted by the software. We normalized the amount of CD36 neurons to mature OMP expressing cells*.

## Discussion

Olfactory cues from foods are important for survival of mammals. We here describe a population of neurons in the olfactory epithelium of mice, which is characterized by the expression of the fatty acid receptor CD36 and has distinct morphological characteristics. Knockout of CD36 does not cause gross morphological or functional alterations in the olfactory system, but reduces the amount of oleic acid-sensitive olfactory neurons.

CD36 is expressed in the cell soma, dendrite, axon termini of olfactory neurons. Also the olfactory receptor proteins are localized not only in cilia, but also in axon termini in the olfactory bulb (Strotmann et al., 2004). Most notably, its expression in olfactory cilia guarantees direct contact to the inhaled air. High resolution STED microscopy showed a scattered distribution of CD36, similar to the localization of mOR-EG. Tubulin, on the other hand, was homogeneously distributed along the cilia. Previous Ca^2+^ measurements in frog cilia revealed discrete signaling events, providing evidence for transduction domains along the ciliary membrane (Castillo et al., 2007). Using high resolution STED microscopy, our observation of scattered mOR-EG expression supports the notion of signaling microdomains in olfactory cilia. An anti-TRPM5 antibody also labeled discrete spots in olfactory cilia (Lin et al., 2007), and CNGA2 had been shown to have a punctate distribution by electron microscopy (Matsuzaki et al., 1999) and by STED microscopy (Henkel et al., 2015). The fact that CD36 was localized in discrete spots in the cilia supports our hypothesis of it having a role in olfactory signal transduction.

CD36 is a membrane glycoprotein, which belongs to the scavenger receptor family. CD36 is not specific for the olfactory system but has pleiotropic physiological functions in immune defense, metabolism, and angiogenesis (Silverstein and Febbraio, 2009). CD36 is described as a fatty acid receptor and/or transporter of long-chain fatty acids. Oleic acid is one of the best described CD36 ligands exhibiting nanomolar affinity (Baillie et al., 1996). We show here that oleic acid can induce Ca^2+^ signals in olfactory neurons, which, in part, depend on CD36 expression. The fact that all key elements of canonical signal transduction, including olfactory receptors, were identified in CD36 neurons suggests a co-receptor function. We did not find evidence for TAAR expression in CD36 neurons (Supplementary Table 1), which is in accordance with the observation that TAAR-expressing sensory neurons converge on glomeruli within a dorsomedial domain of the olfactory bulb (Johnson et al., 2012; Pacifico et al., 2012), distinct from the domain where CD36 expressing neurons project to.

CD36 has already been implicated in fatty acid signaling in other sensory cells. Exposure to long-chain fatty acids causes a CD36-dependent Ca^2+^ rise in taste cells (El-Yassimi et al., 2008), resulting in attraction to lipid-rich food (Laugerette et al., 2005). In addition, the two G-protein coupled receptors GPR40 and GPR120, which are activated by medium and long chain fatty acids, are expressed in the taste buds, and knock-out mice showed a diminished preference for linoleic acid and oleic acid (Cartoni et al., 2010). Recent work on gustatory perception of dietary lipids suggests that CD36 could act as co-factor for these G protein-coupled receptors that mediate fatty acid preference (Gilbertson and Khan, 2014). Both receptors that have been described in the taste buds have not been detected in CD36 expressing neurons (Supplementary Table 1), or in whole olfactory epithelium (Ibarra-Soria et al., 2014; Kanageswaran et al., 2015). These receptors therefore likely do not contribute to fatty acid responses of olfactory neurons.

In addition, CD36 is involved in insect pheromone signaling. Many insect species express SNMP, a CD36 ortholog, as part of a multi-protein signaling complex on dendrites of those antennal neurons that recognize the fatty acid-derived pheromone *cis*-vaccenyl acetate (Rogers et al., 1997; Benton et al., 2007; Jin et al., 2008). SNMP seems to couple lipid-based extracellular ligands to signaling receptors in chemosensory communication (Benton et al., 2007), since SNMP is required by the receptor dimer Or67d/Orco to detect cis-vaccenyl acetate (Benton et al., 2007; Jin et al., 2008), and by the receptor dimer Or83c/Orco for normal responses to the fatty alcohol farnesol (Ronderos et al., 2014). It has been suggested that SNMP/CD36 may couple lipid-based extracellular ligands to signaling receptors in chemosensory communication by capturing fatty acids on the surface of OSN cilia and facilitating their transfer to the odorant-receptor (Benton et al., 2007). CD36 was also previously shown to act as a co-receptor for Toll-like receptors (Hoebe et al., 2005; Jimenez-Dalmaroni et al., 2009), suggesting that CD36-related proteins could have transmembrane partners in all their cellular roles. Alternatively, CD36 may function itself as a receptor by interacting with non-receptor tyrosine kinases (Moore et al., 2002). So far, the precise molecular function remains unclear in all sensory systems studied so far. The mechanistic basis of CD36 ligand interactions and signaling is still poorly understood in any biological system. It is tempting to speculate that the co-expressed olfactory receptors may be involved in fatty acid discrimination since humans, and possibly other mammals, can discriminate vapor-phase long-chain fatty acids (Bolton and Halpern, 2010). Whether CD36 also facilitates the recognition of other fatty acids remains to be elucidated.

Remarkably, CD36 is markedly localized to olfactory cilia during the first days of life, and largely disappears from cilia after weaning. This temporally restricted localization of CD36 in the signaling compartment coincides with the presence of high ligand concentrations, since oleic acid is an abundant fatty acid in breast milk. The exact concentration of oleic acid in vapor phase entering the nasal cavity during suckling is unknown. The concentration of oleic acid in milk (~200 mM, roughly inferred from approximately 6–8% oleic acid in milk, depending on the maternal diet, Baillie et al., 1996) is far above the concentrations that elicited oleic acid responses in our study (2 and 20 nM). During the application of oleic acid to the main olfactory epithelium (Ca^2+^ imaging experiments) we cannot prevent access of the ligand to ambient oxygen, which leaves the possibility that small amounts of the applied oleic acid could be oxidized. Since the substance was always kept under inert gas until the final use in the experiments, we are convinced that oxidation products of oleic acid are only present in trace amounts. Interestingly, oleic acid triggers Ca^2+^ signals that appear somewhat different in shape that responses to conventional odors. It is thus possible that CD36-dependent transduction varies from canonical odor signaling. Future studies will have to address such potential mechanistic differences.

The temporally restricted localization in the signaling compartment is reminiscent to recently published data showing that daily postnatal exposure to lyral induces plasticity in the population of OSNs expressing MOR23 (Cadiou et al., 2014). Although intracellular protein distribution was not analyzed, MOR23 neurons have higher levels of olfactory receptor transcripts density after odorant exposure (Cadiou et al., 2014). It is possible to investigate in future studies whether developmentally regulated splicing accounts for the variable ciliary localization, since several splice variants of CD36 affecting the coding region of the protein have been described (Tang et al., 1994; Andersen et al., 2006).

Olfactory neurons expressing the same olfactory receptor, although randomly dispersed within a broad zone of the epithelium, project axons to two spatially invariant glomeruli in each olfactory bulb (Mori and Sakano, 2011). A marked difference was observed with regard to the number of labeled glomeruli since we found approximately 60–80 CD36 glomeruli clustered on the ventromedial part of the olfactory bulb. Similarly, GC-D expressing neurons terminate in more than two (about 10–20) “necklace” glomeruli encircling the caudal main olfactory bulb (Juilfs et al., 1997; Hu et al., 2007). Previous analysis of the rat pup olfactory bulb during suckling behavior revealed an increase in 2-deoxy-glucose incorporation in a subset of unusually shaped glomeruli, suggesting that receptors projecting to these specialized glomeruli are responsive to a specialized suckling odor cue (Teicher et al., 1980). Although, the position of these glomeruli is not identical to those positive for CD36, there could be substantial overlap. In both cases, significant labeling is observed on the ventromedial bulb. Interestingly, glomeruli innervated by different mOR37-positive neurons are also clustered in the ventral domain of the olfactory bulb (Strotmann et al., 2000). However, using transcriptome profiling, we did not find Olfr37 family members. Also TRPM5-expressing OSNs project axons to glomeruli in the ventral olfactory bulb (Lin et al., 2007), and apparently respond to social or sexual chemosignals, but not to regular odors (Lopez et al., 2014). It has therefore been suggested that the ventromedial/ventrolateral area of the olfactory bulb, in part, constitutes an “olfactory fovea” critical in processing information on semiochemicals (Xu et al., 2005; Lin et al., 2007).

High percentages of completely anosmic mice die shortly after birth, but the survival rate of these mice can be enhanced by reducing the litter sizes (Brunet et al., 1996; Belluscio et al., 1998; Wong et al., 2000). CD36^−/−^ pups do not die due to malnutrition or dehydration since initiation of suckling is dependent on variable blends of maternal “signature odors” that are learned and recognized prior to first suckling (Logan et al., 2012), but not single odor cues such as oleic acid.

## Conclusions

We here identified a novel subset of sensory neurons in the main olfactory epithelium of mice, characterized by expression of CD36. CD36 shows a receptor-like spatial arrangement in olfactory cilia of nursed mice in high resolution STED microscopy, but is redistributed to intracellular sites after weaning. In accordance with the described roles of CD36 as fatty acid receptor or co-receptor in other sensory systems, absence of CD36 causes defects in the recognition of oleic acid by olfactory neurons in young mice. The described dramatic intracellular re-localization of CD36 after weaning constitutes a remarkable example of the main olfactory system plasticity in the first days of life.

## Author note

Recently, another study also reported expression of CD36 mRNA and protein in olfactory sensory neurons and sustentacular cells of 8–12 week old wild-type mice (Lee et al., PLoS ONE 10:e0133412).

## Author contributions

Experiments were performed in the laboratories of MS and EN. SO and EN initiated the study. Research was designed by SO, TA, MS, EN. Data were collected by SO, TA, SG, TP, SP and analyzed by all authors. The manuscript was written by SO and EN (with assistance from TA and MS).

## Funding

This work was supported by the Deutsche Forschungsgemeinschaft (SPP1392, Exc257, SFB958) and the Volkswagen Foundation. MS is a Lichtenberg Professor of the Volkswagen Foundation.

### Conflict of interest statement

The authors declare that the research was conducted in the absence of any commercial or financial relationships that could be construed as a potential conflict of interest.

## Supplementary material

Supplementary Figure 1 **En-face preparations of the indicated ages, staining against CD36 (as in Figure 10)**.

Supplementary Table 1 **Results of transcriptome sequencing of CD36 expressing cells**. Transcripts that were identified in the transcriptome datasets from CD36-positive neurons from P8 animals. Only transcripts with more than 10 raw read counts are displayed. The table displays TMM-normalized read counts.

## Abbreviations

TAAR: trace amine associated receptor
CNG: cyclic nucleotide gated
ACIII: adenylate cyclase type III
CD36: cluster of differentiation 36
GC-D: guanylate cyclase D
cVA: *cis*-vaccenyl acetate (cVA)
OMP: olfactory marker protein.
