# Supplementary figures and images for: CD36 is involved in oleic acid detection by the murine olfactory system
Source: Front Cell Neurosci. 2015 Sep 16;9:366. doi: 10.3389/fncel.2015.00366 (PMC4584952; doi:10.3389/fncel.2015.00366)

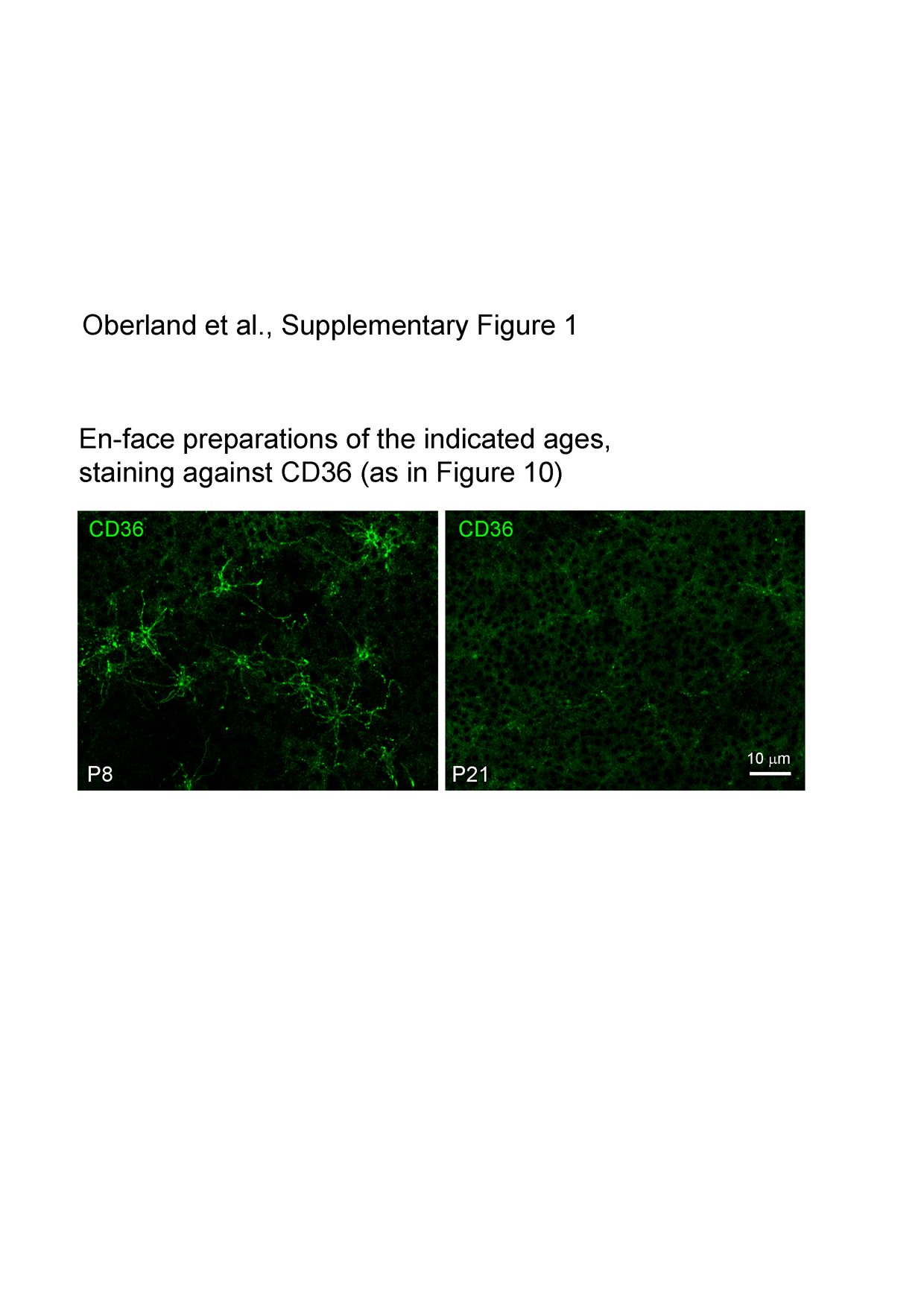

Supplement: Supplementary Figure 1 — En-face preparations of the indicated ages, staining against CD36 (as in Figure 10). [file Image1.TIF]
