# Supplementary material for: CD36 is involved in oleic acid detection by the murine olfactory system
Source: Front Cell Neurosci. 2015 Sep 16;9:366. doi: 10.3389/fncel.2015.00366 (PMC4584952; doi:10.3389/fncel.2015.00366)
Supplement: Supplementary Table 1 — Results of transcriptome sequencing of CD36 expressing cells. Transcripts that were identified in the transcriptome datasets from CD36-positive neurons from P8 animals. Only transcripts with more than 10 raw read counts are displayed. The table displays TMM-normalized read counts. [file Table1.PDF]

Oberland et al., Table 1

Gene		ENSMUSG	Length	normCount
Cyp2a5	cytochrome P450, family 2, subfamily a, polypeptide 5	ENSMUSG00000005547	8244	10640,78
Eef1a1	eukaryotic translation elongation factor 1 alpha 1	ENSMUSG00000037742	10703	6552,40
Tmsb4x	thymosin, beta 4, X chromosome	ENSMUSG00000049775	2223	4806,61
SEC14-like 3		ENSMUSG00000054986	12896	4399,22
Map1b	microtubule-associated protein 1B	ENSMUSG00000052727	95139	3637,64
mt-Atp6	Mitochondrially encoded ATP synthase 6	ENSMUSG00000064357	681	3611,90
mt-Co2	Mitochondrially encoded cytochrome c oxidase II	ENSMUSG00000064354	684	2891,57
Galnt2	UDP-N-acetyl-alpha-D-galactosamine:polypeptide N-acetylgalactosaminyltransferase 2	ENSMUSG00000092329	4434882	2798,42
Sult1c1	sulfotransferase family, cytosolic, 1C, member 1	ENSMUSG00000023943	29060	2773,26
mt-Co1		ENSMUSG00000064351	1545	2629,44
Bpifa1	BPI fold containing family A, member 1	ENSMUSG00000027483	6340	2601,82
mt-Nd2	Mitochondrially encoded NADH dehydrogenase 2	ENSMUSG00000064345	1038	2498,31
Ptma	prothymosin alpha	ENSMUSG00000026238	3963	2480,69
Myo3a	myosin IIIA	ENSMUSG00000025716	390750	2452,56
Tpt1	Tumor protein, translationally-controlled 1	ENSMUSG00000060126	3433	2403,49
Sdk1	sidekick homolog 1 (chicken)	ENSMUSG00000039683	974097	2396,96
mt-Nd1	Mitochondrially encoded NADH dehydrogenase 1	ENSMUSG00000064341	957	2202,26
Actg1	actin, gamma, cytoplasmic 1	ENSMUSG00000062825	2853	2169,63
Auts2	Autism susceptibility candidate 2	ENSMUSG00000029673	1106012	2048,72
Cyp2g1	cytochrome P450, family 2, subfamily g, polypeptide 1	ENSMUSG00000049685	12271	1963,25
Jun	Jun oncogene	ENSMUSG00000052684	3189	1944,62
mt-Nd4	Mitochondrially encoded NADH dehydrogenase 4	ENSMUSG00000064363	1378	1918,24
Cntn4	contactin 4	ENSMUSG00000064293	1021651	1726,86
Egr1	Early growth response 1	ENSMUSG00000038418	5162	1698,67
Tuba1a	tubulin, alpha 1A	ENSMUSG00000072235	3711	1649,44
Atf5	activating transcription factor 5	ENSMUSG00000038539	4402	1643,86
Fstl5	follistatin-like 5	ENSMUSG00000034098	635750	1640,82
Actb	actin, beta	ENSMUSG00000029580	3640	1633,28
Ugt2a1	UDP glucuronosyltransferase 2 family, polypeptide A1	ENSMUSG00000029268	31382	1619,22
Vmp1	vacuole membrane protein 1	ENSMUSG00000018171	99972	1587,54
Nrxn1	neurexin I	ENSMUSG00000024109	1059162	1584,28
Ebf1	early B cell factor 1	ENSMUSG00000057098	390775	1575,87
Rplp1	ribosomal protein, large, P1	ENSMUSG00000007892	1255	1557,16
Hspa8	heat shock protein 8	ENSMUSG00000015656	4217	1542,30
Tshz2	teashirt zinc finger family member 2	ENSMUSG00000047907	438804	1522,30
Gm5148	predicted gene 5148	ENSMUSG00000058174	10301	1516,42
Hsp90ab1	heat shock protein 90 alpha (cytosolic), class B member 1	ENSMUSG00000023944	5497	1474,31

mt-Cytb	Mitochondrially encoded cytochrome b	ENSMUSG00000064370	1144	1470,54
mt-Co3	Mitochondrially encoded cytochrome c oxidase III	ENSMUSG00000064358	784	1469,74
Ubc	ubiquitin C	ENSMUSG00000008348	4238	1375,94
Svopl	SV2 related protein homolog (rat)-like	ENSMUSG00000029830	63258	1375,58
Cdc37l1	cell division cycle 37-like 1	ENSMUSG00000024780	27076	1367,31
Tubb5	tubulin, beta 5 class I	ENSMUSG00000001525	4381	1354,41
Cbr2	carbonyl reductase 2	ENSMUSG00000025150	2626	1352,31
Rpsa-ps10	ribosomal protein SA, pseudogene 10	ENSMUSG00000047676	1031	1344,99
Gm7536	Predicted gene 7536	ENSMUSG00000057036	507	1328,02
Ebf2	early B cell factor 2	ENSMUSG00000022053	197627	1289,60
Por	P450 (cytochrome) oxidoreductase	ENSMUSG00000005514	65294	1282,93
Tubb2b	tubulin, beta 2B class IIB	ENSMUSG00000045136	3347	1272,78
Rimklb	ribosomal modification protein rimK-like family member B	ENSMUSG00000040649	39018	1241,69
Msi2	musashi RNA-binding protein 2	ENSMUSG00000069769	379132	1233,20
Tenm4	teneurin transmembrane protein 4	ENSMUSG00000048078	739850	1218,78
		ENSMUSG00000064368	519	1195,00
Fos	FBJ osteosarcoma oncogene	ENSMUSG00000021250	3384	1178,91
Tenm2	teneurin transmembrane protein 2	ENSMUSG00000049336	1229309	1173,98
Gadl1	glutamate decarboxylase-like 1	ENSMUSG00000056880	166722	1165,72
Tcf4	transcription factor 4	ENSMUSG00000053477	343822	1164,63
Chd9	chromodomain helicase DNA binding protein 9	ENSMUSG00000056608	225674	1162,24
Kif5b	kinesin family member 5B	ENSMUSG00000006740	41173	1141,79
Rpl5	ribosomal protein L5	ENSMUSG00000058558	8504	1133,17
Rpl7	ribosomal protein L7	ENSMUSG00000043716	3368	1125,41
Pla2g4c	phospholipase A2, group IVC (cytosolic, calcium-independent)	ENSMUSG00000033847	36018	1117,51
Lima1	LIM domain and actin binding 1	ENSMUSG00000023022	96959	1115,84
H3f3b	H3 histone, family 3B	ENSMUSG00000016559	6051	1110,55
Eef2	eukaryotic translation elongation factor 2	ENSMUSG00000034994	5877	1101,42
Clstn1	calsyntenin 1	ENSMUSG00000039953	62432	1093,81
Ptprf	protein tyrosine phosphatase, receptor type, F	ENSMUSG00000033295	83193	1087,72
Cadm1	cell adhesion molecule 1	ENSMUSG00000032076	327465	1067,42
Dusp1	dual specificity phosphatase 1	ENSMUSG00000024190	2930	1058,36
		ENSMUSG00000039542	294562	1055,46
Elavl3	ELAV (embryonic lethal, abnormal vision, Drosophila)-like 3 (Hu antigen C)	ENSMUSG00000003410	37019	1053,79
Trim66	tripartite motif-containing 66	ENSMUSG00000031026	59129	1024,72
Calm1	calmodulin 1	ENSMUSG00000001175	10372	1016,24
Mecom	MDS1 and EVI1 complex locus	ENSMUSG00000027684	596710	1012,91
Ank3	ankyrin 3, epithelial	ENSMUSG00000069601	493667	1004,79
Sod1	superoxide dismutase 1, soluble	ENSMUSG00000022982	5581	994,78
Zc3h7a	zinc finger CCCH type containing 7 A	ENSMUSG00000037965	39802	985,43
Rps9	ribosomal protein S9	ENSMUSG00000006333	2909	976,52
Aox2	aldehyde oxidase 2	ENSMUSG00000079554	100939	967,60
Wdr89	WD repeat domain 89	ENSMUSG00000045690	38944	963,90
Rpl41	ribosomal protein L41	ENSMUSG00000093674	1192	962,24

Hs3st5	heparan sulfate (glucosamine) 3-O-sulfotransferase 5	ENSMUSG00000044499	327584	953,03
Rpl26	ribosomal protein L26	ENSMUSG00000060938	5407	951,94
Ssh2	slingshot homolog 2 (Drosophila)	ENSMUSG00000037926	243934	929,18
		ENSMUSG00000028691	15279	922,15
Mbnl2	muscleblind-like 2	ENSMUSG00000022139	156029	915,34
Rnf157	ring finger protein 157	ENSMUSG00000052949	76680	912,65
Krt18	keratin 18	ENSMUSG00000023043	3811	905,84
Trpm3	transient receptor potential cation channel, subfamily M, member 3	ENSMUSG00000052387	850766	888,15
Rps4x	ribosomal protein S4, X-linked	ENSMUSG00000031320	4454	887,93
Zranb3	zinc finger, RAN-binding domain containing 3	ENSMUSG00000036086	148864	883,08
Ctnnd2	catenin (cadherin associated protein), delta 2	ENSMUSG00000022240	856719	881,99
Epcam	epithelial cell adhesion molecule	ENSMUSG00000045394	15128	873,51
Fgfr2	fibroblast growth factor receptor 2	ENSMUSG00000030849	2960900	869,74
Nfib	nuclear factor I/B	ENSMUSG00000008575	415578	869,23
Spag9	sperm associated antigen 9	ENSMUSG00000020859	129995	858,65
Zfhx3	zinc finger homeobox 3	ENSMUSG00000038872	246993	856,84
Pard3	par-3 (partitioning defective 3) homolog (C. elegans)	ENSMUSG00000025812	548394	854,52
Foxp1	forkhead box P1	ENSMUSG00000030067	597384	854,30
		ENSMUSG00000021546	12202	852,41
		ENSMUSG00000019505	2043	846,98
Sec14l2	SEC14-like 2 (S. cerevisiae)	ENSMUSG00000003585	26377	818,34
Atp5b	ATP synthase, H+ transporting mitochondrial F1 complex, beta subunit	ENSMUSG00000025393	7119	811,31
Marcks	myristoylated alanine rich protein kinase C substrate	ENSMUSG00000069662	5546	805,37
Ctnna2	catenin (cadherin associated protein), alpha 2	ENSMUSG00000063063	1098063	796,81
Papss2	3'-phosphoadenosine 5'-phosphosulfate synthase 2	ENSMUSG00000024899	47183	790,14
Pcdh7	protocadherin 7	ENSMUSG00000029108	411331	788,77
Rps8	ribosomal protein S8	ENSMUSG00000047675	2417	780,00
Gnb1	guanine nucleotide binding protein (G protein), beta 1	ENSMUSG00000029064	67909	779,63
Gphn	gephyrin	ENSMUSG00000047454	458113	774,85
Stmn1	stathmin 1	ENSMUSG00000028832	5524	773,25
Zbtb20	zinc finger and BTB domain containing 20	ENSMUSG00000022708	766722	771,66
Myo6	myosin VI	ENSMUSG00000033577	146699	769,92
Grip1	glutamate receptor interacting protein 1	ENSMUSG00000034813	633228	767,74
Atrx	alpha thalassemia/mental retardation syndrome X-linked homolog (human)	ENSMUSG00000031229	131783	759,19
Hspa5	heat shock protein 5	ENSMUSG00000026864	4562	754,70
Lphn3	latrophilin 3	ENSMUSG00000037605	804996	746,07
		ENSMUSG00000060438	498	745,71
Socs2	suppressor of cytokine signaling 2	ENSMUSG00000020027	31819	742,81
Stmn2	stathmin-like 2	ENSMUSG00000027500	52078	740,85
Rplp0	ribosomal protein, large, P0	ENSMUSG00000067274	4261	734,47
Rfk	riboflavin kinase	ENSMUSG00000024712	7307	733,38

Abca13	ATP-binding cassette, sub-family A (ABC1), member 13	ENSMUSG00000004668	492318	731,43
Ddx5	DEAD (Asp-Glu-Ala-Asp) box polypeptide 5	ENSMUSG00000020719	8831	731,21
Pclo	piccolo (presynaptic cytomatrix protein)	ENSMUSG00000061601	345267	730,85
Boll	bol, boule-like (Drosophila)	ENSMUSG00000025977	114812	728,02
Clstn2	calsyntenin 2	ENSMUSG00000032452	588787	727,08
Mgat5	mannoside acetylglucosaminyltransferase 5	ENSMUSG00000036155	278719	723,60
Robo2	roundabout homolog 2 (Drosophila)	ENSMUSG00000052516	519520	718,60
Son	Son DNA binding protein	ENSMUSG00000022961	31716	718,09
Cdk14	cyclin-dependent kinase 14	ENSMUSG00000028926	616922	714,39
Arih1	ariadne ubiquitin-conjugating enzyme E2 binding protein homolog 1 (Drosophila)	ENSMUSG00000025234	98361	712,94
Soga2	SOGA family member 2	ENSMUSG00000052105	112769	711,13
Abr	active BCR-related gene	ENSMUSG00000017631	205581	710,11
Gabarapl1	gamma-aminobutyric acid (GABA) A receptor-associated protein-like 1	ENSMUSG00000030161	9132	709,90
Tuba1b	tubulin, alpha 1B	ENSMUSG00000023004	3141	708,23
Dstn	destrin	ENSMUSG00000015932	28005	708,01
Thsd7b	thrombospondin, type I, domain containing 7B	ENSMUSG00000042581	945977	706,35
Wwp2	WW domain containing E3 ubiquitin protein ligase 2	ENSMUSG00000031930	122197	705,55
Dpysl3	dihydropyrimidinase-like 3	ENSMUSG00000024501	117308	702,94
Rps3	ribosomal protein S3	ENSMUSG00000030744	5843	701,85
Ncoa1	nuclear receptor coactivator 1	ENSMUSG00000020647	229820	696,05
Cltc	clathrin, heavy polypeptide (Hc)	ENSMUSG00000047126	63215	694,31
Cdh1	cadherin 1	ENSMUSG00000000303	66896	693,30
Shroom3	shroom family member 3	ENSMUSG00000029381	281884	692,72
Nbea	neurobeachin	ENSMUSG00000027799	558497	689,67
Nlk	nemo like kinase	ENSMUSG00000017376	130206	687,64
Atp1a1	ATPase, Na+/K+ transporting, alpha 1 polypeptide	ENSMUSG00000033161	28385	678,73
Lrrc58	leucine rich repeat containing 58	ENSMUSG00000034158	20470	678,29
Ebf3	early B cell factor 3	ENSMUSG00000010476	120773	678,00
Gstm1	glutathione S-transferase, mu 1	ENSMUSG00000058135	5719	676,91
Atp6v0c	ATPase, H+ transporting, lysosomal V0 subunit C	ENSMUSG00000024121	5837	676,41
Hint1	histidine triad nucleotide binding protein 1	ENSMUSG00000020267	4119	675,46
Cpm	carboxypeptidase M	ENSMUSG00000020183	57853	668,58
		ENSMUSG00000021699	1503514	666,55
Kalrn	kalirin, RhoGEF kinase	ENSMUSG00000061751	599275	662,49
		ENSMUSG00000031029	8535	661,55
Srrm2	serine/arginine repetitive matrix 2	ENSMUSG00000039218	21553	660,02
Rere	arginine glutamic acid dipeptide (RE) repeats	ENSMUSG00000039852	340321	657,99
Gnao1	guanine nucleotide binding protein, alpha O	ENSMUSG00000031748	159423	656,40
		ENSMUSG00000027359	35837	651,98
Mgea5	meningioma expressed antigen 5 (hyaluronidase)	ENSMUSG00000025220	33260	649,08

		ENSMUSG00000005299	43145	647,05
Bmpr1b	bone morphogenetic protein receptor, type 1B	ENSMUSG00000052430	332290	646,40
Gm10073	predicted pseudogene 10073	ENSMUSG00000060019	496	644,00
Prdx6	peroxiredoxin 6	ENSMUSG00000026701	11108	643,21
Setbp1	SET binding protein 1	ENSMUSG00000024548	359012	642,26
		ENSMUSG00000040681	7112	641,39
Ptprg	protein tyrosine phosphatase, receptor type, G	ENSMUSG00000021745	688510	634,15
Klf6	Kruppel-like factor 6	ENSMUSG00000000078	8905	630,96
Gnaq	guanine nucleotide binding protein, alpha q polypeptide	ENSMUSG00000024639	254633	630,38
Tubb4b	tubulin, beta 4B class IVB	ENSMUSG00000036752	2543	629,94
Hist2h2be	histone cluster 2, H2be	ENSMUSG00000068854	2618	623,34
Skint6	selection and upkeep of intraepithelial T cells 6	ENSMUSG00000087194	482358	616,10
Myh9	myosin, heavy polypeptide 9, non-muscle	ENSMUSG00000022443	81589	615,73
Tmem66	transmembrane protein 66	ENSMUSG00000031532	16283	615,15
Cacnb2	calcium channel, voltage-dependent, beta 2 subunit	ENSMUSG00000057914	383603	613,92
Ece1	endothelin converting enzyme 1	ENSMUSG00000057530	102993	613,05
Kif1b	kinesin family member 1B	ENSMUSG00000063077	131375	612,40
Rbm39	RNA binding motif protein 39	ENSMUSG00000027620	33000	611,67
Rpl4	ribosomal protein L4	ENSMUSG00000032399	5176	609,21
Gtf2i	general transcription factor II I	ENSMUSG00000060261	76927	605,00
Nin	ninein	ENSMUSG00000021068	100491	601,31
Csnk1a1	casein kinase 1, alpha 1	ENSMUSG00000024576	34788	600,73
Eya4	eyes absent 4 homolog (Drosophila)	ENSMUSG00000010461	245728	595,44
Atp5a1	ATP synthase, H+ transporting, mitochondrial F1 complex, alpha subunit 1	ENSMUSG00000025428	9141	592,54
Mrfap1	Morf4 family associated protein 1	ENSMUSG00000055302	1906	592,10
Syt11	synaptotagmin XI	ENSMUSG00000068923	30465	591,09
Mapt	microtubule-associated protein tau	ENSMUSG00000018411	100701	590,36
Baz2b	bromodomain adjacent to zinc finger domain, 2B	ENSMUSG00000026987	310477	589,85
Spag6	sperm associated antigen 6	ENSMUSG00000022783	76441	588,62
		ENSMUSG00000025515	7859	586,37
Uso1	USO1 vesicle docking factor	ENSMUSG00000029407	64857	586,08
Astn2	astrotactin 2	ENSMUSG00000028373	1023809	585,21
		ENSMUSG00000064367	1824	583,47
Krt8	keratin 8	ENSMUSG00000049382	7632	582,02
Ier2	immediate early response 2	ENSMUSG00000053560	1522	581,44
Ttc3	tetratricopeptide repeat domain 3	ENSMUSG00000040785	98605	581,37
		ENSMUSG00000079264	26770	580,72
Ptp4a2	protein tyrosine phosphatase 4a2	ENSMUSG00000028788	38785	574,99
Slc25a3	solute carrier family 25 (mitochondrial carrier, phosphate carrier), member 3	ENSMUSG00000061904	7486	574,12
Tmsb10	thymosin, beta 10	ENSMUSG00000079523	1402	570,14
Ptprm	protein tyrosine phosphatase, receptor type, M	ENSMUSG00000033278	687612	566,37
Rpl22	ribosomal protein L22	ENSMUSG00000028936	8330	563,25
Rbm25	RNA binding motif protein 25	ENSMUSG00000010608	51888	561,51

Atf4	activating transcription factor 4	ENSMUSG00000042406	2357	560,49
Arl6ip1	ADP-ribosylation factor-like 6 interacting protein 1	ENSMUSG00000030654	10735	560,28
Tsn	translin	ENSMUSG00000026374	12616	560,06
Nhs	Nance-Horan syndrome (human)	ENSMUSG00000059493	326435	558,76
Eif1	eukaryotic translation initiation factor 1	ENSMUSG00000035530	2212	557,74
Agap1	ArfGAP with GTPase domain, ankyrin repeat and PH domain 1	ENSMUSG00000055013	440472	556,73
Purb	purine rich element binding protein B	ENSMUSG00000094483	8478	556,51
Epas1	endothelial PAS domain protein 1	ENSMUSG00000024140	79504	554,48
Nos1ap	nitric oxide synthase 1 (neuronal) adaptor protein	ENSMUSG00000038473	287194	552,01
Ddx6	DEAD (Asp-Glu-Ala-Asp) box polypeptide 6	ENSMUSG00000032097	35840	551,29
Ivns1abp	influenza virus NS1A binding protein	ENSMUSG00000023150	19948	551,22
Aff3	AF4/FMR2 family, member 3	ENSMUSG00000037138	487630	550,20
Celf2	CUGBP, Elav-like family member 2	ENSMUSG00000002107	969870	546,36
App	amyloid beta (A4) precursor protein	ENSMUSG00000022892	219268	545,71
		ENSMUSG00000028234	4332	545,27
Sumf1	sulfatase modifying factor 1	ENSMUSG00000030101	78555	545,20
Gpx4	glutathione peroxidase 4	ENSMUSG00000075706	9274	544,40
Rpl8	ribosomal protein L8	ENSMUSG00000003970	2248	543,68
Fosb	FBJ osteosarcoma oncogene B	ENSMUSG00000003545	7325	543,68
Nd3	Mitochondrially encoded NADH dehydrogenase 3	ENSMUSG00000064360	348	542,95
Entpd5	ectonucleoside triphosphate diphosphohydrolase 5	ENSMUSG00000021236	35173	538,17
Pgrmc1	progesterone receptor membrane component 1	ENSMUSG00000006373	7874	536,43
Rbm47	RNA binding motif protein 47	ENSMUSG00000070780	156569	536,21
Cyp2f2	cytochrome P450, family 2, subfamily f, polypeptide 2	ENSMUSG00000052974	13752	534,62
Pbx1	pre B cell leukemia homeobox 1	ENSMUSG00000052534	278744	534,33
Csde1	cold shock domain containing E1, RNA binding	ENSMUSG00000068823	37644	532,22
Gpr133	G protein-coupled receptor 133	ENSMUSG00000044017	107850	527,00
Sfpq	splicing factor proline/glutamine rich (polypyrimidine tract binding protein associated)	ENSMUSG00000028820	15690	525,55
Cmip	c-Maf inducing protein	ENSMUSG00000034390	204485	525,26
		ENSMUSG00000049517	1812	523,09
Sptbn1	spectrin beta, non-erythrocytic 1	ENSMUSG00000020315	168781	521,79
Nfat5	nuclear factor of activated T cells 5	ENSMUSG00000003847	86048	520,12
Syt1	synaptotagmin I	ENSMUSG00000035864	513333	519,97
Rpsa	ribosomal protein SA	ENSMUSG00000032518	4604	519,90
Macf1	microtubule-actin crosslinking factor 1	ENSMUSG00000028649	334728	517,44
Rpl27a	ribosomal protein L27A	ENSMUSG00000046364	3221	514,03
Macrod2	MACRO domain containing 2	ENSMUSG00000068205	1997658	513,96
Clic6	chloride intracellular channel 6	ENSMUSG00000022949	55508	513,01
Rpl32	ribosomal protein L32	ENSMUSG00000057841	3243	511,35
Tecpr2	tectonin beta-propeller repeat containing 2	ENSMUSG00000021275	83131	509,97

Srsf5	serine/arginine-rich splicing factor 5	ENSMUSG00000021134	5004	509,46
Ezr	ezrin	ENSMUSG00000052397	44744	509,03
Sema5b	sema domain, seven thrombospondin repeats (type 1 and type 1-like), transmembrane domain (TM) and short cytoplasmic domain, (semaphorin) 5B	ENSMUSG00000052133	123588	506,27
Fmnl2	formin-like 2	ENSMUSG00000036053	276343	506,20
Arf1	ADP-ribosylation factor 1	ENSMUSG00000048076	16859	506,13
Ppp3ca	protein phosphatase 3, catalytic subunit, alpha isoform	ENSMUSG00000028161	267604	505,84
Ywhaz	tyrosine 3-monooxygenase/tryptophan 5-monooxygenase activation protein, zeta polypeptide	ENSMUSG00000022285	23778	503,01
Il33	interleukin 33	ENSMUSG00000024810	35605	502,94
Tcf12	transcription factor 12	ENSMUSG00000032228	267568	501,20
Myt1l	myelin transcription factor 1-like	ENSMUSG00000061911	394826	500,62
		ENSMUSG00000032036	550591	500,26
Galm	galactose mutarotase	ENSMUSG00000035473	57631	498,44
Pcdh17	protocadherin 17	ENSMUSG00000035566	93498	497,79
Gpm6a	glycoprotein m6a	ENSMUSG00000031517	106150	497,43
Lhx2	LIM homeobox protein 2	ENSMUSG00000000247	30453	497,14
Dapk1	death associated protein kinase 1	ENSMUSG00000021559	161239	496,92
		ENSMUSG00000051951	465598	496,34
Rpl21	ribosomal protein L21	ENSMUSG00000041453	4143	495,91
Vapa	vesicle-associated membrane protein, associated protein A	ENSMUSG00000024091	33500	495,40
		ENSMUSG00000019132	12357	495,25
Ppa1	pyrophosphatase (inorganic) 1	ENSMUSG00000020089	25617	492,64
Strbp	spermatid perinuclear RNA binding protein	ENSMUSG00000026915	133992	488,22
Tex15	testis expressed gene 15	ENSMUSG00000009628	68845	486,34
		ENSMUSG00000026678	40313	485,54
Hsd17b11	hydroxysteroid (17-beta) dehydrogenase 11	ENSMUSG00000029311	32158	484,67
Lcor	ligand dependent nuclear receptor corepressor	ENSMUSG00000025019	79602	482,79
		ENSMUSG00000063696	1075	482,57
Spint2	serine protease inhibitor, Kunitz type 2	ENSMUSG00000074227	25647	482,35
Gsk3b	glycogen synthase kinase 3 beta	ENSMUSG00000022812	157084	481,92
Rtn4	reticulon 4	ENSMUSG00000020458	51385	477,78
Lpgat1	lysophosphatidylglycerol acyltransferase 1	ENSMUSG00000026623	66422	475,90
Txnrd1	thioredoxin reductase 1	ENSMUSG00000020250	38519	475,68
Frmpd4	FERM and PDZ domain containing 4	ENSMUSG00000049176	1105923	473,72
Hsp90aa1	heat shock protein 90, alpha (cytosolic), class A member 1	ENSMUSG00000021270	12124	473,43
Macrod1	MACRO domain containing 1	ENSMUSG00000036278	141294	473,22
Cnbd2	cyclic nucleotide binding domain containing 2	ENSMUSG00000038085	63340	469,52
Jarid2	jumonji, AT rich interactive domain 2	ENSMUSG00000038518	192170	468,65
Ankrd12	ankyrin repeat domain 12	ENSMUSG00000034647	109589	467,42

Ccser1	coiled-coil serine rich 1	ENSMUSG00000039578	1202542	463,43
		ENSMUSG00000048915	277134	462,13
Akap9	A kinase (PRKA) anchor protein (yotiao) 9	ENSMUSG00000040407	152156	461,84
Tanc2	tetratricopeptide repeat, ankyrin repeat and coiled-coil containing 2	ENSMUSG00000053580	339319	461,47
Lca5	Leber congenital amaurosis 5 (human)	ENSMUSG00000032258	50835	461,18
Rps26-ps1	ribosomal protein S26, pseudogene 1	ENSMUSG00000059775	410	455,53
Luc7l2	LUC7-like 2 (S. cerevisiae)	ENSMUSG00000029823	58137	453,72
Ctcf	CCCTC-binding factor	ENSMUSG00000005698	46355	451,90
Cadps	Ca2+-dependent secretion activator	ENSMUSG00000054423	450517	451,83
C230081A13Rik	RIKEN cDNA C230081A13 gene	ENSMUSG00000074305	216920	451,83
Tle3	transducin-like enhancer of split 3, homolog of Drosophila E(spl)	ENSMUSG00000032280	46132	451,47
Phkg1	phosphorylase kinase gamma 1	ENSMUSG00000025537	35129	450,96
Wnk1	WNK lysine deficient protein kinase 1	ENSMUSG00000045962	114704	450,67
Parva	parvin, alpha	ENSMUSG00000030770	164188	449,37
		ENSMUSG00000018849	142128	446,98
Pdia6	protein disulfide isomerase associated 6	ENSMUSG00000020571	18226	446,32
Tenm3	teneurin transmembrane protein 3	ENSMUSG00000031561	447009	445,09
Hnrnpu	heterogeneous nuclear ribonucleoprotein U	ENSMUSG00000039630	14724	444,29
Gpm6b	glycoprotein m6b	ENSMUSG00000031342	150078	443,35
Qdpr	quinoid dihydropteridine reductase	ENSMUSG00000015806	16204	441,39
Hdgf	hepatoma-derived growth factor	ENSMUSG00000004897	9812	441,25
Pard3b	par-3 partitioning defective 3 homolog B (C. elegans)	ENSMUSG00000052062	1003461	440,96
Lpp	LIM domain containing preferred translocation partner in lipoma	ENSMUSG00000033306	599227	440,74
Chchd2	coiled-coil-helix-coiled-coil-helix domain containing 2	ENSMUSG00000070493	6315	440,16
Rps27a	ribosomal protein S27A	ENSMUSG00000020460	2264	440,09
Rtn3	reticulon 3	ENSMUSG00000024758	57389	438,28
Eya1	eyes absent 1 homolog (Drosophila)	ENSMUSG00000025932	141247	438,28
Rangap1	RAN GTPase activating protein 1	ENSMUSG00000022391	25672	436,97
Phactr1	phosphatase and actin regulator 1	ENSMUSG00000054728	457904	432,26
Btbd9	BTB (POZ) domain containing 9	ENSMUSG00000062202	360764	432,04
C530008M17Rik	RIKEN cDNA C530008M17 gene	ENSMUSG00000036377	215872	431,75
Srgap1	SLIT-ROBO Rho GTPase activating protein 1	ENSMUSG00000020121	266325	431,32
Nudcd3	NudC domain containing 3	ENSMUSG00000053838	94725	431,17
Ncl	nucleolin	ENSMUSG00000026234	14737	430,59
Frmd4b	FERM domain containing 4B	ENSMUSG00000030064	330675	430,37
Dpysl2	dihydropyrimidinase-like 2	ENSMUSG00000022048	65825	430,30
		ENSMUSG00000022858	21457	430,30
		ENSMUSG00000028521	44516	429,29
Tmcc1	transmembrane and coiled coil domains 1	ENSMUSG00000030126	174869	427,48
Atp1b1	ATPase, Na+/K+ transporting, beta 1 polypeptide	ENSMUSG00000026576	21089	426,97

Hnrnpa3	heterogeneous nuclear ribonucleoprotein A3	ENSMUSG00000059005	10147	426,61
Rab6a	RAB6A, member RAS oncogene family	ENSMUSG00000030704	33859	425,01
Clta	clathrin, light polypeptide (Lca)	ENSMUSG00000028478	28395	424,43
Pdzd8	PDZ domain containing 8	ENSMUSG00000074746	49697	424,07
Prrc2c	proline-rich coiled-coil 2C	ENSMUSG00000040225	69832	424,00
Anxa5	annexin A5	ENSMUSG00000027712	26919	423,63
		ENSMUSG00000029442	75383	420,88
Car1	carbonic anhydrase 1	ENSMUSG00000027556	42153	419,14
Plekha5	pleckstrin homology domain containing, family A member 5	ENSMUSG00000030231	170808	418,34
		ENSMUSG00000007440	257373	417,69
Scn9a	sodium channel, voltage-gated, type IX, alpha	ENSMUSG00000075316	154873	417,18
Farp1	FERM, RhoGEF (Arhgef) and pleckstrin domain protein 1 (chondrocyte-derived)	ENSMUSG00000025555	248545	415,51
Homer2	homer homolog 2 (Drosophila)	ENSMUSG00000025813	106445	413,85
Rpl19	ribosomal protein L19	ENSMUSG00000017404	3783	413,56
Top1	topoisomerase (DNA) I	ENSMUSG00000070544	76877	412,69
Slc12a2	solute carrier family 12, member 2	ENSMUSG00000024597	68144	412,47
Frmd4a	FERM domain containing 4A	ENSMUSG00000026657	596327	411,53
Zfp608	zinc finger protein 608	ENSMUSG00000052713	102136	410,59
Rpl9-ps6	ribosomal protein L9, pseudogene 6	ENSMUSG00000062456	691	409,64
Kcnma1	potassium large conductance calcium-activated channel, subfamily M, alpha member 1	ENSMUSG00000063142	513874	409,35
Eef1b2	eukaryotic translation elongation factor 1 beta 2	ENSMUSG00000025967	3662	409,21
Map2	microtubule-associated protein 2	ENSMUSG00000015222	267311	407,98
Tst	thiosulfate sulfurtransferase, mitochondrial	ENSMUSG00000044986	6304	407,54
Taf1c	TATA box binding protein (Tbp)-associated factor, RNA polymerase I, C	ENSMUSG00000031832	7248	407,32
		ENSMUSG00000029212	438458	406,38
Ywhaq	tyrosine 3-monooxygenase/tryptophan 5-monooxygenase activation protein, theta polypeptide	ENSMUSG00000076432	27567	406,02
Atp11a	ATPase, class VI, type 11A	ENSMUSG00000031441	111715	405,80
		ENSMUSG00000056222	478293	404,71
Camk2d	calcium/calmodulin-dependent protein kinase II, delta	ENSMUSG00000053819	248612	404,13
Atp5o	ATP synthase, H+ transporting, mitochondrial F1 complex, O subunit	ENSMUSG00000022956	247290	403,12
Ptprd	protein tyrosine phosphatase, receptor type, D	ENSMUSG00000028399	2270724	400,51
Tbc1d8b	TBC1 domain family, member 8B	ENSMUSG00000042473	68410	399,71
Ubr5	ubiquitin protein ligase E3 component n-recognin 5	ENSMUSG00000037487	111526	397,90

Tmtc2	transmembrane and tetratricopeptide repeat containing 2	ENSMUSG00000036019	386789	397,03
Fbxl13	F-box and leucine-rich repeat protein 13	ENSMUSG00000048520	161759	395,29
Wwox	WW domain-containing oxidoreductase	ENSMUSG00000004637	913054	394,42
Nol4	nucleolar protein 4	ENSMUSG00000041923	348473	394,20
Epb4.1	erythrocyte protein band 4.1	ENSMUSG00000028906	151909	393,98
Ddah1	dimethylarginine dimethylaminohydrolase 1	ENSMUSG00000028194	135603	393,26
Atxn7l3b	ataxin 7-like 3B	ENSMUSG00000074748	3589	392,75
		ENSMUSG00000009470	87366	392,61
Arfip1	ADP-ribosylation factor interacting protein 1	ENSMUSG00000074513	1391424	391,01
Slc6a6	solute carrier family 6 (neurotransmitter transporter, taurine), member 6	ENSMUSG00000030096	74997	389,71
Map7	microtubule-associated protein 7	ENSMUSG00000019996	133119	389,49
Apc	adenomatosis polyposis coli	ENSMUSG00000005871	101266	389,20
Srsf3	serine/arginine-rich splicing factor 3	ENSMUSG00000071172	10694	388,33
Mgll	monoglyceride lipase	ENSMUSG00000033174	103949	387,97
Gm10076	predicted gene 10076	ENSMUSG00000060143	384	387,90
Magi3	membrane associated guanylate kinase, WW and PDZ domain containing 3	ENSMUSG00000052539	207116	387,10
Rab44	RAB44, member RAS oncogene family	ENSMUSG00000064147	13925	386,95
Iqgap1	IQ motif containing GTPase activating protein 1	ENSMUSG00000030536	91749	386,59
Gusb	glucuronidase, beta	ENSMUSG00000025534	14039	386,37
H3f3a	H3 histone, family 3A	ENSMUSG00000060743	13112	386,30
Gm10123	predicted pseudogene 10123	ENSMUSG00000062933	736	386,16
Mllt3	myeloid/lymphoid or mixed-lineage leukemia (trithorax homolog, Drosophila); translocated to, 3	ENSMUSG00000028496	263440	385,58
Hnrnpa2b1	heterogeneous nuclear ribonucleoprotein A2/B1	ENSMUSG00000004980	9461	385,21
Rasal2	RAS protein activator like 2	ENSMUSG00000070565	277413	384,78
Mbp	myelin basic protein	ENSMUSG00000041607	110492	383,69
		ENSMUSG00000046079	132468	383,47
Cpe	carboxypeptidase E	ENSMUSG00000037852	100483	383,33
Etl4	enhancer trap locus 4	ENSMUSG00000036617	900934	381,37
Trps1	trichorhinophalangeal syndrome I (human)	ENSMUSG00000038679	235281	381,23
Ube2k	ubiquitin-conjugating enzyme E2K	ENSMUSG00000029203	61756	380,57
Kcnip4	Kv channel interacting protein 4	ENSMUSG00000029088	1135403	380,36
Gm10161	predicted pseudogene 10161	ENSMUSG00000066407	300	380,14
Cox7a2l	cytochrome c oxidase subunit VIIa polypeptide 2-like	ENSMUSG00000024248	12415	379,78
Ephb1	Eph receptor B1	ENSMUSG00000032537	432566	379,70
Mrps28	mitochondrial ribosomal protein S28	ENSMUSG00000040269	121773	379,27
Dnaja1	DnaJ (Hsp40) homolog, subfamily A, member 1	ENSMUSG00000028410	15000	379,05
Brd2	bromodomain containing 2	ENSMUSG00000024335	10612	378,83

		ENSMUSG00000073640	475	378,76
Med13	mediator complex subunit 13	ENSMUSG00000034297	90570	378,76
Kctd16	potassium channel tetramerisation domain containing 16	ENSMUSG00000051401	272808	378,25
Stox2	storkhead box 2	ENSMUSG00000038143	172301	377,53
Robo1	roundabout homolog 1 (Drosophila)	ENSMUSG00000022883	382947	376,66
		ENSMUSG00000071866	4375	376,37
Ctnna1	catenin (cadherin associated protein), alpha 1	ENSMUSG00000037815	135886	375,57
Cpne4	copine IV	ENSMUSG00000032564	487259	375,50
Lars2	leucyl-tRNA synthetase, mitochondrial	ENSMUSG00000035202	95725	375,21
Heph	hephaestin	ENSMUSG00000031209	119127	374,27
Runx1	runt related transcription factor 1	ENSMUSG00000022952	224600	373,54
Tshz1	teashirt zinc finger family member 1	ENSMUSG00000046982	74778	373,33
Rev3l	REV3-like, catalytic subunit of DNA polymerase zeta RAD54 like (S. cerevisiae)	ENSMUSG00000019841	143094	370,57
		ENSMUSG00000055421	873221	370,43
Taldo1	transaldolase 1	ENSMUSG00000025503	10770	369,70
Zmynd8	zinc finger, MYND-type containing 8	ENSMUSG00000039671	114865	369,70
Gap43	growth associated protein 43	ENSMUSG00000047261	92210	369,34
Dnmt3a	DNA methyltransferase 3A	ENSMUSG00000020661	108437	369,34
Lingo2	leucine rich repeat and Ig domain containing 2	ENSMUSG00000045083	1245101	368,90
Basp1	brain abundant, membrane attached signal protein 1	ENSMUSG00000045763	50480	368,03
Exoc6b	exocyst complex component 6B	ENSMUSG00000033769	451027	367,96
Cdh26	cadherin-like 26	ENSMUSG00000039155	56836	367,45
Selt	selenoprotein T	ENSMUSG00000075700	16498	366,80
		ENSMUSG00000025487	16676	365,86
Arglu1	arginine and glutamate rich 1	ENSMUSG00000040459	23961	364,41
Chmp1a	charged multivesicular body protein 1A	ENSMUSG00000000743	8500	363,18
Ubn2	ubinuclein 2	ENSMUSG00000038538	90876	363,03
Rbfox2	RNA binding protein, fox-1 homolog (C. elegans) 2	ENSMUSG00000033565	228064	362,89
Jund	Jun proto-oncogene related gene d	ENSMUSG00000071076	2878	362,23
		ENSMUSG00000032238	734461	361,22
Prrc2b	proline-rich coiled-coil 2B	ENSMUSG00000039262	83456	361,07
Srpk2	serine/arginine-rich protein specific kinase 2	ENSMUSG00000062604	113216	361,07
Ank2	ankyrin 2, brain	ENSMUSG00000032826	577654	360,35
Tkt	transketolase	ENSMUSG00000021957	26364	360,13
Gm9843	predicted gene 9843	ENSMUSG00000050299	504	360,06
Nisch	nischarin	ENSMUSG00000021910	46017	359,91
Reg3g	regenerating islet-derived 3 gamma	ENSMUSG00000030017	2604	359,55
Tspan13	tetraspanin 13	ENSMUSG00000020577	27944	358,25
Nipbl	Nipped-B homolog (Drosophila)	ENSMUSG00000022141	153239	358,03
C330021F23Rik	RIKEN cDNA C330021F23 gene	ENSMUSG00000065952	16950	357,59
Rpl14	ribosomal protein L14	ENSMUSG00000025794	3137	357,52
Sel1l	sel-1 suppressor of lin-12-like (C. elegans)	ENSMUSG00000020964	43115	356,29

Eif4g1	eukaryotic translation initiation factor 4, gamma 1	ENSMUSG00000045983	24572	355,71
Aplp2	amyloid beta (A4) precursor-like protein 2	ENSMUSG00000031996	62259	355,06
Sf3b1	splicing factor 3b, subunit 1	ENSMUSG00000025982	42310	354,41
Agbl4	ATP/GTP binding protein-like 4	ENSMUSG00000061298	1266664	353,17
		ENSMUSG00000059835	624	353,03
Akap6	A kinase (PRKA) anchor protein 6	ENSMUSG00000061603	451633	352,45
Gpatch8	G patch domain containing 8	ENSMUSG00000034621	80478	352,01
		ENSMUSG00000047037	42388	350,56
Ankhd1	ankyrin repeat and KH domain containing 1	ENSMUSG00000024483	105931	350,27
Ugdh	UDP-glucose dehydrogenase	ENSMUSG00000029201	22745	350,20
		ENSMUSG00000003992	243147	349,98
Tulp4	tubby like protein 4	ENSMUSG00000034377	144692	349,84
Vcp	valosin containing protein	ENSMUSG00000028452	20545	349,11
Cox7b	cytochrome c oxidase subunit VIIb	ENSMUSG00000031231	6751	348,32
Rtn1	reticulon 1	ENSMUSG00000021087	197303	348,17
Crebbp	CREB binding protein	ENSMUSG00000022521	129357	348,03
Ide	insulin degrading enzyme	ENSMUSG00000056999	69110	347,66
Hmgcs1	3-hydroxy-3-methylglutaryl-Coenzyme A synthase 1	ENSMUSG00000093930	17625	347,23
Mgst1	microsomal glutathione S-transferase 1	ENSMUSG00000008540	16440	346,87
Mapk10	mitogen-activated protein kinase 10	ENSMUSG00000046709	303387	345,85
Zfp36l1	zinc finger protein 36, C3H type-like 1	ENSMUSG00000021127	5254	345,56
Mycbp2	MYC binding protein 2	ENSMUSG00000033004	233404	345,49
Chd7	chromodomain helicase DNA binding protein 7	ENSMUSG00000041235	177254	344,62
Slc22a23	solute carrier family 22, member 23	ENSMUSG00000038267	166025	344,11
Map4	microtubule-associated protein 4	ENSMUSG00000032479	152490	343,53
Rcor1	REST corepressor 1	ENSMUSG00000037896	76551	342,73
		ENSMUSG00000027309	200207	342,23
Psme4	proteasome (prosome, macropain) activator subunit 4	ENSMUSG00000040850	108636	341,79
Syne2	spectrin repeat containing, nuclear envelope 2	ENSMUSG00000063450	292793	341,57
Tmem132d	transmembrane protein 132D	ENSMUSG00000034310	649587	341,07
		ENSMUSG00000005533	273922	340,99
Ywhab	tyrosine 3-monooxygenase/tryptophan 5-monooxygenase activation protein, beta polypeptide	ENSMUSG00000018326	23629	340,70
Grlf1	glucocorticoid receptor DNA binding factor 1	ENSMUSG00000058230	120521	340,63
Arhgap5	Rho GTPase activating protein 5	ENSMUSG00000035133	51776	339,47
Pon1	paraoxonase 1	ENSMUSG00000002588	25857	338,53
Magi1	membrane associated guanylate kinase, WW and PDZ domain containing 1	ENSMUSG00000045095	607868	338,09
Lrba	LPS-responsive beige-like anchor	ENSMUSG00000028080	558004	337,59
Kmt2c	lysine (K)-specific methyltransferase 2C	ENSMUSG00000038056	226986	337,30
Gnal	guanine nucleotide binding protein, alpha stimulating, olfactory type	ENSMUSG00000024524	138457	337,22

Ccdc88a	coiled coil domain containing 88A	ENSMUSG00000032740	137151	337,01
Bcl7a	B cell CLL/lymphoma 7A	ENSMUSG00000029438	30249	336,72
Pcf11	cleavage and polyadenylation factor subunit homolog (S. cerevisiae)	ENSMUSG00000041328	26222	336,28
Sdha	succinate dehydrogenase complex, subunit A, flavoprotein (Fp)	ENSMUSG00000021577	28027	336,21
Itfg1	integrin alpha FG-GAP repeat containing 1	ENSMUSG00000031703	123393	336,07
Luc7l	Luc7 homolog (S. cerevisiae)-like	ENSMUSG00000024188	32595	335,41
Ctnnb1	catenin (cadherin associated protein), beta 1	ENSMUSG00000006932	31292	334,98
Rfx3	regulatory factor X, 3 (influences HLA class II expression)	ENSMUSG00000040929	249446	334,98
Cacna2d1	calcium channel, voltage-dependent, alpha2/delta subunit 1	ENSMUSG00000040118	439821	334,69
Nav1	neuron navigator 1	ENSMUSG00000009418	150776	334,54
Scaf11	SR-related CTD-associated factor 11	ENSMUSG00000033228	49146	333,75
Ror1		ENSMUSG00000035305	348975	333,67
Tnrc6b	trinucleotide repeat containing 6b	ENSMUSG00000047888	229768	333,31
Rbms1	RNA binding motif, single stranded interacting protein 1	ENSMUSG00000026970	213000	332,51
Pnpla7	patatin-like phospholipase domain containing 7	ENSMUSG00000036833	78025	331,93
Pcbp1	poly(rC) binding protein 1	ENSMUSG00000051695	1673	331,79
Tuba1c	tubulin, alpha 1C	ENSMUSG00000043091	8215	331,57
Snx14	sorting nexin 14	ENSMUSG00000032422	62212	331,43
Aldh2	aldehyde dehydrogenase 2, miochondrial	ENSMUSG00000029455	27798	330,85
Zmym1	zinc finger, MYM domain containing 1	ENSMUSG00000043872	14059	330,19
Dync1h1	dynein cytoplasmic 1 heavy chain 1	ENSMUSG00000018707	65494	329,76
Gcnt2	glucosaminyl (N-acetyl) transferase 2, I-branching enzyme	ENSMUSG00000021360	101124	329,54
		ENSMUSG00000076431	4795	328,89
1700022I11Rik	RIKEN cDNA 1700022I11 gene	ENSMUSG00000028451	13694	328,53
Psap	prosaposin	ENSMUSG00000004207	24971	328,24
Rock1	Rho-associated coiled-coil containing protein kinase 1	ENSMUSG00000024290	117392	327,58
Pafah1b1	platelet-activating factor acetylhydrolase, isoform 1b, subunit 1	ENSMUSG00000020745	50722	327,37
Ptges3	prostaglandin E synthase 3 (cytosolic)	ENSMUSG00000071072	18319	325,55
Nktr	natural killer tumor recognition sequence	ENSMUSG00000032525	37675	325,48
Abcc4	ATP-binding cassette, sub-family C (CFTR/MRP), member 4	ENSMUSG00000032849	223528	325,26
Hes1	hairy and enhancer of split 1 (Drosophila)	ENSMUSG00000022528	3413	325,05
Bcl11b	B cell leukemia/lymphoma 11B	ENSMUSG00000048251	93200	324,61

Smarca4	SWI/SNF related, matrix associated, actin dependent regulator of chromatin, subfamily a, member 4	ENSMUSG00000032187	88062	324,03
		ENSMUSG00000034040	435997	323,96
Uqcrh	ubiquinol-cytochrome c reductase hinge protein	ENSMUSG00000063882	8107	323,52
Ermn	ermin, ERM-like protein	ENSMUSG00000026830	7752	323,52
Aff4	AF4/FMR2 family, member 4	ENSMUSG00000049470	70998	323,02
Ash1l	ash1 (absent, small, or homeotic)-like (Drosophila)	ENSMUSG00000028053	113562	322,87
Bod1l	biorientation of chromosomes in cell division 1-like	ENSMUSG00000061755	56627	322,80
Rps19	ribosomal protein S19	ENSMUSG00000040952	5436	322,73
Klf7	Kruppel-like factor 7 (ubiquitous)	ENSMUSG00000025959	92836	322,73
Morf4l1	mortality factor 4 like 1	ENSMUSG00000062270	23150	322,51
Cds1	CDP-diacylglycerol synthase 1	ENSMUSG00000029330	58729	322,29
Stat3	signal transducer and activator of transcription 3	ENSMUSG00000004040	54443	322,00
Fam120a	family with sequence similarity 120, member A	ENSMUSG00000038014	88799	321,78
Sh3bgrl	SH3-binding domain glutamic acid-rich protein like	ENSMUSG00000031246	102509	321,42
Eif4a1	eukaryotic translation initiation factor 4A1	ENSMUSG00000059796	5488	320,91
Slc24a2	solute carrier family 24 (sodium/potassium/calcium exchanger), member 2	ENSMUSG00000037996	247354	320,91
Cdh2	cadherin 2	ENSMUSG00000024304	220370	320,62
Pitpnc1	phosphatidylinositol transfer protein, cytoplasmic 1	ENSMUSG00000040430	262808	320,55
Eif3c	eukaryotic translation initiation factor 3, subunit C	ENSMUSG00000030738	19957	320,33
Zfp462	zinc finger protein 462	ENSMUSG00000060206	138516	320,33
Gpc1	glypican 1	ENSMUSG00000034220	28554	319,83
Fam134a	family with sequence similarity 134, member A	ENSMUSG00000049339	5124	319,68
Sobp	sine oculis-binding protein homolog (Drosophila)	ENSMUSG00000038248	172030	319,10
Epb4.1l2	erythrocyte protein band 4.1-like 2	ENSMUSG00000019978	163722	318,67
Usp34	ubiquitin specific peptidase 34	ENSMUSG00000056342	183666	317,94
Fth1	ferritin heavy chain 1	ENSMUSG00000024661	2390	317,15
Atxn10	ataxin 10	ENSMUSG00000016541	127456	316,86
Igfbp5	insulin-like growth factor binding protein 5	ENSMUSG00000026185	16953	316,28
Ywhae	tyrosine 3-monooxygenase/tryptophan 5-monooxygenase activation protein, epsilon polypeptide	ENSMUSG00000020849	32977	315,91
Rpl36al	ribosomal protein L36A-like	ENSMUSG00000049751	1353	315,62
Lcp1	lymphocyte cytosolic protein 1	ENSMUSG00000021998	99742	315,55
Sec62	SEC62 homolog (S. cerevisiae)	ENSMUSG00000027706	28388	315,48
Pawr	PRKC, apoptosis, WT1, regulator	ENSMUSG00000035873	82203	315,33
Zfand3	zinc finger, AN1-type domain 3	ENSMUSG00000044477	204933	314,83
Bcl6	B cell leukemia/lymphoma 6	ENSMUSG00000022508	23801	314,61
Wdr17	WD repeat domain 17	ENSMUSG00000039375	258130	313,45

Ces1d	carboxylesterase 1D	ENSMUSG00000056973	31771	312,36
Hnrnpa0	heterogeneous nuclear ribonucleoprotein A0	ENSMUSG00000007836	2678	312,22
Lmo4	LIM domain only 4	ENSMUSG00000028266	16691	312,07
Psmb1	proteasome (prosome, macropain) subunit, beta type 1	ENSMUSG00000014769	22556	310,98
Pde1c	phosphodiesterase 1C	ENSMUSG00000004347	299814	310,98
Plxnb1	plexin B1	ENSMUSG00000053646	24476	310,69
Foxn3	forkhead box N3	ENSMUSG00000033713	255132	310,69
Ube2h	ubiquitin-conjugating enzyme E2H	ENSMUSG00000039159	93251	310,33
Tcf7l2	transcription factor 7 like 2, T cell specific, HMG box	ENSMUSG00000024985	191845	309,97
Ahcyl1	S-adenosylhomocysteine hydrolase-like 1	ENSMUSG00000027893	33443	309,75
Ggt7	gamma-glutamyltransferase 7	ENSMUSG00000027603	27859	309,75
Rpl34	ribosomal protein L34	ENSMUSG00000062006	3568	309,24
Atp2a2	ATPase, Ca++ transporting, cardiac muscle, slow twitch 2	ENSMUSG00000029467	59524	309,17
Wbp5	WW domain binding protein 5	ENSMUSG00000042712	2061	308,88
Myo9a	myosin IXa	ENSMUSG00000039585	177971	308,66
Lamp1	lysosomal-associated membrane protein 1	ENSMUSG00000031447	16204	308,23
Atp13a5	ATPase type 13A5	ENSMUSG00000048939	146882	308,08
		ENSMUSG00000072294	275279	307,87
Ptprs	protein tyrosine phosphatase, receptor type, S	ENSMUSG00000013236	64053	307,36
Akap13	A kinase (PRKA) anchor protein 13	ENSMUSG00000066406	299076	307,14
Hp1bp3	heterochromatin protein 1, binding protein 3	ENSMUSG00000028759	28388	305,98
Alox15	arachidonate 15-lipoxygenase	ENSMUSG00000018924	7880	305,84
Sftpd	surfactant associated protein D	ENSMUSG00000021795	12987	305,84
Tnrc6c	trinucleotide repeat containing 6C	ENSMUSG00000025571	109151	305,69
Npm1	nucleophosmin 1	ENSMUSG00000057113	10920	305,47
Cd9	CD9 antigen	ENSMUSG00000030342	34526	305,26
Kcnd2	potassium voltage-gated channel, Shal-related family, member 2	ENSMUSG00000060882	514303	305,11
Brd1	bromodomain containing 1	ENSMUSG00000022387	46934	304,68
Kcnk2	potassium channel, subfamily K, member 2	ENSMUSG00000037624	135902	304,60
Hmg20a	high mobility group 20A	ENSMUSG00000032329	78241	304,53
Eif4g2	eukaryotic translation initiation factor 4, gamma 2	ENSMUSG00000005610	15046	304,17
Arid1b	AT rich interactive domain 1B (SWI-like)	ENSMUSG00000069729	353325	303,73
Pbrm1	polybromo 1	ENSMUSG00000042323	102455	303,59
Med13l	mediator complex subunit 13-like	ENSMUSG00000018076	204717	303,08
Rpl13	ribosomal protein L13	ENSMUSG00000000740	2895	302,86
Rbm3	RNA binding motif protein 3	ENSMUSG00000031167	6906	302,79
Phip	pleckstrin homology domain interacting protein	ENSMUSG00000032253	109331	302,50
Calm3	calmodulin 3	ENSMUSG00000019370	8736	301,70
Hdlbp	high density lipoprotein (HDL) binding protein	ENSMUSG00000034088	72869	301,63
Tbcel	tubulin folding cofactor E-like	ENSMUSG00000037287	95494	301,41
Kmt2a	lysine (K)-specific methyltransferase 2A	ENSMUSG00000002028	77942	301,20

Bptf	bromodomain PHD finger transcription factor	ENSMUSG00000040481	99047	300,91
		ENSMUSG00000023036	167539	300,33
		ENSMUSG00000022748	304627	300,18
Tnik	TRAF2 and NCK interacting kinase	ENSMUSG00000027692	412645	299,82
Cox6a1	cytochrome c oxidase subunit VIa polypeptide 1	ENSMUSG00000041697	3340	299,75
Ppap2b	phosphatidic acid phosphatase type 2B	ENSMUSG00000028517	75418	299,75
Laptm4a	lysosomal-associated protein transmembrane 4A	ENSMUSG00000020585	17079	299,75
Ccnl1	cyclin L1	ENSMUSG00000027829	12099	299,38
Rps12-ps3	ribosomal protein S12, pseudogene 3	ENSMUSG00000067038	494	299,17
Pbx3	pre B cell leukemia homeobox 3	ENSMUSG00000038718	201684	299,17
Dtna	dystrobrevin alpha	ENSMUSG00000024302	244300	298,88
Kmt2e	lysine (K)-specific methyltransferase 2E	ENSMUSG00000029004	69795	298,66
Mapre1	microtubule-associated protein, RP/EB family, member 1	ENSMUSG00000027479	32037	297,21
Elavl4	ELAV (embryonic lethal, abnormal vision, Drosophila)-like 4 (Hu antigen D)	ENSMUSG00000028546	148188	296,85
		ENSMUSG00000006649	28909	296,78
Enox1	ecto-NOX disulfide-thiol exchanger 1	ENSMUSG00000022012	564998	296,27
Cnot6	CCR4-NOT transcription complex, subunit 6	ENSMUSG00000020362	41221	295,83
Wdr78	WD repeat domain 78	ENSMUSG00000035126	76491	295,62
Ppp1r10	protein phosphatase 1, regulatory subunit 10	ENSMUSG00000039220	15848	295,47
Pdss2	prenyl (solanesyl) diphosphate synthase, subunit 2	ENSMUSG00000038240	243397	294,02
Nucks1	nuclear casein kinase and cyclin-dependent kinase substrate 1	ENSMUSG00000026434	25864	293,95
Eif3a	eukaryotic translation initiation factor 3, subunit A	ENSMUSG00000024991	29578	293,08
Arid4b	AT rich interactive domain 4B (RBP1-like)	ENSMUSG00000039219	136372	293,01
9930021J03Rik	RIKEN cDNA 9930021J03 gene	ENSMUSG00000046138	91608	292,72
Sox2	SRY-box containing gene 2	ENSMUSG00000074637	2457	292,50
Bclaf1	BCL2-associated transcription factor 1	ENSMUSG00000037608	30033	292,06
Abcb7	ATP-binding cassette, sub-family B (MDR/TAP), member 7	ENSMUSG00000031333	133200	291,63
Kdm5b	lysine (K)-specific demethylase 5B	ENSMUSG00000042207	72707	290,98
Dynll2	dynein light chain LC8-type 2	ENSMUSG00000020483	8009	290,47
Nsd1	nuclear receptor-binding SET-domain protein 1	ENSMUSG00000021488	108544	290,25
Kcnq1	potassium voltage-gated channel, subfamily Q, member 1	ENSMUSG00000009545	319789	290,18
Fhit	fragile histidine triad gene	ENSMUSG00000060579	1611944	289,89
Maml3	mastermind like 3 (Drosophila)	ENSMUSG00000061143	417281	289,82
Tomm7	translocase of outer mitochondrial membrane 7 homolog (yeast)	ENSMUSG00000028998	5218	289,60

Strn3	striatin, calmodulin binding protein 3	ENSMUSG00000020954	83374	289,45
Slc4a7	solute carrier family 4, sodium bicarbonate cotransporter, member 7	ENSMUSG00000021733	96919	289,31
Rps3a2	ribosomal protein S3A2	ENSMUSG00000062611	863	289,24
Prkar1a	protein kinase, cAMP dependent regulatory, type I, alpha	ENSMUSG00000020612	20252	288,37
Endod1	endonuclease domain containing 1	ENSMUSG00000037419	27253	287,86
Ralbp1	ralA binding protein 1	ENSMUSG00000024096	37323	287,21
Smg6	Smg-6 homolog, nonsense mediated mRNA decay factor (C. elegans)	ENSMUSG00000038290	238626	286,63
Camta1	calmodulin binding transcription activator 1	ENSMUSG00000014592	944555	285,83
Chd6	chromodomain helicase DNA binding protein 6	ENSMUSG00000057133	162098	285,76
Filip1l	filamin A interacting protein 1-like	ENSMUSG00000043336	220034	285,18
Pip4k2a	phosphatidylinositol-5-phosphate 4-kinase, type II, alpha	ENSMUSG00000026737	155872	285,10
Mt1	metallothionein 1	ENSMUSG00000031765	1237	284,89
Ppig	peptidyl-prolyl isomerase G (cyclophilin G)	ENSMUSG00000042133	31468	284,67
Immp2l	IMP2 inner mitochondrial membrane peptidase-like (S. cerevisiae)	ENSMUSG00000056899	931499	284,45
Pum1	pumilio 1 (Drosophila)	ENSMUSG00000028580	118244	284,31
Arhgap21	Rho GTPase activating protein 21	ENSMUSG00000036591	120963	284,02
Ncapg2	non-SMC condensin II complex, subunit G2	ENSMUSG00000042029	58130	283,73
		ENSMUSG00000003452	154346	283,51
Gon4l	gon-4-like (C.elegans)	ENSMUSG00000054199	74873	283,00
Rab2a	RAB2A, member RAS oncogene family	ENSMUSG00000047187	72135	282,93
Rufy3	RUN and FYVE domain containing 3	ENSMUSG00000029291	59098	282,06
Ran	RAN, member RAS oncogene family	ENSMUSG00000029430	4255	281,77
Ddx3x	DEAD/H (Asp-Glu-Ala-Asp/His) box polypeptide 3, X-linked	ENSMUSG00000000787	13083	281,70
Bach2	BTB and CNC homology 2	ENSMUSG00000040270	347305	281,26
Tra2a	transformer 2 alpha homolog (Drosophila)	ENSMUSG00000029817	20132	281,04
Dclk1	doublecortin-like kinase 1	ENSMUSG00000027797	296539	280,90
Celf1	CUGBP, Elav-like family member 1	ENSMUSG00000005506	79116	279,52
Wdpcp	WD repeat containing planar cell polarity effector	ENSMUSG00000020319	326755	278,29
		ENSMUSG00000049313	159578	278,29
Pla2r1	phospholipase A2 receptor 1	ENSMUSG00000054580	135766	278,00
Dis3l2	DIS3 mitotic control homolog (S. cerevisiae)-like 2	ENSMUSG00000053333	346294	277,93
Eef1g	eukaryotic translation elongation factor 1 gamma	ENSMUSG00000071644	11439	277,64
Zswim6	zinc finger SWIM-type containing 6	ENSMUSG00000032846	165447	277,64

Sema6a	sema domain, transmembrane domain (TM), and cytoplasmic domain, (semaphorin) 6A	ENSMUSG00000019647	133273	277,57
Hspd1	heat shock protein 1 (chaperonin)	ENSMUSG00000025980	10409	277,49
Fam168a	family with sequence similarity 168, member A	ENSMUSG00000029461	134978	277,20
		ENSMUSG00000029763	724895	277,06
Cdk8	cyclin-dependent kinase 8	ENSMUSG00000029635	71645	276,99
Picalm	phosphatidylinositol binding clathrin assembly protein	ENSMUSG00000039361	79216	275,83
Zfp207	zinc finger protein 207	ENSMUSG00000017421	22455	275,61
2010107E04Rik	RIKEN cDNA 2010107E04 gene	ENSMUSG00000021290	5602	275,32
Cadps2	Ca2+-dependent activator protein for secretion 2	ENSMUSG00000017978	576649	275,32
Rap1gap2	RAP1 GTPase activating protein 2	ENSMUSG00000038807	206831	275,25
Jmjd1c	jumonji domain containing 1C	ENSMUSG00000037876	160202	275,10
Birc6	baculoviral IAP repeat-containing 6	ENSMUSG00000024073	175479	274,59
Pitpna	phosphatidylinositol transfer protein, alpha	ENSMUSG00000017781	40708	274,45
Fam189a1	family with sequence similarity 189, member A1	ENSMUSG00000030518	400458	274,30
		ENSMUSG00000022747	56119	274,30
Emb	embigin	ENSMUSG00000021728	53474	274,23
Trim2	tripartite motif-containing 2	ENSMUSG00000027993	145008	274,16
Dip2c	DIP2 disco-interacting protein 2 homolog C (Drosophila)	ENSMUSG00000048264	392401	272,49
		ENSMUSG00000031558	323128	272,42
Psen2	presenilin 2	ENSMUSG00000010609	36435	271,11
		ENSMUSG00000020671	62540	271,04
Tmcc3	transmembrane and coiled coil domains 3	ENSMUSG00000020023	279008	270,90
Skp1a	S-phase kinase-associated protein 1A	ENSMUSG00000036309	14864	270,53
Ddx24	DEAD (Asp-Glu-Ala-Asp) box polypeptide 24	ENSMUSG00000041645	17892	270,46
Tnrc6a	trinucleotide repeat containing 6a	ENSMUSG00000052707	71404	270,46
Braf	Braf transforming gene	ENSMUSG00000002413	122227	269,59
Kdm2a	lysine (K)-specific demethylase 2A	ENSMUSG00000054611	82142	269,52
		ENSMUSG00000005161	5248	269,16
Mapkap1	mitogen-activated protein kinase associated protein 1	ENSMUSG00000038696	218180	268,94
Cecr2	cat eye syndrome chromosome region, candidate 2	ENSMUSG00000071226	104822	268,72
Rad23b	RAD23b homolog (S. cerevisiae)	ENSMUSG00000028426	42195	268,07
Nckap5	NCK-associated protein 5	ENSMUSG00000049690	917180	267,85
Ablim1	actin-binding LIM protein 1	ENSMUSG00000025085	282187	267,78
Lmo7	LIM domain only 7	ENSMUSG00000033060	204754	267,56
Golga4	golgi autoantigen, golgin subfamily a, 4	ENSMUSG00000038708	76205	267,42
Gab2	growth factor receptor bound protein 2-associated protein 2	ENSMUSG00000004508	227190	267,42
Kidins220	kinase D-interacting substrate 220	ENSMUSG00000036333	84766	267,27
Calb2	calbindin 2	ENSMUSG00000003657	25668	266,98
Lrig1	leucine-rich repeats and immunoglobulin-like domains 1	ENSMUSG00000030029	95630	266,62
		ENSMUSG00000001289	5758	266,26

Smarcc1	SWI/SNF related, matrix associated, actin dependent regulator of chromatin, subfamily c, member 1	ENSMUSG00000032481	108142	266,18
Unc13b	unc-13 homolog B (C. elegans)	ENSMUSG00000028456	205919	265,10
Rps14	ribosomal protein S14	ENSMUSG00000024608	4037	264,88
Ptbp3	polypyrimidine tract binding protein 3	ENSMUSG00000028382	77497	264,81
1700034F02Rik	RIKEN cDNA 1700034F02 gene	ENSMUSG00000020461	30418	264,52
P4hb	prolyl 4-hydroxylase, beta polypeptide	ENSMUSG00000025130	12956	264,08
Fto	fat mass and obesity associated	ENSMUSG00000055932	354908	263,94
Sik3	SIK family kinase 3	ENSMUSG00000034135	211375	263,79
Irs2	insulin receptor substrate 2	ENSMUSG00000038894	21479	263,79
Tgfb2	transforming growth factor, beta 2	ENSMUSG00000039239	82807	263,43
Serbp1	serpine1 mRNA binding protein 1	ENSMUSG00000036371	22324	263,21
Dazap2	DAZ associated protein 2	ENSMUSG00000000346	5104	262,85
Anxa2	annexin A2	ENSMUSG00000032231	38176	262,56
Ell3	elongation factor RNA polymerase II-like 3	ENSMUSG00000027246	5269	262,34
Sh3pxd2a	SH3 and PX domains 2A	ENSMUSG00000053617	204238	262,27
		ENSMUSG00000031812	8400	262,05
Eif3h	eukaryotic translation initiation factor 3, subunit H	ENSMUSG00000022312	78924	261,98
Cyb5r3	cytochrome b5 reductase 3	ENSMUSG00000018042	19099	261,83
		ENSMUSG00000052942	421227	261,83
Bcl11a	B cell CLL/lymphoma 11A (zinc finger protein)	ENSMUSG00000000861	96068	261,62
St6gal1	beta galactoside alpha 2,6 sialyltransferase 1	ENSMUSG00000022885	135611	261,33
Rab1	RAB1, member RAS oncogene family	ENSMUSG00000020149	25425	261,18
Rbbp6	retinoblastoma binding protein 6	ENSMUSG00000030779	36872	261,11
Ulk4	unc-51-like kinase 4	ENSMUSG00000040936	316930	260,68
Srgap3	SLIT-ROBO Rho GTPase activating protein 3	ENSMUSG00000030257	229296	260,53
Rps15	ribosomal protein S15	ENSMUSG00000063457	1662	260,24
Dcn	decorin	ENSMUSG00000019929	38663	260,02
Wdfy3	WD repeat and FYVE domain containing 3	ENSMUSG00000043940	236966	259,44
Dnajc6	DnaJ (Hsp40) homolog, subfamily C, member 6	ENSMUSG00000028528	146152	259,30
Creg1	cellular repressor of E1A-stimulated genes 1	ENSMUSG00000040713	11563	259,23
Arpc1a	actin related protein 2/3 complex, subunit 1A	ENSMUSG00000029621	24932	258,79
Mex3a	mex3 homolog A (C. elegans)	ENSMUSG00000074480	9002	258,72
Schip1	schwannomin interacting protein 1	ENSMUSG00000027777	561680	258,72
Osbpl1a	oxysterol binding protein-like 1A	ENSMUSG00000044252	186528	258,65
Atxn1	ataxin 1	ENSMUSG00000046876	415234	258,57
Ldhb	lactate dehydrogenase B	ENSMUSG00000030246	17709	258,28
Dennd1a	DENN/MADD domain containing 1A	ENSMUSG00000035392	488400	257,99
Glg1	golgi apparatus protein 1	ENSMUSG00000003316	104796	257,92
Stag1	stromal antigen 1	ENSMUSG00000037286	360748	257,63
Cd36	CD36 antigen	ENSMUSG00000002944	107270	257,49

Irf2bp2	interferon regulatory factor 2 binding protein 2	ENSMUSG00000051495	5141	257,41
Iqgap2	IQ motif containing GTPase activating protein 2	ENSMUSG00000021676	264746	257,41
A830018L16Rik	RIKEN cDNA A830018L16 gene	ENSMUSG00000057715	561797	257,20
Cdc26	cell division cycle 26	ENSMUSG00000066149	24989	257,05
Bace1	beta-site APP cleaving enzyme 1	ENSMUSG00000032086	25820	256,83
Zzef1	zinc finger, ZZ-type with EF hand domain 1	ENSMUSG00000055670	130895	256,54
Ptpn3	protein tyrosine phosphatase, non-receptor type 3	ENSMUSG00000038764	110997	255,75
Tmbim6	transmembrane BAX inhibitor motif containing 6	ENSMUSG00000023010	17168	255,67
Fbxo32	F-box protein 32	ENSMUSG00000022358	39014	255,67
		ENSMUSG00000079735	226314	255,46
Rps11	ribosomal protein S11	ENSMUSG00000003429	2002	255,24
Kcnmb2	potassium large conductance calcium-activated channel, subfamily M, beta member 2	ENSMUSG00000037610	297674	254,08
Unc45a	unc-45 homolog A (C. elegans)	ENSMUSG00000030533	15714	253,72
Bri3	brain protein I3	ENSMUSG00000047843	202321	252,92
		ENSMUSG00000023965	442566	252,92
Nsg2	neuron specific gene family member 2	ENSMUSG00000020297	58740	252,27
Bcl2l1	BCL2-like 1	ENSMUSG00000007659	51061	251,90
Golgb1	golgi autoantigen, golgin subfamily b, macrogolgin 1	ENSMUSG00000034243	57946	251,61
Senp6	SUMO/sentrin specific peptidase 6	ENSMUSG00000034252	78051	251,54
Cnot1	CCR4-NOT transcription complex, subunit 1	ENSMUSG00000036550	88012	251,54
Rpl10-ps3	ribosomal protein L10, pseudogene 3	ENSMUSG00000058443	742	251,40
Arl14epl	ADP-ribosylation factor-like 14 effector protein-like	ENSMUSG00000073568	8241	251,32
Il31ra	interleukin 31 receptor A	ENSMUSG00000050377	57853	251,32
Tsc22d1	TSC22 domain family, member 1	ENSMUSG00000022010	92805	251,03
Rap1gds1	RAP1, GTP-GDP dissociation stimulator 1	ENSMUSG00000028149	149294	250,96
Plce1	phospholipase C, epsilon 1	ENSMUSG00000024998	303922	250,74
Sec61b	Sec61 beta subunit	ENSMUSG00000053317	8585	250,53
Fmo6	flavin containing monooxygenase 6	ENSMUSG00000095576	20675	250,45
Adnp	activity-dependent neuroprotective protein	ENSMUSG00000051149	26127	250,24
		ENSMUSG00000028639	16624	250,02
Mphosph8	M-phase phosphoprotein 8	ENSMUSG00000079184	29183	249,95
Cox6b1	cytochrome c oxidase, subunit VIb polypeptide 1	ENSMUSG00000036751	9178	249,87
Stt3b	STT3, subunit of the oligosaccharyltransferase complex, homolog B (S. cerevisiae)	ENSMUSG00000032437	67815	249,29
Arid1a	AT rich interactive domain 1A (SWI-like)	ENSMUSG00000007880	77762	249,22
Cstf3	cleavage stimulation factor, 3' pre-RNA, subunit 3	ENSMUSG00000027176	74907	248,64
Rac1	RAS-related C3 botulinum substrate 1	ENSMUSG00000001847	22559	248,50

Cbx4	chromobox 4	ENSMUSG00000039989	8649	248,42
Ncam2	neural cell adhesion molecule 2	ENSMUSG00000022762	423589	247,41
Dscam	Down syndrome cell adhesion molecule	ENSMUSG00000050272	578674	247,12
Phlpp1	PH domain and leucine rich repeat protein phosphatase 1	ENSMUSG00000044340	222499	247,12
Sart3	squamous cell carcinoma antigen recognized by T cells 3	ENSMUSG00000018974	29204	247,05
Atp6v1a	ATPase, H+ transporting, lysosomal V1 subunit A	ENSMUSG00000052459	54304	247,05
1700028P14Rik	RIKEN cDNA 1700028P14 gene	ENSMUSG00000033053	94053	246,83
Dnajc5	DnaJ (Hsp40) homolog, subfamily C, member 5	ENSMUSG00000000826	34649	246,54
Chd4	chromodomain helicase DNA binding protein 4	ENSMUSG00000063870	34611	246,18
Kansl1	KAT8 regulatory NSL complex subunit 1	ENSMUSG00000018412	135633	246,03
		ENSMUSG00000025352	9424	245,81
Zfp664	zinc finger protein 664	ENSMUSG00000079215	40003	245,45
Phb2	prohibitin 2	ENSMUSG00000004264	4615	245,31
Ptprk	protein tyrosine phosphatase, receptor type, K	ENSMUSG00000019889	522578	245,23
a	nonagouti	ENSMUSG00000027596	259611	245,16
Rpl13a	ribosomal protein L13A	ENSMUSG00000074129	10674	245,09
Sub1	SUB1 homolog (S. cerevisiae)	ENSMUSG00000022205	15645	244,80
Ythdf2	YTH domain family 2	ENSMUSG00000040025	27392	244,51
Uchl1	ubiquitin carboxy-terminal hydrolase L1	ENSMUSG00000029223	11134	244,44
Pon3	paraoxonase 3	ENSMUSG00000029759	35435	244,00
Ctla4	cytotoxic T-lymphocyte-associated protein 4	ENSMUSG00000026011	28833	243,78
Rpl36	ribosomal protein L36	ENSMUSG00000057863	849	243,13
Ftl1	ferritin light chain 1	ENSMUSG00000050708	1941	243,06
Zbtb7a	zinc finger and BTB domain containing 7a	ENSMUSG00000035011	17776	243,06
Snapc3	small nuclear RNA activating complex, polypeptide 3	ENSMUSG00000028483	49953	243,06
Klf4	Kruppel-like factor 4 (gut)	ENSMUSG00000003032	5324	242,70
Esrp1	epithelial splicing regulatory protein 1	ENSMUSG00000040728	54851	242,26
AU021092	expressed sequence AU021092	ENSMUSG00000051669	10472	242,12
Mettl23	methyltransferase like 23	ENSMUSG00000090266	10986	241,83
Gapdh	glyceraldehyde-3-phosphate dehydrogenase	ENSMUSG00000057666	4753	241,54
Nfia	nuclear factor I/A	ENSMUSG00000028565	346141	241,25
Ube2d3	ubiquitin-conjugating enzyme E2D 3	ENSMUSG00000078578	28882	241,03
Arhgef2	rho/rac guanine nucleotide exchange factor (GEF) 2	ENSMUSG00000028059	40599	240,89
		ENSMUSG00000026667	21978	240,74
Ankrd17	ankyrin repeat domain 17	ENSMUSG00000055204	138996	240,52
Atp5g3	ATP synthase, H+ transporting, mitochondrial F0 complex, subunit C3 (subunit 9)	ENSMUSG00000018770	2880	240,23
Hnrnpc	heterogeneous nuclear ribonucleoprotein C	ENSMUSG00000060373	30649	240,02

Cept1	choline/ethanolaminephosphotransferase 1	ENSMUSG00000040774	45543	240,02
Sbno1	sno, strawberry notch homolog 1 (Drosophila)	ENSMUSG00000038095	57213	239,80
Brd3	bromodomain containing 3	ENSMUSG00000026918	62084	239,73
Id1	inhibitor of DNA binding 1	ENSMUSG00000042745	1160	238,86
Sertad2	SERTA domain containing 2	ENSMUSG00000049800	109769	238,49
Ifitm1	interferon induced transmembrane protein 1	ENSMUSG00000025491	2605	238,42
Lpin1	lipin 1	ENSMUSG00000020593	54102	238,35
Dcx	doublecortin	ENSMUSG00000031285	77470	238,28
Stk32a	serine/threonine kinase 32A	ENSMUSG00000039954	109785	238,13
Arid5b	AT rich interactive domain 5B (MRF1-like)	ENSMUSG00000019947	183134	237,84
Hist1h2bc	histone cluster 1, H2bc	ENSMUSG00000018102	8290	237,70
Hecw1	HECT, C2 and WW domain containing E3 ubiquitin protein ligase 1	ENSMUSG00000021301	296789	237,70
Prdm16	PR domain containing 16	ENSMUSG00000039410	320749	237,48
Prkca	protein kinase C, alpha	ENSMUSG00000050965	410542	237,48
Clu	clusterin	ENSMUSG00000022037	13066	237,41
Srek1	splicing regulatory glutamine/lysine-rich protein 1	ENSMUSG00000032621	22890	236,97
Gm10260	predicted gene 10260	ENSMUSG00000069117	459	236,90
Trim44	tripartite motif-containing 44	ENSMUSG00000027189	107710	236,83
Zcchc11	zinc finger, CCHC domain containing 11	ENSMUSG00000034610	99996	236,75
Aqp5	aquaporin 5	ENSMUSG00000044217	3981	236,39
Ptp4a1	protein tyrosine phosphatase 4a1	ENSMUSG00000026064	9468	236,32
		ENSMUSG00000001552	28806	236,17
		ENSMUSG00000020456	65010	236,03
Serinc4	serine incorporator 4	ENSMUSG00000046110	17723	235,74
Mtmr3	myotubularin related protein 3	ENSMUSG00000034354	113996	235,59
Cnn3	calponin 3, acidic	ENSMUSG00000053931	31665	234,87
		ENSMUSG00000025246	151971	234,58
Rps18-ps3	ribosomal protein S18, pseudogene 3	ENSMUSG00000057657	432	234,29
		ENSMUSG00000020451	64934	234,29
		ENSMUSG00000030068	409314	234,22
Rbm4	RNA binding motif protein 4	ENSMUSG00000096370	68700	234,00
Erbb2ip	Erbb2 interacting protein	ENSMUSG00000021709	101728	233,93
		ENSMUSG00000061731	282122	233,78
Mllt4	myeloid/lymphoid or mixed-lineage leukemia (trithorax homolog, Drosophila); translocated to, 4	ENSMUSG00000068036	145612	233,56
Mllt10	myeloid/lymphoid or mixed-lineage leukemia (trithorax homolog, Drosophila); translocated to, 10	ENSMUSG00000026743	157152	233,56
Atp2b1	ATPase, Ca++ transporting, plasma membrane 1	ENSMUSG00000019943	110992	233,49
Dcaf11	DDB1 and CUL4 associated factor 11	ENSMUSG00000022214	10060	233,35
Mbtd1	mbt domain containing 1	ENSMUSG00000059474	61134	232,91
Junb	Jun-B oncogene	ENSMUSG00000052837	1840	232,69
Ehbp1	EH domain binding protein 1	ENSMUSG00000042302	336465	232,19
Zfp148	zinc finger protein 148	ENSMUSG00000022811	123129	231,82

Vcl	vinculin	ENSMUSG00000021823	104241	231,46
Cryl1	crystallin, lambda 1	ENSMUSG00000021947	123450	231,39
Cxadr	coxsackie virus and adenovirus receptor	ENSMUSG00000022865	58290	231,32
Ctsb	cathepsin B	ENSMUSG00000021939	23458	231,24
Pabpc1	poly(A) binding protein, cytoplasmic 1	ENSMUSG00000022283	13313	230,37
Hlf	hepatic leukemia factor	ENSMUSG00000003949	54360	230,23
Fus	fused in sarcoma	ENSMUSG00000030795	14912	229,87
Huwe1	HECT, UBA and WWE domain containing 1	ENSMUSG00000025261	134611	229,79
Rbbp4	retinoblastoma binding protein 4	ENSMUSG00000057236	28271	229,65
Diap1	diaphanous homolog 1 (Drosophila)	ENSMUSG00000024456	91876	229,65
Cebpg	CCAAT/enhancer binding protein (C/EBP), gamma	ENSMUSG00000056216	10082	229,50
Zfp292	zinc finger protein 292	ENSMUSG00000039967	79848	229,43
Rpl10a	ribosomal protein L10A	ENSMUSG00000037805	2562	229,36
Gls	glutaminase	ENSMUSG00000026103	69785	229,36
Got2	glutamate oxaloacetate transaminase 2, mitochondrial	ENSMUSG00000031672	24414	229,29
Ulbp1	UL16 binding protein 1	ENSMUSG00000079685	28605	229,07
Adi1	acireductone dioxygenase 1	ENSMUSG00000020629	6945	229,07
Nudt4	nudix (nucleoside diphosphate linked moiety X)-type motif 4	ENSMUSG00000020029	17161	228,42
Larp1	La ribonucleoprotein domain family, member 1	ENSMUSG00000037331	52971	228,34
Pepd	peptidase D	ENSMUSG00000063931	132332	228,05
		ENSMUSG00000039202	92308	227,55
Sfrs18	serine/arginine-rich splicing factor 18	ENSMUSG00000028248	28893	227,55
Dicer1	dicer 1, ribonuclease type III	ENSMUSG00000041415	64211	227,26
		ENSMUSG00000022905	58382	226,89
		ENSMUSG00000020359	19733	226,75
Phf21a	PHD finger protein 21A	ENSMUSG00000058318	271550	226,68
Srsf1	serine/arginine-rich splicing factor 1	ENSMUSG00000018379	6383	226,10
Osbpl10	oxysterol binding protein-like 10	ENSMUSG00000040875	253657	225,95
Eml6	echinoderm microtubule associated protein like 6	ENSMUSG00000044072	282986	225,66
Ankrd11	ankyrin repeat domain 11	ENSMUSG00000035569	91457	225,23
Syt7	synaptotagmin VII	ENSMUSG00000024743	64092	224,86
Ddr1	discoidin domain receptor family, member 1	ENSMUSG00000003534	23055	224,86
Tbc1d9	TBC1 domain family, member 9	ENSMUSG00000031709	107583	224,72
Ankrd10	ankyrin repeat domain 10	ENSMUSG00000031508	24172	224,43
Luc7l3	LUC7-like 3 (S. cerevisiae)	ENSMUSG00000020863	34099	224,21
Rb1cc1	RB1-inducible coiled-coil 1	ENSMUSG00000025907	70452	224,21
Rps28	ribosomal protein S28	ENSMUSG00000067288	5536	224,14
		ENSMUSG00000042541	21365	224,07
Rpn2	ribophorin II	ENSMUSG00000027642	47303	223,85
		ENSMUSG00000061578	361004	223,49
Zcchc7	zinc finger, CCHC domain containing 7	ENSMUSG00000035649	176339	223,13
Alms1	Alstrom syndrome 1	ENSMUSG00000063810	115223	222,98
Ppp1r15a	protein phosphatase 1, regulatory (inhibitor) subunit 15A	ENSMUSG00000040435	3353	222,84
Fbxo11	F-box protein 11	ENSMUSG00000005371	74433	222,69

Rbm6	RNA binding motif protein 6	ENSMUSG00000032582	99679	222,62
Whsc1l1	Wolf-Hirschhorn syndrome candidate 1-like 1 (human)	ENSMUSG00000054823	118067	222,33
Rif1	Rap1 interacting factor 1 homolog (yeast)	ENSMUSG00000036202	49547	221,82
Pex5l	peroxisomal biogenesis factor 5-like	ENSMUSG00000027674	193840	221,60
Trp53inp2	transformation related protein 53 inducible nuclear protein 2	ENSMUSG00000038375	8792	221,46
Ppfia2	protein tyrosine phosphatase, receptor type, f polypeptide (PTPRF), interacting protein (liprin), alpha 2	ENSMUSG00000053825	463158	221,46
Smchd1	SMC hinge domain containing 1	ENSMUSG00000024054	130855	221,24
Ssbp3	single-stranded DNA binding protein 3	ENSMUSG00000061887	138994	221,10
Zc3h13	zinc finger CCCH type containing 13	ENSMUSG00000022000	60054	221,10
Glrx5	glutaredoxin 5 homolog (S. cerevisiae)	ENSMUSG00000021102	8222	221,02
		ENSMUSG00000005534	128696	220,73
		ENSMUSG00000043991	20425	220,59
Hmcn1	hemicentin 1	ENSMUSG00000066842	429909	220,44
Cyfip2	cytoplasmic FMR1 interacting protein 2	ENSMUSG00000020340	119010	220,30
March6	membrane-associated ring finger (C3HC4) 6	ENSMUSG00000039100	75139	220,30
Glyr1	glyoxylate reductase 1 homolog (Arabidopsis)	ENSMUSG00000022536	36005	220,01
Pik3r3	phosphatidylinositol 3 kinase, regulatory subunit, polypeptide 3 (p55)	ENSMUSG00000028698	81439	220,01
B230219D22Rik	RIKEN cDNA B230219D22 gene	ENSMUSG00000045767	10376	219,86
Lcorl	ligand dependent nuclear receptor corepressor-like	ENSMUSG00000015882	160435	219,79
Oaz1	ornithine decarboxylase antizyme 1	ENSMUSG00000035242	2635	219,50
Appl1	adaptor protein, phosphotyrosine interaction, PH domain and leucine zipper containing 1	ENSMUSG00000040760	52245	219,21
Hsp90b1	heat shock protein 90, beta (Grp94), member 1	ENSMUSG00000020048	15301	218,85
		ENSMUSG00000056050	42805	218,78
Brd4	bromodomain containing 4	ENSMUSG00000024002	88449	218,70
Mast4	microtubule associated serine/threonine kinase family member 4	ENSMUSG00000034751	602012	218,49
Pkm	pyruvate kinase, muscle	ENSMUSG00000032294	23008	218,34
Cradd	CASP2 and RIPK1 domain containing adaptor with death domain	ENSMUSG00000045867	149352	218,27
Ube2r2	ubiquitin-conjugating enzyme E2R 2	ENSMUSG00000036241	57638	217,98
Atpif1	ATPase inhibitory factor 1	ENSMUSG00000054428	3105	217,91
Arrdc3	arrestin domain containing 3	ENSMUSG00000074794	12652	217,62
Osbpl3	oxysterol binding protein-like 3	ENSMUSG00000029822	162872	217,62

		ENSMUSG00000034336	10630	217,47
		ENSMUSG00000095597	652	217,40
		ENSMUSG00000049606	196394	217,18
Agpat3	1-acylglycerol-3-phosphate O-acyltransferase 3	ENSMUSG00000001211	83312	217,11
Mapre2	microtubule-associated protein, RP/EB family, member 2	ENSMUSG00000024277	141529	216,89
Pkn2	protein kinase N2	ENSMUSG00000004591	91103	216,75
Tax1bp1	Tax1 (human T cell leukemia virus type I) binding protein 1	ENSMUSG00000004535	53052	216,67
Nrp1	neuropilin 1	ENSMUSG00000025810	146390	216,60
Nup93	nucleoporin 93	ENSMUSG00000032939	100470	216,31
Polr2m	polymerase (RNA) II (DNA directed) polypeptide M	ENSMUSG00000032199	7499	216,09
Yeats2	YEATS domain containing 2	ENSMUSG00000041215	91511	215,88
Pkia	protein kinase inhibitor, alpha	ENSMUSG00000027499	78763	215,22
Btg2	B cell translocation gene 2, anti-proliferative	ENSMUSG00000020423	3951	215,08
Tardbp	TAR DNA binding protein	ENSMUSG00000041459	14638	215,01
		ENSMUSG00000031715	40991	214,79
Trip12	thyroid hormone receptor interactor 12	ENSMUSG00000026219	118116	214,72
		ENSMUSG00000021131	10320	214,57
Tpm1	tropomyosin 1, alpha	ENSMUSG00000032366	26817	214,28
Ythdf3	YTH domain family 3	ENSMUSG00000047213	33853	214,14
Cox6c	cytochrome c oxidase subunit VIc	ENSMUSG00000014313	12361	214,06
Grhl1	grainyhead-like 1 (Drosophila)	ENSMUSG00000020656	45105	213,77
Vmn2r53	vomeronasal 2, receptor 53	ENSMUSG00000096002	25075	213,70
Rab11fip1	RAB11 family interacting protein 1 (class I)	ENSMUSG00000031488	35874	213,48
Ncor1	nuclear receptor co-repressor 1	ENSMUSG00000018501	142116	213,34
Gnb1l	guanine nucleotide binding protein (G protein), beta polypeptide 1-like	ENSMUSG00000000884	67912	213,05
Spats1	spermatogenesis associated, serine-rich 1	ENSMUSG00000023935	26002	212,98
Ip6k2	inositol hexaphosphate kinase 2	ENSMUSG00000032599	10340	212,54
Ctdsp2	CTD (carboxy-terminal domain, RNA polymerase II, polypeptide A) small phosphatase 2	ENSMUSG00000078429	21259	212,54
Dnajb6	DnaJ (Hsp40) homolog, subfamily B, member 6	ENSMUSG00000029131	50791	212,40
Sltm	SAFB-like, transcription modulator	ENSMUSG00000032212	49318	212,03
Usp9x	ubiquitin specific peptidase 9, X chromosome	ENSMUSG00000031010	101831	211,82
Psip1	PC4 and SFRS1 interacting protein 1	ENSMUSG00000028484	30780	211,60
Sgms1	sphingomyelin synthase 1	ENSMUSG00000040451	266988	211,60
		ENSMUSG00000022421	47982	211,53
Hipk2	homeodomain interacting protein kinase 2	ENSMUSG00000061436	181776	211,53
Tjp2	tight junction protein 2	ENSMUSG00000024812	130504	211,53
Akt3	thymoma viral proto-oncogene 3	ENSMUSG00000019699	238131	211,45
Rapgef6	Rap guanine nucleotide exchange factor (GEF) 6	ENSMUSG00000037533	176439	211,31
		ENSMUSG00000027744	19776	210,87
		ENSMUSG00000022186	128962	210,87

Rpl15	ribosomal protein L15	ENSMUSG00000012405	3569	210,80
Gm10275	predicted pseudogene 10275	ENSMUSG00000069682	634	210,58
		ENSMUSG00000037857	55767	210,44
Atrnl1	attractin like 1	ENSMUSG00000054843	522305	210,44
Foxo1	forkhead box O1	ENSMUSG00000044167	81773	210,37
Calm2	calmodulin 2	ENSMUSG00000036438	13524	210,29
Plcb4	phospholipase C, beta 4	ENSMUSG00000039943	355583	210,08
Arhgef28	Rho guanine nucleotide exchange factor (GEF) 28	ENSMUSG00000021662	307571	210,00
Sypl	synaptophysin-like protein	ENSMUSG00000020570	25160	209,71
		ENSMUSG00000029578	41047	209,64
		ENSMUSG00000020349	29098	209,57
Myo1d	myosin ID	ENSMUSG00000035441	297900	209,50
Aldoa	aldolase A, fructose-bisphosphate	ENSMUSG00000030695	5518	208,99
Ctsd	cathepsin D	ENSMUSG00000007891	16891	208,99
Canx	calnexin	ENSMUSG00000020368	31713	208,92
Igf2bp2	insulin-like growth factor 2 mRNA binding protein 2	ENSMUSG00000033581	104291	208,77
Rab39	RAB39, member RAS oncogene family	ENSMUSG00000055069	22123	208,55
Setd5	SET domain containing 5	ENSMUSG00000034269	76071	208,55
Vamp2	vesicle-associated membrane protein 2	ENSMUSG00000020894	3895	208,48
Pan3	PAN3 polyA specific ribonuclease subunit homolog (S. cerevisiae)	ENSMUSG00000029647	118342	208,41
Cux1	cut-like homeobox 1	ENSMUSG00000029705	319356	208,26
Rarb	retinoic acid receptor, beta	ENSMUSG00000017491	144631	208,19
Sqle	squalene epoxidase	ENSMUSG00000022351	16116	207,76
Ccdc171	coiled-coil domain containing 171	ENSMUSG00000052407	339126	207,76
Mprip	myosin phosphatase Rho interacting protein	ENSMUSG00000005417	119556	207,54
Cyp2j6	cytochrome P450, family 2, subfamily j, polypeptide 6	ENSMUSG00000052914	37524	207,47
Ppp1r9a	protein phosphatase 1, regulatory (inhibitor) subunit 9A	ENSMUSG00000032827	262745	207,47
Naip1	NLR family, apoptosis inhibitory protein 1	ENSMUSG00000021640	38637	207,39
		ENSMUSG00000021218	28187	207,32
Mdh1	malate dehydrogenase 1, NAD (soluble)	ENSMUSG00000020321	15581	207,32
Srrm1	serine/arginine repetitive matrix 1	ENSMUSG00000028809	32838	207,18
Ubqln2	ubiquilin 2	ENSMUSG00000050148	3306	207,03
Zmynd11	zinc finger, MYND domain containing 11	ENSMUSG00000021156	80486	206,81
Hist2h2aa1	histone cluster 2, H2aa1	ENSMUSG00000063954	578	206,52
Tmem63b	transmembrane protein 63b	ENSMUSG00000036026	26735	206,38
Sf1	splicing factor 1	ENSMUSG00000024949	14341	206,38
Tmem57	transmembrane protein 57	ENSMUSG00000028826	50587	206,38
		ENSMUSG00000021730	374323	206,38
Slc38a2	solute carrier family 38, member 2	ENSMUSG00000022462	12305	206,09
		ENSMUSG00000018286	2492	205,51
Wdr26	WD repeat domain 26	ENSMUSG00000038733	38325	205,51
Nck2	non-catalytic region of tyrosine kinase adaptor protein 2	ENSMUSG00000066877	124765	205,44
Ddb1	damage specific DNA binding protein 1	ENSMUSG00000024740	24189	205,22

Ano6	anoctamin 6	ENSMUSG00000064210	184622	205,22
Ccni	cyclin I	ENSMUSG00000063015	24563	205,15
Spop	speckle-type POZ protein	ENSMUSG00000057522	79327	205,00
Pomp	proteasome maturation protein	ENSMUSG00000029649	15151	205,00
Cisd1	CDGSH iron sulfur domain 1	ENSMUSG00000037710	14475	204,93
Rps16	ribosomal protein S16	ENSMUSG00000037563	2045	204,35
Ascc2	activating signal cointegrator 1 complex subunit 2	ENSMUSG00000020412	47953	204,28
Ube2d2a	ubiquitin-conjugating enzyme E2D 2A	ENSMUSG00000091896	35614	204,21
Nfatc3	nuclear factor of activated T cells, cytoplasmic, calcineurin dependent 3	ENSMUSG00000031902	70935	204,21
Rpl10	ribosomal protein L10	ENSMUSG00000008682	2324	204,13
Eif4g3	eukaryotic translation initiation factor 4 gamma, 3	ENSMUSG00000028760	215487	203,63
Prkce	protein kinase C, epsilon	ENSMUSG00000045038	490135	203,48
Thoc2	THO complex 2	ENSMUSG00000037475	125684	203,19
H2afy	H2A histone family, member Y	ENSMUSG00000015937	62743	202,90
		ENSMUSG00000061136	56581	202,90
1700112E06Rik	RIKEN cDNA 1700112E06 gene	ENSMUSG00000063458	1036374	202,90
		ENSMUSG00000028495	2753	202,76
Sf3b2	splicing factor 3b, subunit 2	ENSMUSG00000024853	21533	202,32
Phactr2	phosphatase and actin regulator 2	ENSMUSG00000062866	266680	202,32
Rpl23	ribosomal protein L23	ENSMUSG00000071415	4911	202,25
Foxo3	forkhead box O3	ENSMUSG00000048756	94915	201,89
D4Wsu53e	DNA segment, Chr 4, Wayne State University 53, expressed	ENSMUSG00000037266	4080	201,81
Maged1	melanoma antigen, family D, 1	ENSMUSG00000025151	6670	201,81
Zfr	zinc finger RNA binding protein	ENSMUSG00000022201	67853	201,60
Tead1	TEA domain family member 1	ENSMUSG00000055320	227490	201,09
Aqp4	aquaporin 4	ENSMUSG00000024411	14291	200,80
Srgap2	SLIT-ROBO Rho GTPase activating protein 2	ENSMUSG00000026425	242111	200,73
Yy1	YY1 transcription factor	ENSMUSG00000021264	23660	200,51
Snrnp70	small nuclear ribonucleoprotein 70 (U1)	ENSMUSG00000063511	19231	200,44
Marcksl1	MARCKS-like 1	ENSMUSG00000047945	2405	200,36
Shank2	SH3/ankyrin domain gene 2	ENSMUSG00000037541	422567	200,15
Set	SET nuclear oncogene	ENSMUSG00000054766	15200	199,93
Hsd17b4	hydroxysteroid (17-beta) dehydrogenase 4	ENSMUSG00000024507	68069	199,93
Gm10020	predicted pseudogene 10020	ENSMUSG00000057262	615	199,64
Fam101b	family with sequence similarity 101, member B	ENSMUSG00000020846	8589	199,64
Dynll1	dynein light chain LC8-type 1	ENSMUSG00000009013	3890	199,42
Pcbp2	poly(rC) binding protein 2	ENSMUSG00000056851	29428	199,42
Nr6a1	nuclear receptor subfamily 6, group A, member 1	ENSMUSG00000063972	204319	199,42
Cspp1	centrosome and spindle pole associated protein 1	ENSMUSG00000056763	98551	199,35
Ndufa6	NADH dehydrogenase (ubiquinone) 1 alpha subcomplex, 6 (B14)	ENSMUSG00000022450	4153	198,77
Baz1b	bromodomain adjacent to zinc finger domain, 1B	ENSMUSG00000002748	58866	198,62
Lrrc59	leucine rich repeat containing 59	ENSMUSG00000020869	15450	198,41

Rpn1	ribophorin I	ENSMUSG00000030062	20832	198,41
Itpr1	inositol 1,4,5-trisphosphate receptor 1	ENSMUSG00000030102	337992	198,41
Dpysl5	dihydropyrimidinase-like 5	ENSMUSG00000029168	87812	198,12
Akr1a1	aldo-keto reductase family 1, member A1 (aldehyde reductase)	ENSMUSG00000028692	15171	198,12
Rabgap1	RAB GTPase activating protein 1	ENSMUSG00000035437	123176	197,83
Ralgapa2	Ral GTPase activating protein, alpha subunit 2 (catalytic)	ENSMUSG00000037110	272148	197,61
C330007P06Rik	RIKEN cDNA C330007P06 gene	ENSMUSG00000006423	50375	197,46
Bzw2	basic leucine zipper and W2 domains 2	ENSMUSG00000020547	64980	197,10
Rc3h2	ring finger and CCCH-type zinc finger domains 2	ENSMUSG00000075376	52835	196,88
Phf20	PHD finger protein 20	ENSMUSG00000038116	113487	196,74
Zfp704	zinc finger protein 704	ENSMUSG00000040209	183075	196,74
Bcas3	breast carcinoma amplified sequence 3	ENSMUSG00000059439	472892	196,67
Arhgef38	Rho guanine nucleotide exchange factor (GEF) 38	ENSMUSG00000040969	122672	196,67
Map3k12	mitogen-activated protein kinase kinase kinase 12	ENSMUSG00000023050	19416	196,59
Cacna1d	calcium channel, voltage-dependent, L type, alpha 1D subunit	ENSMUSG00000015968	451208	196,59
Eprs	glutamyl-prolyl-tRNA synthetase	ENSMUSG00000026615	65261	196,45
Atp6v1g1	ATPase, H+ transporting, lysosomal V1 subunit G1	ENSMUSG00000039105	5979	196,09
Arhgap6	Rho GTPase activating protein 6	ENSMUSG00000031355	509337	196,09
Scaper	S phase cyclin A-associated protein in the ER	ENSMUSG00000034007	369723	196,01
		ENSMUSG00000052572	1356298	195,80
Igf2r	insulin-like growth factor 2 receptor	ENSMUSG00000023830	87259	195,80
Nr3c2	nuclear receptor subfamily 3, group C, member 2	ENSMUSG00000031618	345571	195,80
Tmem50a	transmembrane protein 50A	ENSMUSG00000028822	17176	195,65
Gbf1	golgi-specific brefeldin A-resistance factor 1	ENSMUSG00000025224	134002	195,65
Etv6	ets variant gene 6 (TEL oncogene)	ENSMUSG00000030199	234459	195,36
Trappc9	trafficking protein particle complex 9	ENSMUSG00000047921	471584	195,00
Gapvd1	GTPase activating protein and VPS9 domains 1	ENSMUSG00000026867	79049	194,93
Nampt	nicotinamide phosphoribosyltransferase	ENSMUSG00000020572	33035	194,85
Ccnd1	cyclin D1	ENSMUSG00000070348	9939	194,71
Nav2	neuron navigator 2	ENSMUSG00000052512	363899	194,49
Aplp1	amyloid beta (A4) precursor-like protein 1	ENSMUSG00000006651	10602	194,42
		ENSMUSG00000008333	9815	194,42
Tmem178b	transmembrane protein 178B	ENSMUSG00000057716	381880	194,42
Pam	peptidylglycine alpha-amidating monooxygenase	ENSMUSG00000026335	300533	194,35
Med12l	mediator of RNA polymerase II transcription, subunit 12 homolog (yeast)-like	ENSMUSG00000056476	311433	194,20

Gm581	predicted gene 581	ENSMUSG00000095276	3249	193,91
Uvrag	UV radiation resistance associated gene	ENSMUSG00000035354	254402	193,91
Ncoa2	nuclear receptor coactivator 2	ENSMUSG00000005886	234979	193,84
		ENSMUSG00000028439	51848	193,77
Camsap1	calmodulin regulated spectrin-associated protein 1	ENSMUSG00000026933	56445	193,48
Olfm1	olfactomedin 1	ENSMUSG00000026833	37745	193,33
Rabgap1l	RAB GTPase activating protein 1-like	ENSMUSG00000026721	573765	193,04
Herc2	hect (homologous to the E6-AP (UBE3A) carboxyl terminus) domain and RCC1 (CHC1)-like domain (RLD) 2	ENSMUSG00000030451	181640	192,97
Kdm5a	lysine (K)-specific demethylase 5A	ENSMUSG00000030180	80476	192,90
Bbx	bobby sox homolog (Drosophila)	ENSMUSG00000022641	240547	192,82
Cnbp	cellular nucleic acid binding protein	ENSMUSG00000030057	8492	192,75
Chtop	chromatin target of PRMT1	ENSMUSG00000001017	10543	192,68
Faah	fatty acid amide hydrolase	ENSMUSG00000034171	50782	192,61
Fam193a	family with sequence similarity 193, member A	ENSMUSG00000037210	116524	192,61
Sec61a1	Sec61 alpha 1 subunit (S. cerevisiae)	ENSMUSG00000030082	15305	192,46
2700089E24Rik	RIKEN cDNA 2700089E24 gene	ENSMUSG00000072704	2509	192,39
Arhgap32	Rho GTPase activating protein 32	ENSMUSG00000041444	152311	192,39
Etf1	eukaryotic translation termination factor 1	ENSMUSG00000024360	29223	192,32
Cyp1a2	cytochrome P450, family 1, subfamily a, polypeptide 2	ENSMUSG00000032310	6719	192,03
		ENSMUSG00000079641	2883	191,95
Csnk1g1	casein kinase 1, gamma 1	ENSMUSG00000032384	136036	191,88
Pten	phosphatase and tensin homolog	ENSMUSG00000013663	68664	191,81
Stk39	serine/threonine kinase 39	ENSMUSG00000027030	261824	191,81
Rnf144a	ring finger protein 144A	ENSMUSG00000020642	108460	191,66
Prdm2	PR domain containing 2, with ZNF domain	ENSMUSG00000057637	105605	191,66
Mef2a	myocyte enhancer factor 2A	ENSMUSG00000030557	141696	191,66
Sord	sorbitol dehydrogenase	ENSMUSG00000027227	30592	191,59
Zc2hc1a	zinc finger, C2HC-type containing 1A	ENSMUSG00000043542	50423	191,45
Mcf2l	mcf.2 transforming sequence-like	ENSMUSG00000031442	147100	191,45
Rrbp1	ribosome binding protein 1	ENSMUSG00000027422	63869	191,16
Plekha7	pleckstrin homology domain containing, family A member 7	ENSMUSG00000045659	184884	191,16
Mfsd1	major facilitator superfamily domain containing 1	ENSMUSG00000027775	21464	191,08
Tet3	tet methylcytosine dioxygenase 3	ENSMUSG00000034832	79306	191,08
Hn1	hematological and neurological expressed sequence 1	ENSMUSG00000020737	17035	191,01
Tshz3	teashirt zinc finger family member 3	ENSMUSG00000021217	75428	190,87
Ubr4	ubiquitin protein ligase E3 component n-recognin 4	ENSMUSG00000066036	136980	190,72
Mark3	MAP/microtubule affinity-regulating kinase 3	ENSMUSG00000007411	81718	190,65
Tob2	transducer of ERBB2, 2	ENSMUSG00000048546	10057	190,36

Hmgb1	high mobility group box 1	ENSMUSG00000066551	6812	190,29
Clmn	calmin	ENSMUSG00000021097	101963	190,21
		ENSMUSG00000027784	243942	190,07
1810011O10Rik	RIKEN cDNA 1810011O10 gene	ENSMUSG00000056313	1331	189,92
Usp3	ubiquitin specific peptidase 3	ENSMUSG00000032376	78506	189,63
Rapgef2	Rap guanine nucleotide exchange factor (GEF) 2	ENSMUSG00000062232	118825	189,63
Fetub	fetuin beta	ENSMUSG00000022871	21385	189,49
Galnt14	UDP-N-acetyl-alpha-D-galactosamine:polypeptide N-acetylgalactosaminyltransferase 14	ENSMUSG00000024064	217226	189,42
Pgam1	phosphoglycerate mutase 1	ENSMUSG00000011752	6794	189,34
		ENSMUSG00000020496	6629	189,27
Chd2	chromodomain helicase DNA binding protein 2	ENSMUSG00000078671	115056	189,27
Gtf2ird1	general transcription factor II I repeat domain-containing 1	ENSMUSG00000023079	99048	189,05
Ccar1	cell division cycle and apoptosis regulator 1	ENSMUSG00000020074	48441	188,98
Cpsf2	cleavage and polyadenylation specific factor 2	ENSMUSG00000041781	30020	188,91
Ninl	ninein-like	ENSMUSG00000068115	104864	188,62
		ENSMUSG00000001627	46938	188,62
		ENSMUSG00000056201	3545	188,47
Mier1	mesoderm induction early response 1 homolog (Xenopus laevis	ENSMUSG00000028522	51365	188,47
Hnrnpul2	heterogeneous nuclear ribonucleoprotein U-like 2	ENSMUSG00000071659	14742	188,40
Ndufs4	NADH dehydrogenase (ubiquinone) Fe-S protein 4	ENSMUSG00000021764	100300	188,33
Khdrbs1	KH domain containing, RNA binding, signal transduction associated 1	ENSMUSG00000028790	39140	188,11
Myo5b	myosin VB	ENSMUSG00000025885	330558	188,11
Arid2	AT rich interactive domain 2 (ARID, RFX-like)	ENSMUSG00000033237	117475	187,60
Gabarap	gamma-aminobutyric acid receptor associated protein	ENSMUSG00000018567	3809	187,46
Id2	inhibitor of DNA binding 2	ENSMUSG00000020644	2292	187,31
Map6	microtubule-associated protein 6	ENSMUSG00000055407	69691	187,17
Zfp407	zinc finger protein 407	ENSMUSG00000048410	461699	187,10
Lsr	lipolysis stimulated lipoprotein receptor	ENSMUSG00000001247	25761	186,59
Laptm4b	lysosomal-associated protein transmembrane 4B	ENSMUSG00000022257	46270	186,45
		ENSMUSG00000019806	221927	186,45
Fubp1	far upstream element (FUSE) binding protein 1	ENSMUSG00000028034	26373	186,23
Ccdc173	coiled-coil domain containing 173	ENSMUSG00000070883	31543	186,01
Actn4	actinin alpha 4	ENSMUSG00000054808	69093	186,01
Atp6v0d1	ATPase, H+ transporting, lysosomal V0 subunit D1	ENSMUSG00000013160	41583	185,94
Rragd	Ras-related GTP binding D	ENSMUSG00000028278	39144	185,94
Ckb	creatine kinase, brain	ENSMUSG00000001270	2984	185,72
Tpm3	tropomyosin 3, gamma	ENSMUSG00000027940	28254	185,50

Ric8	resistance to inhibitors of cholinesterase 8 homolog (C. elegans)	ENSMUSG00000025485	6774	185,43
Fchsd2	FCH and double SH3 domains 2	ENSMUSG00000030691	175631	185,36
		ENSMUSG00000030315	109367	185,21
Wapal	wings apart-like homolog (Drosophila)	ENSMUSG00000041408	74056	185,07
Srrm4	serine/arginine repetitive matrix 4	ENSMUSG00000063919	152546	184,85
		ENSMUSG00000022601	42639	184,71
Clcn3	chloride channel 3	ENSMUSG00000004319	72912	184,71
Thrap3	thyroid hormone receptor associated protein 3	ENSMUSG00000043962	38679	184,63
		ENSMUSG00000078126	514	184,56
Rnf149	ring finger protein 149	ENSMUSG00000048234	26110	184,49
Larp4b	La ribonucleoprotein domain family, member 4B	ENSMUSG00000033499	79182	184,49
2010111I01Rik	RIKEN cDNA 2010111I01 gene	ENSMUSG00000021458	311163	184,34
Slc5a8	solute carrier family 5 (iodide transporter), member 8	ENSMUSG00000020062	43514	184,27
Dner	delta/notch-like EGF-related receptor	ENSMUSG00000036766	326381	184,20
Srsf2	serine/arginine-rich splicing factor 2	ENSMUSG00000034120	3194	183,98
Limd1	LIM domains containing 1	ENSMUSG00000025239	42848	183,84
H1f0	H1 histone family, member 0	ENSMUSG00000096210	2287	183,76
Frmd5	FERM domain containing 5	ENSMUSG00000027238	261559	183,69
Ulk2	unc-51 like kinase 2	ENSMUSG00000004798	79425	183,55
Ptk2	PTK2 protein tyrosine kinase 2	ENSMUSG00000022607	218087	183,55
Uqcrb	ubiquinol-cytochrome c reductase binding protein	ENSMUSG00000021520	4762	183,33
Snrpe	small nuclear ribonucleoprotein E	ENSMUSG00000090553	6421	183,18
Nedd4	neural precursor cell expressed, developmentally down-regulated 4	ENSMUSG00000032216	87286	183,04
Fars2	phenylalanine-tRNA synthetase 2 (mitochondrial)	ENSMUSG00000021420	420182	183,04
Osbpl8	oxysterol binding protein-like 8	ENSMUSG00000020189	132446	182,89
Gars	glycyl-tRNA synthetase	ENSMUSG00000029777	41504	182,75
Caprin1	cell cycle associated protein 1	ENSMUSG00000027184	34709	182,53
Hook3	hook homolog 3 (Drosophila)	ENSMUSG00000037234	97804	182,53
Cldn7	claudin 7	ENSMUSG00000018569	3107	182,46
Tpr	translocated promoter region	ENSMUSG00000006005	57098	182,46
Plxna4	plexin A4	ENSMUSG00000029765	442267	182,31
Acadl	acyl-Coenzyme A dehydrogenase, long-chain	ENSMUSG00000026003	32439	181,95
Casz1	castor zinc finger 1	ENSMUSG00000028977	150461	181,95
Ldb3	LIM domain binding 3	ENSMUSG00000021798	61983	181,95
Tspan7	tetraspanin 7	ENSMUSG00000058254	111448	181,88
Odf2	outer dense fiber of sperm tails 2	ENSMUSG00000026790	42526	181,88
Gatad2a	GATA zinc finger domain containing 2A	ENSMUSG00000036180	89311	181,81
Fkbp1a	FK506 binding protein 1a	ENSMUSG00000032966	19210	181,59
Ambra1	autophagy/beclin 1 regulator 1	ENSMUSG00000040506	188716	181,59
Rabep1	rabaptin, RAB GTPase binding effector protein 1	ENSMUSG00000020817	98328	181,08
Ik	IK cytokine	ENSMUSG00000024474	12984	180,86
Tmpo	thymopoietin	ENSMUSG00000019961	24012	180,79

Cnot2	CCR4-NOT transcription complex, subunit 2	ENSMUSG00000020166	96351	180,79
Dlg1	discs, large homolog 1 (Drosophila)	ENSMUSG00000022770	209914	180,57
Rps3a1	ribosomal protein S3A1	ENSMUSG00000028081	4763	180,50
Fmn2	formin 2	ENSMUSG00000028354	320893	180,50
Dync1li2	dynein, cytoplasmic 1 light intermediate chain 2	ENSMUSG00000035770	25374	180,43
Nol7	nucleolar protein 7	ENSMUSG00000063200	4483	180,43
Rbm26	RNA binding motif protein 26	ENSMUSG00000022119	70577	180,43
Slc38a1	solute carrier family 38, member 1	ENSMUSG00000023169	71496	180,36
Faf1	Fas-associated factor 1	ENSMUSG00000010517	287373	180,21
Irf2bpl	interferon regulatory factor 2 binding protein-like	ENSMUSG00000034168	4112	179,99
Egln1	EGL nine homolog 1 (C. elegans)	ENSMUSG00000031987	40659	179,78
Mtdh	metadherin	ENSMUSG00000022255	59693	179,63
Sumo1	SMT3 suppressor of mif two 3 homolog 1 (yeast)	ENSMUSG00000026021	31401	179,56
		ENSMUSG00000027782	59965	179,56
Gldn	gliomedin	ENSMUSG00000046167	55301	179,41
Gigyf2	GRB10 interacting GYF protein 2	ENSMUSG00000048000	123799	179,34
Oxr1	oxidation resistance 1	ENSMUSG00000022307	413567	179,20
Tyro3	TYRO3 protein tyrosine kinase 3	ENSMUSG00000027298	20372	178,98
Lrrfip1	leucine rich repeat (in FLII) interacting protein 1	ENSMUSG00000026305	130218	178,62
Stmn3	stathmin-like 3	ENSMUSG00000027581	8042	178,54
Map3k5	mitogen-activated protein kinase kinase kinase 5	ENSMUSG00000071369	208282	178,47
Wdr1	WD repeat domain 1	ENSMUSG00000005103	34853	178,40
Inadl	InaD-like (Drosophila)	ENSMUSG00000061859	323819	178,40
		ENSMUSG00000030287	393925	178,33
Agr2	anterior gradient 2	ENSMUSG00000020581	11181	178,25
Cntnap5b	contactin associated protein-like 5B	ENSMUSG00000067028	713178	178,25
Nadk2	NAD kinase 2, mitochondrial	ENSMUSG00000022253	39228	178,18
Trpm4	transient receptor potential cation channel, subfamily M, member 4	ENSMUSG00000038260	30626	178,11
		ENSMUSG00000024750	10012	177,60
Puf60	poly-U binding splicing factor 60	ENSMUSG00000002524	10763	177,53
1700017B05Rik	RIKEN cDNA 1700017B05 gene	ENSMUSG00000032300	10278	177,38
Ric8b	resistance to inhibitors of cholinesterase 8 homolog B (C. elegans)	ENSMUSG00000035620	98831	177,31
Ptprj	protein tyrosine phosphatase, receptor type, J	ENSMUSG00000025314	150894	177,24
Pkp4	plakophilin 4	ENSMUSG00000026991	194359	177,17
Ptpn1	protein tyrosine phosphatase, non-receptor type 1	ENSMUSG00000027540	47329	177,09
Lyn	Yamaguchi sarcoma viral (v-yes-1) oncogene homolog	ENSMUSG00000042228	135008	176,66
Rps17	ribosomal protein S17	ENSMUSG00000061787	2523	176,59
Cdk12	cyclin-dependent kinase 12	ENSMUSG00000003119	75446	176,51
Parp8	poly (ADP-ribose) polymerase family, member 8	ENSMUSG00000021725	170693	176,37
Stxbp6	syntaxin binding protein 6 (amisyn)	ENSMUSG00000046314	222006	176,22
Zfml	zinc finger, matrin-like	ENSMUSG00000030016	72517	176,08

Brwd1	bromodomain and WD repeat domain containing 1	ENSMUSG00000022914	90435	176,08
Rd3	retinal degeneration 3	ENSMUSG00000049353	10910	175,64
Gpbp1	GC-rich promoter binding protein 1	ENSMUSG00000032745	64432	175,57
		ENSMUSG00000025917	13526	175,35
Gramd1b	GRAM domain containing 1B	ENSMUSG00000040111	238151	175,21
Sox21	SRY-box containing gene 21	ENSMUSG00000061517	3799	175,06
Gm16039	predicted gene 16039	ENSMUSG00000089862	338193	174,99
		ENSMUSG00000041440	65266	174,99
Prpf19	PRP19/PSO4 pre-mRNA processing factor 19 homolog (S. cerevisiae)	ENSMUSG00000024735	21404	174,92
8430427H17Rik	RIKEN cDNA 8430427H17 gene	ENSMUSG00000061411	122510	174,92
Atf3	activating transcription factor 3	ENSMUSG00000026628	13045	174,92
Ctnnbip1	catenin beta interacting protein 1	ENSMUSG00000028988	48202	174,77
Mettl7a1	methyltransferase like 7A1	ENSMUSG00000054619	9532	174,70
		ENSMUSG00000021219	516820	174,70
Thsd4	thrombospondin, type I, domain containing 4	ENSMUSG00000032289	555116	174,63
Rab11b	RAB11B, member RAS oncogene family	ENSMUSG00000077450	18047	174,48
Papola	poly (A) polymerase alpha	ENSMUSG00000021111	54251	174,48
Phf3	PHD finger protein 3	ENSMUSG00000048874	60915	174,48
Nfkbia	nuclear factor of kappa light polypeptide gene enhancer in B cells inhibitor, alpha	ENSMUSG00000021025	3237	174,41
Chchd3	coiled-coil-helix-coiled-coil-helix domain containing 3	ENSMUSG00000053768	269285	174,27
Zfp91	zinc finger protein 91	ENSMUSG00000024695	32467	174,19
		ENSMUSG00000026457	17974	173,98
1110051M20Rik	RIKEN cDNA 1110051M20 gene	ENSMUSG00000040591	169637	173,90
Srsf7	serine/arginine-rich splicing factor 7	ENSMUSG00000024097	7217	173,69
Ubtf	upstream binding transcription factor, RNA polymerase I	ENSMUSG00000020923	15183	173,47
Irgq	immunity-related GTPase family, Q	ENSMUSG00000041037	7952	173,47
Rps26	ribosomal protein S26	ENSMUSG00000025362	1977	173,25
Pik3c2a	phosphatidylinositol 3-kinase, C2 domain containing, alpha polypeptide	ENSMUSG00000030660	106183	173,25
Notch2	notch 2	ENSMUSG00000027878	136830	173,25
Atp5j	ATP synthase, H+ transporting, mitochondrial F0 complex, subunit F	ENSMUSG00000022890	7760	173,18
Flrt1	fibronectin leucine rich transmembrane protein 1	ENSMUSG00000047787	13716	173,18
Rnh1	ribonuclease/angiogenin inhibitor 1	ENSMUSG00000038650	12524	173,18
Gm20594	predicted gene, 20594	ENSMUSG00000096887	334	173,18
Rnf182	ring finger protein 182	ENSMUSG00000044164	55236	173,11
Gys1	glycogen synthase 1, muscle	ENSMUSG00000003865	21776	173,11
Cask	calcium/calmodulin-dependent serine protein kinase (MAGUK family)	ENSMUSG00000031012	334288	172,89
Erc1	ELKS/RAB6-interacting/CAST family member 1	ENSMUSG00000030172	277347	172,89
Asxl2	additional sex combs like 2 (Drosophila)	ENSMUSG00000037486	79996	172,82

Dscaml1	Down syndrome cell adhesion molecule like 1	ENSMUSG00000032087	322872	172,60
Hnrnpm	heterogeneous nuclear ribonucleoprotein M	ENSMUSG00000059208	40628	172,45
Nhp2l1	NHP2 non-histone chromosome protein 2-like 1 (S. cerevisiae)	ENSMUSG00000063480	6280	172,24
Alcam	activated leukocyte cell adhesion molecule	ENSMUSG00000022636	205079	172,02
		ENSMUSG00000059751	861	171,15
Zfp36	zinc finger protein 36	ENSMUSG00000044786	2472	170,93
Klc1	kinesin light chain 1	ENSMUSG00000021288	48996	170,79
Herc1	hect (homologous to the E6-AP (UBE3A) carboxyl terminus) domain and RCC1 (CHC1)-like domain (RLD) 1	ENSMUSG00000038664	158326	170,79
Nsmce2	non-SMC element 2 homolog (MMS21, S. cerevisiae)	ENSMUSG00000059586	227486	170,79
Hmgn3	high mobility group nucleosomal binding domain 3	ENSMUSG00000066456	36738	170,57
Taok1	TAO kinase 1	ENSMUSG00000017291	78654	170,57
Trim33	tripartite motif-containing 33	ENSMUSG00000033014	79476	170,28
Cdc42bpa	CDC42 binding protein kinase alpha	ENSMUSG00000026490	205132	170,21
Lrch3	leucine-rich repeats and calponin homology (CH) domain containing 3	ENSMUSG00000022801	101548	170,13
Kcnh8	potassium voltage-gated channel, subfamily H (eag-related), member 8	ENSMUSG00000035580	376271	170,06
Kras	v-Ki-ras2 Kirsten rat sarcoma viral oncogene homolog	ENSMUSG00000030265	33541	169,99
Pcp4l1	Purkinje cell protein 4-like 1	ENSMUSG00000038370	23007	169,92
Enah	enabled homolog (Drosophila)	ENSMUSG00000022995	115536	169,92
Ttll7	tubulin tyrosine ligase-like family, member 7	ENSMUSG00000036745	131643	169,84
Slc16a2	solute carrier family 16 (monocarboxylic acid transporters), member 2	ENSMUSG00000033965	124552	169,77
Nr2c2	nuclear receptor subfamily 2, group C, member 2	ENSMUSG00000005893	81668	169,77
Setd2	SET domain containing 2	ENSMUSG00000044791	86037	169,48
Mapk14	mitogen-activated protein kinase 14	ENSMUSG00000053436	57063	169,34
Ndrg1	N-myc downstream regulated gene 1	ENSMUSG00000005125	40320	169,19
C130026I21Rik	RIKEN cDNA C130026I21 gene	ENSMUSG00000052477	277877	169,12
Fam213a	family with sequence similarity 213, member A	ENSMUSG00000021792	20049	169,12
Tacc2	transforming, acidic coiled-coil containing protein 2	ENSMUSG00000030852	187301	169,12
Dnm1l	dynamin 1-like	ENSMUSG00000022789	46796	169,05
Trim24	tripartite motif-containing 24	ENSMUSG00000029833	95486	168,97
Phtf1	putative homeodomain transcription factor 1	ENSMUSG00000058388	56489	168,83
Polr2a	polymerase (RNA) II (DNA directed) polypeptide A	ENSMUSG00000005198	24641	168,76

Acsl1	acyl-CoA synthetase long-chain family member 1	ENSMUSG00000018796	65011	168,54
Calr	calreticulin	ENSMUSG00000003814	5085	168,39
Ktn1	kinectin 1	ENSMUSG00000021843	72808	168,39
Mga	MAX gene associated	ENSMUSG00000033943	72354	167,82
Shc4	SHC (Src homology 2 domain containing) family, member 4	ENSMUSG00000035109	96702	167,60
Npepps	aminopeptidase puromycin sensitive	ENSMUSG00000001441	74797	167,60
		ENSMUSG00000078515	45871	167,31
Aff1	AF4/FMR2 family, member 1	ENSMUSG00000029313	162949	167,31
Rbbp8	retinoblastoma binding protein 8	ENSMUSG00000041238	109932	167,31
Nxn	nucleoredoxin	ENSMUSG00000020844	141943	167,24
Fam195b	family with sequence similarity 195, member B	ENSMUSG00000061111	6840	167,09
Ppp1r12a	protein phosphatase 1, regulatory (inhibitor) subunit 12A	ENSMUSG00000019907	115176	166,58
Psma7	proteasome (prosome, macropain) subunit, alpha type 7	ENSMUSG00000027566	6061	166,51
Pvrl3	poliovirus receptor-related 3	ENSMUSG00000022656	110820	166,51
Dek	DEK oncogene (DNA binding)	ENSMUSG00000021377	21427	166,44
Slk	STE20-like kinase	ENSMUSG00000025060	65569	166,44
		ENSMUSG00000037822	90341	166,37
Rsf1	remodeling and spacing factor 1	ENSMUSG00000035623	112887	166,22
Lgr4	leucine-rich repeat-containing G protein-coupled receptor 4	ENSMUSG00000050199	96611	166,15
Dync1i2	dynein cytoplasmic 1 intermediate chain 2	ENSMUSG00000027012	51598	165,93
Mgat4b	mannoside acetylglucosaminyltransferase 4, isoenzyme B	ENSMUSG00000036620	24214	165,79
Smc4	structural maintenance of chromosomes 4	ENSMUSG00000034349	29886	165,71
Zfp106	zinc finger protein 106	ENSMUSG00000027288	57024	165,50
2610507B11Rik	RIKEN cDNA 2610507B11 gene	ENSMUSG00000010277	28872	165,42
Aftph	aftiphilin	ENSMUSG00000049659	56506	165,35
Hdac7	histone deacetylase 7	ENSMUSG00000022475	51839	165,35
Luzp1	leucine zipper protein 1	ENSMUSG00000001089	85020	165,28
		ENSMUSG00000048388	70851	164,92
Ubn1	ubinuclein 1	ENSMUSG00000039473	36218	164,92
Vps13b	vacuolar protein sorting 13B (yeast)	ENSMUSG00000037646	559683	164,92
Slc1a4	solute carrier family 1 (glutamate/neutral amino acid transporter), member 4	ENSMUSG00000020142	30534	164,77
Tm9sf3	transmembrane 9 superfamily member 3	ENSMUSG00000025016	53163	164,70
Atxn2	ataxin 2	ENSMUSG00000042605	105157	164,63
		ENSMUSG00000022710	102714	164,55
Sptan1	spectrin alpha, non-erythrocytic 1	ENSMUSG00000057738	65892	164,48
Cox8a	cytochrome c oxidase subunit VIIIa	ENSMUSG00000035885	2459	164,34
Slc9a9	solute carrier family 9 (sodium/hydrogen exchanger), member 9	ENSMUSG00000031129	560537	164,34
Zfp367	zinc finger protein 367	ENSMUSG00000044934	20181	164,12
Prkcd	protein kinase C, delta	ENSMUSG00000021948	30855	164,12
Stim2	stromal interaction molecule 2	ENSMUSG00000039156	122641	163,97

Cds2	CDP-diacylglycerol synthase (phosphatidate cytidylyltransferase) 2	ENSMUSG00000058793	48903	163,90
Man1a2	mannosidase, alpha, class 1A, member 2	ENSMUSG00000008763	123296	163,90
Acadm	acyl-Coenzyme A dehydrogenase, medium chain	ENSMUSG00000062908	22276	163,90
Fyttd1	forty-two-three domain containing 1	ENSMUSG00000022800	31464	163,68
Eif5b	eukaryotic translation initiation factor 5B	ENSMUSG00000026083	57570	163,39
Pde7b	phosphodiesterase 7B	ENSMUSG00000019990	327075	163,32
Msrb3	methionine sulfoxide reductase B3	ENSMUSG00000051236	118006	163,32
Cul3	cullin 3	ENSMUSG00000004364	75558	163,10
		ENSMUSG00000025584	120938	163,10
B4galnt3	beta-1,4-N-acetyl-galactosaminyl transferase 3	ENSMUSG00000041372	91487	163,03
Ncoa6	nuclear receptor coactivator 6	ENSMUSG00000038369	83239	162,89
		ENSMUSG00000040274	179326	162,89
Utp3	UTP3, small subunit (SSU) processome component, homolog (S. cerevisiae)	ENSMUSG00000070697	1601	162,67
Kansl3	KAT8 regulatory NSL complex subunit 3	ENSMUSG00000010453	33452	162,67
		ENSMUSG00000079139	1360	162,60
Klhl7	kelch-like 7	ENSMUSG00000028986	60634	162,52
		ENSMUSG00000089945	328804	162,52
		ENSMUSG00000036792	372775	162,52
Rdx	radixin	ENSMUSG00000032050	41588	162,38
		ENSMUSG00000027210	202776	162,38
Dmrta2	doublesex and mab-3 related transcription factor like family A2	ENSMUSG00000047143	5635	162,16
Rps25	ribosomal protein S25	ENSMUSG00000009927	2692	161,87
Cct7	chaperonin containing Tcp1, subunit 7 (eta)	ENSMUSG00000030007	16973	161,87
Aamp	angio-associated migratory protein	ENSMUSG00000006299	4899	161,80
Osbpl9	oxysterol binding protein-like 9	ENSMUSG00000028559	141128	161,73
Vti1a	vesicle transport through interaction with t-SNAREs 1A	ENSMUSG00000024983	310211	161,51
Slc25a35	solute carrier family 25, member 35	ENSMUSG00000018740	6235	161,44
1110032A03Rik	RIKEN cDNA 1110032A03 gene	ENSMUSG00000037971	12693	161,00
Zfp609	zinc finger protein 609	ENSMUSG00000040524	135174	161,00
Nsf	N-ethylmaleimide sensitive fusion protein	ENSMUSG00000034187	132275	160,93
Map4k4	mitogen-activated protein kinase kinase kinase kinase 4	ENSMUSG00000026074	125398	160,86
Vim	vimentin	ENSMUSG00000026728	8900	160,64
Clasp1	CLIP associating protein 1	ENSMUSG00000064302	220375	160,64
Dock8	dedicator of cytokinesis 8	ENSMUSG00000052085	202904	160,49
Blnk	B cell linker	ENSMUSG00000061132	65609	160,42
Phf14	PHD finger protein 14	ENSMUSG00000029629	173389	160,20
Tspan3	tetraspanin 3	ENSMUSG00000032324	25186	160,13
Trim30a	tripartite motif-containing 30A	ENSMUSG00000030921	56169	160,06
Klf13	Kruppel-like factor 13	ENSMUSG00000052040	52565	160,06
Anxa1	annexin A1	ENSMUSG00000024659	17517	160,06
Farsb	phenylalanyl-tRNA synthetase, beta subunit	ENSMUSG00000026245	70902	159,99

Ryr3	ryanodine receptor 3	ENSMUSG00000057378	585742	159,99
Dync2h1	dynein cytoplasmic 2 heavy chain 1	ENSMUSG00000047193	255944	159,91
Hnrnpl	heterogeneous nuclear ribonucleoprotein L	ENSMUSG00000015165	13726	159,84
Pcm1	pericentriolar material 1	ENSMUSG00000031592	94328	159,70
Tab2	TGF-beta activated kinase 1/MAP3K7 binding protein 2	ENSMUSG00000015755	50578	159,62
Sumo2	SMT3 suppressor of mif two 3 homolog 2 (yeast)	ENSMUSG00000020738	13175	159,55
Mapk8	mitogen-activated protein kinase 8	ENSMUSG00000021936	69261	159,33
Arhgef11	Rho guanine nucleotide exchange factor (GEF) 11	ENSMUSG00000041977	120476	159,33
		ENSMUSG00000014956	58460	159,19
Arrb2	arrestin, beta 2	ENSMUSG00000060216	8194	159,12
Atp8b4	ATPase, class I, type 8B, member 4	ENSMUSG00000060131	179702	158,97
AI314180	expressed sequence AI314180	ENSMUSG00000050812	113839	158,97
Fut8	fucosyltransferase 8	ENSMUSG00000021065	238235	158,90
		ENSMUSG00000037386	483609	158,83
Ppp1r15b	protein phosphatase 1, regulatory (inhibitor) subunit 15b	ENSMUSG00000046062	8641	158,75
Fnbp1l	formin binding protein 1-like	ENSMUSG00000039735	80949	158,75
Sipa1l3	signal-induced proliferation-associated 1 like 3	ENSMUSG00000030583	198270	158,61
Rab6b	RAB6B, member RAS oncogene family	ENSMUSG00000032549	73197	158,54
Gsn	gelsolin	ENSMUSG00000026879	28230	158,54
Tmed10	transmembrane emp24-like trafficking protein 10 (yeast)	ENSMUSG00000021248	34104	158,54
Csnk1d	casein kinase 1, delta	ENSMUSG00000025162	29582	158,46
Atf2	activating transcription factor 2	ENSMUSG00000027104	76131	158,46
Nptn	neuroplastin	ENSMUSG00000032336	75716	158,39
Ap2m1	adaptor-related protein complex 2, mu 1 subunit	ENSMUSG00000022841	9432	158,32
Fam63b	family with sequence similarity 63, member B	ENSMUSG00000042444	58161	158,32
Tpd52	tumor protein D52	ENSMUSG00000027506	76098	158,17
Hist2h2aa2	histone cluster 2, H2aa2	ENSMUSG00000064220	539	158,10
		ENSMUSG00000025757	51609	158,10
2410089E03Rik	RIKEN cDNA 2410089E03 gene	ENSMUSG00000039801	102053	158,10
Atp6v0a4	ATPase, H+ transporting, lysosomal V0 subunit A4	ENSMUSG00000038600	76104	158,10
Klhl32	kelch-like 32	ENSMUSG00000040387	238571	158,03
Ptpra	protein tyrosine phosphatase, receptor type, A	ENSMUSG00000027303	103749	157,96
Acss2	acyl-CoA synthetase short-chain family member 2	ENSMUSG00000027605	67777	157,74
BC005561	cDNA sequence BC005561	ENSMUSG00000079065	14032	157,67
		ENSMUSG00000041272	305205	157,52
Nras	neuroblastoma ras oncogene	ENSMUSG00000027852	9576	157,45
Tnks2	tankyrase, TRF1-interacting ankyrin-related ADP-ribose polymerase 2	ENSMUSG00000024811	59246	157,45
Prkacb	protein kinase, cAMP dependent, catalytic, beta	ENSMUSG00000005034	83382	157,23

Eml4	echinoderm microtubule associated protein like 4	ENSMUSG00000032624	129431	157,23
Rcc2	regulator of chromosome condensation 2	ENSMUSG00000040945	22680	157,16
Psmd8	proteasome (prosome, macropain) 26S subunit, non-ATPase, 8	ENSMUSG00000030591	6514	156,94
Rc3h1	RING CCCH (C3H) domains 1	ENSMUSG00000040423	68561	156,94
Eid1	EP300 interacting inhibitor of differentiation 1	ENSMUSG00000091337	2539	156,43
Pde4b	phosphodiesterase 4B, cAMP specific	ENSMUSG00000028525	519717	156,43
Cbx1	chromobox 1	ENSMUSG00000018666	19514	156,22
Gm5428	predicted gene 5428	ENSMUSG00000091086	888	155,85
Myo1e	myosin IE	ENSMUSG00000032220	192718	155,78
Nhlh1	nescient helix loop helix 1	ENSMUSG00000051251	5282	155,71
Rfc1	replication factor C (activator 1) 1	ENSMUSG00000029191	73819	155,71
Mxd4	Max dimerization protein 4	ENSMUSG00000037235	13834	155,71
Rmnd5a	required for meiotic nuclear division 5 homolog A (S. cerevisiae)	ENSMUSG00000002222	52004	155,71
D14Abb1e	DNA segment, Chr 14, Abbott 1 expressed	ENSMUSG00000040651	54706	155,64
Abcf1	ATP-binding cassette, sub-family F (GCN20), member 1	ENSMUSG00000038762	12943	155,56
Akap17b	A kinase (PRKA) anchor protein 17B	ENSMUSG00000059708	37082	155,56
Ddx17	DEAD (Asp-Glu-Ala-Asp) box polypeptide 17	ENSMUSG00000055065	19035	155,56
Hecw2	HECT, C2 and WW domain containing E3 ubiquitin protein ligase 2	ENSMUSG00000042807	388287	155,56
Eid2	EP300 interacting inhibitor of differentiation 2	ENSMUSG00000046058	1285	155,49
Morf4l2	mortality factor 4 like 2	ENSMUSG00000031422	10749	155,42
Ggnbp2	gametogenetin binding protein 2	ENSMUSG00000020530	38457	155,42
Zbtb38	zinc finger and BTB domain containing 38	ENSMUSG00000040433	70069	155,27
Baz2a	bromodomain adjacent to zinc finger domain, 2A	ENSMUSG00000040054	36521	155,27
Serp1	stress-associated endoplasmic reticulum protein 1	ENSMUSG00000027808	3905	155,20
Pi4k2a	phosphatidylinositol 4-kinase type 2 alpha	ENSMUSG00000025178	31784	155,13
Med26	mediator complex subunit 26	ENSMUSG00000045248	53753	155,13
Dot1l	DOT1-like, histone H3 methyltransferase (S. cerevisiae)	ENSMUSG00000061589	40256	155,06
Rlf	rearranged L-myc fusion sequence	ENSMUSG00000049878	69712	155,06
N4bp2l2	NEDD4 binding protein 2-like 2	ENSMUSG00000029655	57606	155,06
Atp5f1	ATP synthase, H+ transporting, mitochondrial F0 complex, subunit B1	ENSMUSG00000000563	17402	154,98
Ccdc88c	coiled-coil domain containing 88C	ENSMUSG00000021182	116284	154,98
Slc9a3r1	solute carrier family 9 (sodium/hydrogen exchanger), member 3 regulator 1	ENSMUSG00000020733	17841	154,84
		ENSMUSG00000020873	7085	154,77
		ENSMUSG00000022403	36409	154,69

Sema3a	sema domain, immunoglobulin domain (Ig), short basic domain, secreted, (semaphorin) 3A	ENSMUSG00000028883	205782	154,69
Cldn3	claudin 3	ENSMUSG00000070473	1263	154,62
		ENSMUSG00000028797	23392	154,62
Xpo7	exportin 7	ENSMUSG00000022100	112383	154,62
Zfhx4	zinc finger homeodomain 4	ENSMUSG00000025255	197330	154,62
		ENSMUSG00000029616	23917	154,40
Fdps	farnesyl diphosphate synthetase	ENSMUSG00000059743	8376	154,40
Ppp3cb	protein phosphatase 3, catalytic subunit, beta isoform	ENSMUSG00000021816	47210	154,33
Kcnh6	potassium voltage-gated channel, subfamily H (eag-related), member 6	ENSMUSG00000001901	26426	154,33
Lin7c	lin-7 homolog C (C. elegans)	ENSMUSG00000027162	10151	154,26
Clcn4-2	chloride channel 4-2	ENSMUSG00000000605	17196	154,11
D17Wsu92e	DNA segment, Chr 17, Wayne State University 92, expressed	ENSMUSG00000056692	69327	154,04
Atp6v1c2	ATPase, H+ transporting, lysosomal V1 subunit C2	ENSMUSG00000020566	44639	154,04
Ube2g1	ubiquitin-conjugating enzyme E2G 1	ENSMUSG00000020794	79199	153,82
Ube2b	ubiquitin-conjugating enzyme E2B	ENSMUSG00000020390	15266	153,61
Dab2ip	disabled 2 interacting protein	ENSMUSG00000026883	172729	153,53
Ubr3	ubiquitin protein ligase E3 component n-recognin 3	ENSMUSG00000044308	126768	153,46
Magt1	magnesium transporter 1	ENSMUSG00000031232	43823	153,39
Msi1	musashi RNA-binding protein 1	ENSMUSG00000054256	26100	153,32
Elavl1	ELAV (embryonic lethal, abnormal vision)-like 1 (Hu antigen R)	ENSMUSG00000040028	40319	153,32
Kcnk1	potassium channel, subfamily K, member 1	ENSMUSG00000033998	35582	153,24
Rrm2b	ribonucleotide reductase M2 B (TP53 inducible)	ENSMUSG00000022292	37367	153,17
Tmem30a	transmembrane protein 30A	ENSMUSG00000032328	24565	153,03
Btrc	beta-transducin repeat containing protein	ENSMUSG00000025217	169608	152,81
Large	like-glycosyltransferase	ENSMUSG00000004383	538942	152,81
Rnf24	ring finger protein 24	ENSMUSG00000048911	54829	152,52
Hnrnph1	heterogeneous nuclear ribonucleoprotein H1	ENSMUSG00000007850	9539	152,45
		ENSMUSG00000024646	29064	152,23
Tgfbr1	transforming growth factor, beta receptor I	ENSMUSG00000007613	61710	152,16
Crebrf	CREB3 regulatory factor	ENSMUSG00000048249	60986	152,01
		ENSMUSG00000026977	40790	151,94
Poldip3	polymerase (DNA-directed), delta interacting protein 3	ENSMUSG00000041815	23407	151,87
Cox4i1	cytochrome c oxidase subunit IV isoform 1	ENSMUSG00000031818	5986	151,79
		ENSMUSG00000000827	20802	151,79
BC031181	cDNA sequence BC031181	ENSMUSG00000036299	4034	151,72
Chpt1	choline phosphotransferase 1	ENSMUSG00000060002	51329	151,72
Adh5	alcohol dehydrogenase 5 (class III), chi polypeptide	ENSMUSG00000028138	12407	151,50
Znrf1	zinc and ring finger 1	ENSMUSG00000033545	89934	151,50

Ctnnd1	catenin (cadherin associated protein), delta 1	ENSMUSG00000034101	50695	151,50
Ndrg3	N-myc downstream regulated gene 3	ENSMUSG00000027634	64712	151,43
Abcd3	ATP-binding cassette, sub-family D (ALD), member 3	ENSMUSG00000028127	56394	151,36
Tapbp	TAP binding protein	ENSMUSG00000024308	13390	151,21
Fnip1	folliculin interacting protein 1	ENSMUSG00000035992	80037	151,00
BC049352	cDNA sequence BC049352	ENSMUSG00000091996	68014	150,78
Fau	Finkel-Biskis-Reilly murine sarcoma virus (FBR-MuSV) ubiquitously expressed (fox derived)	ENSMUSG00000038274	1637	150,78
Arpc5l	actin related protein 2/3 complex, subunit 5-like	ENSMUSG00000026755	7802	150,71
Ctr9	Ctr9, Paf1/RNA polymerase II complex component, homolog (S. cerevisiae)	ENSMUSG00000005609	27427	150,71
Reps2	RALBP1 associated Eps domain containing protein 2	ENSMUSG00000040855	231696	150,71
Surf4	surfeit gene 4	ENSMUSG00000014867	13889	150,71
Kif5c	kinesin family member 5C	ENSMUSG00000026764	155481	150,63
Ube3c	ubiquitin protein ligase E3C	ENSMUSG00000039000	106836	150,63
Pacs2	phosphofurin acidic cluster sorting protein 2	ENSMUSG00000021143	59894	150,56
B4galt1	UDP-Gal:betaGlcNAc beta 1,4-galactosyltransferase, polypeptide 1	ENSMUSG00000028413	49404	150,56
Gnas	GNAS (guanine nucleotide binding protein, alpha stimulating) complex locus	ENSMUSG00000027523	62425	150,49
Rnf20	ring finger protein 20	ENSMUSG00000028309	24882	150,20
Fam3b	family with sequence similarity 3, member B	ENSMUSG00000022938	33887	150,20
Ssr1	signal sequence receptor, alpha	ENSMUSG00000021427	22790	150,05
Hnrnpab	heterogeneous nuclear ribonucleoprotein A/B	ENSMUSG00000020358	6748	149,91
Hnrnpa1	heterogeneous nuclear ribonucleoprotein A1	ENSMUSG00000046434	4524	149,91
Anks1b	ankyrin repeat and sterile alpha motif domain containing 1B	ENSMUSG00000058589	1099792	149,91
		ENSMUSG00000034868	16868	149,84
Prss12	protease, serine, 12 neurotrypsin (motopsin)	ENSMUSG00000027978	59690	149,76
Cct8	chaperonin containing Tcp1, subunit 8 (theta)	ENSMUSG00000025613	12548	149,69
Ube3b	ubiquitin protein ligase E3B	ENSMUSG00000029577	40541	149,40
Neurl1a	neuralized homolog 1A (Drosophila)	ENSMUSG00000006435	80621	149,40
Hnrnpf	heterogeneous nuclear ribonucleoprotein F	ENSMUSG00000042079	25299	149,33
Fam172a	family with sequence similarity 172, member A	ENSMUSG00000064138	457549	149,33
Hk1	hexokinase 1	ENSMUSG00000037012	111054	149,26
H2afj	H2A histone family, member J	ENSMUSG00000060032	1827	149,19
1700007G11Rik	RIKEN cDNA 1700007G11 gene	ENSMUSG00000057816	472557	149,19

Gpsm1		G-protein signalling modulator 1 (AGS3-like, C. elegans)	ENSMUSG00000026930	32723	148,97
Nf1		neurofibromatosis 1	ENSMUSG00000020716	241920	148,82
Gpd2		glycerol phosphate dehydrogenase 2, mitochondrial	ENSMUSG00000026827	133085	148,75
Sfswap		splicing factor, suppressor of white-apricot homolog (Drosophila)	ENSMUSG00000029439	70153	148,68
Prpf4b		PRP4 pre-mRNA processing factor 4 homolog B (yeast)	ENSMUSG00000021413	27384	148,68
Mcc		mutated in colorectal cancers	ENSMUSG00000071856	387122	148,68
Herc3		hect domain and RLD 3	ENSMUSG00000029804	88934	148,46
			ENSMUSG00000070985	26808	148,46
Acox1		acyl-Coenzyme A oxidase 1, palmitoyl	ENSMUSG00000020777	27158	148,39
Slc31a1		solute carrier family 31, member 1	ENSMUSG00000066150	31043	148,32
Zc3h11a		zinc finger CCCH type containing 11A	ENSMUSG00000026464	41510	148,32
			ENSMUSG00000039470	86454	148,32
Gm9790		predicted gene 9790	ENSMUSG00000044330	442	148,17
Wac		WW domain containing adaptor with coiled-coil	ENSMUSG00000024283	104716	148,17
	Sep 03	septin 3	ENSMUSG00000022456	19508	148,10
H13		histocompatibility 13	ENSMUSG00000019188	39210	148,10
Plekhg1		pleckstrin homology domain containing, family G (with RhoGef domain) member 1	ENSMUSG00000040624	226940	148,10
Slc25a25		solute carrier family 25 (mitochondrial carrier, phosphate carrier), member 25	ENSMUSG00000026819	36959	147,95
Socs7		suppressor of cytokine signaling 7	ENSMUSG00000038485	35992	147,88
			ENSMUSG00000094410	41379	147,66
			ENSMUSG00000046201	83884	147,59
Specc1l		sperm antigen with calponin homology and coiled-coil domains 1-like	ENSMUSG00000033444	100327	147,52
Elf5		E74-like factor 5	ENSMUSG00000027186	39302	147,52
Trnp1		TMF1-regulated nuclear protein 1	ENSMUSG00000056596	7451	147,30
Fbxw11		F-box and WD-40 domain protein 11	ENSMUSG00000020271	104093	147,30
Smc1a		structural maintenance of chromosomes 1A	ENSMUSG00000041133	46267	147,23
Med8		mediator of RNA polymerase II transcription, subunit 8 homolog (yeast)	ENSMUSG00000006392	6446	147,08
Tnfaip2		tumor necrosis factor, alpha-induced protein 2	ENSMUSG00000021281	12550	147,01
			ENSMUSG00000029245	359592	146,94
Syn3		synapsin III	ENSMUSG00000059602	443772	146,87
Elovl6		ELOVL family member 6, elongation of long chain fatty acids (yeast)	ENSMUSG00000041220	106110	146,87
Fryl		furry homolog-like (Drosophila)	ENSMUSG00000070733	236297	146,87
			ENSMUSG00000022619	10556	146,43
Litaf		LPS-induced TN factor	ENSMUSG00000022500	106883	146,29
Tmed4		transmembrane emp24 protein transport domain containing 4	ENSMUSG00000004394	4502	146,21

Soga3	SOGA family member 3	ENSMUSG00000038916	55635	146,14
Tcf20	transcription factor 20	ENSMUSG00000041852	103506	146,07
2210408I21Rik	RIKEN cDNA 2210408I21 gene	ENSMUSG00000071252	477752	146,07
Phf20l1	PHD finger protein 20-like 1	ENSMUSG00000072501	67684	145,92
		ENSMUSG00000025609	118077	145,92
Map2k2	mitogen-activated protein kinase kinase 2	ENSMUSG00000035027	18783	145,71
Gstm2	glutathione S-transferase, mu 2	ENSMUSG00000040562	4752	145,71
Rock2	Rho-associated coiled-coil containing protein kinase 2	ENSMUSG00000020580	93297	145,71
Snd1	staphylococcal nuclease and tudor domain containing 1	ENSMUSG00000001424	460024	145,63
Eml5	echinoderm microtubule associated protein like 5	ENSMUSG00000051166	114881	145,63
Btbd1	BTB (POZ) domain containing 1	ENSMUSG00000025103	37357	145,42
Ap2a2	adaptor-related protein complex 2, alpha 2 subunit	ENSMUSG00000002957	70832	145,34
Hmgcr	3-hydroxy-3-methylglutaryl-Coenzyme A reductase	ENSMUSG00000021670	21970	145,13
Ntn1	netrin 1	ENSMUSG00000020902	191460	144,98
		ENSMUSG00000094089	801	144,76
Stxbp1	syntaxin binding protein 1	ENSMUSG00000026797	59638	144,69
Mtpn	myotrophin	ENSMUSG00000029840	30983	144,69
Hsd17b12	hydroxysteroid (17-beta) dehydrogenase 12	ENSMUSG00000027195	125276	144,69
Fam134b	family with sequence similarity 134, member B	ENSMUSG00000022270	130424	144,69
		ENSMUSG00000028124	24428	144,55
Ube3a	ubiquitin protein ligase E3A	ENSMUSG00000025326	77976	144,55
Rnf11	ring finger protein 11	ENSMUSG00000028557	33631	144,40
Tagln2	transgelin 2	ENSMUSG00000026547	7334	144,26
Csnk1g2	casein kinase 1, gamma 2	ENSMUSG00000003345	17992	144,18
Cerk	ceramide kinase	ENSMUSG00000035891	47014	144,11
Rptor	regulatory associated protein of MTOR, complex 1	ENSMUSG00000025583	296672	144,04
Cnga2	cyclic nucleotide gated channel alpha 2	ENSMUSG00000005864	18370	143,97
U2af1	U2 small nuclear ribonucleoprotein auxiliary factor (U2AF) 1	ENSMUSG00000061613	11673	143,89
Smurf2	SMAD specific E3 ubiquitin protein ligase 2	ENSMUSG00000018363	100650	143,75
Sp3	trans-acting transcription factor 3	ENSMUSG00000027109	44020	143,68
		ENSMUSG00000028173	96097	143,68
Nhlh2	nescient helix loop helix 2	ENSMUSG00000048540	5348	143,60
Itm2b	integral membrane protein 2B	ENSMUSG00000022108	23041	143,60
Psmd2	proteasome (prosome, macropain) 26S subunit, non-ATPase, 2	ENSMUSG00000006998	11763	143,46
Pim3	proviral integration site 3	ENSMUSG00000035828	3533	143,46
Cnpy1	canopy 1 homolog (zebrafish)	ENSMUSG00000044681	44974	143,46
Nrf1	nuclear respiratory factor 1	ENSMUSG00000058440	105471	143,17
Cand1	cullin associated and neddylation disassociated 1	ENSMUSG00000020114	40801	143,10
F11r	F11 receptor	ENSMUSG00000038235	27069	142,95
Atp5j2	ATP synthase, H+ transporting, mitochondrial F0 complex, subunit F2	ENSMUSG00000038690	8365	142,88

Gpc6	glypican 6	ENSMUSG00000058571	1054233	142,52
		ENSMUSG00000018583	35154	142,44
Ahi1	Abelson helper integration site 1	ENSMUSG00000019986	127882	142,30
Acsl3	acyl-CoA synthetase long-chain family member 3	ENSMUSG00000032883	49163	142,23
Atp6ap2	ATPase, H+ transporting, lysosomal accessory protein 2	ENSMUSG00000031007	29249	142,08
Eml1	echinoderm microtubule associated protein like 1	ENSMUSG00000058070	168661	142,01
Alad	aminolevulinate, delta-, dehydratase	ENSMUSG00000028393	10750	142,01
Sdhb	succinate dehydrogenase complex, subunit B, iron sulfur (Ip)	ENSMUSG00000009863	17991	141,94
Cers4	ceramide synthase 4	ENSMUSG00000008206	32732	141,94
Diap2	diaphanous homolog 2 (Drosophila)	ENSMUSG00000034480	716093	141,79
Mb21d2	Mab-21 domain containing 2	ENSMUSG00000051065	103523	141,72
Rpl3	ribosomal protein L3	ENSMUSG00000060036	5626	141,65
Chd8	chromodomain helicase DNA binding protein 8	ENSMUSG00000053754	39641	141,65
Smarce1	SWI/SNF related, matrix associated, actin dependent regulator of chromatin, subfamily e, member 1	ENSMUSG00000037935	21971	141,57
		ENSMUSG00000030335	3783	141,50
Imp3	IMP3, U3 small nucleolar ribonucleoprotein, homolog (yeast)	ENSMUSG00000032288	898	141,50
Eif3l	eukaryotic translation initiation factor 3, subunit L	ENSMUSG00000033047	19178	141,50
		ENSMUSG00000066324	30872	141,43
Chka	choline kinase alpha	ENSMUSG00000024843	42597	141,21
Apbb2	amyloid beta (A4) precursor protein-binding, family B, member 2	ENSMUSG00000029207	319968	141,14
Aff2	AF4/FMR2 family, member 2	ENSMUSG00000031189	507744	141,07
Sep 11	septin 11	ENSMUSG00000058013	81502	141,07
Rsrc2	arginine/serine-rich coiled-coil 2	ENSMUSG00000029422	20989	140,92
Prkd1	protein kinase D1	ENSMUSG00000002688	307993	140,70
Tgoln1	trans-golgi network protein	ENSMUSG00000056429	8569	140,63
Osbp	oxysterol binding protein	ENSMUSG00000024687	28262	140,56
Copa	coatomer protein complex subunit alpha	ENSMUSG00000026553	39802	140,49
Trpm7	transient receptor potential cation channel, subfamily M, member 7	ENSMUSG00000027365	84666	140,49
Dazap1	DAZ associated protein 1	ENSMUSG00000069565	26923	140,34
Naa35	N(alpha)-acetyltransferase 35, NatC auxiliary subunit	ENSMUSG00000021555	49540	140,20
Rbm5	RNA binding motif protein 5	ENSMUSG00000032580	30626	139,98
Ints6	integrator complex subunit 6	ENSMUSG00000035161	84783	139,98
Pebp1	phosphatidylethanolamine binding protein 1	ENSMUSG00000032959	4975	139,83
Mki67	antigen identified by monoclonal antibody Ki 67	ENSMUSG00000031004	26592	139,83
Ythdc1	YTH domain containing 1	ENSMUSG00000035851	32439	139,83
Ptbp2	polypyrimidine tract binding protein 2	ENSMUSG00000028134	64647	139,83
Rpl35a	ribosomal protein L35A	ENSMUSG00000060636	3737	139,69

Lrp2	low density lipoprotein receptor-related protein 2	ENSMUSG00000027070	161726	139,69
Suz12	suppressor of zeste 12 homolog (Drosophila)	ENSMUSG00000017548	41018	139,47
Gm9846	predicted gene 9846	ENSMUSG00000050621	341	139,40
Wdr33	WD repeat domain 33	ENSMUSG00000024400	104931	139,40
Dpf3	D4, zinc and double PHD fingers, family 3	ENSMUSG00000021221	273972	139,33
Cirbp	cold inducible RNA binding protein	ENSMUSG00000045193	6802	139,25
Smim4	small itegral membrane protein 4	ENSMUSG00000058351	40062	139,18
Rad21	RAD21 homolog (S. pombe)	ENSMUSG00000022314	29156	139,18
Cep350	centrosomal protein 350	ENSMUSG00000033671	127925	139,18
Tcf25	transcription factor 25 (basic helix-loop-helix)	ENSMUSG00000001472	30350	139,11
Rassf2	Ras association (RalGDS/AF-6) domain family member 2	ENSMUSG00000027339	40844	138,96
Gpr126	G protein-coupled receptor 126	ENSMUSG00000039116	142452	138,60
Clip1	CAP-GLY domain containing linker protein 1	ENSMUSG00000049550	106824	138,53
Fyn	Fyn proto-oncogene	ENSMUSG00000019843	196527	138,46
Agrn	agrin	ENSMUSG00000041936	32199	138,31
Lims1	LIM and senescent cell antigen-like domains 1	ENSMUSG00000019920	101226	138,31
Txnip	thioredoxin interacting protein	ENSMUSG00000038393	3927	138,17
Aebp2	AE binding protein 2	ENSMUSG00000030232	56024	138,17
Evl	Ena-vasodilator stimulated phosphoprotein	ENSMUSG00000021262	133794	138,17
Arhgef12	Rho guanine nucleotide exchange factor (GEF) 12	ENSMUSG00000059495	141877	137,95
Lrrc16a	leucine rich repeat containing 16A	ENSMUSG00000021338	268452	137,95
		ENSMUSG00000089865	57468	137,73
Hectd1	HECT domain containing 1	ENSMUSG00000035247	85815	137,73
Gjc1	gap junction protein, gamma 1	ENSMUSG00000034520	20122	137,73
Rsbn1l	round spermatid basic protein 1-like	ENSMUSG00000039968	58795	137,66
Ppp2r5c	protein phosphatase 2, regulatory subunit B (B56), gamma isoform	ENSMUSG00000017843	97323	137,59
Psmd4	proteasome (prosome, macropain) 26S subunit, non-ATPase, 4	ENSMUSG00000005625	9921	137,51
Mapk1ip1l	mitogen-activated protein kinase 1 interacting protein 1-like	ENSMUSG00000021840	24891	137,51
Hmga2	high mobility group AT-hook 2	ENSMUSG00000056758	115195	137,51
Cep120	centrosomal protein 120	ENSMUSG00000048799	62824	137,30
Fam222b	family with sequence similarity 222, member B	ENSMUSG00000037750	62041	137,22
Lasp1	LIM and SH3 protein 1	ENSMUSG00000038366	39765	137,22
Atp9a	ATPase, class II, type 9A	ENSMUSG00000027546	107972	137,01
Clk1	CDC-like kinase 1	ENSMUSG00000026034	13878	136,93
Papd5	PAP associated domain containing 5	ENSMUSG00000036779	60510	136,86
		ENSMUSG00000058076	23439	136,79
Herpud1	homocysteine-inducible, endoplasmic reticulum stress-inducible, ubiquitin-like domain member 1	ENSMUSG00000031770	8940	136,79
		ENSMUSG00000051355	147639	136,79

		ENSMUSG00000094439	59506	136,72
Manf	mesencephalic astrocyte-derived neurotrophic factor	ENSMUSG00000032575	53668	136,35
Cnot4	CCR4-NOT transcription complex, subunit 4	ENSMUSG00000038784	111672	136,35
Lsm14b	LSM14 homolog B (SCD6, S. cerevisiae)	ENSMUSG00000039108	10479	136,28
Ppm1b	protein phosphatase 1B, magnesium dependent, beta isoform	ENSMUSG00000061130	67251	136,28
Mib1	mindbomb homolog 1 (Drosophila)	ENSMUSG00000024294	93157	136,28
Btg1	B cell translocation gene 1, anti-proliferative	ENSMUSG00000036478	5813	136,21
Kat6a	K(lysine) acetyltransferase 6A	ENSMUSG00000031540	83725	136,21
Tex2	testis expressed gene 2	ENSMUSG00000040548	111277	136,14
4930452B06Rik	RIKEN cDNA 4930452B06 gene	ENSMUSG00000021747	235049	135,99
Arl15	ADP-ribosylation factor-like 15	ENSMUSG00000042348	362957	135,99
Trio	triple functional domain (PTPRF interacting)	ENSMUSG00000022263	295197	135,92
Pgd	phosphogluconate dehydrogenase	ENSMUSG00000028961	16781	135,63
Bdp1	B double prime 1, subunit of RNA polymerase III transcription initiation factor IIIB	ENSMUSG00000049658	86077	135,63
Cd302	CD302 antigen	ENSMUSG00000060703	32496	135,56
Slc15a2	solute carrier family 15 (H+/peptide transporter), member 2	ENSMUSG00000022899	34785	135,41
Cpsf6	cleavage and polyadenylation specific factor 6	ENSMUSG00000055531	35343	135,27
Srcin1	SRC kinase signaling inhibitor 1	ENSMUSG00000038453	65887	135,12
Lypd2	Ly6/Plaur domain containing 2	ENSMUSG00000022595	2061	135,12
Sms	spermine synthase	ENSMUSG00000071708	48433	135,05
Reep3	receptor accessory protein 3	ENSMUSG00000019873	87800	135,05
Nrip1	nuclear receptor interacting protein 1	ENSMUSG00000048490	86428	134,98
Acp1	acid phosphatase 1, soluble	ENSMUSG00000044573	18287	134,90
Nudt3	nudix (nucleotide diphosphate linked moiety X)-type motif 3	ENSMUSG00000024213	44084	134,83
Ppp6r3	protein phosphatase 6, regulatory subunit 3	ENSMUSG00000024908	120817	134,83
E030002O03Rik	RIKEN cDNA E030002O03 gene	ENSMUSG00000044265	11819	134,83
Sdc3	syndecan 3	ENSMUSG00000025743	33783	134,76
Wiz	widely-interspaced zinc finger motifs	ENSMUSG00000024050	35347	134,76
		ENSMUSG00000091747	4223	134,76
AU040320	expressed sequence AU040320	ENSMUSG00000028830	116527	134,76
Mapk4	mitogen-activated protein kinase 4	ENSMUSG00000024558	136874	134,76
		ENSMUSG00000034755	620361	134,61
Rbpms	RNA binding protein gene with multiple splicing	ENSMUSG00000031586	147221	134,61
Pcnx	pecanex homolog (Drosophila)	ENSMUSG00000021140	140895	134,47
Hnrnpdl	heterogeneous nuclear ribonucleoprotein D-like	ENSMUSG00000029328	6088	134,40
		ENSMUSG00000032667	34309	134,40
		ENSMUSG00000041670	519744	134,40

Smarcc2	SWI/SNF related, matrix associated, actin dependent regulator of chromatin, subfamily c, member 2	ENSMUSG00000025369	30938	134,32
Apba1	amyloid beta (A4) precursor protein binding, family A, member 1	ENSMUSG00000024897	190722	134,25
Kif1a	kinesin family member 1A	ENSMUSG00000014602	86410	134,18
Nek7	NIMA (never in mitosis gene a)-related expressed kinase 7	ENSMUSG00000026393	134983	134,18
Dpp3	dipeptidylpeptidase 3	ENSMUSG00000063904	21059	134,11
Wbp2	WW domain binding protein 2	ENSMUSG00000034341	8423	134,03
Sae1	SUMO1 activating enzyme subunit 1	ENSMUSG00000052833	60745	133,96
Grhl2	grainyhead-like 2 (Drosophila)	ENSMUSG00000022286	130534	133,96
Npas2	neuronal PAS domain protein 2	ENSMUSG00000026077	169504	133,96
Rell1	RELT-like 1	ENSMUSG00000047881	60010	133,82
Gtf2b	general transcription factor IIB	ENSMUSG00000028271	18378	133,82
Dcaf6	DDB1 and CUL4 associated factor 6	ENSMUSG00000026571	130955	133,60
Lyrm4	LYR motif containing 4	ENSMUSG00000046573	138561	133,45
Rbm27	RNA binding motif protein 27	ENSMUSG00000024491	66190	133,45
		ENSMUSG00000094248	480	133,31
Timm8b	translocase of inner mitochondrial membrane 8B	ENSMUSG00000039016	1420	133,31
Smg7	Smg-7 homolog, nonsense mediated mRNA decay factor (C. elegans)	ENSMUSG00000042772	65651	133,31
Hrk	harakiri, BCL2 interacting protein (contains only BH3 domain)	ENSMUSG00000046607	19715	133,24
Zfp318	zinc finger protein 318	ENSMUSG00000015597	37190	133,24
Pkp2	plakophilin 2	ENSMUSG00000041957	59395	133,24
Mkl2	MKL/myocardin-like 2	ENSMUSG00000009569	161049	133,16
Mettl9	methyltransferase like 9	ENSMUSG00000030876	42390	133,09
Nuak1	NUAK family, SNF1-like kinase, 1	ENSMUSG00000020032	69693	133,02
Usp48	ubiquitin specific peptidase 48	ENSMUSG00000043411	64783	132,95
Dym	dymeclin	ENSMUSG00000035765	268195	132,87
Slmap	sarcolemma associated protein	ENSMUSG00000021870	121764	132,80
Uba1	ubiquitin-like modifier activating enzyme 1	ENSMUSG00000001924	24854	132,73
Smek1	SMEK homolog 1, suppressor of mek1 (Dictyostelium)	ENSMUSG00000041846	44294	132,73
Nrros	negative regulator of reactive oxygen species	ENSMUSG00000052384	22810	132,73
Gclc	glutamate-cysteine ligase, catalytic subunit	ENSMUSG00000032350	39951	132,73
Rai14	retinoic acid induced 14	ENSMUSG00000022246	145653	132,73
Zadh2	zinc binding alcohol dehydrogenase, domain containing 2	ENSMUSG00000049090	9357	132,58
Csnk2a1	casein kinase 2, alpha 1 polypeptide	ENSMUSG00000074698	55014	132,58
Nfe2l2	nuclear factor, erythroid derived 2, like 2	ENSMUSG00000015839	29129	132,44
		ENSMUSG00000030729	51475	132,29
Ing1	inhibitor of growth family, member 1	ENSMUSG00000045969	7185	132,15

Cmtm4	CKLF-like MARVEL transmembrane domain containing 4	ENSMUSG00000096188	47617	132,08
Whsc1	Wolf-Hirschhorn syndrome candidate 1 (human)	ENSMUSG00000057406	77251	132,08
Pou2f1	POU domain, class 2, transcription factor 1	ENSMUSG00000026565	137525	132,08
Gm10250	predicted pseudogene 10250	ENSMUSG00000068706	571	132,00
Sil1	endoplasmic reticulum chaperone SIL1 homolog (S. cerevisiae)	ENSMUSG00000024357	232526	132,00
Nbr1	neighbor of Brca1 gene 1	ENSMUSG00000017119	29803	131,93
Smarcd1	SWI/SNF related, matrix associated, actin dependent regulator of chromatin, subfamily d, member 1	ENSMUSG00000023018	11709	131,86
Mbnl1	muscleblind-like 1 (Drosophila)	ENSMUSG00000027763	128497	131,86
Hmgb2	high mobility group box 2	ENSMUSG00000054717	4157	131,79
Gcnt1	glucosaminyl (N-acetyl) transferase 1, core 2	ENSMUSG00000038843	30527	131,79
Plekha6	pleckstrin homology domain containing, family A member 6	ENSMUSG00000041757	57339	131,64
Top2b	topoisomerase (DNA) II beta	ENSMUSG00000017485	65609	131,64
2010300C02Rik	RIKEN cDNA 2010300C02 gene	ENSMUSG00000026090	108409	131,64
Elavl2	ELAV (embryonic lethal, abnormal vision, Drosophila)-like 2 (Hu antigen B)	ENSMUSG00000008489	150023	131,50
Actr2	ARP2 actin-related protein 2	ENSMUSG00000020152	50610	131,50
Ttc7	tetratricopeptide repeat domain 7	ENSMUSG00000036918	98884	131,42
Rspry1	ring finger and SPRY domain containing 1	ENSMUSG00000050079	58321	131,42
Anp32a	acidic (leucine-rich) nuclear phosphoprotein 32 family, member A	ENSMUSG00000032249	37520	131,35
Ell2	elongation factor RNA polymerase II 2	ENSMUSG00000001542	64873	131,35
Cdk13	cyclin-dependent kinase 13	ENSMUSG00000041297	89136	131,21
Add2	adducin 2 (beta)	ENSMUSG00000030000	41472	131,13
Ank	progressive ankylosis	ENSMUSG00000022265	128233	131,13
Gpbp1l1	GC-rich promoter binding protein 1-like 1	ENSMUSG00000034042	36225	130,84
		ENSMUSG00000018481	47869	130,84
F2rl1	coagulation factor II (thrombin) receptor-like 1	ENSMUSG00000021678	13509	130,84
Kazn	kazrin, periplakin interacting protein	ENSMUSG00000040606	137012	130,77
Shoc2	soc-2 (suppressor of clear) homolog (C. elegans)	ENSMUSG00000024976	88963	130,70
Bloc1s5	biogenesis of organelles complex-1, subunit 5, muted	ENSMUSG00000038982	32401	130,56
Eif3b	eukaryotic translation initiation factor 3, subunit B	ENSMUSG00000056076	24054	130,48
Nasp	nuclear autoantigenic sperm protein (histone-binding)	ENSMUSG00000028693	26890	130,48
Dynlt3	dynein light chain Tctex-type 3	ENSMUSG00000031176	8737	130,48
Eif2s2	eukaryotic translation initiation factor 2, subunit 2 (beta)	ENSMUSG00000074656	21526	130,41

Nap1l4	nucleosome assembly protein 1-like 4	ENSMUSG00000059119	35512	130,34
Axdnd1	axonemal dynein light chain domain containing 1	ENSMUSG00000026601	97651	130,34
Med21	mediator complex subunit 21	ENSMUSG00000030291	8056	130,12
Lgmn	legumain	ENSMUSG00000021190	45730	130,12
		ENSMUSG00000031214	336749	130,12
Rnf216	ring finger protein 216	ENSMUSG00000045078	122128	130,12
Cdc42	cell division cycle 42	ENSMUSG00000006699	38025	130,05
Otx2	orthodenticle homolog 2 (Drosophila)	ENSMUSG00000021848	9966	130,05
Esyt2	extended synaptotagmin-like protein 2	ENSMUSG00000021171	91875	130,05
Aqp3	aquaporin 3	ENSMUSG00000028435	5462	129,98
Ttll10	tubulin tyrosine ligase-like family, member 10	ENSMUSG00000029074	24505	129,83
Numa1	nuclear mitotic apparatus protein 1	ENSMUSG00000066306	45117	129,83
		ENSMUSG00000035498	45215	129,69
Garem	GRB2 associated, regulator of MAPK1	ENSMUSG00000042680	172938	129,69
Map4k3	mitogen-activated protein kinase kinase kinase kinase 3	ENSMUSG00000024242	147582	129,69
Srsf4	serine/arginine-rich splicing factor 4	ENSMUSG00000028911	28090	129,61
Ywhah	tyrosine 3-monooxygenase/tryptophan 5-monooxygenase activation protein, eta polypeptide	ENSMUSG00000018965	9151	129,54
Cntnap5c	contactin associated protein-like 5C	ENSMUSG00000038048	640773	129,47
		ENSMUSG00000022781	63053	129,47
		ENSMUSG00000024236	198760	129,47
Ephb2	Eph receptor B2	ENSMUSG00000028664	188450	129,40
Ctbp2	C-terminal binding protein 2	ENSMUSG00000030970	136792	129,40
Fdft1	farnesyl diphosphate farnesyl transferase 1	ENSMUSG00000021273	32641	129,32
Gm10132	predicted gene 10132	ENSMUSG00000063556	527	129,32
Mcl1	myeloid cell leukemia sequence 1	ENSMUSG00000038612	4456	129,32
Cox20	COX20 Cox2 chaperone	ENSMUSG00000026500	3564	129,25
Scai	suppressor of cancer cell invasion	ENSMUSG00000035236	124517	129,18
Minos1	mitochondrial inner membrane organizing system 1	ENSMUSG00000050608	29300	129,11
		ENSMUSG00000026087	8059	129,03
Tnks	tankyrase, TRF1-interacting ankyrin-related ADP-ribose polymerase	ENSMUSG00000031529	136512	129,03
Nedd4l	neural precursor cell expressed, developmentally down-regulated gene 4-like	ENSMUSG00000024589	330071	128,96
Mfap3l	microfibrillar-associated protein 3-like	ENSMUSG00000031647	43903	128,89
Ly6e	lymphocyte antigen 6 complex, locus E	ENSMUSG00000022587	4855	128,60
		ENSMUSG00000090625	16394	128,60
		ENSMUSG00000050556	86778	128,60
Phyh	phytanoyl-CoA hydroxylase	ENSMUSG00000026664	19712	128,53
Rnf10	ring finger protein 10	ENSMUSG00000041740	31487	128,53

Rhoc	ras homolog gene family, member C	ENSMUSG00000002233	5449	128,53
		ENSMUSG00000026260	34851	128,38
Leprotl1	leptin receptor overlapping transcript-like 1	ENSMUSG00000031513	11462	128,31
Ap1b1	adaptor protein complex AP-1, beta 1 subunit	ENSMUSG00000009090	55968	128,31
		ENSMUSG00000095041	7501	128,24
Fbrsl1	fibrosin-like 1	ENSMUSG00000043323	86750	128,24
Trak1	trafficking protein, kinesin binding 1	ENSMUSG00000032536	107959	128,16
Dctn2	dynactin 2	ENSMUSG00000025410	15698	127,95
Hnrnpll	heterogeneous nuclear ribonucleoprotein L-like	ENSMUSG00000024095	32848	127,95
Smek2	SMEK homolog 2, suppressor of mek1 (Dictyostelium)	ENSMUSG00000020463	47908	127,95
		ENSMUSG00000095403	31669	127,95
Ralgapb	Ral GTPase activating protein, beta subunit (non-catalytic)	ENSMUSG00000027652	89406	127,95
Sclt1	sodium channel and clathrin linker 1	ENSMUSG00000059834	115798	127,95
Eif3e	eukaryotic translation initiation factor 3, subunit E	ENSMUSG00000022336	32697	127,80
9130011E15Rik	RIKEN cDNA 9130011E15 gene	ENSMUSG00000039901	180345	127,73
Naa50	N(alpha)-acetyltransferase 50, NatE catalytic subunit	ENSMUSG00000022698	23537	127,66
H2afv	H2A histone family, member V	ENSMUSG00000041126	17215	127,51
Pdcd6ip	programmed cell death 6 interacting protein	ENSMUSG00000032504	56516	127,51
Wwtr1	WW domain containing transcription regulator 1	ENSMUSG00000027803	120262	127,51
Hba-a2	hemoglobin alpha, adult chain 2	ENSMUSG00000069917	810	127,44
		ENSMUSG00000021557	135691	127,44
Pik3r1	phosphatidylinositol 3-kinase, regulatory subunit, polypeptide 1 (p85 alpha)	ENSMUSG00000041417	87457	127,44
Mmp16	matrix metallopeptidase 16	ENSMUSG00000028226	266055	127,37
Tbc1d5	TBC1 domain family, member 5	ENSMUSG00000023923	446223	127,37
Pja2	praja 2, RING-H2 motif containing	ENSMUSG00000024083	50912	127,29
Ssb	Sjogren syndrome antigen B	ENSMUSG00000068882	10285	127,15
Zfp652	zinc finger protein 652	ENSMUSG00000075595	122443	127,15
Cox14	cytochrome c oxidase assembly protein 14	ENSMUSG00000023020	2519	126,93
Vezf1	vascular endothelial zinc finger 1	ENSMUSG00000018377	16451	126,93
H2afz	H2A histone family, member Z	ENSMUSG00000037894	2436	126,86
Amfr	autocrine motility factor receptor	ENSMUSG00000031751	41076	126,79
		ENSMUSG00000058126	2106	126,79
Hk2	hexokinase 2	ENSMUSG00000000628	49430	126,79
Cdk19	cyclin-dependent kinase 19	ENSMUSG00000038481	134511	126,64
Unc13c	unc-13 homolog C (C. elegans)	ENSMUSG00000062151	452698	126,64
Ccdc34	coiled-coil domain containing 34	ENSMUSG00000027160	155544	126,57
Ylpm1	YLP motif containing 1	ENSMUSG00000021244	74195	126,57
Galnt5	UDP-N-acetyl-alpha-D-galactosamine:polypeptide N-acetylgalactosaminyltransferase 5	ENSMUSG00000026828	47977	126,57
Sec24b	Sec24 related gene family, member B (S. cerevisiae)	ENSMUSG00000001052	78795	126,50

Smad4	SMAD family member 4	ENSMUSG00000024515	64772	126,50
Trib1	tribbles homolog 1 (Drosophila)	ENSMUSG00000032501	8201	126,50
Abi1	abl-interactor 1	ENSMUSG00000058835	100169	126,50
Nup50	nucleoporin 50	ENSMUSG00000016619	19528	126,35
		ENSMUSG00000094777	519	126,28
R3hdm1	R3H domain containing 1	ENSMUSG00000056211	134430	126,28
Keap1	kelch-like ECH-associated protein 1	ENSMUSG00000003308	9603	126,21
Cab39	calcium binding protein 39	ENSMUSG00000036707	58136	126,13
Ndufb7	NADH dehydrogenase (ubiquinone) 1 beta subcomplex, 7	ENSMUSG00000033938	4956	126,06
Ibtk	inhibitor of Bruton agammaglobulinemia tyrosine kinase	ENSMUSG00000035941	61974	125,77
Rnf150	ring finger protein 150	ENSMUSG00000047747	227916	125,77
Trim36	tripartite motif-containing 36	ENSMUSG00000033949	47306	125,77
Rps6kb1	ribosomal protein S6 kinase, polypeptide 1	ENSMUSG00000020516	45935	125,77
N4bp1	NEDD4 binding protein 1	ENSMUSG00000031652	44120	125,63
Srsf11	serine/arginine-rich splicing factor 11	ENSMUSG00000055436	26167	125,63
		ENSMUSG00000029249	20942	125,63
Slc22a14	solute carrier family 22 (organic cation transporter), member 14	ENSMUSG00000070280	196099	125,55
Dlgap4	discs, large homolog-associated protein 4 (Drosophila)	ENSMUSG00000061689	150659	125,48
Dcxr	dicarbonyl L-xylulose reductase	ENSMUSG00000039450	1883	125,48
Dmxl1	Dmx-like 1	ENSMUSG00000037416	132804	125,48
Smg1	SMG1 homolog, phosphatidylinositol 3-kinase-related kinase (C. elegans)	ENSMUSG00000030655	112330	125,34
Pkig	protein kinase inhibitor, gamma	ENSMUSG00000035268	67773	125,26
Tom1l2	target of myb1-like 2 (chicken)	ENSMUSG00000000538	126192	125,19
		ENSMUSG00000030717	3762	125,19
Apopt1	apoptogenic, mitochondrial 1	ENSMUSG00000037787	41720	125,19
Zmym4	zinc finger, MYM-type 4	ENSMUSG00000042446	106197	125,12
Cdkal1	CDK5 regulatory subunit associated protein 1-like 1	ENSMUSG00000006191	663929	125,05
Apbb1	amyloid beta (A4) precursor protein-binding, family B, member 1	ENSMUSG00000037032	15917	124,90
Eif5	eukaryotic translation initiation factor 5	ENSMUSG00000021282	8653	124,83
Kitl	kit ligand	ENSMUSG00000019966	84784	124,76
Slc25a37	solute carrier family 25, member 37	ENSMUSG00000034248	43253	124,68
Slc7a1	solute carrier family 7 (cationic amino acid transporter, y+ system), member 1	ENSMUSG00000041313	72495	124,54
Invs	inversin	ENSMUSG00000028344	152195	124,47
		ENSMUSG00000021395	51667	124,39
Zfp385b	zinc finger protein 385B	ENSMUSG00000027016	409006	124,39
Amd1	S-adenosylmethionine decarboxylase 1	ENSMUSG00000075232	14731	124,39
Gm684	predicted gene 684	ENSMUSG00000079559	8297	124,32
Ralgapa1	Ral GTPase activating protein, alpha subunit 1	ENSMUSG00000021027	218239	124,25
Per2	period circadian clock 2	ENSMUSG00000055866	43347	124,25

		ENSMUSG00000029221	51611	124,18
Rapgef1	Rap guanine nucleotide exchange factor (GEF) 1	ENSMUSG00000039844	121259	124,18
Pdzd2	PDZ domain containing 2	ENSMUSG00000022197	235503	124,10
Itsn2	intersectin 2	ENSMUSG00000020640	120943	123,96
Kansl1l	KAT8 regulatory NSL complex subunit 1-like	ENSMUSG00000026004	98315	123,89
Fcho2	FCH domain only 2	ENSMUSG00000041685	92043	123,74
Napg	N-ethylmaleimide sensitive fusion protein attachment protein gamma	ENSMUSG00000024581	21615	123,67
Tubb2a	tubulin, beta 2A class IIA	ENSMUSG00000058672	3708	123,67
Zfp131	zinc finger protein 131	ENSMUSG00000094870	25619	123,60
Snx29	sorting nexin 29	ENSMUSG00000071669	432565	123,60
Cyr61	cysteine rich protein 61	ENSMUSG00000028195	3015	123,52
Tnfaip1	tumor necrosis factor, alpha-induced protein 1 (endothelial)	ENSMUSG00000017615	13483	123,38
Fam102a	family with sequence similarity 102, member A	ENSMUSG00000039157	34425	123,38
Cdc42se1	CDC42 small effector 1	ENSMUSG00000046722	7678	123,31
		ENSMUSG00000039809	329556	123,31
D5Ertd579e	DNA segment, Chr 5, ERATO Doi 579, expressed	ENSMUSG00000029190	111237	123,31
Ephx1	epoxide hydrolase 1, microsomal	ENSMUSG00000038776	44750	123,31
		ENSMUSG00000043279	10566	123,31
Acin1	apoptotic chromatin condensation inducer 1	ENSMUSG00000022185	44771	123,23
Twsg1	twisted gastrulation homolog 1 (Drosophila)	ENSMUSG00000024098	28122	123,16
Tmx3	thioredoxin-related transmembrane protein 3	ENSMUSG00000024614	33114	123,16
Dbnl	drebrin-like	ENSMUSG00000020476	12475	123,09
		ENSMUSG00000094817	153439	123,09
Bsg	basigin	ENSMUSG00000023175	7479	123,02
		ENSMUSG00000028868	69252	123,02
Gnb2l1	guanine nucleotide binding protein (G protein), beta polypeptide 2 like 1	ENSMUSG00000020372	6103	122,94
Arf3	ADP-ribosylation factor 3	ENSMUSG00000051853	25594	122,94
		ENSMUSG00000027167	207245	122,94
Tesc	tescalcin	ENSMUSG00000029359	34136	122,87
Zcchc6	zinc finger, CCHC domain containing 6	ENSMUSG00000035248	51269	122,87
Drosha	drosha, ribonuclease type III	ENSMUSG00000022191	110476	122,80
Abcc1	ATP-binding cassette, sub-family C (CFTR/MRP), member 1	ENSMUSG00000023088	114180	122,80
		ENSMUSG00000044894	2910	122,73
Vezt	vezatin, adherens junctions transmembrane protein	ENSMUSG00000036099	96653	122,73
Rbm22	RNA binding motif protein 22	ENSMUSG00000024604	12075	122,65
		ENSMUSG00000039221	1917	122,65
Rab3ip	RAB3A interacting protein	ENSMUSG00000064181	44597	122,58
Rab11fip3	RAB11 family interacting protein 3 (class II)	ENSMUSG00000037098	80374	122,58
Elf1	E74-like factor 1	ENSMUSG00000036461	101283	122,58
Rsrc1	arginine/serine-rich coiled-coil 1	ENSMUSG00000034544	377007	122,51
Arf4	ADP-ribosylation factor 4	ENSMUSG00000021877	27011	122,51

Osbpl6	oxysterol binding protein-like 6	ENSMUSG00000042359	194140	122,51
Smdt1	single-pass membrane protein with aspartate rich tail 1	ENSMUSG00000022452	10133	122,36
		ENSMUSG00000055067	566082	122,36
Amd2	S-adenosylmethionine decarboxylase 2	ENSMUSG00000063953	3201	122,29
Mpzl1	myelin protein zero-like 1	ENSMUSG00000026566	42361	122,15
Ptpn12	protein tyrosine phosphatase, non-receptor type 12	ENSMUSG00000028771	69267	122,15
Snx6	sorting nexin 6	ENSMUSG00000005656	49355	122,00
Slc37a2	solute carrier family 37 (glycerol-3-phosphate transporter), member 2	ENSMUSG00000032122	26590	121,86
Sec11a	SEC11 homolog A (S. cerevisiae)	ENSMUSG00000025724	42892	121,86
Rala	v-ral simian leukemia viral oncogene homolog A (ras related)	ENSMUSG00000008859	63665	121,71
Iffo2	intermediate filament family orphan 2	ENSMUSG00000041025	89835	121,71
		ENSMUSG00000026483	150754	121,71
Psma6	proteasome (prosome, macropain) subunit, alpha type 6	ENSMUSG00000021024	34233	121,42
Bbs9	Bardet-Biedl syndrome 9 (human)	ENSMUSG00000035919	412566	121,42
Akap12	A kinase (PRKA) anchor protein (gravin) 12	ENSMUSG00000038587	93140	121,42
Chd3	chromodomain helicase DNA binding protein 3	ENSMUSG00000018474	26134	121,42
Sgta	small glutamine-rich tetratricopeptide repeat (TPR)-containing, alpha	ENSMUSG00000004937	16104	121,35
		ENSMUSG00000060534	1092332	121,35
Hspb8	heat shock protein 8	ENSMUSG00000041548	14374	121,28
Wdr7	WD repeat domain 7	ENSMUSG00000040560	281066	121,20
Mgat3	mannoside acetylglucosaminyltransferase 3	ENSMUSG00000042428	41799	121,20
Arfgef1	ADP-ribosylation factor guanine nucleotide-exchange factor 1(brefeldin A-inhibited)	ENSMUSG00000067851	95100	121,13
Pak7	p21 protein (Cdc42/Rac)-activated kinase 7	ENSMUSG00000039913	306864	121,06
Dsp	desmoplakin	ENSMUSG00000054889	47250	120,91
2700060E02Rik	RIKEN cDNA 2700060E02 gene	ENSMUSG00000021807	12422	120,70
Sep 07	septin 7	ENSMUSG00000001833	56133	120,70
Psd3	pleckstrin and Sec7 domain containing 3	ENSMUSG00000030465	373205	120,70
Acss3	acyl-CoA synthetase short-chain family member 3	ENSMUSG00000035948	187505	120,70
Mob4	MOB family member 4, phocein	ENSMUSG00000025979	23669	120,62
Sppl3	signal peptide peptidase 3	ENSMUSG00000029550	87654	120,62
Antxr1	anthrax toxin receptor 1	ENSMUSG00000033420	201922	120,55
Daam1	dishevelled associated activator of morphogenesis 1	ENSMUSG00000034574	161290	120,41
Rb1	retinoblastoma 1	ENSMUSG00000022105	142150	120,33
BC005764	cDNA sequence BC005764	ENSMUSG00000035835	14160	120,26
Galnt7	UDP-N-acetyl-alpha-D-galactosamine: polypeptide N-acetylgalactosaminyltransferase 7	ENSMUSG00000031608	129205	120,26
Alas1	aminolevulinic acid synthase 1	ENSMUSG00000032786	14672	120,26

Cacna1b	calcium channel, voltage-dependent, N type, alpha 1B subunit	ENSMUSG00000004113	159266	120,12
Dmtf1	cyclin D binding myb-like transcription factor 1	ENSMUSG00000042508	42949	120,12
		ENSMUSG00000026999	41671	120,04
Mxi1	Max interacting protein 1	ENSMUSG00000025025	65305	120,04
Mpped2	metallophosphoesterase domain containing 2	ENSMUSG00000016386	175088	119,97
Six3	sine oculis-related homeobox 3	ENSMUSG00000038805	18206	119,90
Clasp2	CLIP associating protein 2	ENSMUSG00000033392	107112	119,83
Uqcrfs1	ubiquinol-cytochrome c reductase, Rieske iron-sulfur polypeptide 1	ENSMUSG00000038462	5055	119,75
		ENSMUSG00000007617	100373	119,68
		ENSMUSG00000068240	475	119,46
Atp2c1	ATPase, Ca++-sequestering	ENSMUSG00000032570	117719	119,39
Fam135a	family with sequence similarity 135, member A	ENSMUSG00000026153	89584	119,39
Orc5	origin recognition complex, subunit 5	ENSMUSG00000029012	63945	119,32
Aldh18a1	aldehyde dehydrogenase 18 family, member A1	ENSMUSG00000025007	38207	119,32
Smim7	small integral membrane protein 7	ENSMUSG00000044600	5901	119,17
Phc2	polyhomeotic-like 2 (Drosophila)	ENSMUSG00000028796	98180	119,17
Cntnap2	contactin associated protein-like 2	ENSMUSG00000039419	2241309	119,17
Ppp2cb	protein phosphatase 2 (formerly 2A), catalytic subunit, beta isoform	ENSMUSG00000009630	20174	119,10
Swi5	SWI5 recombination repair homolog (yeast)	ENSMUSG00000044627	9260	119,03
Ccnt1	cyclin T1	ENSMUSG00000011960	32235	119,03
Gm10175	predicted gene 10175	ENSMUSG00000066724	589	118,96
Zmiz1	zinc finger, MIZ-type containing 1	ENSMUSG00000007817	207559	118,96
Grb2	growth factor receptor bound protein 2	ENSMUSG00000059923	64553	118,96
Pigq	phosphatidylinositol glycan anchor biosynthesis, class Q	ENSMUSG00000025728	18084	118,88
Usp25	ubiquitin specific peptidase 25	ENSMUSG00000022867	102711	118,88
		ENSMUSG00000069919	955	118,81
Arih2	ariadne homolog 2 (Drosophila)	ENSMUSG00000064145	46439	118,74
Pygb	brain glycogen phosphorylase	ENSMUSG00000033059	45024	118,67
Egln2	EGL nine homolog 2 (C. elegans)	ENSMUSG00000058709	8145	118,59
Hspa9	heat shock protein 9	ENSMUSG00000024359	16944	118,59
		ENSMUSG00000028995	115415	118,59
Espn	espin	ENSMUSG00000028943	32041	118,52
Tbc1d30	TBC1 domain family, member 30	ENSMUSG00000052302	47366	118,45
Sh3rf1	SH3 domain containing ring finger 1	ENSMUSG00000031642	171902	118,23
		ENSMUSG00000064356	204	118,23
Snrpg	small nuclear ribonucleoprotein polypeptide G	ENSMUSG00000057278	7320	118,16
Irf6	interferon regulatory factor 6	ENSMUSG00000026638	18931	118,16
Dnajc13	DnaJ (Hsp40) homolog, subfamily C, member 13	ENSMUSG00000032560	111334	118,16
Hnrnpul1	heterogeneous nuclear ribonucleoprotein U-like 1	ENSMUSG00000040725	33233	118,01
		ENSMUSG00000029622	16491	118,01

Dynlrb1	dynein light chain roadblock-type 1	ENSMUSG00000047459	13745	117,87
Fry	furry homolog (Drosophila)	ENSMUSG00000056602	237823	117,87
		ENSMUSG00000025856	21357	117,80
Tsc22d2	TSC22 domain family, member 2	ENSMUSG00000027806	51099	117,80
Gm10288	predicted gene 10288	ENSMUSG00000070343	591	117,65
Efcab14	EF-hand calcium binding domain 14	ENSMUSG00000034210	39584	117,65
Arap2	ArfGAP with RhoGAP domain, ankyrin repeat and PH domain 2	ENSMUSG00000037999	163754	117,58
Dusp16	dual specificity phosphatase 16	ENSMUSG00000030203	77153	117,51
Fgfr1	fibroblast growth factor receptor 1	ENSMUSG00000031565	62061	117,51
Sbf2	SET binding factor 2	ENSMUSG00000038371	306910	117,51
Kifap3	kinesin-associated protein 3	ENSMUSG00000026585	137527	117,43
Rfx7	regulatory factor X, 7	ENSMUSG00000037674	90698	117,43
Cdk5rap1	CDK5 regulatory subunit associated protein 1	ENSMUSG00000027487	37631	117,36
A130010J15Rik	RIKEN cDNA A130010J15 gene	ENSMUSG00000079144	9784	117,29
Ppm1a	protein phosphatase 1A, magnesium dependent, alpha isoform	ENSMUSG00000021096	33730	117,29
Xpr1	xenotropic and polytropic retrovirus receptor 1	ENSMUSG00000026469	141715	117,29
Gprasp1	G protein-coupled receptor associated sorting protein 1	ENSMUSG00000043384	60742	117,22
Tc2n	tandem C2 domains, nuclear	ENSMUSG00000021187	73081	117,22
Tlk2	tousled-like kinase 2 (Arabidopsis)	ENSMUSG00000020694	105153	117,14
Hivep2	human immunodeficiency virus type I enhancer binding protein 2	ENSMUSG00000015501	184998	117,14
		ENSMUSG00000051367	16959	117,07
Cotl1	coactosin-like 1 (Dictyostelium)	ENSMUSG00000031827	31355	117,00
Nono	non-POU-domain-containing, octamer binding protein	ENSMUSG00000031311	19274	117,00
Rnf4	ring finger protein 4	ENSMUSG00000029110	19341	117,00
Dstyk	dual serine/threonine and tyrosine protein kinase	ENSMUSG00000042046	49507	117,00
Helz	helicase with zinc finger domain	ENSMUSG00000020721	145897	117,00
		ENSMUSG00000071052	807	116,93
Gm9844	predicted pseudogene 9844	ENSMUSG00000050347	474	116,93
Zfp280d	zinc finger protein 280D	ENSMUSG00000038535	88872	116,93
Snca	synuclein, alpha	ENSMUSG00000025889	98283	116,64
Ssrp1	structure specific recognition protein 1	ENSMUSG00000027067	9876	116,64
Slc39a7	solute carrier family 39 (zinc transporter), member 7	ENSMUSG00000024327	3424	116,64
Fnbp4	formin binding protein 4	ENSMUSG00000008200	35652	116,64
Dcaf8	DDB1 and CUL4 associated factor 8	ENSMUSG00000026554	48379	116,56
Jakmip2	janus kinase and microtubule interacting protein 2	ENSMUSG00000024502	156366	116,42
Zmym2	zinc finger, MYM-type 2	ENSMUSG00000021945	74785	116,42
Cog5	component of oligomeric golgi complex 5	ENSMUSG00000035933	282762	116,42
Satb1	special AT-rich sequence binding protein 1	ENSMUSG00000023927	97104	116,27
Ap2b1	adaptor-related protein complex 2, beta 1 subunit	ENSMUSG00000035152	106012	116,27

Dnajc1	DnaJ (Hsp40) homolog, subfamily C, member 1	ENSMUSG00000026740	197177	116,27
Adam28	a disintegrin and metallopeptidase domain 28	ENSMUSG00000014725	49816	116,20
Tmprss6	transmembrane serine protease 6	ENSMUSG00000016942	28967	116,13
Tax1bp3	Tax1 (human T cell leukemia virus type I) binding protein 3	ENSMUSG00000040158	6080	116,13
Gmcl1	germ cell-less homolog 1 (Drosophila)	ENSMUSG00000001157	41611	115,91
Sf3a1	splicing factor 3a, subunit 1	ENSMUSG00000002129	22192	115,91
Apba2	amyloid beta (A4) precursor protein-binding, family A, member 2	ENSMUSG00000030519	252165	115,91
Srp14	signal recognition particle 14	ENSMUSG00000009549	3862	115,77
Fem1b	feminization 1 homolog b (C. elegans)	ENSMUSG00000032244	19817	115,77
Cenpf	centromere protein F	ENSMUSG00000026605	47488	115,77
Hnrnph3	heterogeneous nuclear ribonucleoprotein H3	ENSMUSG00000020069	9554	115,77
		ENSMUSG00000025159	40072	115,69
Dnaja2	DnaJ (Hsp40) homolog, subfamily A, member 2	ENSMUSG00000031701	17632	115,62
S100a1	S100 calcium binding protein A1	ENSMUSG00000044080	3359	115,62
Brdt	bromodomain, testis-specific	ENSMUSG00000029279	55900	115,48
Ube2e2	ubiquitin-conjugating enzyme E2E 2	ENSMUSG00000058317	320693	115,48
Lrp6	low density lipoprotein receptor-related protein 6	ENSMUSG00000030201	120490	115,40
Desi2	desumoylating isopeptidase 2	ENSMUSG00000026502	65182	115,40
Cx3cr1	chemokine (C-X3-C) receptor 1	ENSMUSG00000052336	166668	115,33
		ENSMUSG00000041609	83447	115,26
Dnaja3	DnaJ (Hsp40) homolog, subfamily A, member 3	ENSMUSG00000004069	67707	115,19
Nt5dc1	5'-nucleotidase domain containing 1	ENSMUSG00000039480	130265	115,19
Nrep	neuronal regeneration related protein	ENSMUSG00000042834	27011	115,11
D130043K22Rik	RIKEN cDNA D130043K22 gene	ENSMUSG00000006711	56136	115,11
Mapk1	mitogen-activated protein kinase 1	ENSMUSG00000063358	64072	115,11
Blvrb	biliverdin reductase B (flavin reductase (NADPH))	ENSMUSG00000040466	18167	115,11
Pum2	pumilio 2 (Drosophila)	ENSMUSG00000020594	78448	114,97
Dyrk1a	dual-specificity tyrosine-(Y)-phosphorylation regulated kinase 1a	ENSMUSG00000022897	125508	114,97
Pak3	p21 protein (Cdc42/Rac)-activated kinase 3	ENSMUSG00000031284	279206	114,90
Qrich1	glutamine-rich 1	ENSMUSG00000006673	43081	114,90
Cpeb1	cytoplasmic polyadenylation element binding protein 1	ENSMUSG00000025586	108440	114,82
Mapk8ip3	mitogen-activated protein kinase 8 interacting protein 3	ENSMUSG00000024163	44825	114,75
Smc3	structural maintenance of chromosomes 3	ENSMUSG00000024974	45436	114,68
Sipa1l1	signal-induced proliferation-associated 1 like 1	ENSMUSG00000042700	281767	114,68

Mpp7	membrane protein, palmitoylated 7 (MAGUK p55 subfamily member 7)	ENSMUSG00000057440	278902	114,68
Ubqln1	ubiquilin 1	ENSMUSG00000005312	39498	114,53
Nup210l	nucleoporin 210-like	ENSMUSG00000027939	107886	114,39
		ENSMUSG00000036964	18414	114,39
Galnt1	UDP-N-acetyl-alpha-D-galactosamine:polypeptide N-acetylgalactosaminyltransferase 1	ENSMUSG00000000420	81475	114,24
Nr2e1	nuclear receptor subfamily 2, group E, member 1	ENSMUSG00000019803	21670	114,24
		ENSMUSG00000031309	157938	114,24
Plxna2	plexin A2	ENSMUSG00000026640	197041	114,24
Ttc19	tetratricopeptide repeat domain 19	ENSMUSG00000042298	46979	114,17
		ENSMUSG00000055447	59393	114,03
Pcdh15	protocadherin 15	ENSMUSG00000052613	1546973	114,03
Ttc14	tetratricopeptide repeat domain 14	ENSMUSG00000027677	14906	113,95
Rassf1	Ras association (RalGDS/AF-6) domain family member 1	ENSMUSG00000010067	10709	113,95
Dst	dystonin	ENSMUSG00000026131	400428	113,88
Wfdc2	WAP four-disulfide core domain 2	ENSMUSG00000017723	6098	113,88
Knop1	lysine rich nucleolar protein 1	ENSMUSG00000030980	14033	113,81
Ier5	immediate early response 5	ENSMUSG00000056708	3270	113,74
Kxd1	KxDL motif containing 1	ENSMUSG00000055553	19685	113,66
Fam214a	family with sequence similarity 214, member A	ENSMUSG00000034858	79416	113,66
Pmepa1	prostate transmembrane protein, androgen induced 1	ENSMUSG00000038400	52076	113,59
Ryk	receptor-like tyrosine kinase	ENSMUSG00000032547	73389	113,59
		ENSMUSG00000044424	579	113,59
Wdfy1	WD repeat and FYVE domain containing 1	ENSMUSG00000073643	73882	113,59
Synrg	synergin, gamma	ENSMUSG00000034940	80151	113,37
Syf2	SYF2 homolog, RNA splicing factor (S. cerevisiae)	ENSMUSG00000028821	6651	113,30
Gnai2	guanine nucleotide binding protein (G protein), alpha inhibiting 2	ENSMUSG00000032562	21205	113,30
Camk1d	calcium/calmodulin-dependent protein kinase ID	ENSMUSG00000039145	421059	113,16
Mob1b	MOB kinase activator 1B	ENSMUSG00000006262	41204	113,08
Ammecr1l	AMME chromosomal region gene 1-like	ENSMUSG00000041915	24248	113,08
Sqstm1	sequestosome 1	ENSMUSG00000015837	11462	112,94
Slc44a1	solute carrier family 44, member 1	ENSMUSG00000028412	182066	112,94
Ep300	E1A binding protein p300	ENSMUSG00000055024	65864	112,94
Plch1	phospholipase C, eta 1	ENSMUSG00000036834	203239	112,94
Ptprn2	protein tyrosine phosphatase, receptor type, N polypeptide 2	ENSMUSG00000056553	792448	112,79
		ENSMUSG00000091955	485	112,72
Zyg11b	zyg-ll family member B, cell cycle regulator	ENSMUSG00000034636	71373	112,72
Sv2c	synaptic vesicle glycoprotein 2c	ENSMUSG00000051111	173135	112,72
		ENSMUSG00000039270	107931	112,65
Nr3c1	nuclear receptor subfamily 3, group C, member 1	ENSMUSG00000024431	80757	112,65
Zdhhc20	zinc finger, DHHC domain containing 20	ENSMUSG00000021969	57561	112,65

Ppp4r2	protein phosphatase 4, regulatory subunit 2	ENSMUSG00000052144	35080	112,58
Uqcrc2	ubiquinol cytochrome c reductase core protein 2	ENSMUSG00000030884	24303	112,50
Gde1	glycerophosphodiester phosphodiesterase 1	ENSMUSG00000033917	17234	112,50
		ENSMUSG00000019897	6767	112,50
Rrp12	ribosomal RNA processing 12 homolog (S. cerevisiae)	ENSMUSG00000035049	33302	112,50
Cpeb4	cytoplasmic polyadenylation element binding protein 4	ENSMUSG00000020300	63424	112,43
Ttc39b	tetratricopeptide repeat domain 39B	ENSMUSG00000038172	103956	112,21
Flnb	filamin, beta	ENSMUSG00000025278	133632	112,21
Tspan5	tetraspanin 5	ENSMUSG00000028152	162239	112,21
Dip2b	DIP2 disco-interacting protein 2 homolog B (Drosophila)	ENSMUSG00000023026	180810	112,21
Pi4ka	phosphatidylinositol 4-kinase, catalytic, alpha polypeptide	ENSMUSG00000041720	125964	112,21
Epb4.1l5	erythrocyte protein band 4.1-like 5	ENSMUSG00000026383	103968	112,14
Plin3	perilipin 3	ENSMUSG00000024197	11550	112,07
Herc4	hect domain and RLD 4	ENSMUSG00000020064	74085	112,07
Nsg1	neuron specific gene family member 1	ENSMUSG00000029126	22275	112,00
Dennd1b	DENN/MADD domain containing 1B	ENSMUSG00000056268	212608	112,00
Cggbp1	CGG triplet repeat binding protein 1	ENSMUSG00000054604	7512	111,93
Ddx50	DEAD (Asp-Glu-Ala-Asp) box polypeptide 50	ENSMUSG00000020076	35196	111,93
Snrpa	small nuclear ribonucleoprotein polypeptide A	ENSMUSG00000061479	9267	111,78
		ENSMUSG00000044694	626	111,78
Dhx30	DEAH (Asp-Glu-Ala-His) box polypeptide 30	ENSMUSG00000032480	33511	111,71
Dennd4c	DENN/MADD domain containing 4C	ENSMUSG00000038024	102049	111,71
Ndufa2	NADH dehydrogenase (ubiquinone) 1 alpha subcomplex, 2	ENSMUSG00000014294	2226	111,64
Cep85l	centrosomal protein 85-like	ENSMUSG00000038594	101771	111,64
Setd8	SET domain containing (lysine methyltransferase) 8	ENSMUSG00000049327	22379	111,56
Sirpa	signal-regulatory protein alpha	ENSMUSG00000037902	39394	111,56
Usp22	ubiquitin specific peptidase 22	ENSMUSG00000042506	23271	111,42
Slc23a2	solute carrier family 23 (nucleobase transporters), member 2	ENSMUSG00000027340	92613	111,42
Tmed9	transmembrane emp24 protein transport domain containing 9	ENSMUSG00000058569	4529	111,35
Dapp1	dual adaptor for phosphotyrosine and 3-phosphoinositides 1	ENSMUSG00000028159	50539	111,35
Cycs	cytochrome c, somatic	ENSMUSG00000063694	3976	111,20
Gm10335	predicted gene 10335	ENSMUSG00000071532	510	111,06
Fam177a	family with sequence similarity 177, member A	ENSMUSG00000095595	17551	111,06
Rab3d	RAB3D, member RAS oncogene family	ENSMUSG00000019066	10702	110,98

Srf	serum response factor	ENSMUSG00000015605	9324	110,91
Ucp2	uncoupling protein 2 (mitochondrial, proton carrier)	ENSMUSG00000033685	8684	110,84
Atp8a2	ATPase, aminophospholipid transporter-like, class I, type 8A, member 2	ENSMUSG00000021983	549649	110,84
Cachd1	cache domain containing 1	ENSMUSG00000028532	252546	110,84
Rab5a	RAB5A, member RAS oncogene family	ENSMUSG00000017831	28438	110,77
Rap1b	RAS related protein 1b	ENSMUSG00000052681	31465	110,77
Krt23	keratin 23	ENSMUSG00000006777	15164	110,77
Phf23	PHD finger protein 23	ENSMUSG00000018572	4233	110,62
Map2k4	mitogen-activated protein kinase kinase 4	ENSMUSG00000033352	100055	110,62
Slc11a2	solute carrier family 11 (proton-coupled divalent metal ion transporters), member 2	ENSMUSG00000023030	37175	110,62
Pafah1b2	platelet-activating factor acetylhydrolase, isoform 1b, subunit 2	ENSMUSG00000003131	19561	110,55
Cyp51	cytochrome P450, family 51	ENSMUSG00000001467	23602	110,55
Usp1	ubiquitin specific peptidase 1	ENSMUSG00000028560	11734	110,48
Rnf6	ring finger protein (C3H2C3 type) 6	ENSMUSG00000029634	12364	110,40
Pcnt	pericentrin (kendrin)	ENSMUSG00000001151	91659	110,40
		ENSMUSG00000014748	6932	110,33
		ENSMUSG00000031995	42452	110,26
Rhoq	ras homolog gene family, member Q	ENSMUSG00000024143	36988	110,19
Klhl23	kelch-like 23	ENSMUSG00000042155	14708	110,19
Tspan14	tetraspanin 14	ENSMUSG00000037824	60363	110,11
Atp5d	ATP synthase, H+ transporting, mitochondrial F1 complex, delta subunit	ENSMUSG00000003072	7187	110,04
Rps24	ribosomal protein S24	ENSMUSG00000025290	5466	110,04
Plxna1	plexin A1	ENSMUSG00000030084	46298	110,04
Abhd17a	abhydrolase domain containing 17A	ENSMUSG00000003346	6693	109,97
Met	met proto-oncogene	ENSMUSG00000009376	110181	109,97
		ENSMUSG00000028333	21756	109,90
Mt2	metallothionein 2	ENSMUSG00000031762	950	109,82
Slc45a4	solute carrier family 45, member 4	ENSMUSG00000079020	68339	109,82
Ascc3	activating signal cointegrator 1 complex subunit 3	ENSMUSG00000038774	258534	109,82
Phf17	PHD finger protein 17	ENSMUSG00000025764	61129	109,75
		ENSMUSG00000059742	490874	109,75
Rgl1	ral guanine nucleotide dissociation stimulator,-like 1	ENSMUSG00000026482	249593	109,75
Rpl38	ribosomal protein L38	ENSMUSG00000057322	3808	109,68
		ENSMUSG00000036427	28168	109,68
Limch1	LIM and calponin homology domains 1	ENSMUSG00000037736	311325	109,68
Scgb1c1	secretoglobin, family 1C, member 1	ENSMUSG00000038801	1204	109,61
Zfp503	zinc finger protein 503	ENSMUSG00000039081	5640	109,53
Sh3d19	SH3 domain protein D19	ENSMUSG00000028082	159418	109,53
Mapk11	mitogen-activated protein kinase 11	ENSMUSG00000053137	7121	109,46

Bcl2	B cell leukemia/lymphoma 2	ENSMUSG00000057329	176097	109,39
		ENSMUSG00000054640	260917	109,39
Hsph1	heat shock 105kDa/110kDa protein 1	ENSMUSG00000029657	22090	109,32
Dock7	dedicator of cytokinesis 7	ENSMUSG00000028556	184251	109,24
4930506M07Rik	RIKEN cDNA 4930506M07 gene	ENSMUSG00000041362	102712	109,24
Jag1	jagged 1	ENSMUSG00000027276	35189	109,24
AW554918	expressed sequence AW554918	ENSMUSG00000033632	298323	109,24
Fbxw2	F-box and WD-40 domain protein 2	ENSMUSG00000035949	21798	109,17
Snrk	SNF related kinase	ENSMUSG00000038145	52420	109,10
Gorasp2	golgi reassembly stacking protein 2	ENSMUSG00000014959	51061	109,10
Fgf12	fibroblast growth factor 12	ENSMUSG00000022523	285108	109,03
Serpine2	serine (or cysteine) peptidase inhibitor, clade E, member 2	ENSMUSG00000026249	64500	108,95
Jak1	Janus kinase 1	ENSMUSG00000028530	112916	108,95
Rps5	ribosomal protein S5	ENSMUSG00000012848	4397	108,81
Lonp2	lon peptidase 2, peroxisomal	ENSMUSG00000047866	99831	108,81
Add3	adducin 3 (gamma)	ENSMUSG00000025026	106955	108,74
		ENSMUSG00000058239	11552	108,66
Mpc2	mitochondrial pyruvate carrier 2	ENSMUSG00000026568	20178	108,66
Ndufb5	NADH dehydrogenase (ubiquinone) 1 beta subcomplex, 5	ENSMUSG00000027673	14577	108,59
Rtf1	Rtf1, Paf1/RNA polymerase II complex component, homolog (S. cerevisiae)	ENSMUSG00000027304	60340	108,59
Kif2a	kinesin family member 2A	ENSMUSG00000021693	63131	108,59
Pappa	pregnancy-associated plasma protein A	ENSMUSG00000028370	233336	108,59
Nipal2	NIPA-like domain containing 2	ENSMUSG00000038879	105908	108,52
2810474O19Rik	RIKEN cDNA 2810474O19 gene	ENSMUSG00000032712	26245	108,37
		ENSMUSG00000015354	60496	108,23
Cgn	cingulin	ENSMUSG00000068876	26424	108,23
		ENSMUSG00000049124	386	108,16
Plekhf2	pleckstrin homology domain containing, family F (with FYVE domain) member 2	ENSMUSG00000049969	19266	108,16
Crtc3	CREB regulated transcription coactivator 3	ENSMUSG00000030527	102251	108,16
Suv420h1	suppressor of variegation 4-20 homolog 1 (Drosophila)	ENSMUSG00000045098	50883	108,16
		ENSMUSG00000021188	79225	108,16
Senp2	SUMO/sentrin specific peptidase 2	ENSMUSG00000022855	39786	108,16
Gsr	glutathione reductase	ENSMUSG00000031584	45641	108,01
Ldha	lactate dehydrogenase A	ENSMUSG00000063229	14153	107,94
Tmem106b	transmembrane protein 106B	ENSMUSG00000029571	19511	107,94
Myo5a	myosin VA	ENSMUSG00000034593	152674	107,94
Psmd12	proteasome (prosome, macropain) 26S subunit, non-ATPase, 12	ENSMUSG00000020720	24879	107,87
Efcab8	EF-hand calcium binding domain 8	ENSMUSG00000044083	63874	107,87
Scaf4	SR-related CTD-associated factor 4	ENSMUSG00000022983	55469	107,87
Capn2	calpain 2	ENSMUSG00000026509	50240	107,87
Cacfd1	calcium channel flower domain containing 1	ENSMUSG00000015488	11164	107,79
Crip2	cysteine rich protein 2	ENSMUSG00000006356	5269	107,79
Rpl18a	ribosomal protein L18A	ENSMUSG00000045128	2722	107,79

		ENSMUSG00000045438	9045	107,72
Csnk1e	casein kinase 1, epsilon	ENSMUSG00000022433	26064	107,72
Ckap5	cytoskeleton associated protein 5	ENSMUSG00000040549	93903	107,72
Ddit3	DNA-damage inducible transcript 3	ENSMUSG00000025408	5515	107,65
Lsm14a	LSM14 homolog A (SCD6, S. cerevisiae)	ENSMUSG00000066568	44895	107,65
Abcc5	ATP-binding cassette, sub-family C (CFTR/MRP), member 5	ENSMUSG00000022822	95092	107,58
Cul5	cullin 5	ENSMUSG00000032030	55433	107,50
Vps37a	vacuolar protein sorting 37A (yeast)	ENSMUSG00000031600	39352	107,50
Cyb561	cytochrome b-561	ENSMUSG00000019590	19635	107,43
Timm13	translocase of inner mitochondrial membrane 13	ENSMUSG00000020219	1520	107,43
Lpin2	lipin 2	ENSMUSG00000024052	67258	107,43
Maob	monoamine oxidase B	ENSMUSG00000040147	108085	107,43
Hbegf	heparin-binding EGF-like growth factor	ENSMUSG00000024486	10877	107,43
		ENSMUSG00000021139	174046	107,36
		ENSMUSG00000023826	568322	107,36
Vimp	VCP-interacting membrane protein	ENSMUSG00000075701	9764	107,21
Dido1	death inducer-obliterator 1	ENSMUSG00000038914	52036	107,21
		ENSMUSG00000034765	13323	107,21
Taf3	TAF3 RNA polymerase II, TATA box binding protein (TBP)-associated factor	ENSMUSG00000025782	134045	107,14
		ENSMUSG00000051586	71447	107,14
		ENSMUSG00000028341	41295	107,14
Tnpo3	transportin 3	ENSMUSG00000012535	69061	107,07
Tbl1xr1	transducin (beta)-like 1X-linked receptor 1	ENSMUSG00000027630	139943	107,07
Zdhhc14	zinc finger, DHHC domain containing 14	ENSMUSG00000034265	261292	107,07
Prpf8	pre-mRNA processing factor 8	ENSMUSG00000020850	22634	107,00
Kdm4c	lysine (K)-specific demethylase 4C	ENSMUSG00000028397	163364	107,00
Serf2	small EDRK-rich factor 2	ENSMUSG00000074884	9119	106,92
Sugt1	SGT1, suppressor of G2 allele of SKP1 (S. cerevisiae)	ENSMUSG00000022024	42065	106,78
Qser1	glutamine and serine rich 1	ENSMUSG00000074994	61902	106,78
Dsg2	desmoglein 2	ENSMUSG00000044393	46448	106,78
Nr4a1	nuclear receptor subfamily 4, group A, member 1	ENSMUSG00000023034	7947	106,78
Bcap31	B cell receptor associated protein 31	ENSMUSG00000002015	29998	106,71
		ENSMUSG00000029227	63342	106,71
Insm1	insulinoma-associated 1	ENSMUSG00000068154	3096	106,63
Pde4a	phosphodiesterase 4A, cAMP specific	ENSMUSG00000032177	47535	106,63
		ENSMUSG00000031660	31156	106,63
		ENSMUSG00000026349	33898	106,63
Tmem163	transmembrane protein 163	ENSMUSG00000026347	191551	106,56
Sdcbp	syndecan binding protein	ENSMUSG00000028249	42774	106,49
Epc2	enhancer of polycomb homolog 2 (Drosophila)	ENSMUSG00000069495	100463	106,49
		ENSMUSG00000074212	48883	106,42
Ldlr	low density lipoprotein receptor	ENSMUSG00000032193	26341	106,42

Vmac	vimentin-type intermediate filament associated coiled-coil protein	ENSMUSG00000054723	3768	106,34
Cntn5	contactin 5	ENSMUSG00000039488	1243885	106,34
Hspb1	heat shock protein 1	ENSMUSG00000004951	1645	106,34
Zzz3	zinc finger, ZZ domain containing 3	ENSMUSG00000039068	67354	106,27
Lclat1	lysocardiolipin acyltransferase 1	ENSMUSG00000054469	135384	106,20
U2surp	U2 snRNP-associated SURP domain containing	ENSMUSG00000032407	55103	106,20
Kcnk10	potassium channel, subfamily K, member 10	ENSMUSG00000033854	143947	106,13
Ssr3	signal sequence receptor, gamma	ENSMUSG00000027828	12969	106,05
Rp2h	retinitis pigmentosa 2 homolog (human)	ENSMUSG00000060090	41173	106,05
Nqo1	NAD(P)H dehydrogenase, quinone 1	ENSMUSG00000003849	14982	105,98
Ankrd44	ankyrin repeat domain 44	ENSMUSG00000052331	281048	105,98
Ust	uronyl-2-sulfotransferase	ENSMUSG00000047712	314073	105,98
Ubl3	ubiquitin-like 3	ENSMUSG00000001687	48159	105,91
Itch	itchy, E3 ubiquitin protein ligase	ENSMUSG00000027598	93347	105,84
Rnps1	ribonucleic acid binding protein S1	ENSMUSG00000034681	11221	105,76
Psmd1	proteasome (prosome, macropain) 26S subunit, non-ATPase, 1	ENSMUSG00000026229	74765	105,76
Mid1ip1	Mid1 interacting protein 1 (gastrulation specific G12-like (zebrafish))	ENSMUSG00000008035	4678	105,69
Capzb	capping protein (actin filament) muscle Z-line, beta	ENSMUSG00000028745	98920	105,69
		ENSMUSG00000052305	1563	105,62
Vapb	vesicle-associated membrane protein, associated protein B and C	ENSMUSG00000054455	46826	105,62
Arl4a	ADP-ribosylation factor-like 4A	ENSMUSG00000047446	32579	105,47
Gas1	growth arrest specific 1	ENSMUSG00000052957	2961	105,40
Matr3	matrin 3	ENSMUSG00000037236	29888	105,40
Ubxn8	UBX domain protein 8	ENSMUSG00000052906	22391	105,33
Tle1	transducin-like enhancer of split 1, homolog of Drosophila E(spl)	ENSMUSG00000008305	83778	105,33
Atf6	activating transcription factor 6	ENSMUSG00000026663	163098	105,33
Gpr155	G protein-coupled receptor 155	ENSMUSG00000041762	45067	105,33
Tcp1	t-complex protein 1	ENSMUSG00000068039	9367	105,26
Sort1	sortilin 1	ENSMUSG00000068747	77421	105,26
Map3k3	mitogen-activated protein kinase kinase kinase 3	ENSMUSG00000020700	70834	105,18
Kif16b	kinesin family member 16B	ENSMUSG00000038844	284058	105,18
Pld5	phospholipase D family, member 5	ENSMUSG00000055214	313007	105,11
Supt5	suppressor of Ty 5	ENSMUSG00000003435	23822	105,04
Ndufs2	NADH dehydrogenase (ubiquinone) Fe-S protein 2	ENSMUSG00000013593	12270	104,97
Pp2d1	protein phosphatase 2C-like domain containing 1	ENSMUSG00000044957	31992	104,97
Fbxl20	F-box and leucine-rich repeat protein 20	ENSMUSG00000020883	67848	104,97
Ahcyl2	S-adenosylhomocysteine hydrolase-like 2	ENSMUSG00000029772	144300	104,89
Hes6	hairy and enhancer of split 6	ENSMUSG00000067071	2556	104,82
Flot2	flotillin 2	ENSMUSG00000061981	22504	104,82

Jhdm1d	jumonji C domain-containing histone demethylase 1 homolog D (S. cerevisiae)	ENSMUSG00000042599	70167	104,82
Tia1	cytotoxic granule-associated RNA binding protein 1	ENSMUSG00000071337	29185	104,75
Pnrc2	proline-rich nuclear receptor coactivator 2	ENSMUSG00000028675	2933	104,60
Ppp2r2b	protein phosphatase 2 (formerly 2A), regulatory subunit B (PR 52), beta isoform	ENSMUSG00000024500	422040	104,60
Entpd1	ectonucleoside triphosphate diphosphohydrolase 1	ENSMUSG00000048120	129237	104,53
Tomm20	translocase of outer mitochondrial membrane 20 homolog (yeast)	ENSMUSG00000093904	15258	104,46
Tbca	tubulin cofactor A	ENSMUSG00000042043	53957	104,46
		ENSMUSG00000022194	5670	104,39
Srebf2	sterol regulatory element binding factor 2	ENSMUSG00000022463	58111	104,39
Gm10221	predicted gene 10221	ENSMUSG00000067719	422	104,31
Hdgfrp3	hepatoma-derived growth factor, related protein 3	ENSMUSG00000025104	53223	104,31
Mmp14	matrix metallopeptidase 14 (membrane-inserted)	ENSMUSG00000000957	9655	104,17
Ubap2l	ubiquitin associated protein 2-like	ENSMUSG00000042520	52887	104,17
Ubap2	ubiquitin-associated protein 2	ENSMUSG00000028433	80832	104,17
Ptger3	prostaglandin E receptor 3 (subtype EP3)	ENSMUSG00000040016	77867	104,17
Emg1	EMG1 nucleolar protein homolog (S. cerevisiae)	ENSMUSG00000004268	7798	104,10
Atp6v0a1	ATPase, H+ transporting, lysosomal V0 subunit A1	ENSMUSG00000019302	54268	104,10
Zfp263	zinc finger protein 263	ENSMUSG00000022529	6698	104,10
Cry2	cryptochrome 2 (photolyase-like)	ENSMUSG00000068742	30398	104,10
4932438A13Rik	RIKEN cDNA 4932438A13 gene	ENSMUSG00000037270	189928	103,95
Mat2a	methionine adenosyltransferase II, alpha	ENSMUSG00000053907	6760	103,88
		ENSMUSG00000079057	63546	103,88
		ENSMUSG00000027651	103801	103,73
Mical2	microtubule associated monooxygenase, calponin and LIM domain containing 2	ENSMUSG00000038244	129339	103,73
Btaf1	BTAF1 RNA polymerase II, B-TFIID transcription factor-associated, (Mot1 homolog, S. cerevisiae)	ENSMUSG00000040565	86674	103,73
Tubb3	tubulin, beta 3 class III	ENSMUSG00000062380	10592	103,66
Ubxn4	UBX domain protein 4	ENSMUSG00000026353	35197	103,66
Ap3d1	adaptor-related protein complex 3, delta 1 subunit	ENSMUSG00000020198	35234	103,59
Polb	polymerase (DNA directed), beta	ENSMUSG00000031536	25319	103,59
Tbcd	tubulin-specific chaperone d	ENSMUSG00000039230	165216	103,59
Lmbrd1	LMBR1 domain containing 1	ENSMUSG00000073725	87756	103,52
Gpatch2	G patch domain containing 2	ENSMUSG00000039210	136197	103,52
Sptlc1	serine palmitoyltransferase, long chain base subunit 1	ENSMUSG00000021468	44650	103,44
Crim1	cysteine rich transmembrane BMP regulator 1 (chordin like)	ENSMUSG00000024074	176345	103,23

Erdr1	erythroid differentiation regulator 1	ENSMUSG00000096768	31727	103,23
Mtfr1l	mitochondrial fission regulator 1-like	ENSMUSG00000046671	9838	103,15
		ENSMUSG00000051510	8484	103,15
Adipor2	adiponectin receptor 2	ENSMUSG00000030168	64334	103,15
Ankle2	ankyrin repeat and LEM domain containing 2	ENSMUSG00000029501	25648	103,15
Ept1	ethanolaminephosphotransferase 1 (CDP-ethanolamine-specific)	ENSMUSG00000075703	39432	103,01
Limd2	LIM domain containing 2	ENSMUSG00000040699	4605	103,01
9530068E07Rik	RIKEN cDNA 9530068E07 gene	ENSMUSG00000036275	12301	102,94
Otud7b	OTU domain containing 7B	ENSMUSG00000038495	56603	102,94
Gpr137c	G protein-coupled receptor 137C	ENSMUSG00000049092	61512	102,94
		ENSMUSG00000031633	4488	102,86
Il17rd	interleukin 17 receptor D	ENSMUSG00000040717	68286	102,86
Cbx3	chromobox 3	ENSMUSG00000029836	13345	102,79
Nemf	nuclear export mediator factor	ENSMUSG00000020982	45633	102,79
Lpar1	lysophosphatidic acid receptor 1	ENSMUSG00000038668	118644	102,79
Rpl7a	ribosomal protein L7A	ENSMUSG00000062647	2555	102,65
Slc39a11	solute carrier family 39 (metal ion transporter), member 11	ENSMUSG00000041654	405227	102,65
Usp47	ubiquitin specific peptidase 47	ENSMUSG00000059263	87881	102,57
Rbm33	RNA binding motif protein 33	ENSMUSG00000048271	102124	102,50
Dhcr24	24-dehydrocholesterol reductase	ENSMUSG00000034926	28076	102,43
Prrc1	proline-rich coiled-coil 1	ENSMUSG00000024594	37987	102,43
Psmc4	proteasome (prosome, macropain) 26S subunit, ATPase, 4	ENSMUSG00000030603	8395	102,36
Dlgap1	discs, large (Drosophila) homolog-associated protein 1	ENSMUSG00000003279	852341	102,36
Kcmf1	potassium channel modulatory factor 1	ENSMUSG00000055239	58866	102,36
Tcf7l1	transcription factor 7 like 1 (T cell specific, HMG box)	ENSMUSG00000055799	162877	102,36
Ccdc129	coiled-coil domain containing 129	ENSMUSG00000037973	141841	102,36
		ENSMUSG00000052920	1197483	102,28
Dock11	dedicator of cytokinesis 11	ENSMUSG00000031093	187731	102,28
Msl1	male-specific lethal 1 homolog (Drosophila)	ENSMUSG00000052915	12344	102,14
Sarnp	SAP domain containing ribonucleoprotein	ENSMUSG00000078427	55868	102,14
Aldh1a7	aldehyde dehydrogenase family 1, subfamily A7	ENSMUSG00000024747	34610	102,14
Kdm6b	KDM1 lysine (K)-specific demethylase 6B	ENSMUSG00000018476	15168	102,07
Tpm4	tropomyosin 4	ENSMUSG00000031799	18168	102,07
Cdc14a	CDC14 cell division cycle 14A	ENSMUSG00000033502	151480	102,07
Dnah7b	dynein, axonemal, heavy chain 7B	ENSMUSG00000041144	306813	101,99
Hdc	histidine decarboxylase	ENSMUSG00000027360	25633	101,99
Arpc3	actin related protein 2/3 complex, subunit 3	ENSMUSG00000029465	14302	101,85
Nmt2	N-myristoyltransferase 2	ENSMUSG00000026643	44666	101,85
Col4a3bp	collagen, type IV, alpha 3 (Goodpasture antigen) binding protein	ENSMUSG00000021669	97550	101,85
Palld	palladin, cytoskeletal associated protein	ENSMUSG00000058056	389445	101,85

Yjefn3	YjeF N-terminal domain containing 3	ENSMUSG00000048967	14925	101,78
Ptpn11	protein tyrosine phosphatase, non-receptor type 11	ENSMUSG00000043733	60865	101,78
Jph1	junctophilin 1	ENSMUSG00000042686	102952	101,78
Klf3	Kruppel-like factor 3 (basic)	ENSMUSG00000029178	26607	101,70
Myeov2	myeloma overexpressed 2	ENSMUSG00000073616	4841	101,63
Hspa4	heat shock protein 4	ENSMUSG00000020361	40644	101,63
Irak1	interleukin-1 receptor-associated kinase 1	ENSMUSG00000031392	10005	101,63
Pax6	paired box gene 6	ENSMUSG00000027168	29515	101,63
Wdr6	WD repeat domain 6	ENSMUSG00000066357	6358	101,49
Rprd1a	regulation of nuclear pre-mRNA domain containing 1A	ENSMUSG00000040446	45243	101,49
Mau2	MAU2 chromatid cohesion factor homolog (C. elegans)	ENSMUSG00000031858	26612	101,49
Nceh1	neutral cholesterol ester hydrolase 1	ENSMUSG00000027698	101644	101,49
Bmpr2	bone morphogenetic protein receptor, type II (serine/threonine kinase)	ENSMUSG00000067336	106271	101,41
Ldlrad3	low density lipoprotein receptor class A domain containing 3	ENSMUSG00000048058	236183	101,34
Ddah2	dimethylarginine dimethylaminohydrolase 2	ENSMUSG00000007039	3061	101,27
Sep 15	selenoprotein	ENSMUSG00000037072	27377	101,27
Arhgap39	Rho GTPase activating protein 39	ENSMUSG00000033697	94186	101,27
Nsa2	NSA2 ribosome biogenesis homolog (S. cerevisiae)	ENSMUSG00000060739	8500	101,12
Fkbp4	FK506 binding protein 4	ENSMUSG00000030357	8943	101,12
Khdrbs3	KH domain containing, RNA binding, signal transduction associated 3	ENSMUSG00000022332	165093	101,12
Asxl1	additional sex combs like 1	ENSMUSG00000042548	58163	101,05
Vdac2	voltage-dependent anion channel 2	ENSMUSG00000021771	14611	100,98
Btbd7	BTB (POZ) domain containing 7	ENSMUSG00000041702	93751	100,98
Slc25a11	solute carrier family 25 (mitochondrial carrier oxoglutarate carrier), member 11	ENSMUSG00000014606	3284	100,91
Angel2	angel homolog 2 (Drosophila)	ENSMUSG00000026634	21851	100,83
Pbxip1	pre B cell leukemia transcription factor interacting protein 1	ENSMUSG00000042613	14247	100,83
Tjp1	tight junction protein 1	ENSMUSG00000030516	75075	100,83
Ptplad1	protein tyrosine phosphatase-like A domain containing 1	ENSMUSG00000033629	34733	100,76
Rgs3	regulator of G-protein signaling 3	ENSMUSG00000059810	143172	100,76
Rnf145	ring finger protein 145	ENSMUSG00000019189	46557	100,76
Ubr2	ubiquitin protein ligase E3 component n-recognin 2	ENSMUSG00000023977	82241	100,76
Usp6nl	USP6 N-terminal like	ENSMUSG00000039046	123724	100,76
Dalrd3	DALR anticodon binding domain containing 3	ENSMUSG00000019039	2880	100,69
Lnx1	ligand of numb-protein X 1	ENSMUSG00000029228	110466	100,69
Slc4a11	solute carrier family 4, sodium bicarbonate transporter-like, member 11	ENSMUSG00000074796	13407	100,69

Hist1h2ag	histone cluster 1, H2ag	ENSMUSG00000069301	471	100,62
Odc1	ornithine decarboxylase, structural 1	ENSMUSG00000011179	6630	100,47
		ENSMUSG00000089837	44563	100,47
Zfp605	zinc finger protein 605	ENSMUSG00000023284	19703	100,47
Rabl6	RAB, member of RAS oncogene family-like 6	ENSMUSG00000015087	25504	100,47
Ndufc2	NADH dehydrogenase (ubiquinone) 1, subcomplex unknown, 2	ENSMUSG00000030647	7800	100,33
Dmxl2	Dmx-like 2	ENSMUSG00000041268	136603	100,33
Kat6b	K(lysine) acetyltransferase 6B	ENSMUSG00000021767	191045	100,33
Acat3	acetyl-Coenzyme A acetyltransferase 3	ENSMUSG00000062480	16570	100,25
Itgb1	integrin beta 1 (fibronectin receptor beta)	ENSMUSG00000025809	47547	100,25
Qk	quaking	ENSMUSG00000062078	112891	100,25
Csk	c-src tyrosine kinase	ENSMUSG00000032312	19007	100,18
Nfs1	nitrogen fixation gene 1 (S. cerevisiae)	ENSMUSG00000027618	65258	100,18
Nbeal1	neurobeachin like 1	ENSMUSG00000073664	157730	100,11
Sec63	SEC63-like (S. cerevisiae)	ENSMUSG00000019802	71019	100,04
Ep400	E1A binding protein p400	ENSMUSG00000029505	106345	100,04
Baiap2l1	BAI1-associated protein 2-like 1	ENSMUSG00000038859	93587	100,04
Tef	thyrotroph embryonic factor	ENSMUSG00000022389	24441	99,96
Vps13d	vacuolar protein sorting 13 D (yeast)	ENSMUSG00000020220	222370	99,96
Igsf8	immunoglobulin superfamily, member 8	ENSMUSG00000038034	58201	99,89
Pknox2	Pbx/knotted 1 homeobox 2	ENSMUSG00000035934	257350	99,89
		ENSMUSG00000007041	8784	99,82
Ube4b	ubiquitination factor E4B, UFD2 homolog (S. cerevisiae)	ENSMUSG00000028960	98334	99,82
Dnm2	dynamin 2	ENSMUSG00000033335	82852	99,82
Kcnq3	potassium voltage-gated channel, subfamily Q, member 3	ENSMUSG00000056258	291054	99,82
Aamdc	adipogenesis associated Mth938 domain containing	ENSMUSG00000035642	29167	99,75
Pacrg	PARK2 co-regulated	ENSMUSG00000037196	437300	99,67
Actn1	actinin, alpha 1	ENSMUSG00000015143	92830	99,67
Enc1	ectodermal-neural cortex 1	ENSMUSG00000041773	11935	99,60
Strada	STE20-related kinase adaptor alpha	ENSMUSG00000069631	38839	99,60
Rictor	RPTOR independent companion of MTOR, complex 2	ENSMUSG00000050310	92018	99,60
Maea	macrophage erythroblast attacher	ENSMUSG00000079562	37723	99,53
Ino80	INO80 homolog (S. cerevisiae)	ENSMUSG00000034154	104646	99,46
Pdap1	PDGFA associated protein 1	ENSMUSG00000029623	11470	99,38
Fam13c	family with sequence similarity 13, member C	ENSMUSG00000043259	118064	99,38
Arl14ep	ADP-ribosylation factor-like 14 effector protein	ENSMUSG00000027122	11898	99,31
Oaz2	ornithine decarboxylase antizyme 2	ENSMUSG00000040652	22300	99,31
Rps15a	ribosomal protein S15A	ENSMUSG00000008683	11809	99,31
Selk	selenoprotein K	ENSMUSG00000042682	6767	99,24
1110004F10Rik	RIKEN cDNA 1110004F10 gene	ENSMUSG00000030663	11910	99,09

		ENSMUSG00000017286	122739	99,09
Rpl29	ribosomal protein L29	ENSMUSG00000048758	2115	99,02
Mllt6	myeloid/lymphoid or mixed-lineage leukemia (trithorax homolog, Drosophila); translocated to, 6	ENSMUSG00000038437	22050	99,02
Srsf10	serine/arginine-rich splicing factor 10	ENSMUSG00000028676	14162	99,02
Npdc1	neural proliferation, differentiation and control 1	ENSMUSG00000015094	10144	98,95
Tiam1	T cell lymphoma invasion and metastasis 1	ENSMUSG00000002489	192970	98,88
Mcmbp	MCM (minichromosome maintenance deficient) binding protein	ENSMUSG00000048170	44055	98,88
Upp2	uridine phosphorylase 2	ENSMUSG00000026839	225674	98,88
Tmem135	transmembrane protein 135	ENSMUSG00000039428	264500	98,88
		ENSMUSG00000051469	11109	98,80
E130308A19Rik	RIKEN cDNA E130308A19 gene	ENSMUSG00000045071	135229	98,73
Selenbp1	selenium binding protein 1	ENSMUSG00000068874	11703	98,73
Bzw1	basic leucine zipper and W2 domains 1	ENSMUSG00000051223	13413	98,66
Cc2d1b	coiled-coil and C2 domain containing 1B	ENSMUSG00000028582	14184	98,59
G3bp2	GTPase activating protein (SH3 domain) binding protein 2	ENSMUSG00000029405	31590	98,59
Ybx3	Y box protein 3	ENSMUSG00000030189	23593	98,51
Tacstd2	tumor-associated calcium signal transducer 2	ENSMUSG00000051397	1764	98,51
		ENSMUSG00000055044	50670	98,44
Zbtb18	zinc finger and BTB domain containing 18	ENSMUSG00000063659	6100	98,37
Bri3bp	Bri3 binding protein	ENSMUSG00000037905	19305	98,30
Casc3	cancer susceptibility candidate 3	ENSMUSG00000078676	28910	98,30
Grn	granulin	ENSMUSG00000034708	6734	98,30
Azin1	antizyme inhibitor 1	ENSMUSG00000037458	31837	98,30
Wdr77	WD repeat domain 77	ENSMUSG00000000561	25232	98,22
Ehmt1	euchromatic histone methyltransferase 1	ENSMUSG00000036893	128841	98,22
Cdkn1a	cyclin-dependent kinase inhibitor 1A (P21)	ENSMUSG00000023067	9744	98,15
Cyld	cylindromatosis (turban tumor syndrome)	ENSMUSG00000036712	54918	98,15
Rffl	ring finger and FYVE like domain containing protein	ENSMUSG00000020696	68762	97,93
Upf2	UPF2 regulator of nonsense transcripts homolog (yeast)	ENSMUSG00000043241	105235	97,93
Enox2	ecto-NOX disulfide-thiol exchanger 2	ENSMUSG00000031109	278553	97,93
Yme1l1	YME1-like 1 (S. cerevisiae)	ENSMUSG00000026775	42892	97,86
Clint1	clathrin interactor 1	ENSMUSG00000006169	58575	97,86
Csad	cysteine sulfinic acid decarboxylase	ENSMUSG00000023044	12046	97,86
		ENSMUSG00000032985	608001	97,79
Lrp1b	low density lipoprotein-related protein 1B (deleted in tumors)	ENSMUSG00000049252	2058351	97,72
Srp72	signal recognition particle 72	ENSMUSG00000036323	25255	97,57

Suclg1	succinate-CoA ligase, GDP-forming, alpha subunit	ENSMUSG00000052738	28530	97,50
Akap8	A kinase (PRKA) anchor protein 8	ENSMUSG00000024045	17478	97,50
Scg5	secretogranin V	ENSMUSG00000023236	52760	97,43
Atg12	autophagy related 12	ENSMUSG00000032905	9163	97,43
Dnajb4	DnaJ (Hsp40) homolog, subfamily B, member 4	ENSMUSG00000028035	31769	97,43
Xiap	X-linked inhibitor of apoptosis	ENSMUSG00000025860	49978	97,28
Atg10	autophagy related 10	ENSMUSG00000021619	288639	97,28
Kcnj15	potassium inwardly-rectifying channel, subfamily J, member 15	ENSMUSG00000062609	42703	97,21
Ap3b1	adaptor-related protein complex 3, beta 1 subunit	ENSMUSG00000021686	207357	97,21
Elof1	elongation factor 1 homolog (ELF1, S. cerevisiae)	ENSMUSG00000013822	4160	97,14
Myl6	myosin, light polypeptide 6, alkali, smooth muscle and non-muscle	ENSMUSG00000090841	3016	97,14
Rtn2	reticulon 2 (Z-band associated protein)	ENSMUSG00000030401	13503	97,06
Ppp2r2a	protein phosphatase 2 (formerly 2A), regulatory subunit B (PR 52), alpha isoform	ENSMUSG00000022052	58415	97,06
Cep70	centrosomal protein 70	ENSMUSG00000056267	56960	97,06
Atrn	attractin	ENSMUSG00000027312	123839	97,06
Brd8	bromodomain containing 8	ENSMUSG00000003778	25987	96,99
Dock1	dedicator of cytokinesis 1	ENSMUSG00000058325	502953	96,99
Akap2	A kinase (PRKA) anchor protein 2	ENSMUSG00000038729	179328	96,85
Dgkd	diacylglycerol kinase, delta	ENSMUSG00000070738	91203	96,77
		ENSMUSG00000020457	79056	96,77
Rpl17	ribosomal protein L17	ENSMUSG00000062328	2905	96,63
Rab14	RAB14, member RAS oncogene family	ENSMUSG00000026878	20916	96,63
Pcdh19	protocadherin 19	ENSMUSG00000051323	106131	96,56
Nop56	NOP56 ribonucleoprotein	ENSMUSG00000027405	4884	96,48
Stx3	syntaxin 3	ENSMUSG00000041488	44285	96,48
Hist1h1c	histone cluster 1, H1c	ENSMUSG00000036181	1560	96,41
Neo1	neogenin	ENSMUSG00000032340	161763	96,41
Ctnnal1	catenin (cadherin associated protein), alpha-like 1	ENSMUSG00000038816	54254	96,41
Epc1	enhancer of polycomb homolog 1 (Drosophila)	ENSMUSG00000024240	80158	96,34
Zfp467	zinc finger protein 467	ENSMUSG00000068551	18129	96,27
Mtch1	mitochondrial carrier homolog 1 (C. elegans)	ENSMUSG00000024012	15833	96,12
		ENSMUSG00000030706	62547	96,12
Pik3r4	phosphatidylinositol 3 kinase, regulatory subunit, polypeptide 4, p150	ENSMUSG00000032571	44661	95,98
Dennd5b	DENN/MADD domain containing 5B	ENSMUSG00000030313	113610	95,98
		ENSMUSG00000054934	56018	95,90
Spata13	spermatogenesis associated 13	ENSMUSG00000021990	130556	95,90
Efnb2	ephrin B2	ENSMUSG00000001300	43340	95,90
Mex3c	mex3 homolog C (C. elegans)	ENSMUSG00000037253	19532	95,83
Wdr70	WD repeat domain 70	ENSMUSG00000039828	226155	95,83
Dctn4	dynactin 4	ENSMUSG00000024603	32542	95,83

Fam83e	family with sequence similarity 83, member E	ENSMUSG00000054161	8281	95,83
Gfod1	glucose-fructose oxidoreductase domain containing 1	ENSMUSG00000051335	108654	95,76
Eif4h	eukaryotic translation initiation factor 4H	ENSMUSG00000040731	19469	95,69
Chmp4b	charged multivesicular body protein 4B	ENSMUSG00000038467	43081	95,69
Arhgap12	Rho GTPase activating protein 12	ENSMUSG00000041225	111672	95,69
Mapk12	mitogen-activated protein kinase 12	ENSMUSG00000022610	10119	95,69
Pds5a	PDS5, regulator of cohesion maintenance, homolog A (S. cerevisiae)	ENSMUSG00000029202	82597	95,69
		ENSMUSG00000092470	56395	95,61
Slx4ip	SLX4 interacting protein	ENSMUSG00000027281	180733	95,61
Spast	spastin	ENSMUSG00000024068	52127	95,54
		ENSMUSG00000032020	150600	95,54
Mid1	midline 1	ENSMUSG00000035299	320538	95,54
Calml4	calmodulin-like 4	ENSMUSG00000032246	17815	95,47
Kat2b	K(lysine) acetyltransferase 2B	ENSMUSG00000000708	105860	95,47
Snap25	synaptosomal-associated protein 25	ENSMUSG00000027273	68976	95,40
Plk2	polo-like kinase 2	ENSMUSG00000021701	5801	95,40
Agbl3	ATP/GTP binding protein-like 3	ENSMUSG00000038836	79028	95,32
Ipcef1	interaction protein for cytohesin exchange factors 1	ENSMUSG00000064065	166683	95,32
Rnf19a	ring finger protein 19A	ENSMUSG00000022280	43214	95,32
Add1	adducin 1 (alpha)	ENSMUSG00000029106	58645	95,25
Mast2	microtubule associated serine/threonine kinase 2	ENSMUSG00000003810	157422	95,25
Gtl3	gene trap locus 3	ENSMUSG00000031796	14618	95,18
Ier3	immediate early response 3	ENSMUSG00000003541	1240	95,18
Zkscan1	zinc finger with KRAB and SCAN domains 1	ENSMUSG00000029729	22739	95,11
Kif21a	kinesin family member 21A	ENSMUSG00000022629	116673	95,11
Cd63	CD63 antigen	ENSMUSG00000025351	3898	95,03
Shprh	SNF2 histone linker PHD RING helicase	ENSMUSG00000090112	68169	95,03
Tnrc18	trinucleotide repeat containing 18	ENSMUSG00000039477	67283	95,03
Glo1	glyoxalase 1	ENSMUSG00000024026	19794	94,89
Ip6k1	inositol hexaphosphate kinase 1	ENSMUSG00000032594	46280	94,89
S100a11	S100 calcium binding protein A11 (calgizzarin)	ENSMUSG00000027907	5800	94,89
Uhrf2	ubiquitin-like, containing PHD and RING finger domains 2	ENSMUSG00000024817	63210	94,89
Abca3	ATP-binding cassette, sub-family A (ABC1), member 3	ENSMUSG00000024130	58252	94,82
Dennd5a	DENN/MADD domain containing 5A	ENSMUSG00000035901	66691	94,82
Hivep1	human immunodeficiency virus type I enhancer binding protein 1	ENSMUSG00000021366	133006	94,82
Pdlim3	PDZ and LIM domain 3	ENSMUSG00000031636	34062	94,82
Cblb	Casitas B-lineage lymphoma b	ENSMUSG00000022637	176499	94,67
Phkb	phosphorylase kinase beta	ENSMUSG00000036879	220418	94,67

Cops4	COP9 (constitutive photomorphogenic) homolog, subunit 4 (Arabidopsis thaliana)	ENSMUSG00000035297	29495	94,60
Cdyl2	chromodomain protein, Y chromosome-like 2	ENSMUSG00000031758	164268	94,60
Avl9	AVL9 homolog (S. cerevisiase)	ENSMUSG00000029787	46985	94,60
Mbtps1	membrane-bound transcription factor peptidase, site 1	ENSMUSG00000031835	50606	94,53
Eme1	essential meiotic endonuclease 1 homolog 1 (S. pombe)	ENSMUSG00000039055	8969	94,45
Vrk3	vaccinia related kinase 3	ENSMUSG00000002205	29103	94,45
Abcd2	ATP-binding cassette, sub-family D (ALD), member 2	ENSMUSG00000055782	45924	94,45
Plxnb2	plexin B2	ENSMUSG00000036606	15572	94,45
Mppe1	metallophosphoesterase 1	ENSMUSG00000062526	20301	94,38
Park7	Parkinson disease (autosomal recessive, early onset) 7	ENSMUSG00000028964	17305	94,38
Pou2f3	POU domain, class 2, transcription factor 3	ENSMUSG00000032015	87742	94,38
Nuak2	NUAK family, SNF1-like kinase, 2	ENSMUSG00000009772	17363	94,31
Setd7	SET domain containing (lysine methyltransferase) 7	ENSMUSG00000037111	45560	94,31
Ttll5	tubulin tyrosine ligase-like family, member 5	ENSMUSG00000012609	237235	94,31
Ilf3	interleukin enhancer binding factor 3	ENSMUSG00000032178	37343	94,24
Lap3	leucine aminopeptidase 3	ENSMUSG00000039682	19301	94,24
		ENSMUSG00000026384	179570	94,24
Tollip	toll interacting protein	ENSMUSG00000025139	43695	94,16
Eif1b	eukaryotic translation initiation factor 1B	ENSMUSG00000006941	2722	94,16
Fndc3a	fibronectin type III domain containing 3A	ENSMUSG00000033487	172058	94,16
Ncald	neurocalcin delta	ENSMUSG00000051359	426396	94,16
		ENSMUSG00000026361	104097	94,09
Klhdc2	kelch domain containing 2	ENSMUSG00000020978	14007	94,02
Igsf3	immunoglobulin superfamily, member 3	ENSMUSG00000042035	85936	94,02
Foxo6	forkhead box O6	ENSMUSG00000052135	20271	93,95
Wbp11	WW domain binding protein 11	ENSMUSG00000030216	14580	93,95
Zfp821	zinc finger protein 821	ENSMUSG00000031728	19384	93,95
Mtch2	mitochondrial carrier homolog 2 (C. elegans)	ENSMUSG00000027282	19656	93,80
		ENSMUSG00000009555	7293	93,73
Fam193b	family with sequence similarity 193, member B	ENSMUSG00000021495	31802	93,73
Cant1	calcium activated nucleotidase 1	ENSMUSG00000025575	12798	93,66
Srpr	signal recognition particle receptor ('docking protein')	ENSMUSG00000032042	47799	93,66
Kif13a	kinesin family member 13A	ENSMUSG00000021375	180632	93,66
		ENSMUSG00000007989	61438	93,66
Ncbp2	nuclear cap binding protein subunit 2	ENSMUSG00000022774	13269	93,58
Trim41	tripartite motif-containing 41	ENSMUSG00000040365	10950	93,58
Kdm6a	lysine (K)-specific demethylase 6A	ENSMUSG00000037369	117362	93,58
Man2a2	mannosidase 2, alpha 2	ENSMUSG00000038886	22278	93,51

C2cd2l	C2 calcium-dependent domain containing 2-like	ENSMUSG00000032120	11046	93,44
Sfi1	Sfi1 homolog, spindle assembly associated (yeast)	ENSMUSG00000023764	61614	93,44
Usp4	ubiquitin specific peptidase 4 (proto-oncogene)	ENSMUSG00000032612	44699	93,37
Gxylt1	glucoside xylosyltransferase 1	ENSMUSG00000036197	35420	93,37
Creb1	cAMP responsive element binding protein 1	ENSMUSG00000025958	71744	93,37
Adam22	a disintegrin and metallopeptidase domain 22	ENSMUSG00000040537	295809	93,22
Ap1s3	adaptor-related protein complex AP-1, sigma 3	ENSMUSG00000054702	65097	93,22
Tmtc1	transmembrane and tetratricopeptide repeat containing 1	ENSMUSG00000030306	211960	93,22
		ENSMUSG00000041112	517847	93,22
Cpq	carboxypeptidase Q	ENSMUSG00000039007	511424	93,22
Cep128	centrosomal protein 128	ENSMUSG00000061533	385918	93,22
Psma4	proteasome (prosome, macropain) subunit, alpha type 4	ENSMUSG00000032301	7241	93,15
		ENSMUSG00000025262	127668	93,15
Umodl1	uromodulin-like 1	ENSMUSG00000054134	56030	93,08
Polr2c	polymerase (RNA) II (DNA directed) polypeptide C	ENSMUSG00000031783	6793	93,08
Mecp2	methyl CpG binding protein 2	ENSMUSG00000031393	108772	93,08
		ENSMUSG00000031626	320119	93,08
Trrap	transformation/transcription domain-associated protein	ENSMUSG00000045482	91687	93,08
Kpna3	karyopherin (importin) alpha 3	ENSMUSG00000021929	74762	93,01
Rnf38	ring finger protein 38	ENSMUSG00000035696	107580	93,01
Slc37a3	solute carrier family 37 (glycerol-3-phosphate transporter), member 3	ENSMUSG00000029924	42938	92,93
Ikbkap	inhibitor of kappa light polypeptide enhancer in B cells, kinase complex-associated protein	ENSMUSG00000028431	52652	92,93
		ENSMUSG00000001419	29715	92,93
Actr10	ARP10 actin-related protein 10	ENSMUSG00000021076	26862	92,86
Dtnb	dystrobrevin, beta	ENSMUSG00000071454	209416	92,86
Prpf38b	PRP38 pre-mRNA processing factor 38 (yeast) domain containing B	ENSMUSG00000027881	9061	92,72
Grik3	glutamate receptor, ionotropic, kainate 3	ENSMUSG00000001985	223474	92,72
Wdfy2	WD repeat and FYVE domain containing 2	ENSMUSG00000014547	119197	92,72
Timm22	translocase of inner mitochondrial membrane 22	ENSMUSG00000020843	9341	92,57
Fam107b	family with sequence similarity 107, member B	ENSMUSG00000026655	168385	92,57
Uggt1	UDP-glucose glycoprotein glucosyltransferase 1	ENSMUSG00000037470	102776	92,57
Lrch1	leucine-rich repeats and calponin homology (CH) domain containing 1	ENSMUSG00000068015	193204	92,57
		ENSMUSG00000023904	1632	92,50

Aars	alanyl-tRNA synthetase	ENSMUSG00000031960	24521	92,50
Polr1d	polymerase (RNA) I polypeptide D	ENSMUSG00000029642	34312	92,50
Tulp2	tubby-like protein 2	ENSMUSG00000023467	37371	92,50
Samd14	sterile alpha motif domain containing 14	ENSMUSG00000047181	16209	92,43
Ppp1r12b	protein phosphatase 1, regulatory (inhibitor) subunit 12B	ENSMUSG00000073557	201285	92,43
Ppp2r3a	protein phosphatase 2, regulatory subunit B'', alpha	ENSMUSG00000043154	146841	92,43
		ENSMUSG00000090053	277770	92,43
		ENSMUSG00000036698	86249	92,35
Pdxdc1	pyridoxal-dependent decarboxylase domain containing 1	ENSMUSG00000022680	69984	92,35
Anapc11	anaphase promoting complex subunit 11	ENSMUSG00000025135	9778	92,28
Clock	circadian locomotor output cycles kaput	ENSMUSG00000029238	92372	92,28
Rhoa	ras homolog gene family, member A	ENSMUSG00000007815	31735	92,21
Clgn	calmegin	ENSMUSG00000002190	38686	92,14
		ENSMUSG00000096403	1602	92,14
Suds3	suppressor of defective silencing 3 homolog (S. cerevisiae)	ENSMUSG00000066900	24312	92,14
Use1	unconventional SNARE in the ER 1 homolog (S. cerevisiae)	ENSMUSG00000002395	2885	92,14
Spef1	sperm flagellar 1	ENSMUSG00000027329	17022	92,06
Tmem127	transmembrane protein 127	ENSMUSG00000034850	13200	92,06
Sgpl1	sphingosine phosphate lyase 1	ENSMUSG00000020097	49062	92,06
Trove2	TROVE domain family, member 2	ENSMUSG00000018199	26279	91,99
Tes	testis derived transcript	ENSMUSG00000029552	40680	91,99
Cit	citron	ENSMUSG00000029516	163670	91,99
Rtp1	receptor transporter protein 1	ENSMUSG00000033383	4828	91,92
Usp24	ubiquitin specific peptidase 24	ENSMUSG00000028514	125110	91,92
Anapc5	anaphase-promoting complex subunit 5	ENSMUSG00000029472	33871	91,85
March8	membrane-associated ring finger (C3HC4) 8	ENSMUSG00000025702	71517	91,85
Nup98	nucleoporin 98	ENSMUSG00000063550	75917	91,85
Usp32	ubiquitin specific peptidase 32	ENSMUSG00000000804	155720	91,77
Mfap1a	microfibrillar-associated protein 1A	ENSMUSG00000068479	14755	91,70
Gm21685	predicted gene, 21685	ENSMUSG00000022066	30097	91,70
Hist1h2ac	histone cluster 1, H2ac	ENSMUSG00000069270	2482	91,63
Psmd11	proteasome (prosome, macropain) 26S subunit, non-ATPase, 11	ENSMUSG00000017428	44634	91,63
Rgs12	regulator of G-protein signaling 12	ENSMUSG00000029101	90200	91,63
Ccny	cyclin Y	ENSMUSG00000024286	136107	91,63
Ergic1	endoplasmic reticulum-golgi intermediate compartment (ERGIC) 1	ENSMUSG00000001576	95443	91,56
Zmat2	zinc finger, matrin type 2	ENSMUSG00000001383	5738	91,48
Zfand6	zinc finger, AN1-type domain 6	ENSMUSG00000030629	64308	91,48
Cd82	CD82 antigen	ENSMUSG00000027215	44030	91,48
Far1	fatty acyl CoA reductase 1	ENSMUSG00000030759	57678	91,41
Sfrp1	secreted frizzled-related protein 1	ENSMUSG00000031548	38131	91,41
		ENSMUSG00000042225	115583	91,34
Nhlrc2	NHL repeat containing 2	ENSMUSG00000025078	50586	91,34

Dhdh	dihydrodiol dehydrogenase (dimeric)	ENSMUSG00000011382	15850	91,34
Kdelr1	KDEL (Lys-Asp-Glu-Leu) endoplasmic reticulum protein retention receptor 1	ENSMUSG00000002778	10960	91,19
Megf8	multiple EGF-like-domains 8	ENSMUSG00000045039	48754	91,19
Gsk3a	glycogen synthase kinase 3 alpha	ENSMUSG00000057177	9594	91,12
Ankfy1	ankyrin repeat and FYVE domain containing 1	ENSMUSG00000020790	82141	91,12
Rasa2	RAS p21 protein activator 2	ENSMUSG00000032413	92318	91,12
Acvr2a	activin receptor IIA	ENSMUSG00000052155	89161	91,12
Fggy	FGGY carbohydrate kinase domain containing	ENSMUSG00000028573	369433	91,12
Carhsp1	calcium regulated heat stable protein 1	ENSMUSG00000008393	13567	91,05
Aes	amino-terminal enhancer of split	ENSMUSG00000054452	6869	91,05
Agpat4	1-acylglycerol-3-phosphate O-acyltransferase 4 (lysophosphatidic acid acyltransferase, delta)	ENSMUSG00000023827	100942	91,05
Cbx6	chromobox 6	ENSMUSG00000089715	10793	91,05
Lman2	lectin, mannose-binding 2	ENSMUSG00000021484	18951	91,05
Topors	topoisomerase I binding, arginine/serine-rich	ENSMUSG00000036822	10250	91,05
Snx27	sorting nexin family member 27	ENSMUSG00000028136	85173	91,05
Mthfd1	methylenetetrahydrofolate dehydrogenase (NADP+ dependent), methenyltetrahydrofolate cyclohydrolase, formyltetrahydrofolate synthase	ENSMUSG00000021048	64589	91,05
Rai1	retinoic acid induced 1	ENSMUSG00000062115	94185	91,05
Ddhd2	DDHD domain containing 2	ENSMUSG00000061313	28957	90,98
Acbd5	acyl-Coenzyme A binding domain containing 5	ENSMUSG00000026781	46343	90,90
Cpne8	copine VIII	ENSMUSG00000052560	191951	90,90
Cbx5	chromobox 5	ENSMUSG00000009575	48273	90,76
Zc3hav1	zinc finger CCCH type, antiviral 1	ENSMUSG00000029826	49318	90,76
Ddost	dolichyl-di-phosphooligosaccharide-protein glycotransferase	ENSMUSG00000028757	7899	90,69
Ilf2	interleukin enhancer binding factor 2	ENSMUSG00000001016	12179	90,69
Ganab	alpha glucosidase 2 alpha neutral subunit	ENSMUSG00000071650	18574	90,69
Nr1d2	nuclear receptor subfamily 1, group D, member 2	ENSMUSG00000021775	35074	90,69
		ENSMUSG00000015120	12707	90,61
Dgcr2	DiGeorge syndrome critical region gene 2	ENSMUSG00000003166	54664	90,61
Taf1	TAF1 RNA polymerase II, TATA box binding protein (TBP)-associated factor	ENSMUSG00000031314	69056	90,61
Abl2	v-abl Abelson murine leukemia viral oncogene 2 (arg, Abelson-related gene)	ENSMUSG00000026596	90783	90,61
Numb	numb gene homolog (Drosophila)	ENSMUSG00000021224	127901	90,61
		ENSMUSG00000019124	60600	90,54

		ENSMUSG00000019795	51764	90,54
Ppp2r1a	protein phosphatase 2 (formerly 2A), regulatory subunit A (PR 65), alpha isoform	ENSMUSG00000007564	20606	90,54
Usp37	ubiquitin specific peptidase 37	ENSMUSG00000033364	108777	90,54
Stom	stomatin	ENSMUSG00000026880	22991	90,47
Chfr	checkpoint with forkhead and ring finger domains	ENSMUSG00000014668	36133	90,47
Cd99l2	CD99 antigen-like 2	ENSMUSG00000035776	72790	90,47
Hmgn2	high mobility group nucleosomal binding domain 2	ENSMUSG00000003038	3913	90,40
Scamp1	secretory carrier membrane protein 1	ENSMUSG00000021687	84548	90,40
Dock4	dedicator of cytokinesis 4	ENSMUSG00000035954	400434	90,40
		ENSMUSG00000000568	20513	90,32
Zc3h15	zinc finger CCCH-type containing 15	ENSMUSG00000027091	20188	90,25
Ireb2	iron responsive element binding protein 2	ENSMUSG00000032293	48780	90,25
Jmy	junction-mediating and regulatory protein	ENSMUSG00000021690	69712	90,25
		ENSMUSG00000042616	33631	90,18
Sun1	Sad1 and UNC84 domain containing 1	ENSMUSG00000036817	49204	90,18
Arpp19	cAMP-regulated phosphoprotein 19	ENSMUSG00000007656	22700	90,18
Fam178a	family with sequence similarity 178, member A	ENSMUSG00000036097	52669	90,18
Slc20a2	solute carrier family 20, member 2	ENSMUSG00000037656	92908	90,18
		ENSMUSG00000036452	774751	90,18
Mslnl	mesothelin-like	ENSMUSG00000041062	12291	90,11
Nek9	NIMA (never in mitosis gene a)-related expressed kinase 9	ENSMUSG00000034290	39849	90,11
Pcmtd1	protein-L-isoaspartate (D-aspartate) O-methyltransferase domain containing 1	ENSMUSG00000051285	84709	90,11
2700054A10Rik	RIKEN cDNA 2700054A10 gene	ENSMUSG00000038347	279273	90,11
Slc25a23	solute carrier family 25 (mitochondrial carrier; phosphate carrier), member 23	ENSMUSG00000046329	16153	89,96
Cdc5l	cell division cycle 5-like (S. pombe)	ENSMUSG00000023932	41816	89,96
Ube2n	ubiquitin-conjugating enzyme E2N	ENSMUSG00000074781	30497	89,96
Naa15	N(alpha)-acetyltransferase 15, NatA auxiliary subunit	ENSMUSG00000063273	59970	89,96
		ENSMUSG00000028126	48401	89,89
Specc1	sperm antigen with calponin homology and coiled-coil domains 1	ENSMUSG00000042331	266235	89,89
Dnajb11	DnaJ (Hsp40) homolog, subfamily B, member 11	ENSMUSG00000004460	21790	89,82
		ENSMUSG00000048100	11525	89,82
Hivep3	human immunodeficiency virus type I enhancer binding protein 3	ENSMUSG00000028634	404262	89,82
Urod	uroporphyrinogen decarboxylase	ENSMUSG00000028684	4449	89,74
Fap	fibroblast activation protein	ENSMUSG00000000392	73133	89,74
Hmga1-rs1	high mobility group AT-hook I, related sequence 1	ENSMUSG00000078249	1599	89,67

Cnot6l	CCR4-NOT transcription complex, subunit 6-like	ENSMUSG00000034724	93839	89,67
Ypel2	yippee-like 2 (Drosophila)	ENSMUSG00000018427	57283	89,67
Map2k5	mitogen-activated protein kinase kinase 5	ENSMUSG00000058444	214134	89,67
Nrp2	neuropilin 2	ENSMUSG00000025969	115411	89,60
Pik3ca	phosphatidylinositol 3-kinase, catalytic, alpha polypeptide	ENSMUSG00000027665	70816	89,60
Brinp2	bone morphogenic protein/retinoic acid inducible neural-specific 2	ENSMUSG00000004031	110990	89,53
Raly	hnRNP-associated with lethal yellow	ENSMUSG00000027593	76166	89,53
Ppp1r13b	protein phosphatase 1, regulatory (inhibitor) subunit 13B	ENSMUSG00000021285	79598	89,45
Hmga1	high mobility group AT-hook 1	ENSMUSG00000046711	7055	89,38
Rsbn1	rosbin, round spermatid basic protein 1	ENSMUSG00000044098	52517	89,38
Gsta4	glutathione S-transferase, alpha 4	ENSMUSG00000032348	17383	89,38
Nenf	neuron derived neurotrophic factor	ENSMUSG00000037499	11406	89,31
Ctbp1	C-terminal binding protein 1	ENSMUSG00000037373	27281	89,31
Ccdc6	coiled-coil domain containing 6	ENSMUSG00000048701	96080	89,31
Irf2	interferon regulatory factor 2	ENSMUSG00000031627	107714	89,31
Coch	coagulation factor C homolog (Limulus polyphemus)	ENSMUSG00000020953	12433	89,24
Epo	erythropoietin	ENSMUSG00000029711	50984	89,24
Zwint	ZW10 interactor	ENSMUSG00000019923	20120	89,24
Ebf4	early B cell factor 4	ENSMUSG00000053552	75313	89,24
Fbxo45	F-box protein 45	ENSMUSG00000035764	17047	89,09
		ENSMUSG00000003418	142789	89,09
		ENSMUSG00000092232	14697	88,95
Arid3a	AT rich interactive domain 3A (BRIGHT-like)	ENSMUSG00000019564	27976	88,95
Cul1	cullin 1	ENSMUSG00000029686	72742	88,95
Cd2ap	CD2-associated protein	ENSMUSG00000061665	83474	88,95
Pcnp	PEST proteolytic signal containing nuclear protein	ENSMUSG00000071533	22495	88,87
Scn3a	sodium channel, voltage-gated, type III, alpha	ENSMUSG00000057182	110510	88,80
Arcn1	archain 1	ENSMUSG00000032096	26282	88,73
Pigs	phosphatidylinositol glycan anchor biosynthesis, class S	ENSMUSG00000041958	14368	88,73
Sh3glb1	SH3-domain GRB2-like B1 (endophilin)	ENSMUSG00000037062	31480	88,73
Pank3	pantothenate kinase 3	ENSMUSG00000018846	21802	88,66
Patz1	POZ (BTB) and AT hook containing zinc finger 1	ENSMUSG00000020453	20210	88,66
Kdsr	3-ketodihydrosphingosine reductase	ENSMUSG00000009905	39333	88,66
Cep112	centrosomal protein 112	ENSMUSG00000020728	435424	88,66
Spen	SPEN homolog, transcriptional regulator (Drosophila)	ENSMUSG00000040761	70708	88,58
Ptprt	protein tyrosine phosphatase, receptor type, T	ENSMUSG00000053141	1139158	88,44
		ENSMUSG00000027002	80869	88,37
Rnf14	ring finger protein 14	ENSMUSG00000060450	21213	88,29

Sec22a	SEC22 vesicle trafficking protein homolog A (S. cerevisiae)	ENSMUSG00000034473	52788	88,29
Uap1	UDP-N-acetylglucosamine pyrophosphorylase 1	ENSMUSG00000026670	33020	88,22
Stau2	staufen (RNA binding protein) homolog 2 (Drosophila)	ENSMUSG00000025920	291439	88,22
		ENSMUSG00000027236	28053	88,22
Tmem164	transmembrane protein 164	ENSMUSG00000047045	162089	88,22
Asxl3	additional sex combs like 3 (Drosophila)	ENSMUSG00000045215	185345	88,15
Rfng	RFNG O-fucosylpeptide 3-beta-N-acetylglucosaminyltransferase	ENSMUSG00000025158	3462	88,08
Ppfia1	protein tyrosine phosphatase, receptor type, f polypeptide (PTPRF), interacting protein (liprin), alpha 1	ENSMUSG00000037519	76972	88,08
Xpo1	exportin 1, CRM1 homolog (yeast)	ENSMUSG00000020290	42209	88,08
Galnt18	UDP-N-acetyl-alpha-D-galactosamine:polypeptide N-acetylgalactosaminyltransferase 18	ENSMUSG00000038296	308317	88,08
Psma2	proteasome (prosome, macropain) subunit, alpha type 2	ENSMUSG00000015671	12430	88,00
Ythdf1	YTH domain family 1	ENSMUSG00000038848	16573	88,00
		ENSMUSG00000068798	74064	88,00
Spint1	serine protease inhibitor, Kunitz type 1	ENSMUSG00000027315	12166	88,00
Znrf3	zinc and ring finger 3	ENSMUSG00000041961	168524	88,00
Cab39l	calcium binding protein 39-like	ENSMUSG00000021981	107923	87,93
Midn	midnolin	ENSMUSG00000035621	10097	87,86
Prune2	prune homolog 2 (Drosophila)	ENSMUSG00000039126	267815	87,86
Myh10	myosin, heavy polypeptide 10, non-muscle	ENSMUSG00000020900	125074	87,86
Eps15	epidermal growth factor receptor pathway substrate 15	ENSMUSG00000028552	107550	87,86
Ctxn1	cortexin 1	ENSMUSG00000048644	1627	87,79
		ENSMUSG00000025909	938404	87,64
Adcy2	adenylate cyclase 2	ENSMUSG00000021536	379499	87,64
Mdh2	malate dehydrogenase 2, NAD (mitochondrial)	ENSMUSG00000019179	11919	87,57
Ndufa13	NADH dehydrogenase (ubiquinone) 1 alpha subcomplex, 13	ENSMUSG00000036199	8371	87,57
Mtf2	metal response element binding transcription factor 2	ENSMUSG00000029267	43331	87,57
Etnk1	ethanolamine kinase 1	ENSMUSG00000030275	41313	87,57
		ENSMUSG00000021596	647276	87,50
Dhx15	DEAH (Asp-Glu-Ala-His) box polypeptide 15	ENSMUSG00000029169	40311	87,50
Lrrc4c	leucine rich repeat containing 4C	ENSMUSG00000050587	1313498	87,50
		ENSMUSG00000027465	20169	87,35
Xpa	xeroderma pigmentosum, complementation group A	ENSMUSG00000028329	40965	87,35
Ube2w	ubiquitin-conjugating enzyme E2W (putative)	ENSMUSG00000025939	78700	87,35
Wdr96	WD repeat domain 96	ENSMUSG00000044948	181727	87,35
Rpl6	ribosomal protein L6	ENSMUSG00000029614	4761	87,28
		ENSMUSG00000078193	445	87,28

		ENSMUSG00000057286	19566	87,21
Csrp2	cysteine and glycine-rich protein 2	ENSMUSG00000020186	19423	87,21
Psmb5	proteasome (prosome, macropain) subunit, beta type 5	ENSMUSG00000022193	3876	87,13
Wnk2	WNK lysine deficient protein kinase 2	ENSMUSG00000037989	111712	87,13
Zmym5	zinc finger, MYM-type 5	ENSMUSG00000040123	21132	87,06
Reps1	RalBP1 associated Eps domain containing protein	ENSMUSG00000019854	69295	87,06
Camkk2	calcium/calmodulin-dependent protein kinase kinase 2, beta	ENSMUSG00000029471	48238	86,99
Dpy30	dpy-30 homolog (C. elegans)	ENSMUSG00000024067	24471	86,99
Faf2	Fas associated factor family member 2	ENSMUSG00000025873	42285	86,84
Atp6v1f	ATPase, H+ transporting, lysosomal V1 subunit F	ENSMUSG00000004285	2795	86,84
Tmem183a	transmembrane protein 183A	ENSMUSG00000042305	15903	86,84
Dlg3	discs, large homolog 3 (Drosophila)	ENSMUSG00000000881	50689	86,84
Mon2	MON2 homolog (yeast)	ENSMUSG00000034602	84445	86,77
Chn1	chimerin (chimaerin) 1	ENSMUSG00000056486	164687	86,77
Utrn	utrophin	ENSMUSG00000019820	479548	86,77
		ENSMUSG00000090996	29794	86,70
Atp6ap1	ATPase, H+ transporting, lysosomal accessory protein 1	ENSMUSG00000019087	7625	86,55
Sfr1	SWI5 dependent recombination repair 1	ENSMUSG00000025066	3833	86,55
Atp6v1e1	ATPase, H+ transporting, lysosomal V1 subunit E1	ENSMUSG00000019210	27441	86,55
Acsm4	acyl-CoA synthetase medium-chain family member 4	ENSMUSG00000047026	24542	86,55
Sema4b	sema domain, immunoglobulin domain (Ig), transmembrane domain (TM) and short cytoplasmic domain, (semaphorin) 4B	ENSMUSG00000030539	39554	86,55
		ENSMUSG00000027879	22087	86,48
Peli1	pellino 1	ENSMUSG00000020134	59033	86,48
Arl2bp	ADP-ribosylation factor-like 2 binding protein	ENSMUSG00000031776	7663	86,41
Stau1	staufen (RNA binding protein) homolog 1 (Drosophila)	ENSMUSG00000039536	48751	86,41
Kif26b	kinesin family member 26B	ENSMUSG00000026494	403731	86,41
Rab40c	Rab40c, member RAS oncogene family	ENSMUSG00000025730	37614	86,34
Pds5b	PDS5, regulator of cohesion maintenance, homolog B (S. cerevisiae)	ENSMUSG00000034021	136952	86,34
Tmem59	transmembrane protein 59	ENSMUSG00000028618	22598	86,26
Zbtb44	zinc finger and BTB domain containing 44	ENSMUSG00000047412	45236	86,26
Cdh4	cadherin 4	ENSMUSG00000000305	456943	86,19
Csnk1g3	casein kinase 1, gamma 3	ENSMUSG00000073563	93572	86,19
Eif5a	eukaryotic translation initiation factor 5A	ENSMUSG00000078812	5245	86,12
Ugp2	UDP-glucose pyrophosphorylase 2	ENSMUSG00000001891	50064	86,12
		ENSMUSG00000026014	83914	86,12

Dcaf5	DDB1 and CUL4 associated factor 5	ENSMUSG00000049106	100755	86,12
Kdm5c	lysine (K)-specific demethylase 5C	ENSMUSG00000025332	41516	86,12
Ccdc157	coiled-coil domain containing 157	ENSMUSG00000051427	19171	85,97
Ndufa7	NADH dehydrogenase (ubiquinone) 1 alpha subcomplex, 7 (B14.5a)	ENSMUSG00000041881	13726	85,90
Hmgn5	high-mobility group nucleosome binding domain 5	ENSMUSG00000031245	8871	85,90
Ssx2ip	synovial sarcoma, X breakpoint 2 interacting protein	ENSMUSG00000036825	35498	85,90
Dcaf17	DDB1 and CUL4 associated factor 17	ENSMUSG00000041966	43815	85,90
Gm11149	predicted gene 11149	ENSMUSG00000079564	44609	85,90
Atxn7l1	ataxin 7-like 1	ENSMUSG00000020564	227967	85,90
Trib2	tribbles homolog 2 (Drosophila)	ENSMUSG00000020601	25151	85,83
Clec16a	C-type lectin domain family 16, member A	ENSMUSG00000068663	199540	85,83
Trerf1	transcriptional regulating factor 1	ENSMUSG00000064043	218517	85,83
Ranbp9	RAN binding protein 9	ENSMUSG00000038546	78301	85,83
Uqcr10	ubiquinol-cytochrome c reductase, complex III subunit X	ENSMUSG00000059534	2370	85,76
Epm2aip1	EPM2A (laforin) interacting protein 1	ENSMUSG00000046785	7245	85,76
Bre	brain and reproductive organ-expressed protein	ENSMUSG00000052139	387279	85,76
Naca	nascent polypeptide-associated complex alpha polypeptide	ENSMUSG00000061315	13292	85,68
Fam168b	family with sequence similarity 168, member B	ENSMUSG00000037503	29847	85,68
Pitpnb	phosphatidylinositol transfer protein, beta	ENSMUSG00000050017	57597	85,61
Marc2	mitochondrial amidoxime reducing component 2	ENSMUSG00000073481	33384	85,61
Micu1	mitochondrial calcium uptake 1	ENSMUSG00000020111	161656	85,61
Hunk	hormonally upregulated Neu-associated kinase	ENSMUSG00000053414	113157	85,54
Gna13	guanine nucleotide binding protein, alpha 13	ENSMUSG00000020611	38539	85,47
Acap2	ArfGAP with coiled-coil, ankyrin repeat and PH domains 2	ENSMUSG00000049076	108826	85,47
Csnk2b	casein kinase 2, beta polypeptide	ENSMUSG00000024387	5858	85,39
		ENSMUSG00000024725	52462	85,32
Ildr1	immunoglobulin-like domain containing receptor 1	ENSMUSG00000022900	32827	85,32
Med28	mediator of RNA polymerase II transcription, subunit 28 homolog (yeast)	ENSMUSG00000015804	9056	85,25
		ENSMUSG00000057388	4997	85,25
Prkar2a	protein kinase, cAMP dependent regulatory, type II alpha	ENSMUSG00000032601	57370	85,25
Fam73a	family with sequence similarity 73, member A	ENSMUSG00000054942	66949	85,25
Abhd15	abhydrolase domain containing 15	ENSMUSG00000000686	23487	85,25
Gpr158	G protein-coupled receptor 158	ENSMUSG00000045967	463006	85,25
Prkaca	protein kinase, cAMP dependent, catalytic, alpha	ENSMUSG00000005469	23468	85,18

		ENSMUSG00000012422	54785	85,18
		ENSMUSG00000053935	2012	85,18
Traf3	TNF receptor-associated factor 3	ENSMUSG00000021277	100784	85,18
Rreb1	ras responsive element binding protein 1	ENSMUSG00000039087	173603	85,18
Pifo	primary cilia formation	ENSMUSG00000010136	17690	85,18
Btf3	basic transcription factor 3	ENSMUSG00000021660	7111	85,03
Frmd3	FERM domain containing 3	ENSMUSG00000049122	188773	85,03
Ppt1	palmitoyl-protein thioesterase 1	ENSMUSG00000028657	22934	84,96
		ENSMUSG00000032263	199090	84,96
Osbpl5	oxysterol binding protein-like 5	ENSMUSG00000037606	68224	84,89
Mcu	mitochondrial calcium uniporter	ENSMUSG00000009647	169709	84,89
Sft2d2	SFT2 domain containing 2	ENSMUSG00000040848	20093	84,81
Ostc	oligosaccharyltransferase complex subunit	ENSMUSG00000041084	13524	84,81
Zranb1	zinc finger, RAN-binding domain containing 1	ENSMUSG00000030967	55250	84,74
Slc25a46	solute carrier family 25, member 46	ENSMUSG00000024259	29735	84,67
Nfe2l1	nuclear factor, erythroid derived 2,-like 1	ENSMUSG00000038615	12555	84,60
Tusc2	tumor suppressor candidate 2	ENSMUSG00000010054	2854	84,60
Snx3	sorting nexin 3	ENSMUSG00000019804	33352	84,60
Eri3	exoribonuclease 3	ENSMUSG00000033423	123933	84,60
Gm10054	predicted gene 10054	ENSMUSG00000058932	402	84,52
Aimp1	aminoacyl tRNA synthetase complex-interacting multifunctional protein 1	ENSMUSG00000028029	23382	84,52
Ten1	TEN1 telomerase capping complex subunit	ENSMUSG00000020778	16464	84,52
Itpa	inosine triphosphatase (nucleoside triphosphate pyrophosphatase)	ENSMUSG00000074797	14005	84,45
Mlec	malectin	ENSMUSG00000048578	15199	84,45
Zc3h6	zinc finger CCCH type containing 6	ENSMUSG00000042851	51162	84,45
Cep170	centrosomal protein 170	ENSMUSG00000057335	73472	84,38
Vps13a	vacuolar protein sorting 13A (yeast)	ENSMUSG00000046230	164060	84,38
Strip1	striatin interacting protein 1	ENSMUSG00000014601	19179	84,31
Mfsd6	major facilitator superfamily domain containing 6	ENSMUSG00000041439	71177	84,31
		ENSMUSG00000069379	618	84,23
Lrriq1	leucine-rich repeats and IQ motif containing 1	ENSMUSG00000019892	173125	84,23
		ENSMUSG00000028577	35731	84,16
S100a13	S100 calcium binding protein A13	ENSMUSG00000042312	10147	84,16
Prpf38a	PRP38 pre-mRNA processing factor 38 (yeast) domain containing A	ENSMUSG00000063800	16165	84,16
Ppp2r4	protein phosphatase 2A, regulatory subunit B (PR 53)	ENSMUSG00000039515	31768	84,16
Prcp	prolylcarboxypeptidase (angiotensinase C)	ENSMUSG00000061119	59321	84,16
Sfmbt1	Scm-like with four mbt domains 1	ENSMUSG00000006527	107865	84,16
Wdsub1	WD repeat, SAM and U-box domain containing 1	ENSMUSG00000026988	30228	84,09
Pphln1	periphilin 1	ENSMUSG00000036167	93564	84,02
Pnn	pinin	ENSMUSG00000020994	7099	84,02

Suco	SUN domain containing ossification factor	ENSMUSG00000040297	60550	84,02
Fam199x	family with sequence similarity 199, X-linked	ENSMUSG00000042595	32910	84,02
Mrps12	mitochondrial ribosomal protein S12	ENSMUSG00000045948	2180	83,94
Thoc7	THO complex 7 homolog (Drosophila)	ENSMUSG00000053453	12212	83,94
Tnfrsf21	tumor necrosis factor receptor superfamily, member 21	ENSMUSG00000023915	72634	83,94
Echs1	enoyl Coenzyme A hydratase, short chain, 1, mitochondrial	ENSMUSG00000025465	10767	83,87
		ENSMUSG00000052214	28210	83,87
Snx5	sorting nexin 5	ENSMUSG00000027423	20784	83,87
Eef1d	eukaryotic translation elongation factor 1 delta (guanine nucleotide exchange protein)	ENSMUSG00000055762	14761	83,80
Hlcs	holocarboxylase synthetase (biotin-[propriony-Coenzyme A-carboxylase (ATP-hydrolysing)] ligase)	ENSMUSG00000040820	184369	83,80
Uba2	ubiquitin-like modifier activating enzyme 2	ENSMUSG00000052997	28911	83,80
Adam10	a disintegrin and metallopeptidase domain 10	ENSMUSG00000054693	101233	83,73
Uri1	URI1, prefoldin-like chaperone	ENSMUSG00000030421	59541	83,73
Prkcz	protein kinase C, zeta	ENSMUSG00000029053	101233	83,65
Tbc1d1	TBC1 domain family, member 1	ENSMUSG00000029174	192036	83,65
Loh12cr1	loss of heterozygosity, 12, chromosomal region 1 homolog (human)	ENSMUSG00000042992	71558	83,65
		ENSMUSG00000046727	57362	83,65
Pla2g16	phospholipase A2, group XVI	ENSMUSG00000060675	31087	83,65
Irf1	interferon regulatory factor 1	ENSMUSG00000018899	8361	83,65
Fam20b	family with sequence similarity 20, member B	ENSMUSG00000033557	40457	83,58
Rab3gap1	RAB3 GTPase activating protein subunit 1	ENSMUSG00000036104	75096	83,58
Rnf169	ring finger protein 169	ENSMUSG00000058761	60202	83,58
Pias1	protein inhibitor of activated STAT 1	ENSMUSG00000032405	100803	83,58
Tars	threonyl-tRNA synthetase	ENSMUSG00000022241	15996	83,51
Psmd7	proteasome (prosome, macropain) 26S subunit, non-ATPase, 7	ENSMUSG00000039067	8102	83,51
Hira	histone cell cycle regulation defective homolog A (S. cerevisiae)	ENSMUSG00000022702	93273	83,51
Bmpr1a	bone morphogenetic protein receptor, type 1A	ENSMUSG00000021796	92608	83,51
		ENSMUSG00000091228	18475	83,44
Hipk1	homeodomain interacting protein kinase 1	ENSMUSG00000008730	51749	83,44
1110001J03Rik	RIKEN cDNA 1110001J03 gene	ENSMUSG00000019689	5948	83,36
Dlst	dihydrolipoamide S-succinyltransferase (E2 component of 2-oxo-glutarate complex)	ENSMUSG00000004789	23259	83,36
Emc3	ER membrane protein complex subunit 3	ENSMUSG00000030286	16765	83,36

Ski	ski sarcoma viral oncogene homolog (avian)	ENSMUSG00000029050	68518	83,29
Cdv3	carnitine deficiency-associated gene expressed in ventricle 3	ENSMUSG00000032803	12679	83,22
Acss1	acyl-CoA synthetase short-chain family member 1	ENSMUSG00000027452	50396	83,15
		ENSMUSG00000002265	26530	83,15
Oard1	O-acyl-ADP-ribose deacylase 1	ENSMUSG00000040771	7253	83,07
Tmx1	thioredoxin-related transmembrane protein 1	ENSMUSG00000021072	14946	83,07
Vps8	vacuolar protein sorting 8 homolog (S. cerevisiae)	ENSMUSG00000033653	221563	83,07
Camk2b	calcium/calmodulin-dependent protein kinase II, beta	ENSMUSG00000057897	96719	83,07
Mrpl14	mitochondrial ribosomal protein L14	ENSMUSG00000023939	12174	83,00
		ENSMUSG00000028879	31009	83,00
Tmem38a	transmembrane protein 38A	ENSMUSG00000031791	15180	83,00
		ENSMUSG00000004317	165549	83,00
Sacm1l	SAC1 (suppressor of actin mutations 1, homolog)-like (S. cerevisiae)	ENSMUSG00000025240	62717	83,00
Nr2f2	nuclear receptor subfamily 2, group F, member 2	ENSMUSG00000030551	14790	82,93
Peli2	pellino 2	ENSMUSG00000021846	140015	82,93
Pi4kb	phosphatidylinositol 4-kinase, catalytic, beta polypeptide	ENSMUSG00000038861	32113	82,93
Arhgef5	Rho guanine nucleotide exchange factor (GEF) 5	ENSMUSG00000033542	23739	82,78
Elmsan1	ELM2 and Myb/SANT-like domain containing 1	ENSMUSG00000042507	69706	82,71
Tuft1	tuftelin 1	ENSMUSG00000005968	46116	82,71
S100a6	S100 calcium binding protein A6 (calcyclin)	ENSMUSG00000001025	1521	82,64
Rab5b	RAB5B, member RAS oncogene family	ENSMUSG00000000711	19076	82,64
Pias2	protein inhibitor of activated STAT 2	ENSMUSG00000025423	90501	82,64
Cyp4f15	cytochrome P450, family 4, subfamily f, polypeptide 15	ENSMUSG00000073424	17726	82,64
Fbxo36	F-box protein 36	ENSMUSG00000073633	60647	82,57
Rab18	RAB18, member RAS oncogene family	ENSMUSG00000073639	25027	82,49
Rbpj	recombination signal binding protein for immunoglobulin kappa J region	ENSMUSG00000039191	101667	82,49
0610007P14Rik	RIKEN cDNA 0610007P14 gene	ENSMUSG00000021252	9103	82,42
Chst9	carbohydrate (N-acetylgalactosamine 4-0) sulfotransferase 9	ENSMUSG00000047161	308234	82,42
Clic4	chloride intracellular channel 4 (mitochondrial)	ENSMUSG00000037242	58846	82,28
Zfp329	zinc finger protein 329	ENSMUSG00000057894	13882	82,28
Aldh1a1	aldehyde dehydrogenase family 1, subfamily A1	ENSMUSG00000053279	41502	82,28
Arpc2	actin related protein 2/3 complex, subunit 2	ENSMUSG00000006304	31713	82,20

Nucb2	nucleobindin 2	ENSMUSG00000030659	36186	82,20
Chd1	chromodomain helicase DNA binding protein 1	ENSMUSG00000023852	67644	82,20
Atp6v1b1	ATPase, H+ transporting, lysosomal V1 subunit B1	ENSMUSG00000006269	15793	82,20
1810041L15Rik	RIKEN cDNA 1810041L15 gene	ENSMUSG00000062760	67893	82,13
Rpl37a	ribosomal protein L37a	ENSMUSG00000046330	2550	82,13
Atp9b	ATPase, class II, type 9B	ENSMUSG00000024566	199916	82,13
Smarca2	SWI/SNF related, matrix associated, actin dependent regulator of chromatin, subfamily a, member 2	ENSMUSG00000024921	173207	82,06
Zbtb4	zinc finger and BTB domain containing 4	ENSMUSG00000018750	18112	82,06
Lonp1	lon peptidase 1, mitochondrial	ENSMUSG00000041168	12603	81,99
Ei24	etoposide induced 2.4 mRNA	ENSMUSG00000062762	18235	81,99
Rhbdd2	rhomboid domain containing 2	ENSMUSG00000039917	13831	81,99
Dcdc2a	doublecortin domain containing 2a	ENSMUSG00000035910	154703	81,91
Rbx1	ring-box 1	ENSMUSG00000022400	10054	81,91
Magoh	mago-nashi homolog, proliferation-associated (Drosophila)	ENSMUSG00000028609	7670	81,91
Nlrp4e	NLR family, pyrin domain containing 4E	ENSMUSG00000045693	60996	81,91
Ankrd28	ankyrin repeat domain 28	ENSMUSG00000014496	130401	81,91
Zfp260	zinc finger protein 260	ENSMUSG00000049421	12846	81,84
		ENSMUSG00000096764	140006	81,84
Ykt6	YKT6 homolog (S. Cerevisiae)	ENSMUSG00000002741	12088	81,77
Hmbox1	homeobox containing 1	ENSMUSG00000021972	138270	81,77
Hagh	hydroxyacyl glutathione hydrolase	ENSMUSG00000024158	24308	81,70
Cdk11b	cyclin-dependent kinase 11B	ENSMUSG00000029062	25085	81,70
Mov10	Moloney leukemia virus 10	ENSMUSG00000002227	23728	81,70
Itga9	integrin alpha 9	ENSMUSG00000039115	294314	81,62
Rfwd2	ring finger and WD repeat domain 2	ENSMUSG00000040782	115255	81,55
Ptpn18	protein tyrosine phosphatase, non-receptor type 18	ENSMUSG00000026126	14030	81,48
Chi3l7	chitinase 3-like 7	ENSMUSG00000043873	7998	81,41
Fezf1	Fez family zinc finger 1	ENSMUSG00000029697	3218	81,33
Ociad1	OCIA domain containing 1	ENSMUSG00000029152	21284	81,33
Thada	thyroid adenoma associated	ENSMUSG00000024251	276141	81,33
Hadha	hydroxyacyl-Coenzyme A dehydrogenase/3-ketoacyl-Coenzyme A thiolase/enoyl-Coenzyme A hydratase (trifunctional protein), alpha subunit	ENSMUSG00000025745	36862	81,26
Tspan1	tetraspanin 1	ENSMUSG00000028699	5733	81,26
Otud4	OTU domain containing 4	ENSMUSG00000036990	38107	81,26
Ccnyl1	cyclin Y-like 1	ENSMUSG00000070871	35190	81,26
Dgki	diacylglycerol kinase, iota	ENSMUSG00000038665	454163	81,26
Arl3	ADP-ribosylation factor-like 3	ENSMUSG00000025035	41977	81,19
		ENSMUSG00000049571	45040	81,12
		ENSMUSG00000037152	4312	81,12
Baz1a	bromodomain adjacent to zinc finger domain 1A	ENSMUSG00000035021	121360	81,12
Tmem150c	transmembrane protein 150C	ENSMUSG00000050640	81937	81,12
Actr3	ARP3 actin-related protein 3	ENSMUSG00000026341	42823	81,12

Nr4a2	nuclear receptor subfamily 4, group A, member 2	ENSMUSG00000026826	17174	81,04
Cd164	CD164 antigen	ENSMUSG00000019818	11543	80,97
Siah2	seven in absentia 2	ENSMUSG00000036432	17463	80,83
Rrp1	ribosomal RNA processing 1 homolog (S. cerevisiae)	ENSMUSG00000061032	12682	80,83
Il13ra1	interleukin 13 receptor, alpha 1	ENSMUSG00000017057	59150	80,83
Nsmf	NMDA receptor synaptonuclear signaling and neuronal migration factor	ENSMUSG00000006476	8527	80,75
Psmc3	proteasome (prosome, macropain) 26S subunit, ATPase 3	ENSMUSG00000002102	12361	80,75
Tsg101	tumor susceptibility gene 101	ENSMUSG00000014402	31021	80,75
Higd1a	HIG1 domain family, member 1A	ENSMUSG00000038412	9441	80,75
Nacc1	nucleus accumbens associated 1, BEN and BTB (POZ) domain containing	ENSMUSG00000001910	17380	80,75
Trim8	tripartite motif-containing 8	ENSMUSG00000025034	14797	80,68
		ENSMUSG00000024019	156181	80,68
Steap2	six transmembrane epithelial antigen of prostate 2	ENSMUSG00000015653	29748	80,68
Usp12	ubiquitin specific peptidase 12	ENSMUSG00000029640	60198	80,61
Hist3h2a	histone cluster 3, H2a	ENSMUSG00000078851	1990	80,61
Rgs22	regulator of G-protein signalling 22	ENSMUSG00000037627	130922	80,61
Ikzf2	IKAROS family zinc finger 2	ENSMUSG00000025997	154747	80,61
Dennd4a	DENN/MADD domain containing 4A	ENSMUSG00000053641	108328	80,61
Rab11a	RAB11a, member RAS oncogene family	ENSMUSG00000004771	22460	80,54
Ppil4	peptidylprolyl isomerase (cyclophilin)-like 4	ENSMUSG00000015757	30245	80,54
Rab28	RAB28, member RAS oncogene family	ENSMUSG00000029128	83180	80,54
Zfp516	zinc finger protein 516	ENSMUSG00000058881	94436	80,46
Smyd1	SET and MYND domain containing 1	ENSMUSG00000055027	108294	80,39
		ENSMUSG00000025607	149241	80,39
Camsap2	calmodulin regulated spectrin-associated protein family, member 2	ENSMUSG00000041570	77982	80,39
		ENSMUSG00000055296	80515	80,39
Nfkbiz	nuclear factor of kappa light polypeptide gene enhancer in B cells inhibitor, zeta	ENSMUSG00000035356	27525	80,32
Trp53bp1	transformation related protein 53 binding protein 1	ENSMUSG00000043909	78127	80,25
Bag3	BCL2-associated athanogene 3	ENSMUSG00000030847	23395	80,25
Gulp1	GULP, engulfment adaptor PTB domain containing 1	ENSMUSG00000056870	245323	80,25
Tpk1	thiamine pyrophosphokinase	ENSMUSG00000029735	321278	80,17
Dpyd	dihydropyrimidine dehydrogenase	ENSMUSG00000033308	870790	80,17
Nedd8	neural precursor cell expressed, developmentally down-regulated gene 8	ENSMUSG00000010376	9642	80,10
		ENSMUSG00000039478	78851	80,10

Sgk1	serum/glucocorticoid regulated kinase 1	ENSMUSG00000019970	117720	80,10
Errfi1	ERBB receptor feedback inhibitor 1	ENSMUSG00000028967	14974	80,10
March5	membrane-associated ring finger (C3HC4) 5	ENSMUSG00000023307	14597	80,03
Ap1s1	adaptor protein complex AP-1, sigma 1	ENSMUSG00000004849	11143	80,03
Slc43a2	solute carrier family 43, member 2	ENSMUSG00000038178	45882	80,03
2310035C23Rik	RIKEN cDNA 2310035C23 gene	ENSMUSG00000026319	91271	80,03
		ENSMUSG00000020723	62201	79,96
Mnat1	menage a trois 1	ENSMUSG00000021103	150272	79,88
Nbas	neuroblastoma amplified sequence	ENSMUSG00000020576	314684	79,88
Ift43	intraflagellar transport 43 homolog (Chlamydomonas)	ENSMUSG00000007867	79898	79,81
Trim37	tripartite motif-containing 37	ENSMUSG00000018548	93607	79,81
Eif4e	eukaryotic translation initiation factor 4E	ENSMUSG00000028156	31409	79,74
Klhl29	kelch-like 29	ENSMUSG00000020627	298211	79,74
Nfe2l3	nuclear factor, erythroid derived 2, like 3	ENSMUSG00000029832	26099	79,74
		ENSMUSG00000045534	165640	79,67
Ppm1g	protein phosphatase 1G (formerly 2C), magnesium-dependent, gamma isoform	ENSMUSG00000029147	17878	79,67
Actr1b	ARP1 actin-related protein 1B, centractin beta	ENSMUSG00000037351	15221	79,59
Atp5l	ATP synthase, H+ transporting, mitochondrial F0 complex, subunit g	ENSMUSG00000038717	7493	79,59
Cacul1	CDK2 associated, cullin domain 1	ENSMUSG00000033417	56328	79,59
Wdr72	WD repeat domain 72	ENSMUSG00000044976	172953	79,59
Grm8	glutamate receptor, metabotropic 8	ENSMUSG00000024211	860058	79,59
4833439L19Rik	RIKEN cDNA 4833439L19 gene	ENSMUSG00000025871	14218	79,52
Mtus1	mitochondrial tumor suppressor 1	ENSMUSG00000045636	142813	79,52
Rnf34	ring finger protein 34	ENSMUSG00000029474	18758	79,52
Ppp2r5e	protein phosphatase 2, regulatory subunit B (B56), epsilon isoform	ENSMUSG00000021051	145318	79,52
Tex14	testis expressed gene 14	ENSMUSG00000010342	150759	79,52
Ccdc115	coiled-coil domain containing 115	ENSMUSG00000042111	3002	79,45
Crot	carnitine O-octanoyltransferase	ENSMUSG00000003623	31292	79,45
Egr2	early growth response 2	ENSMUSG00000037868	6714	79,45
Unc119	unc-119 homolog (C. elegans)	ENSMUSG00000002058	5683	79,38
Atf7	activating transcription factor 7	ENSMUSG00000052414	88768	79,38
Nap1l1	nucleosome assembly protein 1-like 1	ENSMUSG00000058799	24951	79,30
Ngfrap1	nerve growth factor receptor (TNFRSF16) associated protein 1	ENSMUSG00000046432	1726	79,30
Fam149a	family with sequence similarity 149, member A	ENSMUSG00000070044	45575	79,30
Blmh	bleomycin hydrolase	ENSMUSG00000020840	62571	79,30
Sap130	Sin3A associated protein	ENSMUSG00000024260	88691	79,30
Trpc1	transient receptor potential cation channel, subfamily C, member 1	ENSMUSG00000032839	43732	79,30

Smarcd2	SWI/SNF related, matrix associated, actin dependent regulator of chromatin, subfamily d, member 2	ENSMUSG00000078619	9794	79,23
Arpc5	actin related protein 2/3 complex, subunit 5	ENSMUSG00000008475	9056	79,23
Tmem63a	transmembrane protein 63a	ENSMUSG00000026519	32769	79,23
Pvrl2	poliovirus receptor-related 2	ENSMUSG00000062300	32930	79,23
Ddx3y	DEAD (Asp-Glu-Ala-Asp) box polypeptide 3, Y-linked	ENSMUSG00000069045	25899	79,23
Eif2s3x	eukaryotic translation initiation factor 2, subunit 3, structural gene X-linked	ENSMUSG00000035150	24156	79,16
		ENSMUSG00000052812	126644	79,16
		ENSMUSG00000034910	26537	79,09
Fntb	farnesyltransferase, CAAX box, beta	ENSMUSG00000033373	155824	79,09
Pfdn1	prefoldin 1	ENSMUSG00000024346	50817	79,09
Anxa7	annexin A7	ENSMUSG00000021814	24873	79,09
		ENSMUSG00000034981	109280	79,09
R3hdm2	R3H domain containing 2	ENSMUSG00000025404	119058	79,09
Gm10068	predicted gene 10068	ENSMUSG00000059647	1710	79,01
Tm9sf4	transmembrane 9 superfamily protein member 4	ENSMUSG00000068040	49164	79,01
Cdc27	cell division cycle 27	ENSMUSG00000020687	47876	78,94
Cc2d2a	coiled-coil and C2 domain containing 2A	ENSMUSG00000039765	78594	78,94
Cep85	centrosomal protein 85	ENSMUSG00000037443	57255	78,94
		ENSMUSG00000092083	427037	78,94
Fras1	Fraser syndrome 1 homolog (human)	ENSMUSG00000034687	410774	78,94
		ENSMUSG00000034345	6732	78,87
Smndc1	survival motor neuron domain containing 1	ENSMUSG00000025024	11360	78,87
		ENSMUSG00000040044	44149	78,80
Astn1	astrotactin 1	ENSMUSG00000026587	329483	78,80
Ewsr1	Ewing sarcoma breakpoint region 1	ENSMUSG00000009079	29578	78,72
Slc9a8	solute carrier family 9 (sodium/hydrogen exchanger), member 8	ENSMUSG00000039463	55289	78,72
Sec24a	Sec24 related gene family, member A (S. cerevisiae)	ENSMUSG00000036391	71371	78,72
Olig2	oligodendrocyte transcription factor 2	ENSMUSG00000039830	3128	78,65
Lars	leucyl-tRNA synthetase	ENSMUSG00000024493	59773	78,65
Podxl	podocalyxin-like	ENSMUSG00000025608	44494	78,65
Farsa	phenylalanyl-tRNA synthetase, alpha subunit	ENSMUSG00000003808	12269	78,65
Nit1	nitrilase 1	ENSMUSG00000013997	7639	78,65
		ENSMUSG00000020936	40723	78,65
Zcchc2	zinc finger, CCHC domain containing 2	ENSMUSG00000038866	43669	78,65
Pogz	pogo transposable element with ZNF domain	ENSMUSG00000038902	44760	78,58
Mob3b	MOB kinase activator 3B	ENSMUSG00000073910	208411	78,58
2510003E04Rik	RIKEN cDNA 2510003E04 gene	ENSMUSG00000036955	39832	78,51

Ttc5	tetratricopeptide repeat domain 5	ENSMUSG00000006288	20104	78,51
		ENSMUSG00000090213	47215	78,51
Ccpg1	cell cycle progression 1	ENSMUSG00000034563	30912	78,51
Lmtk2	lemur tyrosine kinase 2	ENSMUSG00000038970	87769	78,51
Havcr2	hepatitis A virus cellular receptor 2	ENSMUSG00000020399	26321	78,43
Psmd3	proteasome (prosome, macropain) 26S subunit, non-ATPase, 3	ENSMUSG00000017221	13426	78,43
Tmco1	transmembrane and coiled-coil domains 1	ENSMUSG00000052428	25309	78,43
		ENSMUSG00000063236	2191	78,36
Myo10	myosin X	ENSMUSG00000022272	191149	78,36
Pdlim4	PDZ and LIM domain 4	ENSMUSG00000020388	14087	78,29
Ccdc50	coiled-coil domain containing 50	ENSMUSG00000038127	63350	78,29
Anks1	ankyrin repeat and SAM domain containing 1	ENSMUSG00000024219	153261	78,29
Ubp1	upstream binding protein 1	ENSMUSG00000009741	46266	78,22
Otx1	orthodenticle homolog 1 (Drosophila)	ENSMUSG00000005917	8134	78,22
Lyst	lysosomal trafficking regulator	ENSMUSG00000019726	187032	78,22
4930562C15Rik	RIKEN cDNA 4930562C15 gene	ENSMUSG00000022518	32276	78,22
Hist3h2ba	histone cluster 3, H2ba	ENSMUSG00000056895	622	78,14
Pdpk1	3-phosphoinositide dependent protein kinase 1	ENSMUSG00000024122	77245	78,14
Bicd2	bicaudal D homolog 2 (Drosophila)	ENSMUSG00000037933	45477	78,14
Amotl1	angiomotin-like 1	ENSMUSG00000013076	73518	78,07
Lman2l	lectin, mannose-binding 2-like	ENSMUSG00000001143	23207	78,07
Ndfip2	Nedd4 family interacting protein 2	ENSMUSG00000053253	50726	78,07
Ttc38	tetratricopeptide repeat domain 38	ENSMUSG00000035944	26517	78,00
Mthfr	5,10-methylenetetrahydrofolate reductase	ENSMUSG00000029009	20475	78,00
Kdm3b	KDM3B lysine (K)-specific demethylase 3B	ENSMUSG00000038773	62363	78,00
Scmh1	sex comb on midleg homolog 1	ENSMUSG00000000085	124906	78,00
2510009E07Rik	RIKEN cDNA 2510009E07 gene	ENSMUSG00000043391	45621	77,93
Cnst	consortin, connexin sorting protein	ENSMUSG00000038949	81109	77,93
Hey1	hairy/enhancer-of-split related with YRPW motif 1	ENSMUSG00000040289	3678	77,93
Notch3	notch 3	ENSMUSG00000038146	46061	77,93
Rims3	regulating synaptic membrane exocytosis 3	ENSMUSG00000032890	41764	77,78
Sf3a3	splicing factor 3a, subunit 3	ENSMUSG00000028902	17685	77,78
1810013L24Rik	RIKEN cDNA 1810013L24 gene	ENSMUSG00000022507	28817	77,78
Fmo2	flavin containing monooxygenase 2	ENSMUSG00000040170	24410	77,78
Cct5	chaperonin containing Tcp1, subunit 5 (epsilon)	ENSMUSG00000022234	11048	77,71
Mysm1	myb-like, SWIRM and MPN domains 1	ENSMUSG00000062627	37061	77,71
		ENSMUSG00000039166	138781	77,71
Rpl37	ribosomal protein L37	ENSMUSG00000041841	2528	77,64
Akap8l	A kinase (PRKA) anchor protein 8-like	ENSMUSG00000002625	29157	77,64
Dhx32	DEAH (Asp-Glu-Ala-His) box polypeptide 32	ENSMUSG00000030986	61785	77,56
Sec22c	SEC22 vesicle trafficking protein homolog C (S. cerevisiae)	ENSMUSG00000061536	25444	77,56
Ypel5	yippee-like 5 (Drosophila)	ENSMUSG00000039770	14492	77,56

Dexi	dexamethasone-induced transcript	ENSMUSG00000038055	12848	77,56
Scn5a	sodium channel, voltage-gated, type V, alpha	ENSMUSG00000032511	95609	77,56
Zdhhc17	zinc finger, DHHC domain containing 17	ENSMUSG00000035798	68285	77,56
Cd44	CD44 antigen	ENSMUSG00000005087	90525	77,56
		ENSMUSG00000041298	120892	77,49
Mark1	MAP/microtubule affinity-regulating kinase 1	ENSMUSG00000026620	103107	77,49
		ENSMUSG00000066798	17672	77,42
Cox7a2	cytochrome c oxidase subunit VIIa 2	ENSMUSG00000032330	4613	77,42
Mfhas1	malignant fibrous histiocytoma amplified sequence 1	ENSMUSG00000070056	91652	77,42
1810037I17Rik	RIKEN cDNA 1810037I17 gene	ENSMUSG00000054091	1832	77,42
Klhdc10	kelch domain containing 10	ENSMUSG00000029775	53312	77,42
Nt5c2	5'-nucleotidase, cytosolic II	ENSMUSG00000025041	128323	77,42
Gpc4	glypican 4	ENSMUSG00000031119	112232	77,42
Cttnbp2	cortactin binding protein 2	ENSMUSG00000000416	148861	77,42
Prr14	proline rich 14	ENSMUSG00000030822	17149	77,35
Hid1	HID1 domain containing	ENSMUSG00000034586	20050	77,35
Immt	inner membrane protein, mitochondrial	ENSMUSG00000052337	43927	77,27
Stk19	serine/threonine kinase 19	ENSMUSG00000061207	12953	77,27
Akirin2	akirin 2	ENSMUSG00000028291	15972	77,20
		ENSMUSG00000047642	51305	77,20
E130309F12Rik	RIKEN cDNA E130309F12 gene	ENSMUSG00000063446	280987	77,20
Ttc7b	tetratricopeptide repeat domain 7B	ENSMUSG00000033530	220052	77,13
Mkl1	MKL (megakaryoblastic leukemia)/myocardin-like 1	ENSMUSG00000042292	178477	77,13
Maml2	mastermind like 2 (Drosophila)	ENSMUSG00000031925	89544	77,06
Fut9	fucosyltransferase 9	ENSMUSG00000055373	190913	77,06
Afap1	actin filament associated protein 1	ENSMUSG00000029094	110605	76,98
Eya3	eyes absent 3 homolog (Drosophila)	ENSMUSG00000028886	85779	76,98
Il17re	interleukin 17 receptor E	ENSMUSG00000043088	12268	76,91
Pcsk5	proprotein convertase subtilisin/kexin type 5	ENSMUSG00000024713	404801	76,91
Ncoa5	nuclear receptor coactivator 5	ENSMUSG00000039804	34511	76,84
Zfp706	zinc finger protein 706	ENSMUSG00000062397	10372	76,84
B3gat3	beta-1,3-glucuronyltransferase 3 (glucuronosyltransferase I)	ENSMUSG00000071649	6861	76,84
Glcci1	glucocorticoid induced transcript 1	ENSMUSG00000029638	87949	76,84
Azi2	5-azacytidine induced gene 2	ENSMUSG00000039285	29296	76,77
Wdr13	WD repeat domain 13	ENSMUSG00000031166	9592	76,77
Stx5a	syntaxin 5A	ENSMUSG00000010110	14657	76,77
H2-K1	histocompatibility 2, K1, K region	ENSMUSG00000061232	4317	76,77
Cd2bp2	CD2 antigen (cytoplasmic tail) binding protein 2	ENSMUSG00000042502	4341	76,62
Fam20c	family with sequence similarity 20, member C	ENSMUSG00000025854	55564	76,62
Zdhhc21	zinc finger, DHHC domain containing 21	ENSMUSG00000028403	61221	76,62
Scarb1	scavenger receptor class B, member 1	ENSMUSG00000037936	64008	76,62
Tpi1	triosephosphate isomerase 1	ENSMUSG00000023456	3711	76,55

Samd4b	sterile alpha motif domain containing 4B	ENSMUSG00000037513	198596	76,48
Igsf9	immunoglobulin superfamily, member 9	ENSMUSG00000037995	17091	76,48
Dld	dihydrolipoamide dehydrogenase	ENSMUSG00000020664	19874	76,33
Cabin1	calcineurin binding protein 1	ENSMUSG00000020196	118248	76,33
Paip2	polyadenylate-binding protein-interacting protein 2	ENSMUSG00000037058	18570	76,33
Ly75	lymphocyte antigen 75	ENSMUSG00000026980	91201	76,33
Nicn1	nicolin 1	ENSMUSG00000032606	6051	76,26
Psmd14	proteasome (prosome, macropain) 26S subunit, non-ATPase, 14	ENSMUSG00000026914	88683	76,26
Ss18	synovial sarcoma translocation, Chromosome 18	ENSMUSG00000037013	58717	76,26
Arhgap17	Rho GTPase activating protein 17	ENSMUSG00000030766	90766	76,26
Rnf170	ring finger protein 170	ENSMUSG00000013878	24504	76,26
Lrrc45	leucine rich repeat containing 45	ENSMUSG00000025145	7204	76,26
Myh14	myosin, heavy polypeptide 14	ENSMUSG00000030739	65041	76,26
Slc44a3	solute carrier family 44, member 3	ENSMUSG00000039865	72816	76,26
Fgfr1op2	FGFR1 oncogene partner 2	ENSMUSG00000040242	21996	76,19
Sel1l3	sel-1 suppressor of lin-12-like 3 (C. elegans)	ENSMUSG00000029189	106369	76,11
		ENSMUSG00000028409	21382	76,11
Glt25d1	glycosyltransferase 25 domain containing 1	ENSMUSG00000034807	13886	76,11
Rab3c	RAB3C, member RAS oncogene family	ENSMUSG00000021700	226020	76,11
		ENSMUSG00000051934	86629	76,04
Tmem117	transmembrane protein 117	ENSMUSG00000063296	466913	76,04
Acsl4	acyl-CoA synthetase long-chain family member 4	ENSMUSG00000031278	72543	75,97
Etfb	electron transferring flavoprotein, beta polypeptide	ENSMUSG00000004610	13693	75,90
Ube2z	ubiquitin-conjugating enzyme E2Z (putative)	ENSMUSG00000014349	17957	75,90
Dnajc7	DnaJ (Hsp40) homolog, subfamily C, member 7	ENSMUSG00000014195	37351	75,90
Slc24a4	solute carrier family 24 (sodium/potassium/calcium exchanger), member 4	ENSMUSG00000041771	138359	75,90
Taf15	TAF15 RNA polymerase II, TATA box binding protein (TBP)-associated factor	ENSMUSG00000020680	33658	75,90
AK129341	cDNA sequence AK129341	ENSMUSG00000040729	57662	75,90
Usp15	ubiquitin specific peptidase 15	ENSMUSG00000020124	83692	75,90
BC048546	cDNA sequence BC048546	ENSMUSG00000047228	41785	75,82
Zfp322a	zinc finger protein 322A	ENSMUSG00000046351	16106	75,82
Ezh2	enhancer of zeste homolog 2 (Drosophila)	ENSMUSG00000029687	65068	75,82
Cep95	centrosomal protein 95	ENSMUSG00000018372	30679	75,75
Casp8ap2	caspase 8 associated protein 2	ENSMUSG00000028282	37815	75,75
		ENSMUSG00000090602	855	75,68
Iars	isoleucine-tRNA synthetase	ENSMUSG00000037851	52168	75,68
Ttc4	tetratricopeptide repeat domain 4	ENSMUSG00000025413	16689	75,68
Dock9	dedicator of cytokinesis 9	ENSMUSG00000025558	255696	75,68

Slc4a4	solute carrier family 4 (anion exchanger), member 4	ENSMUSG00000060961	352836	75,61
Rnd3	Rho family GTPase 3	ENSMUSG00000017144	18674	75,61
Coq7	demethyl-Q 7	ENSMUSG00000030652	23698	75,53
Strap	serine/threonine kinase receptor associated protein	ENSMUSG00000030224	16855	75,53
1700021K19Rik	RIKEN cDNA 1700021K19 gene	ENSMUSG00000035629	56064	75,53
Dlg5	discs, large homolog 5 (Drosophila)	ENSMUSG00000021782	111968	75,53
Gtf3c2	general transcription factor IIIC, polypeptide 2, beta	ENSMUSG00000029144	29003	75,46
Sc4mol	sterol-C4-methyl oxidase-like	ENSMUSG00000031604	15654	75,46
Cbl	Casitas B-lineage lymphoma	ENSMUSG00000034342	84785	75,46
Sumo3	SMT3 suppressor of mif two 3 homolog 3 (yeast)	ENSMUSG00000020265	35447	75,39
Hif1a	hypoxia inducible factor 1, alpha subunit	ENSMUSG00000021109	46156	75,39
Fndc3b	fibronectin type III domain containing 3B	ENSMUSG00000039286	294278	75,39
Uty	ubiquitously transcribed tetratricopeptide repeat gene, Y chromosome	ENSMUSG00000068457	148899	75,39
Otud7a	OTU domain containing 7A	ENSMUSG00000033510	314278	75,32
Ostm1	osteopetrosis associated transmembrane protein 1	ENSMUSG00000038280	118638	75,32
Sec31a	Sec31 homolog A (S. cerevisiae)	ENSMUSG00000035325	54586	75,32
Prpf39	PRP39 pre-mRNA processing factor 39 homolog (yeast)	ENSMUSG00000035597	27054	75,32
Rprd2	regulation of nuclear pre-mRNA domain containing 2	ENSMUSG00000028106	59081	75,32
Trmt112	tRNA methyltransferase 11-2	ENSMUSG00000038812	1352	75,24
Sod2	superoxide dismutase 2, mitochondrial	ENSMUSG00000006818	10281	75,24
Myo5c	myosin VC	ENSMUSG00000033590	73438	75,24
Cox5a	cytochrome c oxidase subunit Va	ENSMUSG00000000088	11193	75,17
Mtf1	metal response element binding transcription factor 1	ENSMUSG00000028890	47697	75,17
Nomo1	nodal modulator 1	ENSMUSG00000030835	50515	75,17
		ENSMUSG00000068250	31566	75,10
Vps54	vacuolar protein sorting 54 (yeast)	ENSMUSG00000020128	81856	75,10
Uhrf1bp1l	UHRF1 (ICBP90) binding protein 1-like	ENSMUSG00000019951	74879	75,10
Pex14	peroxisomal biogenesis factor 14	ENSMUSG00000028975	139342	75,10
Man1b1	mannosidase, alpha, class 1B, member 1	ENSMUSG00000036646	19875	75,03
Celsr2	cadherin, EGF LAG seven-pass G-type receptor 2 (flamingo homolog, Drosophila)	ENSMUSG00000068740	24702	75,03
Dnah5	dynein, axonemal, heavy chain 5	ENSMUSG00000022262	268294	75,03
Kcnc4	potassium voltage gated channel, Shaw-related subfamily, member 4	ENSMUSG00000027895	20596	74,95
Necap1	NECAP endocytosis associated 1	ENSMUSG00000030327	14378	74,95
Ddx42	DEAD (Asp-Glu-Ala-Asp) box polypeptide 42	ENSMUSG00000020705	32214	74,88
Rps18	ribosomal protein S18	ENSMUSG00000008668	4003	74,81

Smg5	Smg-5 homolog, nonsense mediated mRNA decay factor (C. elegans)	ENSMUSG00000001415	26078	74,81
Ndel1	nuclear distribution gene E-like homolog 1 (A. nidulans)	ENSMUSG00000018736	50425	74,81
Eftud2	elongation factor Tu GTP binding domain containing 2	ENSMUSG00000020929	42513	74,74
Adcy9	adenylate cyclase 9	ENSMUSG00000005580	132970	74,74
Fam13b	family with sequence similarity 13, member B	ENSMUSG00000036501	64472	74,74
Ube2q1	ubiquitin-conjugating enzyme E2Q (putative) 1	ENSMUSG00000042572	10389	74,67
		ENSMUSG00000024298	17052	74,67
Os9	amplified in osteosarcoma	ENSMUSG00000040462	26902	74,59
Gdi1	guanosine diphosphate (GDP) dissociation inhibitor 1	ENSMUSG00000015291	6865	74,59
Flywch1	FLYWCH-type zinc finger 1	ENSMUSG00000040097	16169	74,52
Stk38	serine/threonine kinase 38	ENSMUSG00000024006	37061	74,52
Shb	src homology 2 domain-containing transforming protein B	ENSMUSG00000044813	109193	74,52
Slc10a7	solute carrier family 10 (sodium/bile acid cotransporter family), member 7	ENSMUSG00000031684	224671	74,52
Tmem184b	transmembrane protein 184b	ENSMUSG00000009035	42620	74,45
		ENSMUSG00000089739	53647	74,45
Memo1	mediator of cell motility 1	ENSMUSG00000058704	94178	74,45
Steap1	six transmembrane epithelial antigen of the prostate 1	ENSMUSG00000015652	13010	74,45
Ptpdc1	protein tyrosine phosphatase domain containing 1	ENSMUSG00000038042	47701	74,38
Ube2e1	ubiquitin-conjugating enzyme E2E 1	ENSMUSG00000021774	49138	74,38
Setd1b	SET domain containing 1B	ENSMUSG00000038384	25243	74,38
Lpcat3	lysophosphatidylcholine acyltransferase 3	ENSMUSG00000004270	41392	74,38
Rundc3b	RUN domain containing 3B	ENSMUSG00000040570	132619	74,38
Emx2	empty spiracles homeobox 2	ENSMUSG00000043969	6986	74,30
Reep1	receptor accessory protein 1	ENSMUSG00000052852	103150	74,30
Exoc5	exocyst complex component 5	ENSMUSG00000061244	54508	74,30
Amot	angiomotin	ENSMUSG00000041688	58757	74,30
Atl2	atlastin GTPase 2	ENSMUSG00000059811	47732	74,30
Ddx39b	DEAD (Asp-Glu-Ala-Asp) box polypeptide 39B	ENSMUSG00000019432	11962	74,23
Hdac2	histone deacetylase 2	ENSMUSG00000019777	27346	74,16
Frk	fyn-related kinase	ENSMUSG00000019779	127827	74,16
Rps6kc1	ribosomal protein S6 kinase polypeptide 1	ENSMUSG00000089872	211569	74,16
Usp8	ubiquitin specific peptidase 8	ENSMUSG00000027363	51970	74,09
Kctd9	potassium channel tetramerisation domain containing 9	ENSMUSG00000034327	26374	74,09
Fktn	fukutin	ENSMUSG00000028414	51788	74,01
Gtf2f2	general transcription factor IIF, polypeptide 2	ENSMUSG00000067995	113929	74,01
Eed	embryonic ectoderm development	ENSMUSG00000030619	26328	73,94
Nrd1	nardilysin, N-arginine dibasic convertase, NRD convertase 1	ENSMUSG00000053510	61123	73,94

Rlim	ring finger protein, LIM domain interacting	ENSMUSG00000056537	24122	73,94
		ENSMUSG00000032134	17431	73,94
Hs6st1	heparan sulfate 6-O-sulfotransferase 1	ENSMUSG00000045216	38047	73,87
Txn1	thioredoxin 1	ENSMUSG00000028367	13039	73,87
4931423N10Rik	RIKEN cDNA 4931423N10 gene	ENSMUSG00000026774	59654	73,87
Rgl2	ral guanine nucleotide dissociation stimulator-like 2	ENSMUSG00000041354	8145	73,87
Rbl2	retinoblastoma-like 2	ENSMUSG00000031666	53788	73,87
Elf2	E74-like factor 2	ENSMUSG00000037174	84980	73,87
Zfp949	zinc finger protein 949	ENSMUSG00000032425	23067	73,87
Asap2	ArfGAP with SH3 domain, ankyrin repeat and PH domain 2	ENSMUSG00000052632	158424	73,87
Rsph9	radial spoke head 9 homolog (Chlamydomonas)	ENSMUSG00000023966	22182	73,80
		ENSMUSG00000023021	39380	73,80
Tox4	TOX high mobility group box family member 4	ENSMUSG00000016831	17246	73,80
Dcun1d1	DCN1, defective in cullin neddylation 1, domain containing 1 (S. cerevisiae)	ENSMUSG00000027708	45341	73,80
		ENSMUSG00000070426	46333	73,80
Cntln	centlein, centrosomal protein	ENSMUSG00000038070	247613	73,80
2810004N23Rik	RIKEN cDNA 2810004N23 gene	ENSMUSG00000031984	23675	73,72
		ENSMUSG00000027035	252842	73,72
Xrn1	5'-3' exoribonuclease 1	ENSMUSG00000032410	98268	73,65
Pdha1	pyruvate dehydrogenase E1 alpha 1	ENSMUSG00000031299	16205	73,58
Map9	microtubule-associated protein 9	ENSMUSG00000033900	37197	73,58
Mark2	MAP/microtubule affinity-regulating kinase 2	ENSMUSG00000024969	66465	73,58
Pid1	phosphotyrosine interaction domain containing 1	ENSMUSG00000045658	327885	73,58
Sf3b5	splicing factor 3b, subunit 5	ENSMUSG00000078348	733	73,51
Nelfb	negative elongation factor complex member B, Cobra1	ENSMUSG00000013465	11778	73,51
Pspc1	paraspeckle protein 1	ENSMUSG00000021938	55876	73,51
Rnf128	ring finger protein 128	ENSMUSG00000031438	109830	73,43
Txndc11	thioredoxin domain containing 11	ENSMUSG00000022498	59740	73,43
		ENSMUSG00000039530	159246	73,43
Trip4	thyroid hormone receptor interactor 4	ENSMUSG00000032386	79865	73,36
Stk24	serine/threonine kinase 24	ENSMUSG00000063410	92992	73,36
Atraid	all-trans retinoic acid induced differentiation factor	ENSMUSG00000013622	6322	73,29
Rngtt	RNA guanylyltransferase and 5'-phosphatase	ENSMUSG00000028274	192304	73,29
Pqlc1	PQ loop repeat containing 1	ENSMUSG00000034006	39434	73,22
Adamts9	a disintegrin-like and metallopeptidase (reprolysin type) with thrombospondin type 1 motif, 9	ENSMUSG00000030022	170794	73,22
Ckmt1	creatine kinase, mitochondrial 1, ubiquitous	ENSMUSG00000000308	6024	73,14

Ndufb10	NADH dehydrogenase (ubiquinone) 1 beta subcomplex, 10	ENSMUSG00000040048	2419	73,14
Ndufb9	NADH dehydrogenase (ubiquinone) 1 beta subcomplex, 9	ENSMUSG00000022354	5680	73,14
Tomm70a	translocase of outer mitochondrial membrane 70 homolog A (yeast)	ENSMUSG00000022752	32817	73,14
4930529M08Rik	RIKEN cDNA 4930529M08 gene	ENSMUSG00000037143	280256	73,14
Zdhhc9	zinc finger, DHHC domain containing 9	ENSMUSG00000036985	36910	73,14
Fert2	fer (fms/fps related) protein kinase, testis specific 2	ENSMUSG00000000127	243477	73,14
Srsf9	serine/arginine-rich splicing factor 9	ENSMUSG00000029538	5904	73,07
Mpv17	MpV17 mitochondrial inner membrane protein	ENSMUSG00000090262	17494	73,07
Pik3c3	phosphoinositide-3-kinase, class 3	ENSMUSG00000033628	75380	73,07
Lamp2	lysosomal-associated membrane protein 2	ENSMUSG00000016534	55098	73,07
Lrrc1	leucine rich repeat containing 1	ENSMUSG00000032352	113237	73,07
Sptbn4	spectrin beta, non-erythrocytic 4	ENSMUSG00000011751	91304	73,07
		ENSMUSG00000071424	1412407	73,00
Fam53c	family with sequence similarity 53, member C	ENSMUSG00000034300	14855	72,93
		ENSMUSG00000072680	4087	72,93
		ENSMUSG00000029530	105019	72,93
Grsf1	G-rich RNA sequence binding factor 1	ENSMUSG00000044221	16724	72,85
Clptm1l	CLPTM1-like	ENSMUSG00000021610	16638	72,85
Naa40	N(alpha)-acetyltransferase 40, NatD catalytic subunit, homolog (S. cerevisiae)	ENSMUSG00000024764	15555	72,85
		ENSMUSG00000072663	170676	72,85
Rasa1	RAS p21 protein activator 1	ENSMUSG00000021549	74250	72,85
Nrxn3	neurexin III	ENSMUSG00000066392	1612060	72,85
Dusp19	dual specificity phosphatase 19	ENSMUSG00000027001	15317	72,78
Pknox1	Pbx/knotted 1 homeobox	ENSMUSG00000006705	42932	72,71
Tmed2	transmembrane emp24 domain trafficking protein 2	ENSMUSG00000029390	9812	72,71
		ENSMUSG00000096262	1096	72,71
Syn2	synapsin II	ENSMUSG00000009394	147725	72,71
Nus1	nuclear undecaprenyl pyrophosphate synthase 1 homolog (S. cerevisiae)	ENSMUSG00000023068	22646	72,71
		ENSMUSG00000032369	21919	72,64
Sik2	salt inducible kinase 2	ENSMUSG00000037112	116273	72,64
Slc28a1	solute carrier family 28 (sodium-coupled nucleoside transporter), member 1	ENSMUSG00000025726	55618	72,56
Tor1aip1	torsin A interacting protein 1	ENSMUSG00000026466	31882	72,56
Ptpn2	protein tyrosine phosphatase, non-receptor type 2	ENSMUSG00000024539	59085	72,56
		ENSMUSG00000079477	46165	72,49
Safb	scaffold attachment factor B	ENSMUSG00000071054	21470	72,49
Phlpp2	PH domain and leucine rich repeat protein phosphatase 2	ENSMUSG00000031732	76130	72,49
		ENSMUSG00000015536	10763	72,42
Zfp618	zinc finger protein 618	ENSMUSG00000028358	174136	72,42

Esf1	ESF1, nucleolar pre-rRNA processing protein, homolog (S. cerevisiae)	ENSMUSG00000045624	50682	72,42
Map3k4	mitogen-activated protein kinase kinase kinase 4	ENSMUSG00000014426	91040	72,42
Ltbp1	latent transforming growth factor beta binding protein 1	ENSMUSG00000001870	386945	72,42
Vmn2r91	vomeronasal 2, receptor 91	ENSMUSG00000091206	51587	72,42
Serinc3	serine incorporator 3	ENSMUSG00000017707	21860	72,35
Fam114a2	family with sequence similarity 114, member A2	ENSMUSG00000020523	35625	72,35
D430042O09Rik	RIKEN cDNA D430042O09 gene	ENSMUSG00000032743	166879	72,27
Tm9sf2	transmembrane 9 superfamily member 2	ENSMUSG00000025544	52567	72,27
Slc39a10	solute carrier family 39 (zinc transporter), member 10	ENSMUSG00000025986	46503	72,20
Sos1	son of sevenless homolog 1 (Drosophila)	ENSMUSG00000024241	86702	72,20
Trappc10	trafficking protein particle complex 10	ENSMUSG00000000374	57918	72,13
Top2a	topoisomerase (DNA) II alpha	ENSMUSG00000020914	31247	72,06
Tnpo2	transportin 2 (importin 3, karyopherin beta 2b)	ENSMUSG00000031691	20669	72,06
Ankrd6	ankyrin repeat domain 6	ENSMUSG00000040183	146807	72,06
Opa1	optic atrophy 1	ENSMUSG00000038084	75551	71,91
Tcerg1	transcription elongation regulator 1 (CA150)	ENSMUSG00000024498	64042	71,91
Eea1	early endosome antigen 1	ENSMUSG00000036499	104856	71,91
Hnrnph2	heterogeneous nuclear ribonucleoprotein H2	ENSMUSG00000045427	5882	71,84
Psma1	proteasome (prosome, macropain) subunit, alpha type 1	ENSMUSG00000030751	11513	71,84
		ENSMUSG00000033623	45745	71,84
Arl13b	ADP-ribosylation factor-like 13B	ENSMUSG00000022911	53356	71,84
Tbc1d22a	TBC1 domain family, member 22a	ENSMUSG00000051864	284045	71,84
		ENSMUSG00000025006	107219	71,84
Adamts6	a disintegrin-like and metallopeptidase (reprolysin type) with thrombospondin type 1 motif, 6	ENSMUSG00000046169	206891	71,77
Tbcb	tubulin folding cofactor B	ENSMUSG00000006095	8142	71,69
1700024P16Rik	RIKEN cDNA 1700024P16 gene	ENSMUSG00000078612	103408	71,69
Iscu	IscU iron-sulfur cluster scaffold homolog (E. coli)	ENSMUSG00000025825	5541	71,62
Klf9	Kruppel-like factor 9	ENSMUSG00000033863	25686	71,62
Sf3b3	splicing factor 3b, subunit 3	ENSMUSG00000033732	36312	71,55
		ENSMUSG00000060073	21727	71,55
Acbd3	acyl-Coenzyme A binding domain containing 3	ENSMUSG00000026499	28161	71,55
		ENSMUSG00000063663	97357	71,55
Setx	senataxin	ENSMUSG00000043535	58291	71,55
Agps	alkylglycerone phosphate synthase	ENSMUSG00000042410	99174	71,55
Micu2	mitochondrial calcium uptake 2	ENSMUSG00000021973	82983	71,48
Depdc7	DEP domain containing 7	ENSMUSG00000027173	21095	71,48

Sys1	SYS1 Golgi-localized integral membrane protein homolog (S. cerevisiae)	ENSMUSG00000045503	22675	71,40
9430023L20Rik	RIKEN cDNA 9430023L20 gene	ENSMUSG00000037204	6634	71,40
Phlda1	pleckstrin homology-like domain, family A, member 1	ENSMUSG00000020205	2360	71,33
Mageh1	melanoma antigen, family H, 1	ENSMUSG00000047238	1398	71,33
Usp36	ubiquitin specific peptidase 36	ENSMUSG00000033909	30594	71,33
Hipk3	homeodomain interacting protein kinase 3	ENSMUSG00000027177	67966	71,33
Smarcb1	SWI/SNF related, matrix associated, actin dependent regulator of chromatin, subfamily b, member 1	ENSMUSG00000000902	24849	71,26
Sap18	Sin3-associated polypeptide 18	ENSMUSG00000021963	6801	71,26
Ppat	phosphoribosyl pyrophosphate amidotransferase	ENSMUSG00000029246	38330	71,26
Mrps9	mitochondrial ribosomal protein S9	ENSMUSG00000060679	54450	71,26
Ubac2	ubiquitin associated domain containing 2	ENSMUSG00000041765	142429	71,26
Zfp827	zinc finger protein 827	ENSMUSG00000071064	165330	71,26
Pgap2	post-GPI attachment to proteins 2	ENSMUSG00000030990	28360	71,26
9130230L23Rik	RIKEN cDNA 9130230L23 gene	ENSMUSG00000054598	24928	71,26
Psma5	proteasome (prosome, macropain) subunit, alpha type 5	ENSMUSG00000068749	23049	71,19
Elp5	elongator acetyltransferase complex subunit 5	ENSMUSG00000018565	14296	71,19
Setd3	SET domain containing 3	ENSMUSG00000056770	72884	71,19
Snap91	synaptosomal-associated protein 91	ENSMUSG00000033419	114719	71,19
Actr1a	ARP1 actin-related protein 1A, centractin alpha	ENSMUSG00000025228	18922	71,11
Dhx9	DEAH (Asp-Glu-Ala-His) box polypeptide 9	ENSMUSG00000042699	31903	71,11
Mtmr14	myotubularin related protein 14	ENSMUSG00000030269	43545	71,11
Stag2	stromal antigen 2	ENSMUSG00000025862	127869	71,11
Emc10	ER membrane protein complex subunit 10	ENSMUSG00000008140	6593	71,04
Sep 02	septin 2	ENSMUSG00000026276	31297	71,04
Med1	mediator complex subunit 1	ENSMUSG00000018160	41140	71,04
Hdac8	histone deacetylase 8	ENSMUSG00000067567	220721	71,04
Tango6	transport and golgi organization 6	ENSMUSG00000041949	168372	71,04
Zkscan3	zinc finger with KRAB and SCAN domains 3	ENSMUSG00000021327	15743	70,97
Cd81	CD81 antigen	ENSMUSG00000037706	15196	70,90
Crabp1	cellular retinoic acid binding protein I	ENSMUSG00000032291	8363	70,90
St3gal4	ST3 beta-galactoside alpha-2,3-sialyltransferase 4	ENSMUSG00000032038	70232	70,90
Acaa1a	acetyl-Coenzyme A acyltransferase 1A	ENSMUSG00000036138	10624	70,82
Chuk	conserved helix-loop-helix ubiquitous kinase	ENSMUSG00000025199	34146	70,82
Stat5a	signal transducer and activator of transcription 5A	ENSMUSG00000004043	25819	70,82

Chmp2b	charged multivesicular body protein 2B	ENSMUSG00000004843	23565	70,68
Ccdc62	coiled-coil domain containing 62	ENSMUSG00000061882	42478	70,68
2310044G17Rik	RIKEN cDNA 2310044G17 gene	ENSMUSG00000034157	18020	70,61
Cdh18	cadherin 18	ENSMUSG00000040420	925397	70,61
Gucd1	guanylyl cyclase domain containing 1	ENSMUSG00000033416	166862	70,61
		ENSMUSG00000090663	5881	70,61
Cfdp1	craniofacial development protein 1	ENSMUSG00000031954	85838	70,61
Thra	thyroid hormone receptor alpha	ENSMUSG00000058756	28369	70,53
Tsku	tsukushi	ENSMUSG00000049580	10661	70,53
Tox3	TOX high mobility group box family member 3	ENSMUSG00000043668	101304	70,53
Fam117a	family with sequence similarity 117, member A	ENSMUSG00000038893	44855	70,53
B630005N14Rik	RIKEN cDNA B630005N14 gene	ENSMUSG00000042742	52290	70,53
Agpat6	1-acylglycerol-3-phosphate O-acyltransferase 6 (lysophosphatidic acid acyltransferase, zeta)	ENSMUSG00000031545	35508	70,46
		ENSMUSG00000027364	74363	70,46
March3	membrane-associated ring finger (C3HC4) 3	ENSMUSG00000032656	163833	70,46
Slc25a51	solute carrier family 25, member 51	ENSMUSG00000045973	12844	70,39
Pmvk	phosphomevalonate kinase	ENSMUSG00000027952	9896	70,39
4833420G17Rik	RIKEN cDNA 4833420G17 gene	ENSMUSG00000062822	23359	70,39
Rnf214	ring finger protein 214	ENSMUSG00000042790	43470	70,39
Atxn7	ataxin 7	ENSMUSG00000021738	94806	70,39
		ENSMUSG00000026032	9581	70,32
Mut	methylmalonyl-Coenzyme A mutase	ENSMUSG00000023921	27305	70,32
Nln	neurolysin (metallopeptidase M3 family)	ENSMUSG00000021710	86176	70,32
Klf5	Kruppel-like factor 5	ENSMUSG00000005148	14715	70,32
Raver2	ribonucleoprotein, PTB-binding 2	ENSMUSG00000035275	83388	70,32
Enkur	enkurin, TRPC channel interacting protein	ENSMUSG00000026679	24635	70,24
Kif5a	kinesin family member 5A	ENSMUSG00000074657	37668	70,24
		ENSMUSG00000013150	44811	70,24
		ENSMUSG00000027010	96687	70,24
Ergic2	ERGIC and golgi 2	ENSMUSG00000030304	33296	70,24
		ENSMUSG00000028842	97853	70,24
Adcy3	adenylate cyclase 3	ENSMUSG00000020654	80423	70,17
Ghitm	growth hormone inducible transmembrane protein	ENSMUSG00000041028	14879	70,17
		ENSMUSG00000000149	72024	70,17
Atg13	autophagy related 13	ENSMUSG00000027244	35959	70,17
Spata6	spermatogenesis associated 6	ENSMUSG00000034401	109201	70,17
Amotl2	angiomotin-like 2	ENSMUSG00000032531	16747	70,17
Lrsam1	leucine rich repeat and sterile alpha motif containing 1	ENSMUSG00000026792	36399	70,10
Tmem254b	transmembrane protein 254b	ENSMUSG00000021867	4375	70,10
Ttll3	tubulin tyrosine ligase-like family, member 3	ENSMUSG00000030276	25324	70,03
Tcea1	transcription elongation factor A (SII) 1	ENSMUSG00000033813	40096	70,03
Bsdc1	BSD domain containing 1	ENSMUSG00000040859	26918	70,03

Arfgap3	ADP-ribosylation factor GTPase activating protein 3	ENSMUSG00000054277	50508	70,03
Myof	myoferlin	ENSMUSG00000048612	144542	70,03
Unc5b	unc-5 homolog B (C. elegans)	ENSMUSG00000020099	68972	69,95
Alkbh5	alkB, alkylation repair homolog 5 (E. coli)	ENSMUSG00000042650	22132	69,95
Ncor2	nuclear receptor co-repressor 2	ENSMUSG00000029478	162067	69,95
Ralgds	ral guanine nucleotide dissociation stimulator	ENSMUSG00000026821	39957	69,88
Cct4	chaperonin containing Tcp1, subunit 4 (delta)	ENSMUSG00000007739	13262	69,88
Arid4a	AT rich interactive domain 4A (RBP1-like)	ENSMUSG00000048118	82603	69,88
Vps29	vacuolar protein sorting 29 (S. pombe)	ENSMUSG00000029462	10616	69,81
Plekhm3	pleckstrin homology domain containing, family M, member 3	ENSMUSG00000051344	170842	69,81
Rpl36-ps3	ribosomal protein L36, pseudogene 3	ENSMUSG00000066629	384	69,74
Axin1	axin 1	ENSMUSG00000024182	57124	69,74
Nmnat3	nicotinamide nucleotide adenylyltransferase 3	ENSMUSG00000032456	133093	69,74
Map1a	microtubule-associated protein 1 A	ENSMUSG00000027254	21233	69,66
Mapk3	mitogen-activated protein kinase 3	ENSMUSG00000063065	6219	69,59
		ENSMUSG00000031950	12910	69,59
Sh3gl2	SH3-domain GRB2-like 2	ENSMUSG00000028488	434070	69,59
Atp5c1	ATP synthase, H+ transporting, mitochondrial F1 complex, gamma polypeptide 1	ENSMUSG00000025781	24495	69,52
Stard10	START domain containing 10	ENSMUSG00000030688	29541	69,52
Ptpn13	protein tyrosine phosphatase, non-receptor type 13	ENSMUSG00000034573	173168	69,52
Usp33	ubiquitin specific peptidase 33	ENSMUSG00000025437	47135	69,45
Map3k7	mitogen-activated protein kinase kinase kinase 7	ENSMUSG00000028284	59371	69,45
Taf7	TAF7 RNA polymerase II, TATA box binding protein (TBP)-associated factor	ENSMUSG00000051316	3303	69,45
Kif1c	kinesin family member 1C	ENSMUSG00000020821	31417	69,45
Rps6ka5	ribosomal protein S6 kinase, polypeptide 5	ENSMUSG00000021180	175251	69,45
Agtrap	angiotensin II, type I receptor-associated protein	ENSMUSG00000029007	10971	69,37
		ENSMUSG00000031563	164461	69,37
		ENSMUSG00000027770	36966	69,37
Ttc28	tetratricopeptide repeat domain 28	ENSMUSG00000033209	409978	69,37
St3gal5	ST3 beta-galactoside alpha-2,3-sialyltransferase 5	ENSMUSG00000056091	56958	69,37
Ocln	occludin	ENSMUSG00000021638	56212	69,37
Trib3	tribbles homolog 3 (Drosophila)	ENSMUSG00000032715	6611	69,30
Atg4b	autophagy related 4B, cysteine peptidase	ENSMUSG00000026280	37935	69,30
Ift81	intraflagellar transport 81	ENSMUSG00000029469	64315	69,30
Cdyl	chromodomain protein, Y chromosome-like	ENSMUSG00000059288	214203	69,30
Prom1	prominin 1	ENSMUSG00000029086	108408	69,30

Ctage5	CTAGE family, member 5	ENSMUSG00000021000	61864	69,30
Ints2	integrator complex subunit 2	ENSMUSG00000018068	46895	69,30
Pcyox1	prenylcysteine oxidase 1	ENSMUSG00000029998	11145	69,23
Pa2g4	proliferation-associated 2G4	ENSMUSG00000025364	8169	69,23
Pcmtd2	protein-L-isoaspartate (D-aspartate) O-methyltransferase domain containing 2	ENSMUSG00000027589	19608	69,23
Senp7	SUMO1/sentrin specific peptidase 7	ENSMUSG00000052917	114603	69,23
Tmed5	transmembrane emp24 protein transport domain containing 5	ENSMUSG00000063406	26255	69,23
Ppfibp1	PTPRF interacting protein, binding protein 1 (liprin beta 1)	ENSMUSG00000016487	143539	69,23
Unc50	unc-50 homolog (C. elegans)	ENSMUSG00000026111	8955	69,16
Mlxip	MLX interacting protein	ENSMUSG00000038342	63135	69,16
Xrcc5	X-ray repair complementing defective repair in Chinese hamster cells 5	ENSMUSG00000026187	87532	69,08
Amer1	APC membrane recruitment 1	ENSMUSG00000050332	24555	69,08
Cflar	CASP8 and FADD-like apoptosis regulator	ENSMUSG00000026031	47377	69,01
Epb4.1l4b	erythrocyte protein band 4.1-like 4b	ENSMUSG00000028434	151466	69,01
Dhx40	DEAH (Asp-Glu-Ala-His) box polypeptide 40	ENSMUSG00000018425	38851	69,01
Med14	mediator complex subunit 14	ENSMUSG00000064127	86705	68,94
Snrpc	U1 small nuclear ribonucleoprotein C	ENSMUSG00000024217	11995	68,87
		ENSMUSG00000072640	20287	68,87
Pgp	phosphoglycolate phosphatase	ENSMUSG00000043445	1119	68,87
Tanc1	tetratricopeptide repeat, ankyrin repeat and coiled-coil containing 1	ENSMUSG00000035168	234108	68,87
Wnt7b	wingless-related MMTV integration site 7B	ENSMUSG00000022382	46383	68,87
Sntb1	syntrophin, basic 1	ENSMUSG00000060429	268107	68,87
Wdr45b	WD repeat domain 45B	ENSMUSG00000025173	27222	68,72
Mpp5	membrane protein, palmitoylated 5 (MAGUK p55 subfamily member 5)	ENSMUSG00000021112	91765	68,72
Pibf1	progesterone immunomodulatory binding factor 1	ENSMUSG00000022064	155061	68,72
Aggf1	angiogenic factor with G patch and FHA domains 1	ENSMUSG00000021681	24670	68,65
Mtss1	metastasis suppressor 1	ENSMUSG00000022353	140783	68,65
Madd	MAP-kinase activating death domain	ENSMUSG00000040687	46478	68,58
Wbp1l	WW domain binding protein 1 like	ENSMUSG00000047731	58306	68,58
Cldn23	claudin 23	ENSMUSG00000055976	1851	68,58
Usp42	ubiquitin specific peptidase 42	ENSMUSG00000051306	21956	68,58
Syncrip	synaptotagmin binding, cytoplasmic RNA interacting protein	ENSMUSG00000032423	32846	68,58
Eps15l1	epidermal growth factor receptor pathway substrate 15-like 1	ENSMUSG00000006276	80474	68,58
Vdac3	voltage-dependent anion channel 3	ENSMUSG00000008892	16739	68,50
Gtf3c1	general transcription factor III C 1	ENSMUSG00000032777	66826	68,50
1810046K07Rik	RIKEN cDNA 1810046K07 gene	ENSMUSG00000036027	39232	68,50
Trim35	tripartite motif-containing 35	ENSMUSG00000022043	14394	68,43

Plagl1	pleiomorphic adenoma gene-like 1	ENSMUSG00000019817	41004	68,43
		ENSMUSG00000096319	869	68,36
Gatc	glutamyl-tRNA(Gln) amidotransferase, subunit C homolog (bacterial)	ENSMUSG00000029536	7937	68,36
Cdc42bpg	CDC42 binding protein kinase gamma (DMPK-like)	ENSMUSG00000024769	19197	68,36
N4bp2	NEDD4 binding protein 2	ENSMUSG00000037795	66588	68,36
Samm50	sorting and assembly machinery component 50 homolog (S. cerevisiae)	ENSMUSG00000022437	22071	68,29
Ltbr	lymphotoxin B receptor	ENSMUSG00000030339	7315	68,29
D10Bwg1379e	DNA segment, Chr 10, Brigham & Women's Genetics 1379 expressed	ENSMUSG00000019852	155748	68,29
Rae1	RAE1 RNA export 1 homolog (S. pombe)	ENSMUSG00000027509	15623	68,21
Psmb7	proteasome (prosome, macropain) subunit, beta type 7	ENSMUSG00000026750	56052	68,21
Lats2	large tumor suppressor 2	ENSMUSG00000021959	116312	68,21
Vti1b	vesicle transport through interaction with t-SNAREs 1B	ENSMUSG00000021124	16651	68,21
Gaa	glucosidase, alpha, acid	ENSMUSG00000025579	17568	68,14
Mtmr4	myotubularin related protein 4	ENSMUSG00000018401	24141	68,14
Fbxw7	F-box and WD-40 domain protein 7	ENSMUSG00000028086	163931	68,14
Nolc1	nucleolar and coiled-body phosphoprotein 1	ENSMUSG00000015176	9666	68,07
Rrn3	RRN3 RNA polymerase I transcription factor homolog (yeast)	ENSMUSG00000022682	34142	68,07
Scand1	SCAN domain-containing 1	ENSMUSG00000046229	858	68,07
Nfic	nuclear factor I/C	ENSMUSG00000055053	34820	68,07
Rrnad1	ribosomal RNA adenine dimethylase domain containing 1	ENSMUSG00000004896	8117	68,07
Tns3	tensin 3	ENSMUSG00000020422	232884	68,07
BC004004	cDNA sequence BC004004	ENSMUSG00000052712	34094	68,00
Mtr	5-methyltetrahydrofolate-homocysteine methyltransferase	ENSMUSG00000021311	71572	68,00
Sec23b	SEC23B (S. cerevisiae)	ENSMUSG00000027429	34521	67,92
Atad1	ATPase family, AAA domain containing 1	ENSMUSG00000013662	39736	67,92
Cdkn2aipnl	CDKN2A interacting protein N-terminal like	ENSMUSG00000020392	9674	67,85
Map2k1	mitogen-activated protein kinase kinase 1	ENSMUSG00000004936	67862	67,85
Arhgef7	Rho guanine nucleotide exchange factor (GEF7)	ENSMUSG00000031511	107115	67,85
Sdc2	syndecan 2	ENSMUSG00000022261	114008	67,71
		ENSMUSG00000027011	53170	67,71
		ENSMUSG00000087153	16008	67,71
Sppl2a	signal peptide peptidase like 2A	ENSMUSG00000027366	42845	67,71
9330182L06Rik	RIKEN cDNA 9330182L06 gene	ENSMUSG00000056004	215708	67,71
Irf5	interferon regulatory factor 5	ENSMUSG00000029771	15245	67,63
Supt6	suppressor of Ty 6	ENSMUSG00000002052	39242	67,63
Gng12	guanine nucleotide binding protein (G protein), gamma 12	ENSMUSG00000036402	124954	67,63

Parp1	poly (ADP-ribose) polymerase family, member 1	ENSMUSG00000026496	32299	67,56
Atp5h	ATP synthase, H+ transporting, mitochondrial F0 complex, subunit d	ENSMUSG00000034566	4274	67,56
Rgma	RGM domain family, member A	ENSMUSG00000070509	44380	67,56
Tmem161b	transmembrane protein 161B	ENSMUSG00000035762	73671	67,56
Snapc5	small nuclear RNA activating complex, polypeptide 5	ENSMUSG00000032398	3388	67,49
Lacc1	laccase (multicopper oxidoreductase) domain containing 1	ENSMUSG00000044350	12705	67,49
Ahsa1	AHA1, activator of heat shock protein ATPase 1	ENSMUSG00000021037	7252	67,42
Atg16l1	autophagy related 16-like 1 (S. cerevisiae)	ENSMUSG00000026289	36559	67,42
		ENSMUSG00000006050	4516	67,34
Rpl27	ribosomal protein L27	ENSMUSG00000063316	3232	67,34
Mmadhc	methylmalonic aciduria (cobalamin deficiency) cblD type, with homocystinuria	ENSMUSG00000026766	16921	67,34
Ube2d1	ubiquitin-conjugating enzyme E2D 1	ENSMUSG00000019927	30283	67,34
Ubald2	UBA-like domain containing 2	ENSMUSG00000050628	4984	67,34
Metap2	methionine aminopeptidase 2	ENSMUSG00000036112	38605	67,34
Nup210	nucleoporin 210	ENSMUSG00000030091	103762	67,34
Dlg4	discs, large homolog 4 (Drosophila)	ENSMUSG00000020886	28448	67,34
Vwa8	von Willebrand factor A domain containing 8	ENSMUSG00000058997	353133	67,34
Camk2n1	calcium/calmodulin-dependent protein kinase II inhibitor 1	ENSMUSG00000046447	5810	67,27
Krcc1	lysine-rich coiled-coil 1	ENSMUSG00000053012	13300	67,27
Exoc3	exocyst complex component 3	ENSMUSG00000034152	38896	67,27
Pola1	polymerase (DNA directed), alpha 1	ENSMUSG00000006678	327389	67,27
Nt5dc3	5'-nucleotidase domain containing 3	ENSMUSG00000054027	59385	67,27
Sycp3	synaptonemal complex protein 3	ENSMUSG00000020059	13668	67,27
Ndufv3	NADH dehydrogenase (ubiquinone) flavoprotein 3	ENSMUSG00000024038	11207	67,20
		ENSMUSG00000035086	16335	67,20
Ano2	anoctamin 2	ENSMUSG00000038115	349708	67,20
Pfkfb2	6-phosphofructo-2-kinase/fructose-2,6-biphosphatase 2	ENSMUSG00000026409	40211	67,20
Fxr1	fragile X mental retardation gene 1, autosomal homolog	ENSMUSG00000027680	49264	67,20
Ccdc77	coiled-coil domain containing 77	ENSMUSG00000030177	40052	67,13
Abcb6	ATP-binding cassette, sub-family B (MDR/TAP), member 6	ENSMUSG00000026198	8676	67,13
Prr14l	proline rich 14-like	ENSMUSG00000054280	65039	67,13
Fermt2	fermitin family homolog 2 (Drosophila)	ENSMUSG00000037712	71327	67,13
Gm12695	predicted gene 12695	ENSMUSG00000078639	61540	67,13
Ift46	intraflagellar transport 46	ENSMUSG00000002031	24807	67,05
Gm5577	predicted gene 5577	ENSMUSG00000084950	24589	67,05
Slain2	SLAIN motif family, member 2	ENSMUSG00000036087	64526	67,05

Ift88	intraflagellar transport 88	ENSMUSG00000040040	93875	67,05
Dapk2	death-associated protein kinase 2	ENSMUSG00000032380	114020	67,05
Bod1	biorientation of chromosomes in cell division 1	ENSMUSG00000044502	6705	66,98
Cs	citrate synthase	ENSMUSG00000005683	24648	66,98
Cdc42se2	CDC42 small effector 2	ENSMUSG00000052298	70220	66,98
Klhl24	kelch-like 24	ENSMUSG00000062901	30191	66,98
Synj1	synaptojanin 1	ENSMUSG00000022973	75217	66,98
Dnmt1	DNA methyltransferase (cytosine-5) 1	ENSMUSG00000004099	52683	66,91
Nrbp2	nuclear receptor binding protein 2	ENSMUSG00000075590	4419	66,91
Phf12	PHD finger protein 12	ENSMUSG00000037791	47786	66,91
Sult4a1	sulfotransferase family 4A, member 1	ENSMUSG00000018865	29658	66,84
Fam103a1	family with sequence similarity 103, member A1	ENSMUSG00000038646	6567	66,84
Bcr	breakpoint cluster region	ENSMUSG00000009681	124028	66,84
Rab43	RAB43, member RAS oncogene family	ENSMUSG00000030055	23312	66,84
Hbs1l	Hbs1-like (S. cerevisiae)	ENSMUSG00000019977	72911	66,76
Pigb	phosphatidylinositol glycan anchor biosynthesis, class B	ENSMUSG00000079469	24004	66,76
Pdia3	protein disulfide isomerase associated 3	ENSMUSG00000027248	24913	66,69
		ENSMUSG00000022772	40782	66,69
Sulf1	sulfatase 1	ENSMUSG00000016918	167942	66,69
Smad3	SMAD family member 3	ENSMUSG00000032402	111228	66,69
Nxf1	nuclear RNA export factor 1	ENSMUSG00000010097	13807	66,62
		ENSMUSG00000030447	88956	66,62
		ENSMUSG00000044783	15163	66,55
Ipo11	importin 11	ENSMUSG00000042590	142477	66,55
Vangl2	vang-like 2 (van gogh, Drosophila)	ENSMUSG00000026556	27485	66,47
Arhgap18	Rho GTPase activating protein 18	ENSMUSG00000039031	165228	66,47
Rps10	ribosomal protein S10	ENSMUSG00000052146	6208	66,40
Sp1	trans-acting transcription factor 1	ENSMUSG00000001280	30262	66,40
Slco4c1	solute carrier organic anion transporter family, member 4C1	ENSMUSG00000040693	53388	66,40
Crym	crystallin, mu	ENSMUSG00000030905	15732	66,33
Nsun7	NOL1/NOP2/Sun domain family, member 7	ENSMUSG00000029206	38105	66,33
Capn5	calpain 5	ENSMUSG00000035547	56716	66,26
Fbxl2	F-box and leucine-rich repeat protein 2	ENSMUSG00000032507	82555	66,26
Map3k2	mitogen-activated protein kinase kinase kinase 2	ENSMUSG00000024383	73663	66,26
Fam3c	family with sequence similarity 3, member C	ENSMUSG00000029672	49724	66,18
A630007B06Rik	RIKEN cDNA A630007B06 gene	ENSMUSG00000035173	34710	66,18
Fam179b	family with sequence similarity 179, member B	ENSMUSG00000035614	56832	66,18
Uvssa	UV stimulated scaffold protein A	ENSMUSG00000037355	41204	66,11
Slc17a6	solute carrier family 17 (sodium-dependent inorganic phosphate cotransporter), member 6	ENSMUSG00000030500	49296	66,04
Tom1	target of myb1 homolog (chicken)	ENSMUSG00000042870	36436	66,04
		ENSMUSG00000067194	17751	66,04

Hydin	HYDIN, axonemal central pair apparatus protein	ENSMUSG00000059854	343277	66,04
Mfn2	mitofusin 2	ENSMUSG00000029020	31106	65,97
Atox1	ATX1 (antioxidant protein 1) homolog 1 (yeast)	ENSMUSG00000018585	14599	65,97
Chmp5	charged multivesicular body protein 5	ENSMUSG00000028419	16897	65,89
Parg	poly (ADP-ribose) glycohydrolase	ENSMUSG00000021911	95602	65,89
Larp4	La ribonucleoprotein domain family, member 4	ENSMUSG00000023025	46278	65,89
Ranbp2	RAN binding protein 2	ENSMUSG00000003226	47304	65,89
		ENSMUSG00000022383	67837	65,89
		ENSMUSG00000025495	34640	65,82
Pdlim5	PDZ and LIM domain 5	ENSMUSG00000028273	156111	65,82
Atp8a1	ATPase, aminophospholipid transporter (APLT), class I, type 8A, member 1	ENSMUSG00000037685	229293	65,82
Eif2b5	eukaryotic translation initiation factor 2B, subunit 5 epsilon	ENSMUSG00000003235	11139	65,75
Kif3b	kinesin family member 3B	ENSMUSG00000027475	41978	65,75
Ubap1	ubiquitin-associated protein 1	ENSMUSG00000028437	41530	65,75
		ENSMUSG00000029475	144413	65,75
Elmod3	ELMO/CED-12 domain containing 3	ENSMUSG00000056698	32492	65,60
Napa	N-ethylmaleimide sensitive fusion protein attachment protein alpha	ENSMUSG00000006024	19518	65,60
Mrpl50	mitochondrial ribosomal protein L50	ENSMUSG00000044018	8498	65,60
Dhrs1	dehydrogenase/reductase (SDR family) member 1	ENSMUSG00000002332	6671	65,60
		ENSMUSG00000045576	62358	65,60
Dnajc3	DnaJ (Hsp40) homolog, subfamily C, member 3	ENSMUSG00000022136	43771	65,60
Ddx10	DEAD (Asp-Glu-Ala-Asp) box polypeptide 10	ENSMUSG00000053289	149659	65,60
Pnkd	paroxysmal nonkinesiogenic dyskinesia	ENSMUSG00000026179	68765	65,60
Rtca	RNA 3'-terminal phosphate cyclase	ENSMUSG00000000339	19246	65,53
Pink1	PTEN induced putative kinase 1	ENSMUSG00000028756	12899	65,53
Pcyt1a	phosphate cytidylyltransferase 1, choline, alpha isoform	ENSMUSG00000005615	44150	65,53
		ENSMUSG00000041842	36395	65,53
Tfg	Trk-fused gene	ENSMUSG00000022757	27119	65,46
Sestd1	SEC14 and spectrin domains 1	ENSMUSG00000042272	100253	65,46
Slc25a13	solute carrier family 25 (mitochondrial carrier, adenine nucleotide translocator), member 13	ENSMUSG00000015112	175901	65,46
Hist1h2ae	histone cluster 1, H2ae	ENSMUSG00000069272	704	65,39
		ENSMUSG00000069302	531	65,39
Scoc	short coiled-coil protein	ENSMUSG00000063253	23899	65,39
Fam171a2	family with sequence similarity 171, member A2	ENSMUSG00000034685	10702	65,39
Ranbp10	RAN binding protein 10	ENSMUSG00000037415	59043	65,39
Pik3cb	phosphatidylinositol 3-kinase, catalytic, beta polypeptide	ENSMUSG00000032462	102220	65,39

		ENSMUSG00000057230	142348	65,39
Dnah9	dynein, axonemal, heavy chain 9	ENSMUSG00000056752	337270	65,39
Eaf1	ELL associated factor 1	ENSMUSG00000021890	14780	65,31
Tdrp	testis development related protein	ENSMUSG00000050052	23024	65,31
Stx8	syntaxin 8	ENSMUSG00000020903	240956	65,31
Adarb2	adenosine deaminase, RNA-specific, B2	ENSMUSG00000052551	565882	65,24
Kpnb1	karyopherin (importin) beta 1	ENSMUSG00000001440	28168	65,24
Tspan12	tetraspanin 12	ENSMUSG00000029669	81121	65,24
Dgat2	diacylglycerol O-acyltransferase 2	ENSMUSG00000030747	29051	65,24
Cyth1	cytohesin 1	ENSMUSG00000017132	116574	65,24
Atxn2l	ataxin 2-like	ENSMUSG00000032637	11730	65,17
Pdp1	pyruvate dehyrogenase phosphatase catalytic subunit 1	ENSMUSG00000049225	8269	65,17
Fasn	fatty acid synthase	ENSMUSG00000025153	18239	65,17
Rhbdd1	rhomboid domain containing 1	ENSMUSG00000026142	128915	65,17
Fyco1	FYVE and coiled-coil domain containing 1	ENSMUSG00000025241	62390	65,17
Cmtm8	CKLF-like MARVEL transmembrane domain containing 8	ENSMUSG00000041012	54808	65,17
Gcc2	GRIP and coiled-coil domain containing 2	ENSMUSG00000038039	50103	65,17
		ENSMUSG00000023089	9348	65,10
R3hcc1l	R3H domain and coiled-coil containing 1 like	ENSMUSG00000025184	73585	65,10
Gbe1	glucan (1,4-alpha-), branching enzyme 1	ENSMUSG00000022707	255768	65,10
Elmo2	engulfment and cell motility 2	ENSMUSG00000017670	38449	65,02
Srp19	signal recognition particle 19	ENSMUSG00000014504	5753	65,02
Gab1	growth factor receptor bound protein 2-associated protein 1	ENSMUSG00000031714	116046	65,02
Negr1	neuronal growth regulator 1	ENSMUSG00000040037	754652	65,02
Htatsf1	HIV TAT specific factor 1	ENSMUSG00000067873	13601	64,95
		ENSMUSG00000018661	17886	64,95
Spidr	scaffolding protein involved i DNA repair	ENSMUSG00000041974	257625	64,95
		ENSMUSG00000018166	22130	64,95
Egfr	epidermal growth factor receptor	ENSMUSG00000020122	165956	64,95
		ENSMUSG00000029199	19197	64,88
Mybbp1a	MYB binding protein (P160) 1a	ENSMUSG00000040463	10414	64,88
Pogk	pogo transposable element with KRAB domain	ENSMUSG00000040596	25242	64,88
Gm872	predicted gene 872	ENSMUSG00000020014	306000	64,88
Sbno2	strawberry notch homolog 2 (Drosophila)	ENSMUSG00000035673	45283	64,88
Asb7	ankyrin repeat and SOCS box-containing 7	ENSMUSG00000030509	45032	64,73
		ENSMUSG00000027746	8619	64,73
Gns	glucosamine (N-acetyl)-6-sulfatase	ENSMUSG00000034707	32156	64,73
Fbxw8	F-box and WD-40 domain protein 8	ENSMUSG00000032867	90478	64,73
Wipf2	WAS/WASL interacting protein family, member 2	ENSMUSG00000038013	41403	64,73
		ENSMUSG00000021578	15475	64,66
3110057O12Rik	RIKEN cDNA 3110057O12 gene	ENSMUSG00000037818	91169	64,66
D930015E06Rik	RIKEN cDNA D930015E06 gene	ENSMUSG00000033767	141875	64,66

Tmem131	transmembrane protein 131	ENSMUSG00000026116	147337	64,66
		ENSMUSG00000025134	6245	64,59
1700009P17Rik	RIKEN cDNA 1700009P17 gene	ENSMUSG00000026649	13050	64,59
Acad11	acyl-Coenzyme A dehydrogenase family, member 11	ENSMUSG00000090150	86802	64,59
Dach1	dachshund 1 (Drosophila)	ENSMUSG00000055639	382913	64,59
Tmod2	tropomodulin 2	ENSMUSG00000032186	45705	64,52
Efr3b	EFR3 homolog B (S. cerevisiae)	ENSMUSG00000020658	76362	64,52
Scp2	sterol carrier protein 2, liver	ENSMUSG00000028603	101160	64,52
Mfsd4	major facilitator superfamily domain containing 4	ENSMUSG00000059149	45257	64,44
Cetn2	centrin 2	ENSMUSG00000031347	4880	64,44
Zfp217	zinc finger protein 217	ENSMUSG00000052056	39461	64,44
Ankmy2	ankyrin repeat and MYND domain containing 2	ENSMUSG00000036188	40168	64,44
Stmn4	stathmin-like 4	ENSMUSG00000022044	17385	64,37
Blm	Bloom syndrome, RecQ helicase-like	ENSMUSG00000030528	80127	64,37
Fam134c	family with sequence similarity 134, member C	ENSMUSG00000017802	23572	64,37
Fkbp15	FK506 binding protein 15	ENSMUSG00000066151	60207	64,37
Sdccag8	serologically defined colon cancer antigen 8	ENSMUSG00000026504	205778	64,37
Fbxo38	F-box protein 38	ENSMUSG00000042211	44675	64,30
2210018M11Rik	RIKEN cDNA 2210018M11 gene	ENSMUSG00000035401	65955	64,30
Cwc27	CWC27 spliceosome-associated protein homolog (S. cerevisiae)	ENSMUSG00000021715	186003	64,30
Eno1	enolase 1, alpha non-neuron	ENSMUSG00000063524	12159	64,23
Parp6	poly (ADP-ribose) polymerase family, member 6	ENSMUSG00000025237	33007	64,23
Uqcr11	ubiquinol-cytochrome c reductase, complex III subunit XI	ENSMUSG00000020163	3834	64,23
Fam104a	family with sequence similarity 104, member A	ENSMUSG00000041629	22837	64,23
Cd24a	CD24a antigen	ENSMUSG00000047139	5094	64,23
Gramd1c	GRAM domain containing 1C	ENSMUSG00000036292	47596	64,15
Hdhd2	haloacid dehalogenase-like hydrolase domain containing 2	ENSMUSG00000025421	42777	64,15
Anks3	ankyrin repeat and sterile alpha motif domain containing 3	ENSMUSG00000022515	22823	64,15
Mrps33	mitochondrial ribosomal protein S33	ENSMUSG00000029918	9185	64,08
Rbms3	RNA binding motif, single stranded interacting protein	ENSMUSG00000039607	1057168	64,08
		ENSMUSG00000037709	92460	64,08
Uqcc1	ubiquinol-cytochrome c reductase complex assembly factor 1	ENSMUSG00000005882	83417	64,08
Agfg1	ArfGAP with FG repeats 1	ENSMUSG00000026159	56793	64,08
Gpcpd1	glycerophosphocholine phosphodiesterase GDE1 homolog (S. cerevisiae)	ENSMUSG00000027346	58648	64,01
Coa3	cytochrome C oxidase assembly factor 3	ENSMUSG00000017188	1147	63,94
		ENSMUSG00000037740	4954	63,94
Hhla1	HERV-H LTR-associating 1	ENSMUSG00000072511	54362	63,86
		ENSMUSG00000060923	143597	63,86

Ptk7	PTK7 protein tyrosine kinase 7	ENSMUSG00000023972	65054	63,86
Zeb1	zinc finger E-box binding homeobox 1	ENSMUSG00000024238	183608	63,86
Rtfdc1	replication termination factor 2 domain containing 1	ENSMUSG00000027502	29353	63,79
Tmed3	transmembrane emp24 domain containing 3	ENSMUSG00000032353	5841	63,79
Ypel1	yippee-like 1 (Drosophila)	ENSMUSG00000022773	17350	63,79
Snx18	sorting nexin 18	ENSMUSG00000042364	26385	63,79
Ube2l3	ubiquitin-conjugating enzyme E2L 3	ENSMUSG00000038965	50637	63,79
Zcchc14	zinc finger, CCHC domain containing 14	ENSMUSG00000061410	54199	63,79
Ubxn7	UBX domain protein 7	ENSMUSG00000053774	61496	63,79
H2-D1	histocompatibility 2, D region locus 1	ENSMUSG00000073411	4770	63,79
Gm15800	predicted gene 15800	ENSMUSG00000042744	148359	63,79
Mtmr12	myotubularin related protein 12	ENSMUSG00000039458	67213	63,79
Pecr	peroxisomal trans-2-enoyl-CoA reductase	ENSMUSG00000026189	25148	63,72
Arl8b	ADP-ribosylation factor-like 8B	ENSMUSG00000030105	40665	63,72
Socs6	suppressor of cytokine signaling 6	ENSMUSG00000056153	262258	63,72
P4ha1	procollagen-proline, 2-oxoglutarate 4-dioxygenase (proline 4-hydroxylase), alpha 1 polypeptide	ENSMUSG00000019916	50009	63,72
Lrrk1	leucine-rich repeat kinase 1	ENSMUSG00000015133	161439	63,72
Txnl4a	thioredoxin-like 4A	ENSMUSG00000057130	16739	63,65
Mrpl33	mitochondrial ribosomal protein L33	ENSMUSG00000029142	105038	63,65
Fxr2	fragile X mental retardation, autosomal homolog 2	ENSMUSG00000018765	20308	63,65
2610015P09Rik	RIKEN cDNA 2610015P09 gene	ENSMUSG00000022701	74515	63,65
Arfgef2	ADP-ribosylation factor guanine nucleotide-exchange factor 2 (brefeldin A-inhibited)	ENSMUSG00000074582	92465	63,65
Nrxn2	neurexin II	ENSMUSG00000033768	125439	63,57
		ENSMUSG00000051255	869	63,57
Phc1	polyhomeotic-like 1 (Drosophila)	ENSMUSG00000040669	22831	63,57
Tank	TRAF family member-associated Nf-kappa B activator	ENSMUSG00000064289	75587	63,57
Atp10b	ATPase, class V, type 10B	ENSMUSG00000055415	112409	63,57
Derl2	Der1-like domain family, member 2	ENSMUSG00000018442	12678	63,50
Cnot10	CCR4-NOT transcription complex, subunit 10	ENSMUSG00000056167	54323	63,50
Zfp451	zinc finger protein 451	ENSMUSG00000042197	54124	63,50
Rgs20	regulator of G-protein signaling 20	ENSMUSG00000002459	160710	63,50
		ENSMUSG00000070713	1159	63,43
Coro1c	coronin, actin binding protein 1C	ENSMUSG00000004530	66323	63,43
Zcrb1	zinc finger CCHC-type and RNA binding motif 1	ENSMUSG00000022635	12238	63,43
Dusp6	dual specificity phosphatase 6	ENSMUSG00000019960	4258	63,43
Ppp1r12c	protein phosphatase 1, regulatory (inhibitor) subunit 12C	ENSMUSG00000019254	20161	63,43
Ackr3	atypical chemokine receptor 3	ENSMUSG00000044337	12772	63,43
Tapt1	transmembrane anterior posterior transformation 1	ENSMUSG00000046985	51443	63,43

Kif13b	kinesin family member 13B	ENSMUSG00000060012	153766	63,43
Rnf7	ring finger protein 7	ENSMUSG00000051234	7735	63,36
Ccdc104	coiled-coil domain containing 104	ENSMUSG00000020462	25878	63,36
Sgsm2	small G protein signaling modulator 2	ENSMUSG00000038351	47800	63,36
Ppm1h	protein phosphatase 1H (PP2C domain containing)	ENSMUSG00000034613	267034	63,36
Ttyh3	tweety homolog 3 (Drosophila)	ENSMUSG00000036565	28446	63,28
Tbce	tubulin-specific chaperone E	ENSMUSG00000039233	41690	63,28
Zfp553	zinc finger protein 553	ENSMUSG00000045598	5119	63,21
Slc25a39	solute carrier family 25, member 39	ENSMUSG00000018677	4962	63,21
Pde4dip	phosphodiesterase 4D interacting protein (myomegalin)	ENSMUSG00000038170	198884	63,21
Plat	plasminogen activator, tissue	ENSMUSG00000031538	25104	63,21
Atp6v0b	ATPase, H+ transporting, lysosomal V0 subunit B	ENSMUSG00000033379	3008	63,14
Iws1	IWS1 homolog (S. cerevisiae)	ENSMUSG00000024384	36598	63,14
Rybp	RING1 and YY1 binding protein	ENSMUSG00000072872	58876	63,14
Sall1	sal-like 1 (Drosophila)	ENSMUSG00000031665	16915	63,14
Spag16	sperm associated antigen 16	ENSMUSG00000053153	898145	63,14
Pycr2	pyrroline-5-carboxylate reductase family, member 2	ENSMUSG00000026520	3814	63,07
Ift122	intraflagellar transport 122	ENSMUSG00000030323	73230	63,07
Rnf115	ring finger protein 115	ENSMUSG00000028098	63545	63,07
Fbxo3	F-box protein 3	ENSMUSG00000027180	35520	63,07
Adss	adenylosuccinate synthetase, non muscle	ENSMUSG00000015961	33550	63,07
Ggps1	geranylgeranyl diphosphate synthase 1	ENSMUSG00000021302	10953	63,07
Gramd3	GRAM domain containing 3	ENSMUSG00000001700	71661	63,07
Sntb2	syntrophin, basic 2	ENSMUSG00000041308	78443	63,07
Bfar	bifunctional apoptosis regulator	ENSMUSG00000022684	31755	62,99
Swt1	SWT1 RNA endoribonuclease homolog (S. cerevisiae)	ENSMUSG00000052748	60757	62,99
		ENSMUSG00000053465	731180	62,99
Anp32e	acidic (leucine-rich) nuclear phosphoprotein 32 family, member E	ENSMUSG00000015749	18145	62,92
Atg2b	autophagy related 2B	ENSMUSG00000041341	71703	62,92
		ENSMUSG00000066415	30058	62,92
Gsta3	glutathione S-transferase, alpha 3	ENSMUSG00000025934	24773	62,92
		ENSMUSG00000038576	132739	62,92
Pcca	propionyl-Coenzyme A carboxylase, alpha polypeptide	ENSMUSG00000041650	356777	62,92
		ENSMUSG00000062758	801	62,85
Nsun2	NOL1/NOP2/Sun domain family member 2	ENSMUSG00000021595	102035	62,85
		ENSMUSG00000029176	33783	62,85
Ash2l	ash2 (absent, small, or homeotic)-like (Drosophila)	ENSMUSG00000031575	31504	62,85
Ccdc73	coiled-coil domain containing 73	ENSMUSG00000045106	150100	62,85
Bnip2	BCL2/adenovirus E1B interacting protein 2	ENSMUSG00000011958	18852	62,78
Crmp1	collapsin response mediator protein 1	ENSMUSG00000029121	50053	62,78
Rbm10	RNA binding motif protein 10	ENSMUSG00000031060	33399	62,78

Pign	phosphatidylinositol glycan anchor biosynthesis, class N	ENSMUSG00000056536	140587	62,78
Tmem165	transmembrane protein 165	ENSMUSG00000029234	25366	62,78
		ENSMUSG00000071516	1041	62,70
Vopp1	vesicular, overexpressed in cancer, prosurvival protein 1	ENSMUSG00000037788	97348	62,70
Kdm3a	lysine (K)-specific demethylase 3A	ENSMUSG00000053470	43932	62,70
Kctd20	potassium channel tetramerisation domain containing 20	ENSMUSG00000005936	17600	62,63
Dlx6	distal-less homeobox 6	ENSMUSG00000029754	5235	62,63
Hsbp1	heat shock factor binding protein 1	ENSMUSG00000031839	4390	62,56
Lrrc14	leucine rich repeat containing 14	ENSMUSG00000033728	7077	62,56
		ENSMUSG00000030879	4442	62,56
Bpnt1	bisphosphate 3'-nucleotidase 1	ENSMUSG00000026617	25629	62,56
A830010M20Rik	RIKEN cDNA A830010M20 gene	ENSMUSG00000044060	83219	62,49
Idh1	isocitrate dehydrogenase 1 (NADP+), soluble	ENSMUSG00000025950	27864	62,49
Fbxo33	F-box protein 33	ENSMUSG00000035329	19071	62,49
Dydc2	DPY30 domain containing 2	ENSMUSG00000021791	19866	62,49
		ENSMUSG00000015733	30739	62,41
Prmt5	protein arginine N-methyltransferase 5	ENSMUSG00000023110	10339	62,34
Pttg1ip	pituitary tumor-transforming 1 interacting protein	ENSMUSG00000009291	17013	62,34
Rbm42	RNA binding motif protein 42	ENSMUSG00000036733	9324	62,27
Sgip1	SH3-domain GRB2-like (endophilin) interacting protein 1	ENSMUSG00000028524	232332	62,27
Hdac4	histone deacetylase 4	ENSMUSG00000026313	215637	62,27
Rnf220	ring finger protein 220	ENSMUSG00000028677	225590	62,20
Bcl9	B cell CLL/lymphoma 9	ENSMUSG00000038256	25185	62,20
Dad1	defender against cell death 1	ENSMUSG00000022174	18626	62,20
Vwa3a	von Willebrand factor A domain containing 3A	ENSMUSG00000030889	66186	62,20
Dock5	dedicator of cytokinesis 5	ENSMUSG00000044447	181645	62,20
		ENSMUSG00000032435	35513	62,12
Pop5	processing of precursor 5, ribonuclease P/MRP family (S. cerevisiae)	ENSMUSG00000060152	9516	62,12
		ENSMUSG00000060904	13237	62,12
Rcbtb2	regulator of chromosome condensation (RCC1) and BTB (POZ) domain containing protein 2	ENSMUSG00000022106	84807	62,12
Nsmaf	neutral sphingomyelinase (N-SMase) activation associated factor	ENSMUSG00000028245	58065	62,12
Tfcp2l1	transcription factor CP2-like 1	ENSMUSG00000026380	57223	62,12
Atf7ip	activating transcription factor 7 interacting protein	ENSMUSG00000030213	88527	62,12
Nedd9	neural precursor cell expressed, developmentally down-regulated gene 9	ENSMUSG00000021365	177405	62,12
D10Jhu81e	DNA segment, Chr 10, Johns Hopkins University 81 expressed	ENSMUSG00000053329	7702	62,05
Ppp1r2	protein phosphatase 1, regulatory (inhibitor) subunit 2	ENSMUSG00000047714	23741	62,05
		ENSMUSG00000027323	34653	62,05

Terf2ip	telomeric repeat binding factor 2, interacting protein	ENSMUSG00000033430	9170	61,91
Rfc2	replication factor C (activator 1) 2	ENSMUSG00000023104	15639	61,91
Sox5	SRY-box containing gene 5	ENSMUSG00000041540	953553	61,91
Tom1l1	target of myb1-like 1 (chicken)	ENSMUSG00000020541	44902	61,91
Atp11c	ATPase, class VI, type 11C	ENSMUSG00000062949	369409	61,91
Il11ra1	interleukin 11 receptor, alpha chain 1	ENSMUSG00000073889	69486	61,83
Nhp2	NHP2 ribonucleoprotein	ENSMUSG00000001056	3980	61,83
Kansl2	KAT8 regulatory NSL complex subunit 2	ENSMUSG00000022992	15424	61,83
C2cd3	C2 calcium-dependent domain containing 3	ENSMUSG00000047248	97927	61,83
Rnf130	ring finger protein 130	ENSMUSG00000020376	79413	61,83
Olig1	oligodendrocyte transcription factor 1	ENSMUSG00000046160	2171	61,76
Diablo	diablo homolog (Drosophila)	ENSMUSG00000029433	14412	61,76
Btbd10	BTB (POZ) domain containing 10	ENSMUSG00000038187	53767	61,76
Rph3al	rabphilin 3A-like (without C2 domains)	ENSMUSG00000020847	107431	61,76
Ugt8a	UDP galactosyltransferase 8A	ENSMUSG00000032854	73192	61,76
Fhad1	forkhead-associated (FHA) phosphopeptide binding domain 1	ENSMUSG00000051435	124645	61,69
Pitx1	paired-like homeodomain transcription factor 1	ENSMUSG00000021506	11139	61,69
		ENSMUSG00000040473	84962	61,69
Cntd1	cyclin N-terminal domain containing 1	ENSMUSG00000078653	17306	61,62
Snw1	SNW domain containing 1	ENSMUSG00000021039	22364	61,62
Cmtm6	CKLF-like MARVEL transmembrane domain containing 6	ENSMUSG00000032434	18141	61,62
Rin2	Ras and Rab interactor 2	ENSMUSG00000001768	102883	61,62
Kif6	kinesin family member 6	ENSMUSG00000023999	294712	61,62
		ENSMUSG00000071478	572	61,54
Rpl36a	ribosomal protein L36A	ENSMUSG00000079435	2409	61,54
Necab1	N-terminal EF-hand calcium binding protein 1	ENSMUSG00000040536	197550	61,54
		ENSMUSG00000035725	34868	61,54
Acvr2b	activin receptor IIB	ENSMUSG00000061393	31058	61,47
Tceanc2	transcription elongation factor A (SII) N-terminal and central domain containing 2	ENSMUSG00000028619	44962	61,47
Magi2	membrane associated guanylate kinase, WW and PDZ domain containing 2	ENSMUSG00000040003	1477753	61,47
Tbc1d32	TBC1 domain family, member 32	ENSMUSG00000038122	214392	61,47
Stk3	serine/threonine kinase 3	ENSMUSG00000022329	280311	61,47
		ENSMUSG00000034584	75845	61,47
Fam32a	family with sequence similarity 32, member A	ENSMUSG00000003039	4035	61,40
Zc3h14	zinc finger CCCH type containing 14	ENSMUSG00000021012	40807	61,40
Mapk6	mitogen-activated protein kinase 6	ENSMUSG00000042688	40944	61,40
Serinc5	serine incorporator 5	ENSMUSG00000021703	100807	61,40
		ENSMUSG00000062203	34979	61,33

Jak2	Janus kinase 2	ENSMUSG00000024789	61253	61,33
Smarcad1	SWI/SNF-related, matrix-associated actin-dependent regulator of chromatin, subfamily a, containing DEAD/H box 1	ENSMUSG00000029920	73384	61,33
C2cd5	C2 calcium-dependent domain containing 5	ENSMUSG00000030279	89188	61,33
Golga7b	golgi autoantigen, golgin subfamily a, 7B	ENSMUSG00000042532	22775	61,25
Trp53i11	transformation related protein 53 inducible protein 11	ENSMUSG00000068735	14212	61,25
Stard7	START domain containing 7	ENSMUSG00000027367	28715	61,25
Sdf2	stromal cell derived factor 2	ENSMUSG00000002064	9751	61,25
Lmna	lamin A	ENSMUSG00000028063	28809	61,25
Ccdc91	coiled-coil domain containing 91	ENSMUSG00000030301	156742	61,25
Upf3b	UPF3 regulator of nonsense transcripts homolog B (yeast)	ENSMUSG00000036572	18645	61,25
Eif3j2	eukaryotic translation initiation factor 3, subunit J2	ENSMUSG00000043424	2419	61,18
Kif11	kinesin family member 11	ENSMUSG00000012443	45457	61,11
Pygl	liver glycogen phosphorylase	ENSMUSG00000021069	40678	61,11
Ncln	nicalin homolog (zebrafish)	ENSMUSG00000020238	10144	61,04
Arl5a	ADP-ribosylation factor-like 5A	ENSMUSG00000036093	26951	61,04
Sertad1	SERTA domain containing 1	ENSMUSG00000008384	3407	61,04
		ENSMUSG00000092072	395	61,04
Rtcb	RNA 2',3'-cyclic phosphate and 5'-OH ligase	ENSMUSG00000001783	19187	60,96
		ENSMUSG00000039849	15151	60,96
Atp6v0e	ATPase, H+ transporting, lysosomal V0 subunit E	ENSMUSG00000015575	23249	60,96
Metap1	methionyl aminopeptidase 1	ENSMUSG00000005813	30423	60,96
		ENSMUSG00000026748	399536	60,96
A830080D01Rik	RIKEN cDNA A830080D01 gene	ENSMUSG00000044150	66394	60,96
Nfkb1	nuclear factor of kappa light polypeptide gene enhancer in B cells 1, p105	ENSMUSG00000028163	106893	60,96
Cdipt	CDP-diacylglycerol--inositol 3-phosphatidyltransferase (phosphatidylinositol synthase)	ENSMUSG00000030682	4151	60,89
Uba52	ubiquitin A-52 residue ribosomal protein fusion product 1	ENSMUSG00000090137	2539	60,89
Hadh	hydroxyacyl-Coenzyme A dehydrogenase	ENSMUSG00000027984	38683	60,82
Chmp3	charged multivesicular body protein 3	ENSMUSG00000053119	38813	60,82
Prdm10	PR domain containing 10	ENSMUSG00000042496	98006	60,82
Fmr1	fragile X mental retardation syndrome 1	ENSMUSG00000000838	39423	60,75
Oraov1	oral cancer overexpressed 1	ENSMUSG00000031072	15649	60,67
Sfxn1	sideroflexin 1	ENSMUSG00000021474	36502	60,67
2300009A05Rik	RIKEN cDNA 2300009A05 gene	ENSMUSG00000032403	4798	60,67
Tasp1	taspase, threonine aspartase 1	ENSMUSG00000039033	233326	60,67
Gnl1	guanine nucleotide binding protein-like 1	ENSMUSG00000024429	9612	60,53
Smc6	structural maintenance of chromosomes 6	ENSMUSG00000020608	53900	60,53

Slbp	stem-loop binding protein	ENSMUSG00000004642	17623	60,53
Ralgps1	Ral GEF with PH domain and SH3 binding motif 1	ENSMUSG00000038831	238066	60,53
Tex9	testis expressed gene 9	ENSMUSG00000090626	33905	60,46
Srp9	signal recognition particle 9	ENSMUSG00000026511	7671	60,46
Cox10	cytochrome c oxidase assembly protein 10	ENSMUSG00000042148	116842	60,46
Mapkapk2	MAP kinase-activated protein kinase 2	ENSMUSG00000016528	43840	60,46
Fhod3	formin homology 2 domain containing 3	ENSMUSG00000034295	424056	60,46
Lnpep	leucyl/cystinyl aminopeptidase	ENSMUSG00000023845	96767	60,46
Paics	phosphoribosylaminoimidazole carboxylase, phosphoribosylaminoribosylaminoimidazole, succinocarboxamide synthetase	ENSMUSG00000029247	16203	60,38
Foxk2	forkhead box K2	ENSMUSG00000039275	49907	60,38
		ENSMUSG00000021824	21067	60,38
Scd2	stearoyl-Coenzyme A desaturase 2	ENSMUSG00000025203	13189	60,31
R3hdm4	R3H domain containing 4	ENSMUSG00000035781	6954	60,31
Bet1l	blocked early in transport 1 homolog (S. cerevisiae)-like	ENSMUSG00000025484	3000	60,31
Plekhm2	pleckstrin homology domain containing, family M (with RUN domain) member 2	ENSMUSG00000028917	39166	60,31
Cpox	coproporphyrinogen oxidase	ENSMUSG00000022742	10184	60,31
Sdf4	stromal cell derived factor 4	ENSMUSG00000029076	20739	60,31
Banp	BTG3 associated nuclear protein	ENSMUSG00000025316	79509	60,31
Ppip5k2	diphosphoinositol pentakisphosphate kinase 2	ENSMUSG00000040648	64364	60,24
Gak	cyclin G associated kinase	ENSMUSG00000062234	60329	60,24
Cul9	cullin 9	ENSMUSG00000040327	45817	60,24
Ddx46	DEAD (Asp-Glu-Ala-Asp) box polypeptide 46	ENSMUSG00000021500	46230	60,17
		ENSMUSG00000036279	3079	60,17
Ino80d	INO80 complex subunit D	ENSMUSG00000040865	156250	60,17
Capza1	capping protein (actin filament) muscle Z-line, alpha 1	ENSMUSG00000070372	41727	60,09
Zfp266	zinc finger protein 266	ENSMUSG00000060510	26350	60,09
Cox16	cytochrome c oxidase assembly protein 16	ENSMUSG00000091803	126102	60,09
Tecr	trans-2,3-enoyl-CoA reductase	ENSMUSG00000031708	22792	60,02
		ENSMUSG00000020922	22800	60,02
Dmd	dystrophin, muscular dystrophy	ENSMUSG00000045103	2257272	60,02
Gtdc1	glycosyltransferase-like domain containing 1	ENSMUSG00000036890	363246	60,02
Tbcc	tubulin-specific chaperone C	ENSMUSG00000036430	1843	59,95
0610009O20Rik	RIKEN cDNA 0610009O20 gene	ENSMUSG00000024442	12381	59,95
Csnk2a2	casein kinase 2, alpha prime polypeptide	ENSMUSG00000046707	42725	59,95
Golga1	golgi autoantigen, golgin subfamily a, 1	ENSMUSG00000026754	49281	59,95
		ENSMUSG00000078620	7011	59,95
Phf8	PHD finger protein 8	ENSMUSG00000041229	113188	59,95

Git2	G protein-coupled receptor kinase-interactor 2	ENSMUSG00000041890	48111	59,95
Slc25a5	solute carrier family 25 (mitochondrial carrier, adenine nucleotide translocator), member 5	ENSMUSG00000016319	3157	59,88
Slc18b1	solute carrier family 18, subfamily B, member 1	ENSMUSG00000037455	30983	59,88
Fkbp5	FK506 binding protein 5	ENSMUSG00000024222	118430	59,80
Vprbp	Vpr (HIV-1) binding protein	ENSMUSG00000040325	61445	59,80
Rnf144b	ring finger protein 144B	ENSMUSG00000038068	125272	59,80
Cpt1a	carnitine palmitoyltransferase 1a, liver	ENSMUSG00000024900	62432	59,80
Rheb	Ras homolog enriched in brain	ENSMUSG00000028945	39802	59,80
Ddhd1	DDHD domain containing 1	ENSMUSG00000037697	64970	59,80
Mtx1	metaxin 1	ENSMUSG00000064068	18008	59,80
Ptms	parathymosin	ENSMUSG00000030122	4266	59,73
Pfn1	profilin 1	ENSMUSG00000018293	2795	59,73
		ENSMUSG00000025786	47046	59,66
Tcof1	Treacher Collins Franceschetti syndrome 1, homolog	ENSMUSG00000024613	35217	59,66
Rbm28	RNA binding motif protein 28	ENSMUSG00000029701	41434	59,66
Pck2	phosphoenolpyruvate carboxykinase 2 (mitochondrial)	ENSMUSG00000040618	9752	59,59
Pnpla3	patatin-like phospholipase domain containing 3	ENSMUSG00000041653	21697	59,59
Eif4b	eukaryotic translation initiation factor 4B	ENSMUSG00000058655	23400	59,59
Spock2	sparc/osteonectin, cwcv and kazal-like domains proteoglycan 2	ENSMUSG00000058297	28980	59,51
Zfp113	zinc finger protein 113	ENSMUSG00000037007	16043	59,51
Serpinb6a	serine (or cysteine) peptidase inhibitor, clade B, member 6a	ENSMUSG00000060147	84877	59,51
Smap1	small ArfGAP 1	ENSMUSG00000026155	77472	59,51
Zc3h12b	zinc finger CCCH-type containing 12B	ENSMUSG00000035045	220960	59,51
Ier3ip1	immediate early response 3 interacting protein 1	ENSMUSG00000090000	11589	59,44
Zfp423	zinc finger protein 423	ENSMUSG00000045333	297786	59,44
Idh2	isocitrate dehydrogenase 2 (NADP+), mitochondrial	ENSMUSG00000030541	20547	59,44
		ENSMUSG00000027519	47584	59,44
Dph5	DPH5 homolog (S. cerevisiae)	ENSMUSG00000033554	41160	59,37
Gstz1	glutathione transferase zeta 1 (maleylacetoacetate isomerase)	ENSMUSG00000021033	17015	59,37
		ENSMUSG00000024563	64152	59,37
Rtp2	receptor transporter protein 2	ENSMUSG00000047531	5240	59,30
Eif1a	eukaryotic translation initiation factor 1A	ENSMUSG00000057561	12522	59,30
Zhx2	zinc fingers and homeoboxes 2	ENSMUSG00000071757	145169	59,30
Zfp362	zinc finger protein 362	ENSMUSG00000028799	32958	59,22
Wdr52	WD repeat domain 52	ENSMUSG00000071550	87633	59,22
Tle4	transducin-like enhancer of split 4, homolog of Drosophila E(spl)	ENSMUSG00000024642	149980	59,22
Swap70	SWA-70 protein	ENSMUSG00000031015	61801	59,22
Morn4	MORN repeat containing 4	ENSMUSG00000049670	11432	59,15
Cdip1	cell death inducing Trp53 target 1	ENSMUSG00000004071	39945	59,15

Get4	golgi to ER traffic protein 4 homolog (S. cerevisiae)	ENSMUSG00000025858	17728	59,15
Stk35	serine/threonine kinase 35	ENSMUSG00000037885	31771	59,15
Gbas	glioblastoma amplified sequence	ENSMUSG00000029432	33265	59,15
		ENSMUSG00000033792	97651	59,15
Lrig2	leucine-rich repeats and immunoglobulin-like domains 2	ENSMUSG00000032913	57873	59,08
Sos2	son of sevenless homolog 2 (Drosophila)	ENSMUSG00000034801	98091	59,08
Meis1	Meis homeobox 1	ENSMUSG00000020160	138529	59,08
Csrp1	cysteine and glycine-rich protein 1	ENSMUSG00000026421	32172	59,08
9130401M01Rik	RIKEN cDNA 9130401M01 gene	ENSMUSG00000022362	54150	59,01
Rab1b	RAB1B, member RAS oncogene family	ENSMUSG00000024870	7790	59,01
Hat1	histone aminotransferase 1	ENSMUSG00000027018	52665	59,01
Emd	emerin	ENSMUSG00000001964	6862	59,01
Slc22a18	solute carrier family 22 (organic cation transporter), member 18	ENSMUSG00000000154	25614	58,93
Sesn1	sestrin 1	ENSMUSG00000038332	97851	58,93
Myo18a	myosin XVIIIA	ENSMUSG00000000631	102735	58,93
Map2k3	mitogen-activated protein kinase kinase 3	ENSMUSG00000018932	20779	58,93
		ENSMUSG00000020961	149846	58,93
Opn3	opsin 3	ENSMUSG00000026525	30356	58,93
Ergic3	ERGIC and golgi 3	ENSMUSG00000005881	10235	58,86
Cyth2	cytohesin 2	ENSMUSG00000003269	7945	58,79
Cttn	cortactin	ENSMUSG00000031078	35277	58,79
Ahdc1	AT hook, DNA binding motif, containing 1	ENSMUSG00000037692	66851	58,79
Phka1	phosphorylase kinase alpha 1	ENSMUSG00000034055	130272	58,79
Pold3	polymerase (DNA-directed), delta 3, accessory subunit	ENSMUSG00000030726	39455	58,72
Ptpn9	protein tyrosine phosphatase, non-receptor type 9	ENSMUSG00000032290	67838	58,72
Evi5	ecotropic viral integration site 5	ENSMUSG00000011831	130313	58,72
Celsr1	cadherin, EGF LAG seven-pass G-type receptor 1 (flamingo homolog, Drosophila)	ENSMUSG00000016028	135020	58,72
Ttc9c	tetratricopeptide repeat domain 9C	ENSMUSG00000071660	10259	58,64
Seh1l	SEH1-like (S. cerevisiae	ENSMUSG00000079614	20586	58,64
Ndufs5	NADH dehydrogenase (ubiquinone) Fe-S protein 5	ENSMUSG00000028648	5493	58,64
Cyb5b	cytochrome b5 type B	ENSMUSG00000031924	36810	58,64
Cramp1l	Crm, cramped-like (Drosophila)	ENSMUSG00000038002	54003	58,64
Mlf2	myeloid leukemia factor 2	ENSMUSG00000030120	4751	58,57
Snx1	sorting nexin 1	ENSMUSG00000032382	38455	58,57
		ENSMUSG00000037280	37564	58,57
Mlh3	mutL homolog 3 (E coli)	ENSMUSG00000021245	36063	58,57
Hist1h2ab	histone cluster 1, H2ab	ENSMUSG00000061615	506	58,50
Uncx	UNC homeobox	ENSMUSG00000029546	4686	58,50
Uhrf1	ubiquitin-like, containing PHD and RING finger domains, 1	ENSMUSG00000001228	20166	58,50
Snrpd3	small nuclear ribonucleoprotein D3	ENSMUSG00000020180	17399	58,50
Ap3b2	adaptor-related protein complex 3, beta 2 subunit	ENSMUSG00000062444	33527	58,50
Stxbp4	syntaxin binding protein 4	ENSMUSG00000020546	161593	58,50

Edem3	ER degradation enhancer, mannosidase alpha-like 3	ENSMUSG00000043019	66955	58,50
Atg7	autophagy related 7	ENSMUSG00000030314	217518	58,50
Cdc14b	CDC14 cell division cycle 14B	ENSMUSG00000033102	82429	58,50
Dcun1d5	DCN1, defective in cullin neddylation 1, domain containing 5 (S. cerevisiae)	ENSMUSG00000032002	22466	58,43
Runx1t1	runt-related transcription factor 1; translocated to, 1 (cyclin D-related)	ENSMUSG00000006586	150214	58,43
Cwh43	cell wall biogenesis 43 C-terminal homolog (S. cerevisiae)	ENSMUSG00000029154	47360	58,43
Tlk1	tousled-like kinase 1	ENSMUSG00000041997	113322	58,43
Tuba4a	tubulin, alpha 4A	ENSMUSG00000026202	4280	58,35
1700025G04Rik	RIKEN cDNA 1700025G04 gene	ENSMUSG00000032666	205797	58,35
Hook2	hook homolog 2 (Drosophila)	ENSMUSG00000052566	12770	58,35
Cobl	cordon-bleu WH2 repeat	ENSMUSG00000020173	228353	58,35
Rexo1	REX1, RNA exonuclease 1 homolog (S. cerevisiae)	ENSMUSG00000047417	20639	58,28
		ENSMUSG00000020156	17470	58,28
		ENSMUSG00000030788	44026	58,28
Fam188a	family with sequence similarity 188, member A	ENSMUSG00000026767	72207	58,28
Rev1	REV1 homolog (S. cerevisiae)	ENSMUSG00000026082	76876	58,28
U2af2	U2 small nuclear ribonucleoprotein auxiliary factor (U2AF) 2	ENSMUSG00000030435	17803	58,21
		ENSMUSG00000037295	23290	58,14
Kif21b	kinesin family member 21B	ENSMUSG00000041642	46610	58,14
Mef2b	myocyte enhancer factor 2B	ENSMUSG00000002345	27990	58,06
Fam126b	family with sequence similarity 126, member B	ENSMUSG00000038174	63518	58,06
Fam189a2	family with sequence similarity 189, member A2	ENSMUSG00000071604	58269	58,06
Fbxo28	F-box protein 28	ENSMUSG00000047539	28505	57,99
Dopey1	dopey family member 1	ENSMUSG00000034973	70400	57,99
Rnf213	ring finger protein 213	ENSMUSG00000070327	94319	57,99
Nek11	NIMA (never in mitosis gene a)-related expressed kinase 11	ENSMUSG00000035032	233058	57,99
Mta1	metastasis associated 1	ENSMUSG00000021144	38929	57,92
		ENSMUSG00000058298	94111	57,92
		ENSMUSG00000078622	18937	57,92
Ascl4	achaete-scute complex homolog 4 (Drosophila)	ENSMUSG00000085111	17716	57,85
Ypel3	yippee-like 3 (Drosophila)	ENSMUSG00000042675	3560	57,85
Myef2	myelin basic protein expression factor 2, repressor	ENSMUSG00000027201	39034	57,85
Zfyve27	zinc finger, FYVE domain containing 27	ENSMUSG00000018820	30640	57,85
Smc5	structural maintenance of chromosomes 5	ENSMUSG00000024943	67457	57,85
Raf1	v-raf-leukemia viral oncogene 1	ENSMUSG00000000441	58569	57,85
Prickle2	prickle homolog 2 (Drosophila)	ENSMUSG00000030020	335254	57,85
		ENSMUSG00000040824	3014	57,77
Slc12a7	solute carrier family 12, member 7	ENSMUSG00000017756	53047	57,77
		ENSMUSG00000046324	40202	57,77
Slc12a6	solute carrier family 12, member 6	ENSMUSG00000027130	97339	57,77
Oprm1	opioid receptor, mu 1	ENSMUSG00000000766	279693	57,77

Dtx1	deltex 1 homolog (Drosophila)	ENSMUSG00000029603	31726	57,70
		ENSMUSG00000094962	10226	57,70
Cbfa2t2	core-binding factor, runt domain, alpha subunit 2, translocated to, 2 (human)	ENSMUSG00000038533	102876	57,70
Prr13	proline rich 13	ENSMUSG00000023048	3779	57,63
Zfp326	zinc finger protein 326	ENSMUSG00000029290	39254	57,63
Ctdspl2	CTD (carboxy-terminal domain, RNA polymerase II, polypeptide A) small phosphatase like 2	ENSMUSG00000033411	57642	57,63
		ENSMUSG00000019578	7984	57,56
		ENSMUSG00000000194	67233	57,56
2900026A02Rik	RIKEN cDNA 2900026A02 gene	ENSMUSG00000051339	77029	57,56
Gm12185	predicted gene 12185	ENSMUSG00000048852	87571	57,48
		ENSMUSG00000020070	36988	57,48
Csmd1	CUB and Sushi multiple domains 1	ENSMUSG00000060924	1643050	57,48
Agr3	anterior gradient 3	ENSMUSG00000036231	24117	57,48
Ccdc57	coiled-coil domain containing 57	ENSMUSG00000048445	106344	57,41
Emc6	ER membrane protein complex subunit 6	ENSMUSG00000047260	1519	57,34
Eef2k	eukaryotic elongation factor-2 kinase	ENSMUSG00000035064	64389	57,34
Tmem205	transmembrane protein 205	ENSMUSG00000040883	6526	57,34
D1Ertd622e	DNA segment, Chr 1, ERATO Doi 622, expressed	ENSMUSG00000044768	18172	57,34
Unk	unkempt homolog (Drosophila)	ENSMUSG00000020770	30893	57,34
Hpse2	heparanase 2	ENSMUSG00000074852	599365	57,34
Sh3glb2	SH3-domain GRB2-like endophilin B2	ENSMUSG00000026860	14529	57,19
Dusp18	dual specificity phosphatase 18	ENSMUSG00000047205	6057	57,19
Kctd1	potassium channel tetramerisation domain containing 1	ENSMUSG00000036225	182762	57,19
Stxbp5	syntaxin binding protein 5 (tomosyn)	ENSMUSG00000019790	145533	57,19
Ccnd3	cyclin D3	ENSMUSG00000034165	94641	57,19
Prrg2	proline-rich Gla (G-carboxyglutamic acid) polypeptide 2	ENSMUSG00000007837	9065	57,12
Tomm40	translocase of outer mitochondrial membrane 40 homolog (yeast)	ENSMUSG00000002984	14126	57,12
Txnl1	thioredoxin-like 1	ENSMUSG00000024583	29559	57,12
Cpd	carboxypeptidase D	ENSMUSG00000020841	68595	57,12
Alg14	asparagine-linked glycosylation 14	ENSMUSG00000039887	70195	57,12
Zfp386	zinc finger protein 386 (Kruppel-like)	ENSMUSG00000042063	15637	57,05
Arf6	ADP-ribosylation factor 6	ENSMUSG00000044147	3830	57,05
Zhx3	zinc fingers and homeoboxes 3	ENSMUSG00000035877	129072	57,05
Wsb1	WD repeat and SOCS box-containing 1	ENSMUSG00000017677	15300	57,05
2810025M15Rik	RIKEN cDNA 2810025M15 gene	ENSMUSG00000049881	7884	56,98
Rad9b	RAD9 homolog B	ENSMUSG00000038569	31011	56,98
Pgrmc2	progesterone receptor membrane component 2	ENSMUSG00000049940	16721	56,90
		ENSMUSG00000027263	28117	56,90
Klhl14	kelch-like 14	ENSMUSG00000042514	104342	56,90
Atp7b	ATPase, Cu++ transporting, beta polypeptide	ENSMUSG00000006567	67521	56,83

Nadk	NAD kinase	ENSMUSG00000029063	28624	56,76
Mbd2	methyl-CpG binding domain protein 2	ENSMUSG00000024513	57943	56,76
Siah1a	seven in absentia 1A	ENSMUSG00000036840	22067	56,76
Zfp687	zinc finger protein 687	ENSMUSG00000019338	8859	56,76
Wdr37	WD repeat domain 37	ENSMUSG00000021147	68942	56,76
3110043O21Rik	RIKEN cDNA 3110043O21 gene	ENSMUSG00000028300	34891	56,69
Rab36	RAB36, member RAS oncogene family	ENSMUSG00000020175	17691	56,69
		ENSMUSG00000043556	351966	56,69
		ENSMUSG00000078879	48564	56,69
Ranbp17	RAN binding protein 17	ENSMUSG00000040594	301952	56,69
Coa5	cytochrome C oxidase assembly factor 5	ENSMUSG00000026112	13020	56,61
Ufc1	ubiquitin-fold modifier conjugating enzyme 1	ENSMUSG00000062963	6462	56,61
Map1lc3a	microtubule-associated protein 1 light chain 3 alpha	ENSMUSG00000027602	1777	56,61
Rhpn2	rhophilin, Rho GTPase binding protein 2	ENSMUSG00000030494	58111	56,61
		ENSMUSG00000039640	4453	56,54
Copz1	coatomer protein complex, subunit zeta 1	ENSMUSG00000060992	26952	56,54
Nif3l1	Ngg1 interacting factor 3-like 1 (S. pombe)	ENSMUSG00000026036	36666	56,54
Golga2	golgi autoantigen, golgin subfamily a, 2	ENSMUSG00000002546	20538	56,54
Cog7	component of oligomeric golgi complex 7	ENSMUSG00000034951	58873	56,54
Map4k5	mitogen-activated protein kinase kinase kinase kinase 5	ENSMUSG00000034761	89451	56,54
Tmem2	transmembrane protein 2	ENSMUSG00000024754	79986	56,47
		ENSMUSG00000039523	33539	56,47
Naa30	N(alpha)-acetyltransferase 30, NatC catalytic subunit	ENSMUSG00000036282	18806	56,47
Rad54l2	RAD54 like 2 (S. cerevisiae)	ENSMUSG00000040661	101132	56,47
Clptm1	cleft lip and palate associated transmembrane protein 1	ENSMUSG00000002981	33450	56,47
Ces1e	carboxylesterase 1E	ENSMUSG00000061959	28402	56,47
Dennd2a	DENN/MADD domain containing 2A	ENSMUSG00000038456	95490	56,47
Lifr	leukemia inhibitory factor receptor	ENSMUSG00000054263	67931	56,47
Coq10b	coenzyme Q10 homolog B (S. cerevisiae)	ENSMUSG00000025981	19932	56,47
Rnf165	ring finger protein 165	ENSMUSG00000025427	109038	56,40
Fuca1	fucosidase, alpha-L- 1, tissue	ENSMUSG00000028673	19577	56,40
0610010F05Rik	RIKEN cDNA 0610010F05 gene	ENSMUSG00000042208	68679	56,32
Cd247	CD247 antigen	ENSMUSG00000005763	88596	56,32
Oma1	OMA1 homolog, zinc metallopeptidase (S. cerevisiae)	ENSMUSG00000035069	58057	56,32
		ENSMUSG00000031754	19252	56,25
Nelfcd	negative elongation factor complex member C/D, Th1l	ENSMUSG00000016253	11699	56,25
Wbp4	WW domain binding protein 4	ENSMUSG00000022023	21332	56,25
		ENSMUSG00000019868	50806	56,25
		ENSMUSG00000002881	43386	56,25

Hs3st1	heparan sulfate (glucosamine) 3-O-sulfotransferase 1	ENSMUSG00000051022	141541	56,25
Rhob	ras homolog gene family, member B	ENSMUSG00000054364	2223	56,25
Slc10a1	solute carrier family 10 (sodium/bile acid cotransporter family), member 1	ENSMUSG00000021135	14895	56,25
Trappc3	trafficking protein particle complex 3	ENSMUSG00000028847	13559	56,18
Pex19	peroxisomal biogenesis factor 19	ENSMUSG00000003464	9739	56,18
Podn	podocan	ENSMUSG00000028600	81655	56,18
Cyc1	cytochrome c-1	ENSMUSG00000022551	2412	56,11
Tmem65	transmembrane protein 65	ENSMUSG00000062373	41262	56,11
Ado	2-aminoethanethiol (cysteamine) dioxygenase	ENSMUSG00000057134	4444	56,11
Serhl	serine hydrolase-like	ENSMUSG00000058586	27169	56,11
Eya2	eyes absent 2 homolog (Drosophila)	ENSMUSG00000017897	176696	56,11
Sptssa	serine palmitoyltransferase, small subunit A	ENSMUSG00000044408	11199	56,04
Tecpr1	tectonin beta-propeller repeat containing 1	ENSMUSG00000066621	29174	56,04
Pkd1l1	polycystic kidney disease 1 like 1	ENSMUSG00000046634	146559	56,04
Aktip	thymoma viral proto-oncogene 1 interacting protein	ENSMUSG00000031667	22425	56,04
Smad1	SMAD family member 1	ENSMUSG00000031681	61124	56,04
Spata7	spermatogenesis associated 7	ENSMUSG00000021007	41659	56,04
Cdkl4	cyclin-dependent kinase-like 4	ENSMUSG00000033966	40285	56,04
Amph	amphiphysin	ENSMUSG00000021314	202543	56,04
Pls1	plastin 1 (I-isoform)	ENSMUSG00000049493	92670	56,04
Chst8	carbohydrate (N-acetylgalactosamine 4-0) sulfotransferase 8	ENSMUSG00000060402	138244	55,96
Denr	density-regulated protein	ENSMUSG00000023106	21658	55,96
Kpna6	karyopherin (importin) alpha 6	ENSMUSG00000003731	28788	55,96
Fkbp3	FK506 binding protein 3	ENSMUSG00000020949	11509	55,96
Sgsm1	small G protein signaling modulator 1	ENSMUSG00000042216	67567	55,96
Rsph3b	radial spoke 3B homolog (Chlamydomonas)	ENSMUSG00000023806	43641	55,96
Kdm1a	lysine (K)-specific demethylase 1A	ENSMUSG00000036940	52184	55,96
Stx7	syntaxin 7	ENSMUSG00000019998	39643	55,89
		ENSMUSG00000078970	51690	55,89
Mdc1	mediator of DNA damage checkpoint 1	ENSMUSG00000061607	18156	55,89
Capns1	calpain, small subunit 1	ENSMUSG00000001794	8223	55,82
Dhdds	dehydrodolichyl diphosphate synthase	ENSMUSG00000012117	31891	55,82
Sec16a	SEC16 homolog A (S. cerevisiae)	ENSMUSG00000026924	35786	55,82
Anxa11	annexin A11	ENSMUSG00000021866	44622	55,82
Eps8l1	EPS8-like 1	ENSMUSG00000006154	18731	55,82
Mgat5b	mannoside acetylglucosaminyltransferase 5, isoenzyme B	ENSMUSG00000043857	68086	55,75

Slc16a11	solute carrier family 16 (monocarboxylic acid transporters), member 11	ENSMUSG00000040938	3666	55,75
Arhgap40	Rho GTPase activating protein 40	ENSMUSG00000074625	37967	55,75
Scfd1	Sec1 family domain containing 1	ENSMUSG00000020952	72517	55,67
Cnp	2',3'-cyclic nucleotide 3' phosphodiesterase	ENSMUSG00000006782	16826	55,60
Sec13	SEC13 homolog (S. cerevisiae)	ENSMUSG00000030298	12677	55,60
Txndc9	thioredoxin domain containing 9	ENSMUSG00000058407	13480	55,60
Usp30	ubiquitin specific peptidase 30	ENSMUSG00000029592	23174	55,60
Hs2st1	heparan sulfate 2-O-sulfotransferase 1	ENSMUSG00000040151	139079	55,60
Fkbp2	FK506 binding protein 2	ENSMUSG00000056629	2721	55,53
Snrpb	small nuclear ribonucleoprotein B	ENSMUSG00000027404	7990	55,53
Mvb12b	multivesicular body subunit 12B	ENSMUSG00000038740	157994	55,53
Txndc15	thioredoxin domain containing 15	ENSMUSG00000021497	11572	55,53
Rbbp7	retinoblastoma binding protein 7	ENSMUSG00000031353	18691	55,53
Fam120b	family with sequence similarity 120, member B	ENSMUSG00000014763	37335	55,53
Cenpc1	centromere protein C1	ENSMUSG00000029253	53541	55,53
Mcm3ap	minichromosome maintenance deficient 3 (S. cerevisiae) associated protein	ENSMUSG00000001150	46886	55,53
Akap10	A kinase (PRKA) anchor protein 10	ENSMUSG00000047804	58946	55,53
Smpd1	sphingomyelin phosphodiesterase 1, acid lysosomal	ENSMUSG00000037049	4029	55,46
Gcc1	golgi coiled coil 1	ENSMUSG00000029708	12300	55,46
Ssh1	slingshot homolog 1 (Drosophila)	ENSMUSG00000042121	56801	55,46
Wrn	Werner syndrome homolog (human)	ENSMUSG00000031583	151144	55,46
Cplx2	complexin 2	ENSMUSG00000025867	12569	55,38
Mrpl43	mitochondrial ribosomal protein L43	ENSMUSG00000025208	1429	55,38
Fam53a	family with sequence similarity 53, member A	ENSMUSG00000037339	29285	55,38
Fam58b	family with sequence similarity 58, member B	ENSMUSG00000049489	1223	55,38
Ccnl2	cyclin L2	ENSMUSG00000029068	12055	55,38
Ppp4r1	protein phosphatase 4, regulatory subunit 1	ENSMUSG00000061950	59354	55,38
Abcc3	ATP-binding cassette, sub-family C (CFTR/MRP), member 3	ENSMUSG00000020865	49703	55,38
Sars	seryl-aminoacyl-tRNA synthetase	ENSMUSG00000068739	20345	55,31
Ube2m	ubiquitin-conjugating enzyme E2M	ENSMUSG00000005575	3156	55,31
Ikbkg	inhibitor of kappaB kinase gamma	ENSMUSG00000004221	60565	55,31
Nudt16l1	nudix (nucleoside diphosphate linked moiety X)-type motif 16-like 1	ENSMUSG00000022516	1910	55,24
Ndufa12	NADH dehydrogenase (ubiquinone) 1 alpha subcomplex, 12	ENSMUSG00000020022	22489	55,24
		ENSMUSG00000019710	4167	55,24
		ENSMUSG00000093575	34870	55,24
		ENSMUSG00000009907	32658	55,24
Tmod1	tropomodulin 1	ENSMUSG00000028328	77098	55,24
Ripk1	receptor (TNFRSF)-interacting serine-threonine kinase 1	ENSMUSG00000021408	32808	55,24

Sin3b	transcriptional regulator, SIN3B (yeast)	ENSMUSG00000031622	34916	55,17
Wtap	Wilms' tumour 1-associating protein	ENSMUSG00000060475	25751	55,17
4933434E20Rik	RIKEN cDNA 4933434E20 gene	ENSMUSG00000027942	11706	55,17
Rin3	Ras and Rab interactor 3	ENSMUSG00000044456	107808	55,17
Thoc3	THO complex 3	ENSMUSG00000025872	10013	55,09
Nrcam	neuron-glia-CAM-related cell adhesion molecule	ENSMUSG00000020598	272962	55,09
Zc3h4	zinc finger CCCH-type containing 4	ENSMUSG00000059273	36499	55,09
Lemd3	LEM domain containing 3	ENSMUSG00000048661	55920	55,09
Ccdc138	coiled-coil domain containing 138	ENSMUSG00000038010	78273	55,09
Cpeb3	cytoplasmic polyadenylation element binding protein 3	ENSMUSG00000039652	187311	55,09
Ptbp1	polypyrimidine tract binding protein 1	ENSMUSG00000006498	10345	55,02
1700003M02Rik	RIKEN cDNA 1700003M02 gene	ENSMUSG00000028294	41648	55,02
Nhsl1	NHS-like 1	ENSMUSG00000039835	143571	55,02
Glrx3	glutaredoxin 3	ENSMUSG00000031068	30947	54,95
Rcbtb1	regulator of chromosome condensation (RCC1) and BTB (POZ) domain containing protein 1	ENSMUSG00000035469	36057	54,95
Rab12	RAB12, member RAS oncogene family	ENSMUSG00000023460	25206	54,95
Syt14	synaptotagmin XIV	ENSMUSG00000016200	138392	54,95
Aspscr1	alveolar soft part sarcoma chromosome region, candidate 1 (human)	ENSMUSG00000025142	36475	54,95
Syt4	synaptotagmin IV	ENSMUSG00000024261	9608	54,88
Wdr73	WD repeat domain 73	ENSMUSG00000025722	10547	54,88
9030624J02Rik	RIKEN cDNA 9030624J02 gene	ENSMUSG00000030982	102741	54,88
Mipol1	mirror-image polydactyly gene 1 homolog (human)	ENSMUSG00000047022	266773	54,88
Zfp64	zinc finger protein 64	ENSMUSG00000027551	62257	54,80
Snx30	sorting nexin family member 30	ENSMUSG00000028385	98898	54,80
Lrpprc	leucine-rich PPR-motif containing	ENSMUSG00000024120	85543	54,80
Vps53	vacuolar protein sorting 53 (yeast)	ENSMUSG00000017288	133422	54,80
Osgin1	oxidative stress induced growth inhibitor 1	ENSMUSG00000074063	12133	54,80
Nipal1	NIPA-like domain containing 1	ENSMUSG00000067219	23283	54,80
Fbxo24	F-box protein 24	ENSMUSG00000089984	16500	54,73
Lphn1	latrophilin 1	ENSMUSG00000013033	41850	54,73
Erp44	endoplasmic reticulum protein 44	ENSMUSG00000028343	86236	54,73
Myb	myeloblastosis oncogene	ENSMUSG00000019982	36055	54,73
Tgs1	trimethylguanosine synthase homolog (S. cerevisiae)	ENSMUSG00000028233	41745	54,73
D630045J12Rik	RIKEN cDNA D630045J12 gene	ENSMUSG00000063455	130836	54,73
Xrcc4	X-ray repair complementing defective repair in Chinese hamster cells 4	ENSMUSG00000021615	315582	54,66
Dhrs4	dehydrogenase/reductase (SDR family) member 4	ENSMUSG00000022210	11581	54,59
Ppap2c	phosphatidic acid phosphatase type 2C	ENSMUSG00000052151	7358	54,59
1110037F02Rik	RIKEN cDNA 1110037F02 gene	ENSMUSG00000040720	64727	54,59

Hbp1	high mobility group box transcription factor 1	ENSMUSG00000002996	24282	54,59
Hmgxb4	HMG box domain containing 4	ENSMUSG00000034518	38623	54,59
Wrap73	WD repeat containing, antisense to Trp73	ENSMUSG00000029029	25049	54,51
Gtf2e2	general transcription factor II E, polypeptide 2 (beta subunit)	ENSMUSG00000031585	45260	54,51
		ENSMUSG00000049119	464134	54,51
L1cam	L1 cell adhesion molecule	ENSMUSG00000031391	42328	54,51
Rabggtb	RAB geranylgeranyl transferase, b subunit	ENSMUSG00000038975	5678	54,44
Cops7a	COP9 (constitutive photomorphogenic) homolog, subunit 7a (Arabidopsis thaliana)	ENSMUSG00000030127	7126	54,44
Gabpb1	GA repeat binding protein, beta 1	ENSMUSG00000027361	48896	54,44
Mrps6	mitochondrial ribosomal protein S6	ENSMUSG00000039680	53958	54,44
Ttc13	tetratricopeptide repeat domain 13	ENSMUSG00000037300	50649	54,44
Tmem211	transmembrane protein 211	ENSMUSG00000066964	12355	54,44
Cartpt	CART prepropeptide	ENSMUSG00000021647	2200	54,37
Rsl1d1	ribosomal L1 domain containing 1	ENSMUSG00000005846	10362	54,37
Psmc6	proteasome (prosome, macropain) 26S subunit, ATPase, 6	ENSMUSG00000021832	19248	54,37
Bspry	B-box and SPRY domain containing	ENSMUSG00000028392	17246	54,37
Nup153	nucleoporin 153	ENSMUSG00000021374	48036	54,37
Efcab11	EF-hand calcium binding domain 11	ENSMUSG00000021176	165912	54,37
Gtf2a1	general transcription factor II A, 1	ENSMUSG00000020962	34572	54,30
		ENSMUSG00000031916	10401	54,30
Capn12	calpain 12	ENSMUSG00000054083	11930	54,30
Wdr61	WD repeat domain 61	ENSMUSG00000061559	19785	54,30
Camk2g	calcium/calmodulin-dependent protein kinase II gamma	ENSMUSG00000021820	59214	54,30
Brk1	BRICK1, SCAR/WAVE actin-nucleating complex subunit	ENSMUSG00000033940	12177	54,22
Gmeb1	glucocorticoid modulatory element binding protein 1	ENSMUSG00000028901	40578	54,22
Cmtm7	CKLF-like MARVEL transmembrane domain containing 7	ENSMUSG00000032436	25157	54,22
		ENSMUSG00000065947	297	54,22
Spg11	spastic paraplegia 11	ENSMUSG00000033396	64867	54,22
Ipo5	importin 5	ENSMUSG00000030662	36849	54,15
Cers2	ceramide synthase 2	ENSMUSG00000015714	8797	54,15
Gabpb2	GA repeat binding protein, beta 2	ENSMUSG00000038766	36151	54,15
Tmem30b	transmembrane protein 30B	ENSMUSG00000034435	3282	54,15
Fam117b	family with sequence similarity 117, member B	ENSMUSG00000041040	72341	54,15
Dusp10	dual specificity phosphatase 10	ENSMUSG00000039384	62335	54,15
Pgls	6-phosphogluconolactonase	ENSMUSG00000031807	8917	54,08
Atp5e	ATP synthase, H+ transporting, mitochondrial F1 complex, epsilon subunit	ENSMUSG00000016252	3034	54,08
Ago1	argonaute RISC catalytic subunit 1	ENSMUSG00000041530	33572	54,08
Sdc4	syndecan 4	ENSMUSG00000017009	19641	54,08
Zfp710	zinc finger protein 710	ENSMUSG00000048897	67938	54,08

Fbxo42	F-box protein 42	ENSMUSG00000028920	56150	54,08
		ENSMUSG00000026275	30636	54,01
Nup88	nucleoporin 88	ENSMUSG00000040667	26916	54,01
Klhl8	kelch-like 8	ENSMUSG00000029312	49287	54,01
Gm5506	predicted gene 5506	ENSMUSG00000059040	3044	53,93
Ercc5	excision repair cross-complementing rodent repair deficiency, complementation group 5	ENSMUSG00000026048	33414	53,93
Xpot	exportin, tRNA (nuclear export receptor for tRNAs)	ENSMUSG00000034667	38937	53,93
Ppp2r2d	protein phosphatase 2, regulatory subunit B, delta isoform	ENSMUSG00000041769	36979	53,93
Anapc1	anaphase promoting complex subunit 1	ENSMUSG00000014355	77288	53,93
Rsc1a1	regulatory solute carrier protein, family 1, member 1	ENSMUSG00000040715	39857	53,93
Lrrc28	leucine rich repeat containing 28	ENSMUSG00000030556	131826	53,86
Hdac1	histone deacetylase 1	ENSMUSG00000028800	26610	53,86
Ogt	O-linked N-acetylglucosamine (GlcNAc) transferase (UDP-N-acetylglucosamine:polypeptide-N-acetylglucosaminyl transferase)	ENSMUSG00000034160	44292	53,86
Adamdec1	ADAM-like, decysin 1	ENSMUSG00000022057	18699	53,86
Pex6	peroxisomal biogenesis factor 6	ENSMUSG00000002763	14079	53,79
		ENSMUSG00000047879	49688	53,79
Tmem87b	transmembrane protein 87B	ENSMUSG00000014353	36144	53,79
		ENSMUSG00000054814	69968	53,79
Btg3	B cell translocation gene 3	ENSMUSG00000022863	17333	53,79
Mocos	molybdenum cofactor sulfurase	ENSMUSG00000039616	47866	53,79
Tmprss2	transmembrane protease, serine 2	ENSMUSG00000000385	46512	53,79
Nutf2	nuclear transport factor 2	ENSMUSG00000008450	19769	53,72
		ENSMUSG00000040843	22022	53,72
Ttll11	tubulin tyrosine ligase-like family, member 11	ENSMUSG00000026885	228673	53,72
D15Ertd621e	DNA segment, Chr 15, ERATO Doi 621, expressed	ENSMUSG00000037119	42334	53,72
Pigu	phosphatidylinositol glycan anchor biosynthesis, class U	ENSMUSG00000038383	79188	53,72
Rnf111	ring finger 111	ENSMUSG00000032217	78297	53,72
Slc1a1	solute carrier family 1 (neuronal/epithelial high affinity glutamate transporter, system Xag), member 1	ENSMUSG00000024935	78912	53,72
Snrnp48	small nuclear ribonucleoprotein 48 (U11/U12)	ENSMUSG00000021431	22725	53,72
Sirt3	sirtuin 3	ENSMUSG00000025486	18640	53,72
Pou6f1	POU domain, class 6, transcription factor 1	ENSMUSG00000009739	24667	53,64
Cap1	CAP, adenylate cyclase-associated protein 1 (yeast)	ENSMUSG00000028656	27010	53,64
Rbm34	RNA binding motif protein 34	ENSMUSG00000033931	23905	53,64
Ocrl	oculocerebrorenal syndrome of Lowe	ENSMUSG00000001173	53482	53,64
Map3k15	mitogen-activated protein kinase kinase kinase 15	ENSMUSG00000031303	134919	53,57

Cdc37	cell division cycle 37	ENSMUSG00000019471	11366	53,57
Sh2b1	SH2B adaptor protein 1	ENSMUSG00000030733	8137	53,57
Cpne1	copine I	ENSMUSG00000074643	40115	53,57
Zfp532	zinc finger protein 532	ENSMUSG00000042439	109214	53,57
Chm	choroidermia	ENSMUSG00000025531	144923	53,57
Fosl2	fos-like antigen 2	ENSMUSG00000029135	21360	53,57
Tmem14c	transmembrane protein 14C	ENSMUSG00000021361	6326	53,50
Bphl	biphenyl hydrolase-like (serine hydrolase, breast epithelial mucin-associated antigen)	ENSMUSG00000038286	36434	53,50
Commd7	COMM domain containing 7	ENSMUSG00000056941	15849	53,43
Marf1	meiosis arrest female 1	ENSMUSG00000060657	50109	53,43
Commd8	COMM domain containing 8	ENSMUSG00000029213	11184	53,43
Zak	sterile alpha motif and leucine zipper containing kinase AZK	ENSMUSG00000004085	156974	53,43
Dnah11	dynein, axonemal, heavy chain 11	ENSMUSG00000018581	321062	53,43
Fem1a	feminization 1 homolog a (C. elegans)	ENSMUSG00000043683	6816	53,35
Crls1	cardiolipin synthase 1	ENSMUSG00000027357	20120	53,35
Uimc1	ubiquitin interaction motif containing 1	ENSMUSG00000025878	72421	53,35
Nyap2	neuronal tyrosine-phophorylated phosphoinositide 3-kinase adaptor 2	ENSMUSG00000054976	264815	53,35
Pik3c2b	phosphoinositide-3-kinase, class 2, beta polypeptide	ENSMUSG00000026447	63021	53,35
Casc4	cancer susceptibility candidate 4	ENSMUSG00000060227	69251	53,28
Lphn2	latrophilin 2	ENSMUSG00000028184	173731	53,28
Ydjc	YdjC homolog (bacterial)	ENSMUSG00000041774	16081	53,21
Npc2	Niemann Pick type C2	ENSMUSG00000021242	18553	53,21
Lmnb1	lamin B1	ENSMUSG00000024590	45612	53,21
Cstb	cystatin B	ENSMUSG00000005054	1950	53,14
Sdhd	succinate dehydrogenase complex, subunit D, integral membrane protein	ENSMUSG00000000171	7512	53,14
Ap3s2	adaptor-related protein complex 3, sigma 2 subunit	ENSMUSG00000063801	45309	53,14
Purg	purine-rich element binding protein G	ENSMUSG00000049184	31144	53,14
Tmem261	transmembrane protein 261	ENSMUSG00000028398	952	53,06
Fbxw9	F-box and WD-40 domain protein 9	ENSMUSG00000008167	7070	53,06
Spag7	sperm associated antigen 7	ENSMUSG00000018287	5646	53,06
		ENSMUSG00000078941	15071	53,06
Cpt2	carnitine palmitoyltransferase 2	ENSMUSG00000028607	19630	53,06
Chchd6	coiled-coil-helix-coiled-coil-helix domain containing 6	ENSMUSG00000030086	212507	53,06
Anxa3	annexin A3	ENSMUSG00000029484	52584	53,06
Nipa2	non imprinted in Prader-Willi/Angelman syndrome 2 homolog (human)	ENSMUSG00000030452	31190	53,06
Lsm5	LSM5 homolog, U6 small nuclear RNA associated (S. cerevisiae)	ENSMUSG00000091625	3630	52,99
Ncoa7	nuclear receptor coactivator 7	ENSMUSG00000039697	157524	52,99
		ENSMUSG00000021036	83298	52,99
Lgr5	leucine rich repeat containing G protein coupled receptor 5	ENSMUSG00000020140	137470	52,99

Tbpl1	TATA box binding protein-like 1	ENSMUSG00000071359	28060	52,92
Gm10644	predicted gene 10644	ENSMUSG00000074219	22877	52,92
Grb10	growth factor receptor bound protein 10	ENSMUSG00000020176	108176	52,92
Chrac1	chromatin accessibility complex 1	ENSMUSG00000068391	7163	52,92
Arhgef3	Rho guanine nucleotide exchange factor (GEF) 3	ENSMUSG00000021895	165866	52,85
Pip4k2b	phosphatidylinositol-5-phosphate 4-kinase, type II, beta	ENSMUSG00000018547	29548	52,85
Stk40	serine/threonine kinase 40	ENSMUSG00000042608	37073	52,85
Mrpl46	mitochondrial ribosomal protein L46	ENSMUSG00000030612	8296	52,77
		ENSMUSG00000040928	41723	52,77
		ENSMUSG00000010554	57696	52,77
Krit1	KRIT1, ankyrin repeat containing	ENSMUSG00000000600	41351	52,77
St3gal3	ST3 beta-galactoside alpha-2,3-sialyltransferase 3	ENSMUSG00000028538	202761	52,77
Zbtb10	zinc finger and BTB domain containing 10	ENSMUSG00000069114	34732	52,77
Mmp24	matrix metallopeptidase 24	ENSMUSG00000027612	43025	52,70
Papss1	3'-phosphoadenosine 5'-phosphosulfate synthase 1	ENSMUSG00000028032	78903	52,70
Cldn9	claudin 9	ENSMUSG00000066720	1443	52,70
		ENSMUSG00000004865	34874	52,70
Gmeb2	glucocorticoid modulatory element binding protein 2	ENSMUSG00000038705	36587	52,70
		ENSMUSG00000004665	7479	52,70
Jakmip1	janus kinase and microtubule interacting protein 1	ENSMUSG00000063646	119667	52,63
Ormdl3	ORM1-like 3 (S. cerevisiae)	ENSMUSG00000038150	6113	52,63
Tmem256	transmembrane protein 256	ENSMUSG00000070394	1105	52,63
Pigk	phosphatidylinositol glycan anchor biosynthesis, class K	ENSMUSG00000039047	266309	52,63
Smurf1	SMAD specific E3 ubiquitin protein ligase 1	ENSMUSG00000038780	89353	52,63
Igf2bp3	insulin-like growth factor 2 mRNA binding protein 3	ENSMUSG00000029814	129734	52,63
Pcbp3	poly(rC) binding protein 3	ENSMUSG00000001120	200031	52,56
Mef2c	myocyte enhancer factor 2C	ENSMUSG00000005583	163046	52,56
Fbxl18	F-box and leucine-rich repeat protein 18	ENSMUSG00000066640	28476	52,56
		ENSMUSG00000028394	2420	52,48
Sult1d1	sulfotransferase family 1D, member 1	ENSMUSG00000029273	14383	52,48
Gprc5c	G protein-coupled receptor, family C, group 5, member C	ENSMUSG00000051043	21466	52,48
Kif19a	kinesin family member 19A	ENSMUSG00000010021	25352	52,48
Secisbp2	SECIS binding protein 2	ENSMUSG00000035139	32348	52,48
Cdan1	congenital dyserythropoietic anemia, type I (human)	ENSMUSG00000027284	133975	52,48
Dph6	diphthamine biosynthesis 6	ENSMUSG00000057147	138549	52,48
Tsc1	tuberous sclerosis 1	ENSMUSG00000026812	49940	52,48
Aasdh	aminoadipate-semialdehyde dehydrogenase	ENSMUSG00000055923	31856	52,48
		ENSMUSG00000053877	24350	52,48
		ENSMUSG00000025429	87463	52,48

Anapc13	anaphase promoting complex subunit 13	ENSMUSG00000035048	7946	52,41
5730455P16Rik	RIKEN cDNA 5730455P16 gene	ENSMUSG00000057181	17543	52,41
Lrguk	leucine-rich repeats and guanylate kinase domain containing	ENSMUSG00000056215	104587	52,41
		ENSMUSG00000018040	9369	52,41
Ikzf5	IKAROS family zinc finger 5	ENSMUSG00000040167	21870	52,41
Fnbp1	formin binding protein 1	ENSMUSG00000075415	115803	52,41
Vps41	vacuolar protein sorting 41 (yeast)	ENSMUSG00000041236	149518	52,41
Tbc1d4	TBC1 domain family, member 4	ENSMUSG00000033083	166832	52,41
Sec14l1	SEC14-like 1 (S. cerevisiae)	ENSMUSG00000020823	44101	52,34
Dram2	DNA-damage regulated autophagy modulator 2	ENSMUSG00000027900	27153	52,34
Mtor	mechanistic target of rapamycin (serine/threonine kinase)	ENSMUSG00000028991	109073	52,34
Orc4	origin recognition complex, subunit 4	ENSMUSG00000026761	47454	52,34
Cyth3	cytohesin 3	ENSMUSG00000018001	87804	52,34
Rer1	RER1 retention in endoplasmic reticulum 1 homolog (S. cerevisiae)	ENSMUSG00000029048	12273	52,27
Gstm5	glutathione S-transferase, mu 5	ENSMUSG00000004032	2866	52,27
Ddx21	DEAD (Asp-Glu-Ala-Asp) box polypeptide 21	ENSMUSG00000020075	22051	52,27
B3gnt2	UDP-GlcNAc:betaGal beta-1,3-N-acetylglucosaminyltransferase 2	ENSMUSG00000051650	26215	52,27
Ttf1	transcription termination factor, RNA polymerase I	ENSMUSG00000026803	27395	52,27
Cebpb	CCAAT/enhancer binding protein (C/EBP), beta	ENSMUSG00000056501	1504	52,19
Snx13	sorting nexin 13	ENSMUSG00000020590	100281	52,19
Epb4.1l4a	erythrocyte protein band 4.1-like 4a	ENSMUSG00000024376	210880	52,19
AI597468	expressed sequence AI597468	ENSMUSG00000060935	15121	52,12
Gcn1l1	GCN1 general control of amino-acid synthesis 1-like 1 (yeast)	ENSMUSG00000041638	57403	52,12
Cnppd1	cyclin Pas1/PHO80 domain containing 1	ENSMUSG00000033159	7815	52,05
Smad5	SMAD family member 5	ENSMUSG00000021540	39368	52,05
Lats1	large tumor suppressor	ENSMUSG00000040021	35250	52,05
		ENSMUSG00000033751	3961	52,05
		ENSMUSG00000026618	44671	52,05
Galnt2	UDP-N-acetyl-alpha-D-galactosamine:polypeptide N-acetylgalactosaminyltransferase 2	ENSMUSG00000089704	114334	52,05
		ENSMUSG00000048186	84309	52,05
Cyhr1	cysteine and histidine rich 1	ENSMUSG00000053929	16723	51,98
Ift74	intraflagellar transport 74	ENSMUSG00000028576	78739	51,98
Osbpl11	oxysterol binding protein-like 11	ENSMUSG00000022807	58241	51,98
Thoc1	THO complex 1	ENSMUSG00000024287	37305	51,98
Spred2	sprouty-related, EVH1 domain containing 2	ENSMUSG00000045671	99652	51,98
Mical3	microtubule associated monooxygenase, calponin and LIM domain containing 3	ENSMUSG00000003178	74369	51,98
Prnd	prion protein dublet	ENSMUSG00000027338	46174	51,90
		ENSMUSG00000005983	13511	51,90
Exoc8	exocyst complex component 8	ENSMUSG00000074030	7406	51,90

Arhgef17	Rho guanine nucleotide exchange factor (GEF) 17	ENSMUSG00000032875	62416	51,90
Lrrc48	leucine rich repeat containing 48	ENSMUSG00000056598	41013	51,90
Gpr137b	G protein-coupled receptor 137B	ENSMUSG00000021306	36002	51,90
B4galt6	UDP-Gal:betaGlcNAc beta 1,4-galactosyltransferase, polypeptide 6	ENSMUSG00000056124	61806	51,90
Creb5	cAMP responsive element binding protein 5	ENSMUSG00000053007	122624	51,90
Zdhhc5	zinc finger, DHHC domain containing 5	ENSMUSG00000034075	27211	51,83
		ENSMUSG00000006728	4387	51,83
Maats1	MYCBP-associated, testis expressed 1	ENSMUSG00000022805	44390	51,83
Rad17	RAD17 homolog (S. pombe)	ENSMUSG00000021635	33895	51,83
E2f6	E2F transcription factor 6	ENSMUSG00000057469	15779	51,83
		ENSMUSG00000036054	45690	51,83
Fbxo7	F-box protein 7	ENSMUSG00000001786	29902	51,83
Lrrc8a	leucine rich repeat containing 8A	ENSMUSG00000007476	44434	51,76
Arel1	apoptosis resistant E3 ubiquitin protein ligase 1	ENSMUSG00000042350	52753	51,76
Atp6v1c1	ATPase, H+ transporting, lysosomal V1 subunit C1	ENSMUSG00000022295	30540	51,76
Tor1aip2	torsin A interacting protein 2	ENSMUSG00000050565	33459	51,76
Ehd4	EH-domain containing 4	ENSMUSG00000027293	65432	51,76
Acaca	acetyl-Coenzyme A carboxylase alpha	ENSMUSG00000020532	271980	51,76
Slc25a36	solute carrier family 25, member 36	ENSMUSG00000032449	35561	51,76
Idua	iduronidase, alpha-L-	ENSMUSG00000033540	24227	51,76
		ENSMUSG00000026527	431395	51,76
Mrps25	mitochondrial ribosomal protein S25	ENSMUSG00000014551	14509	51,69
Fam63a	family with sequence similarity 63, member A	ENSMUSG00000038712	14822	51,69
Epb4.1l3	erythrocyte protein band 4.1-like 3	ENSMUSG00000024044	133176	51,69
Immp1l	IMP1 inner mitochondrial membrane peptidase-like (S. cerevisiae)	ENSMUSG00000042670	60921	51,69
		ENSMUSG00000007038	4701	51,61
Ift172	intraflagellar transport 172	ENSMUSG00000038564	37835	51,61
Tmcc2	transmembrane and coiled-coil domains 2	ENSMUSG00000042066	34967	51,54
Lyrm5	LYR motif containing 5	ENSMUSG00000040370	5806	51,54
Zfp523	zinc finger protein 523	ENSMUSG00000024220	28680	51,54
Pgam5	phosphoglycerate mutase family member 5	ENSMUSG00000029500	10784	51,54
Sat1	spermidine/spermine N1-acetyl transferase 1	ENSMUSG00000025283	3318	51,54
Rdm1	RAD52 motif 1	ENSMUSG00000010362	8906	51,54
		ENSMUSG00000071037	367414	51,54
Supt3	suppressor of Ty 3	ENSMUSG00000038954	342139	51,54
Phax	phosphorylated adaptor for RNA export	ENSMUSG00000008301	25270	51,47
Lipo1	lipase, member O1	ENSMUSG00000024766	244212	51,47
Rhbg	Rhesus blood group-associated B glycoprotein	ENSMUSG00000001417	44337	51,47

Slirp	SRA stem-loop interacting RNA binding protein	ENSMUSG00000021040	8311	51,47
Copb1	coatomer protein complex, subunit beta 1	ENSMUSG00000030754	39122	51,47
Gm10222	predicted gene 10222	ENSMUSG00000067736	297	51,47
Tmem248	transmembrane protein 248	ENSMUSG00000053094	24022	51,47
Thbs1	thrombospondin 1	ENSMUSG00000040152	15258	51,47
Ccndbp1	cyclin D-type binding-protein 1	ENSMUSG00000023572	8502	51,40
Mnt	max binding protein	ENSMUSG00000000282	14806	51,40
Zbtb46	zinc finger and BTB domain containing 46	ENSMUSG00000027583	71665	51,40
Nudt5	nudix (nucleoside diphosphate linked moiety X)-type motif 5	ENSMUSG00000025817	26877	51,40
Riok3	RIO kinase 3	ENSMUSG00000024404	28518	51,40
Gpd1l	glycerol-3-phosphate dehydrogenase 1-like	ENSMUSG00000050627	34647	51,40
Lbr	lamin B receptor	ENSMUSG00000004880	27086	51,40
Recql	RecQ protein-like	ENSMUSG00000030243	36746	51,40
Cdon	cell adhesion molecule-related/down-regulated by oncogenes	ENSMUSG00000038119	86524	51,40
Tex33	testis expressed 33	ENSMUSG00000062154	17513	51,40
Serpinh1	serine (or cysteine) peptidase inhibitor, clade H, member 1	ENSMUSG00000070436	7856	51,32
Thap11	THAP domain containing 11	ENSMUSG00000036442	1848	51,32
Epn2	epsin 2	ENSMUSG00000001036	62439	51,32
Dpm1	dolichol-phosphate (beta-D) mannosyltransferase 1	ENSMUSG00000078919	21544	51,32
Rmdn3	regulator of microtubule dynamics 3	ENSMUSG00000070730	20034	51,32
Dag1	dystroglycan 1	ENSMUSG00000039952	57970	51,32
Cybrd1	cytochrome b reductase 1	ENSMUSG00000027015	25004	51,32
D19Ertd737e	DNA segment, Chr 19, ERATO Doi 737, expressed	ENSMUSG00000057858	28112	51,25
Polr2h	polymerase (RNA) II (DNA directed) polypeptide H	ENSMUSG00000021018	4603	51,25
Derl1	Der1-like domain family, member 1	ENSMUSG00000022365	22917	51,25
Tcte1	t-complex-associated testis expressed 1	ENSMUSG00000023949	19246	51,18
Elk4	ELK4, member of ETS oncogene family	ENSMUSG00000026436	25006	51,18
Cops3	COP9 (constitutive photomorphogenic) homolog, subunit 3 (Arabidopsis thaliana)	ENSMUSG00000019373	22044	51,18
Aida	axin interactor, dorsalization associated	ENSMUSG00000042901	27442	51,18
Ncaph2	non-SMC condensin II complex, subunit H2	ENSMUSG00000008690	17108	51,18
Rab8b	RAB8B, member RAS oncogene family	ENSMUSG00000036943	76034	51,18
Ube2q2	ubiquitin-conjugating enzyme E2Q (putative) 2	ENSMUSG00000032307	58676	51,18
Fam49b	family with sequence similarity 49, member B	ENSMUSG00000022378	131352	51,18
Prnp	prion protein	ENSMUSG00000079037	28502	51,11
		ENSMUSG00000031934	40318	51,11

		ENSMUSG00000038648	114892	51,11
Tmem56	transmembrane protein 56	ENSMUSG00000028132	81338	51,11
Wnt4	wingless-related MMTV integration site 4	ENSMUSG00000036856	22238	51,11
Tmem151b	transmembrane protein 151B	ENSMUSG00000096847	7738	51,03
Eif4e2	eukaryotic translation initiation factor 4E member 2	ENSMUSG00000026254	26575	51,03
Aco2	aconitase 2, mitochondrial	ENSMUSG00000022477	42825	51,03
Spata5	spermatogenesis associated 5	ENSMUSG00000027722	159201	51,03
Scn8a	sodium channel, voltage-gated, type VIII, alpha	ENSMUSG00000023033	175247	51,03
Hiat1	hippocampus abundant gene transcript 1	ENSMUSG00000089911	50097	51,03
		ENSMUSG00000055137	836878	51,03
Gid4	GID complex subunit 4, VID24 homolog (S. cerevisiae)	ENSMUSG00000018415	33783	50,96
Zfp397	zinc finger protein 397	ENSMUSG00000024276	9984	50,96
Mpdu1	mannose-P-dolichol utilization defect 1	ENSMUSG00000018761	5946	50,96
Tmem55a	transmembrane protein 55A	ENSMUSG00000028221	51101	50,96
Wdr60	WD repeat domain 60	ENSMUSG00000042050	55976	50,96
Acbd6	acyl-Coenzyme A binding domain containing 6	ENSMUSG00000033701	129114	50,96
Pdgfc	platelet-derived growth factor, C polypeptide	ENSMUSG00000028019	177625	50,96
Ctsl	cathepsin L	ENSMUSG00000021477	7093	50,89
Surf2	surfeit gene 2	ENSMUSG00000014873	3817	50,89
Stx1a	syntaxin 1A (brain)	ENSMUSG00000007207	27619	50,89
Heatr3	HEAT repeat containing 3	ENSMUSG00000031657	34173	50,89
		ENSMUSG00000031731	82805	50,89
		ENSMUSG00000025739	2236	50,82
Itm2c	integral membrane protein 2C	ENSMUSG00000026223	14395	50,82
Otub1	OTU domain, ubiquitin aldehyde binding 1	ENSMUSG00000024767	8115	50,82
		ENSMUSG00000020783	32435	50,82
Ankrd13a	ankyrin repeat domain 13a	ENSMUSG00000041870	31524	50,82
Cenpe	centromere protein E	ENSMUSG00000045328	60978	50,82
Omp	olfactory marker protein	ENSMUSG00000074006	2143	50,82
Dut	deoxyuridine triphosphatase	ENSMUSG00000027203	11419	50,74
Gnl3l	guanine nucleotide binding protein-like 3 (nucleolar)-like	ENSMUSG00000025266	34182	50,74
Abi2	abl-interactor 2	ENSMUSG00000026782	71544	50,74
Stim1	stromal interaction molecule 1	ENSMUSG00000030987	169025	50,74
Insig1	insulin induced gene 1	ENSMUSG00000045294	7300	50,67
Gpsm2	G-protein signalling modulator 2 (AGS3-like, C. elegans)	ENSMUSG00000027883	43550	50,67
Cnot3	CCR4-NOT transcription complex, subunit 3	ENSMUSG00000035632	15819	50,67
Dnttip2	deoxynucleotidyltransferase, terminal, interacting protein 2	ENSMUSG00000039756	10856	50,67
Arhgef18	rho/rac guanine nucleotide exchange factor (GEF) 18	ENSMUSG00000004568	63596	50,67
Bcas2	breast carcinoma amplified sequence 2	ENSMUSG00000005687	7512	50,60
Pde6a	phosphodiesterase 6A, cGMP-specific, rod, alpha	ENSMUSG00000024575	69443	50,60

		ENSMUSG00000048222	13833	50,60
Phrf1	PHD and ring finger domains 1	ENSMUSG00000038611	33967	50,60
Ppip5k1	diphosphoinositol pentakisphosphate kinase 1	ENSMUSG00000033526	44836	50,60
5330417C22Rik	RIKEN cDNA 5330417C22 gene	ENSMUSG00000040412	80833	50,60
Pacsin2	protein kinase C and casein kinase substrate in neurons 2	ENSMUSG00000016664	89000	50,60
Mmp15	matrix metallopeptidase 15	ENSMUSG00000031790	22026	50,53
Cep89	centrosomal protein 89	ENSMUSG00000023072	41655	50,53
Cdk7	cyclin-dependent kinase 7	ENSMUSG00000069089	33916	50,53
Vps33a	vacuolar protein sorting 33A (yeast)	ENSMUSG00000029434	44252	50,53
Cul4a	cullin 4A	ENSMUSG00000031446	42320	50,53
Ndufb6	NADH dehydrogenase (ubiquinone) 1 beta subcomplex, 6	ENSMUSG00000071014	8831	50,45
Suv420h2	suppressor of variegation 4-20 homolog 2 (Drosophila)	ENSMUSG00000059851	7400	50,45
Gopc	golgi associated PDZ and coiled-coil motif containing	ENSMUSG00000019861	45101	50,45
		ENSMUSG00000030059	23859	50,45
Prpf6	PRP6 pre-mRNA splicing factor 6 homolog (yeast)	ENSMUSG00000002455	63793	50,45
Armc8	armadillo repeat containing 8	ENSMUSG00000032468	90509	50,45
Nrg1	neuregulin 1	ENSMUSG00000062991	100176	50,45
Prdm15	PR domain containing 15	ENSMUSG00000014039	60384	50,45
Gnb2	guanine nucleotide binding protein (G protein), beta 2	ENSMUSG00000029713	5384	50,38
Bnip3	BCL2/adenovirus E1B interacting protein 3	ENSMUSG00000078566	18684	50,38
Chp1	calcineurin-like EF hand protein 1	ENSMUSG00000014077	39331	50,38
Osbpl2	oxysterol binding protein-like 2	ENSMUSG00000039050	43375	50,38
Gpt2	glutamic pyruvate transaminase (alanine aminotransferase) 2	ENSMUSG00000031700	34985	50,31
Pde6d	phosphodiesterase 6D, cGMP-specific, rod, delta	ENSMUSG00000026239	39636	50,31
Rnf185	ring finger protein 185	ENSMUSG00000020448	36382	50,31
Rnf103	ring finger protein 103	ENSMUSG00000052656	31127	50,31
Snx24	sorting nexing 24	ENSMUSG00000024535	145162	50,31
Lypla2	lysophospholipase 2	ENSMUSG00000028670	4403	50,24
Manea	mannosidase, endo-alpha	ENSMUSG00000040520	22386	50,24
Ppic	peptidylprolyl isomerase C	ENSMUSG00000024538	11667	50,24
Gzf1	GDNF-inducible zinc finger protein 1	ENSMUSG00000027439	11927	50,24
Vbp1	von Hippel-Lindau binding protein 1	ENSMUSG00000031197	20644	50,16
Tbrg1	transforming growth factor beta regulated gene 1	ENSMUSG00000011114	8131	50,16
Mfap3	microfibrillar-associated protein 3	ENSMUSG00000020522	15152	50,16
Strn4	striatin, calmodulin binding protein 4	ENSMUSG00000030374	25043	50,16
		ENSMUSG00000020668	41363	50,16
Xrcc6	X-ray repair complementing defective repair in Chinese hamster cells 6	ENSMUSG00000022471	52251	50,16
Ate1	arginyltransferase 1	ENSMUSG00000030850	128436	50,16
Psen1	presenilin 1	ENSMUSG00000019969	46997	50,16
Prkdc	protein kinase, DNA activated, catalytic polypeptide	ENSMUSG00000022672	204370	50,16

Cse1l	chromosome segregation 1-like (S. cerevisiae)	ENSMUSG00000002718	40350	50,16
Zfp398	zinc finger protein 398	ENSMUSG00000062519	32597	50,16
Cenpo	centromere protein O	ENSMUSG00000020652	38291	50,09
Yipf1	Yip1 domain family, member 1	ENSMUSG00000057375	45461	50,09
C2cd2	C2 calcium-dependent domain containing 2	ENSMUSG00000045975	67424	50,09
Ccdc41	coiled-coil domain containing 41	ENSMUSG00000020024	101547	50,09
Wdr20a	WD repeat domain 20A	ENSMUSG00000037957	57080	50,09
Rhod	ras homolog gene family, member D	ENSMUSG00000041845	13974	50,09
Rnf114	ring finger protein 114	ENSMUSG00000006418	23529	50,02
Disp1	dispatched homolog 1 (Drosophila)	ENSMUSG00000030768	135266	50,02
Rpia	ribose 5-phosphate isomerase A	ENSMUSG00000053604	26513	50,02
Micalcl	MICAL C-terminal like	ENSMUSG00000030771	44797	50,02
Ing2	inhibitor of growth family, member 2	ENSMUSG00000063049	8379	50,02
Ift52	intraflagellar transport 52	ENSMUSG00000017858	28788	49,95
		ENSMUSG00000019579	8177	49,95
Xpnpep1	X-prolyl aminopeptidase (aminopeptidase P) 1, soluble	ENSMUSG00000025027	96798	49,95
F2r	coagulation factor II (thrombin) receptor	ENSMUSG00000048376	16656	49,95
Ap4s1	adaptor-related protein complex AP-4, sigma 1	ENSMUSG00000020955	47974	49,87
Slc35a4	solute carrier family 35, member A4	ENSMUSG00000033272	4280	49,87
Ywhag	tyrosine 3-monooxygenase/tryptophan 5-monooxygenase activation protein, gamma polypeptide	ENSMUSG00000051391	26208	49,87
Pygo2	pygopus 2	ENSMUSG00000047824	4915	49,87
H2-T24	histocompatibility 2, T region locus 24	ENSMUSG00000053835	14866	49,87
Tcp11	t-complex protein 11	ENSMUSG00000062859	13893	49,87
Nfx1	nuclear transcription factor, X-box binding 1	ENSMUSG00000028423	55088	49,87
Tsga10	testis specific 10	ENSMUSG00000060771	110727	49,87
Inpp5f	inositol polyphosphate-5-phosphatase F	ENSMUSG00000042105	85098	49,80
		ENSMUSG00000045210	29761	49,80
Tiparp	TCDD-inducible poly(ADP-ribose) polymerase	ENSMUSG00000034640	26736	49,80
Ap2s1	adaptor-related protein complex 2, sigma 1 subunit	ENSMUSG00000008036	10885	49,73
Foxj1	forkhead box J1	ENSMUSG00000034227	4696	49,73
Agpat1	1-acylglycerol-3-phosphate O-acyltransferase 1 (lysophosphatidic acid acyltransferase, alpha)	ENSMUSG00000034254	9188	49,73
Crb3	crumbs homolog 3 (Drosophila)	ENSMUSG00000044279	6814	49,73
Fam160b1	family with sequence similarity 160, member B1	ENSMUSG00000033478	28586	49,73
Mllt1	myeloid/lymphoid or mixed-lineage leukemia (trithorax homolog, Drosophila); translocated to, 1	ENSMUSG00000024212	42777	49,73

Fam149b	family with sequence similarity 149, member B	ENSMUSG00000039599	35328	49,73
Plxdc1	plexin domain containing 1	ENSMUSG00000017417	63207	49,66
Slc3a2	solute carrier family 3 (activators of dibasic and neutral amino acid transport), member 2	ENSMUSG00000010095	16488	49,66
		ENSMUSG00000028057	14212	49,66
Fam160a2	family with sequence similarity 160, member A2	ENSMUSG00000044465	28844	49,66
Snx12	sorting nexin 12	ENSMUSG00000046032	124778	49,66
Ccdc132	coiled-coil domain containing 132	ENSMUSG00000001376	105139	49,66
Kif24	kinesin family member 24	ENSMUSG00000028438	74143	49,66
Arhgef26	Rho guanine nucleotide exchange factor (GEF) 26	ENSMUSG00000036885	123878	49,66
Rpl28	ribosomal protein L28	ENSMUSG00000030432	1689	49,58
Ankra2	ankyrin repeat, family A (RFXANK-like), 2	ENSMUSG00000021661	11593	49,58
Zfx	zinc finger protein X-linked	ENSMUSG00000079509	49072	49,58
Gnptab	N-acetylglucosamine-1-phosphate transferase, alpha and beta subunits	ENSMUSG00000035311	68198	49,58
Ctdspl	CTD (carboxy-terminal domain, RNA polymerase II, polypeptide A) small phosphatase-like	ENSMUSG00000047409	117546	49,58
Dcp1b	DCP1 decapping enzyme homolog B (S. cerevisiae)	ENSMUSG00000041477	46363	49,58
Nek10	NIMA (never in mitosis gene a)- related kinase 10	ENSMUSG00000042567	185979	49,58
Mapk9	mitogen-activated protein kinase 9	ENSMUSG00000020366	39671	49,51
		ENSMUSG00000033799	45073	49,51
Safb2	scaffold attachment factor B2	ENSMUSG00000042625	23621	49,51
2810403A07Rik	RIKEN cDNA 2810403A07 gene	ENSMUSG00000028060	27140	49,51
Sp4	trans-acting transcription factor 4	ENSMUSG00000025323	66508	49,51
1700001K19Rik	RIKEN cDNA 1700001K19 gene	ENSMUSG00000056508	14931	49,51
Usp39	ubiquitin specific peptidase 39	ENSMUSG00000056305	26507	49,44
		ENSMUSG00000079941	390	49,44
Timp3	tissue inhibitor of metalloproteinase 3	ENSMUSG00000020044	49135	49,44
		ENSMUSG00000063632	2013	49,44
Gse1	genetic suppressor element 1	ENSMUSG00000031822	350855	49,44
Rnf13	ring finger protein 13	ENSMUSG00000036503	99168	49,44
Arpp21	cyclic AMP-regulated phosphoprotein, 21	ENSMUSG00000032503	170848	49,44
2700049A03Rik	RIKEN cDNA 2700049A03 gene	ENSMUSG00000034601	106456	49,44
Ston1	stonin 1	ENSMUSG00000033855	64903	49,44
Coasy	Coenzyme A synthase	ENSMUSG00000001755	4055	49,37
Heatr6	HEAT repeat containing 6	ENSMUSG00000000976	30059	49,37
Rhov	ras homolog gene family, member V	ENSMUSG00000034226	2072	49,37
Pea15a	phosphoprotein enriched in astrocytes 15A	ENSMUSG00000013698	10077	49,37
Fbxo34	F-box protein 34	ENSMUSG00000037536	59402	49,37
Ankib1	ankyrin repeat and IBR domain containing 1	ENSMUSG00000040351	113110	49,37
		ENSMUSG00000021879	198430	49,37
1110058L19Rik	RIKEN cDNA 1110058L19 gene	ENSMUSG00000026154	9718	49,29

Dnajc11	DnaJ (Hsp40) homolog, subfamily C, member 11	ENSMUSG00000039768	48447	49,29
Dcun1d3	DCN1, defective in cullin neddylation 1, domain containing 3 (S. cerevisiae)	ENSMUSG00000048787	43129	49,29
Rnpep	arginyl aminopeptidase (aminopeptidase B)	ENSMUSG00000041926	21373	49,22
Zfp667	zinc finger protein 667	ENSMUSG00000054893	21305	49,22
Mepce	methylphosphate capping enzyme	ENSMUSG00000029726	4810	49,22
Ncoa3	nuclear receptor coactivator 3	ENSMUSG00000027678	80607	49,22
Rasef	RAS and EF hand domain containing	ENSMUSG00000043003	76131	49,22
Nlrc4	NLR family, CARD domain containing 4	ENSMUSG00000039193	32814	49,15
Apmap	adipocyte plasma membrane associated protein	ENSMUSG00000033096	25488	49,15
Fnta	farnesyltransferase, CAAX box, alpha	ENSMUSG00000015994	16873	49,15
Atp6v1h	ATPase, H+ transporting, lysosomal V1 subunit H	ENSMUSG00000033793	79377	49,15
Zfyve26	zinc finger, FYVE domain containing 26	ENSMUSG00000066440	63936	49,15
Klhl5	kelch-like 5	ENSMUSG00000054920	60621	49,08
Gipc1	GIPC PDZ domain containing family, member 1	ENSMUSG00000019433	12111	49,08
Pnrc1	proline-rich nuclear receptor coactivator 1	ENSMUSG00000040128	44741	49,08
Gtf2h1	general transcription factor II H, polypeptide 1	ENSMUSG00000006599	27698	49,08
L3mbtl3	l(3)mbt-like 3 (Drosophila)	ENSMUSG00000039089	100718	49,08
		ENSMUSG00000032925	315648	49,00
		ENSMUSG00000030882	81238	49,00
Rpl31	ribosomal protein L31	ENSMUSG00000073702	4058	49,00
Zfp369	zinc finger protein 369	ENSMUSG00000021514	25408	49,00
Vdac1	voltage-dependent anion channel 1	ENSMUSG00000020402	28538	48,93
Tbc1d9b	TBC1 domain family, member 9B	ENSMUSG00000036644	41390	48,93
		ENSMUSG00000036257	90974	48,93
Abhd17b	abhydrolase domain containing 17B	ENSMUSG00000047368	32329	48,93
Slc38a6	solute carrier family 38, member 6	ENSMUSG00000044712	67271	48,93
Serpinb11	serine (or cysteine) peptidase inhibitor, clade B (ovalbumin), member 11	ENSMUSG00000026327	18547	48,93
Cdk1	cyclin-dependent kinase 1	ENSMUSG00000019942	16304	48,86
Tmeff1	transmembrane protein with EGF-like and two follistatin-like domains 1	ENSMUSG00000028347	77958	48,86
Slc25a44	solute carrier family 25, member 44	ENSMUSG00000050144	14642	48,86
Rnf40	ring finger protein 40	ENSMUSG00000030816	14908	48,86
Lzts2	leucine zipper, putative tumor suppressor 2	ENSMUSG00000035342	11929	48,86
Ube2o	ubiquitin-conjugating enzyme E2O	ENSMUSG00000020802	43708	48,86
Fam105b	family with sequence similarity 105, member B	ENSMUSG00000046034	24775	48,86
Mbd3	methyl-CpG binding domain protein 3	ENSMUSG00000035478	7012	48,86

Grin2a	glutamate receptor, ionotropic, NMDA2A (epsilon 1)	ENSMUSG00000059003	417212	48,86
1700037H04Rik	RIKEN cDNA 1700037H04 gene	ENSMUSG00000027327	13757	48,79
4930427A07Rik	RIKEN cDNA 4930427A07 gene	ENSMUSG00000037466	9036	48,79
Thoc6	THO complex 6 homolog (Drosophila)	ENSMUSG00000041319	5269	48,79
Armc2	armadillo repeat containing 2	ENSMUSG00000071324	103453	48,79
Tdrd7	tudor domain containing 7	ENSMUSG00000035517	69428	48,79
Agap3	ArfGAP with GTPase domain, ankyrin repeat and PH domain 3	ENSMUSG00000023353	49871	48,71
Tceb2	transcription elongation factor B (SIII), polypeptide 2	ENSMUSG00000055839	4370	48,71
Prune	prune homolog (Drosophila)	ENSMUSG00000015711	28403	48,71
Pacsin3	protein kinase C and casein kinase substrate in neurons 3	ENSMUSG00000027257	8726	48,71
Senp1	SUMO1/sentrin specific peptidase 1	ENSMUSG00000033075	55001	48,71
Ntan1	N-terminal Asn amidase	ENSMUSG00000022681	16201	48,71
Med15	mediator complex subunit 15	ENSMUSG00000012114	71710	48,71
Ctif	CBP80/20-dependent translation initiation factor	ENSMUSG00000052928	266473	48,71
Ankrd32	ankyrin repeat domain 32	ENSMUSG00000021597	92386	48,71
Wfdc18	WAP four-disulfide core domain 18	ENSMUSG00000000983	2334	48,64
Ap3m2	adaptor-related protein complex 3, mu 2 subunit	ENSMUSG00000031539	18301	48,64
Mrpl40	mitochondrial ribosomal protein L40	ENSMUSG00000022706	4750	48,64
Tor1b	torsin family 1, member B	ENSMUSG00000026848	6057	48,64
Slc35f1	solute carrier family 35, member F1	ENSMUSG00000038602	421090	48,64
Rab21	RAB21, member RAS oncogene family	ENSMUSG00000020132	25729	48,57
Ctnnbl1	catenin, beta like 1	ENSMUSG00000027649	154214	48,57
Eftud1	elongation factor Tu GTP binding domain containing 1	ENSMUSG00000038563	129239	48,57
Spns1	spinster homolog 1	ENSMUSG00000030741	7391	48,50
Pgk1	phosphoglycerate kinase 1	ENSMUSG00000062070	16600	48,50
Sbds	Shwachman-Bodian-Diamond syndrome homolog (human)	ENSMUSG00000025337	9800	48,50
		ENSMUSG00000054477	417020	48,50
Ercc6l2	excision repair cross-complementing rodent repair deficiency, complementation group 6 like 2	ENSMUSG00000021470	85063	48,50
Commd10	COMM domain containing 10	ENSMUSG00000042705	129131	48,50
Rpl18	ribosomal protein L18	ENSMUSG00000059070	5379	48,42
		ENSMUSG00000026277	13821	48,42
Herpud2	HERPUD family member 2	ENSMUSG00000008429	43652	48,42
Msantd2	Myb/SANT-like DNA-binding domain containing 2	ENSMUSG00000042138	34828	48,42
Fgd4	FYVE, RhoGEF and PH domain containing 4	ENSMUSG00000022788	183633	48,42
Stoml2	stomatin (Epb7.2)-like 2	ENSMUSG00000028455	4021	48,35
		ENSMUSG00000027332	20956	48,35
		ENSMUSG00000094790	501	48,35

Zscan21	zinc finger and SCAN domain containing 21	ENSMUSG00000037017	17363	48,35
Eif4enif1	eukaryotic translation initiation factor 4E nuclear import factor 1	ENSMUSG00000020454	42197	48,35
Pcyt2	phosphate cytidylyltransferase 2, ethanolamine	ENSMUSG00000025137	7850	48,35
Cep78	centrosomal protein 78	ENSMUSG00000041491	29216	48,35
Tet2	tet methylcytosine dioxygenase 2	ENSMUSG00000040943	80712	48,35
Slc26a7		ENSMUSG00000040569	119376	48,35
Cwc15	CWC15 homolog (S. cerevisiae)	ENSMUSG00000004096	9961	48,28
Rap2a	RAS related protein 2a	ENSMUSG00000051615	28734	48,28
Crk	v-crk sarcoma virus CT10 oncogene homolog (avian)	ENSMUSG00000017776	27650	48,28
Otud5	OTU domain containing 5	ENSMUSG00000031154	35263	48,28
Ift20	intraflagellar transport 20	ENSMUSG00000001105	5377	48,28
Fign	fidgetin	ENSMUSG00000075324	126532	48,28
Zbtb22	zinc finger and BTB domain containing 22	ENSMUSG00000051390	3421	48,21
Hdac3	histone deacetylase 3	ENSMUSG00000024454	19145	48,21
Trim28	tripartite motif-containing 28	ENSMUSG00000005566	6884	48,21
Mlf1	myeloid leukemia factor 1	ENSMUSG00000048416	25907	48,21
Pus10	pseudouridylate synthase 10	ENSMUSG00000020280	67203	48,21
Cpne3	copine III	ENSMUSG00000028228	50855	48,21
Ifnar2	interferon (alpha and beta) receptor 2	ENSMUSG00000022971	32807	48,21
Palm	paralemmin	ENSMUSG00000035863	27325	48,13
Atmin	ATM interactor	ENSMUSG00000047388	17053	48,13
Rsph1	radial spoke head 1 homolog (Chlamydomonas)	ENSMUSG00000024033	22338	48,13
Pofut1	protein O-fucosyltransferase 1	ENSMUSG00000046020	28715	48,13
Abhd12	abhydrolase domain containing 12	ENSMUSG00000032046	72249	48,13
Eda	ectodysplasin-A	ENSMUSG00000059327	425157	48,13
Arhgdig	Rho GDP dissociation inhibitor (GDI) gamma	ENSMUSG00000073433	11561	48,06
Cnpy2	canopy 2 homolog (zebrafish)	ENSMUSG00000025381	4729	48,06
March2	membrane-associated ring finger (C3HC4) 2	ENSMUSG00000079557	32979	48,06
Ipo9	importin 9	ENSMUSG00000041879	48188	48,06
Prkcsh	protein kinase C substrate 80K-H	ENSMUSG00000003402	11185	48,06
Akt2	thymoma viral proto-oncogene 2	ENSMUSG00000004056	49275	48,06
Rassf3	Ras association (RalGDS/AF-6) domain family member 3	ENSMUSG00000025795	65901	48,06
Tbc1d13	TBC1 domain family, member 13	ENSMUSG00000039678	18268	48,06
Mif4gd	MIF4G domain containing	ENSMUSG00000020743	5052	48,06
Tstd2	thiosulfate sulfurtransferase (rhodanese)-like domain containing 2	ENSMUSG00000035495	23949	48,06
Xkr6	X Kell blood group precursor related family member 6 homolog	ENSMUSG00000035067	214307	48,06
Zswim5	zinc finger SWIM-type containing 5	ENSMUSG00000033948	111889	48,06
Plekhb2	pleckstrin homology domain containing, family B (evectins) member 2	ENSMUSG00000026123	29605	47,99
Leprot	leptin receptor overlapping transcript	ENSMUSG00000035212	11647	47,99

Sh3bp5	SH3-domain binding protein 5 (BTK-associated)	ENSMUSG00000021892	76199	47,99
Ankrd42	ankyrin repeat domain 42	ENSMUSG00000041343	55420	47,99
Cep290	centrosomal protein 290	ENSMUSG00000019971	85367	47,99
Ubr1	ubiquitin protein ligase E3 component n-recognin 1	ENSMUSG00000027272	110447	47,99
Sde2	SDE2 telomere maintenance homolog (S. pombe)	ENSMUSG00000038806	16947	47,92
Ccdc112	coiled-coil domain containing 112	ENSMUSG00000071855	29778	47,92
Slc27a4	solute carrier family 27 (fatty acid transporter), member 4	ENSMUSG00000059316	14889	47,92
Cbfb	core binding factor beta	ENSMUSG00000031885	47313	47,92
Glt28d2	glycosyltransferase 28 domain containing 2	ENSMUSG00000031286	86472	47,92
Hist1h2an	histone cluster 1, H2an	ENSMUSG00000069309	393	47,84
		ENSMUSG00000053192	9543	47,84
Irak4	interleukin-1 receptor-associated kinase 4	ENSMUSG00000059883	38173	47,84
Prpf31	PRP31 pre-mRNA processing factor 31 homolog (yeast)	ENSMUSG00000008373	12502	47,84
Ralb	v-ral simian leukemia viral oncogene homolog B (ras related)	ENSMUSG00000004451	34490	47,84
Ikbkb	inhibitor of kappaB kinase beta	ENSMUSG00000031537	47378	47,84
		ENSMUSG00000022802	65175	47,84
Cntn1	contactin 1	ENSMUSG00000055022	290803	47,84
Bok	BCL2-related ovarian killer protein	ENSMUSG00000026278	10069	47,77
Copg1	coatomer protein complex, subunit gamma 1	ENSMUSG00000030058	25782	47,77
		ENSMUSG00000038957	43960	47,77
Pcyt1b	phosphate cytidylyltransferase 1, choline, beta isoform	ENSMUSG00000035246	95089	47,77
Eif4a2	eukaryotic translation initiation factor 4A2	ENSMUSG00000022884	6693	47,77
Coq6	coenzyme Q6 homolog (yeast)	ENSMUSG00000021235	12140	47,77
Rab3a	RAB3A, member RAS oncogene family	ENSMUSG00000031840	3999	47,70
Dnal4	dynein, axonemal, light chain 4	ENSMUSG00000022420	16397	47,70
Bbs4	Bardet-Biedl syndrome 4 (human)	ENSMUSG00000025235	31543	47,70
Uqcc2	ubiquinol-cytochrome c reductase complex assembly factor 2	ENSMUSG00000024208	11254	47,70
Trappc6a	trafficking protein particle complex 6A	ENSMUSG00000002043	7476	47,70
Rp9	retinitis pigmentosa 9 (human)	ENSMUSG00000032239	20046	47,70
Cep170b	centrosomal protein 170B	ENSMUSG00000072825	24418	47,70
		ENSMUSG00000034320	18694	47,63
Psmb2	proteasome (prosome, macropain) subunit, beta type 2	ENSMUSG00000028837	32085	47,63
Fbxw4	F-box and WD-40 domain protein 4	ENSMUSG00000040913	82059	47,63
Zfyve9	zinc finger, FYVE domain containing 9	ENSMUSG00000034557	143333	47,63
Ugcg	UDP-glucose ceramide glucosyltransferase	ENSMUSG00000028381	33577	47,55
Carm1	coactivator-associated arginine methyltransferase 1	ENSMUSG00000032185	42594	47,55
Cx3cl1	chemokine (C-X3-C motif) ligand 1	ENSMUSG00000031778	10415	47,55

Dcaf10	DDB1 and CUL4 associated factor 10	ENSMUSG00000035572	37659	47,55
Mapkapk5	MAP kinase-activated protein kinase 5	ENSMUSG00000029454	27293	47,55
Mthfd2	methylenetetrahydrofolate dehydrogenase (NAD+ dependent), methenyltetrahydrofolate cyclohydrolase	ENSMUSG00000005667	11916	47,48
Eif2b4	eukaryotic translation initiation factor 2B, subunit 4 delta	ENSMUSG00000029145	5873	47,48
Celf3	CUGBP, Elav-like family member 3	ENSMUSG00000028137	13363	47,48
Trpc2	transient receptor potential cation channel, subfamily C, member 2	ENSMUSG00000070425	31354	47,48
Actr8	ARP8 actin-related protein 8	ENSMUSG00000015971	14885	47,48
		ENSMUSG00000057572	18853	47,48
BC003331	cDNA sequence BC003331	ENSMUSG00000006010	31776	47,48
Scfd2	Sec1 family domain containing 2	ENSMUSG00000062110	326932	47,48
Spcs3	signal peptidase complex subunit 3 homolog (S. cerevisiae)	ENSMUSG00000054408	9566	47,41
Ncstn	nicastrin	ENSMUSG00000003458	16783	47,41
Elmod2	ELMO/CED-12 domain containing 2	ENSMUSG00000035151	19855	47,41
Gripap1	GRIP1 associated protein 1	ENSMUSG00000031153	30803	47,41
Tmod3	tropomodulin 3	ENSMUSG00000058587	61862	47,41
Srrt	serrate RNA effector molecule homolog (Arabidopsis)	ENSMUSG00000037364	11971	47,41
Depdc5	DEP domain containing 5	ENSMUSG00000037426	130531	47,41
Gramd4	GRAM domain containing 4	ENSMUSG00000035900	79940	47,41
Slc20a1	solute carrier family 20, member 1	ENSMUSG00000027397	12853	47,34
Muc5b	mucin 5, subtype B, tracheobronchial	ENSMUSG00000066108	34014	47,34
Dzip3	DAZ interacting protein 3, zinc finger	ENSMUSG00000064061	69934	47,34
		ENSMUSG00000050530	113583	47,26
Bmp2k	BMP2 inducible kinase	ENSMUSG00000034663	94179	47,26
Zfc3h1	zinc finger, C3H1-type containing	ENSMUSG00000034163	47814	47,26
		ENSMUSG00000022829	270021	47,26
Lmbr1	limb region 1	ENSMUSG00000010721	148577	47,26
Ppil1	peptidylprolyl isomerase (cyclophilin)-like 1	ENSMUSG00000024007	13356	47,19
Atp6v1b2	ATPase, H+ transporting, lysosomal V1 subunit B2	ENSMUSG00000006273	25066	47,19
Fam20a	family with sequence similarity 20, member A	ENSMUSG00000020614	52531	47,19
Mien1	migration and invasion enhancer 1	ENSMUSG00000002580	1284	47,12
Elp3	elongator acetyltransferase complex subunit 3	ENSMUSG00000022031	62667	47,12
Rabgef1	RAB guanine nucleotide exchange factor (GEF) 1	ENSMUSG00000025340	42545	47,12
Nkiras1	NFKB inhibitor interacting Ras-like protein 1	ENSMUSG00000021772	12807	47,12
Psmg4	proteasome (prosome, macropain) assembly chaperone 4	ENSMUSG00000071451	15209	47,12
Araf	v-raf murine sarcoma 3611 viral oncogene homolog	ENSMUSG00000001127	62706	47,12
Gkap1	G kinase anchoring protein 1	ENSMUSG00000021552	40838	47,12

Fgd6	FYVE, RhoGEF and PH domain containing 6	ENSMUSG00000020021	109339	47,12
Stpg2	sperm tail PG rich repeat containing 2	ENSMUSG00000047940	504606	47,05
Psmb4	proteasome (prosome, macropain) subunit, beta type 4	ENSMUSG00000005779	2869	47,05
Ppp6r1	protein phosphatase 6, regulatory subunit 1	ENSMUSG00000052296	27450	47,05
Usp19	ubiquitin specific peptidase 19	ENSMUSG00000006676	11658	47,05
Polr3b	polymerase (RNA) III (DNA directed) polypeptide B	ENSMUSG00000034453	104741	47,05
Mogat1	monoacylglycerol O-acyltransferase 1	ENSMUSG00000012187	27183	47,05
Atf6b	activating transcription factor 6 beta	ENSMUSG00000015461	7929	46,97
Mboat1	membrane bound O-acyltransferase domain containing 1	ENSMUSG00000038732	110220	46,97
Asph	aspartate-beta-hydroxylase	ENSMUSG00000028207	221276	46,97
Uba6	ubiquitin-like modifier activating enzyme 6	ENSMUSG00000035898	62084	46,97
H1fx	H1 histone family, member X	ENSMUSG00000044927	1061	46,90
Ppp1r8	protein phosphatase 1, regulatory (inhibitor) subunit 8	ENSMUSG00000028882	16241	46,90
Ddx27	DEAD (Asp-Glu-Ala-Asp) box polypeptide 27	ENSMUSG00000017999	19755	46,90
Commd3	COMM domain containing 3	ENSMUSG00000051154	3848	46,90
		ENSMUSG00000091306	7342	46,90
Med19	mediator of RNA polymerase II transcription, subunit 19 homolog (yeast)	ENSMUSG00000027080	9814	46,90
Fam169a	family with sequence similarity 169, member A	ENSMUSG00000041817	62235	46,90
Cebpz	CCAAT/enhancer binding protein zeta	ENSMUSG00000024081	18065	46,90
		ENSMUSG00000058446	76585	46,90
Fam129b	family with sequence similarity 129, member B	ENSMUSG00000026796	49141	46,90
Dab2	disabled 2, mitogen-responsive phosphoprotein	ENSMUSG00000022150	140925	46,90
		ENSMUSG00000017639	106812	46,83
0610009L18Rik	RIKEN cDNA 0610009L18 gene	ENSMUSG00000043644	2513	46,83
Dtd1	D-tyrosyl-tRNA deacylase 1	ENSMUSG00000027430	168862	46,83
Dpys	dihydropyrimidinase	ENSMUSG00000022304	88986	46,83
		ENSMUSG00000002250	68716	46,83
Ptdss1	phosphatidylserine synthase 1	ENSMUSG00000021518	65572	46,76
Rnf43	ring finger protein 43	ENSMUSG00000034177	72818	46,76
Tsacc	TSSK6 activating co-chaperone	ENSMUSG00000010538	14243	46,68
Tsc2	tuberous sclerosis 2	ENSMUSG00000002496	36693	46,68
Ndufs1	NADH dehydrogenase (ubiquinone) Fe-S protein 1	ENSMUSG00000025968	33227	46,68
		ENSMUSG00000033488	30481	46,68
Rnf8	ring finger protein 8	ENSMUSG00000090083	88570	46,68
Tmx4	thioredoxin-related transmembrane protein 4	ENSMUSG00000034723	49961	46,68
		ENSMUSG00000094638	29152	46,61

Ptov1	prostate tumor over expressed gene 1	ENSMUSG00000038502	6721	46,61
Mff	mitochondrial fission factor	ENSMUSG00000026150	27505	46,61
		ENSMUSG00000040818	53817	46,61
Ppp2r5a	protein phosphatase 2, regulatory subunit B (B56), alpha isoform	ENSMUSG00000026626	45061	46,61
Htt	huntingtin	ENSMUSG00000029104	150795	46,61
5031439G07Rik	RIKEN cDNA 5031439G07 gene	ENSMUSG00000036046	44616	46,54
		ENSMUSG00000028719	27906	46,54
		ENSMUSG00000061988	645	46,54
Tubgcp3	tubulin, gamma complex associated protein 3	ENSMUSG00000000759	57972	46,54
Rbm4	RNA binding motif protein 4	ENSMUSG00000094936	9609	46,54
Heatr5a	HEAT repeat containing 5A	ENSMUSG00000035181	95449	46,54
Sin3a	transcriptional regulator, SIN3A (yeast)	ENSMUSG00000042557	56327	46,54
Pdzrn4	PDZ domain containing RING finger 4	ENSMUSG00000036218	374905	46,54
Casp6	caspase 6	ENSMUSG00000027997	12679	46,47
Lztfl1	leucine zipper transcription factor-like 1	ENSMUSG00000025245	23210	46,47
Tifa	TRAF-interacting protein with forkhead-associated domain	ENSMUSG00000046688	42264	46,47
		ENSMUSG00000029229	43749	46,47
Uba5	ubiquitin-like modifier activating enzyme 5	ENSMUSG00000032557	16536	46,47
Xrn2	5'-3' exoribonuclease 2	ENSMUSG00000027433	65005	46,47
Mgrn1	mahogunin, ring finger 1	ENSMUSG00000022517	52197	46,47
Ist1	increased sodium tolerance 1 homolog (yeast)	ENSMUSG00000031729	21973	46,47
Msh3	mutS homolog 3 (E. coli)	ENSMUSG00000014850	143123	46,47
Ehf	ets homologous factor	ENSMUSG00000012350	39846	46,47
Kremen1	kringle containing transmembrane protein 1	ENSMUSG00000020393	70007	46,47
2700029M09Rik	RIKEN cDNA 2700029M09 gene	ENSMUSG00000038005	17163	46,39
Aagab	alpha- and gamma-adaptin binding protein	ENSMUSG00000037257	39235	46,39
Mki67ip	Mki67 (FHA domain) interacting nucleolar phosphoprotein	ENSMUSG00000026377	11984	46,39
Psmg2	proteasome (prosome, macropain) assembly chaperone 2	ENSMUSG00000024537	12564	46,39
Pja1	praja1, RING-H2 motif containing	ENSMUSG00000034403	5540	46,39
Gadd45b	growth arrest and DNA-damage-inducible 45 beta	ENSMUSG00000015312	2114	46,39
Naa38	N(alpha)-acetyltransferase 38, NatC auxiliary subunit	ENSMUSG00000044155	7173	46,32
Rtdr1	rhabdoid tumor deletion region gene 1	ENSMUSG00000009070	75110	46,32
		ENSMUSG00000078584	29143	46,32
Asap1	ArfGAP with SH3 domain, ankyrin repeat and PH domain1	ENSMUSG00000022377	296063	46,32
		ENSMUSG00000069094	88215	46,32
Tdrd3	tudor domain containing 3	ENSMUSG00000022019	128735	46,32
Ap1m2	adaptor protein complex AP-1, mu 2 subunit	ENSMUSG00000003309	16877	46,25
Trmt2a	TRM2 tRNA methyltransferase 2A	ENSMUSG00000022721	6094	46,25

Ccdc55	coiled-coil domain containing 55	ENSMUSG00000037958	34144	46,25
Lamc2	laminin, gamma 2	ENSMUSG00000026479	63692	46,25
Cpeb2	cytoplasmic polyadenylation element binding protein 2	ENSMUSG00000039782	56262	46,25
		ENSMUSG00000034799	47341	46,18
Prep	prolyl endopeptidase	ENSMUSG00000019849	91792	46,18
Asic2	acid-sensing (proton-gated) ion channel 2	ENSMUSG00000020704	1088289	46,18
Wwp1	WW domain containing E3 ubiquitin protein ligase 1	ENSMUSG00000041058	100691	46,18
Milr1	mast cell immunoglobulin like receptor 1	ENSMUSG00000040528	17569	46,18
Zfand2a	zinc finger, AN1-type domain 2A	ENSMUSG00000053581	13339	46,10
Spa17	sperm autoantigenic protein 17	ENSMUSG00000001948	10426	46,10
Abhd17c	abhydrolase domain containing 17C	ENSMUSG00000038459	42538	46,10
Ipmk	inositol polyphosphate multikinase	ENSMUSG00000060733	85565	46,10
Smc2	structural maintenance of chromosomes 2	ENSMUSG00000028312	49018	46,10
Tmem260	transmembrane protein 260	ENSMUSG00000036339	69008	46,10
Psd	pleckstrin and Sec7 domain containing	ENSMUSG00000037126	15070	46,10
Calu	calumenin	ENSMUSG00000029767	29042	46,03
Ppil2	peptidylprolyl isomerase (cyclophilin)-like 2	ENSMUSG00000022771	24703	46,03
H2afx	H2A histone family, member X	ENSMUSG00000049932	1357	45,96
Snrpd1	small nuclear ribonucleoprotein D1	ENSMUSG00000002477	10435	45,96
Ehmt2	euchromatic histone lysine N-methyltransferase 2	ENSMUSG00000013787	15584	45,96
Sft2d1	SFT2 domain containing 1	ENSMUSG00000073468	85751	45,96
Arrdc1	arrestin domain containing 1	ENSMUSG00000026972	9901	45,96
		ENSMUSG00000061390	381	45,89
Gng8	guanine nucleotide binding protein (G protein), gamma 8	ENSMUSG00000063594	3650	45,89
Naa60	N(alpha)-acetyltransferase 60, NatF catalytic subunit	ENSMUSG00000005982	32396	45,89
Tm2d3	TM2 domain containing 3	ENSMUSG00000078681	10745	45,89
Cdc123	cell division cycle 123	ENSMUSG00000039128	50871	45,89
Terf2	telomeric repeat binding factor 2	ENSMUSG00000031921	27148	45,89
Ing4	inhibitor of growth family, member 4	ENSMUSG00000030330	11740	45,89
Supt16	suppressor of Ty 16	ENSMUSG00000035726	36821	45,89
Efr3a	EFR3 homolog A (S. cerevisiae)	ENSMUSG00000015002	86783	45,89
Mettl5	methyltransferase like 5	ENSMUSG00000051730	14418	45,81
Ctdsp1	CTD (carboxy-terminal domain, RNA polymerase II, polypeptide A) small phosphatase 1	ENSMUSG00000026176	5777	45,81
Iqsec2	IQ motif and Sec7 domain 2	ENSMUSG00000041115	80969	45,81
Tmem216	transmembrane protein 216	ENSMUSG00000024667	22374	45,81
Pwwp2a	PWWP domain containing 2A	ENSMUSG00000044950	39494	45,81
Ttbk2	tau tubulin kinase 2	ENSMUSG00000090100	117789	45,81
Vkorc1l1	vitamin K epoxide reductase complex, subunit 1-like 1	ENSMUSG00000066735	42637	45,81
Xrcc1	X-ray repair complementing defective repair in Chinese hamster cells 1	ENSMUSG00000051768	26289	45,74

Amz1	archaelysin family metallopeptidase 1	ENSMUSG00000050022	37313	45,74
Scap	SREBF chaperone	ENSMUSG00000032485	51659	45,74
Uhrf1bp1	UHRF1 (ICBP90) binding protein 1	ENSMUSG00000039512	43551	45,74
Thap3	THAP domain containing, apoptosis associated protein 3	ENSMUSG00000039759	6359	45,74
Cdr2l	cerebellar degeneration-related protein 2-like	ENSMUSG00000050910	14217	45,74
Pomgnt1	protein O-linked mannose beta 1,2-N-acetylglucosaminyltransferase	ENSMUSG00000028700	36010	45,74
Acvr1	activin A receptor, type 1	ENSMUSG00000026836	178514	45,74
Ext2	exostoses (multiple) 2	ENSMUSG00000027198	126938	45,74
Dock3	dedicator of cyto-kinesis 3	ENSMUSG00000039716	339076	45,74
Adprh	ADP-ribosylarginine hydrolase	ENSMUSG00000002844	7291	45,67
Ankrd40	ankyrin repeat domain 40	ENSMUSG00000020864	13841	45,67
Cgrrf1	cell growth regulator with ring finger domain 1	ENSMUSG00000055128	22060	45,67
Stk4	serine/threonine kinase 4	ENSMUSG00000018209	85203	45,67
Sall2	sal-like 2 (Drosophila)	ENSMUSG00000049532	17591	45,67
Eepd1	endonuclease/exonuclease/phosphatase family domain containing 1	ENSMUSG00000036611	122564	45,67
Ppcs	phosphopantothenoylcysteine synthetase	ENSMUSG00000028636	3891	45,60
Gfpt1	glutamine fructose-6-phosphate transaminase 1	ENSMUSG00000029992	49352	45,60
Cyb5r4	cytochrome b5 reductase 4	ENSMUSG00000032872	55761	45,60
Tma16	translation machinery associated 16 homolog (S. cerevisiae)	ENSMUSG00000025591	12201	45,60
Armc9	armadillo repeat containing 9	ENSMUSG00000062590	123505	45,60
		ENSMUSG00000039431	83703	45,60
Zbtb7c	zinc finger and BTB domain containing 7C	ENSMUSG00000044646	328384	45,60
Slc25a26	solute carrier family 25 (mitochondrial carrier, phosphate carrier), member 26	ENSMUSG00000045100	104335	45,60
Anxa6	annexin A6	ENSMUSG00000018340	54338	45,52
4933426M11Rik	RIKEN cDNA 4933426M11 gene	ENSMUSG00000021133	90301	45,52
Dnajc8	DnaJ (Hsp40) homolog, subfamily C, member 8	ENSMUSG00000054405	18193	45,45
Lig1	ligase I, DNA, ATP-dependent	ENSMUSG00000056394	34151	45,45
		ENSMUSG00000018395	34589	45,45
Hmg20b	high mobility group 20B	ENSMUSG00000020232	4433	45,45
Cpsf7	cleavage and polyadenylation specific factor 7	ENSMUSG00000034820	22492	45,45
Nfasc	neurofascin	ENSMUSG00000026442	177107	45,45
Slc14a1	solute carrier family 14 (urea transporter), member 1	ENSMUSG00000059336	42029	45,45
Wbp1	WW domain binding protein 1	ENSMUSG00000030035	2516	45,38
Aco1	aconitase 1	ENSMUSG00000028405	55258	45,38
Ncapd2	non-SMC condensin I complex, subunit D2	ENSMUSG00000038252	23587	45,38
Tln1	talin 1	ENSMUSG00000028465	31173	45,38
Cep68	centrosomal protein 68	ENSMUSG00000044066	22393	45,38
Fam155a	family with sequence similarity 155, member A	ENSMUSG00000079157	565018	45,38

Cdkl2	cyclin-dependent kinase-like 2 (CDC2-related kinase)	ENSMUSG00000029403	36969	45,31
Zfp512	zinc finger protein 512	ENSMUSG00000062761	29314	45,31
Myo1b	myosin IB	ENSMUSG00000018417	166307	45,31
Gda	guanine deaminase	ENSMUSG00000058624	82139	45,31
Arhgap23	Rho GTPase activating protein 23	ENSMUSG00000049807	86870	45,31
		ENSMUSG00000025898	75152	45,31
Focad	focadhesin	ENSMUSG00000038368	316383	45,31
Fmo3	flavin containing monooxygenase 3	ENSMUSG00000026691	30727	45,31
Cenpp	centromere protein P	ENSMUSG00000021391	188720	45,23
1810030O07Rik	RIKEN cDNA 1810030O07 gene	ENSMUSG00000044148	18790	45,23
Ccnk	cyclin K	ENSMUSG00000021258	23622	45,23
Lrif1	ligand dependent nuclear receptor interacting factor 1	ENSMUSG00000056260	51591	45,23
Xylt1	xylosyltransferase 1	ENSMUSG00000030657	286773	45,23
Nme2	NME/NM23 nucleoside diphosphate kinase 2	ENSMUSG00000020857	6446	45,16
Il10	interleukin 10	ENSMUSG00000016529	5130	45,16
		ENSMUSG00000019810	19496	45,16
		ENSMUSG00000093456	48922	45,16
Slc22a17	solute carrier family 22 (organic cation transporter), member 17	ENSMUSG00000022199	6406	45,09
Bola1	bolA-like 1 (E. coli)	ENSMUSG00000015943	23122	45,09
Ankrd49	ankyrin repeat domain 49	ENSMUSG00000031931	2756	45,09
Ppl	periplakin	ENSMUSG00000039457	46191	45,09
Vat1l	vesicle amine transport protein 1 homolog-like (T. californica)	ENSMUSG00000046844	168460	45,09
Pptc7	PTC7 protein phosphatase homolog (S. cerevisiae)	ENSMUSG00000038582	39917	45,09
		ENSMUSG00000039568	5538	45,09
Avpi1	arginine vasopressin-induced 1	ENSMUSG00000018821	5787	45,09
Gba	glucosidase, beta, acid	ENSMUSG00000028048	5754	45,09
Zbtb5	zinc finger and BTB domain containing 5	ENSMUSG00000049657	21171	45,02
Mboat2	membrane bound O-acyltransferase domain containing 2	ENSMUSG00000020646	128703	45,02
Nlgn1	neuroligin 1	ENSMUSG00000063887	701924	45,02
Zmpste24	zinc metallopeptidase, STE24	ENSMUSG00000043207	39005	45,02
Fam76a	family with sequence similarity 76, member A	ENSMUSG00000028878	23346	45,02
		ENSMUSG00000062190	36838	45,02
Dsc3	desmocollin 3	ENSMUSG00000059898	41168	45,02
Rraga	Ras-related GTP binding A	ENSMUSG00000070934	1609	44,94
Elp2	elongator acetyltransferase complex subunit 2	ENSMUSG00000024271	34870	44,94
Pigf	phosphatidylinositol glycan anchor biosynthesis, class F	ENSMUSG00000024145	28151	44,94
Psmd9	proteasome (prosome, macropain) 26S subunit, non-ATPase, 9	ENSMUSG00000029440	21942	44,94
Far2	fatty acyl CoA reductase 2	ENSMUSG00000030303	135500	44,94
Sgpp2	sphingosine-1-phosphate phosphotase 2	ENSMUSG00000032908	109941	44,94
		ENSMUSG00000024030	58080	44,94
Btbd3	BTB (POZ) domain containing 3	ENSMUSG00000062098	332728	44,94

Zbtb2	zinc finger and BTB domain containing 2	ENSMUSG00000075327	21035	44,94
Fam183b	family with sequence similarity 183, member B	ENSMUSG00000049154	9164	44,94
Nptx2	neuronal pentraxin 2	ENSMUSG00000059991	11592	44,87
Erlin2	ER lipid raft associated 2	ENSMUSG00000031483	15635	44,87
Eif4ebp2	eukaryotic translation initiation factor 4E binding protein 2	ENSMUSG00000020091	20173	44,87
Zfp62	zinc finger protein 62	ENSMUSG00000046311	15525	44,87
Nop58	NOP58 ribonucleoprotein	ENSMUSG00000026020	26505	44,87
Lpcat2	lysophosphatidylcholine acyltransferase 2	ENSMUSG00000033192	63930	44,87
Leng8	leukocyte receptor cluster (LRC) member 8	ENSMUSG00000035545	11135	44,87
Dnajb1	DnaJ (Hsp40) homolog, subfamily B, member 1	ENSMUSG00000005483	3728	44,87
Lrrfip2	leucine rich repeat (in FLII) interacting protein 2	ENSMUSG00000032497	107558	44,87
Dcbld2	discoidin, CUB and LCCL domain containing 2	ENSMUSG00000035107	61285	44,87
Prelid1	PRELI domain containing 1	ENSMUSG00000021486	3217	44,80
Spcs2	signal peptidase complex subunit 2 homolog (S. cerevisiae)	ENSMUSG00000035227	21315	44,80
		ENSMUSG00000074141	24440	44,80
Zfand1	zinc finger, AN1-type domain 1	ENSMUSG00000039795	11365	44,80
Frs2	fibroblast growth factor receptor substrate 2	ENSMUSG00000020170	78346	44,80
Cdkl5	cyclin-dependent kinase-like 5	ENSMUSG00000031292	210395	44,80
Znhit1	zinc finger, HIT domain containing 1	ENSMUSG00000059518	5858	44,73
		ENSMUSG00000055943	17166	44,73
Polr2b	polymerase (RNA) II (DNA directed) polypeptide B	ENSMUSG00000029250	39178	44,73
Lta4h	leukotriene A4 hydrolase	ENSMUSG00000015889	31501	44,73
		ENSMUSG00000029613	86469	44,73
Trappc13	trafficking protein particle complex 13	ENSMUSG00000021711	36321	44,73
		ENSMUSG00000078134	1311	44,73
		ENSMUSG00000090935	34971	44,73
Arnt	aryl hydrocarbon receptor nuclear translocator	ENSMUSG00000015522	62853	44,73
Gtpbp4	GTP binding protein 4	ENSMUSG00000021149	23947	44,65
		ENSMUSG00000059912	468	44,65
Fam98a	family with sequence similarity 98, member A	ENSMUSG00000002017	14861	44,65
Dedd	death effector domain-containing	ENSMUSG00000013973	13187	44,65
Mars	methionine-tRNA synthetase	ENSMUSG00000040354	15566	44,65
Ralgps2	Ral GEF with PH domain and SH3 binding motif 2	ENSMUSG00000026594	135461	44,65
Pkd1l3	polycystic kidney disease 1 like 3	ENSMUSG00000048827	58076	44,65
Ercc3	excision repair cross-complementing rodent repair deficiency, complementation group 3	ENSMUSG00000024382	29852	44,58
Slc26a1	solute carrier family 26 (sulfate transporter), member 1	ENSMUSG00000046959	5692	44,58

		ENSMUSG00000020715	93203	44,58
6330416G13Rik	RIKEN cDNA 6330416G13 gene	ENSMUSG00000045917	27577	44,51
Rbmxl1	RNA binding motif protein, X linked-like-1	ENSMUSG00000037070	3658	44,51
Mrps35	mitochondrial ribosomal protein S35	ENSMUSG00000040112	28140	44,51
Nars	asparaginyl-tRNA synthetase	ENSMUSG00000024587	16893	44,51
Ppp3r1	protein phosphatase 3, regulatory subunit B, alpha isoform (calcineurin B, type I)	ENSMUSG00000033953	41113	44,51
Pdcd4	programmed cell death 4	ENSMUSG00000024975	37631	44,51
Tmem159	transmembrane protein 159	ENSMUSG00000030917	18617	44,51
Atp13a3	ATPase type 13A3	ENSMUSG00000022533	76415	44,51
Capn15	calpain 15	ENSMUSG00000037326	26125	44,51
Syp	synaptophysin	ENSMUSG00000031144	14786	44,44
Slc24a5	solute carrier family 24, member 5	ENSMUSG00000035183	20554	44,44
Gnl2	guanine nucleotide binding protein-like 2 (nucleolar)	ENSMUSG00000028869	38796	44,44
Slc5a10	solute carrier family 5 (sodium/glucose cotransporter), member 10	ENSMUSG00000042371	48046	44,44
Rmdn1	regulator of microtubule dynamics 1	ENSMUSG00000028229	31771	44,44
Eml2	echinoderm microtubule associated protein like 2	ENSMUSG00000040811	30062	44,36
Ppp1r16a	protein phosphatase 1, regulatory (inhibitor) subunit 16A	ENSMUSG00000033819	23305	44,36
Lsm6	LSM6 homolog, U6 small nuclear RNA associated (S. cerevisiae)	ENSMUSG00000031683	16276	44,36
Tcp11l1	t-complex 11 like 1	ENSMUSG00000027175	54882	44,36
		ENSMUSG00000031360	131271	44,36
5830418K08Rik	RIKEN cDNA 5830418K08 gene	ENSMUSG00000046111	40874	44,36
Synm	synemin, intermediate filament protein	ENSMUSG00000030554	29582	44,36
Lingo1	leucine rich repeat and Ig domain containing 1	ENSMUSG00000049556	66779	44,29
Dctn1	dynactin 1	ENSMUSG00000031865	34198	44,29
Tsc22d4	TSC22 domain family, member 4	ENSMUSG00000029723	22721	44,29
Golga7	golgi autoantigen, golgin subfamily a, 7	ENSMUSG00000015341	15722	44,29
Srp68	signal recognition particle 68	ENSMUSG00000020780	29052	44,29
Impa1	inositol (myo)-1(or 4)-monophosphatase 1	ENSMUSG00000027531	17900	44,29
Notch1	notch 1	ENSMUSG00000026923	58761	44,29
Nploc4	nuclear protein localization 4 homolog (S. cerevisiae)	ENSMUSG00000039703	57339	44,29
Epb4.1l1	erythrocyte protein band 4.1-like 1	ENSMUSG00000027624	122306	44,22
Fam210b	family with sequence similarity 210, member B	ENSMUSG00000027495	10185	44,22
Abhd14b	abhydrolase domain containing 14b	ENSMUSG00000042073	4276	44,22
Ccz1	CCZ1 vacuolar protein trafficking and biogenesis associated	ENSMUSG00000029617	26938	44,22
Spag17	sperm associated antigen 17	ENSMUSG00000027867	257917	44,22
		ENSMUSG00000029363	10945	44,15
Bad	BCL2-associated agonist of cell death	ENSMUSG00000024959	10038	44,07

Ankrd50	ankyrin repeat domain 50	ENSMUSG00000044864	35584	44,07
Acly	ATP citrate lyase	ENSMUSG00000020917	51648	44,07
Gpr56	G protein-coupled receptor 56	ENSMUSG00000031785	37100	44,07
Api5	apoptosis inhibitor 5	ENSMUSG00000027193	26455	44,07
Pls3	plastin 3 (T-isoform)	ENSMUSG00000016382	89529	44,07
Kctd3	potassium channel tetramerisation domain containing 3	ENSMUSG00000026608	36743	44,07
		ENSMUSG00000041671	17604	44,07
		ENSMUSG00000078153	837	44,00
Skiv2l2	superkiller viralicidic activity 2-like 2 (S. cerevisiae)	ENSMUSG00000016018	59601	44,00
Serinc1	serine incorporator 1	ENSMUSG00000019877	16757	44,00
Spire1	spire homolog 1 (Drosophila)	ENSMUSG00000024533	64569	44,00
Il17ra	interleukin 17 receptor A	ENSMUSG00000002897	20531	44,00
Tbck	TBC1 domain containing kinase	ENSMUSG00000028030	154363	44,00
Brox	BRO1 domain and CAAX motif containing	ENSMUSG00000046836	20915	43,93
Ilkap	integrin-linked kinase-associated serine/threonine phosphatase 2C	ENSMUSG00000026309	22953	43,93
Pithd1	PITH (C-terminal proteasome-interacting domain of thioredoxin-like) domain containing 1	ENSMUSG00000028669	11805	43,93
BC017643	cDNA sequence BC017643	ENSMUSG00000039294	6735	43,93
Twistnb	TWIST neighbor	ENSMUSG00000020561	9757	43,93
Efcab6	EF-hand calcium binding domain 6	ENSMUSG00000022441	198668	43,93
Tbc1d14	TBC1 domain family, member 14	ENSMUSG00000029192	102673	43,93
Casp3	caspase 3	ENSMUSG00000031628	21258	43,93
Mdm2	transformed mouse 3T3 cell double minute 2	ENSMUSG00000020184	21871	43,86
Ccnh	cyclin H	ENSMUSG00000021548	34062	43,86
Ptplb	protein tyrosine phosphatase-like (proline instead of catalytic arginine), member b	ENSMUSG00000035376	86755	43,86
Celf4	CUGBP, Elav-like family member 4	ENSMUSG00000024268	276352	43,78
		ENSMUSG00000089847	15693	43,78
		ENSMUSG00000092345	38679	43,78
Gnptg	N-acetylglucosamine-1-phosphotransferase, gamma subunit	ENSMUSG00000035521	6794	43,78
Psmc2	proteasome (prosome, macropain) 26S subunit, ATPase 2	ENSMUSG00000028932	18505	43,78
AU019823	expressed sequence AU019823	ENSMUSG00000059820	12225	43,78
Ola1	Obg-like ATPase 1	ENSMUSG00000027108	126124	43,78
Acer3	alkaline ceramidase 3	ENSMUSG00000030760	107551	43,78
Abhd16a	abhydrolase domain containing 16A	ENSMUSG00000007036	13725	43,71
Eif3d	eukaryotic translation initiation factor 3, subunit D	ENSMUSG00000016554	11827	43,71
Trappc4	trafficking protein particle complex 4	ENSMUSG00000032112	3788	43,71
Celsr3	cadherin, EGF LAG seven-pass G-type receptor 3 (flamingo homolog, Drosophila)	ENSMUSG00000023473	26644	43,71
Krt19	keratin 19	ENSMUSG00000020911	7856	43,71
Megf11	multiple EGF-like-domains 11	ENSMUSG00000036466	323580	43,71

Adam17	a disintegrin and metallopeptidase domain 17	ENSMUSG00000052593	50124	43,71
Nfil3	nuclear factor, interleukin 3, regulated	ENSMUSG00000056749	13865	43,71
Gm10094	predicted gene 10094	ENSMUSG00000061104	780	43,64
		ENSMUSG00000037747	98284	43,64
		ENSMUSG00000036206	84654	43,64
		ENSMUSG00000056919	65964	43,64
Mroh1	maestro heat-like repeat family member 1	ENSMUSG00000022558	72778	43,64
Adam8	a disintegrin and metallopeptidase domain 8	ENSMUSG00000025473	13631	43,64
Chga	chromogranin A	ENSMUSG00000021194	10059	43,57
Vps26b	vacuolar protein sorting 26 homolog B (yeast)	ENSMUSG00000031988	25592	43,57
Cttnbp2nl	CTTNBP2 N-terminal like	ENSMUSG00000062127	51232	43,57
Tfdp2	transcription factor Dp 2	ENSMUSG00000032411	127372	43,57
Zcchc9	zinc finger, CCHC domain containing 9	ENSMUSG00000021621	11173	43,57
		ENSMUSG00000027823	46474	43,49
Pole4	polymerase (DNA-directed), epsilon 4 (p12 subunit)	ENSMUSG00000030042	86374	43,49
Pcx	pyruvate carboxylase	ENSMUSG00000024892	111281	43,49
Ano1	anoctamin 1, calcium activated chloride channel	ENSMUSG00000031075	163426	43,49
Diap3	diaphanous homolog 3 (Drosophila)	ENSMUSG00000022021	485858	43,49
Lsmem1	leucine-rich single-pass membrane protein 1	ENSMUSG00000071342	22930	43,49
Coro2a	coronin, actin binding protein 2A	ENSMUSG00000028337	65266	43,42
Dos	downstream of Stk11	ENSMUSG00000035640	10385	43,42
Rufy1	RUN and FYVE domain containing 1	ENSMUSG00000020375	41840	43,42
Fat1	FAT tumor suppressor homolog 1 (Drosophila)	ENSMUSG00000070047	102050	43,42
Mesdc2	mesoderm development candidate 2	ENSMUSG00000038503	17067	43,35
Ifitm3	interferon induced transmembrane protein 3	ENSMUSG00000025492	1185	43,35
Snrnp200	small nuclear ribonucleoprotein 200 (U5)	ENSMUSG00000003660	32066	43,35
Ccdc12	coiled-coil domain containing 12	ENSMUSG00000019659	55088	43,35
Dcaf7	DDB1 and CUL4 associated factor 7	ENSMUSG00000049354	22453	43,35
Grb14	growth factor receptor bound protein 14	ENSMUSG00000026888	112512	43,35
Ahctf1	AT hook containing transcription factor 1	ENSMUSG00000026491	58787	43,35
Ube4a	ubiquitination factor E4A, UFD2 homolog (S. cerevisiae)	ENSMUSG00000059890	42470	43,35
Ldoc1l	leucine zipper, down-regulated in cancer 1-like	ENSMUSG00000055745	4426	43,28
Higd2a	HIG1 domain family, member 2A	ENSMUSG00000025868	952	43,28
		ENSMUSG00000031467	45085	43,28
Pank2	pantothenate kinase 2	ENSMUSG00000037514	36694	43,28
4931406P16Rik	RIKEN cDNA 4931406P16 gene	ENSMUSG00000066571	76845	43,28
Sash1	SAM and SH3 domain containing 1	ENSMUSG00000015305	163852	43,28

Usp16	ubiquitin specific peptidase 16	ENSMUSG00000025616	28815	43,28
Mrpl28	mitochondrial ribosomal protein L28	ENSMUSG00000024181	3094	43,20
		ENSMUSG00000090425	2416	43,20
		ENSMUSG00000058291	15146	43,20
Proser1	proline and serine rich 1	ENSMUSG00000049504	18090	43,20
Cabyr	calcium-binding tyrosine-(Y)-phosphorylation regulated (fibrousheathin 2)	ENSMUSG00000024430	13823	43,20
Cxxc5	CXXC finger 5	ENSMUSG00000046668	31870	43,13
Brix1	BRX1, biogenesis of ribosomes, homolog (S. cerevisiae)	ENSMUSG00000022247	11169	43,13
Snx10	sorting nexin 10	ENSMUSG00000038301	66779	43,13
Wdr43	WD repeat domain 43	ENSMUSG00000041057	42817	43,13
Slc38a9	solute carrier family 38, member 9	ENSMUSG00000047789	77978	43,13
Slc39a1	solute carrier family 39 (zinc transporter), member 1	ENSMUSG00000052310	5441	43,06
Sirt5	sirtuin 5	ENSMUSG00000054021	24493	43,06
Slc23a1	solute carrier family 23 (nucleobase transporters), member 1	ENSMUSG00000024354	12641	43,06
Phospho2	phosphatase, orphan 2	ENSMUSG00000027088	10383	43,06
Prkag1	protein kinase, AMP-activated, gamma 1 non-catalytic subunit	ENSMUSG00000067713	18711	43,06
Ncoa4	nuclear receptor coactivator 4	ENSMUSG00000056234	19991	43,06
Dlat	dihydrolipoamide S-acetyltransferase (E2 component of pyruvate dehydrogenase complex)	ENSMUSG00000000168	25148	43,06
Aldh3b1	aldehyde dehydrogenase 3 family, member B1	ENSMUSG00000024885	16269	43,06
Cadm2	cell adhesion molecule 2	ENSMUSG00000064115	965488	43,06
Gm21451	predicted gene, 21451	ENSMUSG00000094526	89434	43,06
Yeats4	YEATS domain containing 4	ENSMUSG00000020171	9366	42,99
Sidt2	SID1 transmembrane family, member 2	ENSMUSG00000034908	17391	42,99
Nf2	neurofibromatosis 2	ENSMUSG00000009073	83692	42,99
Adk	adenosine kinase	ENSMUSG00000039197	395995	42,99
Bnip3l	BCL2/adenovirus E1B interacting protein 3-like	ENSMUSG00000022051	23639	42,99
		ENSMUSG00000025231	91909	42,99
Fastk	Fas-activated serine/threonine kinase	ENSMUSG00000028959	6848	42,91
Rgs7bp	regulator of G-protein signalling 7 binding protein	ENSMUSG00000021719	107778	42,91
Cd55	CD55 antigen	ENSMUSG00000026399	23718	42,91
Bbs7	Bardet-Biedl syndrome 7 (human)	ENSMUSG00000037325	40336	42,84
Abcc10	ATP-binding cassette, sub-family C (CFTR/MRP), member 10	ENSMUSG00000032842	25132	42,84
Rbm15b	RNA binding motif protein 15B	ENSMUSG00000045365	3016	42,77
Llgl1	lethal giant larvae homolog 1 (Drosophila)	ENSMUSG00000020536	14464	42,77
Ssu72	Ssu72 RNA polymerase II CTD phosphatase homolog (yeast)	ENSMUSG00000029038	29080	42,77
Cwc25	CWC25 spliceosome-associated protein homolog (S. cerevisiae)	ENSMUSG00000018541	21076	42,77

Smpdl3a	sphingomyelin phosphodiesterase, acid-like 3A	ENSMUSG00000019872	17496	42,77
Dnah6	dynein, axonemal, heavy chain 6	ENSMUSG00000052861	204046	42,77
Cyb5r1	cytochrome b5 reductase 1	ENSMUSG00000026456	5960	42,70
Rabac1	Rab acceptor 1 (prenylated)	ENSMUSG00000003380	2979	42,70
		ENSMUSG00000015944	44633	42,70
Brpf1	bromodomain and PHD finger containing, 1	ENSMUSG00000001632	17677	42,70
Afg3l1	AFG3(ATPase family gene 3)-like 1 (yeast)	ENSMUSG00000031967	26014	42,70
Gm3448	predicted gene 3448	ENSMUSG00000079710	45755	42,70
Tnfrsf1a	tumor necrosis factor receptor superfamily, member 1a	ENSMUSG00000030341	13123	42,70
Hspa14	heat shock protein 14	ENSMUSG00000051396	23965	42,70
BC018507	cDNA sequence BC018507	ENSMUSG00000034525	48946	42,70
Tigd2	tigger transposable element derived 2	ENSMUSG00000049232	3164	42,70
Pfkp	phosphofructokinase, platelet	ENSMUSG00000021196	69010	42,70
Cep152	centrosomal protein 152	ENSMUSG00000068394	62026	42,70
Paxbp1	PAX3 and PAX7 binding protein 1	ENSMUSG00000022974	30507	42,70
Mrpl42	mitochondrial ribosomal protein L42	ENSMUSG00000062981	21122	42,62
Gatad1	GATA zinc finger domain containing 1	ENSMUSG00000007415	15003	42,62
		ENSMUSG00000037395	39355	42,62
Ift80	intraflagellar transport 80	ENSMUSG00000027778	112072	42,62
Erich1	glutamate rich 1	ENSMUSG00000051978	62763	42,62
Ripk4	receptor-interacting serine-threonine kinase 4	ENSMUSG00000005251	21805	42,62
Nup85	nucleoporin 85	ENSMUSG00000020739	19552	42,55
Gng2	guanine nucleotide binding protein (G protein), gamma 2	ENSMUSG00000043004	105069	42,55
Sema4d	sema domain, immunoglobulin domain (Ig), transmembrane domain (TM) and short cytoplasmic domain, (semaphorin) 4D	ENSMUSG00000021451	92502	42,55
Zfhx2	zinc finger homeobox 2	ENSMUSG00000040721	32063	42,55
Cep164	centrosomal protein 164	ENSMUSG00000043987	61746	42,55
Rangrf	RAN guanine nucleotide release factor	ENSMUSG00000032892	2702	42,55
Anks4b	ankyrin repeat and sterile alpha motif domain containing 4B	ENSMUSG00000030909	9859	42,55
		ENSMUSG00000031644	138152	42,55
		ENSMUSG00000036667	42071	42,48
Ddb2	damage specific DNA binding protein 2	ENSMUSG00000002109	25411	42,48
Spty2d1	SPT2, Suppressor of Ty, domain containing 1 (S. cerevisiae)	ENSMUSG00000049516	18016	42,48
Mapre3	microtubule-associated protein, RP/EB family, member 3	ENSMUSG00000029166	51466	42,48
Atp11b	ATPase, class VI, type 11B	ENSMUSG00000037400	102141	42,48
Galnt13	UDP-N-acetyl-alpha-D-galactosamine:polypeptide N-acetylgalactosaminyltransferase 13	ENSMUSG00000060988	681993	42,48
Cnksr3	Cnksr family member 3	ENSMUSG00000015202	93175	42,48
Rbm8a	RNA binding motif protein 8a	ENSMUSG00000038374	3852	42,41

Mkrn1	makorin, ring finger protein, 1	ENSMUSG00000029922	22659	42,41
Prkrir	protein-kinase, interferon-inducible double stranded RNA dependent inhibitor, repressor of (P58 repressor)	ENSMUSG00000030753	14960	42,41
Fzd2	frizzled homolog 2 (Drosophila)	ENSMUSG00000050288	3628	42,41
Dmrt3	doublesex and mab-3 related transcription factor 3	ENSMUSG00000042372	13384	42,41
Golph3	golgi phosphoprotein 3	ENSMUSG00000022200	29770	42,41
Itsn1	intersectin 1 (SH3 domain protein 1A)	ENSMUSG00000022957	191317	42,41
Sp8	trans-acting transcription factor 8	ENSMUSG00000048562	6250	42,41
Bach1	BTB and CNC homology 1	ENSMUSG00000025612	34402	42,41
Yap1	yes-associated protein 1	ENSMUSG00000053110	72598	42,41
5031425E22Rik	RIKEN cDNA 5031425E22 gene	ENSMUSG00000073147	2110	42,33
Grina	glutamate receptor, ionotropic, N-methyl D-aspartate-associated protein 1 (glutamate binding)	ENSMUSG00000022564	3098	42,33
Gpr108	G protein-coupled receptor 108	ENSMUSG00000005823	12728	42,33
Cep110	centrosomal protein 110	ENSMUSG00000057110	69331	42,33
Pisd	phosphatidylserine decarboxylase	ENSMUSG00000023452	49346	42,26
Evc	Ellis van Creveld gene syndrome	ENSMUSG00000029122	47797	42,26
		ENSMUSG00000091957	948	42,26
Rnf215	ring finger protein 215	ENSMUSG00000003581	5971	42,26
Fam86	family with sequence similarity 86	ENSMUSG00000022544	11832	42,26
Lrrc20	leucine rich repeat containing 20	ENSMUSG00000037151	106394	42,26
Iqsec1	IQ motif and Sec7 domain 1	ENSMUSG00000034312	150526	42,26
Itpkb	inositol 1,4,5-trisphosphate 3-kinase B	ENSMUSG00000038855	94318	42,26
Usp40	ubiquitin specific peptidase 40	ENSMUSG00000005501	63431	42,26
Perp	PERP, TP53 apoptosis effector	ENSMUSG00000019851	12003	42,26
Katnal2	katanin p60 subunit A-like 2	ENSMUSG00000025420	70161	42,26
Atm	ataxia telangiectasia mutated homolog (human)	ENSMUSG00000034218	97592	42,26
Tmem87a	transmembrane protein 87A	ENSMUSG00000033808	48802	42,26
Tmc4	transmembrane channel-like gene family 4	ENSMUSG00000019734	11735	42,26
Nphp1	nephronophthisis 1 (juvenile) homolog (human)	ENSMUSG00000027378	48166	42,19
Cog4	component of oligomeric golgi complex 4	ENSMUSG00000031753	35628	42,19
Srsf6	serine/arginine-rich splicing factor 6	ENSMUSG00000016921	5594	42,19
Msh2	mutS homolog 2 (E. coli)	ENSMUSG00000024151	51384	42,19
Yars	tyrosyl-tRNA synthetase	ENSMUSG00000028811	29848	42,19
Atp6v0a2	ATPase, H+ transporting, lysosomal V0 subunit A2	ENSMUSG00000038023	93078	42,19
Samd8	sterile alpha motif domain containing 8	ENSMUSG00000021770	48195	42,19
Plcl2	phospholipase C-like 2	ENSMUSG00000038910	178947	42,19
Cul2	cullin 2	ENSMUSG00000024231	53390	42,19
Slco1a6	solute carrier organic anion transporter family, member 1a6	ENSMUSG00000079262	122761	42,19
Lgals3	lectin, galactose binding, soluble 3	ENSMUSG00000050335	18410	42,19
Armc1	armadillo repeat containing 1	ENSMUSG00000027599	30920	42,12
Mmd	monocyte to macrophage differentiation-associated	ENSMUSG00000003948	90906	42,12

Wrnip1	Werner helicase interacting protein 1	ENSMUSG00000021400	20572	42,12
		ENSMUSG00000022674	41848	42,12
Nup62	nucleoporin 62	ENSMUSG00000043858	14722	42,12
Taf5l	TAF5-like RNA polymerase II, p300/CBP-associated factor (PCAF)-associated factor	ENSMUSG00000038697	24992	42,12
Dcaf13	DDB1 and CUL4 associated factor 13	ENSMUSG00000022300	33983	42,12
Mon1a	MON1 homolog A (yeast)	ENSMUSG00000032583	14997	42,12
		ENSMUSG00000039298	193588	42,12
Ago4	argonaute RISC catalytic subunit 4	ENSMUSG00000042500	43932	42,12
Fopnl	Fgfr1op N-terminal like	ENSMUSG00000022677	18132	42,04
Tmbim4	transmembrane BAX inhibitor motif containing 4	ENSMUSG00000020225	23328	42,04
Bbip1	BBSome interacting protein 1	ENSMUSG00000084957	14951	42,04
St5	suppression of tumorigenicity 5	ENSMUSG00000031024	93237	42,04
Zmat4	zinc finger, matrin type 4	ENSMUSG00000037492	520567	42,04
Fam118a	family with sequence similarity 118, member A	ENSMUSG00000022434	25767	41,97
		ENSMUSG00000066632	1731	41,97
Dlx5	distal-less homeobox 5	ENSMUSG00000029755	4281	41,97
F8	coagulation factor VIII	ENSMUSG00000031196	209901	41,97
Prpsap1	phosphoribosyl pyrophosphate synthetase-associated protein 1	ENSMUSG00000015869	23358	41,97
Arhgap44	Rho GTPase activating protein 44	ENSMUSG00000033389	160923	41,97
Sprr2a2	small proline-rich protein 2A2	ENSMUSG00000068893	41379	41,90
Ppid	peptidylprolyl isomerase D (cyclophilin D)	ENSMUSG00000027804	12309	41,83
Ciapin1	cytokine induced apoptosis inhibitor 1	ENSMUSG00000031781	18541	41,83
Crebzf	CREB/ATF bZIP transcription factor	ENSMUSG00000051451	4989	41,83
Bcor	BCL6 interacting corepressor	ENSMUSG00000040363	123616	41,83
Plcb1	phospholipase C, beta 1	ENSMUSG00000051177	689100	41,83
		ENSMUSG00000037601	11543	41,75
Mul1	mitochondrial ubiquitin ligase activator of NFKB 1	ENSMUSG00000041241	7595	41,75
Stambp	STAM binding protein	ENSMUSG00000006906	29299	41,75
Nt5c3	5'-nucleotidase, cytosolic III	ENSMUSG00000029780	41533	41,75
		ENSMUSG00000025792	7348	41,75
Wars	tryptophanyl-tRNA synthetase	ENSMUSG00000021266	34145	41,75
Klhl28	kelch-like 28	ENSMUSG00000020948	23097	41,75
9330159F19Rik	RIKEN cDNA 9330159F19 gene	ENSMUSG00000004360	19137	41,75
Ncf2	neutrophil cytosolic factor 2	ENSMUSG00000026480	29101	41,75
		ENSMUSG00000040904	4625	41,68
Snap29	synaptosomal-associated protein 29	ENSMUSG00000022765	24824	41,68
Srr	serine racemase	ENSMUSG00000001323	19590	41,68
Usp31	ubiquitin specific peptidase 31	ENSMUSG00000063317	65233	41,68
Heca	headcase homolog (Drosophila)	ENSMUSG00000039879	47602	41,68
3010026O09Rik	RIKEN cDNA 3010026O09 gene	ENSMUSG00000020381	25672	41,68
Rela	v-rel reticuloendotheliosis viral oncogene homolog A (avian)	ENSMUSG00000024927	10648	41,61
		ENSMUSG00000018378	96083	41,61
Ttc8	tetratricopeptide repeat domain 8	ENSMUSG00000021013	62665	41,61

Galnt11	UDP-N-acetyl-alpha-D-galactosamine:polypeptide N-acetylgalactosaminyltransferase 11	ENSMUSG00000038072	43037	41,61
Rps29	ribosomal protein S29	ENSMUSG00000034892	1465	41,61
Topbp1	topoisomerase (DNA) II binding protein 1	ENSMUSG00000032555	45218	41,61
Acsl6	acyl-CoA synthetase long-chain family member 6	ENSMUSG00000020333	60959	41,54
Tm7sf3	transmembrane 7 superfamily member 3	ENSMUSG00000040234	40473	41,54
Rpl30	ribosomal protein L30	ENSMUSG00000058600	3136	41,54
Skiv2l	superkiller viralicidic activity 2-like (S. cerevisiae)	ENSMUSG00000040356	10983	41,54
Trmt6	tRNA methyltransferase 6	ENSMUSG00000037376	11849	41,54
Vsig10	V-set and immunoglobulin domain containing 10	ENSMUSG00000066894	35740	41,54
Dhx29	DEAH (Asp-Glu-Ala-His) box polypeptide 29	ENSMUSG00000042426	41702	41,54
Casd1	CAS1 domain containing 1	ENSMUSG00000015189	42542	41,54
Chic1	cysteine-rich hydrophobic domain 1	ENSMUSG00000031327	39617	41,54
Mreg	melanoregulin	ENSMUSG00000039395	53075	41,46
		ENSMUSG00000017778	2089	41,46
Snn	stannin	ENSMUSG00000037972	8686	41,46
Gdap1	ganglioside-induced differentiation-associated-protein 1	ENSMUSG00000025777	18910	41,46
Zfp629	zinc finger protein 629	ENSMUSG00000045639	8767	41,46
Mrps16	mitochondrial ribosomal protein S16	ENSMUSG00000049960	2325	41,46
		ENSMUSG00000025903	78983	41,46
B4galt4	UDP-Gal:betaGlcNAc beta 1,4-galactosyltransferase, polypeptide 4	ENSMUSG00000022793	26786	41,46
Mpzl2	myelin protein zero-like 2	ENSMUSG00000032092	11591	41,46
Bahcc1	BAH domain and coiled-coil containing 1	ENSMUSG00000039741	59350	41,46
Mrps17	mitochondrial ribosomal protein S17	ENSMUSG00000034211	3379	41,39
Ddx54	DEAD (Asp-Glu-Ala-Asp) box polypeptide 54	ENSMUSG00000029599	15461	41,39
Stk38l	serine/threonine kinase 38 like	ENSMUSG00000001630	53818	41,39
Glb1	galactosidase, beta 1	ENSMUSG00000045594	73301	41,39
Snx4	sorting nexin 4	ENSMUSG00000022808	48107	41,39
Pdik1l	PDLIM1 interacting kinase 1 like	ENSMUSG00000050890	12894	41,39
Chchd7	coiled-coil-helix-coiled-coil-helix domain containing 7	ENSMUSG00000042198	12159	41,39
BC052040	cDNA sequence BC052040	ENSMUSG00000040282	197053	41,39
Ppargc1a	peroxisome proliferative activated receptor, gamma, coactivator 1 alpha	ENSMUSG00000029167	113477	41,32
Dcp2	DCP2 decapping enzyme homolog (S. cerevisiae)	ENSMUSG00000024472	44470	41,32
Mtif2	mitochondrial translational initiation factor 2	ENSMUSG00000020459	18872	41,32
Sfn	stratifin	ENSMUSG00000047281	1613	41,32
Morn1	MORN repeat containing 1	ENSMUSG00000029049	58929	41,32

		ENSMUSG00000053702	387556	41,32
Ndfip1	Nedd4 family interacting protein 1	ENSMUSG00000024425	45425	41,32
Chmp2a	charged multivesicular body protein 2A	ENSMUSG00000033916	2772	41,25
Gemin5	gem (nuclear organelle) associated protein 5	ENSMUSG00000037275	48538	41,25
Stam2	signal transducing adaptor molecule (SH3 domain and ITAM motif) 2	ENSMUSG00000055371	50618	41,25
Dnah7a	dynein, axonemal, heavy chain 7A	ENSMUSG00000096141	309783	41,25
Gm609	predicted gene 609	ENSMUSG00000053182	81202	41,17
Ndufa9	NADH dehydrogenase (ubiquinone) 1 alpha subcomplex, 9	ENSMUSG00000000399	27282	41,17
Cacybp	calcyclin binding protein	ENSMUSG00000014226	10526	41,17
Zpbp	zona pellucida binding protein	ENSMUSG00000020193	182783	41,17
Akap11	A kinase (PRKA) anchor protein 11	ENSMUSG00000022016	44615	41,17
Slc2a1	solute carrier family 2 (facilitated glucose transporter), member 1	ENSMUSG00000028645	28620	41,17
Phc3	polyhomeotic-like 3 (Drosophila)	ENSMUSG00000037652	70121	41,17
Preb	prolactin regulatory element binding	ENSMUSG00000045302	9478	41,10
Fbxo21	F-box protein 21	ENSMUSG00000032898	33422	41,10
Ppp1r1a	protein phosphatase 1, regulatory (inhibitor) subunit 1A	ENSMUSG00000022490	7714	41,10
Zfp518b	zinc finger protein 518B	ENSMUSG00000046572	16343	41,10
Kirrel2	kin of IRRE like 2 (Drosophila)	ENSMUSG00000036915	10157	41,03
Dnajc9	DnaJ (Hsp40) homolog, subfamily C, member 9	ENSMUSG00000021811	4273	41,03
		ENSMUSG00000022538	27099	41,03
Lrrc3b	leucine rich repeat containing 3B	ENSMUSG00000045201	81469	41,03
Ncbp1	nuclear cap binding protein subunit 1	ENSMUSG00000028330	33791	41,03
Tmem176b	transmembrane protein 176B	ENSMUSG00000029810	7564	41,03
Paip2b	poly(A) binding protein interacting protein 2B	ENSMUSG00000045896	26341	41,03
Coro2b	coronin, actin binding protein, 2B	ENSMUSG00000041729	117553	41,03
Cyp39a1	cytochrome P450, family 39, subfamily a, polypeptide 1	ENSMUSG00000023963	84007	41,03
Rttn	rotatin	ENSMUSG00000023066	159224	41,03
		ENSMUSG00000029559	7626	40,96
Prmt2	protein arginine N-methyltransferase 2	ENSMUSG00000020230	30644	40,96
Pdhb	pyruvate dehydrogenase (lipoamide) beta	ENSMUSG00000021748	7002	40,96
Nmnat2	nicotinamide nucleotide adenylyltransferase 2	ENSMUSG00000042751	164160	40,96
		ENSMUSG00000025912	32795	40,96
Ipo8	importin 8	ENSMUSG00000040029	60785	40,96
Fam46a	family with sequence similarity 46, member A	ENSMUSG00000032265	6686	40,96
Bex2	brain expressed X-linked 2	ENSMUSG00000042750	1673	40,88
Rars	arginyl-tRNA synthetase	ENSMUSG00000018848	26126	40,88
		ENSMUSG00000005362	21826	40,88
Btbd8	BTB (POZ) domain containing 8	ENSMUSG00000070632	53632	40,88
S100a5	S100 calcium binding protein A5	ENSMUSG00000001023	3258	40,81
Jtb	jumping translocation breakpoint	ENSMUSG00000027937	4242	40,81

Tcf3	transcription factor 3	ENSMUSG00000020167	24134	40,81
Ccdc40	coiled-coil domain containing 40	ENSMUSG00000039963	36665	40,81
Glud1	glutamate dehydrogenase 1	ENSMUSG00000021794	34418	40,81
1110059E24Rik	RIKEN cDNA 1110059E24 gene	ENSMUSG00000035171	71775	40,81
Ppargc1b	peroxisome proliferative activated receptor, gamma, coactivator 1 beta	ENSMUSG00000033871	102296	40,81
Marveld3	MARVEL (membrane-associating) domain containing 3	ENSMUSG00000001672	14290	40,81
Ppp1cc	protein phosphatase 1, catalytic subunit, gamma isoform	ENSMUSG00000004455	16996	40,74
Atxn3	ataxin 3	ENSMUSG00000021189	39346	40,74
Ap1g2	adaptor protein complex AP-1, gamma 2 subunit	ENSMUSG00000040701	8016	40,74
Galk2	galactokinase 2	ENSMUSG00000027207	125191	40,74
Snx17	sorting nexin 17	ENSMUSG00000029146	5674	40,67
Mief1	mitochondrial elongation factor 1	ENSMUSG00000022412	19287	40,67
Mvp	major vault protein	ENSMUSG00000030681	27762	40,67
Clpb	ClpB caseinolytic peptidase B	ENSMUSG00000001829	126536	40,67
		ENSMUSG00000034460	14429	40,67
Sergef	secretion regulating guanine nucleotide exchange factor	ENSMUSG00000030839	196640	40,67
		ENSMUSG00000003037	22705	40,59
Iah1	isoamyl acetate-hydrolyzing esterase 1 homolog (S. cerevisiae)	ENSMUSG00000062054	7214	40,59
Gltscr1l	GLTSCR1-like	ENSMUSG00000036568	33298	40,59
Aifm1	apoptosis-inducing factor, mitochondrion-associated 1	ENSMUSG00000036932	38620	40,59
Ispd	isoprenoid synthase domain containing	ENSMUSG00000043153	307926	40,59
Lsamp	limbic system-associated membrane protein	ENSMUSG00000061080	648822	40,59
Rpl9	ribosomal protein L9	ENSMUSG00000047215	3080	40,52
Amz2	archaelysin family metallopeptidase 2	ENSMUSG00000020610	26868	40,52
		ENSMUSG00000047040	9727	40,52
		ENSMUSG00000028572	58174	40,52
Map2k6	mitogen-activated protein kinase kinase 6	ENSMUSG00000020623	126401	40,52
Slc5a11	solute carrier family 5 (sodium/glucose cotransporter), member 11	ENSMUSG00000030769	58474	40,52
Cdkn2d	cyclin-dependent kinase inhibitor 2D (p19, inhibits CDK4)	ENSMUSG00000096472	2747	40,45
Cald1	caldesmon 1	ENSMUSG00000029761	176974	40,45
		ENSMUSG00000006289	18416	40,45
3830406C13Rik	RIKEN cDNA 3830406C13 gene	ENSMUSG00000033111	19023	40,45
Mplkip	M-phase specific PLK1 intereacting protein	ENSMUSG00000012429	3693	40,45
Dip2a	DIP2 disco-interacting protein 2 homolog A (Drosophila)	ENSMUSG00000020231	85863	40,45
Efcab5	EF-hand calcium binding domain 5	ENSMUSG00000050944	99054	40,45
Mpv17l	Mpv17 transgene, kidney disease mutant-like	ENSMUSG00000022679	46459	40,45
Fam83g	family with sequence similarity 83, member G	ENSMUSG00000042377	25861	40,45

Kat8	K(lysine) acetyltransferase 8	ENSMUSG00000030801	13316	40,45
Ndufa8	NADH dehydrogenase (ubiquinone) 1 alpha subcomplex, 8	ENSMUSG00000026895	13081	40,38
Crybb2	crystallin, beta B2	ENSMUSG00000042240	11860	40,38
Maoa	monoamine oxidase A	ENSMUSG00000025037	68121	40,38
Mtmr1	myotubularin related protein 1	ENSMUSG00000015214	54437	40,38
Adrbk2	adrenergic receptor kinase, beta 2	ENSMUSG00000042249	105037	40,38
Yes1	Yamaguchi sarcoma viral (v-yes) oncogene homolog 1	ENSMUSG00000014932	75896	40,38
Reep5	receptor accessory protein 5	ENSMUSG00000005873	28527	40,30
Zmiz2	zinc finger, MIZ-type containing 2	ENSMUSG00000041164	17085	40,30
Rbmx	RNA binding motif protein, X chromosome	ENSMUSG00000031134	9507	40,30
Lrrc51	leucine rich repeat containing 51	ENSMUSG00000064307	20912	40,30
Rcan1	regulator of calcineurin 1	ENSMUSG00000022951	74194	40,30
Itgb5	integrin beta 5	ENSMUSG00000022817	119489	40,30
Suclg2	succinate-Coenzyme A ligase, GDP-forming, beta subunit	ENSMUSG00000061838	244704	40,30
Tmem232	transmembrane protein 232	ENSMUSG00000045036	284778	40,30
Fam184b	family with sequence similarity 184, member B	ENSMUSG00000015879	109796	40,30
Gemin7	gem (nuclear organelle) associated protein 7	ENSMUSG00000044709	8878	40,23
Dtx4	deltex 4 homolog (Drosophila)	ENSMUSG00000039982	35656	40,23
Kif9	kinesin family member 9	ENSMUSG00000032489	48186	40,23
Clcn7	chloride channel 7	ENSMUSG00000036636	28714	40,23
Brf1	BRF1 homolog, subunit of RNA polymerase III transcription initiation factor IIIB (S. cerevisiae)	ENSMUSG00000011158	40760	40,23
Fancc	Fanconi anemia, complementation group C	ENSMUSG00000021461	192570	40,23
Pdhx	pyruvate dehydrogenase complex, component X	ENSMUSG00000010914	52439	40,16
Rdh14	retinol dehydrogenase 14 (all-trans and 9-cis)	ENSMUSG00000020621	4783	40,16
		ENSMUSG00000039316	198418	40,16
Mlh1	mutL homolog 1 (E. coli)	ENSMUSG00000032498	43381	40,16
Zfp120	zinc finger protein 120	ENSMUSG00000068134	22303	40,16
Stk16	serine/threonine kinase 16	ENSMUSG00000026201	4769	40,09
Prpf4	PRP4 pre-mRNA processing factor 4 homolog (yeast)	ENSMUSG00000066148	18194	40,09
Gnai3	guanine nucleotide binding protein (G protein), alpha inhibiting 3	ENSMUSG00000000001	38867	40,09
Cdkl3	cyclin-dependent kinase-like 3	ENSMUSG00000020389	85564	40,09
Cdk17	cyclin-dependent kinase 17	ENSMUSG00000020015	80467	40,09
Ufd1l	ubiquitin fusion degradation 1 like	ENSMUSG00000005262	23483	40,01
Maml1	mastermind like 1 (Drosophila)	ENSMUSG00000050567	36678	40,01
Epg5	ectopic P-granules autophagy protein 5 homolog (C. elegans)	ENSMUSG00000039840	96561	40,01
Mcph1	microcephaly, primary autosomal recessive 1	ENSMUSG00000039842	208059	40,01
Timm17a	translocase of inner mitochondrial membrane 17a	ENSMUSG00000062580	12247	39,94
Mycbp	c-myc binding protein	ENSMUSG00000028647	7438	39,94
Sike1	suppressor of IKBKE 1	ENSMUSG00000027854	8187	39,94

Mrpl44	mitochondrial ribosomal protein L44	ENSMUSG00000026248	5428	39,94
Mdm1	transformed mouse 3T3 cell double minute 1	ENSMUSG00000020212	27211	39,94
Coq5	coenzyme Q5 homolog, methyltransferase (yeast)	ENSMUSG00000041733	17307	39,94
		ENSMUSG00000035142	215008	39,94
2610203C22Rik	RIKEN cDNA 2610203C22 gene	ENSMUSG00000079671	70344	39,87
Rcan3	regulator of calcineurin 3	ENSMUSG00000059713	21546	39,87
Stip1	stress-induced phosphoprotein 1	ENSMUSG00000024966	19331	39,87
Rpgrip1l	Rpgrip1-like	ENSMUSG00000033282	96233	39,87
Adar	adenosine deaminase, RNA-specific	ENSMUSG00000027951	38425	39,87
Tgif2	TGFB-induced factor homeobox 2	ENSMUSG00000062175	15494	39,80
Dpp9	dipeptidylpeptidase 9	ENSMUSG00000001229	32208	39,80
Stx16	syntaxin 16	ENSMUSG00000027522	23464	39,80
Cdc25a	cell division cycle 25A	ENSMUSG00000032477	18310	39,80
Dhx57	DEAH (Asp-Glu-Ala-Asp/His) box polypeptide 57	ENSMUSG00000035051	52173	39,80
Pdcd10	programmed cell death 10	ENSMUSG00000027835	40367	39,80
Aph1c	anterior pharynx defective 1c homolog (C. elegans)	ENSMUSG00000053040	19733	39,80
Tlr4	toll-like receptor 4	ENSMUSG00000039005	102701	39,80
		ENSMUSG00000022634	52658	39,80
Stx18	syntaxin 18	ENSMUSG00000029125	99024	39,72
Tmem218	transmembrane protein 218	ENSMUSG00000032121	14878	39,72
Edc4	enhancer of mRNA decapping 4	ENSMUSG00000036270	14028	39,72
Wsb2	WD repeat and SOCS box-containing 2	ENSMUSG00000029364	21298	39,72
Rhobtb2	Rho-related BTB domain containing 2	ENSMUSG00000022075	20541	39,72
Crabp2	cellular retinoic acid binding protein II	ENSMUSG00000004885	4711	39,72
Ublcp1	ubiquitin-like domain containing CTD phosphatase 1	ENSMUSG00000041231	15928	39,72
Trappc8	trafficking protein particle complex 8	ENSMUSG00000033382	78855	39,72
Rpl7l1	ribosomal protein L7-like 1	ENSMUSG00000063888	8750	39,65
Ttbk1	tau tubulin kinase 1	ENSMUSG00000015599	45228	39,65
Zranb2	zinc finger, RAN-binding domain containing 2	ENSMUSG00000028180	14231	39,65
Atp1b3	ATPase, Na+/K+ transporting, beta 3 polypeptide	ENSMUSG00000032412	31620	39,65
Shc1	src homology 2 domain-containing transforming protein C1	ENSMUSG00000042626	11573	39,65
Trappc12	trafficking protein particle complex 12	ENSMUSG00000020628	59825	39,65
Cluh	clustered mitochondria (cluA/CLU1) homolog	ENSMUSG00000020741	21353	39,65
Slc30a7	solute carrier family 30 (zinc transporter), member 7	ENSMUSG00000054414	68434	39,65
Cdhr1	cadherin-related family member 1	ENSMUSG00000021803	20462	39,58
Map3k13	mitogen-activated protein kinase kinase kinase 13	ENSMUSG00000033618	39909	39,58
Kin	antigenic determinant of rec-A protein	ENSMUSG00000037262	12214	39,51

		ENSMUSG00000006057	6819	39,51
Naf1	nuclear assembly factor 1 homolog (S. cerevisiae)	ENSMUSG00000014907	30348	39,51
Tmem175	transmembrane protein 175	ENSMUSG00000013495	18001	39,51
Gtf2h2	general transcription factor II H, polypeptide 2	ENSMUSG00000021639	32362	39,51
Aar2	AAR2 splicing factor homolog (S. cerevisiae)	ENSMUSG00000027628	21389	39,43
Zfp1	zinc finger protein 1	ENSMUSG00000055835	27566	39,43
Prss8	protease, serine, 8 (prostasin)	ENSMUSG00000030800	4389	39,43
Gyk	glycerol kinase	ENSMUSG00000025059	74883	39,43
Mrpl54	mitochondrial ribosomal protein L54	ENSMUSG00000034932	2222	39,36
Edf1	endothelial differentiation-related factor 1	ENSMUSG00000015092	4236	39,36
Ndufaf4	NADH dehydrogenase (ubiquinone) 1 alpha subcomplex, assembly factor 4	ENSMUSG00000028261	6919	39,36
		ENSMUSG00000036555	40552	39,36
Nfatc1	nuclear factor of activated T cells, cytoplasmic, calcineurin dependent 1	ENSMUSG00000033016	106867	39,36
Dgcr8	DiGeorge syndrome critical region gene 8	ENSMUSG00000022718	35302	39,36
Catsperb	catsper channel auxiliary subunit beta	ENSMUSG00000047014	221337	39,36
Arl6	ADP-ribosylation factor-like 6	ENSMUSG00000022722	26443	39,29
Rnf44	ring finger protein 44	ENSMUSG00000034928	14509	39,29
Adgb	androglobin	ENSMUSG00000050994	136624	39,29
Fnip2	folliculin interacting protein 2	ENSMUSG00000061175	111826	39,29
Slc6a20a	solute carrier family 6 (neurotransmitter transporter), member 20A	ENSMUSG00000036814	42764	39,29
Rragc	Ras-related GTP binding C	ENSMUSG00000028646	19552	39,22
Smg9	smg-9 homolog, nonsense mediated mRNA decay factor (C. elegans)	ENSMUSG00000002210	23160	39,22
Ccdc30	coiled-coil domain containing 30	ENSMUSG00000028637	92629	39,22
Dync1i1	dynein cytoplasmic 1 intermediate chain 1	ENSMUSG00000029757	302401	39,22
Nfyc	nuclear transcription factor-Y gamma	ENSMUSG00000032897	74135	39,22
C030046E11Rik	RIKEN cDNA C030046E11 gene	ENSMUSG00000038658	84548	39,22
Gabbr1	gamma-aminobutyric acid (GABA) B receptor, 1	ENSMUSG00000024462	29102	39,14
Hint2	histidine triad nucleotide binding protein 2	ENSMUSG00000028470	2240	39,14
Sbf1	SET binding factor 1	ENSMUSG00000036529	27076	39,14
Tbc1d23	TBC1 domain family, member 23	ENSMUSG00000022749	62601	39,14
Map3k14	mitogen-activated protein kinase kinase kinase 14	ENSMUSG00000020941	47711	39,14
Apoe	apolipoprotein E	ENSMUSG00000002985	3058	39,07
Tmem231	transmembrane protein 231	ENSMUSG00000031951	21774	39,07
		ENSMUSG00000026753	32098	39,07
Acad8	acyl-Coenzyme A dehydrogenase family, member 8	ENSMUSG00000031969	25432	39,07

Ubxn2a	UBX domain protein 2A	ENSMUSG00000020634	28674	39,07
Phtf2	putative homeodomain transcription factor 2	ENSMUSG00000039987	123462	39,07
S100a14	S100 calcium binding protein A14	ENSMUSG00000042306	1989	39,07
Ccdc162	coiled-coil domain containing 162	ENSMUSG00000075225	170455	39,07
Ngrn	neugrin, neurite outgrowth associated	ENSMUSG00000047084	4160	39,00
Fank1	fibronectin type 3 and ankyrin repeat domains 1	ENSMUSG00000053111	104672	39,00
Slc17a5	solute carrier family 17 (anion/sugar transporter), member 5	ENSMUSG00000049624	51554	39,00
Plcd3	phospholipase C, delta 3	ENSMUSG00000020937	31355	39,00
Tbc1d12	TBC1D12: TBC1 domain family, member 12	ENSMUSG00000048720	83345	39,00
Dctn5	dynactin 5	ENSMUSG00000030868	16004	38,93
Oser1	oxidative stress responsive serine rich 1	ENSMUSG00000035399	18876	38,93
Dpcd	deleted in primary ciliary dyskinesia	ENSMUSG00000041035	17665	38,93
		ENSMUSG00000075254	87111	38,93
Nub1	negative regulator of ubiquitin-like proteins 1	ENSMUSG00000028954	24741	38,93
Ptch1	patched homolog 1	ENSMUSG00000021466	53988	38,93
		ENSMUSG00000027957	43363	38,93
		ENSMUSG00000069049	18305	38,93
		ENSMUSG00000001911	100469	38,85
Mdp1	magnesium-dependent phosphatase 1	ENSMUSG00000002329	2630	38,85
Mtmr10	myotubularin related protein 10	ENSMUSG00000030522	52755	38,85
Npat	nuclear protein in the AT region	ENSMUSG00000033054	37296	38,85
Timp2	tissue inhibitor of metalloproteinase 2	ENSMUSG00000017466	54672	38,78
Slco3a1	solute carrier organic anion transporter family, member 3a1	ENSMUSG00000025790	279362	38,78
Eif6	eukaryotic translation initiation factor 6	ENSMUSG00000027613	7094	38,78
Afg3l2	AFG3(ATPase family gene 3)-like 2 (yeast)	ENSMUSG00000024527	44400	38,78
Tet1	tet methylcytosine dioxygenase 1	ENSMUSG00000047146	94549	38,78
2310036O22Rik	RIKEN cDNA 2310036O22 gene	ENSMUSG00000041203	3453	38,78
Hspe1	heat shock protein 1 (chaperonin 10)	ENSMUSG00000073676	3176	38,78
Ube2j2	ubiquitin-conjugating enzyme E2J 2	ENSMUSG00000023286	15774	38,78
Ints9	integrator complex subunit 9	ENSMUSG00000021975	89791	38,78
Prkag2	protein kinase, AMP-activated, gamma 2 non-catalytic subunit	ENSMUSG00000028944	237899	38,78
Dcbld1	discoidin, CUB and LCCL domain containing 1	ENSMUSG00000019891	87760	38,78
Slco1a5	solute carrier organic anion transporter family, member 1a5	ENSMUSG00000063975	88755	38,78
Unc5c	unc-5 homolog C (C. elegans)	ENSMUSG00000059921	369361	38,78
		ENSMUSG00000023075	15787	38,71
Aldoc	aldolase C, fructose-bisphosphate	ENSMUSG00000017390	4814	38,71
Dclk2	doublecortin-like kinase 2	ENSMUSG00000028078	134735	38,71

Lrp4	low density lipoprotein receptor-related protein 4	ENSMUSG00000027253	56269	38,71
Mroh5	maestro heat-like repeat family member 5	ENSMUSG00000072487	78290	38,64
Ndufb11	NADH dehydrogenase (ubiquinone) 1 beta subcomplex, 11	ENSMUSG00000031059	2294	38,64
Txn2	thioredoxin 2	ENSMUSG00000005354	13960	38,64
Vamp4	vesicle-associated membrane protein 4	ENSMUSG00000026696	28570	38,64
Prpf18	PRP18 pre-mRNA processing factor 18 homolog (yeast)	ENSMUSG00000039449	30056	38,64
Ak3	adenylate kinase 3	ENSMUSG00000024782	27129	38,64
		ENSMUSG00000048379	19168	38,64
Lekr1	leucine, glutamate and lysine rich 1	ENSMUSG00000074579	164932	38,64
Atr	ataxia telangiectasia and Rad3 related	ENSMUSG00000032409	94048	38,64
Rprm	reprimo, TP53 dependent G2 arrest mediator candidate	ENSMUSG00000075334	1460	38,56
Yipf4	Yip1 domain family, member 4	ENSMUSG00000024072	10785	38,56
Sec11c	SEC11 homolog C (S. cerevisiae)	ENSMUSG00000024516	17117	38,56
Vps4a	vacuolar protein sorting 4a (yeast)	ENSMUSG00000031913	14534	38,56
Aup1	ancient ubiquitous protein 1	ENSMUSG00000068328	3028	38,56
		ENSMUSG00000026771	65887	38,56
Pxk	PX domain containing serine/threonine kinase	ENSMUSG00000033885	66899	38,56
		ENSMUSG00000096401	14040	38,56
Clpx	caseinolytic peptidase X (E.coli)	ENSMUSG00000015357	36399	38,56
		ENSMUSG00000032116	36405	38,49
Bcar1	breast cancer anti-estrogen resistance 1	ENSMUSG00000031955	33365	38,49
2410015M20Rik	RIKEN cDNA 2410015M20 gene	ENSMUSG00000049760	2320	38,49
Btf3l4	basic transcription factor 3-like 4	ENSMUSG00000028568	19320	38,49
Mcur1	mitochondrial calcium uniporter regulator 1	ENSMUSG00000021371	21780	38,49
Arfrp1	ADP-ribosylation factor related protein 1	ENSMUSG00000038671	7715	38,49
Tmem55b	transmembrane protein 55b	ENSMUSG00000035953	4787	38,42
Clpp	ClpP caseinolytic peptidase, ATP-dependent, proteolytic subunit	ENSMUSG00000002660	6108	38,42
Zfp316	zinc finger protein 316	ENSMUSG00000046658	20326	38,42
Clcc1	chloride channel CLIC-like 1	ENSMUSG00000027884	24928	38,42
Vps45	vacuolar protein sorting 45 (yeast)	ENSMUSG00000015747	58635	38,42
Apobec3	apolipoprotein B mRNA editing enzyme, catalytic polypeptide 3	ENSMUSG00000009585	24248	38,42
Abca16	ATP-binding cassette, sub-family A (ABC1), member 16	ENSMUSG00000051900	135167	38,42
Lrrcc1	leucine rich repeat and coiled-coil domain containing 1	ENSMUSG00000027550	38871	38,42
Arl8a	ADP-ribosylation factor-like 8A	ENSMUSG00000026426	9446	38,35
Wdr38	WD repeat domain 38	ENSMUSG00000035295	8693	38,35
Kbtbd4	kelch repeat and BTB (POZ) domain containing 4	ENSMUSG00000005505	6887	38,35
Klc2	kinesin light chain 2	ENSMUSG00000024862	10815	38,35
Ccdc181	coiled-coil domain containing 181	ENSMUSG00000026578	12263	38,35
Cnnm3	cyclin M3	ENSMUSG00000001138	16371	38,35

Emc2	ER membrane protein complex subunit 2	ENSMUSG00000022337	50549	38,27
Sv2a	synaptic vesicle glycoprotein 2 a	ENSMUSG00000038486	14371	38,27
		ENSMUSG00000039826	27279	38,27
Mcat	malonyl CoA:ACP acyltransferase (mitochondrial)	ENSMUSG00000048755	8915	38,27
Igbp1	immunoglobulin (CD79A) binding protein 1	ENSMUSG00000031221	21835	38,27
Cnot8	CCR4-NOT transcription complex, subunit 8	ENSMUSG00000020515	14442	38,27
Stam	signal transducing adaptor molecule (SH3 domain and ITAM motif) 1	ENSMUSG00000026718	74239	38,27
Ltn1	listerin E3 ubiquitin protein ligase 1	ENSMUSG00000052299	55956	38,27
Prc1	protein regulator of cytokinesis 1	ENSMUSG00000038943	21810	38,27
Plekhm1	pleckstrin homology domain containing, family M (with RUN domain) member 1	ENSMUSG00000034247	47577	38,27
Zfp7	zinc finger protein 7	ENSMUSG00000033669	13134	38,27
Hdac9	histone deacetylase 9	ENSMUSG00000004698	157387	38,27
Bdh1	3-hydroxybutyrate dehydrogenase, type 1	ENSMUSG00000046598	36622	38,20
Ppdpf	pancreatic progenitor cell differentiation and proliferation factor homolog (zebrafish)RIKEN cDNA 2700038C09 gene	ENSMUSG00000016344	1525	38,20
C130060K24Rik	RIKEN cDNA C130060K24 gene	ENSMUSG00000029917	77046	38,20
Gdpd5	glycerophosphodiester phosphodiesterase domain containing 5	ENSMUSG00000035314	79435	38,20
Hip1	huntingtin interacting protein 1	ENSMUSG00000039959	138600	38,20
Clcn6	chloride channel 6	ENSMUSG00000029016	34563	38,20
Tmc5	transmembrane channel-like gene family 5	ENSMUSG00000030650	77790	38,20
Xpo6	exportin 6	ENSMUSG00000000131	98787	38,20
Lrrc8b	leucine rich repeat containing 8 family, member B	ENSMUSG00000070639	70415	38,20
Neurl4	neuralized homolog 4 (Drosophila)	ENSMUSG00000047284	12749	38,13
Cops2	COP9 (constitutive photomorphogenic) homolog, subunit 2 (Arabidopsis thaliana)	ENSMUSG00000027206	28836	38,13
		ENSMUSG00000092541	49185	38,13
Pcsk6	proprotein convertase subtilisin/kexin type 6	ENSMUSG00000030513	188607	38,13
Plk1s1	polo-like kinase 1 substrate 1	ENSMUSG00000074749	114234	38,13
Banf1	barrier to autointegration factor 1	ENSMUSG00000024844	2005	38,06
Zfp637	zinc finger protein 637	ENSMUSG00000059689	4777	38,06
Emc1	ER membrane protein complex subunit 1	ENSMUSG00000078517	26144	38,06
Tmem161a	transmembrane protein 161A	ENSMUSG00000002342	11326	38,06
Prkaa1	protein kinase, AMP-activated, alpha 1 catalytic subunit	ENSMUSG00000050697	38039	38,06
Gmds	GDP-mannose 4, 6-dehydratase	ENSMUSG00000038372	519134	38,06
Snx16	sorting nexin 16	ENSMUSG00000027534	22306	38,06
Tulp3	tubby-like protein 3	ENSMUSG00000001521	34712	37,98

Uchl5	ubiquitin carboxyl-terminal esterase L5	ENSMUSG00000018189	30189	37,98
Vldlr	very low density lipoprotein receptor	ENSMUSG00000024924	37748	37,98
Gss	glutathione synthetase	ENSMUSG00000027610	29630	37,98
Taf1d	TATA box binding protein (Tbp)-associated factor, RNA polymerase I, D	ENSMUSG00000031939	5891	37,98
		ENSMUSG00000055301	15560	37,98
Sult1e1	sulfotransferase family 1E, member 1	ENSMUSG00000029272	15644	37,91
Cdc34	cell division cycle 34	ENSMUSG00000020307	6200	37,91
Myt1	myelin transcription factor 1	ENSMUSG00000010505	64466	37,91
Phf2	PHD finger protein 2	ENSMUSG00000038025	69136	37,91
Sec23ip	Sec23 interacting protein	ENSMUSG00000055319	39967	37,91
Hnrnpr	heterogeneous nuclear ribonucleoprotein R	ENSMUSG00000066037	32027	37,91
Rbms2	RNA binding motif, single stranded interacting protein 2	ENSMUSG00000040043	50828	37,91
Tpp2	tripeptidyl peptidase II	ENSMUSG00000041763	68998	37,91
		ENSMUSG00000032203	7177	37,91
Sec23a	SEC23A (S. cerevisiae)	ENSMUSG00000020986	53635	37,91
8030462N17Rik	RIKEN cDNA 8030462N17 gene	ENSMUSG00000047466	80730	37,91
Gar1	GAR1 ribonucleoprotein homolog (yeast)	ENSMUSG00000028010	6485	37,84
Trp53bp2	transformation related protein 53 binding protein 2	ENSMUSG00000026510	53261	37,84
Col23a1	collagen, type XXIII, alpha 1	ENSMUSG00000063564	293999	37,77
Bcdin3d	BCDIN3 domain containing	ENSMUSG00000037525	4647	37,77
Gmfb	glia maturation factor, beta	ENSMUSG00000062014	14094	37,77
Rnasek	ribonuclease, RNase K	ENSMUSG00000093989	1719	37,77
Fam98b	family with sequence similarity 98, member B	ENSMUSG00000027349	21802	37,77
Rbm18	RNA binding motif protein 18	ENSMUSG00000026889	20695	37,77
Sgcd	sarcoglycan, delta (dystrophin-associated glycoprotein)	ENSMUSG00000020354	1092717	37,77
Dnajb13	DnaJ (Hsp40) related, subfamily B, member 13	ENSMUSG00000030708	11943	37,77
Trim26	tripartite motif-containing 26	ENSMUSG00000024457	22265	37,77
Chchd10	coiled-coil-helix-coiled-coil-helix domain containing 10	ENSMUSG00000049422	2153	37,69
Tspyl4	TSPY-like 4	ENSMUSG00000039485	3888	37,69
Mcfd2	multiple coagulation factor deficiency 2	ENSMUSG00000024150	11278	37,69
Tmem11	transmembrane protein 11	ENSMUSG00000043284	14821	37,69
Otud6b	OTU domain containing 6B	ENSMUSG00000040550	17090	37,69
Ccnjl	cyclin J-like	ENSMUSG00000044707	58214	37,69
Golga3	golgi autoantigen, golgin subfamily a, 3	ENSMUSG00000029502	49770	37,69
Pip5k1b	phosphatidylinositol-4-phosphate 5-kinase, type 1 beta	ENSMUSG00000024867	261079	37,69
1500012F01Rik	RIKEN cDNA 1500012F01 gene	ENSMUSG00000074578	2929	37,69
Cdc42bpb	CDC42 binding protein kinase beta	ENSMUSG00000021279	84747	37,69
Caap1	caspase activity and apoptosis inhibitor 1	ENSMUSG00000028578	56716	37,69

Eif2b1	eukaryotic translation initiation factor 2B, subunit 1 (alpha)	ENSMUSG00000029388	8919	37,62
Sec24c	Sec24 related gene family, member C (S. cerevisiae)	ENSMUSG00000039367	20530	37,62
Stx17	syntaxin 17	ENSMUSG00000061455	61593	37,62
Mxd1	MAX dimerization protein 1	ENSMUSG00000001156	22116	37,62
Asb3	ankyrin repeat and SOCS box-containing 3	ENSMUSG00000020305	148310	37,62
Tbk1	TANK-binding kinase 1	ENSMUSG00000020115	40340	37,62
Gmpr	guanosine monophosphate reductase	ENSMUSG00000000253	38939	37,62
Rhno1	RAD9-HUS1-RAD1 interacting nuclear orphan 1	ENSMUSG00000048668	5912	37,55
Zfp36l2	zinc finger protein 36, C3H type-like 2	ENSMUSG00000045817	4017	37,55
Leo1	Leo1, Paf1/RNA polymerase II complex component, homolog (S. cerevisiae)	ENSMUSG00000042487	24909	37,55
Got1	glutamate oxaloacetate transaminase 1, soluble	ENSMUSG00000025190	24854	37,55
Prkci	protein kinase C, iota	ENSMUSG00000037643	56995	37,55
Scaf1	SR-related CTD-associated factor 1	ENSMUSG00000038406	13416	37,55
2700081O15Rik	RIKEN cDNA 2700081O15 gene	ENSMUSG00000053080	8280	37,55
Thumpd3	THUMP domain containing 3	ENSMUSG00000030264	22049	37,55
Armc10	armadillo repeat containing 10	ENSMUSG00000038525	16882	37,48
Cnrip1	cannabinoid receptor interacting protein 1	ENSMUSG00000044629	27786	37,48
		ENSMUSG00000022370	23655	37,48
Wdr35	WD repeat domain 35	ENSMUSG00000066643	54956	37,48
Gga3	golgi associated, gamma adaptin ear containing, ARF binding protein 3	ENSMUSG00000020740	19797	37,48
Naa20	N(alpha)-acetyltransferase 20, NatB catalytic subunit	ENSMUSG00000002728	14327	37,48
Stat2	signal transducer and activator of transcription 2	ENSMUSG00000040033	22274	37,48
Trak2	trafficking protein, kinesin binding 2	ENSMUSG00000026028	72982	37,48
		ENSMUSG00000039131	72698	37,48
Aldh4a1	aldehyde dehydrogenase 4 family, member A1	ENSMUSG00000028737	26825	37,40
Scyl2	SCY1-like 2 (S. cerevisiae)	ENSMUSG00000069539	47565	37,40
Farp2	FERM, RhoGEF and pleckstrin domain protein 2	ENSMUSG00000034066	109898	37,40
Frem2	Fras1 related extracellular matrix protein 2	ENSMUSG00000037016	143417	37,40
Tmem47	transmembrane protein 47	ENSMUSG00000025666	27175	37,40
E2f3	E2F transcription factor 3	ENSMUSG00000016477	79094	37,40
Mrps21	mitochondrial ribosomal protein S21	ENSMUSG00000054312	8886	37,33
		ENSMUSG00000058503	25967	37,33
Gpr98	G protein-coupled receptor 98	ENSMUSG00000069170	538087	37,33
Prss36	protease, serine, 36	ENSMUSG00000070371	14088	37,33
Banf2	barrier to autointegration factor 2	ENSMUSG00000037307	40921	37,33
Pes1	pescadillo homolog 1, containing BRCT domain (zebrafish)	ENSMUSG00000020430	16030	37,26
Tmem33	transmembrane protein 33	ENSMUSG00000037720	30897	37,26

Ggnbp1	gametogenetin binding protein 1	ENSMUSG00000048731	63162	37,26
Dpp8	dipeptidylpeptidase 8	ENSMUSG00000032393	50193	37,26
Mrap2	melanocortin 2 receptor accessory protein 2	ENSMUSG00000042761	39740	37,26
Relb	avian reticuloendotheliosis viral (v-rel) oncogene related B	ENSMUSG00000002983	23222	37,26
Tmem208	transmembrane protein 208	ENSMUSG00000014856	8866	37,19
Lamtor2	late endosomal/lysosomal adaptor, MAPK and MTOR activator 2	ENSMUSG00000028062	3256	37,19
Hmox2	heme oxygenase (decycling) 2	ENSMUSG00000004070	40382	37,19
Capn7	calpain 7	ENSMUSG00000021893	35349	37,19
Jmjd6	jumonji domain containing 6	ENSMUSG00000056962	6018	37,19
Itga6	integrin alpha 6	ENSMUSG00000027111	112801	37,19
1700040L02Rik	RIKEN cDNA 1700040L02 gene	ENSMUSG00000019945	110944	37,19
Syt9	synaptotagmin IX	ENSMUSG00000062542	177929	37,19
Cldn12	claudin 12	ENSMUSG00000046798	9944	37,12
Tdg	thymine DNA glycosylase	ENSMUSG00000034674	20972	37,12
Tacc1	transforming, acidic coiled-coil containing protein 1	ENSMUSG00000065954	46898	37,12
Cryab	crystallin, alpha B	ENSMUSG00000032060	3876	37,12
Zfp180	zinc finger protein 180	ENSMUSG00000057101	25775	37,12
E330009J07Rik	RIKEN cDNA E330009J07 gene	ENSMUSG00000037172	34761	37,12
Eefsec	eukaryotic elongation factor, selenocysteine-tRNA-specific	ENSMUSG00000033216	189206	37,12
		ENSMUSG00000033427	34769	37,12
Txndc16	thioredoxin domain containing 16	ENSMUSG00000021830	85881	37,04
		ENSMUSG00000036109	89264	37,04
Unc79	unc-79 homolog (C. elegans)	ENSMUSG00000021198	235207	37,04
Ccdc146	coiled-coil domain containing 146	ENSMUSG00000064280	131717	37,04
Per3	period circadian clock 3	ENSMUSG00000028957	41014	37,04
Ppp1ca	protein phosphatase 1, catalytic subunit, alpha isoform	ENSMUSG00000040385	3262	36,97
Rcn1	reticulocalbin 1	ENSMUSG00000005973	13029	36,97
Acp2	acid phosphatase 2, lysosomal	ENSMUSG00000002103	11214	36,97
Mrps30	mitochondrial ribosomal protein S30	ENSMUSG00000021731	7143	36,97
Pkib	protein kinase inhibitor beta, cAMP dependent, testis specific	ENSMUSG00000019876	109132	36,97
Rchy1	ring finger and CHY zinc finger domain containing 1	ENSMUSG00000029397	14165	36,97
Pacs1	phosphofurin acidic cluster sorting protein 1	ENSMUSG00000024855	139432	36,97
Mta3	metastasis associated 3	ENSMUSG00000055817	115354	36,97
Myo9b	myosin IXb	ENSMUSG00000004677	87999	36,97
Mad1l1	MAD1 mitotic arrest deficient 1-like 1	ENSMUSG00000029554	312864	36,97
Incenp	inner centromere protein	ENSMUSG00000024660	27237	36,90
Abca7	ATP-binding cassette, sub-family A (ABC1), member 7	ENSMUSG00000035722	19079	36,90
Eif2a	eukaryotic translation initiation factor 2A	ENSMUSG00000027810	31681	36,90
Cpsf3	cleavage and polyadenylation specificity factor 3	ENSMUSG00000054309	28760	36,90
Setdb1	SET domain, bifurcated 1	ENSMUSG00000015697	33678	36,90
Tcta	T cell leukemia translocation altered gene	ENSMUSG00000039461	2993	36,83

Cenpv	centromere protein V	ENSMUSG00000018509	14316	36,83
Mccc1	methylcrotonoyl-Coenzyme A carboxylase 1 (alpha)	ENSMUSG00000027709	41367	36,83
Rhot1	ras homolog gene family, member T1	ENSMUSG00000017686	58889	36,83
Klhl12	kelch-like 12	ENSMUSG00000026455	35488	36,83
Abca5	ATP-binding cassette, sub-family A (ABC1), member 5	ENSMUSG00000018800	68348	36,83
Sgms2	sphingomyelin synthase 2	ENSMUSG00000050931	25956	36,83
Snx25	sorting nexin 25	ENSMUSG00000038291	118899	36,83
Loxl2	lysyl oxidase-like 2	ENSMUSG00000034205	86663	36,83
Mrpl16	mitochondrial ribosomal protein L16	ENSMUSG00000024683	4530	36,75
Ppp1r14c	protein phosphatase 1, regulatory (inhibitor) subunit 14c	ENSMUSG00000040653	98826	36,75
Ica1	islet cell autoantigen 1	ENSMUSG00000062995	147962	36,75
Cdk5r2	cyclin-dependent kinase 5, regulatory subunit 2 (p39)	ENSMUSG00000090071	2704	36,68
Dcaf12	DDB1 and CUL4 associated factor 12	ENSMUSG00000028436	23590	36,68
Patl2	protein associated with topoisomerase II homolog 2 (yeast)	ENSMUSG00000027233	66082	36,68
Gm281	predicted gene 281	ENSMUSG00000084902	99530	36,68
		ENSMUSG00000028613	74580	36,61
Spata2	spermatogenesis associated 2	ENSMUSG00000047030	11755	36,61
Mgat4a	mannoside acetylglucosaminyltransferase 4, isoenzyme A	ENSMUSG00000026110	101677	36,61
Gcsh	glycine cleavage system protein H (aminomethyl carrier)	ENSMUSG00000034424	11728	36,54
Dap	death-associated protein	ENSMUSG00000039168	49954	36,54
Gtf2a2	general transcription factor II A, 2	ENSMUSG00000033543	10317	36,54
Adamts3	a disintegrin-like and metallopeptidase (reprolysin type) with thrombospondin type 1 motif, 3	ENSMUSG00000043635	209494	36,54
		ENSMUSG00000003068	14880	36,54
Rplp2	ribosomal protein, large P2	ENSMUSG00000025508	4216	36,54
Gga2	golgi associated, gamma adaptin ear containing, ARF binding protein 2	ENSMUSG00000030872	34501	36,54
Ints5	integrator complex subunit 5	ENSMUSG00000071652	4904	36,54
Ccdc117	coiled-coil domain containing 117	ENSMUSG00000020482	13301	36,54
		ENSMUSG00000038909	35988	36,54
Gm10563	predicted gene 10563	ENSMUSG00000073682	30996	36,54
Wars2	tryptophanyl tRNA synthetase 2 (mitochondrial)	ENSMUSG00000004233	98119	36,54
Vmo1	vitelline membrane outer layer 1 homolog (chicken)	ENSMUSG00000020830	1101	36,46
Rabl2	RAB, member of RAS oncogene family-like 2	ENSMUSG00000022621	9391	36,46
Aptx	aprataxin	ENSMUSG00000028411	20813	36,46
Mta2	metastasis-associated gene family, member 2	ENSMUSG00000071646	10429	36,46
Ccdc39	coiled-coil domain containing 39	ENSMUSG00000027676	31949	36,46
Wdr47	WD repeat domain 47	ENSMUSG00000040389	54441	36,39

4933427D14Rik	RIKEN cDNA 4933427D14 gene	ENSMUSG00000020807	53531	36,39
Atg2a	autophagy related 2A	ENSMUSG00000024773	20668	36,32
Ccser2	coiled-coil serine rich 2	ENSMUSG00000058690	93842	36,32
Sri	sorcin	ENSMUSG00000003161	23302	36,32
		ENSMUSG00000019763	30474	36,32
Sytl3	synaptotagmin-like 3	ENSMUSG00000041831	64585	36,32
Tprgl	transformation related protein 63 regulated like	ENSMUSG00000029030	3182	36,25
Twf1	twinfilin, actin-binding protein, homolog 1 (Drosophila)	ENSMUSG00000022451	11939	36,25
Clip3	CAP-GLY domain containing linker protein 3	ENSMUSG00000013921	16696	36,25
Faim	Fas apoptotic inhibitory molecule	ENSMUSG00000032463	15646	36,25
		ENSMUSG00000027351	60906	36,25
Carf	calcium response factor	ENSMUSG00000026017	52786	36,25
Rad50	RAD50 homolog (S. cerevisiae)	ENSMUSG00000020380	57801	36,25
Etfa	electron transferring flavoprotein, alpha polypeptide	ENSMUSG00000032314	57736	36,25
Wdr63	WD repeat domain 63	ENSMUSG00000043020	67605	36,25
Slc4a8	solute carrier family 4 (anion exchanger), member 8	ENSMUSG00000023032	62222	36,17
Gm10774	predicted pseudogene 10774	ENSMUSG00000074846	377	36,17
Slc38a10	solute carrier family 38, member 10	ENSMUSG00000061306	47387	36,17
Gfra1	glial cell line derived neurotrophic factor family receptor alpha 1	ENSMUSG00000025089	220306	36,10
Mlycd	malonyl-CoA decarboxylase	ENSMUSG00000074064	16225	36,10
Chid1	chitinase domain containing 1	ENSMUSG00000025512	46722	36,10
Ttc1	tetratricopeptide repeat domain 1	ENSMUSG00000041278	18497	36,10
Abl1	c-abl oncogene 1, non-receptor tyrosine kinase	ENSMUSG00000026842	115852	36,10
Bcl2l13	BCL2-like 13 (apoptosis facilitator)	ENSMUSG00000009112	56613	36,10
Fbxo9	f-box protein 9	ENSMUSG00000001366	27567	36,03
Txndc12	thioredoxin domain containing 12 (endoplasmic reticulum)	ENSMUSG00000028567	27527	36,03
Pef1	penta-EF hand domain containing 1	ENSMUSG00000028779	25578	36,03
Hexa	hexosaminidase A	ENSMUSG00000025232	25439	36,03
		ENSMUSG00000034854	8735	36,03
		ENSMUSG00000029111	39478	36,03
Ubl5	ubiquitin-like 5	ENSMUSG00000084786	4263	36,03
Dnal1	dynein, axonemal, light chain 1	ENSMUSG00000042523	33133	36,03
Mphosph9	M-phase phosphoprotein 9	ENSMUSG00000038126	77014	36,03
Bag6	BCL2-associated athanogene 6	ENSMUSG00000024392	12145	36,03
Phactr4	phosphatase and actin regulator 4	ENSMUSG00000066043	66567	36,03
Ghr	growth hormone receptor	ENSMUSG00000055737	265733	36,03
		ENSMUSG00000023984	72273	36,03
Ttc24	tetratricopeptide repeat domain 24	ENSMUSG00000051036	8895	36,03
Ddx26b	DEAD/H (Asp-Glu-Ala-Asp/His) box polypeptide 26B	ENSMUSG00000035967	52987	36,03
Cldn10	claudin 10	ENSMUSG00000022132	87619	35,96
Sbk1	SH3-binding kinase 1	ENSMUSG00000042978	22381	35,96
Ckap2l	cytoskeleton associated protein 2-like	ENSMUSG00000048327	29003	35,96
		ENSMUSG00000078572	1777	35,96
Ccdc86	coiled-coil domain containing 86	ENSMUSG00000024732	7786	35,96
		ENSMUSG00000031479	28035	35,96

Rras2	related RAS viral (r-ras) oncogene homolog 2	ENSMUSG00000055723	70997	35,96
Meaf6	MYST/Esa1-associated factor 6	ENSMUSG00000028863	28105	35,88
		ENSMUSG00000040697	30743	35,88
		ENSMUSG00000052889	345652	35,88
Alkbh8	alkB, alkylation repair homolog 8 (E. coli)	ENSMUSG00000025899	51573	35,88
Polr2j	polymerase (RNA) II (DNA directed) polypeptide J	ENSMUSG00000039771	6317	35,81
Ensa	endosulfine alpha	ENSMUSG00000038619	7123	35,81
Fbxo10	F-box protein 10	ENSMUSG00000048232	50358	35,81
Ecd	ecdysoneless homolog (Drosophila)	ENSMUSG00000021810	28263	35,81
		ENSMUSG00000044876	19124	35,81
Mgmt	O-6-methylguanine-DNA methyltransferase	ENSMUSG00000054612	233577	35,81
Itgb1bp1	integrin beta 1 binding protein 1	ENSMUSG00000062352	45413	35,81
Hexim1	hexamethylene bis-acetamide inducible 1	ENSMUSG00000048878	3401	35,81
Slc7a15	solute carrier family 7 (cationic amino acid transporter, y+ system), member 15	ENSMUSG00000020600	70584	35,81
		ENSMUSG00000096994	3262	35,74
Npnt	nephronectin	ENSMUSG00000040998	68547	35,74
Bsn	bassoon	ENSMUSG00000032589	94362	35,74
Rnf32	ring finger protein 32	ENSMUSG00000029130	29533	35,74
Atp8b5	ATPase, class I, type 8B, member 5	ENSMUSG00000028457	106675	35,74
Gpc5	glypican 5	ENSMUSG00000022112	1432965	35,74
		ENSMUSG00000097394	6347	35,67
Ccp110	centriolar coiled coil protein 110	ENSMUSG00000033904	24473	35,67
D430041D05Rik	RIKEN cDNA D430041D05 gene	ENSMUSG00000068373	267262	35,67
Gtf2e1	general transcription factor II E, polypeptide 1 (alpha subunit)	ENSMUSG00000022828	30000	35,67
Dirc2	disrupted in renal carcinoma 2 (human)	ENSMUSG00000022848	75295	35,67
Daxx	Fas death domain-associated protein	ENSMUSG00000002307	6176	35,67
Rundc1	RUN domain containing 1	ENSMUSG00000035007	10589	35,67
Adam19	a disintegrin and metallopeptidase domain 19 (meltrin beta)	ENSMUSG00000011256	91352	35,67
Mier3	mesoderm induction early response 1, family member 3	ENSMUSG00000032727	32419	35,67
Kmt2b	lysine (K)-specific methyltransferase 2B	ENSMUSG00000006307	19869	35,67
Sesn3	sestrin 3	ENSMUSG00000032009	49838	35,67
		ENSMUSG00000004018	85742	35,67
Rassf8	Ras association (RalGDS/AF-6) domain family (N-terminal) member 8	ENSMUSG00000030259	74332	35,67
Oit1	oncoprotein induced transcript 1	ENSMUSG00000021749	29816	35,67
		ENSMUSG00000020109	19747	35,59
Med16	mediator complex subunit 16	ENSMUSG00000013833	14216	35,59
Dap3	death associated protein 3	ENSMUSG00000068921	30379	35,59
Eif2b3	eukaryotic translation initiation factor 2B, subunit 3	ENSMUSG00000028683	67905	35,59

		ENSMUSG00000027834	85949	35,59
St6gal2	beta galactoside alpha 2,6 sialyltransferase 2	ENSMUSG00000024172	69200	35,59
Egflam	EGF-like, fibronectin type III and laminin G domains	ENSMUSG00000042961	192276	35,59
Gnl3	guanine nucleotide binding protein-like 3 (nucleolar)	ENSMUSG00000042354	6656	35,59
Zfp236	zinc finger protein 236	ENSMUSG00000041258	99291	35,59
Samd4	sterile alpha motif domain containing 4	ENSMUSG00000021838	222964	35,59
Lzts3	leucine zipper, putative tumor suppressor family member 3	ENSMUSG00000037703	9965	35,59
Pcdh10	protocadherin 10	ENSMUSG00000049100	56169	35,52
		ENSMUSG00000084128	6656	35,52
Klhl9	kelch-like 9	ENSMUSG00000070923	4174	35,52
Il10rb	interleukin 10 receptor, beta	ENSMUSG00000022969	19671	35,52
Itgb6	integrin beta 6	ENSMUSG00000026971	124352	35,52
Garnl3	GTPase activating RANGAP domain-like 3	ENSMUSG00000038860	145289	35,52
Sephs1	selenophosphate synthetase 1	ENSMUSG00000026662	28994	35,45
Etv5	ets variant gene 5	ENSMUSG00000013089	58411	35,45
Mrps18b	mitochondrial ribosomal protein S18B	ENSMUSG00000024436	6011	35,45
Orc2	origin recognition complex, subunit 2	ENSMUSG00000026037	42339	35,45
Cdadc1	cytidine and dCMP deaminase domain containing 1	ENSMUSG00000021982	36941	35,45
Atf1	activating transcription factor 1	ENSMUSG00000023027	33426	35,45
Spats2l	spermatogenesis associated, serine-rich 2-like	ENSMUSG00000038305	174233	35,45
Txlng	taxilin gamma	ENSMUSG00000038344	50536	35,45
		ENSMUSG00000006412	13585	35,38
Eif4e3	eukaryotic translation initiation factor 4E member 3	ENSMUSG00000093661	41637	35,38
Sema4c	sema domain, immunoglobulin domain (Ig), transmembrane domain (TM) and short cytoplasmic domain, (semaphorin) 4C	ENSMUSG00000026121	9708	35,38
Flna	filamin, alpha	ENSMUSG00000031328	26360	35,38
Pvrl1	poliovirus receptor-related 1	ENSMUSG00000032012	62879	35,38
Mrpl37	mitochondrial ribosomal protein L37	ENSMUSG00000028622	10995	35,30
Psmc1	protease (prosome, macropain) 26S subunit, ATPase 1	ENSMUSG00000021178	13179	35,30
Kctd2	potassium channel tetramerisation domain containing 2	ENSMUSG00000016940	11147	35,30
Isca1	iron-sulfur cluster assembly 1 homolog (S. cerevisiae)	ENSMUSG00000044792	14402	35,30
Cdc16	CDC16 cell division cycle 16	ENSMUSG00000038416	24263	35,30
Cep63	centrosomal protein 63	ENSMUSG00000032534	39957	35,30
		ENSMUSG00000042719	46443	35,30
Srebf1	sterol regulatory element binding transcription factor 1	ENSMUSG00000020538	23493	35,30
		ENSMUSG00000037461	46910	35,30
Padi2	peptidyl arginine deiminase, type II	ENSMUSG00000028927	46243	35,23
Hars	histidyl-tRNA synthetase	ENSMUSG00000001380	16676	35,23

H2-Ke2	H2-K region expressed gene 2	ENSMUSG00000024309	1523	35,23
Med4	mediator of RNA polymerase II transcription, subunit 4 homolog (yeast)	ENSMUSG00000022109	8497	35,23
Ick	intestinal cell kinase	ENSMUSG00000009828	62916	35,23
		ENSMUSG00000027133	1344	35,23
Pdzd11	PDZ domain containing 11	ENSMUSG00000015668	3686	35,23
Xbp1	X-box binding protein 1	ENSMUSG00000020484	5235	35,23
		ENSMUSG00000024571	5977	35,16
Agfg2	ArfGAP with FG repeats 2	ENSMUSG00000029722	34244	35,16
		ENSMUSG00000047909	12450	35,16
Ppp1r16b	protein phosphatase 1, regulatory (inhibitor) subunit 16B	ENSMUSG00000037754	100937	35,16
		ENSMUSG00000045205	60823	35,16
Narf	nuclear prelamin A recognition factor	ENSMUSG00000000056	18604	35,09
Copb2	coatomer protein complex, subunit beta 2 (beta prime)	ENSMUSG00000032458	24645	35,09
Ube2f	ubiquitin-conjugating enzyme E2F (putative)	ENSMUSG00000034343	38567	35,09
Pnmal2	PNMA-like 2	ENSMUSG00000070802	4147	35,01
Ndufa11	NADH dehydrogenase (ubiquinone) 1 alpha subcomplex 11	ENSMUSG00000002379	6487	35,01
Polr2l	polymerase (RNA) II (DNA directed) polypeptide L	ENSMUSG00000038489	3273	35,01
Dnajc18	DnaJ (Hsp40) homolog, subfamily C, member 18	ENSMUSG00000024350	32042	35,01
Tob1	transducer of ErbB-2.1	ENSMUSG00000037573	4042	35,01
Zfp277	zinc finger protein 277	ENSMUSG00000055917	130745	35,01
Brpf3	bromodomain and PHD finger containing, 3	ENSMUSG00000063952	37457	35,01
Ets2	E26 avian leukemia oncogene 2, 3' domain	ENSMUSG00000022895	18977	35,01
Tmem123	transmembrane protein 123	ENSMUSG00000050912	30293	35,01
Tbc1d8	TBC1 domain family, member 8	ENSMUSG00000003134	107249	35,01
Degs1	degenerative spermatocyte homolog 1 (Drosophila)	ENSMUSG00000038633	7033	34,94
Rfwd3	ring finger and WD repeat domain 3	ENSMUSG00000033596	29279	34,94
Cisd2	CDGSH iron sulfur domain 2	ENSMUSG00000028165	17514	34,94
Abhd13	abhydrolase domain containing 13	ENSMUSG00000040396	14449	34,94
Psmf1	proteasome (prosome, macropain) inhibitor subunit 1	ENSMUSG00000032869	28375	34,94
Cldn25	claudin 25	ENSMUSG00000022744	6342	34,94
		ENSMUSG00000021908	3339	34,94
Tma7	translational machinery associated 7 homolog (S. cerevisiae)	ENSMUSG00000091537	4394	34,94
Gatad2b	GATA zinc finger domain containing 2B	ENSMUSG00000042390	16467	34,94
Fam160a1	family with sequence similarity 160, member A1	ENSMUSG00000051000	86206	34,94
Cln3	ceroid lipofuscinosis, neuronal 3, juvenile (Batten, Spielmeyer-Vogt disease)	ENSMUSG00000030720	14611	34,94
Ddt	D-dopachrome tautomerase	ENSMUSG00000001666	2185	34,87
Hist2h3c2	histone cluster 2, H3c2	ENSMUSG00000081058	1018	34,87

Snap47	synaptosomal-associated protein, 47	ENSMUSG00000009894	44053	34,87
Vps18	vacuolar protein sorting 18 (yeast)	ENSMUSG00000034216	9714	34,87
Lman1	lectin, mannose-binding, 1	ENSMUSG00000041891	41827	34,80
Habp4	hyaluronic acid binding protein 4	ENSMUSG00000021476	24671	34,80
Ccnc	cyclin C	ENSMUSG00000028252	32222	34,80
Epha7	Eph receptor A7	ENSMUSG00000028289	154369	34,80
Arhgef10l	Rho guanine nucleotide exchange factor (GEF) 10-like	ENSMUSG00000040964	151528	34,80
Eaf2	ELL associated factor 2	ENSMUSG00000022838	82120	34,80
Ccdc92	coiled-coil domain containing 92	ENSMUSG00000037979	28007	34,72
Rpap3	RNA polymerase II associated protein 3	ENSMUSG00000022466	30718	34,72
Supt4a	suppressor of Ty 4A	ENSMUSG00000020485	6072	34,72
Snrnp40	small nuclear ribonucleoprotein 40 (U5)	ENSMUSG00000074088	29895	34,72
Tbc1d15	TBC1 domain family, member 15	ENSMUSG00000020130	53617	34,72
Dnajc21	DnaJ (Hsp40) homolog, subfamily C, member 21	ENSMUSG00000044224	23761	34,72
Plcg1	phospholipase C, gamma 1	ENSMUSG00000016933	44461	34,72
Boc	biregional cell adhesion molecule-related/down-regulated by oncogenes (Cdon) binding protein	ENSMUSG00000022687	73853	34,72
March4	membrane-associated ring finger (C3HC4) 4	ENSMUSG00000039372	109819	34,72
Thnsl1	threonine synthase-like 1 (bacterial)	ENSMUSG00000048550	9286	34,65
Nol10	nucleolar protein 10	ENSMUSG00000061458	81637	34,65
Cln6	ceroid-lipofuscinosis, neuronal 6	ENSMUSG00000032245	13222	34,65
Ttc17	tetratricopeptide repeat domain 17	ENSMUSG00000027194	105923	34,65
Shroom2	shroom family member 2	ENSMUSG00000045180	159957	34,65
Bpgm	2,3-bisphosphoglycerate mutase	ENSMUSG00000038871	29407	34,58
Ndufa4	NADH dehydrogenase (ubiquinone) 1 alpha subcomplex, 4	ENSMUSG00000029632	7074	34,58
Exosc5	exosome component 5	ENSMUSG00000061286	8879	34,58
Wrb	tryptophan rich basic protein	ENSMUSG00000023147	12446	34,58
Dnajc14	DnaJ (Hsp40) homolog, subfamily C, member 14	ENSMUSG00000025354	13771	34,58
Ubxn1	UBX domain protein 1	ENSMUSG00000071655	4105	34,58
Tmem8	transmembrane protein 8 (five membrane-spanning domains)	ENSMUSG00000024180	9939	34,58
Lig3	ligase III, DNA, ATP-dependent	ENSMUSG00000020697	23167	34,58
B230118H07Rik	RIKEN cDNA B230118H07 gene	ENSMUSG00000027165	88752	34,58
Fam60a	family with sequence similarity 60, member A	ENSMUSG00000039985	25433	34,58
Mettl25	methyltransferase like 25	ENSMUSG00000036009	78192	34,58
Mfn1	mitofusin 1	ENSMUSG00000027668	49775	34,58
Lace1	lactation elevated 1	ENSMUSG00000038302	165981	34,58
Arntl	aryl hydrocarbon receptor nuclear translocator-like	ENSMUSG00000055116	106658	34,58
Nat14	N-acetyltransferase 14	ENSMUSG00000035285	2756	34,51
Rpa2	replication protein A2	ENSMUSG00000028884	10421	34,51
Ndufs8	NADH dehydrogenase (ubiquinone) Fe-S protein 8	ENSMUSG00000059734	3848	34,51
Baiap2	brain-specific angiogenesis inhibitor 1-associated protein 2	ENSMUSG00000025372	64020	34,51

Bcar3	breast cancer anti-estrogen resistance 3	ENSMUSG00000028121	110401	34,51
B3gntl1	UDP-GlcNAc:betaGal beta-1,3-N-acetylglucosaminyltransferase-like 1	ENSMUSG00000046605	56957	34,51
2310011J03Rik	RIKEN cDNA 2310011J03 gene	ENSMUSG00000020133	2284	34,43
		ENSMUSG00000059291	25164	34,43
Drap1	Dr1 associated protein 1 (negative cofactor 2 alpha)	ENSMUSG00000024914	2175	34,43
Mdn1	midasin homolog (yeast)	ENSMUSG00000058006	118099	34,43
Mthfd1l	methylenetetrahydrofolate dehydrogenase (NADP+ dependent) 1-like	ENSMUSG00000040675	193964	34,43
Sec24d	Sec24 related gene family, member D (S. cerevisiae)	ENSMUSG00000039234	98140	34,43
Klf2	Kruppel-like factor 2 (lung)	ENSMUSG00000055148	2624	34,43
Glt8d1	glycosyltransferase 8 domain containing 1	ENSMUSG00000021916	11049	34,36
Sgtb	small glutamine-rich tetratricopeptide repeat (TPR)-containing, beta	ENSMUSG00000042743	31995	34,36
Zswim8	zinc finger SWIM-type containing 8	ENSMUSG00000021819	16067	34,36
Urm1	ubiquitin related modifier 1 homolog (S. cerevisiae)	ENSMUSG00000069020	24928	34,36
Irs1	insulin receptor substrate 1	ENSMUSG00000055980	58316	34,36
Atg5	autophagy related 5	ENSMUSG00000038160	95934	34,36
Abcb10	ATP-binding cassette, sub-family B (MDR/TAP), member 10	ENSMUSG00000031974	30664	34,36
Plcxd3	phosphatidylinositol-specific phospholipase C, X domain containing 3	ENSMUSG00000049148	200063	34,36
1700109H08Rik	RIKEN cDNA 1700109H08 gene	ENSMUSG00000008307	12791	34,36
Brap	BRCA1 associated protein	ENSMUSG00000029458	26686	34,29
Cks2	CDC28 protein kinase regulatory subunit 2	ENSMUSG00000062248	5429	34,29
		ENSMUSG00000031198	14061	34,29
Wnt5a	wingless-related MMTV integration site 5A	ENSMUSG00000021994	22698	34,29
Lhfpl4	lipoma HMGIC fusion partner-like protein 4	ENSMUSG00000042873	27295	34,22
Zfp933	zinc finger protein 933	ENSMUSG00000059423	25381	34,22
Zfp740	zinc finger protein 740	ENSMUSG00000046897	11959	34,22
Tmem229b	transmembrane protein 229B	ENSMUSG00000046157	45833	34,22
Adck1	aarF domain containing kinase 1	ENSMUSG00000021044	101166	34,22
Ankrd13c	ankyrin repeat domain 13c	ENSMUSG00000039988	59420	34,22
		ENSMUSG00000039684	2906	34,14
Khsrp	KH-type splicing regulatory protein	ENSMUSG00000007670	10450	34,14
Rnpepl1	arginyl aminopeptidase (aminopeptidase B)-like 1	ENSMUSG00000026269	13602	34,14
Vars	valyl-tRNA synthetase	ENSMUSG00000007029	15336	34,14
Dohh	deoxyhypusine hydroxylase/monooxygenase	ENSMUSG00000078440	7133	34,14
Mapk8ip1	mitogen-activated protein kinase 8 interacting protein 1	ENSMUSG00000027223	17588	34,14
Eif3k	eukaryotic translation initiation factor 3, subunit K	ENSMUSG00000053565	10442	34,14

Msh6	mutS homolog 6 (E. coli)	ENSMUSG00000005370	15834	34,14
Ints8	integrator complex subunit 8	ENSMUSG00000040738	55101	34,14
Cacna1h	calcium channel, voltage-dependent, T type, alpha 1H subunit	ENSMUSG00000024112	59499	34,07
Plrg1	pleiotropic regulator 1, PRL1 homolog (Arabidopsis)	ENSMUSG00000027998	16834	34,07
Rab33b	RAB33B, member of RAS oncogene family	ENSMUSG00000027739	11940	34,07
Rab11fip2	RAB11 family interacting protein 2 (class I)	ENSMUSG00000040022	40771	34,07
Fbp2	fructose bisphosphatase 2	ENSMUSG00000021456	21517	34,07
Ltbp3	latent transforming growth factor beta binding protein 3	ENSMUSG00000024940	17629	34,07
Tgfa	transforming growth factor alpha	ENSMUSG00000029999	80199	34,07
Atp6v0e2	ATPase, H+ transporting, lysosomal V0 subunit E2	ENSMUSG00000039347	4230	34,00
Sox12	SRY-box containing gene 12	ENSMUSG00000051817	4453	34,00
B9d2	B9 protein domain 2	ENSMUSG00000063439	5401	34,00
Tmem70	transmembrane protein 70	ENSMUSG00000025940	13069	34,00
		ENSMUSG00000063328	567	34,00
3632451O06Rik	RIKEN cDNA 3632451O06 gene	ENSMUSG00000036242	101824	34,00
Nvl	nuclear VCP-like	ENSMUSG00000026516	50735	34,00
Poc1b	POC1 centriolar protein homolog B (Chlamydomonas)	ENSMUSG00000019952	91039	34,00
Pdf	peptide deformylase (mitochondrial)	ENSMUSG00000078931	3917	33,93
Noc4l	nucleolar complex associated 4 homolog (S. cerevisiae)	ENSMUSG00000033294	5000	33,93
		ENSMUSG00000021903	33209	33,93
Senp8	SUMO/sentrin specific peptidase 8	ENSMUSG00000051705	16391	33,93
Nfyb	nuclear transcription factor-Y beta	ENSMUSG00000020248	15444	33,93
Caml	calcium modulating ligand	ENSMUSG00000021501	9407	33,93
Hic2	hypermethylated in cancer 2	ENSMUSG00000050240	29859	33,93
Tmem116	transmembrane protein 116	ENSMUSG00000029452	72291	33,93
Gtf2ird2	GTF2I repeat domain containing 2	ENSMUSG00000015942	40337	33,93
Xpo4	exportin 4	ENSMUSG00000021952	84429	33,93
Rpgr	retinitis pigmentosa GTPase regulator	ENSMUSG00000031174	143300	33,93
Adhfe1	alcohol dehydrogenase, iron containing, 1	ENSMUSG00000025911	30023	33,85
Atp13a2	ATPase type 13A2	ENSMUSG00000036622	20458	33,85
Dtymk	deoxythymidylate kinase	ENSMUSG00000026281	9359	33,85
Sspn	sarcospan	ENSMUSG00000030255	33583	33,85
Dsel	dermatan sulfate epimerase-like	ENSMUSG00000038702	6208	33,85
5031414D18Rik	RIKEN cDNA 5031414D18 gene	ENSMUSG00000034959	36506	33,78
Ccdc107	coiled-coil domain containing 107	ENSMUSG00000028461	3022	33,78
		ENSMUSG00000034620	19258	33,78
Spata24	spermatogenesis associated 24	ENSMUSG00000024352	5497	33,78
Pi4k2b	phosphatidylinositol 4-kinase type 2 beta	ENSMUSG00000029186	27758	33,78
Ctnna3	catenin (cadherin associated protein), alpha 3	ENSMUSG00000060843	1573570	33,78
Dgka	diacylglycerol kinase, alpha	ENSMUSG00000025357	23916	33,78
Idh3a	isocitrate dehydrogenase 3 (NAD+) alpha	ENSMUSG00000032279	18151	33,71

Cnga1	cyclic nucleotide gated channel alpha 1	ENSMUSG00000067220	39057	33,71
Tspyl5	testis-specific protein, Y-encoded-like 5	ENSMUSG00000038984	4008	33,71
Zfp219	zinc finger protein 219	ENSMUSG00000049295	14657	33,71
Rnaset2b	ribonuclease T2B	ENSMUSG00000094724	32440	33,71
Bag1	BCL2-associated athanogene 1	ENSMUSG00000028416	11884	33,71
Aldh3a2	aldehyde dehydrogenase family 3, subfamily A2	ENSMUSG00000010025	44048	33,71
Itpk1	inositol 1,3,4-triphosphate 5/6 kinase	ENSMUSG00000057963	136348	33,71
Tatdn1	TatD DNase domain containing 1	ENSMUSG00000050891	43578	33,71
Mcoln1	mucolipin 1	ENSMUSG00000004567	14776	33,64
Cnot7	CCR4-NOT transcription complex, subunit 7	ENSMUSG00000031601	23308	33,64
Znfx1	zinc finger, NFX1-type containing 1	ENSMUSG00000039501	27223	33,64
Dcpp3	demilune cell and parotid protein 3	ENSMUSG00000057417	1984	33,56
Sh2b2	SH2B adaptor protein 2	ENSMUSG00000005057	26755	33,56
		ENSMUSG00000028099	16139	33,56
Galnt10	UDP-N-acetyl-alpha-D-galactosamine:polypeptide N-acetylgalactosaminyltransferase 10	ENSMUSG00000020520	142073	33,56
Ufl1	UFM1 specific ligase 1	ENSMUSG00000040359	33222	33,56
Elk3	ELK3, member of ETS oncogene family	ENSMUSG00000008398	63722	33,56
Rexo4	REX4, RNA exonuclease 4 homolog (S. cerevisiae)	ENSMUSG00000052406	10824	33,56
Rpa1	replication protein A1	ENSMUSG00000000751	50159	33,49
Snrnp35	small nuclear ribonucleoprotein 35 (U11/U12)	ENSMUSG00000029402	7991	33,49
Ppp1r3e	protein phosphatase 1, regulatory (inhibitor) subunit 3E	ENSMUSG00000072494	4882	33,49
Zfp598	zinc finger protein 598	ENSMUSG00000041130	12264	33,49
Zbed3	zinc finger, BED domain containing 3	ENSMUSG00000041995	12605	33,49
Ppp1r21	protein phosphatase 1, regulatory subunit 21	ENSMUSG00000034709	58242	33,49
Osbp2	oxysterol binding protein 2	ENSMUSG00000020435	160173	33,49
Yipf5	Yip1 domain family, member 5	ENSMUSG00000024487	14535	33,49
Ahsa2	AHA1, activator of heat shock protein ATPase 2	ENSMUSG00000020288	10149	33,49
Ift140	intraflagellar transport 140	ENSMUSG00000024169	83405	33,49
Trim3	tripartite motif-containing 3	ENSMUSG00000036989	29109	33,49
Msn	moesin	ENSMUSG00000031207	72511	33,42
Zfp768	zinc finger protein 768	ENSMUSG00000047371	2518	33,42
Aurkaip1	aurora kinase A interacting protein 1	ENSMUSG00000065990	1859	33,42
Mrpl45	mitochondrial ribosomal protein L45	ENSMUSG00000018882	14205	33,42
Ptpla	protein tyrosine phosphatase-like (proline instead of catalytic arginine), member a	ENSMUSG00000063275	80573	33,42
Nusap1	nucleolar and spindle associated protein 1	ENSMUSG00000027306	32947	33,42
Spsb1	splA/ryanodine receptor domain and SOCS box containing 1	ENSMUSG00000039911	58761	33,42

Ociad2	OCIA domain containing 2	ENSMUSG00000029153	16749	33,42
4430402I18Rik	RIKEN cDNA 4430402I18 gene	ENSMUSG00000064202	66533	33,42
1110057K04Rik	RIKEN cDNA 1110057K04 gene	ENSMUSG00000037669	77652	33,42
Tjp3	tight junction protein 3	ENSMUSG00000034917	18061	33,42
Ptp4a3	protein tyrosine phosphatase 4a3	ENSMUSG00000059895	35622	33,35
Sh3bp5l	SH3 binding domain protein 5 like	ENSMUSG00000013646	17005	33,35
Hcfc1	host cell factor C1	ENSMUSG00000031386	23566	33,35
Ctsz	cathepsin Z	ENSMUSG00000016256	11547	33,27
Cct2	chaperonin containing Tcp1, subunit 2 (beta)	ENSMUSG00000034024	12817	33,27
Aen	apoptosis enhancing nuclease	ENSMUSG00000030609	15356	33,27
Cry1	cryptochrome 1 (photolyase-like)	ENSMUSG00000020038	53353	33,27
Zfp800	zinc finger protein 800	ENSMUSG00000039841	158075	33,27
Zmym3	zinc finger, MYM-type 3	ENSMUSG00000031310	16466	33,27
Rab27b	RAB27b, member RAS oncogene family	ENSMUSG00000024511	162475	33,27
Foxk1	forkhead box K1	ENSMUSG00000056493	60518	33,20
Slc25a27	solute carrier family 25, member 27	ENSMUSG00000023912	25116	33,20
0610011F06Rik	RIKEN cDNA 0610011F06 gene	ENSMUSG00000025731	10544	33,13
Coq4	coenzyme Q4 homolog (yeast)	ENSMUSG00000026798	10443	33,13
Dnajc10	DnaJ (Hsp40) homolog, subfamily C, member 10	ENSMUSG00000027006	38578	33,13
C1d	C1D nuclear receptor co-repressor	ENSMUSG00000000581	11598	33,13
Ing5	inhibitor of growth family, member 5	ENSMUSG00000026283	18137	33,13
Asah1	N-acylsphingosine amidohydrolase 1	ENSMUSG00000031591	34577	33,13
Xpnpep3	X-prolyl aminopeptidase (aminopeptidase P) 3, putative	ENSMUSG00000022401	56670	33,13
Rsu1	Ras suppressor protein 1	ENSMUSG00000026727	194465	33,13
Zswim4	zinc finger SWIM-type containing 4	ENSMUSG00000035671	26101	33,13
Ccdc85a	coiled-coil domain containing 85A	ENSMUSG00000032878	198640	33,13
Ccbe1	collagen and calcium binding EGF domains 1	ENSMUSG00000046318	257438	33,13
Dhrs7b	dehydrogenase/reductase (SDR family) member 7B	ENSMUSG00000042569	29565	33,06
Gpc3	glypican 3	ENSMUSG00000055653	341525	33,06
Zfp839	zinc finger protein 839	ENSMUSG00000021271	19720	33,06
Sppl2b	signal peptide peptidase like 2B	ENSMUSG00000035206	13431	33,06
Cbr1	carbonyl reductase 1	ENSMUSG00000051483	2669	33,06
Ints1	integrator complex subunit 1	ENSMUSG00000029547	24397	33,06
Lamc1	laminin, gamma 1	ENSMUSG00000026478	113865	33,06
Odf2l	outer dense fiber of sperm tails 2-like	ENSMUSG00000028256	35327	33,06
Fam19a1	family with sequence similarity 19, member A1	ENSMUSG00000059187	544045	33,06
Sar1a	SAR1 gene homolog A (S. cerevisiae)	ENSMUSG00000020088	12986	32,98
Dzip1l	DAZ interacting protein 1-like	ENSMUSG00000037784	39761	32,98
Plod3	procollagen-lysine, 2-oxoglutarate 5-dioxygenase 3	ENSMUSG00000004846	9630	32,98
Ppp1r11	protein phosphatase 1, regulatory (inhibitor) subunit 11	ENSMUSG00000036398	3386	32,98
Ttll12	tubulin tyrosine ligase-like family, member 12	ENSMUSG00000016757	20039	32,98
		ENSMUSG00000044468	37697	32,98

4933411K20Rik	RIKEN cDNA 4933411K20 gene	ENSMUSG00000031631	43820	32,98
Tbc1d2b	TBC1 domain family, member 2B	ENSMUSG00000037410	68758	32,98
Klhl2	kelch-like 2, Mayven	ENSMUSG00000031605	109956	32,98
Vrk2	vaccinia related kinase 2	ENSMUSG00000064090	122678	32,98
Slc35d2	solute carrier family 35, member D2	ENSMUSG00000033114	33021	32,98
Tomm22	translocase of outer mitochondrial membrane 22 homolog (yeast)	ENSMUSG00000022427	2002	32,91
		ENSMUSG00000020519	8581	32,91
St3gal2	ST3 beta-galactoside alpha-2,3-sialyltransferase 2	ENSMUSG00000031749	52559	32,91
Trnt1	tRNA nucleotidyl transferase, CCA-adding, 1	ENSMUSG00000013736	13355	32,91
Mknk2	MAP kinase-interacting serine/threonine kinase 2	ENSMUSG00000020190	6645	32,91
Tram1	translocating chain-associating membrane protein 1	ENSMUSG00000025935	25163	32,91
Fam162a	family with sequence similarity 162, member A	ENSMUSG00000003955	27672	32,91
Rps27l	ribosomal protein S27-like	ENSMUSG00000036781	3431	32,91
Rab3gap2	RAB3 GTPase activating protein subunit 2	ENSMUSG00000039318	82576	32,91
Tmtc4	transmembrane and tetratricopeptide repeat containing 4	ENSMUSG00000041594	65065	32,91
Atad5	ATPase family, AAA domain containing 5	ENSMUSG00000017550	46395	32,91
Dusp14	dual specificity phosphatase 14	ENSMUSG00000018648	21221	32,84
Ptpn6	protein tyrosine phosphatase, non-receptor type 6	ENSMUSG00000004266	18008	32,84
Smim13	small integral membrane protein 13	ENSMUSG00000091264	26715	32,84
Ccsap	centriole, cilia and spindle associated protein	ENSMUSG00000031971	19379	32,84
Nat10	N-acetyltransferase 10	ENSMUSG00000027185	40015	32,84
Sec61a2	Sec61, alpha subunit 2 (S. cerevisiae)	ENSMUSG00000025816	24446	32,84
		ENSMUSG00000095562	9623	32,84
Sh3kbp1	SH3-domain kinase binding protein 1	ENSMUSG00000040990	350798	32,84
Lrrtm4	leucine rich repeat transmembrane neuronal 4	ENSMUSG00000052581	791267	32,84
Slc12a8	solute carrier family 12 (potassium/chloride transporters), member 8	ENSMUSG00000035506	146808	32,84
Mesdc1	mesoderm development candidate 1	ENSMUSG00000070462	3847	32,77
Zbtb7b	zinc finger and BTB domain containing 7B	ENSMUSG00000028042	17133	32,77
Dpagt1	dolichyl-phosphate (UDP-N-acetylglucosamine) acetylglucosaminephosphotransferase 1 (GlcNAc-1-P transferase)	ENSMUSG00000032123	6756	32,77
Atn1	atrophin 1	ENSMUSG00000004263	17336	32,77
Arpc4	actin related protein 2/3 complex, subunit 4	ENSMUSG00000079426	12336	32,77
Zfand2b	zinc finger, AN1 type domain 2B	ENSMUSG00000026197	2984	32,77

		ENSMUSG00000022175	7334	32,77
		ENSMUSG00000043702	9926	32,77
Esco1	establishment of cohesion 1 homolog 1 (S. cerevisiae)	ENSMUSG00000024293	43751	32,77
Gpatch2l	G patch domain containing 2 like	ENSMUSG00000021254	49525	32,77
Asb14	ankyrin repeat and SOCS box-containing 14	ENSMUSG00000021898	20702	32,77
Rab5c	RAB5C, member RAS oncogene family	ENSMUSG00000019173	23207	32,69
Hyal3	hyaluronoglucosaminidase 3	ENSMUSG00000036091	6542	32,69
Kcnh4	potassium voltage-gated channel, subfamily H (eag-related), member 4	ENSMUSG00000035355	19567	32,69
Tiam2	T cell lymphoma invasion and metastasis 2	ENSMUSG00000023800	192825	32,69
Foxj3	forkhead box J3	ENSMUSG00000032998	92116	32,69
Apex2	apurinic/apyrimidinic endonuclease 2	ENSMUSG00000025269	70350	32,69
Epha1	Eph receptor A1	ENSMUSG00000029859	14782	32,69
Ciao1	cytosolic iron-sulfur protein assembly 1	ENSMUSG00000003662	6879	32,62
Zfp281	zinc finger protein 281	ENSMUSG00000041483	5153	32,62
Trmt1	tRNA methyltransferase 1	ENSMUSG00000001909	13502	32,62
Sphk2	sphingosine kinase 2	ENSMUSG00000057342	8536	32,62
Taz	tafazzin	ENSMUSG00000009995	8240	32,62
Metap1d	methionyl aminopeptidase type 1D (mitochondrial)	ENSMUSG00000041921	71919	32,62
AI846148	expressed sequence AI846148	ENSMUSG00000024970	26563	32,62
1700094D03Rik	RIKEN cDNA 1700094D03 gene	ENSMUSG00000078667	5552	32,62
Pqbp1	polyglutamine binding protein 1	ENSMUSG00000031157	4751	32,55
Zfyve20	zinc finger, FYVE domain containing 20	ENSMUSG00000014550	28214	32,55
Zfp259	zinc finger protein 259	ENSMUSG00000032078	9580	32,55
		ENSMUSG00000069014	1495	32,55
Fundc1	FUN14 domain containing 1	ENSMUSG00000025040	15762	32,55
Ccdc149	coiled-coil domain containing 149	ENSMUSG00000045790	96893	32,55
		ENSMUSG00000009621	164930	32,55
Nsmce1	non-SMC element 1 homolog (S. cerevisiae)	ENSMUSG00000030750	23957	32,55
Kirrel	kin of IRRE like (Drosophila)	ENSMUSG00000041734	96155	32,55
Sirt1	sirtuin 1	ENSMUSG00000020063	62700	32,55
Srrm3	serine/arginine repetitive matrix 3	ENSMUSG00000039860	67883	32,55
Lsm4	LSM4 homolog, U6 small nuclear RNA associated (S. cerevisiae)	ENSMUSG00000031848	5505	32,48
Dnajc19	DnaJ (Hsp40) homolog, subfamily C, member 19	ENSMUSG00000027679	25302	32,48
BC005624	cDNA sequence BC005624	ENSMUSG00000026851	9369	32,48
Nae1	NEDD8 activating enzyme E1 subunit 1	ENSMUSG00000031878	23610	32,48
2410002F23Rik	RIKEN cDNA 2410002F23 gene	ENSMUSG00000045411	15999	32,48
		ENSMUSG00000035578	40834	32,48
Bud31	BUD31 homolog (yeast)	ENSMUSG00000038722	7717	32,40
Camta2	calmodulin binding transcription activator 2	ENSMUSG00000040712	18643	32,40
Rrp1b	ribosomal RNA processing 1 homolog B (S. cerevisiae)	ENSMUSG00000058392	26766	32,40

Dusp11	dual specificity phosphatase 11 (RNA/RNP complex 1-interacting)	ENSMUSG00000030002	19400	32,40
Hiatl1	hippocampus abundant transcript-like 1	ENSMUSG00000038212	48320	32,40
2510002D24Rik	RIKEN cDNA 2510002D24 gene	ENSMUSG00000071632	3581	32,40
Utp14a	UTP14, U3 small nucleolar ribonucleoprotein, homolog A (yeast)	ENSMUSG00000063785	25592	32,40
		ENSMUSG00000024077	87284	32,40
Ranbp3	RAN binding protein 3	ENSMUSG00000002372	38545	32,40
Nup214	nucleoporin 214	ENSMUSG00000001855	79540	32,40
Zcchc16	zinc finger, CCHC domain containing 16	ENSMUSG00000071679	433505	32,40
Tead2	TEA domain family member 2	ENSMUSG00000030796	17883	32,33
Vat1	vesicle amine transport protein 1 homolog (T californica)	ENSMUSG00000034993	7486	32,33
		ENSMUSG00000017299	22625	32,33
Gart	phosphoribosylglycinamide formyltransferase	ENSMUSG00000022962	25767	32,33
Parl	presenilin associated, rhomboid-like	ENSMUSG00000033918	22568	32,33
Kntc1	kinetochore associated 1	ENSMUSG00000029414	71868	32,33
Usp20	ubiquitin specific peptidase 20	ENSMUSG00000026854	41308	32,33
Eny2	enhancer of yellow 2 homolog (Drosophila)	ENSMUSG00000022338	9575	32,33
Snx19	sorting nexin 19	ENSMUSG00000031993	39398	32,33
Ascc1	activating signal cointegrator 1 complex subunit 1	ENSMUSG00000044475	97186	32,33
		ENSMUSG00000021716	46791	32,33
Usp28	ubiquitin specific peptidase 28	ENSMUSG00000032267	57133	32,33
Dpy19l3	dpy-19-like 3 (C. elegans)	ENSMUSG00000043671	69290	32,33
Mis12	MIS12 homolog (yeast)	ENSMUSG00000040599	7761	32,26
Fbf1	Fas (TNFRSF6) binding factor 1	ENSMUSG00000020776	25882	32,26
Crnkl1	Crn, crooked neck-like 1 (Drosophila)	ENSMUSG00000001767	17536	32,26
Ubfd1	ubiquitin family domain containing 1	ENSMUSG00000030870	15020	32,26
Ctdnep1	CTD nuclear envelope phosphatase 1	ENSMUSG00000018559	9446	32,26
Nek6	NIMA (never in mitosis gene a)-related expressed kinase 6	ENSMUSG00000026749	76399	32,26
Gdpd1	glycerophosphodiester phosphodiesterase domain containing 1	ENSMUSG00000061666	40196	32,26
Slc35f5	solute carrier family 35, member F5	ENSMUSG00000026342	34669	32,26
Dzank1	double zinc ribbon and ankyrin repeat domains 1	ENSMUSG00000037259	56858	32,26
Usp53	ubiquitin specific peptidase 53	ENSMUSG00000039701	50847	32,26
Kctd8	potassium channel tetramerisation domain containing 8	ENSMUSG00000037653	232425	32,26
Cped1	cadherin-like and PC-esterase domain containing 1	ENSMUSG00000062980	270489	32,19
Psme3	proteaseome (prosome, macropain) activator subunit 3 (PA28 gamma, Ki)	ENSMUSG00000078652	7325	32,19

Eapp	E2F-associated phosphoprotein	ENSMUSG00000054302	25558	32,19
Tmem19	transmembrane protein 19	ENSMUSG00000069520	21524	32,19
		ENSMUSG00000054720	93631	32,19
Zfp507	zinc finger protein 507	ENSMUSG00000044452	30644	32,19
Ropn1l	ropporin 1-like	ENSMUSG00000022236	12480	32,19
Map2k7	mitogen-activated protein kinase kinase 7	ENSMUSG00000002948	12684	32,19
1110002L01Rik	RIKEN cDNA 1110002L01 gene	ENSMUSG00000071456	23134	32,19
Tssc1	tumor suppressing subtransferable candidate 1	ENSMUSG00000036613	115664	32,19
Cic	capicua homolog (Drosophila)	ENSMUSG00000005442	26456	32,19
Prcc	papillary renal cell carcinoma (translocation-associated)	ENSMUSG00000004895	26709	32,19
Gm7334	predicted gene 7334	ENSMUSG00000044645	1356	32,19
G2e3	G2/M-phase specific E3 ubiquitin ligase	ENSMUSG00000035293	28926	32,19
Zfp282	zinc finger protein 282	ENSMUSG00000025821	31282	32,19
Wasl	Wiskott-Aldrich syndrome-like (human)	ENSMUSG00000029684	51205	32,19
Tgif1	TGFB-induced factor homeobox 1	ENSMUSG00000047407	9342	32,19
Tmbim1	transmembrane BAX inhibitor motif containing 1	ENSMUSG00000006301	17376	32,19
Slc39a2	solute carrier family 39 (zinc transporter), member 2	ENSMUSG00000072572	3857	32,19
Ccdc11	coiled-coil domain containing 11	ENSMUSG00000035394	76897	32,11
Sec14l4	SEC14-like 4 (S. cerevisiae)	ENSMUSG00000019368	16563	32,11
Cdkn1c	cyclin-dependent kinase inhibitor 1C (P57)	ENSMUSG00000037664	2701	32,11
2610002M06Rik	RIKEN cDNA 2610002M06 gene	ENSMUSG00000031242	33584	32,11
Rars2	arginyl-tRNA synthetase 2, mitochondrial	ENSMUSG00000028292	45211	32,11
Dlgap2	discs, large (Drosophila) homolog-associated protein 2	ENSMUSG00000047495	751816	32,11
Tarsl2	threonyl-tRNA synthetase-like 2	ENSMUSG00000030515	47194	32,04
Slc4a2	solute carrier family 4 (anion exchanger), member 2	ENSMUSG00000028962	17114	32,04
Shq1	SHQ1 homolog (S. cerevisiae)	ENSMUSG00000035378	98077	32,04
Erbb2	v-erb-b2 erythroblastic leukemia viral oncogene homolog 2, neuro/glioblastoma derived oncogene homolog (avian)	ENSMUSG00000062312	25247	32,04
Utp20	UTP20, small subunit (SSU) processome component, homolog (yeast)	ENSMUSG00000004356	80208	32,04
Tcf7	transcription factor 7, T cell specific	ENSMUSG00000000782	30644	32,04
Rhot2	ras homolog gene family, member T2	ENSMUSG00000025733	6395	31,97
Cib1	calcium and integrin binding 1 (calmyrin)	ENSMUSG00000030538	5658	31,97
Erc2	ELKS/RAB6-interacting/CAST family member 2	ENSMUSG00000040640	856096	31,97
Fubp3	far upstream element (FUSE) binding protein 3	ENSMUSG00000026843	44876	31,97
Lmcd1	LIM and cysteine-rich domains 1	ENSMUSG00000057604	56668	31,97
Ankrd22	ankyrin repeat domain 22	ENSMUSG00000024774	43505	31,97

Hddc2	HD domain containing 2	ENSMUSG00000000295	14823	31,90
Trappc1	trafficking protein particle complex 1	ENSMUSG00000049299	1814	31,90
		ENSMUSG00000036513	8812	31,90
Usp5	ubiquitin specific peptidase 5 (isopeptidase T)	ENSMUSG00000038429	14466	31,90
Fam76b	family with sequence similarity 76, member B	ENSMUSG00000037808	18826	31,90
Wbscr22	Williams Beuren syndrome chromosome region 22	ENSMUSG00000005378	12003	31,90
Cep135	centrosomal protein 135	ENSMUSG00000036403	57769	31,90
Csrp2bp	cysteine and glycine-rich protein 2 binding protein	ENSMUSG00000027425	38694	31,90
Usp54	ubiquitin specific peptidase 54	ENSMUSG00000034235	69443	31,90
Slc17a9	solute carrier family 17, member 9	ENSMUSG00000023393	17018	31,90
Cbx2	chromobox 2	ENSMUSG00000025577	8309	31,82
Minpp1	multiple inositol polyphosphate histidine phosphatase 1	ENSMUSG00000024896	29602	31,82
Phf6	PHD finger protein 6	ENSMUSG00000025626	44678	31,82
Rab30	RAB30, member RAS oncogene family	ENSMUSG00000030643	95515	31,82
Exoc2	exocyst complex component 2	ENSMUSG00000021357	160129	31,82
Mrpl34	mitochondrial ribosomal protein L34	ENSMUSG00000034880	827	31,75
		ENSMUSG00000061360	6593	31,75
Glb1l2	galactosidase, beta 1-like 2	ENSMUSG00000036395	43425	31,75
Tdp2	tyrosyl-DNA phosphodiesterase 2	ENSMUSG00000035958	10475	31,75
Med7	mediator complex subunit 7	ENSMUSG00000020397	5797	31,75
Rnf146	ring finger protein 146	ENSMUSG00000038876	18265	31,75
Tfe3	transcription factor E3	ENSMUSG00000000134	12643	31,75
Cacnb3	calcium channel, voltage-dependent, beta 3 subunit	ENSMUSG00000003352	12311	31,68
Stoml1	stomatin-like 1	ENSMUSG00000032333	9360	31,68
Gps1	G protein pathway suppressor 1	ENSMUSG00000025156	4831	31,68
Lrpap1	low density lipoprotein receptor-related protein associated protein 1	ENSMUSG00000029103	14277	31,68
Hmbs	hydroxymethylbilane synthase	ENSMUSG00000032126	7879	31,68
Grasp	GRP1 (general receptor for phosphoinositides 1)-associated scaffold protein	ENSMUSG00000000531	8549	31,68
Ddx52	DEAD (Asp-Glu-Ala-Asp) box polypeptide 52	ENSMUSG00000020677	21027	31,68
Chordc1	cysteine and histidine-rich domain (CHORD)-containing, zinc-binding protein 1	ENSMUSG00000001774	21734	31,68
Zcchc8	zinc finger, CCHC domain containing 8	ENSMUSG00000029427	22743	31,68
		ENSMUSG00000056383	19489	31,68
		ENSMUSG00000093574	89880	31,68
Itln1	intelectin 1 (galactofuranose binding)	ENSMUSG00000038209	17173	31,68
Aatf	apoptosis antagonizing transcription factor	ENSMUSG00000018697	90668	31,68
Cend1	cell cycle exit and neuronal differentiation 1	ENSMUSG00000060240	3046	31,61
		ENSMUSG00000024666	6885	31,61

Hadhb	hydroxyacyl-Coenzyme A dehydrogenase/3-ketoacyl-Coenzyme A thiolase/enoyl-Coenzyme A hydratase (trifunctional protein), beta subunit	ENSMUSG00000059447	29346	31,61
Tsnax	translin-associated factor X	ENSMUSG00000056820	21196	31,61
Oxnad1	oxidoreductase NAD-binding domain containing 1	ENSMUSG00000021906	17829	31,61
1110012L19Rik	RIKEN cDNA 1110012L19 gene	ENSMUSG00000045237	3541	31,61
Srp54b	signal recognition particle 54B	ENSMUSG00000079108	34110	31,61
		ENSMUSG00000021891	16711	31,61
Zkscan17	zinc finger with KRAB and SCAN domains 17	ENSMUSG00000020472	41232	31,61
Pla2g4e	phospholipase A2, group IVE	ENSMUSG00000050211	78924	31,53
Rnf126	ring finger protein 126	ENSMUSG00000035890	8438	31,53
Paf1	Paf1, RNA polymerase II associated factor, homolog (S. cerevisiae)	ENSMUSG00000003437	6438	31,53
Krtcap2	keratinocyte associated protein 2	ENSMUSG00000042747	3757	31,53
Wdr31	WD repeat domain 31	ENSMUSG00000028391	22244	31,53
Unc119b	unc-119 homolog B (C. elegans)	ENSMUSG00000046562	12426	31,53
Vamp8	vesicle-associated membrane protein 8	ENSMUSG00000050732	5481	31,53
Marveld2	MARVEL (membrane-associating) domain containing 2	ENSMUSG00000021636	21015	31,53
Zfp942	zinc finger protein 942	ENSMUSG00000071267	35505	31,53
Triobp	TRIO and F-actin binding protein	ENSMUSG00000033088	58144	31,53
Gpr64	G protein-coupled receptor 64	ENSMUSG00000031298	107381	31,53
St7	suppression of tumorigenicity 7	ENSMUSG00000029534	250093	31,53
Armc4	armadillo repeat containing 4	ENSMUSG00000061802	209669	31,53
Tmem212	transmembrane protein 212	ENSMUSG00000043164	30303	31,53
Sepw1	selenoprotein W, muscle 1	ENSMUSG00000041571	5195	31,46
Cdk5r1	cyclin-dependent kinase 5, regulatory subunit 1 (p35)	ENSMUSG00000048895	4134	31,46
Sv2b	synaptic vesicle glycoprotein 2 b	ENSMUSG00000053025	194366	31,46
Trafd1	TRAF type zinc finger domain containing 1	ENSMUSG00000042726	13908	31,46
Clk4	CDC like kinase 4	ENSMUSG00000020385	20037	31,46
Ctsc	cathepsin C	ENSMUSG00000030560	32804	31,46
Pdss1	prenyl (solanesyl) diphosphate synthase, subunit 1	ENSMUSG00000026784	44745	31,46
Pllp	plasma membrane proteolipid	ENSMUSG00000031775	21348	31,39
Vhl	von Hippel-Lindau tumor suppressor	ENSMUSG00000033933	7675	31,39
Itfg3	integrin alpha FG-GAP repeat containing 3	ENSMUSG00000024187	32402	31,39
Slc2a13	solute carrier family 2 (facilitated glucose transporter), member 13	ENSMUSG00000036298	305566	31,39
Maff	v-maf musculoaponeurotic fibrosarcoma oncogene family, protein F (avian)	ENSMUSG00000042622	11532	31,39
Gm17384	predicted gene, 17384	ENSMUSG00000090326	74486	31,39
Ube2a	ubiquitin-conjugating enzyme E2A	ENSMUSG00000016308	9858	31,32
Lrrc40	leucine rich repeat containing 40	ENSMUSG00000063052	31817	31,32
		ENSMUSG00000037531	11751	31,32
Enoph1	enolase-phosphatase 1	ENSMUSG00000029326	28769	31,32

Dync2li1	dynein cytoplasmic 2 light intermediate chain 1	ENSMUSG00000024253	29063	31,32
Cmas	cytidine monophospho-N-acetylneuraminic acid synthetase	ENSMUSG00000030282	19029	31,32
		ENSMUSG00000027245	5383	31,32
Blzf1	basic leucine zipper nuclear factor 1	ENSMUSG00000026577	17690	31,32
Taf11	TAF11 RNA polymerase II, TATA box binding protein (TBP)-associated factor	ENSMUSG00000024218	8121	31,32
Golga5	golgi autoantigen, golgin subfamily a, 5	ENSMUSG00000021192	28769	31,32
Pcsk7	proprotein convertase subtilisin/kexin type 7	ENSMUSG00000035382	23124	31,32
Sh2b3	SH2B adaptor protein 3	ENSMUSG00000042594	21372	31,32
Elovl4	elongation of very long chain fatty acids (FEN1/Elo2, SUR4/Elo3, yeast)-like 4	ENSMUSG00000032262	27613	31,24
Polr2g	polymerase (RNA) II (DNA directed) polypeptide G	ENSMUSG00000071662	5429	31,24
Tmem160	transmembrane protein 160	ENSMUSG00000019158	2711	31,24
Grpel2	GrpE-like 2, mitochondrial	ENSMUSG00000024580	13892	31,24
Anapc16	anaphase promoting complex subunit 16	ENSMUSG00000020107	15727	31,24
Bmi1	Bmi1 polycomb ring finger oncogene	ENSMUSG00000026739	9612	31,24
Ankrd52	ankyrin repeat domain 52	ENSMUSG00000014498	31590	31,24
Birc2	baculoviral IAP repeat-containing 2	ENSMUSG00000057367	17020	31,24
Ciz1	CDKN1A interacting zinc finger protein 1	ENSMUSG00000039205	27900	31,24
Kif15	kinesin family member 15	ENSMUSG00000036768	67645	31,24
Evi5l	ecotropic viral integration site 5 like	ENSMUSG00000011832	44691	31,24
		ENSMUSG00000048706	44983	31,24
Spry2	sprouty homolog 2 (Drosophila)	ENSMUSG00000022114	4871	31,24
Sep 09	septin 9	ENSMUSG00000059248	162665	31,24
Prr12	proline rich 12	ENSMUSG00000046574	25175	31,24
Dnah2	dynein, axonemal, heavy chain 2	ENSMUSG00000005237	128302	31,24
Clk3	CDC-like kinase 3	ENSMUSG00000032316	15150	31,17
Vma21	VMA21 vacuolar H+-ATPase homolog (S. cerevisiae)	ENSMUSG00000073131	23834	31,17
Slc4a1ap	solute carrier family 4 (anion exchanger), member 1, adaptor protein	ENSMUSG00000029141	27048	31,17
Scn3b	sodium channel, voltage-gated, type III, beta	ENSMUSG00000049281	22403	31,17
Cpped1	calcineurin-like phosphoesterase domain containing 1	ENSMUSG00000065979	105725	31,17
Hsf2	heat shock factor 2	ENSMUSG00000019878	26749	31,17
Fzd6	frizzled homolog 6 (Drosophila)	ENSMUSG00000022297	31907	31,17
Arhgap24	Rho GTPase activating protein 24	ENSMUSG00000057315	416546	31,17
Higd1c	HIG1 domain family, member 1C	ENSMUSG00000093789	30816	31,17
Dnah3	dynein, axonemal, heavy chain 3	ENSMUSG00000052273	172461	31,17
Tbrg4	transforming growth factor beta regulated gene 4	ENSMUSG00000000384	10470	31,10
Pskh1	protein serine kinase H1	ENSMUSG00000048310	31338	31,10
		ENSMUSG00000062997	4045	31,10

		ENSMUSG00000041777	29597	31,10
Setdb2	SET domain, bifurcated 2	ENSMUSG00000071350	38876	31,10
Hrasls	HRAS-like suppressor	ENSMUSG00000022525	20837	31,10
Ppp3cc	protein phosphatase 3, catalytic subunit, gamma isoform	ENSMUSG00000022092	71552	31,10
Dsc2	desmocollin 2	ENSMUSG00000024331	28922	31,10
Wipi1	WD repeat domain, phosphoinositide interacting 1	ENSMUSG00000041895	38637	31,03
Lrwd1	leucine-rich repeats and WD repeat domain containing 1	ENSMUSG00000029703	13303	31,03
Fis1	fission 1 (mitochondrial outer membrane) homolog (yeast)	ENSMUSG00000019054	12960	31,03
Lyar	Ly1 antibody reactive clone	ENSMUSG00000067367	13837	31,03
Gpr125	G protein-coupled receptor 125	ENSMUSG00000029090	99046	31,03
Mob2	MOB kinase activator 2	ENSMUSG00000025147	52482	31,03
Clybl	citrate lyase beta like	ENSMUSG00000025545	220539	31,03
Pir	pirin	ENSMUSG00000031379	103641	31,03
Uck2	uridine-cytidine kinase 2	ENSMUSG00000026558	59038	31,03
Tmem64	transmembrane protein 64	ENSMUSG00000043252	20923	30,95
P2rx4	purinergic receptor P2X, ligand-gated ion channel 4	ENSMUSG00000029470	22155	30,95
Mpc1	mitochondrial pyruvate carrier 1	ENSMUSG00000023861	14758	30,95
		ENSMUSG00000027710	28966	30,95
Bckdha	branched chain ketoacid dehydrogenase E1, alpha polypeptide	ENSMUSG00000060376	28910	30,95
Mbd1	methyl-CpG binding domain protein 1	ENSMUSG00000024561	14414	30,95
Pot1a	protection of telomeres 1A	ENSMUSG00000029676	65510	30,95
Scamp2	secretory carrier membrane protein 2	ENSMUSG00000040188	27854	30,95
Sugp1	SURP and G patch domain containing 1	ENSMUSG00000011306	29141	30,95
Lin52	lin-52 homolog (C. elegans)	ENSMUSG00000085793	80026	30,95
Daglb	diacylglycerol lipase, beta	ENSMUSG00000039206	39950	30,95
Esrrg	estrogen-related receptor gamma	ENSMUSG00000026610	606095	30,95
Limk1	LIM-domain containing, protein kinase	ENSMUSG00000029674	32552	30,88
Hgs	HGF-regulated tyrosine kinase substrate	ENSMUSG00000025793	16350	30,88
Triap1	TP53 regulated inhibitor of apoptosis 1	ENSMUSG00000029535	2345	30,88
Jagn1	jagunal homolog 1 (Drosophila)	ENSMUSG00000051256	5661	30,88
Rbm48	RNA binding motif protein 48	ENSMUSG00000040302	12608	30,88
Cdk5	cyclin-dependent kinase 5	ENSMUSG00000028969	5281	30,81
Commd4	COMM domain containing 4	ENSMUSG00000032299	3259	30,81
Krba1	KRAB-A domain containing 1	ENSMUSG00000042810	24270	30,81
Prr5l	proline rich 5 like	ENSMUSG00000032841	168743	30,81
Etv3	ets variant gene 3	ENSMUSG00000003382	14750	30,81
Cacnb4	calcium channel, voltage-dependent, beta 4 subunit	ENSMUSG00000017412	248512	30,81
Atrip	ATR interacting protein	ENSMUSG00000025646	16192	30,81
		ENSMUSG00000052372	1374776	30,81
Zdhhc1	zinc finger, DHHC domain containing 1	ENSMUSG00000039199	11870	30,81
Tacr1	tachykinin receptor 1	ENSMUSG00000030043	157629	30,81

Rad23a	RAD23a homolog (S. cerevisiae)	ENSMUSG00000003813	6647	30,74
Txlna	taxilin alpha	ENSMUSG00000053841	14988	30,74
Exoc1	exocyst complex component 1	ENSMUSG00000036435	40984	30,74
Rnasel	ribonuclease L (2', 5'-oligoisoadenylate synthetase-dependent)	ENSMUSG00000066800	14796	30,74
Slc7a6	solute carrier family 7 (cationic amino acid transporter, y+ system), member 6	ENSMUSG00000031904	29830	30,74
C77370	expressed sequence C77370	ENSMUSG00000046449	123752	30,74
Hykk	hydroxylysine kinase 1	ENSMUSG00000035878	32642	30,74
Cdk2ap1	CDK2 (cyclin-dependent kinase 2)-associated protein 1	ENSMUSG00000029394	9255	30,66
Blcap	bladder cancer associated protein homolog (human)	ENSMUSG00000067787	14913	30,66
		ENSMUSG00000021608	49226	30,66
Zfp90	zinc finger protein 90	ENSMUSG00000031907	10551	30,66
Zfp157	zinc finger protein 157	ENSMUSG00000036898	19227	30,66
		ENSMUSG00000020755	33444	30,66
Mpp6	membrane protein, palmitoylated 6 (MAGUK p55 subfamily member 6)	ENSMUSG00000038388	88358	30,66
Ythdc2	YTH domain containing 2	ENSMUSG00000034653	61056	30,66
Rundc3a	RUN domain containing 3A	ENSMUSG00000006575	9153	30,59
Mrpl55	mitochondrial ribosomal protein L55	ENSMUSG00000036860	3649	30,59
6530409C15Rik	RIKEN cDNA 6530409C15 gene	ENSMUSG00000043340	45308	30,59
Lipc	lipase, hepatic	ENSMUSG00000032207	136680	30,59
Pkd2	polycystic kidney disease 2	ENSMUSG00000034462	46370	30,59
Slc9a2	solute carrier family 9 (sodium/hydrogen exchanger), member 2	ENSMUSG00000026062	87174	30,59
Rasip1	Ras interacting protein 1	ENSMUSG00000044562	11606	30,52
Wash	WAS protein family homolog	ENSMUSG00000024101	8958	30,52
Creb3	cAMP responsive element binding protein 3	ENSMUSG00000028466	4729	30,52
Plk4	polo-like kinase 4	ENSMUSG00000025758	16865	30,52
Rnf167	ring finger protein 167	ENSMUSG00000040746	4187	30,52
		ENSMUSG00000024816	25573	30,52
Npas3	neuronal PAS domain protein 3	ENSMUSG00000021010	823499	30,52
Mre11a	meiotic recombination 11 homolog A (S. cerevisiae)	ENSMUSG00000031928	52470	30,52
Tyms	thymidylate synthase	ENSMUSG00000025747	15416	30,52
Trit1	tRNA isopentenyltransferase 1	ENSMUSG00000028653	38353	30,52
Ccdc176	coiled-coil domain containing 176	ENSMUSG00000057265	24710	30,52
Mak	male germ cell-associated kinase	ENSMUSG00000021363	54587	30,52
Rab24	RAB24, member RAS oncogene family	ENSMUSG00000034789	2758	30,45
Carkd	carbohydrate kinase domain containing	ENSMUSG00000031505	17455	30,45
Atg9a	autophagy related 9A	ENSMUSG00000033124	11058	30,45
Fam221b	family with sequence similarity 221, member B	ENSMUSG00000043633	9524	30,45
		ENSMUSG00000042505	86976	30,45
Efemp1	epidermal growth factor-containing fibulin-like extracellular matrix protein 1	ENSMUSG00000020467	73540	30,45

Mtfr1	mitochondrial fission regulator 1	ENSMUSG00000027601	33491	30,45
Plod2	procollagen lysine, 2-oxoglutarate 5-dioxygenase 2	ENSMUSG00000032374	66206	30,45
		ENSMUSG00000091415	130555	30,45
		ENSMUSG00000044471	52911	30,45
Frmd6	FERM domain containing 6	ENSMUSG00000048285	76719	30,37
Romo1	reactive oxygen species modulator 1	ENSMUSG00000067847	1759	30,37
Insig2	insulin induced gene 2	ENSMUSG00000003721	28237	30,37
		ENSMUSG00000024014	4634	30,37
Mettl20	methyltransferase like 20	ENSMUSG00000039958	11323	30,37
Hpcal1	hippocalcin-like 1	ENSMUSG00000071379	101113	30,37
Sybu	syntabulin (syntaxin-interacting)	ENSMUSG00000022340	116205	30,37
Letm2	leucine zipper-EF-hand containing transmembrane protein 2	ENSMUSG00000037363	18996	30,37
Arhgef1	Rho guanine nucleotide exchange factor (GEF) 1	ENSMUSG00000040940	23681	30,37
Tenm1	teneurin transmembrane protein 1	ENSMUSG00000016150	901261	30,37
Rogdi	rogdi homolog (Drosophila)	ENSMUSG00000022540	4788	30,30
Sgpp1	sphingosine-1-phosphate phosphatase 1	ENSMUSG00000021054	21482	30,30
Uspl1	ubiquitin specific peptidase like 1	ENSMUSG00000041264	31085	30,30
		ENSMUSG00000029720	13725	30,30
Abhd6	abhydrolase domain containing 6	ENSMUSG00000025277	53654	30,30
		ENSMUSG00000070953	22357	30,30
Ccdc148	coiled-coil domain containing 148	ENSMUSG00000036641	338987	30,30
Plagl2	pleiomorphic adenoma gene-like 2	ENSMUSG00000051413	13668	30,30
Gm15155	predicted gene 15155	ENSMUSG00000055109	721147	30,30
Coq10a	coenzyme Q10 homolog A (yeast)	ENSMUSG00000039914	6936	30,23
Orai2	ORAI calcium release-activated calcium modulator 2	ENSMUSG00000039747	23193	30,23
		ENSMUSG00000019731	11974	30,23
Fam160b2	family with sequence similarity 160, member B2	ENSMUSG00000022095	16540	30,23
Flii	flightless I homolog (Drosophila)	ENSMUSG00000002812	13141	30,23
Pccb	propionyl Coenzyme A carboxylase, beta polypeptide	ENSMUSG00000032527	52867	30,23
Mtmr2	myotubularin related protein 2	ENSMUSG00000031918	58072	30,23
Nova2	neuro-oncological ventral antigen 2	ENSMUSG00000030411	39432	30,23
Pkd1	polycystic kidney disease 1 homolog	ENSMUSG00000032855	46565	30,23
Katna1	katanin p60 (ATPase-containing) subunit A1	ENSMUSG00000019794	37151	30,23
Prex1	phosphatidylinositol-3,4,5-trisphosphate-dependent Rac exchange factor 1	ENSMUSG00000039621	147491	30,16
Fam92b	family with sequence similarity 92, member B	ENSMUSG00000042269	11070	30,16
AI462493	expressed sequence AI462493	ENSMUSG00000071654	918	30,16
Slc39a6	solute carrier family 39 (metal ion transporter), member 6	ENSMUSG00000024270	23937	30,16
Cby1	chibby homolog 1 (Drosophila)	ENSMUSG00000022428	8434	30,16
Vps35	vacuolar protein sorting 35	ENSMUSG00000031696	39117	30,16
Rbmx2	RNA binding motif protein, X-linked 2	ENSMUSG00000031107	15720	30,16
Zxdc	ZXD family zinc finger C	ENSMUSG00000034430	33999	30,16

Tmem45b	transmembrane protein 45b	ENSMUSG00000041737	38040	30,16
Qsox1	quiescin Q6 sulfhydryl oxidase 1	ENSMUSG00000033684	34729	30,08
Tnfaip8	tumor necrosis factor, alpha-induced protein 8	ENSMUSG00000062210	127747	30,08
Ptcd1	pentatricopeptide repeat domain 1	ENSMUSG00000029624	19595	30,08
Ezh1	enhancer of zeste homolog 1 (Drosophila)	ENSMUSG00000006920	35349	30,08
1810026J23Rik	RIKEN cDNA 1810026J23 gene	ENSMUSG00000048429	3249	30,01
Wdr41	WD repeat domain 41	ENSMUSG00000042015	46971	30,01
Pak1	p21 protein (Cdc42/Rac)-activated kinase 1	ENSMUSG00000030774	69447	30,01
Pet100	PET100 homolog (S. cerevisiae)	ENSMUSG00000087687	2681	30,01
Fam227a	family with sequence similarity 227, member A	ENSMUSG00000042564	49381	30,01
Prpsap2	phosphoribosyl pyrophosphate synthetase-associated protein 2	ENSMUSG00000020528	32439	30,01
Arnt2	aryl hydrocarbon receptor nuclear translocator 2	ENSMUSG00000015709	163679	30,01
Fam65b	family with sequence similarity 65, member B	ENSMUSG00000036006	151628	30,01
1700007B14Rik	RIKEN cDNA 1700007B14 gene	ENSMUSG00000031620	535810	30,01
Syt16	synaptotagmin XVI	ENSMUSG00000044912	270156	30,01
Arhgef6	Rac/Cdc42 guanine nucleotide exchange factor (GEF) 6	ENSMUSG00000031133	107245	30,01
Kcnab2	potassium voltage-gated channel, shaker-related subfamily, beta member 2	ENSMUSG00000028931	87130	29,94
Mphosph6	M phase phosphoprotein 6	ENSMUSG00000031843	10285	29,94
Ddx1	DEAD (Asp-Glu-Ala-Asp) box polypeptide 1	ENSMUSG00000037149	29906	29,94
Ptrh2	peptidyl-tRNA hydrolase 2	ENSMUSG00000072582	8473	29,94
Slc25a17	solute carrier family 25 (mitochondrial carrier, peroxisomal membrane protein), member 17	ENSMUSG00000022404	41845	29,94
Snx11	sorting nexin 11	ENSMUSG00000020876	10005	29,94
2510039O18Rik	RIKEN cDNA 2510039O18 gene	ENSMUSG00000044496	6420	29,94
Ccs	copper chaperone for superoxide dismutase	ENSMUSG00000034108	13957	29,94
Aph1b	anterior pharynx defective 1b homolog (C. elegans)	ENSMUSG00000032375	20289	29,94
Nat9	N-acetyltransferase 9 (GCN5-related, putative)	ENSMUSG00000015542	5028	29,94
Atl3	atlastin GTPase 3	ENSMUSG00000024759	44569	29,94
Vwa5b2	von Willebrand factor A domain containing 5B2	ENSMUSG00000046613	15907	29,94
Stat5b	signal transducer and activator of transcription 5B	ENSMUSG00000020919	69994	29,94
Med27	mediator complex subunit 27	ENSMUSG00000026799	177975	29,94
Bcl2l11	BCL2-like 11 (apoptosis facilitator)	ENSMUSG00000027381	36510	29,94
		ENSMUSG00000073465	100572	29,94
Gtf2f1	general transcription factor IIF, polypeptide 1	ENSMUSG00000002658	7884	29,87
Rce1	RCE1 homolog, prenyl protein peptidase (S. cerevisiae)	ENSMUSG00000024889	3067	29,87
		ENSMUSG00000092534	15005	29,87
		ENSMUSG00000033184	37301	29,87

Pick1	protein interacting with C kinase 1	ENSMUSG00000068206	27502	29,87
		ENSMUSG00000019715	24946	29,87
		ENSMUSG00000090115	56049	29,87
Tmem110	transmembrane protein 110	ENSMUSG00000006526	51397	29,87
		ENSMUSG00000025451	29618	29,87
Morc2a	microrchidia 2A	ENSMUSG00000034543	40984	29,87
		ENSMUSG00000017418	33062	29,87
Iqca	IQ motif containing with AAA domain	ENSMUSG00000026301	111270	29,87
Sox9	SRY-box containing gene 9	ENSMUSG00000000567	5537	29,79
Tm2d2	TM2 domain containing 2	ENSMUSG00000031556	6049	29,79
Mrps34	mitochondrial ribosomal protein S34	ENSMUSG00000038880	2387	29,79
Tspan8	tetraspanin 8	ENSMUSG00000034127	32610	29,79
Pmpca	peptidase (mitochondrial processing) alpha	ENSMUSG00000026926	7784	29,79
Sgsm3	small G protein signaling modulator 3	ENSMUSG00000042303	34525	29,79
Tmem41a	transmembrane protein 41a	ENSMUSG00000022856	13226	29,79
Nup188	nucleoporin 188	ENSMUSG00000052533	57870	29,79
Zfp655	zinc finger protein 655	ENSMUSG00000007812	15580	29,79
Exosc10	exosome component 10	ENSMUSG00000017264	23973	29,79
Ttll4	tubulin tyrosine ligase-like family, member 4	ENSMUSG00000033257	40088	29,79
Zfp715	zinc finger protein 715	ENSMUSG00000012640	17098	29,79
		ENSMUSG00000079450	85301	29,79
Plekhb1	pleckstrin homology domain containing, family B (evectins) member 1	ENSMUSG00000030701	19523	29,72
Slc37a1	solute carrier family 37 (glycerol-3-phosphate transporter), member 1	ENSMUSG00000024036	55214	29,72
Zc3h18	zinc finger CCCH-type containing 18	ENSMUSG00000017478	40752	29,72
Tbc1d19	TBC1 domain family, member 19	ENSMUSG00000039178	94754	29,72
Slit1	slit homolog 1 (Drosophila)	ENSMUSG00000025020	143409	29,72
Eno4	enolase 4	ENSMUSG00000048029	27995	29,72
Cog6	component of oligomeric golgi complex 6	ENSMUSG00000027742	35101	29,65
Bag5	BCL2-associated athanogene 5	ENSMUSG00000049792	3770	29,65
Fam161b	family with sequence similarity 161, member B	ENSMUSG00000021234	16525	29,65
Bag4	BCL2-associated athanogene 4	ENSMUSG00000037316	20672	29,65
Ttc21a	tetratricopeptide repeat domain 21A	ENSMUSG00000032514	30188	29,65
Itgav	integrin alpha V	ENSMUSG00000027087	82520	29,65
Gm9803	predicted gene 9803	ENSMUSG00000045886	516	29,65
Plec	plectin	ENSMUSG00000022565	61601	29,65
Idh3b	isocitrate dehydrogenase 3 (NAD+) beta	ENSMUSG00000027406	5239	29,58
Tmx2	thioredoxin-related transmembrane protein 2	ENSMUSG00000050043	7825	29,58
Ppox	protoporphyrinogen oxidase	ENSMUSG00000062729	5197	29,58
Src	Rous sarcoma oncogene	ENSMUSG00000027646	53419	29,58
Hif1an	hypoxia-inducible factor 1, alpha subunit inhibitor	ENSMUSG00000036450	13425	29,58

Slc48a1	solute carrier family 48 (heme transporter), member 1	ENSMUSG00000081534	8338	29,58
Tmem237	transmembrane protein 237	ENSMUSG00000038079	19503	29,58
Fbrs	fibrosin	ENSMUSG00000042423	6293	29,58
Nprl3	nitrogen permease regulator-like 3	ENSMUSG00000020289	42080	29,58
Zrsr2	zinc finger (CCCH type), RNA binding motif and serine/arginine rich 2	ENSMUSG00000031370	23219	29,58
Snap23	synaptosomal-associated protein 23	ENSMUSG00000027287	33585	29,58
F3	coagulation factor III	ENSMUSG00000028128	11516	29,58
Brinp1	bone morphogenic protein/retinoic acid inducible neural specific 1	ENSMUSG00000028351	192884	29,58
Cript	cysteine-rich PDZ-binding protein	ENSMUSG00000024146	10261	29,50
Utp6	UTP6, small subunit (SSU) processome component, homolog (yeast)	ENSMUSG00000035575	30070	29,50
Adam12	a disintegrin and metallopeptidase domain 12 (meltrin alpha)	ENSMUSG00000054555	348948	29,50
		ENSMUSG00000028706	21035	29,50
Noc3l	nucleolar complex associated 3 homolog (S. cerevisiae)	ENSMUSG00000024999	31110	29,50
		ENSMUSG00000020935	34287	29,43
Max	Max protein	ENSMUSG00000059436	24980	29,43
Ttll6	tubulin tyrosine ligase-like family, member 6	ENSMUSG00000038756	31666	29,43
Atp6v1d	ATPase, H+ transporting, lysosomal V1 subunit D	ENSMUSG00000021114	18650	29,43
Rnmt	RNA (guanine-7-) methyltransferase	ENSMUSG00000009535	24498	29,43
Matn2	matrilin 2	ENSMUSG00000022324	129563	29,43
Map3k10	mitogen-activated protein kinase kinase kinase 10	ENSMUSG00000040390	18224	29,43
Syne3	spectrin repeat containing, nuclear envelope family member 3	ENSMUSG00000054150	79861	29,43
Hprt	hypoxanthine guanine phosphoribosyl transferase	ENSMUSG00000025630	33523	29,36
Eci2	enoyl-Coenzyme A delta isomerase 2	ENSMUSG00000021417	49347	29,36
Aacs	acetoacetyl-CoA synthetase	ENSMUSG00000029482	41599	29,36
Cstf2	cleavage stimulation factor, 3' pre-RNA subunit 2	ENSMUSG00000031256	27633	29,36
Riok1	RIO kinase 1 (yeast)	ENSMUSG00000021428	24445	29,36
Rara	retinoic acid receptor, alpha	ENSMUSG00000037992	47125	29,36
Pde8b	phosphodiesterase 8B	ENSMUSG00000021684	225883	29,36
Lamtor5	late endosomal/lysosomal adaptor, MAPK and MTOR activator 5	ENSMUSG00000087260	5222	29,29
Adck4	aarF domain containing kinase 4	ENSMUSG00000003762	29230	29,29
Usp38	ubiquitin specific peptidase 38	ENSMUSG00000038250	34173	29,29
Nat6	N-acetyltransferase 6	ENSMUSG00000079334	5162	29,29
Fam189b	family with sequence similarity 189, member B	ENSMUSG00000032657	6153	29,29
Cmc1	COX assembly mitochondrial protein 1	ENSMUSG00000039163	85668	29,29
		ENSMUSG00000032024	99358	29,21
		ENSMUSG00000044788	18042	29,21

Grk6	G protein-coupled receptor kinase 6	ENSMUSG00000074886	15592	29,21
Tsc22d3	TSC22 domain family, member 3	ENSMUSG00000031431	61132	29,21
Smim11	small integral membrane protein 11	ENSMUSG00000051989	11756	29,21
2010012O05Rik	RIKEN cDNA 2010012O05 gene	ENSMUSG00000062376	13477	29,21
Poli	polymerase (DNA directed), iota	ENSMUSG00000038425	21941	29,21
Lsm1	LSM1 homolog, U6 small nuclear RNA associated (S. cerevisiae)	ENSMUSG00000037296	18385	29,21
Ogg1	8-oxoguanine DNA-glycosylase 1	ENSMUSG00000030271	8097	29,21
Zfp668	zinc finger protein 668	ENSMUSG00000049728	11659	29,21
Ctns	cystinosis, nephropathic	ENSMUSG00000005949	15447	29,21
Fgf13	fibroblast growth factor 13	ENSMUSG00000031137	505927	29,21
Xylb	xylulokinase homolog (H. influenzae)	ENSMUSG00000035769	36417	29,14
Ppfibp2	PTPRF interacting protein, binding protein 2 (liprin beta 2)	ENSMUSG00000036528	153532	29,14
Atat1	alpha tubulin acetyltransferase 1	ENSMUSG00000024426	12481	29,14
Slc44a2	solute carrier family 44, member 2	ENSMUSG00000057193	17198	29,14
Ubtd1	ubiquitin domain containing 1	ENSMUSG00000025171	52879	29,14
Zbtb21	zinc finger and BTB domain containing 21	ENSMUSG00000046962	15187	29,14
Inpp5a	inositol polyphosphate-5-phosphatase A	ENSMUSG00000025477	190544	29,14
Trim45	tripartite motif-containing 45	ENSMUSG00000033233	14724	29,07
		ENSMUSG00000001313	6855	29,07
Pdcl	phosducin-like	ENSMUSG00000009030	9259	29,07
Tmem230	transmembrane protein 230	ENSMUSG00000027341	8316	29,07
Gp1bb	glycoprotein Ib, beta polypeptide	ENSMUSG00000050761	2085	29,07
Smim8	small integral membrane protein 8	ENSMUSG00000028295	9760	29,07
		ENSMUSG00000002846	24317	29,07
Ptger4	prostaglandin E receptor 4 (subtype EP4)	ENSMUSG00000039942	37527	29,07
Nup160	nucleoporin 160	ENSMUSG00000051329	59114	29,07
Recql5	RecQ protein-like 5	ENSMUSG00000020752	40883	29,07
Cep57	centrosomal protein 57	ENSMUSG00000031922	19316	29,07
Armc3	armadillo repeat containing 3	ENSMUSG00000037683	110940	29,07
Slc9a7	solute carrier family 9 (sodium/hydrogen exchanger), member 7	ENSMUSG00000037341	186053	29,07
Simc1	SUMO-interacting motifs containing 1	ENSMUSG00000043183	47512	29,07
Arf5	ADP-ribosylation factor 5	ENSMUSG00000020440	3043	29,00
		ENSMUSG00000040236	4957	29,00
Gna11	guanine nucleotide binding protein, alpha 11	ENSMUSG00000034781	16467	29,00
Slu7	SLU7 splicing factor homolog (S. cerevisiae)	ENSMUSG00000020409	14238	29,00
Zdhhc16	zinc finger, DHHC domain containing 16	ENSMUSG00000025157	10624	29,00
Anapc7	anaphase promoting complex subunit 7	ENSMUSG00000029466	23220	29,00
Gfm2	G elongation factor, mitochondrial 2	ENSMUSG00000021666	43259	29,00
Nkap	NFKB activating protein	ENSMUSG00000016409	23952	29,00
Fgfr1op	Fgfr1 oncogene partner	ENSMUSG00000069135	31304	29,00
Traf3ip2	TRAF3 interacting protein 2	ENSMUSG00000019842	42374	29,00

Fbxl3	F-box and leucine-rich repeat protein 3	ENSMUSG00000022124	19328	29,00
Sgce	sarcoglycan, epsilon	ENSMUSG00000004631	72855	29,00
Qtrtd1	queuine tRNA-ribosyltransferase domain containing 1	ENSMUSG00000022704	65403	29,00
Mansc4	MANSC domain containing 4	ENSMUSG00000072662	11973	29,00
Ak7	adenylate kinase 7	ENSMUSG00000041323	76466	29,00
Pbdc1	polysaccharide biosynthesis domain containing 1	ENSMUSG00000031226	37335	28,92
0610030E20Rik	RIKEN cDNA 0610030E20 gene	ENSMUSG00000058706	5842	28,92
Pak1ip1	PAK1 interacting protein 1	ENSMUSG00000038683	11997	28,92
Ints3	integrator complex subunit 3	ENSMUSG00000027933	42257	28,92
Sgk3	serum/glucocorticoid regulated kinase 3	ENSMUSG00000025915	104427	28,92
Rnf138	ring finger protein 138	ENSMUSG00000024317	26883	28,92
E430025E21Rik	RIKEN cDNA E430025E21 gene	ENSMUSG00000022350	42170	28,92
Asb8	ankyrin repeat and SOCS box-containing 8	ENSMUSG00000048175	30989	28,85
Sdhaf1	succinate dehydrogenase complex assembly factor 1	ENSMUSG00000074211	965	28,85
Rnf219	ring finger protein 219	ENSMUSG00000022120	45133	28,85
Cep250	centrosomal protein 250	ENSMUSG00000038241	42443	28,85
Fmo1	flavin containing monooxygenase 1	ENSMUSG00000040181	36988	28,85
Ddx55	DEAD (Asp-Glu-Ala-Asp) box polypeptide 55	ENSMUSG00000029389	16797	28,85
		ENSMUSG00000024948	14481	28,85
		ENSMUSG00000095832	35940	28,85
Tmem102	transmembrane protein 102	ENSMUSG00000089876	2022	28,85
Snx2	sorting nexin 2	ENSMUSG00000034484	44496	28,85
Mdm4	transformed mouse 3T3 cell double minute 4	ENSMUSG00000054387	35522	28,85
Slc22a5	solute carrier family 22 (organic cation transporter), member 5	ENSMUSG00000018900	27119	28,85
Gal3st2	galactose-3-O-sulfotransferase 2	ENSMUSG00000094651	15151	28,85
Pced1b	PC-esterase domain containing 1B	ENSMUSG00000044250	138583	28,85
Mdk	midkine	ENSMUSG00000027239	2493	28,78
Nrbf2	nuclear receptor binding factor 2	ENSMUSG00000075000	18617	28,78
Paxip1	PAX interacting (with transcription-activation domain) protein 1	ENSMUSG00000002221	50673	28,78
Wdr81	WD repeat domain 81	ENSMUSG00000045374	13774	28,78
Arfgap1	ADP-ribosylation factor GTPase activating protein 1	ENSMUSG00000027575	15302	28,78
Slc35b3	solute carrier family 35, member B3	ENSMUSG00000021432	28736	28,78
		ENSMUSG00000039704	57698	28,78
Hsdl1	hydroxysteroid dehydrogenase like 1	ENSMUSG00000034189	13222	28,71
		ENSMUSG00000091449	372	28,71
Tmub2	transmembrane and ubiquitin-like domain containing 2	ENSMUSG00000034757	4307	28,71
Slc3a1	solute carrier family 3, member 1	ENSMUSG00000024131	35897	28,71
		ENSMUSG00000097919	59294	28,71
Akip1	A kinase (PRKA) interacting protein 1	ENSMUSG00000031023	8500	28,71
Noc2l	nucleolar complex associated 2 homolog (S. cerevisiae)	ENSMUSG00000095567	11607	28,71

Sowahc	sosondowah ankyrin repeat domain family member C	ENSMUSG00000098188	4482	28,71
1500011B03Rik	RIKEN cDNA 1500011B03 gene	ENSMUSG00000072694	5781	28,63
Isyna1	myo-inositol 1-phosphate synthase A1	ENSMUSG00000019139	2808	28,63
Smpd2	sphingomyelin phosphodiesterase 2, neutral	ENSMUSG00000019822	4728	28,63
Taok3	TAO kinase 3	ENSMUSG00000061288	155091	28,63
Ndufs3	NADH dehydrogenase (ubiquinone) Fe-S protein 3	ENSMUSG00000005510	10194	28,63
Bin3	bridging integrator 3	ENSMUSG00000022089	38047	28,63
Naa16	N(alpha)-acetyltransferase 16, NatA auxiliary subunit	ENSMUSG00000022020	56575	28,63
Zfyve16	zinc finger, FYVE domain containing 16	ENSMUSG00000021706	43761	28,63
Gm6793	predicted gene 6793	ENSMUSG00000092086	1604	28,56
Elovl1	elongation of very long chain fatty acids (FEN1/Elo2, SUR4/Elo3, yeast)-like 1	ENSMUSG00000006390	4861	28,56
Mat2b	methionine adenosyltransferase II, beta	ENSMUSG00000042032	15890	28,56
		ENSMUSG00000095687	19488	28,56
Ddrgk1	DDRGK domain containing 1	ENSMUSG00000068290	10700	28,56
Ccdc170	coiled-coil domain containing 170	ENSMUSG00000019767	79730	28,56
Mto1	mitochondrial translation optimization 1 homolog (S. cerevisiae)	ENSMUSG00000032342	27141	28,56
Ofd1	oral-facial-digital syndrome 1 gene homolog (human)	ENSMUSG00000040586	50672	28,56
Fen1	flap structure specific endonuclease 1	ENSMUSG00000024742	5038	28,49
Exosc4	exosome component 4	ENSMUSG00000034259	3281	28,49
G6pdx	glucose-6-phosphate dehydrogenase X-linked	ENSMUSG00000031400	19712	28,49
Dhfr	dihydrofolate reductase	ENSMUSG00000021707	34271	28,49
Hrsp12	heat-responsive protein 12	ENSMUSG00000022323	11235	28,49
Mthfd2l	methylenetetrahydrofolate dehydrogenase (NADP+ dependent) 2-like	ENSMUSG00000029376	90165	28,49
Mink1	misshapen-like kinase 1 (zebrafish)	ENSMUSG00000020827	51603	28,49
Prdx3	peroxiredoxin 3	ENSMUSG00000024997	10506	28,42
		ENSMUSG00000093593	7299	28,42
Neurl1b	neuralized homolog 1b (Drosophila)	ENSMUSG00000034413	31521	28,42
Ankrd13b	ankyrin repeat domain 13b	ENSMUSG00000037907	19194	28,42
Samhd1	SAM domain and HD domain, 1	ENSMUSG00000027639	37733	28,42
Tceb3	transcription elongation factor B (SIII), polypeptide 3	ENSMUSG00000028668	18396	28,42
Pcnxl4	pecanex-like 4 (Drosophila)	ENSMUSG00000034501	43857	28,42
		ENSMUSG00000041552	50360	28,42
Shisa5	shisa homolog 5 (Xenopus laevis)	ENSMUSG00000025647	19160	28,42
		ENSMUSG00000078862	21305	28,42
Exoc6	exocyst complex component 6	ENSMUSG00000053799	132828	28,42
Zbed4	zinc finger, BED domain containing 4	ENSMUSG00000034333	32857	28,34
Tial1	Tia1 cytotoxic granule-associated RNA binding protein-like 1	ENSMUSG00000030846	21941	28,34

Psme1	proteasome (prosome, macropain) activator subunit 1 (PA28 alpha)	ENSMUSG00000022216	3407	28,34
Wdr59	WD repeat domain 59	ENSMUSG00000031959	73324	28,34
Stra13	stimulated by retinoic acid 13	ENSMUSG00000025144	2797	28,34
Msl3	male-specific lethal 3 homolog (Drosophila)	ENSMUSG00000031358	19782	28,34
Sowaha	sosondowah ankyrin repeat domain family member A	ENSMUSG00000044352	3617	28,27
Timm44	translocase of inner mitochondrial membrane 44	ENSMUSG00000002949	16183	28,27
Fam174a	family with sequence similarity 174, member A	ENSMUSG00000051185	21657	28,27
Nrde2	nrde-2 necessary for RNA interference, domain containing	ENSMUSG00000021179	34202	28,27
Gtf3c6	general transcription factor IIIC, polypeptide 6, alpha	ENSMUSG00000019837	8463	28,20
Med23	mediator complex subunit 23	ENSMUSG00000019984	43696	28,20
Cog3	component of oligomeric golgi complex 3	ENSMUSG00000034893	52143	28,20
Bzrap1	benzodiazepine receptor associated protein 1	ENSMUSG00000034156	25388	28,13
Vps51	vacuolar protein sorting 51 homolog (S. cerevisiae)	ENSMUSG00000024797	12483	28,13
		ENSMUSG00000064037	18163	28,13
Bax	BCL2-associated X protein	ENSMUSG00000003873	5204	28,13
Slc9a6	solute carrier family 9 (sodium/hydrogen exchanger), member 6	ENSMUSG00000060681	54474	28,13
Llph	LLP homolog, long-term synaptic facilitation (Aplysia)	ENSMUSG00000020224	5002	28,13
Snrnp27	small nuclear ribonucleoprotein 27 (U4/U6.U5)	ENSMUSG00000001158	9302	28,13
Polr3f	polymerase (RNA) III (DNA directed) polypeptide F	ENSMUSG00000027427	14278	28,13
Mospd1	motile sperm domain containing 1	ENSMUSG00000023074	25905	28,13
Tmem180	transmembrane protein 180	ENSMUSG00000025227	34132	28,13
Arhgap1	Rho GTPase activating protein 1	ENSMUSG00000027247	22467	28,13
Dnaja4	DnaJ (Hsp40) homolog, subfamily A, member 4	ENSMUSG00000032285	17443	28,13
Hist1h2be	histone cluster 1, H2be	ENSMUSG00000047246	37383	28,13
		ENSMUSG00000028039	9067	28,05
		ENSMUSG00000029463	7785	28,05
Pip5k1c	phosphatidylinositol-4-phosphate 5-kinase, type 1 gamma	ENSMUSG00000034902	27011	28,05
Rad54l	RAD54 like (S. cerevisiae)	ENSMUSG00000028702	29427	28,05
Katnbl1	katanin p80 subunit B like 1	ENSMUSG00000027132	35030	28,05
Ttpal	tocopherol (alpha) transfer protein-like	ENSMUSG00000017679	16700	28,05
Srbd1	S1 RNA binding domain 1	ENSMUSG00000024135	160511	28,05
Pvrl4	poliovirus receptor-related 4	ENSMUSG00000006411	18500	28,05
		ENSMUSG00000044949	61318	28,05
Hap1	huntingtin-associated protein 1	ENSMUSG00000006930	8802	27,98
Rps13	ribosomal protein S13	ENSMUSG00000090862	2684	27,98
Dvl2	dishevelled 2, dsh homolog (Drosophila)	ENSMUSG00000020888	11707	27,98

Ahnak	AHNAK nucleoprotein (desmoyokin)	ENSMUSG00000069833	87636	27,98
		ENSMUSG00000031095	42923	27,98
		ENSMUSG00000040296	36056	27,98
Dhrs3	dehydrogenase/reductase (SDR family) member 3	ENSMUSG00000066026	35383	27,98
Sptbn2	spectrin beta, non-erythrocytic 2	ENSMUSG00000067889	41146	27,98
		ENSMUSG00000057244	666	27,91
Rnf168	ring finger protein 168	ENSMUSG00000014074	23976	27,91
		ENSMUSG00000030869	16484	27,91
Adrb1	adrenergic receptor, beta 1	ENSMUSG00000035283	2491	27,91
Faim2	Fas apoptotic inhibitory molecule 2	ENSMUSG00000023011	31013	27,84
Gja1	gap junction protein, alpha 1	ENSMUSG00000050953	13120	27,84
		ENSMUSG00000030680	18846	27,84
		ENSMUSG00000031146	3450	27,84
Cnih1	cornichon homolog 1 (Drosophila)	ENSMUSG00000015759	12830	27,84
Kmt2d	lysine (K)-specific methyltransferase 2D	ENSMUSG00000048154	39536	27,84
Spata1	spermatogenesis associated 1	ENSMUSG00000028188	42551	27,84
Rtn4rl1	reticulon 4 receptor-like 1	ENSMUSG00000045287	73987	27,76
Ebna1bp2	EBNA1 binding protein 2	ENSMUSG00000028729	6978	27,76
Cgnl1	cingulin-like 1	ENSMUSG00000032232	145094	27,76
Myl12a	myosin, light chain 12A, regulatory, non-sarcomeric	ENSMUSG00000024048	9223	27,76
BC029214	cDNA sequence BC029214	ENSMUSG00000047617	5954	27,76
Syvn1	synovial apoptosis inhibitor 1, synoviolin	ENSMUSG00000024807	7137	27,76
Msto1	misato homolog 1 (Drosophila)	ENSMUSG00000068922	8893	27,76
Rpap2	RNA polymerase II associated protein 2	ENSMUSG00000033773	64466	27,76
Hmgxb3	HMG box domain containing 3	ENSMUSG00000024622	45772	27,76
Vps13c	vacuolar protein sorting 13C (yeast)	ENSMUSG00000035284	155239	27,76
Malt1	mucosa associated lymphoid tissue lymphoma translocation gene 1	ENSMUSG00000032688	47861	27,76
Birc5	baculoviral IAP repeat-containing 5	ENSMUSG00000017716	6493	27,69
Rfxap	regulatory factor X-associated protein	ENSMUSG00000036615	4677	27,69
Gm10093	predicted pseudogene 10093	ENSMUSG00000061062	1977	27,69
Foxj2	forkhead box J2	ENSMUSG00000003154	25453	27,69
Arrb1	arrestin, beta 1	ENSMUSG00000018909	71306	27,69
Ctdp1	CTD (carboxy-terminal domain, RNA polymerase II, polypeptide A) phosphatase, subunit 1	ENSMUSG00000033323	61737	27,69
Tbc1d16	TBC1 domain family, member 16	ENSMUSG00000039976	85455	27,62
Rpp14	ribonuclease P 14 subunit	ENSMUSG00000023156	11468	27,62
Frat2	frequently rearranged in advanced T cell lymphomas 2	ENSMUSG00000047604	2155	27,62
Kptn	kaptin	ENSMUSG00000006021	7622	27,62
Slc2a3	solute carrier family 2 (facilitated glucose transporter), member 3	ENSMUSG00000003153	73832	27,62
Degs2	degenerative spermatocyte homolog 2 (Drosophila), lipid desaturase	ENSMUSG00000021263	15515	27,55
Tubgcp2	tubulin, gamma complex associated protein 2	ENSMUSG00000025474	40396	27,55

Eci1	enoyl-Coenzyme A delta isomerase 1	ENSMUSG00000024132	12634	27,55
Cenpj	centromere protein J	ENSMUSG00000064128	45086	27,55
Akt1	thymoma viral proto-oncogene 1	ENSMUSG00000001729	21064	27,47
Sccpdh	saccharopine dehydrogenase (putative)	ENSMUSG00000038936	18980	27,47
		ENSMUSG00000031158	8995	27,47
Nsdhl	NAD(P) dependent steroid dehydrogenase-like	ENSMUSG00000031349	39958	27,47
Gfer	growth factor, erv1 (S. cerevisiae)-like (augmenter of liver regeneration)	ENSMUSG00000040888	2966	27,47
1110008F13Rik	RIKEN cDNA 1110008F13 gene	ENSMUSG00000027637	10436	27,47
Dkc1	dyskeratosis congenita 1, dyskerin	ENSMUSG00000031403	13924	27,47
Dagla	diacylglycerol lipase, alpha	ENSMUSG00000035735	59613	27,47
Tppp3	tubulin polymerization-promoting protein family member 3	ENSMUSG00000014846	4034	27,47
Mapkbp1	mitogen-activated protein kinase binding protein 1	ENSMUSG00000033902	54710	27,47
		ENSMUSG00000002365	89706	27,47
2610020H08Rik	RIKEN cDNA 2610020H08 gene	ENSMUSG00000030924	54938	27,47
Gng10	guanine nucleotide binding protein (G protein), gamma 10	ENSMUSG00000038607	6816	27,40
		ENSMUSG00000046756	4112	27,40
Polr3d	polymerase (RNA) III (DNA directed) polypeptide D	ENSMUSG00000000776	4724	27,40
6030458C11Rik	RIKEN cDNA 6030458C11 gene	ENSMUSG00000022195	16481	27,40
4922501L14Rik	RIKEN cDNA 4922501L14 gene	ENSMUSG00000042943	93432	27,40
Taf1b	TATA box binding protein (Tbp)-associated factor, RNA polymerase I, B	ENSMUSG00000059669	59991	27,40
Rpgrip1	retinitis pigmentosa GTPase regulator interacting protein 1	ENSMUSG00000057132	52843	27,40
Tsen34	tRNA splicing endonuclease 34 homolog (S. cerevisiae)	ENSMUSG00000035585	7649	27,40
Prkch	protein kinase C, eta	ENSMUSG00000021108	193389	27,40
Greb1l	growth regulation by estrogen in breast cancer-like	ENSMUSG00000042942	237758	27,40
Mrps24	mitochondrial ribosomal protein S24	ENSMUSG00000020477	11698	27,33
Ctsa	cathepsin A	ENSMUSG00000017760	8160	27,33
Ube2v1	ubiquitin-conjugating enzyme E2 variant 1	ENSMUSG00000078923	24458	27,33
Znhit2	zinc finger, HIT domain containing 2	ENSMUSG00000075227	1279	27,33
Narfl	nuclear prelamin A recognition factor-like	ENSMUSG00000002280	9557	27,33
		ENSMUSG00000039354	49884	27,33
Bnip1	BCL2/adenovirus E1B interacting protein 1	ENSMUSG00000024191	11487	27,33
Abce1	ATP-binding cassette, sub-family E (OABP), member 1	ENSMUSG00000058355	28299	27,33
Zfp426	zinc finger protein 426	ENSMUSG00000059475	24198	27,33
Itgb3bp	integrin beta 3 binding protein (beta3-endonexin)	ENSMUSG00000028549	164412	27,33

Trpc4ap	transient receptor potential cation channel, subfamily C, member 4 associated protein	ENSMUSG00000038324	58114	27,33
Cdc42ep4	CDC42 effector protein (Rho GTPase binding) 4	ENSMUSG00000041598	25032	27,33
		ENSMUSG00000035245	39159	27,33
Ankfn1	ankyrin-repeat and fibronectin type III domain containing 1	ENSMUSG00000047773	218645	27,33
Mest	mesoderm specific transcript	ENSMUSG00000051855	24909	27,26
Racgap1	Rac GTPase-activating protein 1	ENSMUSG00000023015	31161	27,26
Tprkb	Tp53rk binding protein	ENSMUSG00000054226	14547	27,26
Aga	aspartylglucosaminidase	ENSMUSG00000031521	11720	27,26
Rps7	ribosomal protein S7	ENSMUSG00000061477	5107	27,26
Anapc2	anaphase promoting complex subunit 2	ENSMUSG00000026965	13438	27,26
Galnt3	UDP-N-acetyl-alpha-D-galactosamine:polypeptide N-acetylgalactosaminyltransferase 3	ENSMUSG00000026994	42229	27,26
Slc39a9	solute carrier family 39 (zinc transporter), member 9	ENSMUSG00000048833	39127	27,26
Hyou1	hypoxia up-regulated 1	ENSMUSG00000032115	12880	27,26
Eif2ak3	eukaryotic translation initiation factor 2 alpha kinase 3	ENSMUSG00000031668	60731	27,26
Cst3	cystatin C	ENSMUSG00000027447	3971	27,18
Ehd1	EH-domain containing 1	ENSMUSG00000024772	23372	27,18
Sep 05	septin 5	ENSMUSG00000072214	8128	27,18
Tpd52l1	tumor protein D52-like 1	ENSMUSG00000000296	113542	27,18
Aimp2	aminoacyl tRNA synthetase complex-interacting multifunctional protein 2	ENSMUSG00000029610	7144	27,18
Cndp2	CNDP dipeptidase 2 (metallopeptidase M20 family)	ENSMUSG00000024644	18164	27,18
Adprm	ADP-ribose/CDP-alcohol diphosphatase, manganese dependent	ENSMUSG00000020910	14690	27,18
Atad2	ATPase family, AAA domain containing 2	ENSMUSG00000022360	41036	27,18
		ENSMUSG00000033105	25551	27,18
Pcnxl3	pecanex-like 3 (Drosophila)	ENSMUSG00000054874	24274	27,18
Lamb2	laminin, beta 2	ENSMUSG00000052911	10669	27,18
Fbxl5	F-box and leucine-rich repeat protein 5	ENSMUSG00000039753	77024	27,18
Larp1b	La ribonucleoprotein domain family, member 1B	ENSMUSG00000037814	27163	27,18
Kdm4b	lysine (K)-specific demethylase 4B	ENSMUSG00000024201	76809	27,18
Insl6	insulin-like 6	ENSMUSG00000050957	4013	27,18
Mif	macrophage migration inhibitory factor	ENSMUSG00000033307	898	27,11
Hddc3	HD domain containing 3	ENSMUSG00000030532	2961	27,11
Napb	N-ethylmaleimide sensitive fusion protein attachment protein beta	ENSMUSG00000027438	38483	27,11
Ssna1	Sjogren's syndrome nuclear autoantigen 1	ENSMUSG00000026966	1400	27,11
Cpsf3l	cleavage and polyadenylation specific factor 3-like	ENSMUSG00000029034	19558	27,11
Pdcd5	programmed cell death 5	ENSMUSG00000030417	5540	27,11

Zfp653	zinc finger protein 653	ENSMUSG00000038895	15886	27,11
Bbc3	BCL2 binding component 3	ENSMUSG00000002083	8590	27,11
		ENSMUSG00000045319	30187	27,11
Rbm4b	RNA binding motif protein 4B	ENSMUSG00000033760	10523	27,11
Prtg	protogenin homolog (Gallus gallus)	ENSMUSG00000036030	110018	27,11
		ENSMUSG00000032475	53761	27,11
Fam173a	family with sequence similarity 173, member A	ENSMUSG00000057411	1777	27,04
Med11	mediator of RNA polymerase II transcription, subunit 11 homolog (S. cerevisiae)	ENSMUSG00000018923	1809	27,04
Itpkc	inositol 1,4,5-trisphosphate 3-kinase C	ENSMUSG00000003752	21457	27,04
Nudc	nuclear distribution gene C homolog (Aspergillus)	ENSMUSG00000028851	13455	27,04
Mageb18	melanoma antigen family B, 18	ENSMUSG00000067649	480694	27,04
Impact	imprinted and ancient	ENSMUSG00000024423	20697	27,04
Hdgfrp2	hepatoma-derived growth factor, related protein 2	ENSMUSG00000002833	20940	26,97
Aspm	asp (abnormal spindle)-like, microcephaly associated (Drosophila)	ENSMUSG00000033952	39318	26,97
Slc35e2	solute carrier family 35, member E2	ENSMUSG00000042202	21925	26,97
Rgs9	regulator of G-protein signaling 9	ENSMUSG00000020599	72775	26,97
Dopey2	dopey family member 2	ENSMUSG00000022946	98679	26,97
Cdkn1b	cyclin-dependent kinase inhibitor 1B	ENSMUSG00000003031	5113	26,89
Tpgs2	tubulin polyglutamylase complex subunit 2	ENSMUSG00000024269	41785	26,89
Hdac5	histone deacetylase 5	ENSMUSG00000008855	35735	26,89
Lrch4	leucine-rich repeats and calponin homology (CH) domain containing 4	ENSMUSG00000093445	11979	26,89
Urgcp	upregulator of cell proliferation	ENSMUSG00000049680	48960	26,89
Auh	AU RNA binding protein/enoyl-coenzyme A hydratase	ENSMUSG00000021460	94563	26,89
Zfp943	zinc finger prtoein 943	ENSMUSG00000053347	108617	26,89
Ggact	gamma-glutamylamine cyclotransferase	ENSMUSG00000041625	97535	26,89
Syngr1	synaptogyrin 1	ENSMUSG00000022415	28168	26,82
Ccdc38	coiled-coil domain containing 38	ENSMUSG00000036168	43696	26,82
		ENSMUSG00000026526	25262	26,82
B4galt3	UDP-Gal:betaGlcNAc beta 1,4-galactosyltransferase, polypeptide 3	ENSMUSG00000052423	6569	26,82
Lztr1	leucine-zipper-like transcriptional regulator, 1	ENSMUSG00000022761	17646	26,82
Frg1	FSHD region gene 1	ENSMUSG00000031590	19662	26,82
Elmod1	ELMO/CED-12 domain containing 1	ENSMUSG00000041986	63842	26,82
1110008P14Rik	RIKEN cDNA 1110008P14 gene	ENSMUSG00000039195	4842	26,82
Zfp606	zinc finger protein 606	ENSMUSG00000030386	17943	26,82
Acot7	acyl-CoA thioesterase 7	ENSMUSG00000028937	93722	26,82
Dclre1c	DNA cross-link repair 1C, PSO2 homolog (S. cerevisiae)	ENSMUSG00000026648	40000	26,82

Snx32	sorting nexin 32	ENSMUSG00000056185	15212	26,82
E2f7	E2F transcription factor 7	ENSMUSG00000020185	41946	26,82
		ENSMUSG00000020311	23562	26,82
Wdr95	WD40 repeat domain 95	ENSMUSG00000029658	83216	26,82
Kcng2	potassium voltage-gated channel, subfamily G, member 2	ENSMUSG00000059852	69711	26,82
Pop4	processing of precursor 4, ribonuclease P/MRP family, (S. cerevisiae)	ENSMUSG00000030423	8529	26,75
Prps1	phosphoribosyl pyrophosphate synthetase 1	ENSMUSG00000031432	19528	26,75
Pdzrn3	PDZ domain containing RING finger 3	ENSMUSG00000035357	228289	26,75
Coprs	coordinator of PRMT5, differentiation stimulator	ENSMUSG00000031458	5484	26,75
Cops8	COP9 (constitutive photomorphogenic) homolog, subunit 8 (Arabidopsis thaliana)	ENSMUSG00000034432	9917	26,75
Tfcp2	transcription factor CP2	ENSMUSG00000009733	49210	26,75
Ccdc28a	coiled-coil domain containing 28A	ENSMUSG00000059554	21323	26,75
Spg21	spastic paraplegia 21 homolog (human)	ENSMUSG00000032388	27534	26,75
Nox1	NADPH oxidase 1	ENSMUSG00000031257	135536	26,75
		ENSMUSG00000033454	26685	26,75
Zmym6	zinc finger, MYM-type 6	ENSMUSG00000042408	46990	26,75
Chrnb1	cholinergic receptor, nicotinic, beta polypeptide 1 (muscle)	ENSMUSG00000041189	11908	26,75
Fgf14	fibroblast growth factor 14	ENSMUSG00000025551	699221	26,75
Tmem139	transmembrane protein 139	ENSMUSG00000071506	2586	26,75
Ak2	adenylate kinase 2	ENSMUSG00000028792	19572	26,68
Uqcrc1	ubiquinol-cytochrome c reductase core protein 1	ENSMUSG00000025651	12993	26,68
Tekt1	tektin 1	ENSMUSG00000020799	17721	26,68
		ENSMUSG00000074825	4671	26,68
Prdm4	PR domain containing 4	ENSMUSG00000035529	24976	26,68
Esr2	estrogen receptor 2 (beta)	ENSMUSG00000021055	56841	26,68
Arhgap31	Rho GTPase activating protein 31	ENSMUSG00000022799	114935	26,68
Fbxo16	F-box protein 16	ENSMUSG00000034532	54801	26,68
1600029D21Rik	RIKEN cDNA 1600029D21 gene	ENSMUSG00000032068	11115	26,68
Mrpl27	mitochondrial ribosomal protein L27	ENSMUSG00000024414	6323	26,60
		ENSMUSG00000097148	75992	26,60
Pnp	purine-nucleoside phosphorylase	ENSMUSG00000021871	20936	26,60
Bhlhb9	basic helix-loop-helix domain containing, class B9	ENSMUSG00000072964	5231	26,60
Tfap2d	transcription factor AP-2, delta	ENSMUSG00000042596	63325	26,60
Slc25a53	solute carrier family 25, member 53	ENSMUSG00000044348	57187	26,60
Klhl26	kelch-like 26	ENSMUSG00000055707	26716	26,60
Rsph3a	radial spoke 3A homolog (Chlamydomonas)	ENSMUSG00000073471	34172	26,60
Cdc40	cell division cycle 40	ENSMUSG00000038446	51518	26,60
Trmt1l	tRNA methyltransferase 1 like	ENSMUSG00000053286	29535	26,60
Chst11	carbohydrate sulfotransferase 11	ENSMUSG00000034612	210403	26,60
Lonrf1	LON peptidase N-terminal domain and ring finger 1	ENSMUSG00000039633	33453	26,60
Actr3b	ARP3 actin-related protein 3B	ENSMUSG00000056367	90346	26,60

Efhb	EF hand domain family, member B	ENSMUSG00000023931	64433	26,60
Il4ra	interleukin 4 receptor, alpha	ENSMUSG00000030748	27191	26,60
Fam161a	family with sequence similarity 161, member A	ENSMUSG00000049811	23258	26,60
Adat1	adenosine deaminase, tRNA-specific 1	ENSMUSG00000031949	25395	26,60
Iqch	IQ motif containing H	ENSMUSG00000037801	181039	26,60
Sh3yl1	Sh3 domain YSC-like 1	ENSMUSG00000020669	48495	26,60
Upf1	UPF1 regulator of nonsense transcripts homolog (yeast)	ENSMUSG00000058301	21752	26,53
Fam222a	family with sequence similarity 222, member A	ENSMUSG00000041930	45205	26,53
Kri1	KRI1 homolog (S. cerevisiae)	ENSMUSG00000035047	14514	26,53
Nol11	nucleolar protein 11	ENSMUSG00000018433	22719	26,53
Zfp110	zinc finger protein 110	ENSMUSG00000058638	15817	26,53
Ptcd3	pentatricopeptide repeat domain 3	ENSMUSG00000063884	28115	26,53
Nfrkb	nuclear factor related to kappa B binding protein	ENSMUSG00000042185	35142	26,53
Pex13	peroxisomal biogenesis factor 13	ENSMUSG00000020283	19481	26,53
9530077C05Rik	RIKEN cDNA 9530077C05 gene	ENSMUSG00000036411	33101	26,53
Snrpf	small nuclear ribonucleoprotein polypeptide F	ENSMUSG00000020018	6678	26,46
		ENSMUSG00000020492	15402	26,46
Jmjd8	jumonji domain containing 8	ENSMUSG00000025736	2976	26,46
Cdk5rap3	CDK5 regulatory subunit associated protein 3	ENSMUSG00000018669	9071	26,46
		ENSMUSG00000057363	80835	26,46
Bysl	bystin-like	ENSMUSG00000023988	12162	26,46
Ddx19a	DEAD (Asp-Glu-Ala-Asp) box polypeptide 19a	ENSMUSG00000015023	22805	26,46
Yrdc	yrdC domain containing (E.coli)	ENSMUSG00000028889	4564	26,46
Slc6a9	solute carrier family 6 (neurotransmitter transporter, glycine), member 9	ENSMUSG00000028542	40693	26,46
		ENSMUSG00000053644	63265	26,46
Pprc1	peroxisome proliferative activated receptor, gamma, coactivator-related 1	ENSMUSG00000055491	28030	26,46
Mkks	McKusick-Kaufman syndrome	ENSMUSG00000027274	17610	26,46
Slc33a1	solute carrier family 33 (acetyl-CoA transporter), member 1	ENSMUSG00000027822	31262	26,46
Mcts1	malignant T cell amplified sequence 1	ENSMUSG00000000355	12849	26,46
Syap1	synapse associated protein 1	ENSMUSG00000031357	31391	26,46
Thsd7a	thrombospondin, type I, domain containing 7A	ENSMUSG00000032625	437801	26,46
Pld1	phospholipase D1	ENSMUSG00000027695	194668	26,46
Cdhr3	cadherin-related family member 3	ENSMUSG00000035860	59080	26,46
Srp54c	signal recognition particle 54C	ENSMUSG00000021020	23695	26,39
Akr1e1	aldo-keto reductase family 1, member E1	ENSMUSG00000045410	18418	26,39
Dhx38	DEAH (Asp-Glu-Ala-His) box polypeptide 38	ENSMUSG00000037993	17585	26,39
Ocel1	occludin/ELL domain containing 1	ENSMUSG00000002396	8064	26,39
Necap2	NECAP endocytosis associated 2	ENSMUSG00000028923	11846	26,39
		ENSMUSG00000001998	59193	26,39

Atg4c	autophagy related 4C, cysteine peptidase	ENSMUSG00000028550	65854	26,39
Fsd1l	fibronectin type III and SPRY domain containing 1-like	ENSMUSG00000054752	75539	26,39
Gm608	predicted gene 608	ENSMUSG00000068284	54220	26,39
Elf3	E74-like factor 3	ENSMUSG00000003051	4899	26,39
Bhlhe40	basic helix-loop-helix family, member e40	ENSMUSG00000030103	6297	26,39
Smn1	survival motor neuron 1	ENSMUSG00000021645	12839	26,31
Cks1b	CDC28 protein kinase 1b	ENSMUSG00000028044	2912	26,31
Eif1ad	eukaryotic translation initiation factor 1A domain containing	ENSMUSG00000024841	4699	26,31
Sdr39u1	short chain dehydrogenase/reductase family 39U, member 1	ENSMUSG00000022223	2947	26,31
Akap1	A kinase (PRKA) anchor protein 1	ENSMUSG00000018428	33795	26,31
Ccdc96	coiled-coil domain containing 96	ENSMUSG00000050677	3584	26,31
Fam220a	family with sequence similarity 220, member A	ENSMUSG00000048910	15820	26,31
Ccdc174	coiled-coil domain containing 174	ENSMUSG00000034083	21791	26,31
Bin1	bridging integrator 1	ENSMUSG00000024381	58520	26,31
Tep1	telomerase associated protein 1	ENSMUSG00000006281	46499	26,31
Gigyf1	GRB10 interacting GYF protein 1	ENSMUSG00000029714	9055	26,31
Lin54	lin-54 homolog (C. elegans)	ENSMUSG00000035310	58722	26,31
Lrrc6	leucine rich repeat containing 6 (testis)	ENSMUSG00000022375	121053	26,31
Scnn1a	sodium channel, nonvoltage-gated 1 alpha	ENSMUSG00000030340	24285	26,31
Acat2	acetyl-Coenzyme A acetyltransferase 2	ENSMUSG00000023832	17858	26,24
		ENSMUSG00000039737	20837	26,24
Hist1h2af	histone cluster 1, H2af	ENSMUSG00000061991	399	26,24
Mras	muscle and microspikes RAS	ENSMUSG00000032470	51460	26,24
Adamts17	a disintegrin-like and metallopeptidase (reprolysin type) with thrombospondin type 1 motif, 17	ENSMUSG00000058145	312891	26,24
Pik3ip1	phosphoinositide-3-kinase interacting protein 1	ENSMUSG00000034614	12571	26,24
Mpp2	membrane protein, palmitoylated 2 (MAGUK p55 subfamily member 2)	ENSMUSG00000017314	31501	26,24
Ccl25	chemokine (C-C motif) ligand 25	ENSMUSG00000023235	34811	26,24
Slc5a1	solute carrier family 5 (sodium/glucose cotransporter), member 1	ENSMUSG00000011034	58654	26,24
Pcsk2	proprotein convertase subtilisin/kexin type 2	ENSMUSG00000027419	270130	26,24
Arl6ip5	ADP-ribosylation factor-like 6 interacting protein 5	ENSMUSG00000035199	22627	26,24
Cmc2	COX assembly mitochondrial protein 2	ENSMUSG00000014633	32771	26,17
Ufsp2	UFM1-specific peptidase 2	ENSMUSG00000031634	21431	26,17
Srprb	signal recognition particle receptor, B subunit	ENSMUSG00000032553	14105	26,17
		ENSMUSG00000043866	4969	26,17

Arhgef9	CDC42 guanine nucleotide exchange factor (GEF) 9	ENSMUSG00000025656	147887	26,17
Cbll1	Casitas B-lineage lymphoma-like 1	ENSMUSG00000020659	14788	26,17
Ccdc25	coiled-coil domain containing 25	ENSMUSG00000022035	29303	26,17
Ccrn4l	CCR4 carbon catabolite repression 4-like (S. cerevisiae)	ENSMUSG00000023087	27198	26,17
Uckl1	uridine-cytidine kinase 1-like 1	ENSMUSG00000089917	15744	26,10
		ENSMUSG00000036853	24348	26,10
Dnm1	dynamin 1	ENSMUSG00000026825	44859	26,10
		ENSMUSG00000055435	24853	26,10
Tbc1d17	TBC1 domain family, member 17	ENSMUSG00000038520	8179	26,10
Tmem259	transmembrane protein 259	ENSMUSG00000013858	9383	26,10
Fam132a	family with sequence similarity 132, member A	ENSMUSG00000023571	4312	26,10
Pcbp4	poly(rC) binding protein 4	ENSMUSG00000023495	10170	26,10
Wbscr16	Williams-Beuren syndrome chromosome region 16 homolog (human)	ENSMUSG00000061979	28721	26,10
Alg9	asparagine-linked glycosylation 9 (alpha 1,2 mannosyltransferase)	ENSMUSG00000032059	68524	26,10
Rad18	RAD18 homolog (S. cerevisiae)	ENSMUSG00000030254	76837	26,10
Pqlc3	PQ loop repeat containing	ENSMUSG00000045679	11761	26,10
Mertk	c-mer proto-oncogene tyrosine kinase	ENSMUSG00000014361	103939	26,10
Spata17	spermatogenesis associated 17	ENSMUSG00000026611	167040	26,10
Rab3b	RAB3B, member RAS oncogene family	ENSMUSG00000003411	64262	26,02
Ndst1	N-deacetylase/N-sulfotransferase (heparan glucosaminyl) 1	ENSMUSG00000054008	27412	26,02
Ppm1e	protein phosphatase 1E (PP2C domain containing)	ENSMUSG00000046442	132118	26,02
D430019H16Rik	RIKEN cDNA D430019H16 gene	ENSMUSG00000094910	39240	26,02
Wdr11	WD repeat domain 11	ENSMUSG00000042055	43876	26,02
Comt	catechol-O-methyltransferase	ENSMUSG00000000326	19967	26,02
Rft1	RFT1 homolog (S. cerevisiae)	ENSMUSG00000052395	36958	26,02
Polr1a	polymerase (RNA) I polypeptide A	ENSMUSG00000049553	70304	26,02
Hibadh	3-hydroxyisobutyrate dehydrogenase	ENSMUSG00000029776	94162	26,02
Plek	pleckstrin	ENSMUSG00000020120	81176	26,02
Rorc	RAR-related orphan receptor gamma	ENSMUSG00000028150	25483	26,02
2810417H13Rik	RIKEN cDNA 2810417H13 gene	ENSMUSG00000040204	13030	25,95
Ttl	tubulin tyrosine ligase	ENSMUSG00000027394	30342	25,95
		ENSMUSG00000059355	1600	25,95
Rab25	RAB25, member RAS oncogene family	ENSMUSG00000008601	6272	25,95
Vasp	vasodilator-stimulated phosphoprotein	ENSMUSG00000030403	14889	25,95
Wdr75	WD repeat domain 75	ENSMUSG00000025995	28454	25,95
		ENSMUSG00000001569	18843	25,95
Agbl5	ATP/GTP binding protein-like 5	ENSMUSG00000029165	18272	25,95
Mrgbp	MRG/MORF4L binding protein	ENSMUSG00000027569	5001	25,95
Prkar2b	protein kinase, cAMP dependent regulatory, type II beta	ENSMUSG00000002997	102821	25,95
Kdm4a	lysine (K)-specific demethylase 4A	ENSMUSG00000033326	43087	25,95
Tmem181a	transmembrane protein 181A	ENSMUSG00000038141	35309	25,95

Traf3ip1	TRAF3 interacting protein 1	ENSMUSG00000034292	34636	25,95
Nrbp1	nuclear receptor binding protein 1	ENSMUSG00000029148	10703	25,95
Anapc10	anaphase promoting complex subunit 10	ENSMUSG00000036977	65502	25,95
Zgpat	zinc finger, CCCH-type with G patch domain	ENSMUSG00000027582	18701	25,95
Prkab2	protein kinase, AMP-activated, beta 2 non-catalytic subunit	ENSMUSG00000038205	15620	25,95
Polg2	polymerase (DNA directed), gamma 2, accessory subunit	ENSMUSG00000020718	11285	25,95
Foxa1	forkhead box A1	ENSMUSG00000035451	5491	25,95
Impdh1	inosine 5'-phosphate dehydrogenase 1	ENSMUSG00000003500	15931	25,88
Fads1	fatty acid desaturase 1	ENSMUSG00000010663	13983	25,88
		ENSMUSG00000095427	666	25,88
Mrpl36	mitochondrial ribosomal protein L36	ENSMUSG00000021607	1167	25,88
Arhgap4	Rho GTPase activating protein 4	ENSMUSG00000031389	30429	25,88
Acot13	acyl-CoA thioesterase 13	ENSMUSG00000006717	13570	25,88
Stard3nl	STARD3 N-terminal like	ENSMUSG00000003062	38076	25,88
D3Ertd254e	DNA segment, Chr 3, ERATO Doi 254, expressed	ENSMUSG00000033883	10814	25,88
Golim4	golgi integral membrane protein 4	ENSMUSG00000034109	80767	25,88
Slc28a3	solute carrier family 28 (sodium-coupled nucleoside transporter), member 3	ENSMUSG00000021553	65479	25,88
Il1rapl2	interleukin 1 receptor accessory protein-like 2	ENSMUSG00000059203	1276339	25,88
Rhbdl2	rhomboid, veinlet-like 2 (Drosophila)	ENSMUSG00000043333	42031	25,88
Fkbp9	FK506 binding protein 9	ENSMUSG00000029781	47300	25,81
B2m	beta-2 microglobulin	ENSMUSG00000060802	5398	25,81
Phex	phosphate regulating gene with homologies to endopeptidases on the X chromosome (hypophosphatemia, vitamin D resistant rickets)	ENSMUSG00000057457	253238	25,81
Sepp1	selenoprotein P, plasma, 1	ENSMUSG00000064373	11962	25,73
Snrpa1	small nuclear ribonucleoprotein polypeptide A'	ENSMUSG00000030512	14284	25,73
C2	complement component 2 (within H-2S)	ENSMUSG00000024371	41854	25,73
Pcgf2	polycomb group ring finger 2	ENSMUSG00000018537	11675	25,73
Trp53inp1	transformation related protein 53 inducible nuclear protein 1	ENSMUSG00000028211	17949	25,73
Wrap53	WD repeat containing, antisense to Trp53	ENSMUSG00000041346	18498	25,73
Taok2	TAO kinase 2	ENSMUSG00000059981	19026	25,73
Ino80c	INO80 complex subunit C	ENSMUSG00000047989	17197	25,73
Rcn2	reticulocalbin 2	ENSMUSG00000032320	20038	25,73
Fam213b	family with sequence similarity 213, member B	ENSMUSG00000029059	3632	25,66
Chchd1	coiled-coil-helix-coiled-coil-helix domain containing 1	ENSMUSG00000063787	1391	25,66
Vps72	vacuolar protein sorting 72 (yeast)	ENSMUSG00000008958	12030	25,66
Zfp775	zinc finger protein 775	ENSMUSG00000007216	10048	25,66

Stx2	syntaxin 2	ENSMUSG00000029428	24018	25,66
Zfp524	zinc finger protein 524	ENSMUSG00000051184	2986	25,66
Tctn2	tectonic family member 2	ENSMUSG00000029386	28990	25,66
Prkra	protein kinase, interferon inducible double stranded RNA dependent activator	ENSMUSG00000002731	18118	25,66
Las1l	LAS1-like (S. cerevisiae)	ENSMUSG00000057421	21628	25,66
Wdr44	WD repeat domain 44	ENSMUSG00000036769	112975	25,66
Man2a1	mannosidase 2, alpha 1	ENSMUSG00000024085	154375	25,66
Ero1lb	ERO1-like beta (S. cerevisiae)	ENSMUSG00000057069	43644	25,66
Tmprss13	transmembrane protease, serine 13	ENSMUSG00000037129	28482	25,66
Zfp46	zinc finger protein 46	ENSMUSG00000051351	9260	25,59
Vps26a	vacuolar protein sorting 26 homolog A (yeast)	ENSMUSG00000020078	31963	25,59
Mrpl11	mitochondrial ribosomal protein L11	ENSMUSG00000024902	4690	25,59
Slc35b2	solute carrier family 35, member B2	ENSMUSG00000037089	3706	25,59
N6amt1	N-6 adenine-specific DNA methyltransferase 1 (putative)	ENSMUSG00000044442	14558	25,59
Efcab4a	EF-hand calcium binding domain 4A	ENSMUSG00000048200	5520	25,59
Trmt2b	TRM2 tRNA methyltransferase 2B	ENSMUSG00000067369	54646	25,59
Itga2b	integrin alpha 2b	ENSMUSG00000034664	16826	25,59
Gadd45g	growth arrest and DNA-damage-inducible 45 gamma	ENSMUSG00000021453	1791	25,52
Pmpcb	peptidase (mitochondrial processing) beta	ENSMUSG00000029017	20012	25,52
Slc22a27	solute carrier family 22, member 27	ENSMUSG00000067656	101640	25,52
Oxa1l	oxidase assembly 1-like	ENSMUSG00000000959	8829	25,52
		ENSMUSG00000027774	46434	25,52
4931406C07Rik	RIKEN cDNA 4931406C07 gene	ENSMUSG00000031938	23112	25,52
Brsk1	BR serine/threonine kinase 1	ENSMUSG00000035390	25394	25,52
Rnf41	ring finger protein 41	ENSMUSG00000025373	29823	25,52
Foxo4	forkhead box O4	ENSMUSG00000042903	6346	25,52
A230046K03Rik	RIKEN cDNA A230046K03 gene	ENSMUSG00000034560	52529	25,52
Dnah10	dynein, axonemal, heavy chain 10	ENSMUSG00000038011	109224	25,52
Prkd2	protein kinase D2	ENSMUSG00000041187	27560	25,44
Rap1gap	Rap1 GTPase-activating protein	ENSMUSG00000041351	65136	25,44
Lancl1	LanC (bacterial lantibiotic synthetase component C)-like 1	ENSMUSG00000026000	38356	25,44
Filip1	filamin A interacting protein 1	ENSMUSG00000034898	162754	25,44
Oprk1	opioid receptor, kappa 1	ENSMUSG00000025905	17639	25,44
Spcs1	signal peptidase complex subunit 1 homolog (S. cerevisiae)	ENSMUSG00000021917	1847	25,37
Chsy1	chondroitin sulfate synthase 1	ENSMUSG00000032640	64275	25,37
Traf2	TNF receptor-associated factor 2	ENSMUSG00000026942	28959	25,37
Dmtn	dematin actin binding protein	ENSMUSG00000022099	33865	25,37
Pradc1	protease-associated domain containing 1	ENSMUSG00000030008	5161	25,37
Sh3pxd2b	SH3 and PX domains 2B	ENSMUSG00000040711	80354	25,37
Pus7	pseudouridylate synthase 7 homolog (S. cerevisiae)	ENSMUSG00000057541	43064	25,37
Sirt7	sirtuin 7	ENSMUSG00000025138	6869	25,37
		ENSMUSG00000023039	19271	25,37

Slc18a2	solute carrier family 18 (vesicular monoamine), member 2	ENSMUSG00000025094	35135	25,37
		ENSMUSG00000027694	1828	25,30
Alg2	asparagine-linked glycosylation 2 (alpha-1,3-mannosyltransferase)	ENSMUSG00000039740	9267	25,30
Lamtor3	late endosomal/lysosomal adaptor, MAPK and MTOR activator 3	ENSMUSG00000091512	10240	25,30
Efemp2	epidermal growth factor-containing fibulin-like extracellular matrix protein 2	ENSMUSG00000024909	7881	25,30
Rrm1	ribonucleotide reductase M1	ENSMUSG00000030978	28077	25,30
Med17	mediator complex subunit 17	ENSMUSG00000031935	19516	25,30
Rilpl1	Rab interacting lysosomal protein-like 1	ENSMUSG00000029392	38310	25,30
Inpp5k	inositol polyphosphate 5-phosphatase K	ENSMUSG00000006127	17884	25,30
Ddx18	DEAD (Asp-Glu-Ala-Asp) box polypeptide 18	ENSMUSG00000001674	14155	25,30
Dfna5	deafness, autosomal dominant 5 (human)	ENSMUSG00000029821	74494	25,30
Sync	syncoilin	ENSMUSG00000001333	20943	25,30
Gdap2	ganglioside-induced differentiation-associated-protein 2	ENSMUSG00000027865	44601	25,30
		ENSMUSG00000020246	46269	25,30
Ppp2r3c	protein phosphatase 2, regulatory subunit B'', gamma	ENSMUSG00000021022	22187	25,30
Il12rb2	interleukin 12 receptor, beta 2	ENSMUSG00000018341	84871	25,30
Tnk1	tyrosine kinase, non-receptor, 1	ENSMUSG00000001583	7726	25,30
AI836003	expressed sequence AI836003	ENSMUSG00000029875	2329	25,23
Gpank1	G patch domain and ankyrin repeats 1	ENSMUSG00000092417	3360	25,23
Atp8b2	ATPase, class I, type 8B, member 2	ENSMUSG00000060671	24028	25,23
Tmem167b	transmembrane protein 167B	ENSMUSG00000068732	6042	25,23
Ccl27a	chemokine (C-C motif) ligand 27A	ENSMUSG00000073888	4781	25,23
Slc7a5	solute carrier family 7 (cationic amino acid transporter, y+ system), member 5	ENSMUSG00000040010	26545	25,23
Mtmr6	myotubularin related protein 6	ENSMUSG00000021987	37166	25,23
Foxp4	forkhead box P4	ENSMUSG00000023991	57513	25,23
Slc25a28	solute carrier family 25, member 28	ENSMUSG00000040414	11060	25,23
Ogfr	opioid growth factor receptor	ENSMUSG00000049401	6592	25,23
Sh2d3c	SH2 domain containing 3C	ENSMUSG00000059013	34458	25,23
Mtg2	mitochondrial ribosome associated GTPase 2	ENSMUSG00000039069	15315	25,23
Acox3	acyl-Coenzyme A oxidase 3, pristanoyl	ENSMUSG00000029098	30762	25,23
Syt12	synaptotagmin XII	ENSMUSG00000049303	31540	25,23
Amy1	amylase 1, salivary	ENSMUSG00000074264	50990	25,23
Tlcd2	TLC domain containing 2	ENSMUSG00000038217	9185	25,23
Ogfod1	2-oxoglutarate and iron-dependent oxygenase domain containing 1	ENSMUSG00000033009	30724	25,23
Nr1d1	nuclear receptor subfamily 1, group D, member 1	ENSMUSG00000020889	7402	25,15
Zdhhc4	zinc finger, DHHC domain containing 4	ENSMUSG00000001844	12768	25,15

Coq2	coenzyme Q2 homolog, prenyltransferase (yeast)	ENSMUSG00000029319	20418	25,15
Mrpl49	mitochondrial ribosomal protein L49	ENSMUSG00000007338	4122	25,15
Fam211a	family with sequence similarity 211, member A	ENSMUSG00000046417	43640	25,15
Ndufv2	NADH dehydrogenase (ubiquinone) flavoprotein 2	ENSMUSG00000024099	22765	25,15
Acsl5	acyl-CoA synthetase long-chain family member 5	ENSMUSG00000024981	43259	25,15
Ppp4c	protein phosphatase 4, catalytic subunit	ENSMUSG00000030697	6565	25,15
		ENSMUSG00000033319	24226	25,15
Sdad1	SDA1 domain containing 1	ENSMUSG00000029415	26015	25,15
Muc20	mucin 20	ENSMUSG00000035638	20017	25,15
Sh3rf2	SH3 domain containing ring finger 2	ENSMUSG00000057719	105294	25,15
Zfp428	zinc finger protein 428	ENSMUSG00000064264	8677	25,08
Chmp7	charged multivesicular body protein 7	ENSMUSG00000034190	15592	25,08
Armcx1	armadillo repeat containing, X-linked 1	ENSMUSG00000033460	3955	25,08
Acat1	acetyl-Coenzyme A acetyltransferase 1	ENSMUSG00000032047	29861	25,08
Nosip	nitric oxide synthase interacting protein	ENSMUSG00000003421	16075	25,08
Vps16	vacuolar protein sorting 16 (yeast)	ENSMUSG00000027411	19931	25,08
Wasf1	WAS protein family, member 1	ENSMUSG00000019831	55096	25,08
Zbtb41	zinc finger and BTB domain containing 41 homolog	ENSMUSG00000033964	30623	25,08
2310003H01Rik	RIKEN cDNA 2310003H01 gene	ENSMUSG00000025384	9710	25,08
Klhl18	kelch-like 18	ENSMUSG00000054792	50767	25,08
		ENSMUSG00000031841	1041189	25,08
Mrps31	mitochondrial ribosomal protein S31	ENSMUSG00000031533	18326	25,01
Carns1	carnosine synthase 1	ENSMUSG00000075289	11156	25,01
		ENSMUSG00000097064	11156	25,01
Eif3i	eukaryotic translation initiation factor 3, subunit I	ENSMUSG00000028798	8689	25,01
Dedd2	death effector domain-containing DNA binding protein 2	ENSMUSG00000054499	17020	25,01
Mkrn2	makorin, ring finger protein, 2	ENSMUSG00000000439	16733	25,01
Taf2	TAF2 RNA polymerase II, TATA box binding protein (TBP)-associated factor	ENSMUSG00000037343	57024	25,01
Terf1	telomeric repeat binding factor 1	ENSMUSG00000025925	37771	25,01
		ENSMUSG00000063568	299835	25,01
Lactb	lactamase, beta	ENSMUSG00000032370	20092	24,94
		ENSMUSG00000058267	6956	24,94
Rdh11	retinol dehydrogenase 11	ENSMUSG00000066441	17957	24,94
9030025P20Rik	RIKEN cDNA 9030025P20 gene	ENSMUSG00000073455	12714	24,94
Smap2	small ArfGAP 2	ENSMUSG00000032870	48931	24,94
Cdc42ep5	CDC42 effector protein (Rho GTPase binding) 5	ENSMUSG00000063838	13601	24,94
Bcl9l	B cell CLL/lymphoma 9-like	ENSMUSG00000063382	11253	24,94

Ppcdc	phosphopantothenoylcysteine decarboxylase	ENSMUSG00000063849	27447	24,94
Tex264	testis expressed gene 264	ENSMUSG00000040813	27202	24,94
Ano4	anoctamin 4	ENSMUSG00000035189	395769	24,94
		ENSMUSG00000035372	9549	24,94
Tnip1	TNFAIP3 interacting protein 1	ENSMUSG00000020400	52133	24,94
Ptpn21	protein tyrosine phosphatase, non-receptor type 21	ENSMUSG00000021009	60665	24,94
Szt2	seizure threshold 2	ENSMUSG00000033253	46531	24,94
Kcnj5	potassium inwardly-rectifying channel, subfamily J, member 5	ENSMUSG00000032034	29455	24,94
Slc6a14	solute carrier family 6 (neurotransmitter transporter), member 14	ENSMUSG00000031089	27460	24,94
Ube2s	ubiquitin-conjugating enzyme E2S	ENSMUSG00000060860	4197	24,86
Lrrc42	leucine rich repeat containing 42	ENSMUSG00000028617	20019	24,86
Crat	carnitine acetyltransferase	ENSMUSG00000026853	15343	24,86
Gtpbp2	GTP binding protein 2	ENSMUSG00000023952	8339	24,86
Mrpl19	mitochondrial ribosomal protein L19	ENSMUSG00000030045	8108	24,86
Zbtb14	zinc finger and BTB domain containing 14	ENSMUSG00000049672	7701	24,86
Ccdc167	coiled-coil domain containing 167	ENSMUSG00000024018	27635	24,86
Trabd	TraB domain containing	ENSMUSG00000015363	11954	24,86
Aph1a	anterior pharynx defective 1a homolog (C. elegans)	ENSMUSG00000015750	4320	24,86
Phgdh	3-phosphoglycerate dehydrogenase	ENSMUSG00000053398	26821	24,86
Rnf2	ring finger protein 2	ENSMUSG00000026484	31418	24,86
Isoc2a	isochorismatase domain containing 2a	ENSMUSG00000086784	18665	24,86
2410127L17Rik	RIKEN cDNA 2410127L17 gene	ENSMUSG00000024726	34009	24,86
Prkaa2	protein kinase, AMP-activated, alpha 2 catalytic subunit	ENSMUSG00000028518	80017	24,86
Kank1	KN motif and ankyrin repeat domains 1	ENSMUSG00000032702	197522	24,86
Pnmal1	PNMA-like 1	ENSMUSG00000041141	2525	24,79
Lemd2	LEM domain containing 2	ENSMUSG00000044857	14838	24,79
Eif4a3	eukaryotic translation initiation factor 4A3	ENSMUSG00000025580	11727	24,79
Trp53	transformation related protein 53	ENSMUSG00000059552	11515	24,79
Epn3	epsin 3	ENSMUSG00000010080	10376	24,79
Acot8	acyl-CoA thioesterase 8	ENSMUSG00000017307	12115	24,79
Eri1	exoribonuclease 1	ENSMUSG00000031527	30269	24,79
		ENSMUSG00000073158	52268	24,79
Chtf18	CTF18, chromosome transmission fidelity factor 18	ENSMUSG00000019214	8494	24,72
Sema5a	sema domain, seven thrombospondin repeats (type 1 and type 1-like), transmembrane domain (TM) and short cytoplasmic domain, (semaphorin) 5A	ENSMUSG00000022231	451529	24,72
Timm9	translocase of inner mitochondrial membrane 9	ENSMUSG00000021079	13503	24,72
Cdc42ep3	CDC42 effector protein (Rho GTPase binding) 3	ENSMUSG00000036533	21067	24,72

Grpel1	GrpE-like 1, mitochondrial	ENSMUSG00000029198	9083	24,72
Me2	malic enzyme 2, NAD(+)-dependent, mitochondrial	ENSMUSG00000024556	45353	24,72
Thap2	THAP domain containing, apoptosis associated protein 2	ENSMUSG00000020137	14470	24,72
Rnaseh2b	ribonuclease H2, subunit B	ENSMUSG00000021932	80404	24,72
Rnft2	ring finger protein, transmembrane 2	ENSMUSG00000032850	54381	24,72
Rilpl2	Rab interacting lysosomal protein-like 2	ENSMUSG00000029401	15102	24,72
Mospd2	motile sperm domain containing 2	ENSMUSG00000061778	44207	24,72
Nrg3	neuregulin 3	ENSMUSG00000041014	1104137	24,72
Zfp438	zinc finger protein 438	ENSMUSG00000050945	124409	24,72
Igfbp2	insulin-like growth factor binding protein 2	ENSMUSG00000039323	27972	24,65
Sema3d	sema domain, immunoglobulin domain (Ig), short basic domain, secreted, (semaphorin) 3D	ENSMUSG00000040254	205778	24,65
Scyl1	SCY1-like 1 (S. cerevisiae)	ENSMUSG00000024941	12975	24,65
Pcna	proliferating cell nuclear antigen	ENSMUSG00000027342	4153	24,65
Plekhh3	pleckstrin homology domain containing, family H (with MyTH4 domain) member 3	ENSMUSG00000035172	8673	24,65
Mrpl35	mitochondrial ribosomal protein L35	ENSMUSG00000052962	10808	24,65
Zfp61	zinc finger protein 61	ENSMUSG00000050605	10194	24,65
Ormdl2	ORM1-like 2 (S. cerevisiae)	ENSMUSG00000025353	4175	24,65
Mycl	v-myc myelocytomatosis viral oncogene homolog, lung carcinoma derived (avian)	ENSMUSG00000028654	6834	24,65
Fra10ac1	FRA10AC1 homolog (human)	ENSMUSG00000054237	35658	24,65
Tmem246	transmembrane protein 246	ENSMUSG00000039611	13371	24,65
Slc29a3	solute carrier family 29 (nucleoside transporters), member 3	ENSMUSG00000020100	40723	24,65
Sympk	symplekin	ENSMUSG00000023118	30242	24,65
Bcap29	B cell receptor associated protein 29	ENSMUSG00000020650	39305	24,65
Prrc2a	proline-rich coiled-coil 2A	ENSMUSG00000024393	15822	24,65
Polr1c	polymerase (RNA) I polypeptide C	ENSMUSG00000067148	4135	24,65
Athl1	ATH1, acid trehalase-like 1 (yeast)	ENSMUSG00000062031	6274	24,65
Unkl	unkempt-like (Drosophila)	ENSMUSG00000015127	46047	24,65
Micall2	MICAL-like 2	ENSMUSG00000036718	29644	24,65
Fam118b	family with sequence similarity 118, member B	ENSMUSG00000050471	50841	24,65
Samd12	sterile alpha motif domain containing 12	ENSMUSG00000058656	440917	24,65
		ENSMUSG00000073940	1473	24,57
Hras1	Harvey rat sarcoma virus oncogene 1	ENSMUSG00000025499	4901	24,57
Asna1	arsA arsenite transporter, ATP-binding, homolog 1 (bacterial)	ENSMUSG00000052456	7345	24,57
Wdtc1	WD and tetratricopeptide repeats 1	ENSMUSG00000037622	61023	24,57
		ENSMUSG00000038349	345415	24,57
Tub	tubby candidate gene	ENSMUSG00000031028	23636	24,57
Fam84b	family with sequence similarity 84, member B	ENSMUSG00000072568	6085	24,57

		ENSMUSG00000028246	65511	24,57
Dgkq	diacylglycerol kinase, theta	ENSMUSG00000004815	22980	24,57
Ap3s1	adaptor-related protein complex 3, sigma 1 subunit	ENSMUSG00000024480	48910	24,57
Slc1a3	solute carrier family 1 (glial high affinity glutamate transporter), member 3	ENSMUSG00000005360	76641	24,57
Cnnm2	cyclin M2	ENSMUSG00000064105	117200	24,57
Arhgap22	Rho GTPase activating protein 22	ENSMUSG00000063506	155909	24,57
Socs3	suppressor of cytokine signaling 3	ENSMUSG00000053113	3969	24,57
Deb1	differentially expressed in B16F10 1	ENSMUSG00000032526	2533	24,50
Mmgt1	membrane magnesium transporter 1	ENSMUSG00000061273	12558	24,50
Tars2	threonyl-tRNA synthetase 2, mitochondrial (putative)	ENSMUSG00000028107	15002	24,50
Vps28	vacuolar protein sorting 28 (yeast)	ENSMUSG00000062381	3940	24,50
		ENSMUSG00000031864	35482	24,50
Nol8	nucleolar protein 8	ENSMUSG00000021392	25666	24,50
Snf8	SNF8, ESCRT-II complex subunit, homolog (S. cerevisiae)	ENSMUSG00000006058	12546	24,50
Bend5	BEN domain containing 5	ENSMUSG00000028545	45293	24,50
Zbtb37	zinc finger and BTB domain containing 37	ENSMUSG00000043467	26056	24,50
Ube2j1	ubiquitin-conjugating enzyme E2J 1	ENSMUSG00000028277	20948	24,50
Rnf19b	ring finger protein 19B	ENSMUSG00000028793	27616	24,50
Nalcn	sodium leak channel, non-selective	ENSMUSG00000000197	350504	24,50
		ENSMUSG00000054737	33378	24,50
Kcnq5	potassium voltage-gated channel, subfamily Q, member 5	ENSMUSG00000028033	563540	24,43
Adad1	adenosine deaminase domain containing 1 (testis specific)	ENSMUSG00000027719	58398	24,43
Fam179a	family with sequence similarity 179, member A	ENSMUSG00000045761	56409	24,43
Ptrhd1	peptidyl-tRNA hydrolase domain containing 1	ENSMUSG00000096199	6097	24,43
		ENSMUSG00000025001	40137	24,43
Nudcd1	NudC domain containing 1	ENSMUSG00000038736	53081	24,43
Golph3l	golgi phosphoprotein 3-like	ENSMUSG00000046519	30314	24,43
Dhx8	DEAH (Asp-Glu-Ala-His) box polypeptide 8	ENSMUSG00000034931	34440	24,43
1810019J16Rik	RIKEN cDNA 1810019J16 gene	ENSMUSG00000037600	11828	24,43
Tmem213	transmembrane protein 213	ENSMUSG00000029829	6454	24,43
Cxcr4	chemokine (C-X-C motif) receptor 4	ENSMUSG00000045382	4095	24,36
Parp11	poly (ADP-ribose) polymerase family, member 11	ENSMUSG00000037997	47402	24,36
		ENSMUSG00000020973	9343	24,36
Slc19a1	solute carrier family 19 (folate transporter), member 1	ENSMUSG00000001436	28762	24,36
Gm9774	predicted pseudogene 9774	ENSMUSG00000042165	1358	24,36
0610009D07Rik	RIKEN cDNA 0610009D07 gene	ENSMUSG00000037361	10051	24,36
		ENSMUSG00000004934	14658	24,36
Cwc22	CWC22 spliceosome-associated protein homolog (S. cerevisiae)	ENSMUSG00000027014	65217	24,36

Szrd1	SUZ RNA binding domain containing 1	ENSMUSG00000040842	26775	24,36
Slc7a6os	solute carrier family 7, member 6 opposite strand	ENSMUSG00000033106	10496	24,36
Prim2	DNA primase, p58 subunit	ENSMUSG00000026134	215976	24,36
Sgcz	sarcoglycan zeta	ENSMUSG00000039539	1139211	24,36
Pglyrp1	peptidoglycan recognition protein 1	ENSMUSG00000030413	5749	24,36
Mrpl32	mitochondrial ribosomal protein L32	ENSMUSG00000015672	2737	24,28
		ENSMUSG00000083012	15824	24,28
Champ1	chromosome alignment maintaining phosphoprotein 1	ENSMUSG00000047710	11999	24,28
Pkp3	plakophilin 3	ENSMUSG00000054065	12293	24,28
Sema3f	sema domain, immunoglobulin domain (Ig), short basic domain, secreted, (semaphorin) 3F	ENSMUSG00000034684	28974	24,28
Per1	period circadian clock 1	ENSMUSG00000020893	14744	24,28
Cux2	cut-like homeobox 2	ENSMUSG00000042589	192140	24,28
E2f5	E2F transcription factor 5	ENSMUSG00000027552	27638	24,28
Fmn1	formin 1	ENSMUSG00000044042	389032	24,28
Hs6st2	heparan sulfate 6-O-sulfotransferase 2	ENSMUSG00000062184	294645	24,28
Fxyd6	FXYD domain-containing ion transport regulator 6	ENSMUSG00000066705	25974	24,21
Serinc2	serine incorporator 2	ENSMUSG00000023232	25711	24,21
Tesk1	testis specific protein kinase 1	ENSMUSG00000028458	6126	24,21
Sec61g	SEC61, gamma subunit	ENSMUSG00000078974	7955	24,21
Atp2b4	ATPase, Ca++ transporting, plasma membrane 4	ENSMUSG00000026463	98368	24,21
Ppme1	protein phosphatase methylesterase 1	ENSMUSG00000030718	45160	24,21
Dvl1	dishevelled, dsh homolog 1 (Drosophila)	ENSMUSG00000029071	11902	24,21
Gpatch1	G patch domain containing 1	ENSMUSG00000063808	41905	24,21
Fam21	family with sequence similarity 21	ENSMUSG00000024104	54636	24,21
Zw10	zw10 kinetochore protein	ENSMUSG00000032264	23192	24,21
Ppwd1	peptidylprolyl isomerase domain and WD repeat containing 1	ENSMUSG00000021713	23724	24,21
2610008E11Rik	RIKEN cDNA 2610008E11 gene	ENSMUSG00000060301	33227	24,21
Mrs2	MRS2 magnesium homeostasis factor homolog (S. cerevisiae)	ENSMUSG00000021339	27897	24,21
Tprg	transformation related protein 63 regulated	ENSMUSG00000048399	135528	24,21
Lysmd4	LysM, putative peptidoglycan-binding, domain containing 4	ENSMUSG00000043831	5925	24,14
Arl6ip6	ADP-ribosylation factor-like 6 interacting protein 6	ENSMUSG00000026960	27495	24,14
Akt1s1	AKT1 substrate 1 (proline-rich)	ENSMUSG00000011096	6431	24,14
1600012H06Rik	RIKEN cDNA 1600012H06 gene	ENSMUSG00000050088	16387	24,14
		ENSMUSG00000072460	312	24,14
Vrk1	vaccinia related kinase 1	ENSMUSG00000021115	67148	24,14
Nav3	neuron navigator 3	ENSMUSG00000020181	317560	24,14
Vipas39	VPS33B interacting protein, apical-basolateral polarity regulator, spe-39 homolog	ENSMUSG00000021038	27412	24,14
Tfrc	transferrin receptor	ENSMUSG00000022797	23875	24,14

Mastl	microtubule associated serine/threonine kinase-like	ENSMUSG00000026779	40419	24,14
Plekhh1	pleckstrin homology domain containing, family H (with MyTH4 domain) member 1	ENSMUSG00000060716	52486	24,14
Nutf2-ps1	nuclear transport factor 2, pseudogene 1	ENSMUSG00000071497	864	24,07
Nop14	NOP14 nucleolar protein	ENSMUSG00000036693	21617	24,07
Trappc11	trafficking protein particle complex 11	ENSMUSG00000038102	43356	24,07
Cdh3	cadherin 3	ENSMUSG00000061048	45996	24,07
Ccdc66	coiled-coil domain containing 66	ENSMUSG00000046753	26049	24,07
Hltf	helicase-like transcription factor	ENSMUSG00000002428	60680	24,07
Trim34a	tripartite motif-containing 34A	ENSMUSG00000056144	96237	24,07
4833427G06Rik	RIKEN cDNA 4833427G06 gene	ENSMUSG00000032057	20766	24,07
Cnga4	cyclic nucleotide gated channel alpha 4	ENSMUSG00000030897	4171	23,99
Dhrs7	dehydrogenase/reductase (SDR family) member 7	ENSMUSG00000021094	14473	23,99
Gnpat	glyceronephosphate O-acyltransferase	ENSMUSG00000031985	27025	23,99
1110059G10Rik	RIKEN cDNA 1110059G10 gene	ENSMUSG00000032551	5912	23,99
Kctd18	potassium channel tetramerisation domain containing 18	ENSMUSG00000054770	63856	23,99
E2f4	E2F transcription factor 4	ENSMUSG00000014859	7707	23,99
Nr2c1	nuclear receptor subfamily 2, group C, member 1	ENSMUSG00000005897	49189	23,99
Igsf10	immunoglobulin superfamily, member 10	ENSMUSG00000036334	27522	23,99
Obfc1	oligonucleotide/oligosaccharide-binding fold containing 1	ENSMUSG00000042694	36475	23,99
Ano10	anoctamin 10	ENSMUSG00000037949	118496	23,99
Uaca	uveal autoantigen with coiled-coil domains and ankyrin repeats	ENSMUSG00000034485	85823	23,99
Lrp5	low density lipoprotein receptor-related protein 5	ENSMUSG00000024913	101737	23,99
Slc25a18	solute carrier family 25 (mitochondrial carrier), member 18	ENSMUSG00000004902	20215	23,99
Smyd2	SET and MYND domain containing 2	ENSMUSG00000026603	41872	23,92
Prph	peripherin	ENSMUSG00000023484	3805	23,92
Sla2	Src-like-adaptor 2	ENSMUSG00000027636	14736	23,92
Ankzf1	ankyrin repeat and zinc finger domain containing 1	ENSMUSG00000026199	7237	23,92
Camk2a	calcium/calmodulin-dependent protein kinase II alpha	ENSMUSG00000024617	62535	23,92
Cnih4	cornichon homolog 4 (Drosophila)	ENSMUSG00000062169	18064	23,92
Zfp518a	zinc finger protein 518A	ENSMUSG00000049164	23243	23,92
Dtnbp1	dystrobrevin binding protein 1	ENSMUSG00000057531	80018	23,92
Ska3	spindle and kinetochore associated complex subunit 3	ENSMUSG00000021965	19603	23,92
Htatip2	HIV-1 tat interactive protein 2, homolog (human)	ENSMUSG00000039745	14894	23,85
Mcm3	minichromosome maintenance deficient 3 (S. cerevisiae)	ENSMUSG00000041859	17305	23,85
Dusp28	dual specificity phosphatase 28	ENSMUSG00000047067	1632	23,85

Mcm7	minichromosome maintenance deficient 7 (S. cerevisiae)	ENSMUSG00000029730	7840	23,85
Cep97	centrosomal protein 97	ENSMUSG00000022604	34968	23,85
Gm166	predicted gene 166	ENSMUSG00000057176	6212	23,85
Ubr7	ubiquitin protein ligase E3 component n-recognin 7 (putative)	ENSMUSG00000041712	19727	23,85
Arf2	ADP-ribosylation factor 2	ENSMUSG00000062421	18599	23,85
Gpx2	glutathione peroxidase 2	ENSMUSG00000042808	3220	23,85
2700097O09Rik	RIKEN cDNA 2700097O09 gene	ENSMUSG00000062198	34450	23,85
Mast1	microtubule associated serine/threonine kinase 1	ENSMUSG00000053693	25457	23,85
Ccdc122	coiled-coil domain containing 122	ENSMUSG00000034795	75486	23,85
Rexo2	REX2, RNA exonuclease 2 homolog (S. cerevisiae)	ENSMUSG00000032026	12098	23,78
Mrps15	mitochondrial ribosomal protein S15	ENSMUSG00000028861	8608	23,78
Pin4	protein (peptidyl-prolyl cis/trans isomerase) NIMA-interacting, 4 (parvulin)	ENSMUSG00000079480	8223	23,78
Inip	INTS3 and NABP interacting protein	ENSMUSG00000038544	31953	23,78
Idh3g	isocitrate dehydrogenase 3 (NAD+), gamma	ENSMUSG00000002010	7935	23,78
Tmem219	transmembrane protein 219	ENSMUSG00000060538	36747	23,78
Msra	methionine sulfoxide reductase A	ENSMUSG00000054733	333281	23,78
		ENSMUSG00000075585	5521	23,78
Cdc20b	cell division cycle 20B	ENSMUSG00000078926	56085	23,78
Nell1	NEL-like 1	ENSMUSG00000055409	890392	23,78
Pkhd1	polycystic kidney and hepatic disease 1	ENSMUSG00000043760	560286	23,78
Tmem107	transmembrane protein 107	ENSMUSG00000020895	2488	23,70
Cxxc1	CXXC finger 1 (PHD domain)	ENSMUSG00000024560	5361	23,70
		ENSMUSG00000048486	6251	23,70
Gadd45a	growth arrest and DNA-damage-inducible 45 alpha	ENSMUSG00000036390	2362	23,70
		ENSMUSG00000030689	10798	23,70
Traf5	TNF receptor-associated factor 5	ENSMUSG00000026637	95130	23,70
Synj2	synaptojanin 2	ENSMUSG00000023805	103011	23,70
Gm17484	predicted gene, 17484	ENSMUSG00000091514	15196	23,70
Gpkow	G patch domain and KOW motifs	ENSMUSG00000031148	13132	23,70
1110008L16Rik	RIKEN cDNA 1110008L16 gene	ENSMUSG00000021023	79855	23,70
Slfn2	schlafen 2	ENSMUSG00000072620	5567	23,70
Sult1b1	sulfotransferase family 1B, member 1	ENSMUSG00000029269	24857	23,70
		ENSMUSG00000006378	12685	23,63
D2Wsu81e	DNA segment, Chr 2, Wayne State University 81, expressed	ENSMUSG00000039660	5013	23,63
Nsun5	NOL1/NOP2/Sun domain family, member 5	ENSMUSG00000000916	6853	23,63
Ankrd29	ankyrin repeat domain 29	ENSMUSG00000057766	53359	23,63
Sep 08	septin 8	ENSMUSG00000018398	30309	23,63
Ap4b1	adaptor-related protein complex AP-4, beta 1	ENSMUSG00000032952	12506	23,63
Hspa2	heat shock protein 2	ENSMUSG00000059970	2759	23,63
Dnajc15	DnaJ (Hsp40) homolog, subfamily C, member 15	ENSMUSG00000022013	48701	23,63

Nudt13	nudix (nucleoside diphosphate linked moiety X)-type motif 13	ENSMUSG00000021809	22886	23,63
Wdyhv1	WDYHV motif containing 1	ENSMUSG00000022359	17217	23,63
Pag1	phosphoprotein associated with glycosphingolipid microdomains 1	ENSMUSG00000027508	146201	23,63
Klhl13	kelch-like 13	ENSMUSG00000036782	145812	23,63
Muc4	mucin 4	ENSMUSG00000079620	46506	23,63
Wdr5	WD repeat domain 5	ENSMUSG00000026917	21379	23,56
Azi1	5-azacytidine induced gene 1	ENSMUSG00000039781	22398	23,56
Rbl1	retinoblastoma-like 1 (p107)	ENSMUSG00000027641	58642	23,56
Dusp8	dual specificity phosphatase 8	ENSMUSG00000037887	16354	23,56
Kif27	kinesin family member 27	ENSMUSG00000060176	67347	23,56
Tmem41b	transmembrane protein 41B	ENSMUSG00000047554	14743	23,56
Ing3	inhibitor of growth family, member 3	ENSMUSG00000029670	26468	23,56
Slc35a2	solute carrier family 35 (UDP-galactose transporter), member A2	ENSMUSG00000031156	10452	23,56
Mlph	melanophilin	ENSMUSG00000026303	36058	23,56
Fech	ferrochelatase	ENSMUSG00000024588	32517	23,56
Grk5	G protein-coupled receptor kinase 5	ENSMUSG00000003228	202805	23,56
Tmco4	transmembrane and coiled-coil domains 4	ENSMUSG00000041143	86284	23,56
Gstcd	glutathione S-transferase, C-terminal domain containing	ENSMUSG00000028018	110281	23,56
Sik1	salt inducible kinase 1	ENSMUSG00000024042	11545	23,56
		ENSMUSG00000063445	9298	23,49
Wdr34	WD repeat domain 34	ENSMUSG00000039715	17336	23,49
Dnajc27	DnaJ (Hsp40) homolog, subfamily C, member 27	ENSMUSG00000020657	28039	23,49
Med24	mediator complex subunit 24	ENSMUSG00000017210	24845	23,49
		ENSMUSG00000029003	15316	23,49
		ENSMUSG00000036352	23205	23,49
Cct3	chaperonin containing Tcp1, subunit 3 (gamma)	ENSMUSG00000001416	24652	23,49
Ddx19b	DEAD (Asp-Glu-Ala-Asp) box polypeptide 19b	ENSMUSG00000033658	28564	23,49
Ttll1	tubulin tyrosine ligase-like 1	ENSMUSG00000022442	27122	23,49
Gne	glucosamine (UDP-N-acetyl)-2-epimerase/N-acetylmannosamine kinase	ENSMUSG00000028479	50103	23,49
		ENSMUSG00000048351	13405	23,49
2610002J02Rik	RIKEN cDNA 2610002J02 gene	ENSMUSG00000073684	6886	23,49
		ENSMUSG00000026156	87406	23,49
Bank1	B cell scaffold protein with ankyrin repeats 1	ENSMUSG00000037922	272684	23,49
Masp2	mannan-binding lectin serine peptidase 2	ENSMUSG00000028979	12946	23,49
Cherp	calcium homeostasis endoplasmic reticulum protein	ENSMUSG00000052488	14745	23,49
Tmprss11e	transmembrane protease, serine 11e	ENSMUSG00000054537	40630	23,49
Lrrc10b	leucine rich repeat containing 10B	ENSMUSG00000090291	2077	23,41
Ndp	Norrie disease (pseudoglioma) (human)	ENSMUSG00000040138	26254	23,41
Apoa1bp	apolipoprotein A-I binding protein	ENSMUSG00000028070	1973	23,41

Tufm	Tu translation elongation factor, mitochondrial	ENSMUSG00000073838	3376	23,41
Rac3	RAS-related C3 botulinum substrate 3	ENSMUSG00000018012	2500	23,41
Hars2	histidyl-tRNA synthetase 2, mitochondrial (putative)	ENSMUSG00000019143	9555	23,41
Dnlz	DNL-type zinc finger	ENSMUSG00000075467	3990	23,41
Fam83h	family with sequence similarity 83, member H	ENSMUSG00000046761	13244	23,41
Ctsh	cathepsin H	ENSMUSG00000032359	21938	23,41
Thbs3	thrombospondin 3	ENSMUSG00000028047	11658	23,41
Snph	syntaphilin	ENSMUSG00000027457	42045	23,34
Kbtbd2	kelch repeat and BTB (POZ) domain containing 2	ENSMUSG00000059486	20290	23,34
Mtss1l	metastasis suppressor 1-like	ENSMUSG00000033763	19925	23,34
Ap1s2	adaptor-related protein complex 1, sigma 2 subunit	ENSMUSG00000031367	24650	23,34
Fam210a	family with sequence similarity 210, member A	ENSMUSG00000038121	40147	23,34
Rsph4a	radial spoke head 4 homolog A (Chlamydomonas)	ENSMUSG00000039552	10911	23,34
Nek8	NIMA (never in mitosis gene a)-related expressed kinase 8	ENSMUSG00000017405	10570	23,34
Frrs1	ferric-chelate reductase 1	ENSMUSG00000033386	25524	23,34
2310047M10Rik	RIKEN cDNA 2310047M10 gene	ENSMUSG00000045176	1802	23,27
Csrnp2	cysteine-serine-rich nuclear protein 2	ENSMUSG00000044636	15670	23,27
Pmm1	phosphomannomutase 1	ENSMUSG00000022474	9823	23,27
		ENSMUSG00000028907	15441	23,27
Gps2	G protein pathway suppressor 2	ENSMUSG00000023170	2704	23,27
Cables1	CDK5 and Abl enzyme substrate 1	ENSMUSG00000040957	106408	23,27
Zfp212	Zinc finger protein 212	ENSMUSG00000052763	12164	23,27
Heatr2	HEAT repeat containing 2	ENSMUSG00000025857	36288	23,27
Ttc23	tetratricopeptide repeat domain 23	ENSMUSG00000030555	79167	23,27
Cast	calpastatin	ENSMUSG00000021585	113636	23,27
Tomm34	translocase of outer mitochondrial membrane 34	ENSMUSG00000018322	17630	23,20
Isoc1	isochorismatase domain containing 1	ENSMUSG00000024601	20089	23,20
Pdrg1	p53 and DNA damage regulated 1	ENSMUSG00000027472	6538	23,20
Ect2	ect2 oncogene	ENSMUSG00000027699	56657	23,20
Irf2bp1	interferon regulatory factor 2 binding protein 1	ENSMUSG00000044030	2697	23,20
Socs5	suppressor of cytokine signaling 5	ENSMUSG00000037104	29905	23,20
Rint1	RAD50 interactor 1	ENSMUSG00000028999	32659	23,20
Tagap1	T cell activation GTPase activating protein 1	ENSMUSG00000052031	6192	23,20
Gng5	guanine nucleotide binding protein (G protein), gamma 5	ENSMUSG00000068523	5739	23,20
Macc1	metastasis associated in colon cancer 1	ENSMUSG00000041886	23525	23,20
Pigyl	phosphatidylinositol glycan anchor biosynthesis, class Y-like	ENSMUSG00000010607	1513	23,12
		ENSMUSG00000061650	14000	23,12
		ENSMUSG00000033475	1741	23,12

Nedd1	neural precursor cell expressed, developmentally down-regulated gene 1	ENSMUSG00000019988	37675	23,12
Pbx2	pre B cell leukemia homeobox 2	ENSMUSG00000034673	6135	23,12
D8Ertd82e	DNA segment, Chr 8, ERATO Doi 82, expressed	ENSMUSG00000050271	52960	23,12
Tjap1	tight junction associated protein 1	ENSMUSG00000012296	25176	23,12
Mccc2	methylcrotonoyl-Coenzyme A carboxylase 2 (beta)	ENSMUSG00000021646	67107	23,12
Fam69a	family with sequence similarity 69, member A	ENSMUSG00000029270	79033	23,12
Paqr3	progestin and adipoQ receptor family member III	ENSMUSG00000055725	29268	23,12
Tab3	TGF-beta activated kinase 1/MAP3K7 binding protein 3	ENSMUSG00000035476	60448	23,12
		ENSMUSG00000033147	65500	23,12
		ENSMUSG00000046782	173816	23,12
Cactin	cactin, spliceosome C complex subunit	ENSMUSG00000034889	5149	23,05
Ruvbl1	RuvB-like protein 1	ENSMUSG00000030079	32164	23,05
Cdc25b	cell division cycle 25B	ENSMUSG00000027330	11549	23,05
Dpm2	dolichol-phosphate (beta-D) mannosyltransferase 2	ENSMUSG00000026810	2722	23,05
Zfp787	zinc finger protein 787	ENSMUSG00000046792	24483	23,05
Pdlim7	PDZ and LIM domain 7	ENSMUSG00000021493	17882	23,05
Reep6	receptor accessory protein 6	ENSMUSG00000035504	6489	23,05
		ENSMUSG00000079084	42428	23,05
Hibch	3-hydroxyisobutyryl-Coenzyme A hydrolase	ENSMUSG00000041426	75974	23,05
Zbbx	zinc finger, B-box domain containing	ENSMUSG00000034151	127128	23,05
Ankrd54	ankyrin repeat domain 54	ENSMUSG00000033055	9800	22,98
Nipsnap1	4-nitrophenylphosphatase domain and non-neuronal SNAP25-like protein homolog 1 (C. elegans)	ENSMUSG00000034285	20250	22,98
Alkbh7	alkB, alkylation repair homolog 7 (E. coli)	ENSMUSG00000002661	1984	22,98
Lcmt1	leucine carboxyl methyltransferase 1	ENSMUSG00000030763	52377	22,98
Pddc1	Parkinson disease 7 domain containing 1	ENSMUSG00000051007	6002	22,98
Glrx2	glutaredoxin 2 (thioltransferase)	ENSMUSG00000018196	10648	22,98
Stk36	serine/threonine kinase 36	ENSMUSG00000033276	35450	22,98
Extl3	exostoses (multiple)-like 3	ENSMUSG00000021978	46047	22,98
3110002H16Rik	RIKEN cDNA 3110002H16 gene	ENSMUSG00000024410	21281	22,98
		ENSMUSG00000003604	141610	22,98
Itgb4	integrin beta 4	ENSMUSG00000020758	33704	22,98
Mid2	midline 2	ENSMUSG00000000266	103117	22,98
Taf4b	TAF4B RNA polymerase II, TATA box binding protein (TBP)-associated factor	ENSMUSG00000054321	117115	22,98
Ppp1r14b	protein phosphatase 1, regulatory (inhibitor) subunit 14B	ENSMUSG00000056612	2277	22,91
Bicc1	bicaudal C homolog 1 (Drosophila)	ENSMUSG00000014329	236869	22,91
Capn10	calpain 10	ENSMUSG00000026270	13566	22,91

Nip7	nuclear import 7 homolog (S. cerevisiae)	ENSMUSG00000031917	4050	22,91
Tsfm	Ts translation elongation factor, mitochondrial	ENSMUSG00000040521	19269	22,91
Tomm5	translocase of outer mitochondrial membrane 5 homolog (yeast)	ENSMUSG00000078713	2907	22,91
Prr15	proline rich 15	ENSMUSG00000045725	3189	22,91
Net1	neuroepithelial cell transforming gene 1	ENSMUSG00000021215	35656	22,91
Me3	malic enzyme 3, NADP(+)-dependent, mitochondrial	ENSMUSG00000030621	221968	22,91
Gxylt2	glucoside xylosyltransferase 2	ENSMUSG00000030074	100348	22,91
Lrrc49	leucine rich repeat containing 49	ENSMUSG00000047766	119300	22,91
Prr3	proline-rich polypeptide 3	ENSMUSG00000038500	7696	22,83
Dhrs13	dehydrogenase/reductase (SDR family) member 13	ENSMUSG00000020834	5587	22,83
Cd72	CD72 antigen	ENSMUSG00000028459	8167	22,83
Manba	mannosidase, beta A, lysosomal	ENSMUSG00000028164	85794	22,83
Ndufs7	NADH dehydrogenase (ubiquinone) Fe-S protein 7	ENSMUSG00000020153	7674	22,83
Pdzd4	PDZ domain containing 4	ENSMUSG00000002006	31611	22,83
Glb1l	galactosidase, beta 1-like	ENSMUSG00000026200	12536	22,83
Tada2a	transcriptional adaptor 2A	ENSMUSG00000018651	50681	22,83
Necab2	N-terminal EF-hand calcium binding protein 2	ENSMUSG00000031837	25922	22,83
Mipep	mitochondrial intermediate peptidase	ENSMUSG00000021993	119045	22,83
Thap4	THAP domain containing 4	ENSMUSG00000026279	49448	22,83
Eif2ak4	eukaryotic translation initiation factor 2 alpha kinase 4	ENSMUSG00000005102	86617	22,83
Irak2	interleukin-1 receptor-associated kinase 2	ENSMUSG00000060477	56543	22,83
Rad51b	RAD51 homolog B	ENSMUSG00000059060	211306	22,83
Plcb3	phospholipase C, beta 3	ENSMUSG00000024960	16046	22,76
Sfxn3	sideroflexin 3	ENSMUSG00000025212	8808	22,76
Golt1b	golgi transport 1 homolog B (S. cerevisiae)	ENSMUSG00000030245	16638	22,76
Pom121	nuclear pore membrane protein 121	ENSMUSG00000053293	18406	22,76
		ENSMUSG00000092544	32025	22,76
Gskip	GSK3B interacting protein	ENSMUSG00000044715	17696	22,76
Gm10709	predicted gene 10709	ENSMUSG00000074516	654	22,76
Donson	downstream neighbor of SON	ENSMUSG00000022960	11498	22,76
Ap2a1	adaptor-related protein complex 2, alpha 1 subunit	ENSMUSG00000060279	29124	22,76
Bcl2l14	BCL2-like 14 (apoptosis facilitator)	ENSMUSG00000030200	42419	22,76
Rtel1	regulator of telomere elongation helicase 1	ENSMUSG00000038685	36878	22,76
Gm11744	predicted gene 11744	ENSMUSG00000075410	14856	22,76
Parp3	poly (ADP-ribose) polymerase family, member 3	ENSMUSG00000023249	6628	22,76
9030617O03Rik	RIKEN cDNA 9030617O03 gene	ENSMUSG00000021185	117925	22,76
Fam207a	family with sequence similarity 207, member A	ENSMUSG00000032977	29158	22,69
Dbn1	drebrin 1	ENSMUSG00000034675	14683	22,69
Ehd3	EH-domain containing 3	ENSMUSG00000024065	27253	22,69

Lrp12	low density lipoprotein-related protein 12	ENSMUSG00000022305	73392	22,69
Slc35c1	solute carrier family 35, member C1	ENSMUSG00000049922	7775	22,69
Ppp1r3f	protein phosphatase 1, regulatory (inhibitor) subunit 3F	ENSMUSG00000039556	48086	22,69
Kbtbd7	kelch repeat and BTB (POZ) domain containing 7	ENSMUSG00000043881	4529	22,69
Ctu1	cytosolic thiouridylase subunit 1 homolog (S. pombe)	ENSMUSG00000038888	6283	22,69
Tpcn1	two pore channel 1	ENSMUSG00000032741	54454	22,69
Rnf26	ring finger protein 26	ENSMUSG00000053128	47191	22,69
Stard13	StAR-related lipid transfer (START) domain containing 13	ENSMUSG00000016128	196209	22,69
		ENSMUSG00000053091	27368	22,69
Plekhg3	pleckstrin homology domain containing, family G (with RhoGef domain) member 3	ENSMUSG00000052609	45480	22,69
Med25	mediator of RNA polymerase II transcription, subunit 25 homolog (yeast)	ENSMUSG00000002968	13007	22,69
Crybg3	beta-gamma crystallin domain containing 3	ENSMUSG00000022723	64978	22,69
Fbxl22	F-box and leucine-rich repeat protein 22	ENSMUSG00000050503	6135	22,69
Shroom4	shroom family member 4	ENSMUSG00000068270	237595	22,69
Map7d1	MAP7 domain containing 1	ENSMUSG00000028849	24177	22,62
Cuedc2	CUE domain containing 2	ENSMUSG00000036748	8849	22,62
Zscan22	zinc finger and SCAN domain containing 22	ENSMUSG00000054715	11269	22,62
Fhl1	four and a half LIM domains 1	ENSMUSG00000023092	61560	22,62
		ENSMUSG00000025132	3521	22,62
Gm2382	predicted gene 2382	ENSMUSG00000079427	31194	22,62
Ttc32	tetratricopeptide repeat domain 32	ENSMUSG00000066637	6398	22,62
Brip1	BRCA1 interacting protein C-terminal helicase 1	ENSMUSG00000034329	143056	22,62
Klhl15	kelch-like 15	ENSMUSG00000043929	43126	22,62
Ndrg4	N-myc downstream regulated gene 4	ENSMUSG00000036564	38140	22,54
Nt5c3b	5'-nucleotidase, cytosolic IIIB	ENSMUSG00000017176	19488	22,54
Smim9	small integral membrane protein 9	ENSMUSG00000073094	17715	22,54
Ngdn	neuroguidin, EIF4E binding protein	ENSMUSG00000022204	8682	22,54
Cstf2t	cleavage stimulation factor, 3' pre-RNA subunit 2, tau	ENSMUSG00000053536	3750	22,54
		ENSMUSG00000079444	7717	22,54
Lamtor1	late endosomal/lysosomal adaptor, MAPK and MTOR activator 1	ENSMUSG00000030842	6065	22,54
Gucy2c	guanylate cyclase 2c	ENSMUSG00000042638	84481	22,54
Creld2	cysteine-rich with EGF-like domains 2	ENSMUSG00000023272	7036	22,54
Tada2b	transcriptional adaptor 2B	ENSMUSG00000029196	10616	22,54
Ric3	resistance to inhibitors of cholinesterase 3 homolog (C. elegans)	ENSMUSG00000048330	49020	22,54

Gga1	golgi associated, gamma adaptin ear containing, ARF binding protein 1	ENSMUSG00000033128	17396	22,54
Gm10036	predicted gene 10036	ENSMUSG00000058064	537	22,54
Fzd8	frizzled homolog 8 (Drosophila)	ENSMUSG00000036904	3346	22,54
Mamdc4	MAM domain containing 4	ENSMUSG00000026941	11731	22,54
Spice1	spindle and centriole associated protein 1	ENSMUSG00000043065	41377	22,54
Ppp2r1b	protein phosphatase 2 (formerly 2A), regulatory subunit A (PR 65), beta isoform	ENSMUSG00000032058	48929	22,54
Lsm2	LSM2 homolog, U6 small nuclear RNA associated (S. cerevisiae)	ENSMUSG00000007050	4030	22,47
Mogs	mannosyl-oligosaccharide glucosidase	ENSMUSG00000030036	3403	22,47
Poc5	POC5 centriolar protein homolog (Chlamydomonas)	ENSMUSG00000021671	27294	22,47
Rbm14	RNA binding motif protein 14	ENSMUSG00000006456	11066	22,47
Echdc1	enoyl Coenzyme A hydratase domain containing 1	ENSMUSG00000019883	33496	22,47
Scyl3	SCY1-like 3 (S. cerevisiae)	ENSMUSG00000026584	26027	22,47
		ENSMUSG00000025971	11626	22,47
BC055324	cDNA sequence BC055324	ENSMUSG00000041406	48804	22,47
Zfp410	zinc finger protein 410	ENSMUSG00000042472	26972	22,47
Spag1	sperm associated antigen 1	ENSMUSG00000037617	56243	22,47
Gas7	growth arrest specific 7	ENSMUSG00000033066	233554	22,47
Cops6	COP9 (constitutive photomorphogenic) homolog, subunit 6 (Arabidopsis thaliana)	ENSMUSG00000019494	3576	22,40
Agpat9	1-acylglycerol-3-phosphate O-acyltransferase 9	ENSMUSG00000029314	53390	22,40
Fam92a	family with sequence similarity 92, member A	ENSMUSG00000028218	18607	22,40
Pgm2	phosphoglucomutase 2	ENSMUSG00000025791	57881	22,40
Tspyl1	testis-specific protein, Y-encoded-like 1	ENSMUSG00000047514	2696	22,40
Ubox5	U box domain containing 5	ENSMUSG00000027300	40037	22,40
Slc35e3	solute carrier family 35, member E3	ENSMUSG00000060181	12680	22,40
Cables2	CDK5 and Abl enzyme substrate 2	ENSMUSG00000038990	14957	22,40
Wdr48	WD repeat domain 48	ENSMUSG00000032512	31685	22,40
Zmat1	zinc finger, matrin type 1	ENSMUSG00000052676	37838	22,40
Ulk1	unc-51 like kinase 1	ENSMUSG00000029512	25610	22,40
Cars	cysteinyl-tRNA synthetase	ENSMUSG00000010755	42861	22,40
Tm6sf1	transmembrane 6 superfamily member 1	ENSMUSG00000038623	25434	22,40
Pnpt1	polyribonucleotide nucleotidyltransferase 1	ENSMUSG00000020464	31085	22,40
P2ry12	purinergic receptor P2Y, G-protein coupled 12	ENSMUSG00000036353	46561	22,40
Ppp2r2c	protein phosphatase 2 (formerly 2A), regulatory subunit B (PR 52), gamma isoform	ENSMUSG00000029120	86566	22,33
Krt10	keratin 10	ENSMUSG00000019761	4111	22,33
Fam217a	family with sequence similarity 217, member A	ENSMUSG00000021414	10029	22,33

Jmjd7	jumonji domain containing 7	ENSMUSG00000033852	15551	22,33
		ENSMUSG00000025525	61422	22,33
Hax1	HCLS1 associated X-1	ENSMUSG00000027944	3230	22,33
Pigx	phosphatidylinositol glycan anchor biosynthesis, class X	ENSMUSG00000023791	15319	22,33
Tmem192	transmembrane protein 192	ENSMUSG00000025521	21850	22,33
		ENSMUSG00000047547	18944	22,33
Pgpep1	pyroglutamyl-peptidase I	ENSMUSG00000056204	13303	22,33
Phf13	PHD finger protein 13	ENSMUSG00000047777	6626	22,33
Slc9a1	solute carrier family 9 (sodium/hydrogen exchanger), member 1	ENSMUSG00000028854	53997	22,33
Tns1	tensin 1	ENSMUSG00000055322	214217	22,33
Riok2	RIO kinase 2 (yeast)	ENSMUSG00000023846	20567	22,33
Dcun1d2	DCN1, defective in cullin neddylation 1, domain containing 2 (S. cerevisiae)	ENSMUSG00000038506	32164	22,33
Mblac2	metallo-beta-lactamase domain containing 2	ENSMUSG00000051098	41859	22,33
Zmynd12	zinc finger, MYND domain containing 12	ENSMUSG00000070806	44631	22,33
		ENSMUSG00000047793	65225	22,33
Sun2	Sad1 and UNC84 domain containing 2	ENSMUSG00000042524	18467	22,33
Lmbr1l	limb region 1 like	ENSMUSG00000022999	14311	22,33
Ccdc19	coiled-coil domain containing 19	ENSMUSG00000026546	24827	22,33
Rassf4	Ras association (RalGDS/AF-6) domain family member 4	ENSMUSG00000042129	40829	22,25
Fam3a	family with sequence similarity 3, member A	ENSMUSG00000031399	8554	22,25
Trim14	tripartite motif-containing 14	ENSMUSG00000039853	41323	22,25
Senp3	SUMO/sentrin specific peptidase 3	ENSMUSG00000005204	8970	22,25
Csgalnact1	chondroitin sulfate N-acetylgalactosaminyltransferase 1	ENSMUSG00000036356	378366	22,25
Morc3	microrchidia 3	ENSMUSG00000039456	43952	22,25
Thrb	thyroid hormone receptor beta	ENSMUSG00000021779	377129	22,25
Proca1	protein interacting with cyclin A1	ENSMUSG00000044122	12372	22,25
Lamtor4	late endosomal/lysosomal adaptor, MAPK and MTOR activator 4	ENSMUSG00000050552	3791	22,18
Tyk2	tyrosine kinase 2	ENSMUSG00000032175	23278	22,18
Aqr	aquarius	ENSMUSG00000040383	85855	22,18
Slc30a5	solute carrier family 30 (zinc transporter), member 5	ENSMUSG00000021629	30779	22,18
Nexn	nexilin	ENSMUSG00000039103	29335	22,18
Slc2a12	solute carrier family 2 (facilitated glucose transporter), member 12	ENSMUSG00000037490	59275	22,11
		ENSMUSG00000022407	22458	22,11
Wnt5b	wingless-related MMTV integration site 5B	ENSMUSG00000030170	111817	22,11
Pgm3	phosphoglucomutase 3	ENSMUSG00000056131	19367	22,11
Skap2	src family associated phosphoprotein 2	ENSMUSG00000059182	153384	22,11
		ENSMUSG00000093485	30237	22,11
Ids	iduronate 2-sulfatase	ENSMUSG00000035847	22016	22,11
Zbtb40	zinc finger and BTB domain containing 40	ENSMUSG00000060862	69070	22,11

Dgat1	diacylglycerol O-acyltransferase 1	ENSMUSG00000022555	9937	22,11
Plag1	pleiomorphic adenoma gene 1	ENSMUSG00000003282	37428	22,11
Galns	galactosamine (N-acetyl)-6-sulfate sulfatase	ENSMUSG00000015027	33250	22,04
Mcm5	minichromosome maintenance deficient 5, cell division cycle 46 (S. cerevisiae)	ENSMUSG00000005410	18912	22,04
		ENSMUSG00000024378	14508	22,04
Tmem42	transmembrane protein 42	ENSMUSG00000066233	2165	22,04
Deaf1	deformed epidermal autoregulatory factor 1 (Drosophila)	ENSMUSG00000058886	30550	22,04
Tysnd1	trypsin domain containing 1	ENSMUSG00000020087	7260	22,04
Zfp777	zinc finger protein 777	ENSMUSG00000071477	24724	22,04
2310033P09Rik	RIKEN cDNA 2310033P09 gene	ENSMUSG00000020441	2418	22,04
Gm14698	predicted gene 14698	ENSMUSG00000071748	4226	22,04
Ell	elongation factor RNA polymerase II	ENSMUSG00000070002	53184	22,04
Ppm1d	protein phosphatase 1D magnesium-dependent, delta isoform	ENSMUSG00000020525	35823	22,04
Rps6ka2	ribosomal protein S6 kinase, polypeptide 2	ENSMUSG00000023809	133199	22,04
Usp45	ubiquitin specific petidase 45	ENSMUSG00000040455	70717	22,04
Cdc20	cell division cycle 20	ENSMUSG00000006398	4452	21,96
Slc25a33	solute carrier family 25, member 33	ENSMUSG00000028982	30242	21,96
Sdhaf2	succinate dehydrogenase complex assembly factor 2	ENSMUSG00000024668	24697	21,96
		ENSMUSG00000028899	21392	21,96
Acaa2	acetyl-Coenzyme A acyltransferase 2 (mitochondrial 3-oxoacyl-Coenzyme A thiolase)	ENSMUSG00000036880	27011	21,96
Tmem104	transmembrane protein 104	ENSMUSG00000045980	59537	21,96
4932443I19Rik	RIKEN cDNA 4932443I19 gene	ENSMUSG00000090336	37178	21,96
		ENSMUSG00000093701	27731	21,96
		ENSMUSG00000033624	42445	21,96
Casc5	cancer susceptibility candidate 5	ENSMUSG00000027326	58383	21,96
Efcab2	EF-hand calcium binding domain 2	ENSMUSG00000026495	77367	21,96
Dennd2d	DENN/MADD domain containing 2D	ENSMUSG00000027901	20626	21,96
Nsfl1c	NSFL1 (p97) cofactor (p47)	ENSMUSG00000027455	17233	21,89
Dcps	decapping enzyme, scavenger	ENSMUSG00000032040	51654	21,89
Adck2	aarF domain containing kinase 2	ENSMUSG00000046947	22723	21,89
Nup107	nucleoporin 107	ENSMUSG00000052798	42082	21,89
Dhx16	DEAH (Asp-Glu-Ala-His) box polypeptide 16	ENSMUSG00000024422	12852	21,89
		ENSMUSG00000071151	657	21,89
Prmt3	protein arginine N-methyltransferase 3	ENSMUSG00000030505	79920	21,89
Npc1	Niemann Pick type C1	ENSMUSG00000024413	46708	21,89
B3galnt2	UDP-GalNAc:betaGlcNAc beta 1,3-galactosaminyltransferase, polypeptide 2	ENSMUSG00000039242	44395	21,89
Fam107a	family with sequence similarity 107, member A	ENSMUSG00000021750	21750	21,89
Iqck	IQ motif containing K	ENSMUSG00000073856	116893	21,89
Sfxn5	sideroflexin 5	ENSMUSG00000033720	120372	21,89

Pxn	paxillin	ENSMUSG00000029528	49312	21,89
Arsb	arylsulfatase B	ENSMUSG00000042082	171338	21,89
Timm23	translocase of inner mitochondrial membrane 23	ENSMUSG00000013701	21737	21,89
Rhebl1	Ras homolog enriched in brain like 1	ENSMUSG00000023755	3627	21,82
Thap7	THAP domain containing 7	ENSMUSG00000022760	3155	21,82
Xpc	xeroderma pigmentosum, complementation group C	ENSMUSG00000030094	26580	21,82
Ccdc43	coiled-coil domain containing 43	ENSMUSG00000020925	13097	21,82
Dpy19l1	dpy-19-like 1 (C. elegans)	ENSMUSG00000043067	91365	21,82
Gtf3c4	general transcription factor IIIC, polypeptide 4	ENSMUSG00000035666	18062	21,82
Agbl2	ATP/GTP binding protein-like 2	ENSMUSG00000040812	51711	21,82
Trappc6b	trafficking protein particle complex 6B	ENSMUSG00000020993	18369	21,82
Fam192a	family with sequence similarity 192, member A	ENSMUSG00000031774	26784	21,82
Usp10	ubiquitin specific peptidase 10	ENSMUSG00000031826	47201	21,82
Wdr4	WD repeat domain 4	ENSMUSG00000024037	25652	21,82
Zmat3	zinc finger matrin type 3	ENSMUSG00000027663	30883	21,82
Fgf2	fibroblast growth factor 2	ENSMUSG00000037225	92859	21,82
Scrib	scribbled homolog (Drosophila)	ENSMUSG00000022568	22627	21,82
Eif2ak2	eukaryotic translation initiation factor 2-alpha kinase 2	ENSMUSG00000024079	30010	21,82
Gm973	predicted gene 973	ENSMUSG00000047361	118245	21,82
Sh2d4b	SH2 domain containing 4B	ENSMUSG00000037833	79478	21,82
Edn2	endothelin 2	ENSMUSG00000028635	6155	21,82
Mcm6	minichromosome maintenance deficient 6 (MIS5 homolog, S. pombe) (S. cerevisiae)	ENSMUSG00000026355	28066	21,75
Gpalpp1	GPALPP motifs containing 1	ENSMUSG00000022008	24584	21,75
Galc	galactosylceramidase	ENSMUSG00000021003	57156	21,75
Angpt1	angiopoietin 1	ENSMUSG00000022309	252251	21,75
Fam185a	family with sequence similarity 185, member A	ENSMUSG00000047221	57167	21,75
Papd4	PAP associated domain containing 4	ENSMUSG00000042167	44884	21,75
Cobll1	Cobl-like 1	ENSMUSG00000034903	151065	21,75
		ENSMUSG00000024925	1567	21,67
Chpf	chondroitin polymerizing factor	ENSMUSG00000032997	4903	21,67
Rarg	retinoic acid receptor, gamma	ENSMUSG00000001288	22580	21,67
Tmem209	transmembrane protein 209	ENSMUSG00000029782	30731	21,67
M6pr	mannose-6-phosphate receptor, cation dependent	ENSMUSG00000007458	8961	21,67
		ENSMUSG00000030614	7808	21,67
D19Bwg1357e	DNA segment, Chr 19, Brigham & Women's Genetics 1357 expressed	ENSMUSG00000041360	41119	21,67
		ENSMUSG00000091931	37836	21,67
Ppm1k	protein phosphatase 1K (PP2C domain containing)	ENSMUSG00000037826	28925	21,67
Ippk	inositol 1,3,4,5,6-pentakisphosphate 2-kinase	ENSMUSG00000021385	41680	21,67
Mttp	microsomal triglyceride transfer protein	ENSMUSG00000028158	53534	21,67

Dnajc24	DnaJ (Hsp40) homolog, subfamily C, member 24	ENSMUSG00000027166	36841	21,67
Ptk2b	PTK2 protein tyrosine kinase 2 beta	ENSMUSG00000059456	127796	21,67
Timm10	translocase of inner mitochondrial membrane 10	ENSMUSG00000027076	3217	21,60
		ENSMUSG00000006362	70371	21,60
1700088E04Rik	RIKEN cDNA 1700088E04 gene	ENSMUSG00000033029	6597	21,60
Wdr3	WD repeat domain 3	ENSMUSG00000033285	24228	21,60
1700007K13Rik	RIKEN cDNA 1700007K13 gene	ENSMUSG00000026831	4332	21,60
Stxbp3a	syntaxin binding protein 3A	ENSMUSG00000027882	47351	21,60
Inpp4a	inositol polyphosphate-4-phosphatase, type I	ENSMUSG00000026113	110872	21,60
Stat1	signal transducer and activator of transcription 1	ENSMUSG00000026104	42428	21,60
Fabp5	fatty acid binding protein 5, epidermal	ENSMUSG00000027533	4060	21,53
Gm10116	predicted pseudogene 10116	ENSMUSG00000062382	922	21,53
		ENSMUSG00000026974	12284	21,53
Fam35a	family with sequence similarity 35, member A	ENSMUSG00000041471	73463	21,53
Isy1	ISY1 splicing factor homolog (S. cerevisiae)	ENSMUSG00000030056	20313	21,53
Nupl1	nucleoporin like 1	ENSMUSG00000063895	32304	21,53
C330018D20Rik	RIKEN cDNA C330018D20 gene	ENSMUSG00000024592	19536	21,53
		ENSMUSG00000089824	20021	21,53
Dscr3	Down syndrome critical region gene 3	ENSMUSG00000022898	29048	21,53
Dars2	aspartyl-tRNA synthetase 2 (mitochondrial)	ENSMUSG00000026709	30058	21,53
Tango2	transport and golgi organization 2	ENSMUSG00000013539	47279	21,53
Plgrkt	plasminogen receptor, C-terminal lysine transmembrane protein	ENSMUSG00000016495	18792	21,53
4930430F08Rik	RIKEN cDNA 4930430F08 gene	ENSMUSG00000046567	16986	21,53
Guf1	GUF1 GTPase homolog (S. cerevisiae)	ENSMUSG00000029208	19049	21,53
Otud1	OTU domain containing 1	ENSMUSG00000043415	2529	21,53
Cyp27a1	cytochrome P450, family 27, subfamily a, polypeptide 1	ENSMUSG00000026170	24317	21,53
Ttc37	tetratricopeptide repeat domain 37	ENSMUSG00000033991	89250	21,53
Fmo5	flavin containing monooxygenase 5	ENSMUSG00000028088	26479	21,46
Ccdc3	coiled-coil domain containing 3	ENSMUSG00000026676	93103	21,46
Tgfbr2	transforming growth factor, beta receptor II	ENSMUSG00000032440	87666	21,46
Mrpl3	mitochondrial ribosomal protein L3	ENSMUSG00000032563	24228	21,46
Pld3	phospholipase D family, member 3	ENSMUSG00000003363	21219	21,46
Tmem109	transmembrane protein 109	ENSMUSG00000034659	11342	21,46
Sigirr	single immunoglobulin and toll-interleukin 1 receptor (TIR) domain	ENSMUSG00000025494	9372	21,46
Gm16515	predicted gene, Gm16515	ENSMUSG00000018931	12505	21,46
Zfp579	zinc finger protein 579	ENSMUSG00000051550	12676	21,46
		ENSMUSG00000009207	59145	21,46
Pex2	peroxisomal biogenesis factor 2	ENSMUSG00000040374	16052	21,46
Med6	mediator of RNA polymerase II transcription, subunit 6 homolog (yeast)	ENSMUSG00000002679	21452	21,46

Oxsr1	oxidative-stress responsive 1	ENSMUSG00000036737	83996	21,46
Ncapd3	non-SMC condensin II complex, subunit D3	ENSMUSG00000035024	65137	21,46
		ENSMUSG00000078772	45680	21,46
Cftr	cystic fibrosis transmembrane conductance regulator	ENSMUSG00000041301	152082	21,46
Pcdh8	protocadherin 8	ENSMUSG00000036422	4541	21,38
Trpm5	transient receptor potential cation channel, subfamily M, member 5	ENSMUSG00000009246	25490	21,38
Fkbp8	FK506 binding protein 8	ENSMUSG00000019428	7605	21,38
Spr	sepiapterin reductase	ENSMUSG00000033735	4089	21,38
Prodh	proline dehydrogenase	ENSMUSG00000003526	29847	21,38
Prickle3	prickle homolog 3 (Drosophila)	ENSMUSG00000031145	10927	21,38
Fzd1	frizzled homolog 1 (Drosophila)	ENSMUSG00000044674	4375	21,38
Rnpc3	RNA-binding region (RNP1, RRM) containing 3	ENSMUSG00000027981	25083	21,38
Zfp592	zinc finger protein 592	ENSMUSG00000005621	51484	21,38
Dnajb9	DnaJ (Hsp40) homolog, subfamily B, member 9	ENSMUSG00000014905	4172	21,31
Xrcc3	X-ray repair complementing defective repair in Chinese hamster cells 3	ENSMUSG00000021287	10682	21,31
Polr3k	polymerase (RNA) III (DNA directed) polypeptide K	ENSMUSG00000038628	6494	21,31
Hsd17b7	hydroxysteroid (17-beta) dehydrogenase 7	ENSMUSG00000026675	19707	21,31
Ppfia3	protein tyrosine phosphatase, receptor type, f polypeptide (PTPRF), interacting protein (liprin), alpha 3	ENSMUSG00000003863	27894	21,31
Dtx2	deltex 2 homolog (Drosophila)	ENSMUSG00000004947	38073	21,31
Pde1a	phosphodiesterase 1A, calmodulin-dependent	ENSMUSG00000059173	295006	21,31
Klhl42	kelch-like 42	ENSMUSG00000040102	21400	21,24
Cdk16	cyclin-dependent kinase 16	ENSMUSG00000031065	11927	21,24
Phyhd1	phytanoyl-CoA dioxygenase domain containing 1	ENSMUSG00000079484	34878	21,24
Tm7sf2	transmembrane 7 superfamily member 2	ENSMUSG00000024799	5553	21,24
Trim23	tripartite motif-containing 23	ENSMUSG00000021712	24576	21,24
Trim39	tripartite motif-containing 39	ENSMUSG00000045409	13375	21,24
Trmt11	tRNA methyltransferase 11	ENSMUSG00000019792	66525	21,24
Pex7	peroxisomal biogenesis factor 7	ENSMUSG00000020003	47761	21,24
Rfx1	regulatory factor X, 1 (influences HLA class II expression)	ENSMUSG00000031706	30157	21,24
Polr3e	polymerase (RNA) III (DNA directed) polypeptide E	ENSMUSG00000030880	29689	21,24
Misp	mitotic spindle positioning	ENSMUSG00000035852	9432	21,24
Pkn1	protein kinase N1	ENSMUSG00000057672	29418	21,24
P2ry6	pyrimidinergic receptor P2Y, G-protein coupled, 6	ENSMUSG00000048779	26754	21,24
Naa10	N(alpha)-acetyltransferase 10, NatA catalytic subunit	ENSMUSG00000031388	5072	21,17
Magee1	melanoma antigen, family E, 1	ENSMUSG00000031227	3514	21,17
Cfl2	cofilin 2, muscle	ENSMUSG00000062929	4061	21,17

Dbt	dihydrolipoamide branched chain transacylase E2	ENSMUSG00000000340	36907	21,17
Abcf3	ATP-binding cassette, sub-family F (GCN20), member 3	ENSMUSG00000003234	12803	21,17
Hs3st3b1	heparan sulfate (glucosamine) 3-O-sulfotransferase 3B1	ENSMUSG00000070407	36499	21,17
Slc25a32	solute carrier family 25, member 32	ENSMUSG00000022299	18526	21,17
Lgals3bp	lectin, galactoside-binding, soluble, 3 binding protein	ENSMUSG00000033880	9342	21,17
Arhgap10	Rho GTPase activating protein 10	ENSMUSG00000037148	267542	21,17
		ENSMUSG00000071392	81869	21,17
Muc1	mucin 1, transmembrane	ENSMUSG00000042784	4325	21,17
Gpr63	G protein-coupled receptor 63	ENSMUSG00000040372	42827	21,17
Sft2d3	SFT2 domain containing 3	ENSMUSG00000044982	2810	21,09
Pycr1	pyrroline-5-carboxylate reductase 1	ENSMUSG00000025140	8058	21,09
Emc8	ER membrane protein complex subunit 8	ENSMUSG00000031819	14660	21,09
Dhx33	DEAH (Asp-Glu-Ala-His) box polypeptide 33	ENSMUSG00000040620	20347	21,09
Itga8	integrin alpha 8	ENSMUSG00000026768	195289	21,09
		ENSMUSG00000060098	41859	21,09
Igsf5	immunoglobulin superfamily, member 5	ENSMUSG00000000159	163913	21,09
Mitf	microphthalmia-associated transcription factor	ENSMUSG00000035158	214292	21,09
Cxcl1	chemokine (C-X-C motif) ligand 1	ENSMUSG00000029380	1875	21,09
		ENSMUSG00000037827	2259	21,02
B9d1	B9 protein domain 1	ENSMUSG00000001039	7788	21,02
Scg3	secretogranin III	ENSMUSG00000032181	40733	21,02
Bscl2	Bernardinelli-Seip congenital lipodystrophy 2 homolog (human)	ENSMUSG00000071657	11217	21,02
Snx8	sorting nexin 8	ENSMUSG00000029560	48948	21,02
Tmco3	transmembrane and coiled-coil domains 3	ENSMUSG00000038497	34912	21,02
Mef2b	myocyte enhancer factor 2B	ENSMUSG00000079033	14711	20,95
Ftsj3	FtsJ homolog 3 (E. coli)	ENSMUSG00000020706	6938	20,95
		ENSMUSG00000021340	47602	20,95
Zfp12	zinc finger protein 12	ENSMUSG00000029587	13672	20,95
Brd9	bromodomain containing 9	ENSMUSG00000057649	23057	20,95
Zfp788	zinc finger protein 788	ENSMUSG00000074165	18332	20,95
Tinf2	Terf1 (TRF1)-interacting nuclear factor 2	ENSMUSG00000007589	2735	20,95
Sar1b	SAR1 gene homolog B (S. cerevisiae)	ENSMUSG00000020386	28239	20,95
Fbxo18	F-box protein 18	ENSMUSG00000058594	35010	20,95
Fam53b	family with sequence similarity 53, member B	ENSMUSG00000030956	101805	20,95
Ccdc15	coiled-coil domain containing 15	ENSMUSG00000034303	72558	20,95
Fat3	FAT tumor suppressor homolog 3 (Drosophila)	ENSMUSG00000074505	468027	20,95
Tmem67	transmembrane protein 67	ENSMUSG00000049488	50666	20,95
Rapgef5	Rap guanine nucleotide exchange factor (GEF) 5	ENSMUSG00000041992	240500	20,95
Zfyve21	zinc finger, FYVE domain containing 21	ENSMUSG00000021286	14219	20,88
Cenpb	centromere protein B	ENSMUSG00000068267	2722	20,88

Zfp335	zinc finger protein 335	ENSMUSG00000039834	19876	20,88
Exoc7	exocyst complex component 7	ENSMUSG00000020792	19233	20,88
Cntn2	contactin 2	ENSMUSG00000053024	33516	20,88
Tenc1	tensin like C1 domain-containing phosphatase	ENSMUSG00000037003	13414	20,88
Bcorl1	BCL6 co-repressor-like 1	ENSMUSG00000036959	66692	20,88
Ptprr	protein tyrosine phosphatase, receptor type, R	ENSMUSG00000020151	256720	20,88
Tmem108	transmembrane protein 108	ENSMUSG00000042757	278902	20,88
Mfsd5	major facilitator superfamily domain containing 5	ENSMUSG00000045665	2289	20,80
Elp6	elongator acetyltransferase complex subunit 6	ENSMUSG00000054836	16901	20,80
Nudt16	nudix (nucleoside diphosphate linked moiety X)-type motif 16	ENSMUSG00000032565	2468	20,80
Pex26	peroxisomal biogenesis factor 26	ENSMUSG00000067825	15171	20,80
Babam1	BRISC and BRCA1 A complex member 1	ENSMUSG00000031820	7918	20,80
		ENSMUSG00000028893	18470	20,80
Ap5z1	adaptor-related protein complex 5, zeta 1 subunit	ENSMUSG00000039623	14785	20,80
Adrm1	adhesion regulating molecule 1	ENSMUSG00000039041	4802	20,80
Iqub	IQ motif and ubiquitin domain containing	ENSMUSG00000046192	70203	20,80
Nufip1	nuclear fragile X mental retardation protein interacting protein 1	ENSMUSG00000022009	26489	20,80
Wdr19	WD repeat domain 19	ENSMUSG00000037890	60720	20,80
Moxd1	monooxygenase, DBH-like 1	ENSMUSG00000020000	79274	20,80
Ablim2	actin-binding LIM protein 2	ENSMUSG00000029095	127094	20,80
Tgfbr3	transforming growth factor, beta receptor III	ENSMUSG00000029287	183060	20,80
Sult2b1	sulfotransferase family, cytosolic, 2B, member 1	ENSMUSG00000003271	29630	20,80
Mknk1	MAP kinase-interacting serine/threonine kinase 1	ENSMUSG00000028708	40053	20,80
Abtb2	ankyrin repeat and BTB (POZ) domain containing 2	ENSMUSG00000032724	152114	20,80
Ptger2	prostaglandin E receptor 2 (subtype EP2)	ENSMUSG00000037759	15626	20,80
Lrrc4	leucine rich repeat containing 4	ENSMUSG00000049939	169917	20,80
Tec	tec protein tyrosine kinase	ENSMUSG00000029217	112766	20,80
Tppp	tubulin polymerization promoting protein	ENSMUSG00000021573	26347	20,73
Letmd1	LETM1 domain containing 1	ENSMUSG00000037353	10219	20,73
		ENSMUSG00000096221	2080	20,73
		ENSMUSG00000005374	14217	20,73
Slc25a38	solute carrier family 25, member 38	ENSMUSG00000032519	14131	20,73
Fut10	fucosyltransferase 10	ENSMUSG00000046152	74408	20,73
Atp5sl	ATP5S-like	ENSMUSG00000057229	6138	20,73
Taf6	TAF6 RNA polymerase II, TATA box binding protein (TBP)-associated factor	ENSMUSG00000036980	8835	20,73
Parn	poly(A)-specific ribonuclease (deadenylation nuclease)	ENSMUSG00000022685	130207	20,73
Rbm41	RNA binding motif protein 41	ENSMUSG00000031433	109085	20,73

Rfx2	regulatory factor X, 2 (influences HLA class II expression)	ENSMUSG00000024206	55112	20,73
Smim3	small integral membrane protein 3	ENSMUSG00000038059	27791	20,73
Tsen15	tRNA splicing endonuclease 15 homolog (S. cerevisiae)	ENSMUSG00000014980	15954	20,66
Tbc1d10b	TBC1 domain family, member 10b	ENSMUSG00000042492	11004	20,66
Mrpl9	mitochondrial ribosomal protein L9	ENSMUSG00000028140	7813	20,66
Bmp3	bone morphogenetic protein 3	ENSMUSG00000029335	26522	20,66
		ENSMUSG00000040009	49731	20,66
Ntmt1	N-terminal Xaa-Pro-Lys N-methyltransferase 1	ENSMUSG00000026857	15208	20,66
Vps52	vacuolar protein sorting 52 (yeast)	ENSMUSG00000024319	11173	20,66
Bms1	BMS1 homolog, ribosome assembly protein (yeast)	ENSMUSG00000030138	36035	20,66
Cox18	cytochrome c oxidase assembly protein 18	ENSMUSG00000035505	9278	20,66
Col26a1	collagen, type XXVI, alpha 1	ENSMUSG00000004415	141451	20,66
Glis2	GLIS family zinc finger 2	ENSMUSG00000014303	30212	20,66
Prkd3	protein kinase D3	ENSMUSG00000024070	71412	20,66
Tmem51	transmembrane protein 51	ENSMUSG00000040616	53313	20,66
Rassf9	Ras association (RalGDS/AF-6) domain family (N-terminal) member 9	ENSMUSG00000044921	34339	20,66
Rwdd1	RWD domain containing 1	ENSMUSG00000019782	23070	20,59
Mtmr11	myotubularin related protein 11	ENSMUSG00000045934	9715	20,59
Pfn2	profilin 2	ENSMUSG00000027805	5670	20,59
Kdelr2	KDEL (Lys-Asp-Glu-Leu) endoplasmic reticulum protein retention receptor 2	ENSMUSG00000079111	18081	20,59
Zfp617	zinc finger protein 617	ENSMUSG00000066880	25995	20,59
Gpx1	glutathione peroxidase 1	ENSMUSG00000063856	1259	20,59
Zfp35	zinc finger protein 35	ENSMUSG00000063281	15745	20,59
Zkscan8	zinc finger with KRAB and SCAN domains 8	ENSMUSG00000063894	17899	20,59
Nelfe	negative elongation factor complex member E, Rdbp	ENSMUSG00000024369	5982	20,59
Mzt1	mitotic spindle organizing protein 1	ENSMUSG00000033186	11593	20,59
Esyt1	extended synaptotagmin-like protein 1	ENSMUSG00000025366	15612	20,59
Uevld	UEV and lactate/malate dehyrogenase domains	ENSMUSG00000043262	34989	20,59
		ENSMUSG00000055951	23564	20,59
Nudt9	nudix (nucleoside diphosphate linked moiety X)-type motif 9	ENSMUSG00000029310	19074	20,51
Tpx2	TPX2, microtubule-associated protein homolog (Xenopus laevis)	ENSMUSG00000027469	47358	20,51
Abhd5	abhydrolase domain containing 5	ENSMUSG00000032540	29917	20,51
Poldip2	polymerase (DNA-directed), delta interacting protein 2	ENSMUSG00000001100	10544	20,51
Desi1	desumoylating isopeptidase 1	ENSMUSG00000022472	41340	20,51
		ENSMUSG00000055026	670724	20,51
		ENSMUSG00000020019	106527	20,51
Adora1	adenosine A1 receptor	ENSMUSG00000042429	36209	20,51
Iqcb1	IQ calmodulin-binding motif containing 1	ENSMUSG00000022837	44337	20,51
2610028H24Rik	RIKEN cDNA 2610028H24 gene	ENSMUSG00000009114	12030	20,51

Pbk	PDZ binding kinase	ENSMUSG00000022033	11986	20,44
Cdc42ep2	CDC42 effector protein (Rho GTPase binding) 2	ENSMUSG00000045664	7261	20,44
Rps6kb2	ribosomal protein S6 kinase, polypeptide 2	ENSMUSG00000024830	6642	20,44
		ENSMUSG00000063897	20303	20,44
		ENSMUSG00000097721	6642	20,44
Lrrn3	leucine rich repeat protein 3, neuronal	ENSMUSG00000036295	34079	20,44
Hs3st3a1	heparan sulfate (glucosamine) 3-O-sulfotransferase 3A1	ENSMUSG00000047759	87510	20,44
3300002I08Rik	RIKEN cDNA 3300002I08 gene	ENSMUSG00000063364	51831	20,44
Mmaa	methylmalonic aciduria (cobalamin deficiency) type A	ENSMUSG00000037022	28533	20,44
Tssc4	tumor-suppressing subchromosomal transferable fragment 4	ENSMUSG00000045752	1845	20,37
Kat5	K(lysine) acetyltransferase 5	ENSMUSG00000024926	7078	20,37
Ccdc64b	coiled-coil domain containing 64B	ENSMUSG00000043782	8088	20,37
Txnrd3	thioredoxin reductase 3	ENSMUSG00000000811	31542	20,37
Znrd1as	Znrd1 antisense	ENSMUSG00000036214	7031	20,37
Sucla2	succinate-Coenzyme A ligase, ADP-forming, beta subunit	ENSMUSG00000022110	70824	20,37
Slc5a9	solute carrier family 5 (sodium/glucose cotransporter), member 9	ENSMUSG00000028544	27544	20,37
Zfp810	zinc finger protein 810	ENSMUSG00000066829	30891	20,37
Sncaip	synuclein, alpha interacting protein (synphilin)	ENSMUSG00000024534	148227	20,37
Dars	aspartyl-tRNA synthetase	ENSMUSG00000026356	53709	20,37
Hspb6	heat shock protein, alpha-crystallin-related, B6	ENSMUSG00000036854	3266	20,37
Mtpap	mitochondrial poly(A) polymerase	ENSMUSG00000024234	21739	20,37
Llgl2	lethal giant larvae homolog 2 (Drosophila)	ENSMUSG00000020782	31732	20,37
Crocc	ciliary rootlet coiled-coil, rootletin	ENSMUSG00000040860	43914	20,37
		ENSMUSG00000047649	2635	20,30
Fam219b	family with sequence similarity 219, member B	ENSMUSG00000032305	5660	20,30
		ENSMUSG00000057762	1060	20,30
Diexf	digestive organ expansion factor homolog (zebrafish)	ENSMUSG00000016181	25849	20,30
		ENSMUSG00000070476	9905	20,30
2810408A11Rik	RIKEN cDNA 2810408A11 gene	ENSMUSG00000018570	3636	20,30
Zhx1	zinc fingers and homeoboxes 1	ENSMUSG00000022361	29539	20,30
Mrpl1	mitochondrial ribosomal protein L1	ENSMUSG00000029486	57235	20,30
2310061I04Rik	RIKEN cDNA 2310061I04 gene	ENSMUSG00000050705	4755	20,22
Mzt2	mitotic spindle organizing protein 2	ENSMUSG00000022671	14929	20,22
Zfp790	zinc finger protein 790	ENSMUSG00000011427	15971	20,22
Gmpr2	guanosine monophosphate reductase 2	ENSMUSG00000002326	6516	20,22
Ifi27	interferon, alpha-inducible protein 27	ENSMUSG00000064215	6029	20,22
Ntpcr	nucleoside-triphosphatase, cancer-related	ENSMUSG00000031851	18273	20,22

Wbscr27	Williams Beuren syndrome chromosome region 27 (human)	ENSMUSG00000040557	10270	20,22
Lrp11	low density lipoprotein receptor-related protein 11	ENSMUSG00000019796	35684	20,22
Qtrt1	queuine tRNA-ribosyltransferase 1	ENSMUSG00000002825	8441	20,22
Kif20b	kinesin family member 20B	ENSMUSG00000024795	53374	20,22
Utp14b	UTP14, U3 small nucleolar ribonucleoprotein, homolog B (yeast)	ENSMUSG00000079470	9551	20,22
Fam71a	family with sequence similarity 71, member A	ENSMUSG00000091017	2234	20,22
Ak1	adenylate kinase 1	ENSMUSG00000026817	25048	20,15
		ENSMUSG00000036850	982	20,15
Fbxo5	F-box protein 5	ENSMUSG00000019773	6441	20,15
Rnf31	ring finger protein 31	ENSMUSG00000047098	11986	20,15
Ccdc61	coiled-coil domain containing 61	ENSMUSG00000074358	19533	20,15
Bcl7c	B cell CLL/lymphoma 7C	ENSMUSG00000030814	45581	20,15
Spc24	SPC24, NDC80 kinetochore complex component, homolog (S. cerevisiae)	ENSMUSG00000074476	4845	20,15
		ENSMUSG00000026627	25003	20,15
Syn1	synapsin I	ENSMUSG00000037217	60494	20,15
Tatdn2	TatD DNase domain containing 2	ENSMUSG00000056952	14020	20,15
Slco5a1	solute carrier organic anion transporter family, member 5A1	ENSMUSG00000025938	124629	20,15
Tbc1d22b	TBC1 domain family, member 22B	ENSMUSG00000042203	57108	20,15
Skil	SKI-like	ENSMUSG00000027660	27520	20,15
Nkain3	Na+/K+ transporting ATPase interacting 3	ENSMUSG00000055761	659993	20,15
Mmrn2	multimerin 2	ENSMUSG00000041445	28784	20,15
Mettl15	methyltransferase like 15	ENSMUSG00000057234	188452	20,15
Bpifb1	BPI fold containing family B, member 1	ENSMUSG00000027485	29552	20,15
Ppib	peptidylprolyl isomerase B	ENSMUSG00000032383	6461	20,08
Psmb3	proteasome (prosome, macropain) subunit, beta type 3	ENSMUSG00000069744	10102	20,08
		ENSMUSG00000028743	7683	20,08
Smg8	smg-8 homolog, nonsense mediated mRNA decay factor (C. elegans)	ENSMUSG00000020495	9043	20,08
Cdo1	cysteine dioxygenase 1, cytosolic	ENSMUSG00000033022	15138	20,08
Stk11ip	serine/threonine kinase 11 interacting protein	ENSMUSG00000026213	15807	20,08
Larp7	La ribonucleoprotein domain family, member 7	ENSMUSG00000027968	16395	20,08
Pof1b	premature ovarian failure 1B	ENSMUSG00000034607	60221	20,08
Nos1	nitric oxide synthase 1, neuronal	ENSMUSG00000029361	177809	20,08
Sorbs3	sorbin and SH3 domain containing 3	ENSMUSG00000022091	27170	20,08
Rcsd1	RCSD domain containing 1	ENSMUSG00000040723	59150	20,08
Gsap	gamma-secretase activating protein	ENSMUSG00000039934	105435	20,08
Dda1	DET1 and DDB1 associated 1	ENSMUSG00000074247	6900	20,01
Flot1	flotillin 1	ENSMUSG00000059714	9562	20,01
Kctd5	potassium channel tetramerisation domain containing 5	ENSMUSG00000016946	25752	20,01

Agl	amylo-1,6-glucosidase, 4-alpha-glucanotransferase	ENSMUSG00000033400	68168	20,01
Phb	prohibitin	ENSMUSG00000038845	13817	20,01
Rcc1	regulator of chromosome condensation 1	ENSMUSG00000028896	21687	20,01
Pmm2	phosphomannomutase 2	ENSMUSG00000022711	19858	20,01
		ENSMUSG00000062646	57805	20,01
Mettl7a2	methyltransferase like 7A2	ENSMUSG00000056487	8641	20,01
March1	membrane-associated ring finger (C3HC4) 1	ENSMUSG00000036469	853738	20,01
Pcnxl2	pecanex-like 2 (Drosophila)	ENSMUSG00000060212	146785	20,01
		ENSMUSG00000091095	5390	20,01
Eps8	epidermal growth factor receptor pathway substrate 8	ENSMUSG00000015766	177632	20,01
		ENSMUSG00000017386	7091	19,93
Ecsit	ECSIT homolog (Drosophila)	ENSMUSG00000066839	13193	19,93
H2afy2	H2A histone family, member Y2	ENSMUSG00000020086	45483	19,93
		ENSMUSG00000055485	63456	19,93
		ENSMUSG00000030431	5062	19,93
Josd1	Josephin domain containing 1	ENSMUSG00000022426	13623	19,93
Dtl	denticleless homolog (Drosophila)	ENSMUSG00000037474	38170	19,93
Bbs2	Bardet-Biedl syndrome 2 (human)	ENSMUSG00000031755	30858	19,93
BC030336	cDNA sequence BC030336	ENSMUSG00000046096	57235	19,93
Sp2	Sp2 transcription factor	ENSMUSG00000018678	29620	19,93
Adcy6	adenylate cyclase 6	ENSMUSG00000022994	17640	19,93
Brca1	breast cancer 1	ENSMUSG00000017146	63192	19,93
Slc1a5	solute carrier family 1 (neutral amino acid transporter), member 5	ENSMUSG00000001918	16929	19,93
Zscan26	zinc finger and SCAN domain containing 26	ENSMUSG00000022228	11556	19,93
Ccdc18	coiled-coil domain containing 18	ENSMUSG00000056531	100034	19,93
Flcn	folliculin	ENSMUSG00000032633	18609	19,93
Agk	acylglycerol kinase	ENSMUSG00000029916	71285	19,93
		ENSMUSG00000015659	37546	19,93
Trpm6	transient receptor potential cation channel, subfamily M, member 6	ENSMUSG00000024727	142528	19,93
Ifi44	interferon-induced protein 44	ENSMUSG00000028037	19026	19,93
		ENSMUSG00000033099	8702	19,86
Mterfd3	MTERF domain containing 3	ENSMUSG00000049038	8593	19,86
Ahcy	S-adenosylhomocysteine hydrolase	ENSMUSG00000027597	15188	19,86
Nde1	nuclear distribution gene E homolog 1 (A nidulans)	ENSMUSG00000022678	29654	19,86
Elovl5	ELOVL family member 5, elongation of long chain fatty acids (yeast)	ENSMUSG00000032349	67156	19,86
		ENSMUSG00000026688	21876	19,86
Tmem191c	transmembrane protein 191C	ENSMUSG00000055692	6930	19,86
		ENSMUSG00000024829	9937	19,86
Fbxl12	F-box and leucine-rich repeat protein 12	ENSMUSG00000066892	26650	19,86
6430550D23Rik	RIKEN cDNA 6430550D23 gene	ENSMUSG00000074646	34699	19,86
Fam184a	family with sequence similarity 184, member A	ENSMUSG00000019856	117765	19,86
Epha4	Eph receptor A4	ENSMUSG00000026235	147904	19,86
Plscr2	phospholipid scramblase 2	ENSMUSG00000032372	22151	19,86

B4galt5	UDP-Gal:betaGlcNAc beta 1,4-galactosyltransferase, polypeptide 5	ENSMUSG00000017929	50740	19,86
Mrpl4	mitochondrial ribosomal protein L4	ENSMUSG00000003299	6099	19,79
Mettl8	methyltransferase like 8	ENSMUSG00000041975	91023	19,79
Rnf122	ring finger protein 122	ENSMUSG00000039328	19663	19,79
Mis18a	MIS18 kinetochore protein homolog A (S. pombe)	ENSMUSG00000022978	8093	19,79
Wdr53	WD repeat domain 53	ENSMUSG00000022787	9857	19,79
		ENSMUSG00000048497	17691	19,79
Nudt19	nudix (nucleoside diphosphate linked moiety X)-type motif 19	ENSMUSG00000034875	9120	19,79
Prg4	proteoglycan 4 (megakaryocyte stimulating factor, articular superficial zone protein)	ENSMUSG00000006014	16754	19,79
Myo1h	myosin 1H	ENSMUSG00000066952	49636	19,79
Zdhhc6	zinc finger, DHHC domain containing 6	ENSMUSG00000024982	17732	19,79
Tceal8	transcription elongation factor A (SII)-like 8	ENSMUSG00000051579	3359	19,79
Zfp703	zinc finger protein 703	ENSMUSG00000085795	4126	19,79
Dok1	docking protein 1	ENSMUSG00000068335	2538	19,79
Dock6	dedicator of cytokinesis 6	ENSMUSG00000032198	52452	19,79
Smad6	SMAD family member 6	ENSMUSG00000036867	68984	19,79
Cdh12	cadherin 12	ENSMUSG00000040452	478082	19,79
		ENSMUSG00000090861	9777	19,72
Papd7	PAP associated domain containing 7	ENSMUSG00000034575	35881	19,72
Coq3	coenzyme Q3 homolog, methyltransferase (yeast)	ENSMUSG00000028247	32490	19,72
Tmem98	transmembrane protein 98	ENSMUSG00000035413	11859	19,72
Snupn	snurportin 1	ENSMUSG00000055334	32329	19,72
Stk33	serine/threonine kinase 33	ENSMUSG00000031027	159859	19,72
P4htm	prolyl 4-hydroxylase, transmembrane (endoplasmic reticulum)	ENSMUSG00000006675	18775	19,72
		ENSMUSG00000049439	44738	19,72
Dera	2-deoxyribose-5-phosphate aldolase homolog (C. elegans)	ENSMUSG00000030225	83350	19,72
Med10	mediator of RNA polymerase II transcription, subunit 10 homolog (NUT2, S. cerevisiae)	ENSMUSG00000021598	6213	19,64
Tbccd1	TBCC domain containing 1	ENSMUSG00000004462	44355	19,64
Mtus2	microtubule associated tumor suppressor candidate 2	ENSMUSG00000029651	358746	19,64
Trim27	tripartite motif-containing 27	ENSMUSG00000021326	15280	19,64
Idnk	idnK gluconokinase homolog (E. coli)	ENSMUSG00000050002	7045	19,64
Snapc4	small nuclear RNA activating complex, polypeptide 4	ENSMUSG00000036281	17889	19,64
		ENSMUSG00000050373	7994	19,64
Ttc26	tetratricopeptide repeat domain 26	ENSMUSG00000056832	46179	19,64
Nucb1	nucleobindin 1	ENSMUSG00000030824	17533	19,64
Lrrc16b	leucine rich repeat containing 16B	ENSMUSG00000022211	17154	19,64
Psmd10	proteasome (prosome, macropain) 26S subunit, non-ATPase, 10	ENSMUSG00000031429	8300	19,64

Nipal3	NIPA-like domain containing 3	ENSMUSG00000028803	48902	19,64
Slc25a21	solute carrier family 25 (mitochondrial oxodicarboxylate carrier), member 21	ENSMUSG00000035472	484839	19,64
Fam78b	family with sequence similarity 78, member B	ENSMUSG00000060568	89886	19,64
D3Ertd751e	DNA segment, Chr 3, ERATO Doi 751, expressed	ENSMUSG00000025766	16779	19,64
		ENSMUSG00000068011	9033	19,64
Gli3	GLI-Kruppel family member GLI3	ENSMUSG00000021318	266792	19,64
Susd1	sushi domain containing 1	ENSMUSG00000038578	123951	19,64
Scamp4	secretory carrier membrane protein 4	ENSMUSG00000078441	12902	19,57
Commd5	COMM domain containing 5	ENSMUSG00000055041	1396	19,57
Stub1	STIP1 homology and U-Box containing protein 1	ENSMUSG00000039615	2726	19,57
Ttc18	tetratricopeptide repeat domain 18	ENSMUSG00000039543	58037	19,57
Gpatch11	G patch domain containing 11	ENSMUSG00000050668	12784	19,57
Rab4a	RAB4A, member RAS oncogene family	ENSMUSG00000019478	29303	19,57
Rab23	RAB23, member RAS oncogene family	ENSMUSG00000004768	22683	19,57
Cd59a	CD59a antigen	ENSMUSG00000032679	19554	19,57
Sema6d	sema domain, transmembrane domain (TM), and cytoplasmic domain, (semaphorin) 6D	ENSMUSG00000027200	577802	19,57
Ero1l	ERO1-like (S. cerevisiae)	ENSMUSG00000021831	35481	19,57
Tmem53	transmembrane protein 53	ENSMUSG00000048772	16632	19,57
Alg13	asparagine-linked glycosylation 13	ENSMUSG00000041718	56639	19,57
Stil	Scl/Tal1 interrupting locus	ENSMUSG00000028718	43038	19,57
Phka2	phosphorylase kinase alpha 2	ENSMUSG00000031295	96713	19,57
Tsr3	TSR3 20S rRNA accumulation	ENSMUSG00000015126	2629	19,50
Sf3b4	splicing factor 3b, subunit 4	ENSMUSG00000068856	5233	19,50
Ddx47	DEAD (Asp-Glu-Ala-Asp) box polypeptide 47	ENSMUSG00000030204	12165	19,50
Gm10146	predicted gene 10146	ENSMUSG00000064317	448	19,50
Abcb8	ATP-binding cassette, sub-family B (MDR/TAP), member 8	ENSMUSG00000028973	16392	19,50
Lrrn1	leucine rich repeat protein 1, neuronal	ENSMUSG00000034648	40503	19,50
Hip1r	huntingtin interacting protein 1 related	ENSMUSG00000000915	29584	19,50
Snapc1	small nuclear RNA activating complex, polypeptide 1	ENSMUSG00000021113	20291	19,50
Lbp	lipopolysaccharide binding protein	ENSMUSG00000016024	26360	19,50
Nagk	N-acetylglucosamine kinase	ENSMUSG00000034744	8439	19,50
Mylip	myosin regulatory light chain interacting protein	ENSMUSG00000038175	22278	19,50
Ddx31	DEAD/H (Asp-Glu-Ala-Asp/His) box polypeptide 31	ENSMUSG00000026806	65166	19,50
		ENSMUSG00000075270	349634	19,50
Mms22l	MMS22-like, DNA repair protein	ENSMUSG00000045751	106500	19,50
Rnls	renalase, FAD-dependent amine oxidase	ENSMUSG00000071573	254549	19,50
Sptlc3	serine palmitoyltransferase, long chain base subunit 3	ENSMUSG00000039092	143762	19,50

		ENSMUSG00000040502	5174	19,43
Tubg1	tubulin, gamma 1	ENSMUSG00000035198	6482	19,43
2810006K23Rik	RIKEN cDNA 2810006K23 gene	ENSMUSG00000047635	13756	19,43
Mynn	myoneurin	ENSMUSG00000037730	16406	19,43
Engase	endo-beta-N-acetylglucosaminidase	ENSMUSG00000033857	12381	19,43
		ENSMUSG00000028614	48563	19,43
Whamm	WAS protein homolog associated with actin, golgi membranes and microtubules	ENSMUSG00000045795	25545	19,43
2610301B20Rik	RIKEN cDNA 2610301B20 gene	ENSMUSG00000059482	24928	19,43
		ENSMUSG00000090639	40100	19,43
Yipf6	Yip1 domain family, member 6	ENSMUSG00000047694	12702	19,35
		ENSMUSG00000041134	131865	19,35
Ltv1	LTV1 homolog (S. cerevisiae)	ENSMUSG00000019814	14498	19,35
Vac14	Vac14 homolog (S. cerevisiae)	ENSMUSG00000010936	101759	19,35
Cryzl1	crystallin, zeta (quinone reductase)-like 1	ENSMUSG00000058240	39654	19,35
Mecr	mitochondrial trans-2-enoyl-CoA reductase	ENSMUSG00000028910	24317	19,35
Clk2	CDC-like kinase 2	ENSMUSG00000068917	12127	19,35
Grtp1	GH regulated TBC protein 1	ENSMUSG00000038515	23756	19,35
Ngly1	N-glycanase 1	ENSMUSG00000021785	62647	19,35
		ENSMUSG00000028096	37066	19,35
Nup205	nucleoporin 205	ENSMUSG00000038759	70161	19,35
Ints4	integrator complex subunit 4	ENSMUSG00000025133	60442	19,35
Dcpp2	demilune cell and parotid protein 2	ENSMUSG00000096278	19908	19,28
		ENSMUSG00000074102	1361	19,28
Cited2	Cbp/p300-interacting transactivator, with Glu/Asp-rich carboxy-terminal domain, 2	ENSMUSG00000039910	2446	19,28
Igdcc3	immunoglobulin superfamily, DCC subclass, member 3	ENSMUSG00000032394	44704	19,28
St18	suppression of tumorigenicity 18	ENSMUSG00000033740	373710	19,28
Nme5	NME/NM23 family member 5	ENSMUSG00000035984	16482	19,28
Pigp	phosphatidylinositol glycan anchor biosynthesis, class P	ENSMUSG00000022940	12253	19,28
		ENSMUSG00000025439	22246	19,28
Galk1	galactokinase 1	ENSMUSG00000020766	4263	19,28
Nfxl1	nuclear transcription factor, X-box binding-like 1	ENSMUSG00000072889	46384	19,28
Sorcs1	VPS10 domain receptor protein SORCS 1	ENSMUSG00000043531	535346	19,28
Nars2	asparaginyl-tRNA synthetase 2 (mitochondrial)(putative)	ENSMUSG00000018995	113257	19,28
Kcnab1	potassium voltage-gated channel, shaker-related subfamily, beta member 1	ENSMUSG00000027827	268840	19,28
		ENSMUSG00000092116	2654	19,21
Adat3	adenosine deaminase, tRNA-specific 3	ENSMUSG00000035370	12904	19,21
Cep19	centrosomal protein 19	ENSMUSG00000035790	8270	19,21
Lama2	laminin, alpha 2	ENSMUSG00000019899	635655	19,21
Esco2	establishment of cohesion 1 homolog 2 (S. cerevisiae)	ENSMUSG00000022034	14957	19,21
Kcnip1	Kv channel-interacting protein 1	ENSMUSG00000053519	363814	19,21

Sox6	SRY-box containing gene 6	ENSMUSG00000051910	567401	19,21
Fut2	fucosyltransferase 2	ENSMUSG00000055978	17714	19,21
Ppp6r2	protein phosphatase 6, regulatory subunit 2	ENSMUSG00000036561	33227	19,21
		ENSMUSG00000049411	140178	19,21
Zfyve1	zinc finger, FYVE domain containing 1	ENSMUSG00000042628	50207	19,21
		ENSMUSG00000027374	19608	19,14
Ppp2r5d	protein phosphatase 2, regulatory subunit B (B56), delta isoform	ENSMUSG00000059409	22012	19,14
1810009A15Rik	RIKEN cDNA 1810009A15 gene	ENSMUSG00000071653	1838	19,14
		ENSMUSG00000030337	28993	19,14
Tmem39b	transmembrane protein 39b	ENSMUSG00000053730	20484	19,14
Meig1	meiosis expressed gene 1	ENSMUSG00000026650	13606	19,14
Trim46	tripartite motif-containing 46	ENSMUSG00000042766	12133	19,14
1110004E09Rik	RIKEN cDNA 1110004E09 gene	ENSMUSG00000022972	9119	19,14
		ENSMUSG00000051412	28411	19,14
Ctc1	CTS telomere maintenance complex component 1	ENSMUSG00000020898	20563	19,14
		ENSMUSG00000007946	4854	19,14
Wdr76	WD repeat domain 76	ENSMUSG00000027242	38138	19,14
4921524J17Rik	RIKEN cDNA 4921524J17 gene	ENSMUSG00000036934	24083	19,14
Ifnlr1	interferon lambda receptor 1	ENSMUSG00000062157	21895	19,14
		ENSMUSG00000028744	16680	19,14
Slc35a1	solute carrier family 35 (CMP-sialic acid transporter), member 1	ENSMUSG00000028293	24182	19,14
		ENSMUSG00000025949	87263	19,14
Il1rap	interleukin 1 receptor accessory protein	ENSMUSG00000022514	148414	19,14
Rbm24	RNA binding motif protein 24	ENSMUSG00000038132	12791	19,06
B3gnt1	UDP-GlcNAc:betaGal beta-1,3-N-acetylglucosaminyltransferase 1	ENSMUSG00000047379	2309	19,06
		ENSMUSG00000091383	378	19,06
E2f2	E2F transcription factor 2	ENSMUSG00000018983	23664	19,06
Gas2l1	growth arrest-specific 2 like 1	ENSMUSG00000034201	11196	19,06
Rbm17	RNA binding motif protein 17	ENSMUSG00000037197	18715	19,06
		ENSMUSG00000037553	44856	19,06
Pusl1	pseudouridylate synthase-like 1	ENSMUSG00000051557	3903	19,06
Cchcr1	coiled-coil alpha-helical rod protein 1	ENSMUSG00000040312	13916	19,06
Pafah1b3	platelet-activating factor acetylhydrolase, isoform 1b, subunit 3	ENSMUSG00000005447	2938	19,06
C87436	expressed sequence C87436	ENSMUSG00000046679	35127	19,06
N6amt2	N-6 adenine-specific DNA methyltransferase 2 (putative)	ENSMUSG00000021951	21972	19,06
Wdr36	WD repeat domain 36	ENSMUSG00000038299	29196	19,06
Mfsd8	major facilitator superfamily domain containing 8	ENSMUSG00000025759	28816	19,06
Cntrob	centrobin, centrosomal BRCA2 interacting protein	ENSMUSG00000032782	24289	19,06
2700050L05Rik	RIKEN cDNA 2700050L05 gene	ENSMUSG00000039990	35429	19,06
Rad52	RAD52 homolog (S. cerevisiae)	ENSMUSG00000030166	20131	19,06
Cacna1e	calcium channel, voltage-dependent, R type, alpha 1E subunit	ENSMUSG00000004110	333402	19,06

Siva1	SIVA1, apoptosis-inducing factor	ENSMUSG00000064326	4325	18,99
Dlgap3	discs, large (Drosophila) homolog-associated protein 3	ENSMUSG00000042388	67819	18,99
Sssca1	Sjogren's syndrome/scleroderma autoantigen 1 homolog (human)	ENSMUSG00000079478	3645	18,99
		ENSMUSG00000001418	6291	18,99
Dusp22	dual specificity phosphatase 22	ENSMUSG00000069255	51233	18,99
Mcee	methylmalonyl CoA epimerase	ENSMUSG00000033429	19477	18,99
Cdc7	cell division cycle 7 (S. cerevisiae)	ENSMUSG00000029283	20111	18,99
Caprin2	caprin family member 2	ENSMUSG00000030309	53746	18,99
Ndor1	NADPH dependent diflavin oxidoreductase 1	ENSMUSG00000006471	11202	18,99
Ovol2	ovo-like 2 (Drosophila)	ENSMUSG00000037279	26972	18,99
Cep57l1	centrosomal protein 57-like 1	ENSMUSG00000019813	90768	18,99
Hpgds	hematopoietic prostaglandin D synthase	ENSMUSG00000029919	27616	18,99
Mvk	mevalonate kinase	ENSMUSG00000041939	16323	18,92
Zdhhc12	zinc finger, DHHC domain containing 12	ENSMUSG00000015335	2705	18,92
Rnf181	ring finger protein 181	ENSMUSG00000055850	3008	18,92
B3galtl	beta 1,3-galactosyltransferase-like	ENSMUSG00000051950	84370	18,92
Fez2	fasciculation and elongation protein zeta 2 (zygin II)	ENSMUSG00000056121	40247	18,92
		ENSMUSG00000041716	16941	18,92
Itfg2	integrin alpha FG-GAP repeat containing 2	ENSMUSG00000001518	15488	18,92
Prtn3	proteinase 3	ENSMUSG00000057729	8699	18,92
		ENSMUSG00000045160	10990	18,92
Zfp568	zinc finger protein 568	ENSMUSG00000074221	44328	18,92
Stx6	syntaxin 6	ENSMUSG00000026470	44815	18,92
		ENSMUSG00000028085	81504	18,92
Ttc27	tetratricopeptide repeat domain 27	ENSMUSG00000024078	145821	18,92
Zfp346	zinc finger protein 346	ENSMUSG00000021481	29515	18,92
		ENSMUSG00000008999	72310	18,85
Supt7l	suppressor of Ty 7-like	ENSMUSG00000053134	13239	18,85
Coq9	coenzyme Q9 homolog (yeast)	ENSMUSG00000031782	16575	18,85
		ENSMUSG00000026115	33762	18,85
		ENSMUSG00000024944	7033	18,85
Zfp330	zinc finger protein 330	ENSMUSG00000031711	10540	18,85
Pex12	peroxisomal biogenesis factor 12	ENSMUSG00000018733	7945	18,85
Xlr5a	X-linked lymphocyte-regulated 5A	ENSMUSG00000058328	185342	18,85
Fam50a	family with sequence similarity 50, member A	ENSMUSG00000001962	7117	18,85
Poc1a	POC1 centriolar protein homolog A (Chlamydomonas)	ENSMUSG00000023345	68831	18,85
Pdxk	pyridoxal (pyridoxine, vitamin B6) kinase	ENSMUSG00000032788	28232	18,85
Thoc5	THO complex 5	ENSMUSG00000034274	33548	18,85
Erlin1	ER lipid raft associated 1	ENSMUSG00000025198	34842	18,85
		ENSMUSG00000033705	102772	18,85
Cbwd1	COBW domain containing 1	ENSMUSG00000024878	41701	18,85
Tmc6	transmembrane channel-like gene family 6	ENSMUSG00000025572	16211	18,85
Vamp5	vesicle-associated membrane protein 5	ENSMUSG00000073002	12420	18,85

Asic1	acid-sensing (proton-gated) ion channel 1	ENSMUSG00000023017	30410	18,77
Vsnl1	visinin-like 1	ENSMUSG00000054459	111403	18,77
Cerkl	ceramide kinase-like	ENSMUSG00000075256	126243	18,77
Nov	nephroblastoma overexpressed gene	ENSMUSG00000037362	8338	18,77
Ppp2r5b	protein phosphatase 2, regulatory subunit B (B56), beta isoform	ENSMUSG00000024777	8108	18,77
Ndufaf7	NADH dehydrogenase (ubiquinone) 1 alpha subcomplex assembly factor 7	ENSMUSG00000024082	10918	18,77
Mpst	mercaptopyruvate sulfurtransferase	ENSMUSG00000071711	7291	18,77
Fiz1	Flt3 interacting zinc finger protein 1	ENSMUSG00000061374	7641	18,77
Dpysl4	dihydropyrimidinase-like 4	ENSMUSG00000025478	16704	18,70
Plxna3	plexin A3	ENSMUSG00000031398	15624	18,70
Fbxl19	F-box and leucine-rich repeat protein 19	ENSMUSG00000030811	22153	18,70
Mvb12a	multivesicular body subunit 12A	ENSMUSG00000031813	5095	18,70
Dus2l	dihydrouridine synthase 2-like (SMM1, S. cerevisiae)	ENSMUSG00000031901	62504	18,70
2010015L04Rik	RIKEN cDNA 2010015L04 gene	ENSMUSG00000042233	38755	18,70
Scarb2	scavenger receptor class B, member 2	ENSMUSG00000029426	61736	18,70
Gas2	growth arrest specific 2	ENSMUSG00000030498	132961	18,70
		ENSMUSG00000042213	66077	18,70
Fig4	FIG4 homolog (S. cerevisiae)	ENSMUSG00000038417	115070	18,70
Zc3h12c	zinc finger CCCH type containing 12C	ENSMUSG00000035164	56127	18,70
BC051019	cDNA sequence BC051019	ENSMUSG00000031022	11670	18,70
Nkiras2	NFKB inhibitor interacting Ras-like protein 2	ENSMUSG00000017837	8364	18,63
Endog	endonuclease G	ENSMUSG00000015337	2577	18,63
Mospd3	motile sperm domain containing 3	ENSMUSG00000037221	4414	18,63
Rwdd4a	RWD domain containing 4A	ENSMUSG00000031568	19292	18,63
Inppl1	inositol polyphosphate phosphatase-like 1	ENSMUSG00000032737	15594	18,63
Harbi1	harbinger transposase derived 1	ENSMUSG00000027243	10694	18,63
Arhgef16	Rho guanine nucleotide exchange factor (GEF) 16	ENSMUSG00000029032	23191	18,63
Slc35f3	solute carrier family 35, member F3	ENSMUSG00000057060	96925	18,63
Kcnj1	potassium inwardly-rectifying channel, subfamily J, member 1	ENSMUSG00000041248	26775	18,63
Mapkapk3	mitogen-activated protein kinase-activated protein kinase 3	ENSMUSG00000032577	34951	18,63
Mfge8	milk fat globule-EGF factor 8 protein	ENSMUSG00000030605	15292	18,56
Slc6a8	solute carrier family 6 (neurotransmitter transporter, creatine), member 8	ENSMUSG00000019558	9353	18,56
Fgd2	FYVE, RhoGEF and PH domain containing 2	ENSMUSG00000024013	18747	18,56
Zc3h7b	zinc finger CCCH type containing 7B	ENSMUSG00000022390	51422	18,56

Slc36a1	solute carrier family 36 (proton/amino acid symporter), member 1	ENSMUSG00000020261	31981	18,56
Ddx20	DEAD (Asp-Glu-Ala-Asp) box polypeptide 20	ENSMUSG00000027905	9305	18,56
Tmem242	transmembrane protein 242	ENSMUSG00000004945	29397	18,56
Pcdh1	protocadherin 1	ENSMUSG00000051375	26139	18,56
Adck3	aarF domain containing kinase 3	ENSMUSG00000026489	34365	18,56
Decr1	2,4-dienoyl CoA reductase 1, mitochondrial	ENSMUSG00000028223	28268	18,56
AI661453	expressed sequence AI661453	ENSMUSG00000034382	34024	18,56
Trpm2	transient receptor potential cation channel, subfamily M, member 2	ENSMUSG00000009292	62842	18,56
Lin9	lin-9 homolog (C. elegans)	ENSMUSG00000058729	49369	18,56
Dapl1	death associated protein-like 1	ENSMUSG00000026989	20368	18,56
Mei4	meiosis-specific, MEI4 homolog (S. cerevisiae)	ENSMUSG00000043289	342338	18,56
Ercc2	excision repair cross-complementing rodent repair deficiency, complementation group 2	ENSMUSG00000030400	13685	18,49
		ENSMUSG00000028243	30591	18,49
Dixdc1	DIX domain containing 1	ENSMUSG00000032064	76766	18,49
Zfp763	zinc finger protein 763	ENSMUSG00000067430	16540	18,49
Clasrp	CLK4-associating serine/arginine rich protein	ENSMUSG00000061028	23425	18,49
Poln	DNA polymerase N	ENSMUSG00000045102	162251	18,49
Tm4sf1	transmembrane 4 superfamily member 1	ENSMUSG00000027800	16309	18,49
Ybey	ybeY metallopeptidase	ENSMUSG00000033126	9546	18,49
Trmt13	tRNA methyltransferase 13	ENSMUSG00000033439	33495	18,49
Rps6ka1	ribosomal protein S6 kinase polypeptide 1	ENSMUSG00000003644	40508	18,49
Pde10a	phosphodiesterase 10A	ENSMUSG00000023868	461277	18,49
		ENSMUSG00000025921	27961	18,49
Parp9	poly (ADP-ribose) polymerase family, member 9	ENSMUSG00000022906	34136	18,49
Rgs2	regulator of G-protein signaling 2	ENSMUSG00000026360	4824	18,41
		ENSMUSG00000027498	11768	18,41
Nudt14	nudix (nucleoside diphosphate linked moiety X)-type motif 14	ENSMUSG00000002804	7386	18,41
		ENSMUSG00000095406	819	18,41
Vars2	valyl-tRNA synthetase 2, mitochondrial (putative)	ENSMUSG00000038838	11959	18,41
Prorsd1	prolyl-tRNA synthetase domain containing 1	ENSMUSG00000032673	3277	18,41
Pdcd6	programmed cell death 6	ENSMUSG00000021576	14206	18,41
Ankrd27	ankyrin repeat domain 27 (VPS9 domain)	ENSMUSG00000034867	52991	18,41
Cenpm	centromere protein M	ENSMUSG00000068101	10969	18,41
		ENSMUSG00000079286	15169	18,41
Ak8	adenylate kinase 8	ENSMUSG00000026807	113002	18,41
Fancm	Fanconi anemia, complementation group M	ENSMUSG00000055884	56453	18,41
Atg3	autophagy related 3	ENSMUSG00000022663	29754	18,41
Pex1	peroxisomal biogenesis factor 1	ENSMUSG00000005907	41167	18,41

		ENSMUSG00000050122	50401	18,41
Rerg	RAS-like, estrogen-regulated, growth-inhibitor	ENSMUSG00000030222	115673	18,41
Fam188b	family with sequence similarity 188, member B	ENSMUSG00000038022	116840	18,41
		ENSMUSG00000041624	364989	18,41
Nim1	serine/threonine-protein kinase NIM1	ENSMUSG00000095930	45789	18,41
Selm	selenoprotein M	ENSMUSG00000075702	2668	18,34
Impdh2	inosine 5'-phosphate dehydrogenase 2	ENSMUSG00000062867	4986	18,34
		ENSMUSG00000051627	748	18,34
Usp21	ubiquitin specific peptidase 21	ENSMUSG00000053483	6047	18,34
Med22	mediator complex subunit 22	ENSMUSG00000015776	5416	18,34
Ndufb8	NADH dehydrogenase (ubiquinone) 1 beta subcomplex 8	ENSMUSG00000025204	6869	18,34
Psmd6	proteasome (prosome, macropain) 26S subunit, non-ATPase, 6	ENSMUSG00000021737	8801	18,34
Wdr83	WD repeat domain containing 83	ENSMUSG00000005150	6272	18,34
Manbal	mannosidase, beta A, lysosomal-like	ENSMUSG00000063019	29170	18,34
Ccdc53	coiled-coil domain containing 53	ENSMUSG00000020056	45066	18,34
Map10	microtubule-associated protein 10	ENSMUSG00000050930	3548	18,34
Gemin8	gem (nuclear organelle) associated protein 8	ENSMUSG00000040621	20059	18,34
		ENSMUSG00000027331	39955	18,34
Nt5dc2	5'-nucleotidase domain containing 2	ENSMUSG00000071547	4269	18,34
Kars	lysyl-tRNA synthetase	ENSMUSG00000031948	17884	18,34
Ppap2a	phosphatidic acid phosphatase type 2A	ENSMUSG00000021759	66988	18,34
Gtpbp1	GTP binding protein 1	ENSMUSG00000042535	30584	18,34
Aldh6a1	aldehyde dehydrogenase family 6, subfamily A1	ENSMUSG00000021238	20228	18,34
Capn11	calpain 11	ENSMUSG00000058626	29122	18,34
E230008N13Rik	RIKEN cDNA E230008N13 gene	ENSMUSG00000035539	60472	18,34
Xdh	xanthine dehydrogenase	ENSMUSG00000024066	66275	18,27
Uck1	uridine-cytidine kinase 1	ENSMUSG00000002550	5158	18,27
		ENSMUSG00000072915	14799	18,27
Gramd1a	GRAM domain containing 1A	ENSMUSG00000001248	20924	18,27
		ENSMUSG00000024953	3410	18,27
Cluap1	clusterin associated protein 1	ENSMUSG00000014232	32347	18,27
Exosc1	exosome component 1	ENSMUSG00000034321	10335	18,27
Slc35g2	solute carrier family 35, member G2	ENSMUSG00000070287	18893	18,27
Trmt10a	tRNA methyltransferase 10A	ENSMUSG00000004127	16374	18,27
Dynlrb2	dynein light chain roadblock-type 2	ENSMUSG00000034467	10882	18,27
		ENSMUSG00000031147	8088	18,27
1700049G17Rik	RIKEN cDNA 1700049G17 gene	ENSMUSG00000070709	22069	18,27
		ENSMUSG00000062209	1075874	18,27
Mxra7	matrix-remodelling associated 7	ENSMUSG00000020814	24985	18,20
2700094K13Rik	RIKEN cDNA 2700094K13 gene	ENSMUSG00000076437	1553	18,20
Crlf3	cytokine receptor-like factor 3	ENSMUSG00000017561	34499	18,20
Stk31	serine threonine kinase 31	ENSMUSG00000023403	73898	18,20
		ENSMUSG00000038828	9613	18,20

Gng4	guanine nucleotide binding protein (G protein), gamma 4	ENSMUSG00000021303	43839	18,20
Trim25	tripartite motif-containing 25	ENSMUSG00000000275	20918	18,20
		ENSMUSG00000063297	429408	18,20
Nsun6	NOL1/NOP2/Sun domain family member 6	ENSMUSG00000026707	59754	18,20
Kif26a	kinesin family member 26A	ENSMUSG00000021294	35512	18,20
		ENSMUSG00000036264	423905	18,20
Bai3	brain-specific angiogenesis inhibitor 3	ENSMUSG00000033569	762232	18,20
Rmdn2	regulator of microtubule dynamics 2	ENSMUSG00000036368	67253	18,20
Rccd1	RCC1 domain containing 1	ENSMUSG00000038930	7859	18,20
Twf2	twinfilin, actin-binding protein, homolog 2 (Drosophila)	ENSMUSG00000023277	12280	18,12
Cdca8	cell division cycle associated 8	ENSMUSG00000028873	20847	18,12
Scpep1	serine carboxypeptidase 1	ENSMUSG00000000278	31446	18,12
Pdcd7	programmed cell death 7	ENSMUSG00000041837	13575	18,12
Gpc2	glypican 2 (cerebroglycan)	ENSMUSG00000029510	15054	18,12
Mettl17	methyltransferase like 17	ENSMUSG00000004561	8198	18,12
Efna1	ephrin A1	ENSMUSG00000027954	9410	18,12
Asnsd1	asparagine synthetase domain containing 1	ENSMUSG00000026095	27246	18,12
Pgap1	post-GPI attachment to proteins 1	ENSMUSG00000073678	84685	18,12
Mbd6	methyl-CpG binding domain protein 6	ENSMUSG00000025409	7063	18,12
Neto2	neuropilin (NRP) and tolloid (TLL)-like 2	ENSMUSG00000036902	54386	18,12
Trex1	three prime repair exonuclease 1	ENSMUSG00000049734	2406	18,12
Pank1	pantothenate kinase 1	ENSMUSG00000033610	68562	18,12
Rabep2	rabaptin, RAB GTPase binding effector protein 2	ENSMUSG00000030727	20487	18,12
Yif1a	Yip1 interacting factor homolog A (S. cerevisiae)	ENSMUSG00000024875	4342	18,12
Aqp11	aquaporin 11	ENSMUSG00000042797	11869	18,12
		ENSMUSG00000025195	64426	18,12
		ENSMUSG00000074194	9991	18,12
Syt5	synaptotagmin V	ENSMUSG00000004961	6800	18,05
Dbp	D site albumin promoter binding protein	ENSMUSG00000059824	5068	18,05
		ENSMUSG00000059920	19411	18,05
Psenen	presenilin enhancer 2 homolog (C. elegans)	ENSMUSG00000036835	1317	18,05
2210016F16Rik	RIKEN cDNA 2210016F16 gene	ENSMUSG00000021550	5180	18,05
Pdia4	protein disulfide isomerase associated 4	ENSMUSG00000025823	17371	18,05
Tesk2	testis-specific kinase 2	ENSMUSG00000033985	85009	18,05
Brcc3	BRCA1/BRCA2-containing complex, subunit 3	ENSMUSG00000031201	37374	18,05
		ENSMUSG00000043131	17761	18,05
Tex10	testis expressed gene 10	ENSMUSG00000028345	42602	18,05
Phf16	PHD finger protein 16	ENSMUSG00000037315	94252	18,05
Zfp472	zinc finger protein 472	ENSMUSG00000053600	13420	18,05
Lrrc23	leucine rich repeat containing 23	ENSMUSG00000030125	9865	18,05
Hsf1	heat shock factor 1	ENSMUSG00000022556	23528	18,05
		ENSMUSG00000078487	9065	18,05

Nnat	neuronatin	ENSMUSG00000067786	2445	17,98
Dusp3	dual specificity phosphatase 3 (vaccinia virus phosphatase VH1-related)	ENSMUSG00000003518	15871	17,98
Eid2b	EP300 interacting inhibitor of differentiation 2B	ENSMUSG00000070705	2424	17,98
Msantd3	Myb/SANT-like DNA-binding domain containing 3	ENSMUSG00000039693	21985	17,98
Ctss	cathepsin S	ENSMUSG00000038642	29618	17,98
Mea1	male enhanced antigen 1	ENSMUSG00000002768	1969	17,98
Dnajc2	DnaJ (Hsp40) homolog, subfamily C, member 2	ENSMUSG00000029014	27985	17,98
Tyw1	tRNA-yW synthesizing protein 1 homolog (S. cerevisiae)	ENSMUSG00000056310	85945	17,98
Selo	selenoprotein O	ENSMUSG00000035757	11257	17,98
Tfeb	transcription factor EB	ENSMUSG00000023990	55390	17,98
Pld2	phospholipase D2	ENSMUSG00000020828	18047	17,98
Pde9a	phosphodiesterase 9A	ENSMUSG00000041119	90077	17,98
Mpzl3	myelin protein zero-like 3	ENSMUSG00000070305	22251	17,98
Ifih1	interferon induced with helicase C domain 1	ENSMUSG00000026896	50458	17,98
Ptcd2	pentatricopeptide repeat domain 2	ENSMUSG00000021650	25030	17,91
Efhd2	EF hand domain containing 2	ENSMUSG00000040659	16779	17,91
Saal1	serum amyloid A-like 1	ENSMUSG00000006763	24573	17,91
Eme2	essential meiotic endonuclease 1 homolog 2 (S. pombe)	ENSMUSG00000073436	6602	17,91
Tspo	translocator protein	ENSMUSG00000041736	10629	17,91
Fam175a	family with sequence similarity 175, member A	ENSMUSG00000035234	15774	17,91
D10Wsu102e	DNA segment, Chr 10, Wayne State University 102, expressed	ENSMUSG00000020255	8615	17,91
Trnau1ap	tRNA selenocysteine 1 associated protein 1	ENSMUSG00000028898	17776	17,91
Slc46a3	solute carrier family 46, member 3	ENSMUSG00000029650	16379	17,91
2900055J20Rik	RIKEN cDNA 2900055J20 gene	ENSMUSG00000071860	723	17,91
		ENSMUSG00000079546	13517	17,91
		ENSMUSG00000038206	41304	17,91
		ENSMUSG00000053205	22113	17,91
		ENSMUSG00000062874	804	17,83
		ENSMUSG00000006732	4952	17,83
Mpp1	membrane protein, palmitoylated	ENSMUSG00000031402	21284	17,83
Smim12	small integral membrane protein 12	ENSMUSG00000042380	4026	17,83
		ENSMUSG00000047473	10705	17,83
Creld1	cysteine-rich with EGF-like domains 1	ENSMUSG00000030284	10042	17,83
Gnpnat1	glucosamine-phosphate N-acetyltransferase 1	ENSMUSG00000037722	12469	17,83
Pan2	PAN2 polyA specific ribonuclease subunit homolog (S. cerevisiae)	ENSMUSG00000005682	18024	17,83
Slc25a14	solute carrier family 25 (mitochondrial carrier, brain), member 14	ENSMUSG00000031105	38882	17,83
		ENSMUSG00000018841	14181	17,83
Il6st	interleukin 6 signal transducer	ENSMUSG00000021756	31853	17,83
Tada1	transcriptional adaptor 1	ENSMUSG00000026563	14525	17,83
Usp11	ubiquitin specific peptidase 11	ENSMUSG00000031066	16634	17,83

Aldh9a1	aldehyde dehydrogenase 9, subfamily A1	ENSMUSG00000026687	18540	17,83
Tor3a	torsin family 3, member A	ENSMUSG00000060519	20740	17,83
Pelp1	proline, glutamic acid and leucine rich protein 1	ENSMUSG00000018921	17149	17,76
		ENSMUSG00000031516	18377	17,76
Ss18l1	synovial sarcoma translocation gene on chromosome 18-like 1	ENSMUSG00000039086	27693	17,76
Ftsj1	FtsJ homolog 1 (E. coli)	ENSMUSG00000031171	13739	17,76
Snrpn	small nuclear ribonucleoprotein N	ENSMUSG00000000948	157725	17,76
Cpa6	carboxypeptidase A6	ENSMUSG00000042501	395226	17,76
Marveld1	MARVEL (membrane-associating) domain containing 1	ENSMUSG00000044345	4296	17,76
4930430A15Rik	RIKEN cDNA 4930430A15 gene	ENSMUSG00000027157	67542	17,76
Epha6	Eph receptor A6	ENSMUSG00000055540	952049	17,76
Mctp2	multiple C2 domains, transmembrane 2	ENSMUSG00000032776	228764	17,76
Synpr	synaptoporin	ENSMUSG00000056296	330680	17,76
		ENSMUSG00000097239	18105	17,69
Eif2b2	eukaryotic translation initiation factor 2B, subunit 2 beta	ENSMUSG00000004788	7148	17,69
Elac2	elaC homolog 2 (E. coli)	ENSMUSG00000020549	23032	17,69
Arl6ip4	ADP-ribosylation factor-like 6 interacting protein 4	ENSMUSG00000029404	2108	17,69
Fut1	fucosyltransferase 1	ENSMUSG00000008461	3454	17,69
Tsr1	TSR1 20S rRNA accumulation	ENSMUSG00000038335	11272	17,69
Mgat1	mannoside acetylglucosaminyltransferase 1	ENSMUSG00000020346	18840	17,69
Kif17	kinesin family member 17	ENSMUSG00000028758	51533	17,69
Armc5	armadillo repeat containing 5	ENSMUSG00000042178	7744	17,69
Camk1	calcium/calmodulin-dependent protein kinase I	ENSMUSG00000030272	9861	17,69
Fuk	fucokinase	ENSMUSG00000033703	19997	17,69
Zfp41	zinc finger protein 41	ENSMUSG00000047003	8622	17,69
Smo	smoothened homolog (Drosophila)	ENSMUSG00000001761	25672	17,69
Fam19a4	family with sequence similarity 19, member A4	ENSMUSG00000046500	229203	17,69
Nol9	nucleolar protein 9	ENSMUSG00000028948	22174	17,69
Scrn3	secernin 3	ENSMUSG00000008226	25218	17,69
Mocs1	molybdenum cofactor synthesis 1	ENSMUSG00000064120	27074	17,69
		ENSMUSG00000095315	39370	17,69
Als2	amyotrophic lateral sclerosis 2 (juvenile)	ENSMUSG00000026024	74306	17,69
Tmem170b	transmembrane protein 170B	ENSMUSG00000087370	35132	17,69
Plekhg2	pleckstrin homology domain containing, family G (with RhoGef domain) member 2	ENSMUSG00000037552	12996	17,69
Rcor2	REST corepressor 2	ENSMUSG00000024968	7901	17,62
Ube2g2	ubiquitin-conjugating enzyme E2G 2	ENSMUSG00000009293	23719	17,62
Mlst8	MTOR associated protein, LST8 homolog (S. cerevisiae)	ENSMUSG00000024142	5529	17,62
Sec14l5	SEC14-like 5 (S. cerevisiae)	ENSMUSG00000091712	36826	17,62
Prickle1	prickle homolog 1 (Drosophila)	ENSMUSG00000036158	96778	17,62

B4galt2	UDP-Gal:betaGlcNAc beta 1,4-galactosyltransferase, polypeptide 2	ENSMUSG00000028541	14228	17,62
		ENSMUSG00000027285	12178	17,62
Myd88	myeloid differentiation primary response gene 88	ENSMUSG00000032508	5478	17,62
		ENSMUSG00000020691	13970	17,62
Arsg	arylsulfatase G	ENSMUSG00000020604	99957	17,62
Ccdc111	coiled-coil domain containing 111	ENSMUSG00000038225	41634	17,62
Lgalsl	lectin, galactoside binding-like	ENSMUSG00000042363	7481	17,62
Ccbl1	cysteine conjugate-beta lyase 1	ENSMUSG00000039648	20724	17,62
Zfp952	zinc finger protein 952	ENSMUSG00000053390	12329	17,62
Cacna1c	calcium channel, voltage-dependent, L type, alpha 1C subunit	ENSMUSG00000051331	188006	17,62
Plekha1	pleckstrin homology domain containing, family A (phosphoinositide binding specific) member 1	ENSMUSG00000040268	47557	17,62
Lrrc36	leucine rich repeat containing 36	ENSMUSG00000054320	50516	17,62
Dck	deoxycytidine kinase	ENSMUSG00000029366	18286	17,62
Kcns3	potassium voltage-gated channel, delayed-rectifier, subfamily S, member 3	ENSMUSG00000043673	60641	17,54
		ENSMUSG00000091695	465	17,54
Haghl	hydroxyacylglutathione hydrolase-like	ENSMUSG00000061046	5831	17,54
Plekho1	pleckstrin homology domain containing, family O member 1	ENSMUSG00000015745	7573	17,54
Rbak	RB-associated KRAB repressor	ENSMUSG00000061898	8590	17,54
Arfip2	ADP-ribosylation factor interacting protein 2	ENSMUSG00000030881	6214	17,54
		ENSMUSG00000003929	29537	17,54
Cd180	CD180 antigen	ENSMUSG00000021624	46072	17,54
Wdr90	WD repeat domain 90	ENSMUSG00000073434	16731	17,54
1700030K09Rik	RIKEN cDNA 1700030K09 gene	ENSMUSG00000052794	17122	17,54
Ppp1r9b	protein phosphatase 1, regulatory subunit 9B	ENSMUSG00000038976	15865	17,47
Polr2e	polymerase (RNA) II (DNA directed) polypeptide E	ENSMUSG00000004667	3847	17,47
Zfp954	zinc finger protein 954	ENSMUSG00000062116	6756	17,47
Pde5a	phosphodiesterase 5A, cGMP-specific	ENSMUSG00000053965	130217	17,47
Ssr2	signal sequence receptor, beta	ENSMUSG00000041355	8727	17,47
		ENSMUSG00000007833	13901	17,47
Tbc1d10a	TBC1 domain family, member 10a	ENSMUSG00000034412	28717	17,47
Lcmt2	leucine carboxyl methyltransferase 2	ENSMUSG00000074890	12392	17,47
Chdh	choline dehydrogenase	ENSMUSG00000015970	31444	17,47
Rhou	ras homolog gene family, member U	ENSMUSG00000039960	9956	17,47
Trip6	thyroid hormone receptor interactor 6	ENSMUSG00000023348	4343	17,47
Pabpc4	poly(A) binding protein, cytoplasmic 4	ENSMUSG00000011257	36575	17,47
		ENSMUSG00000069633	10576	17,47

Zfp474	zinc finger protein 474	ENSMUSG00000046886	23916	17,47
Kcnj16	potassium inwardly-rectifying channel, subfamily J, member 16	ENSMUSG00000051497	59936	17,47
Tsr2	TSR2 20S rRNA accumulation	ENSMUSG00000025264	9450	17,40
Slc16a6	solute carrier family 16 (monocarboxylic acid transporters), member 6	ENSMUSG00000041920	22744	17,40
2810428I15Rik	RIKEN cDNA 2810428I15 gene	ENSMUSG00000058833	2461	17,40
Rmnd5b	required for meiotic nuclear division 5 homolog B (S. cerevisiae)	ENSMUSG00000001054	12226	17,40
Palb2	partner and localizer of BRCA2	ENSMUSG00000044702	25685	17,40
Ppa2	pyrophosphatase (inorganic) 2	ENSMUSG00000028013	68126	17,40
D230025D16Rik	RIKEN cDNA D230025D16 gene	ENSMUSG00000031889	27909	17,40
Ethe1	ethylmalonic encephalopathy 1	ENSMUSG00000064254	21383	17,40
Kndc1	kinase non-catalytic C-lobe domain (KIND) containing 1	ENSMUSG00000066129	46842	17,40
Fbxl16	F-box and leucine-rich repeat protein 16	ENSMUSG00000025738	12160	17,33
Mrpl10	mitochondrial ribosomal protein L10	ENSMUSG00000001445	7654	17,33
Lbh	limb-bud and heart	ENSMUSG00000024063	23638	17,33
Draxin	dorsal inhibitory axon guidance protein	ENSMUSG00000029005	32262	17,33
Dnah17	dynein, axonemal, heavy chain 17	ENSMUSG00000033987	107497	17,33
Fbxo30	F-box protein 30	ENSMUSG00000047648	16723	17,33
Btbd2	BTB (POZ) domain containing 2	ENSMUSG00000003344	14455	17,33
Arap1	ArfGAP with RhoGAP domain, ankyrin repeat and PH domain 1	ENSMUSG00000032812	64520	17,33
Mtap	methylthioadenosine phosphorylase	ENSMUSG00000062937	43960	17,33
Zfp945	zinc finger protein 945	ENSMUSG00000059142	20438	17,33
Brf2	BRF2, subunit of RNA polymerase III transcription initiation factor, BRF1-like	ENSMUSG00000031487	4801	17,33
Adcy10	adenylate cyclase 10	ENSMUSG00000026567	91592	17,33
		ENSMUSG00000031789	45137	17,25
Fam43a	family with sequence similarity 43, member A	ENSMUSG00000046546	3075	17,25
Parp16	poly (ADP-ribose) polymerase family, member 16	ENSMUSG00000032392	24530	17,25
Saysd1	SAYSVFN motif domain containing 1	ENSMUSG00000045107	7527	17,25
Ndufv1	NADH dehydrogenase (ubiquinone) flavoprotein 1	ENSMUSG00000037916	5310	17,25
Yipf2	Yip1 domain family, member 2	ENSMUSG00000032182	4123	17,25
Rnaseh2a	ribonuclease H2, large subunit	ENSMUSG00000052926	13158	17,25
Sav1	salvador homolog 1 (Drosophila)	ENSMUSG00000021067	21991	17,25
Tmem9b	TMEM9 domain family, member B	ENSMUSG00000031021	17042	17,25
Tagap	T cell activation Rho GTPase activating protein	ENSMUSG00000033450	8898	17,25
Pdcl3	phosducin-like 3	ENSMUSG00000026078	9423	17,25
Ccdc51	coiled-coil domain containing 51	ENSMUSG00000025645	10868	17,25
Ggcx	gamma-glutamyl carboxylase	ENSMUSG00000053460	16405	17,25
Snip1	Smad nuclear interacting protein 1	ENSMUSG00000050213	7371	17,18
Dtx3	deltex 3 homolog (Drosophila)	ENSMUSG00000040415	5351	17,18
Amer2	APC membrane recruitment 2	ENSMUSG00000021986	2718	17,18

Leng1	leukocyte receptor cluster (LRC) member 1	ENSMUSG00000078813	5743	17,18
Pcyox1l	prenylcysteine oxidase 1 like	ENSMUSG00000024579	10799	17,18
Map3k19	mitogen-activated protein kinase kinase kinase 19	ENSMUSG00000051590	25020	17,18
Wdr18	WD repeat domain 18	ENSMUSG00000035754	9095	17,18
Slx1b	SLX1 structure-specific endonuclease subunit homolog B (S. cerevisiae)	ENSMUSG00000059772	6317	17,18
Dgcr6	DiGeorge syndrome critical region gene 6	ENSMUSG00000003531	18773	17,18
Tada3	transcriptional adaptor 3	ENSMUSG00000048930	11859	17,18
		ENSMUSG00000055681	10476	17,18
Ccdc58	coiled-coil domain containing 58	ENSMUSG00000075229	20459	17,18
Hps4	Hermansky-Pudlak syndrome 4 homolog (human)	ENSMUSG00000042328	35332	17,18
Trmu	tRNA 5-methylaminomethyl-2-thiouridylate methyltransferase	ENSMUSG00000022386	18083	17,18
Pex3	peroxisomal biogenesis factor 3	ENSMUSG00000019809	29301	17,18
Ebag9	estrogen receptor-binding fragment-associated gene 9	ENSMUSG00000022339	21384	17,18
Tpp1	tripeptidyl peptidase I	ENSMUSG00000030894	7361	17,18
Tmed8	transmembrane emp24 domain containing 8	ENSMUSG00000034111	33988	17,18
Pdk3	pyruvate dehydrogenase kinase, isoenzyme 3	ENSMUSG00000035232	67595	17,18
Dcp1a	DCP1 decapping enzyme homolog A (S. cerevisiae)	ENSMUSG00000021962	47492	17,18
Lime1	Lck interacting transmembrane adaptor 1	ENSMUSG00000090077	3551	17,18
Hps5	Hermansky-Pudlak syndrome 5 homolog (human)	ENSMUSG00000014418	35599	17,18
Skp2	S-phase kinase-associated protein 2 (p45)	ENSMUSG00000054115	28467	17,18
Tll1	tolloid-like	ENSMUSG00000053626	191224	17,18
Brca2	breast cancer 2	ENSMUSG00000041147	47117	17,18
Gpr160	G protein-coupled receptor 160	ENSMUSG00000037661	41243	17,18
Pot1b	protection of telomeres 1B	ENSMUSG00000024174	60604	17,18
Mtbp	Mdm2, transformed 3T3 cell double minute p53 binding protein	ENSMUSG00000022369	69016	17,18
		ENSMUSG00000093954	81477	17,18
		ENSMUSG00000007872	2259	17,11
Ckap4	cytoskeleton-associated protein 4	ENSMUSG00000046841	7732	17,11
		ENSMUSG00000027833	10045	17,11
Mrpl2	mitochondrial ribosomal protein L2	ENSMUSG00000002767	3911	17,11
Mrps2	mitochondrial ribosomal protein S2	ENSMUSG00000035772	3113	17,11
Klhdc3	kelch domain containing 3	ENSMUSG00000063576	6381	17,11
Alkbh6	alkB, alkylation repair homolog 6 (E. coli)	ENSMUSG00000042831	5588	17,11
Grwd1	glutamate-rich WD repeat containing 1	ENSMUSG00000053801	5718	17,11
		ENSMUSG00000022668	9596	17,11
Zeb2	zinc finger E-box binding homeobox 2	ENSMUSG00000026872	133762	17,11
Ogfrl1	opioid growth factor receptor-like 1	ENSMUSG00000026158	16752	17,11
Ipp	IAP promoted placental gene	ENSMUSG00000028696	30695	17,11

Zkscan6	zinc finger with KRAB and SCAN domains 6	ENSMUSG00000018347	22065	17,11
Alkbh3	alkB, alkylation repair homolog 3 (E. coli)	ENSMUSG00000040174	30258	17,11
Dgke	diacylglycerol kinase, epsilon	ENSMUSG00000000276	31672	17,11
Gm14308	predicted gene 14308	ENSMUSG00000078880	22981	17,11
Ctse	cathepsin E	ENSMUSG00000004552	37198	17,11
Dcun1d4	DCN1, defective in cullin neddylation 1, domain containing 4 (S. cerevisiae)	ENSMUSG00000051674	79793	17,11
Wdr65	WD repeat domain 65	ENSMUSG00000028730	66227	17,11
Clip2	CAP-GLY domain containing linker protein 2	ENSMUSG00000063146	63049	17,11
Chrna7	cholinergic receptor, nicotinic, alpha polypeptide 7	ENSMUSG00000030525	113835	17,11
Dus3l	dihydrouridine synthase 3-like (S. cerevisiae)	ENSMUSG00000007603	5342	17,04
R3hcc1	R3H domain and coiled-coil containing 1	ENSMUSG00000034194	10278	17,04
Arrdc4	arrestin domain containing 4	ENSMUSG00000042659	12197	17,04
Zfp747	zinc finger protein 747	ENSMUSG00000054381	3487	17,04
Fanci	Fanconi anemia, complementation group I	ENSMUSG00000039187	58337	17,04
Top3b	topoisomerase (DNA) III beta	ENSMUSG00000022779	22255	17,04
Pbld2	phenazine biosynthesis-like protein domain containing 2	ENSMUSG00000020068	34499	17,04
Slc2a9	solute carrier family 2 (facilitated glucose transporter), member 9	ENSMUSG00000005107	153871	17,04
Lrrc41	leucine rich repeat containing 41	ENSMUSG00000028703	21775	17,04
Gcfc2	GC-rich sequence DNA binding factor 2	ENSMUSG00000035125	36247	17,04
Rab31	RAB31, member RAS oncogene family	ENSMUSG00000056515	121027	17,04
Tmem222	transmembrane protein 222	ENSMUSG00000028857	11748	16,96
Numbl	numb-like	ENSMUSG00000063160	23712	16,96
Gosr1	golgi SNAP receptor complex member 1	ENSMUSG00000010392	36978	16,96
		ENSMUSG00000031821	10672	16,96
		ENSMUSG00000022545	40934	16,96
2310030G06Rik	RIKEN cDNA 2310030G06 gene	ENSMUSG00000032062	6831	16,96
		ENSMUSG00000022864	32646	16,96
Mrpl23	mitochondrial ribosomal protein L23	ENSMUSG00000037772	7949	16,89
Slc50a1	solute carrier family 50 (sugar transporter), member 1	ENSMUSG00000027953	2325	16,89
Ckm	creatine kinase, muscle	ENSMUSG00000030399	10490	16,89
Ubl4	ubiquitin-like 4	ENSMUSG00000015290	7501	16,89
Dpf2	D4, zinc and double PHD fingers family 2	ENSMUSG00000024826	16495	16,89
Wdr46	WD repeat domain 46	ENSMUSG00000024312	9036	16,89
Phf10	PHD finger protein 10	ENSMUSG00000023883	16265	16,89
Nfkbil1	nuclear factor of kappa light polypeptide gene enhancer in B cells inhibitor like 1	ENSMUSG00000042419	15618	16,89
Fxn	frataxin	ENSMUSG00000059363	19153	16,89

Ermard	ER membrane associated RNA degradation	ENSMUSG00000036552	22592	16,89
Ephb3	Eph receptor B3	ENSMUSG00000005958	18551	16,89
Amdhd2	amidohydrolase domain containing 2	ENSMUSG00000036820	7934	16,89
Trim11	tripartite motif-containing 11	ENSMUSG00000020455	13366	16,89
Spryd7	SPRY domain containing 7	ENSMUSG00000021930	24894	16,89
Cnksr2	connector enhancer of kinase suppressor of Ras 2	ENSMUSG00000025658	221859	16,89
Lad1	ladinin	ENSMUSG00000041782	14744	16,89
		ENSMUSG00000073514	468447	16,89
Scly	selenocysteine lyase	ENSMUSG00000026307	22738	16,89
Pole	polymerase (DNA directed), epsilon	ENSMUSG00000007080	51169	16,89
Sptb	spectrin beta, erythrocytic	ENSMUSG00000021061	130060	16,89
Parp14	poly (ADP-ribose) polymerase family, member 14	ENSMUSG00000034422	38671	16,89
Prdm11	PR domain containing 11	ENSMUSG00000075028	81017	16,89
Exosc3	exosome component 3	ENSMUSG00000028322	26120	16,82
Dbndd2	dysbindin (dystrobrevin binding protein 1) domain containing 2	ENSMUSG00000017734	7214	16,82
		ENSMUSG00000072772	2192	16,82
Tfip11	tuftelin interacting protein 11	ENSMUSG00000029345	11716	16,82
Pard6b	par-6 (partitioning defective 6) homolog beta (C. elegans)	ENSMUSG00000044641	20200	16,82
Nop16	NOP16 nucleolar protein	ENSMUSG00000025869	5906	16,82
Tpst2	protein-tyrosine sulfotransferase 2	ENSMUSG00000029344	38671	16,82
Sass6	spindle assembly 6 homolog (C. elegans)	ENSMUSG00000027959	35977	16,82
Rmi1	RMI1, RecQ mediated genome instability 1, homolog (S. cerevisiae)	ENSMUSG00000035367	8553	16,82
Zfp867	zinc finger protein 867	ENSMUSG00000054519	11278	16,82
Sdr42e1	short chain dehydrogenase/reductase family 42E, member 1	ENSMUSG00000034308	12291	16,82
Hexdc	hexosaminidase (glycosyl hydrolase family 20, catalytic domain) containing	ENSMUSG00000039307	18223	16,82
Mbd4	methyl-CpG binding domain protein 4	ENSMUSG00000030322	12675	16,82
		ENSMUSG00000078870	22969	16,82
Pitrm1	pitrilysin metallepetidase 1	ENSMUSG00000021193	31983	16,82
Satb2	special AT-rich sequence binding protein 2	ENSMUSG00000038331	184665	16,82
Pcgf5	polycomb group ring finger 5	ENSMUSG00000024805	77126	16,82
Cr1l	complement component (3b/4b) receptor 1-like	ENSMUSG00000016481	27782	16,82
Prosc	proline synthetase co-transcribed	ENSMUSG00000031485	13577	16,82
Plxnc1	plexin C1	ENSMUSG00000074785	153713	16,82
		ENSMUSG00000058818	8983	16,82
Gm3336	predicted gene 3336	ENSMUSG00000095026	4093	16,82
Dbi	diazepam binding inhibitor	ENSMUSG00000026385	7799	16,75
Fam89a	family with sequence similarity 89, member A	ENSMUSG00000043068	11553	16,75
Ncs1	neuronal calcium sensor 1	ENSMUSG00000062661	50167	16,75

Smpd4	sphingomyelin phosphodiesterase 4	ENSMUSG00000005899	25475	16,75
Car14	carbonic anhydrase 14	ENSMUSG00000038526	6924	16,75
Gpr180	G protein-coupled receptor 180	ENSMUSG00000022131	27106	16,75
		ENSMUSG00000046185	11270	16,75
Rabif	RAB interacting factor	ENSMUSG00000042229	13223	16,75
Arhgap42	Rho GTPase activating protein 42	ENSMUSG00000050730	244773	16,75
Cep192	centrosomal protein 192	ENSMUSG00000024542	85062	16,75
Ttc39a	tetratricopeptide repeat domain 39A	ENSMUSG00000028555	38123	16,75
Cox5b	cytochrome c oxidase subunit Vb	ENSMUSG00000061518	1899	16,67
Glul	glutamate-ammonia ligase (glutamine synthetase)	ENSMUSG00000026473	9780	16,67
Dusp7	dual specificity phosphatase 7	ENSMUSG00000053716	7093	16,67
Mgat2	mannoside acetylglucosaminyltransferase 2	ENSMUSG00000043998	2615	16,67
Zdhhc8	zinc finger, DHHC domain containing 8	ENSMUSG00000060166	14383	16,67
Dnpep	aspartyl aminopeptidase	ENSMUSG00000026209	9742	16,67
Slc35f6	solute carrier family 35, member F6	ENSMUSG00000029175	11799	16,67
Mrps23	mitochondrial ribosomal protein S23	ENSMUSG00000023723	7120	16,67
Tmem199	transmembrane protein 199	ENSMUSG00000051232	5118	16,67
Klkb1	kallikrein B, plasma 1	ENSMUSG00000031640	25418	16,67
Alg5	asparagine-linked glycosylation 5 (dolichyl-phosphate beta-glucosyltransferase)	ENSMUSG00000036632	15589	16,67
Kir3dl2	killer cell immunoglobulin-like receptor, three domains, long cytoplasmic tail, 2	ENSMUSG00000057439	95950	16,67
Slc41a2	solute carrier family 41, member 2	ENSMUSG00000034591	107035	16,67
Dmrta1	doublesex and mab-3 related transcription factor like family A1	ENSMUSG00000043753	15337	16,67
Sema3e	sema domain, immunoglobulin domain (Ig), short basic domain, secreted, (semaphorin) 3E	ENSMUSG00000063531	231414	16,67
Grm7	glutamate receptor, metabotropic 7	ENSMUSG00000056755	921650	16,67
Col25a1	collagen, type XXV, alpha 1	ENSMUSG00000058897	418987	16,67
Tff2	trefoil factor 2 (spasmolytic protein 1)	ENSMUSG00000024028	3234	16,67
Mrto4	MRT4, mRNA turnover 4, homolog (S. cerevisiae)	ENSMUSG00000028741	5142	16,60
Gm26596	predicted gene, 26596	ENSMUSG00000097185	2655	16,60
Cnot11	CCR4-NOT transcription complex, subunit 11	ENSMUSG00000003135	11890	16,60
Poglut1	protein O-glucosyltransferase 1	ENSMUSG00000034064	25122	16,60
Bloc1s6	biogenesis of organelles complex-1, subunit 6, pallidin	ENSMUSG00000005804	10973	16,60
Scnm1	sodium channel modifier 1	ENSMUSG00000092607	4477	16,60
1700016K19Rik	RIKEN cDNA 1700016K19 gene	ENSMUSG00000053783	3658	16,60
BC023829	cDNA sequence BC023829	ENSMUSG00000073139	17428	16,60
Flad1	RFad1, flavin adenine dinucleotide synthetase, homolog (yeast)	ENSMUSG00000042642	10867	16,60
Prps2	phosphoribosyl pyrophosphate synthetase 2	ENSMUSG00000025742	36428	16,60

Gosr2	golgi SNAP receptor complex member 2	ENSMUSG00000020946	21050	16,60
Anxa4	annexin A4	ENSMUSG00000029994	56745	16,60
Zbtb43	zinc finger and BTB domain containing 43	ENSMUSG00000026788	18273	16,60
Zfp160	zinc finger protein 160	ENSMUSG00000067942	19912	16,60
Ears2	glutamyl-tRNA synthetase 2 (mitochondrial)(putative)	ENSMUSG00000030871	30051	16,60
Chn2	chimerin (chimaerin) 2	ENSMUSG00000004633	262257	16,60
Fam227b	family with sequence similarity 227, member B	ENSMUSG00000027209	168522	16,60
Rhbdf2	rhomboid 5 homolog 2 (Drosophila)	ENSMUSG00000020806	28855	16,60
Fam46b	family with sequence similarity 46, member B	ENSMUSG00000046694	7807	16,60
Spc25	SPC25, NDC80 kinetochore complex component, homolog (S. cerevisiae)	ENSMUSG00000005233	12300	16,53
Lsm11	U7 snRNP-specific Sm-like protein LSM11	ENSMUSG00000044847	16667	16,53
Npepl1	aminopeptidase-like 1	ENSMUSG00000039263	12722	16,53
Prpf3	PRP3 pre-mRNA processing factor 3 homolog (yeast)	ENSMUSG00000015748	25762	16,53
Rab2b	RAB2B, member RAS oncogene family	ENSMUSG00000022159	17787	16,53
Oplah	5-oxoprolinase (ATP-hydrolysing)	ENSMUSG00000022562	31419	16,53
Stk30	serine/threonine kinase 30	ENSMUSG00000056458	33142	16,53
Smad7	SMAD family member 7	ENSMUSG00000025880	28407	16,53
Mrps36-ps1	mitichondrial ribosomal protein S36, pseudogene 1	ENSMUSG00000021631	625	16,46
		ENSMUSG00000092252	14425	16,46
4933413G19Rik	RIKEN cDNA 4933413G19 gene	ENSMUSG00000079304	9689	16,46
Apc2	adenomatosis polyposis coli 2	ENSMUSG00000020135	22287	16,46
Ccar2	cell cycle activator and apoptosis regulator 2	ENSMUSG00000033712	15635	16,46
Mapk1ip1	mitogen-activated protein kinase 1 interacting protein 1	ENSMUSG00000041775	10451	16,46
Ilk	integrin linked kinase	ENSMUSG00000030890	6334	16,46
Fbxw5	F-box and WD-40 domain protein 5	ENSMUSG00000015095	4722	16,46
Nacc2	nucleus accumbens associated 2, BEN and BTB (POZ) domain containing	ENSMUSG00000026932	67686	16,46
		ENSMUSG00000000532	39575	16,46
Stk10	serine/threonine kinase 10	ENSMUSG00000020272	91283	16,46
Shisa6	shisa homolog 6 (Xenopus laevis)	ENSMUSG00000053930	314240	16,46
Lgi2	leucine-rich repeat LGI family, member 2	ENSMUSG00000039252	28440	16,46
Olfm2	olfactomedin 2	ENSMUSG00000032172	60234	16,46
Polr1e	polymerase (RNA) I polypeptide E	ENSMUSG00000028318	17983	16,38
Rnf25	ring finger protein 25	ENSMUSG00000026171	7650	16,38
Adc	arginine decarboxylase	ENSMUSG00000028789	32210	16,38
		ENSMUSG00000038900	2938	16,38
2310022A10Rik	RIKEN cDNA 2310022A10 gene	ENSMUSG00000049643	28800	16,38
Asb1	ankyrin repeat and SOCS box-containing 1	ENSMUSG00000026311	19026	16,38
Slc35a5	solute carrier family 35, member A5	ENSMUSG00000022664	19134	16,38

Usb1	U6 snRNA biogenesis 1	ENSMUSG00000031792	15216	16,38
		ENSMUSG00000074661	1855	16,38
		ENSMUSG00000037640	22314	16,38
		ENSMUSG00000055396	1142	16,38
Inha	inhibin alpha	ENSMUSG00000032968	3290	16,38
Mal2	mal, T cell differentiation protein 2	ENSMUSG00000024479	31481	16,38
Tmem158	transmembrane protein 158	ENSMUSG00000054871	1732	16,38
Gch1	GTP cyclohydrolase 1	ENSMUSG00000037580	35519	16,38
Gnb5	guanine nucleotide binding protein (G protein), beta 5	ENSMUSG00000032192	33570	16,38
Creb3l4	cAMP responsive element binding protein 3-like 4	ENSMUSG00000027938	6013	16,31
St6galnac6	ST6 (alpha-N-acetyl-neuraminyl-2,3-beta-galactosyl-1,3)-N-acetylgalactosaminide alpha-2,6-sialyltransferase 6	ENSMUSG00000026811	21098	16,31
Tmem106c	transmembrane protein 106C	ENSMUSG00000052369	6058	16,31
Bub3	budding uninhibited by benzimidazoles 3 homolog (S. cerevisiae)	ENSMUSG00000066979	11508	16,31
Lemd1	LEM domain containing 1	ENSMUSG00000079330	65946	16,31
Mtif3	mitochondrial translational initiation factor 3	ENSMUSG00000016510	12228	16,31
Otub2	OTU domain, ubiquitin aldehyde binding 2	ENSMUSG00000021203	17669	16,31
Cspg5	chondroitin sulfate proteoglycan 5	ENSMUSG00000032482	18793	16,31
Epn1	epsin 1	ENSMUSG00000035203	17944	16,31
Mtx3	metaxin 3	ENSMUSG00000021704	13444	16,31
		ENSMUSG00000020553	20230	16,31
Klf8	Kruppel-like factor 8	ENSMUSG00000041649	158667	16,31
Ptar1	protein prenyltransferase alpha subunit repeat containing 1	ENSMUSG00000074925	33730	16,31
Crtc1	CREB regulated transcription coactivator 1	ENSMUSG00000003575	57225	16,31
Hhat	hedgehog acyltransferase	ENSMUSG00000037375	258386	16,31
Zfat	zinc finger and AT hook domain containing	ENSMUSG00000022335	175091	16,31
Ebp	phenylalkylamine Ca2+ antagonist (emopamil) binding protein	ENSMUSG00000031168	8184	16,24
Dctn3	dynactin 3	ENSMUSG00000028447	8373	16,24
Nme3	NME/NM23 nucleoside diphosphate kinase 3	ENSMUSG00000073435	1023	16,24
Anks6	ankyrin repeat and sterile alpha motif domain containing 6	ENSMUSG00000066191	41638	16,24
Exosc2	exosome component 2	ENSMUSG00000039356	10636	16,24
		ENSMUSG00000091460	387	16,24
Tmem128	transmembrane protein 128	ENSMUSG00000067365	9438	16,24
Patl1	protein associated with topoisomerase II homolog 1 (yeast)	ENSMUSG00000046139	32698	16,24
Mrpl52	mitochondrial ribosomal protein L52	ENSMUSG00000010406	2842	16,24
Wdr82	WD repeat domain containing 82	ENSMUSG00000020257	20778	16,24
Mum1l1	melanoma associated antigen (mutated) 1-like 1	ENSMUSG00000042515	28294	16,24
Col18a1	collagen, type XVIII, alpha 1	ENSMUSG00000001435	114371	16,24

Tcaim	T cell activation inhibitor, mitochondrial	ENSMUSG00000046603	30796	16,24
Zfp560	zinc finger protein 560	ENSMUSG00000045519	40042	16,24
Tln2	talin 2	ENSMUSG00000052698	342617	16,24
Mdfic	MyoD family inhibitor domain containing	ENSMUSG00000041390	81505	16,24
Ccdc153	coiled-coil domain containing 153	ENSMUSG00000070306	6630	16,24
Arc	activity regulated cytoskeletal-associated protein	ENSMUSG00000022602	3488	16,24
E4f1	E4F transcription factor 1	ENSMUSG00000024137	11520	16,17
1700029I15Rik	RIKEN cDNA 1700029I15 gene	ENSMUSG00000044916	2791	16,17
Gabpa	GA repeat binding protein, alpha	ENSMUSG00000008976	28855	16,17
Msantd4	Myb/SANT-like DNA-binding domain containing 4 with coiled-coils	ENSMUSG00000041124	3327	16,17
Arhgef40	Rho guanine nucleotide exchange factor (GEF) 40	ENSMUSG00000004562	21533	16,17
Tmem184c	transmembrane protein 184C	ENSMUSG00000031617	14717	16,17
Ndufs6	NADH dehydrogenase (ubiquinone) Fe-S protein 6	ENSMUSG00000021606	8705	16,17
		ENSMUSG00000007570	13039	16,17
Zer1	zyg-11 related, cell cycle regulator	ENSMUSG00000039686	27303	16,17
Ldlrad4	low density lipoprotein receptor class A domain containing 4	ENSMUSG00000024544	322293	16,17
Tmem29	transmembrane protein 29	ENSMUSG00000041353	61382	16,17
Lrrc26	leucine rich repeat containing 26	ENSMUSG00000026961	1266	16,17
Tmem88	transmembrane protein 88	ENSMUSG00000045377	1716	16,17
Klf11	Kruppel-like factor 11	ENSMUSG00000020653	11404	16,17
Alk	anaplastic lymphoma kinase	ENSMUSG00000055471	734268	16,17
Apoc1	apolipoprotein C-I	ENSMUSG00000040564	3175	16,09
2310009B15Rik	RIKEN cDNA 2310009B15 gene	ENSMUSG00000079283	4876	16,09
Rps4y2	ribosomal protein S4, Y-linked 2	ENSMUSG00000063171	943	16,09
Utp23	UTP23, small subunit (SSU) processome component, homolog (yeast)	ENSMUSG00000022313	7186	16,09
Abhd11	abhydrolase domain containing 11	ENSMUSG00000040532	3024	16,09
Aarsd1	alanyl-tRNA synthetase domain containing 1	ENSMUSG00000075528	10777	16,09
8430419L09Rik	RIKEN cDNA 8430419L09 gene	ENSMUSG00000030207	46979	16,09
		ENSMUSG00000047721	1658	16,09
Taf5	TAF5 RNA polymerase II, TATA box binding protein (TBP)-associated factor	ENSMUSG00000025049	15732	16,09
0610040J01Rik	RIKEN cDNA 0610040J01 gene	ENSMUSG00000060512	87125	16,09
Pinx1	PIN2/TERF1 interacting, telomerase inhibitor 1	ENSMUSG00000021958	59548	16,09
Asun	asunder, spermatogenesis regulator	ENSMUSG00000040250	28204	16,09
Zfp143	zinc finger protein 143	ENSMUSG00000061079	33691	16,09
Map3k1	mitogen-activated protein kinase kinase kinase 1	ENSMUSG00000021754	62566	16,09
Tmem151a	transmembrane protein 151A	ENSMUSG00000061451	14673	16,02
Mblac1	metallo-beta-lactamase domain containing 1	ENSMUSG00000049285	1308	16,02
		ENSMUSG00000005732	8949	16,02

Ndufaf1	NADH dehydrogenase (ubiquinone) 1 alpha subcomplex, assembly factor 1	ENSMUSG00000027305	7382	16,02
Rnf166	ring finger protein 166	ENSMUSG00000014470	9918	16,02
Hgsnat	heparan-alpha-glucosaminide N-acetyltransferase	ENSMUSG00000037260	32286	16,02
Ccdc142	coiled-coil domain containing 142	ENSMUSG00000079511	8323	16,02
		ENSMUSG00000061461	16405	16,02
Ict1	immature colon carcinoma transcript 1	ENSMUSG00000018858	8923	16,02
Gpaa1	GPI anchor attachment protein 1	ENSMUSG00000022561	3677	16,02
Dph3	diphthamine biosynthesis 3	ENSMUSG00000021905	5127	16,02
Bbs5	Bardet-Biedl syndrome 5 (human)	ENSMUSG00000063145	20401	16,02
Them4	thioesterase superfamily member 4	ENSMUSG00000028145	22452	16,02
Pggt1b	protein geranylgeranyltransferase type I, beta subunit	ENSMUSG00000024477	40902	16,02
Bpifb4	BPI fold containing family B, member 4	ENSMUSG00000074665	25890	15,95
Cxcl12	chemokine (C-X-C motif) ligand 12	ENSMUSG00000061353	12833	15,95
1700123O20Rik	RIKEN cDNA 1700123O20 gene	ENSMUSG00000040822	4572	15,95
Sdc1	syndecan 1	ENSMUSG00000020592	22393	15,95
		ENSMUSG00000095308	801	15,95
2310067B10Rik	RIKEN cDNA 2310067B10 gene	ENSMUSG00000020747	33934	15,95
Tmem130	transmembrane protein 130	ENSMUSG00000043388	25735	15,95
Cadm3	cell adhesion molecule 3	ENSMUSG00000005338	33442	15,95
		ENSMUSG00000036721	6998	15,95
Pfdn4	prefoldin 4	ENSMUSG00000052033	22696	15,95
Armc6	armadillo repeat containing 6	ENSMUSG00000002343	14295	15,95
Ccdc37	coiled-coil domain containing 37	ENSMUSG00000048794	25062	15,95
		ENSMUSG00000020235	12295	15,95
Fam173b	family with sequence similarity 173, member B	ENSMUSG00000039065	19376	15,95
Map3k9	mitogen-activated protein kinase kinase kinase 9	ENSMUSG00000042724	66221	15,95
Ptplad2	protein tyrosine phosphatase-like A domain containing 2	ENSMUSG00000028497	42785	15,95
Pkd2l2	polycystic kidney disease 2-like 2	ENSMUSG00000014503	33367	15,95
Pola2	polymerase (DNA directed), alpha 2	ENSMUSG00000024833	23664	15,95
Pgs1	phosphatidylglycerophosphate synthase 1	ENSMUSG00000017715	37720	15,95
Ift57	intraflagellar transport 57	ENSMUSG00000032965	65894	15,95
Ccl28	chemokine (C-C motif) ligand 28	ENSMUSG00000074715	30536	15,95
Stox1	storkhead box 1	ENSMUSG00000036923	67086	15,95
Zfp738	zinc finger protein 738	ENSMUSG00000048280	19635	15,95
Cxcl16	chemokine (C-X-C motif) ligand 16	ENSMUSG00000018920	6002	15,95
		ENSMUSG00000029338	147951	15,95
Grin1	glutamate receptor, ionotropic, NMDA1 (zeta 1)	ENSMUSG00000026959	28007	15,95
Zfp358	zinc finger protein 358	ENSMUSG00000047264	4071	15,88
Gmnn	geminin	ENSMUSG00000006715	10079	15,88
Rfc3	replication factor C (activator 1) 3	ENSMUSG00000033970	8487	15,88
Timeless	timeless circadian clock 1	ENSMUSG00000039994	20877	15,88
Dmwd	dystrophia myotonica-containing WD repeat motif	ENSMUSG00000030410	6528	15,88
Otud3	OTU domain containing 3	ENSMUSG00000041161	18567	15,88

Pxmp2	peroxisomal membrane protein 2	ENSMUSG00000029499	11905	15,88
Commd9	COMM domain containing 9	ENSMUSG00000027163	15400	15,88
Secisbp2l	SECIS binding protein 2-like	ENSMUSG00000035093	45885	15,88
Zfp746	zinc finger protein 746	ENSMUSG00000057691	24199	15,88
Ube2u	ubiquitin-conjugating enzyme E2U (putative)	ENSMUSG00000069733	71299	15,88
Kif4	kinesin family member 4	ENSMUSG00000034311	101478	15,80
Rhbdd3	rhomboid domain containing 3	ENSMUSG00000034175	7168	15,80
		ENSMUSG00000069237	8535	15,80
		ENSMUSG00000020803	2469	15,80
Atp8b1	ATPase, class I, type 8B, member 1	ENSMUSG00000039529	132022	15,80
Galnt16	UDP-N-acetyl-alpha-D-galactosamine:polypeptide N-acetylgalactosaminyltransferase 16	ENSMUSG00000021130	84907	15,80
Rbm11	RNA binding motif protein 11	ENSMUSG00000032940	9986	15,80
Dcdc2b	doublecortin domain containing 2b	ENSMUSG00000078552	5927	15,80
Chaf1a	chromatin assembly factor 1, subunit A (p150)	ENSMUSG00000002835	27611	15,80
Zfp865	zinc finger protein 865	ENSMUSG00000074405	12763	15,80
Rpe	ribulose-5-phosphate-3-epimerase	ENSMUSG00000026005	18975	15,80
Tfb2m	transcription factor B2, mitochondrial	ENSMUSG00000026492	18213	15,80
Plscr3	phospholipid scramblase 3	ENSMUSG00000019461	5683	15,80
Nsmce4a	non-SMC element 4 homolog A (S. cerevisiae)	ENSMUSG00000040331	40596	15,80
Ndufb2	NADH dehydrogenase (ubiquinone) 1 beta subcomplex, 2	ENSMUSG00000002416	10809	15,80
Ppp1r37	protein phosphatase 1, regulatory subunit 37	ENSMUSG00000051403	31432	15,80
Kifc3	kinesin family member C3	ENSMUSG00000031788	42709	15,80
Mark4	MAP/microtubule affinity-regulating kinase 4	ENSMUSG00000030397	32420	15,80
Enpp2	ectonucleotide pyrophosphatase/phosphodiesterase 2	ENSMUSG00000022425	81249	15,73
Mrpl20	mitochondrial ribosomal protein L20	ENSMUSG00000029066	6241	15,73
Lsm7	LSM7 homolog, U6 small nuclear RNA associated (S. cerevisiae)	ENSMUSG00000035215	2385	15,73
Taf8	TAF8 RNA polymerase II, TATA box binding protein (TBP)-associated factorq	ENSMUSG00000023980	14238	15,73
Plekha3	pleckstrin homology domain-containing, family A (phosphoinositide binding specific) member 3	ENSMUSG00000002733	21548	15,73
Spon1	spondin 1, (f-spondin) extracellular matrix protein	ENSMUSG00000038156	277373	15,73
Cars2	cysteinyl-tRNA synthetase 2 (mitochondrial)(putative)	ENSMUSG00000056228	36751	15,73
		ENSMUSG00000040195	34149	15,73
Ipo7	importin 7	ENSMUSG00000066232	36690	15,73
Erap1	endoplasmic reticulum aminopeptidase 1	ENSMUSG00000021583	52004	15,73
Il1r1	interleukin 1 receptor, type I	ENSMUSG00000026072	91119	15,73

Ap1ar	adaptor-related protein complex 1 associated regulatory protein	ENSMUSG00000074238	30229	15,73
Cmya5	cardiomyopathy associated 5	ENSMUSG00000047419	104012	15,73
Dgcr14	DiGeorge syndrome critical region gene 14	ENSMUSG00000003527	10638	15,66
		ENSMUSG00000078154	1310	15,66
Mcrs1	microspherule protein 1	ENSMUSG00000037570	9144	15,66
		ENSMUSG00000022967	22204	15,66
		ENSMUSG00000026273	6705	15,66
Haus4	HAUS augmin-like complex, subunit 4	ENSMUSG00000022177	12577	15,66
Atg4d	autophagy related 4D, cysteine peptidase	ENSMUSG00000002820	9070	15,66
Ttll13	tubulin tyrosine ligase-like family, member 13	ENSMUSG00000045467	14446	15,66
Mapk15	mitogen-activated protein kinase 15	ENSMUSG00000063704	5384	15,66
		ENSMUSG00000050312	54375	15,66
		ENSMUSG00000032840	20867	15,66
Zfp184	zinc finger protein 184 (Kruppel-like)	ENSMUSG00000006720	15686	15,66
Adarb1	adenosine deaminase, RNA-specific, B1	ENSMUSG00000020262	127545	15,66
Rnf152	ring finger protein 152	ENSMUSG00000047496	79794	15,66
Piga	phosphatidylinositol glycan anchor biosynthesis, class A	ENSMUSG00000031381	14124	15,66
Gna14	guanine nucleotide binding protein, alpha 14	ENSMUSG00000024697	175152	15,66
Ccdc97	coiled-coil domain containing 97	ENSMUSG00000002608	7983	15,59
Rpp25l	ribonuclease P/MRP 25 subunit-like	ENSMUSG00000036114	1502	15,59
Lysmd1	LysM, putative peptidoglycan-binding, domain containing 1	ENSMUSG00000053769	5431	15,59
Glce	glucuronyl C5-epimerase	ENSMUSG00000032252	13359	15,59
Rgp1	RGP1 retrograde golgi transport homolog (S. cerevisiae)	ENSMUSG00000028468	8773	15,59
Zdhhc24	zinc finger, DHHC domain containing 24	ENSMUSG00000006463	6730	15,59
Nans	N-acetylneuraminic acid synthase (sialic acid synthase)	ENSMUSG00000028334	14385	15,59
Hirip3	HIRA interacting protein 3	ENSMUSG00000042606	3406	15,59
Bmf	BCL2 modifying factor	ENSMUSG00000040093	20931	15,59
Camk4	calcium/calmodulin-dependent protein kinase IV	ENSMUSG00000038128	256727	15,59
Ccdc90b	coiled-coil domain containing 90B	ENSMUSG00000030613	21146	15,59
Scnn1b	sodium channel, nonvoltage-gated 1 beta	ENSMUSG00000030873	53405	15,59
Rab11fip5	RAB11 family interacting protein 5 (class I)	ENSMUSG00000051343	39672	15,51
		ENSMUSG00000048070	17896	15,51
Gm10053	predicted gene 10053	ENSMUSG00000058927	949	15,51
Polr3h	polymerase (RNA) III (DNA directed) polypeptide H	ENSMUSG00000022476	11184	15,51
Spsb3	splA/ryanodine receptor domain and SOCS box containing 3	ENSMUSG00000024160	5510	15,51
Wdr91	WD repeat domain 91	ENSMUSG00000058486	30451	15,51

Tdrkh	tudor and KH domain containing protein	ENSMUSG00000041912	19338	15,51
Prmt10	protein arginine methyltransferase 10 (putative)	ENSMUSG00000037134	31942	15,51
Krr1	KRR1, small subunit (SSU) processome component, homolog (yeast)	ENSMUSG00000063334	15769	15,51
Tmem9	transmembrane protein 9	ENSMUSG00000026411	26812	15,51
Atp13a1	ATPase type 13A1	ENSMUSG00000031862	16587	15,51
Rps6ka6	ribosomal protein S6 kinase polypeptide 6	ENSMUSG00000025665	149768	15,51
Intu	inturned planar cell polarity effector homolog (Drosophila)	ENSMUSG00000060798	173489	15,51
Ddx23	DEAD (Asp-Glu-Ala-Asp) box polypeptide 23	ENSMUSG00000003360	17756	15,51
Sbsn	suprabasin	ENSMUSG00000046056	21396	15,51
		ENSMUSG00000039903	78391	15,51
		ENSMUSG00000073184	17762	15,51
Id4	inhibitor of DNA binding 4	ENSMUSG00000021379	2610	15,44
Zcchc17	zinc finger, CCHC domain containing 17	ENSMUSG00000028772	44561	15,44
BC030307	cDNA sequence BC030307	ENSMUSG00000044937	71033	15,44
Zfp422	zinc finger protein 422	ENSMUSG00000059878	4984	15,44
Fbxo25	F-box protein 25	ENSMUSG00000038365	32716	15,44
Arhgap33	Rho GTPase activating protein 33	ENSMUSG00000036882	11955	15,44
Shroom1	shroom family member 1	ENSMUSG00000018387	10562	15,44
Fam19a5	family with sequence similarity 19, member A5	ENSMUSG00000054863	134107	15,44
Coro7	coronin 7	ENSMUSG00000039637	53645	15,44
Hsdl2	hydroxysteroid dehydrogenase like 2	ENSMUSG00000028383	37127	15,44
		ENSMUSG00000079036	18000	15,44
Tmem179b	transmembrane protein 179B	ENSMUSG00000079437	1946	15,44
Slc30a4	solute carrier family 30 (zinc transporter), member 4	ENSMUSG00000005802	21431	15,44
Golm1	golgi membrane protein 1	ENSMUSG00000021556	40789	15,44
Arl4d	ADP-ribosylation factor-like 4D	ENSMUSG00000034936	2292	15,44
Tubgcp6	tubulin, gamma complex associated protein 6	ENSMUSG00000051786	24731	15,44
Ltf	lactotransferrin	ENSMUSG00000032496	23475	15,37
Furin	furin (paired basic amino acid cleaving enzyme)	ENSMUSG00000030530	16852	15,37
Gid8	GID complex subunit 8 homolog (S. cerevisiae)	ENSMUSG00000027573	8617	15,37
Abtb1	ankyrin repeat and BTB (POZ) domain containing 1	ENSMUSG00000030083	6021	15,37
Nt5c	5',3'-nucleotidase, cytosolic	ENSMUSG00000020736	1443	15,37
4931429I11Rik	RIKEN cDNA 4931429I11 gene	ENSMUSG00000032023	69270	15,37
Nudcd2	NudC domain containing 2	ENSMUSG00000020328	6380	15,37
Ccdc124	coiled-coil domain containing 124	ENSMUSG00000007721	5264	15,37
Orai1	ORAI calcium release-activated calcium modulator 1	ENSMUSG00000049686	15377	15,37
Mfsd11	major facilitator superfamily domain containing 11	ENSMUSG00000020818	23196	15,37
Sipa1l2	signal-induced proliferation-associated 1 like 2	ENSMUSG00000001995	74648	15,37

		ENSMUSG00000038047	33201	15,37
Smyd4	SET and MYND domain containing 4	ENSMUSG00000018809	57273	15,37
Ttc25	tetratricopeptide repeat domain 25	ENSMUSG00000006784	26962	15,37
Fam187b	family with sequence similarity 187, member B	ENSMUSG00000046826	15917	15,37
D17H6S53E	DNA segment, Chr 17, human D6S53E	ENSMUSG00000043311	2483	15,37
Slc15a4	solute carrier family 15, member 4	ENSMUSG00000029416	37234	15,37
Trpc6	transient receptor potential cation channel, subfamily C, member 6	ENSMUSG00000031997	136370	15,37
Arid5a	AT rich interactive domain 5A (MRF1-like)	ENSMUSG00000037447	16297	15,37
Arfgap2	ADP-ribosylation factor GTPase activating protein 2	ENSMUSG00000027255	11958	15,37
Agmo	alkylglycerol monooxygenase	ENSMUSG00000050103	340562	15,37
		ENSMUSG00000062083	612	15,30
		ENSMUSG00000091471	9110	15,30
Tmed1	transmembrane emp24 domain containing 1	ENSMUSG00000032180	2807	15,30
Dnm3	dynamin 3	ENSMUSG00000040265	495582	15,30
Actr5	ARP5 actin-related protein 5	ENSMUSG00000037761	14324	15,30
		ENSMUSG00000014075	9398	15,30
Cxxc4	CXXC finger 4	ENSMUSG00000044365	25600	15,30
Phkg2	phosphorylase kinase, gamma 2 (testis)	ENSMUSG00000030815	9966	15,30
Zfp597	zinc finger protein 597	ENSMUSG00000039789	26241	15,30
Plekhg5	pleckstrin homology domain containing, family G (with RhoGef domain) member 5	ENSMUSG00000039713	42903	15,30
		ENSMUSG00000029179	49257	15,30
Brms1	breast cancer metastasis-suppressor 1	ENSMUSG00000080268	8514	15,30
Ifi35	interferon-induced protein 35	ENSMUSG00000010358	10292	15,30
Adam9	a disintegrin and metallopeptidase domain 9 (meltrin gamma)	ENSMUSG00000031555	67312	15,30
Fbxl4	F-box and leucine-rich repeat protein 4	ENSMUSG00000040410	76549	15,30
Il15ra	interleukin 15 receptor, alpha chain	ENSMUSG00000023206	28700	15,30
Kcnh1	potassium voltage-gated channel, subfamily H (eag-related), member 1	ENSMUSG00000058248	319376	15,30
1700101E01Rik	RIKEN cDNA 1700101E01 gene	ENSMUSG00000079502	99692	15,30
Svip	small VCP/p97-interacting protein	ENSMUSG00000074093	8854	15,22
Cyb561d2	cytochrome b-561 domain containing 2	ENSMUSG00000037190	2855	15,22
Qsox2	quiescin Q6 sulfhydryl oxidase 2	ENSMUSG00000036327	28889	15,22
Cap2	CAP, adenylate cyclase-associated protein, 2 (yeast)	ENSMUSG00000021373	147795	15,22
Wdr12	WD repeat domain 12	ENSMUSG00000026019	21778	15,22
Dffa	DNA fragmentation factor, alpha subunit	ENSMUSG00000028974	16502	15,22
Ddx49	DEAD (Asp-Glu-Ala-Asp) box polypeptide 49	ENSMUSG00000057788	9624	15,22

Pop7	processing of precursor 7, ribonuclease P family, (S. cerevisiae)	ENSMUSG00000029715	1081	15,22
Lca5l	Leber congenital amaurosis 5-like	ENSMUSG00000045275	33851	15,22
Tmem39a	transmembrane protein 39a	ENSMUSG00000002845	33465	15,22
Ercc6	excision repair cross-complementing rodent repair deficiency, complementation group 6	ENSMUSG00000054051	67469	15,22
Hmgb3	high mobility group box 3	ENSMUSG00000015217	4759	15,15
Fam81a	family with sequence similarity 81, member A	ENSMUSG00000032224	53251	15,15
Sap30	sin3 associated polypeptide	ENSMUSG00000031609	5154	15,15
P2rx6	purinergic receptor P2X, ligand-gated ion channel, 6	ENSMUSG00000022758	15916	15,15
Ttc9	tetratricopeptide repeat domain 9	ENSMUSG00000042734	33573	15,15
Zfp646	zinc finger protein 646	ENSMUSG00000049739	9201	15,15
Adprhl2	ADP-ribosylhydrolase like 2	ENSMUSG00000042558	5657	15,15
		ENSMUSG00000079709	12183	15,15
Arhgef4	Rho guanine nucleotide exchange factor (GEF) 4	ENSMUSG00000037509	135122	15,15
Snx33	sorting nexin 33	ENSMUSG00000032733	11172	15,15
Crem	cAMP responsive element modulator	ENSMUSG00000063889	71701	15,15
Polk	polymerase (DNA directed), kappa	ENSMUSG00000021668	61797	15,15
Kctd12b	potassium channel tetramerisation domain containing 12b	ENSMUSG00000041633	11238	15,15
		ENSMUSG00000095463	18153	15,15
Ubxn10	UBX domain protein 10	ENSMUSG00000043621	27331	15,15
Slc31a2	solute carrier family 31, member 2	ENSMUSG00000066152	35850	15,08
Trp53i13	transformation related protein 53 inducible protein 13	ENSMUSG00000044328	7882	15,08
Mettl10	methyltransferase like 10	ENSMUSG00000030960	25217	15,08
Gtpbp3	GTP binding protein 3	ENSMUSG00000007610	11481	15,08
Mcm4	minichromosome maintenance deficient 4 homolog (S. cerevisiae)	ENSMUSG00000022673	13504	15,08
Tbp	TATA box binding protein	ENSMUSG00000014767	28492	15,08
Cyp2b13	cytochrome P450, family 2, subfamily b, polypeptide 13	ENSMUSG00000040583	34702	15,08
Sgol1	shugoshin-like 1 (S. pombe)	ENSMUSG00000023940	14548	15,08
Pfas	phosphoribosylformylglycinamidine synthase (FGAR amidotransferase)	ENSMUSG00000020899	22764	15,08
Tmem35	transmembrane protein 35	ENSMUSG00000033578	10745	15,08
Ssfa2	sperm specific antigen 2	ENSMUSG00000027007	37615	15,08
Rasgrp4	RAS guanyl releasing protein 4	ENSMUSG00000030589	19108	15,01
		ENSMUSG00000033157	13231	15,01
Fdx1l	ferredoxin 1-like	ENSMUSG00000079677	5995	15,01
Psmc5	protease (prosome, macropain) 26S subunit, ATPase 5	ENSMUSG00000020708	6967	15,01
Tmem129	transmembrane protein 129	ENSMUSG00000019295	4762	15,01
Fbxo46	F-box protein 46	ENSMUSG00000050428	18403	15,01
Prpf40b	PRP40 pre-mRNA processing factor 40 homolog B (yeast)	ENSMUSG00000023007	21932	15,01
Tmem186	transmembrane protein 186	ENSMUSG00000043140	3829	15,01
Lrrc43	leucine rich repeat containing 43	ENSMUSG00000063409	18881	15,01

Zfp71-rs1	zinc finger protein 71, related sequence	ENSMUSG00000071281	23814	15,01
Dnase2a	deoxyribonuclease II alpha	ENSMUSG00000003812	14356	15,01
3110082I17Rik	RIKEN cDNA 3110082I17 gene	ENSMUSG00000053553	100764	15,01
Cep44	centrosomal protein 44	ENSMUSG00000038215	19526	15,01
Hccs	holocytochrome c synthetase	ENSMUSG00000031352	70180	15,01
Efhc2	EF-hand domain (C-terminal) containing 2	ENSMUSG00000025038	187320	15,01
Scg2	secretogranin II	ENSMUSG00000050711	5422	14,93
Cxx1a	CAAX box 1A	ENSMUSG00000067925	1276	14,93
		ENSMUSG00000063021	513	14,93
LOC101056131	uncharacterized LOC101056131	ENSMUSG00000047692	65656	14,93
Ptn	pleiotrophin	ENSMUSG00000029838	95699	14,93
Klc3	kinesin light chain 3	ENSMUSG00000040714	9668	14,93
Grik5	glutamate receptor, ionotropic, kainate 5 (gamma 2)	ENSMUSG00000003378	62517	14,93
Tk2	thymidine kinase 2, mitochondrial	ENSMUSG00000035824	21868	14,93
Sertad3	SERTA domain containing 3	ENSMUSG00000055200	3592	14,93
Jrkl	jerky homolog-like (mouse)	ENSMUSG00000079083	2952	14,93
Capn1	calpain 1	ENSMUSG00000024942	27280	14,93
Nrg4	neuregulin 4	ENSMUSG00000032311	106623	14,93
Parp2	poly (ADP-ribose) polymerase family, member 2	ENSMUSG00000036023	13355	14,93
Parp4	poly (ADP-ribose) polymerase family, member 4	ENSMUSG00000054509	84176	14,93
Tap1	transporter 1, ATP-binding cassette, sub-family B (MDR/TAP)	ENSMUSG00000037321	9673	14,93
		ENSMUSG00000047036	10215	14,93
		ENSMUSG00000041078	763555	14,93
Plekhg6	pleckstrin homology domain containing, family G (with RhoGef domain) member 6	ENSMUSG00000038167	18134	14,93
Mrps18a	mitochondrial ribosomal protein S18A	ENSMUSG00000023967	17925	14,86
Pomt2	protein-O-mannosyltransferase 2	ENSMUSG00000034126	41037	14,86
BC003965	cDNA sequence BC003965	ENSMUSG00000067722	3101	14,86
Naaa	N-acylethanolamine acid amidase	ENSMUSG00000029413	20512	14,86
Ncdn	neurochondrin	ENSMUSG00000028833	9689	14,86
Polr2k	polymerase (RNA) II (DNA directed) polypeptide K	ENSMUSG00000045996	3001	14,86
Tmem223	transmembrane protein 223	ENSMUSG00000075043	1480	14,86
Hdac6	histone deacetylase 6	ENSMUSG00000031161	17770	14,86
Hspbap1	Hspb associated protein 1	ENSMUSG00000022849	58061	14,86
Atp2a3	ATPase, Ca++ transporting, ubiquitous	ENSMUSG00000020788	31876	14,86
Tirap	toll-interleukin 1 receptor (TIR) domain-containing adaptor protein	ENSMUSG00000032041	15741	14,86
Fhl2	four and a half LIM domains 2	ENSMUSG00000008136	40888	14,86
Acrbp	proacrosin binding protein	ENSMUSG00000072770	13579	14,86
Plin2	perilipin 2	ENSMUSG00000028494	21675	14,86
Pcbd2	pterin 4 alpha carbinolamine dehydratase/dimerization cofactor of hepatocyte nuclear factor 1 alpha (TCF1) 2	ENSMUSG00000021496	49463	14,86
Csmd3	CUB and Sushi multiple domains 3	ENSMUSG00000022311	1211427	14,86
Gm12216	predicted gene 12216	ENSMUSG00000081769	75839	14,86

		ENSMUSG00000031802	1376	14,86
Itga1	integrin alpha 1	ENSMUSG00000042284	143886	14,79
Thumpd1	THUMP domain containing 1	ENSMUSG00000030942	5670	14,79
Erf	Ets2 repressor factor	ENSMUSG00000040857	8201	14,79
Pdpn	podoplanin	ENSMUSG00000028583	32134	14,79
Zfp2	zinc finger protein 2	ENSMUSG00000049321	17454	14,79
Rhobtb3	Rho-related BTB domain containing 3	ENSMUSG00000021589	74389	14,79
Ankrd46	ankyrin repeat domain 46	ENSMUSG00000048307	19124	14,79
Cinp	cyclin-dependent kinase 2 interacting protein	ENSMUSG00000021276	16536	14,79
		ENSMUSG00000091474	29273	14,79
Cep41	centrosomal protein 41	ENSMUSG00000029790	40293	14,79
Edil3	EGF-like repeats and discoidin I-like domains 3	ENSMUSG00000034488	501752	14,79
Vwa5a	von Willebrand factor A domain containing 5A	ENSMUSG00000023186	25070	14,79
Tmc7	transmembrane channel-like gene family 7	ENSMUSG00000042246	48894	14,79
1700016C15Rik	RIKEN cDNA 1700016C15 gene	ENSMUSG00000015962	23511	14,79
Dclk3	doublecortin-like kinase 3	ENSMUSG00000032500	50528	14,72
Aifm2	apoptosis-inducing factor, mitochondrion-associated 2	ENSMUSG00000020085	23998	14,72
Ccdc151	coiled-coil domain containing 151	ENSMUSG00000039632	12764	14,72
Gpatch4	G patch domain containing 4	ENSMUSG00000028069	12886	14,72
Pdk1	pyruvate dehydrogenase kinase, isoenzyme 1	ENSMUSG00000006494	30635	14,72
Chst1	carbohydrate (keratan sulfate Gal-6) sulfotransferase 1	ENSMUSG00000027221	15544	14,72
Coil	coilin	ENSMUSG00000033983	21362	14,72
Emp2	epithelial membrane protein 2	ENSMUSG00000022505	32220	14,72
Pxdn	peroxidasin homolog (Drosophila)	ENSMUSG00000020674	79623	14,72
Xab2	XPA binding protein 2	ENSMUSG00000019470	12896	14,72
4931422A03Rik	RIKEN cDNA 4931422A03 gene	ENSMUSG00000046085	61419	14,72
Snx7	sorting nexin 7	ENSMUSG00000028007	87440	14,72
Malsu1	mitochondrial assembly of ribosomal large subunit 1	ENSMUSG00000029815	12957	14,72
Dab1	disabled 1	ENSMUSG00000028519	1125486	14,72
Cyp4a12b	cytochrome P450, family 4, subfamily a, polypeptide 12B	ENSMUSG00000078597	27411	14,72
Lsm10	U7 snRNP-specific Sm-like protein LSM10	ENSMUSG00000050188	1962	14,64
Col9a2	collagen, type IX, alpha 2	ENSMUSG00000028626	15938	14,64
Atxn7l3	ataxin 7-like 3	ENSMUSG00000059995	7332	14,64
Maz	MYC-associated zinc finger protein (purine-binding transcription factor)	ENSMUSG00000030678	4337	14,64
Gpx8	glutathione peroxidase 8 (putative)	ENSMUSG00000021760	3626	14,64
1700011I03Rik	RIKEN cDNA 1700011I03 gene	ENSMUSG00000058925	197286	14,64
AW549877	expressed sequence AW549877	ENSMUSG00000041935	13717	14,64
Synpo2	synaptopodin 2	ENSMUSG00000050315	159629	14,64
Dtx3l	deltex 3-like (Drosophila)	ENSMUSG00000049502	12641	14,64
Vangl1	vang-like 1 (van gogh, Drosophila)	ENSMUSG00000027860	47993	14,64
		ENSMUSG00000021720	121688	14,64
Mex3b	mex3 homolog B (C. elegans)	ENSMUSG00000057706	4231	14,57
Slc35c2	solute carrier family 35, member C2	ENSMUSG00000017664	11316	14,57

E130309D02Rik	RIKEN cDNA E130309D02 gene	ENSMUSG00000039244	14166	14,57
Reln	reelin	ENSMUSG00000042453	460249	14,57
Vps37b	vacuolar protein sorting 37B (yeast)	ENSMUSG00000066278	27620	14,57
Map1s	microtubule-associated protein 1S	ENSMUSG00000019261	11553	14,57
Cuta	cutA divalent cation tolerance homolog (E. coli)	ENSMUSG00000024194	1506	14,57
Ticam1	toll-like receptor adaptor molecule 1	ENSMUSG00000047123	7468	14,57
Lrrc8e	leucine rich repeat containing 8 family, member E	ENSMUSG00000046589	10644	14,57
Plch2	phospholipase C, eta 2	ENSMUSG00000029055	73670	14,57
Arhgap15	Rho GTPase activating protein 15	ENSMUSG00000049744	647130	14,57
Dnali1	dynein, axonemal, light intermediate polypeptide 1	ENSMUSG00000042707	10366	14,57
Adrb2	adrenergic receptor, beta 2	ENSMUSG00000045730	2143	14,57
		ENSMUSG00000084989	15174	14,57
Sdf2l1	stromal cell-derived factor 2-like 1	ENSMUSG00000022769	2246	14,50
Akna	AT-hook transcription factor	ENSMUSG00000039158	36230	14,50
		ENSMUSG00000034059	4598	14,50
Zfp771	zinc finger protein 771	ENSMUSG00000054716	10278	14,50
Zfp324	zinc finger protein 324	ENSMUSG00000004500	8399	14,50
C1qtnf4	C1q and tumor necrosis factor related protein 4	ENSMUSG00000040794	4666	14,50
Tmem168	transmembrane protein 168	ENSMUSG00000029569	27414	14,50
Arhgap11a	Rho GTPase activating protein 11A	ENSMUSG00000041219	17170	14,50
Edem1	ER degradation enhancer, mannosidase alpha-like 1	ENSMUSG00000030104	30716	14,50
Hmces	5-hydroxymethylcytosine (hmC) binding, ES cell specific	ENSMUSG00000030060	22695	14,50
Tctn3	tectonic family member 3	ENSMUSG00000025008	15788	14,50
Mtm1	X-linked myotubular myopathy gene 1	ENSMUSG00000031337	104925	14,50
Ovol1	OVO homolog-like 1 (Drosophila)	ENSMUSG00000024922	11439	14,50
Ctxn3	cortexin 3	ENSMUSG00000069372	9649	14,43
Sac3d1	SAC3 domain containing 1	ENSMUSG00000024790	2583	14,43
Kcnh3	potassium voltage-gated channel, subfamily H (eag-related), member 3	ENSMUSG00000037579	17842	14,43
Fbl	fibrillarin	ENSMUSG00000046865	9522	14,43
Rsl24d1	ribosomal L24 domain containing 1	ENSMUSG00000032215	9908	14,43
Crcp	calcitonin gene-related peptide-receptor component protein	ENSMUSG00000025532	31500	14,43
Slc25a15	solute carrier family 25 (mitochondrial carrier ornithine transporter), member 15	ENSMUSG00000031482	23068	14,43
Surf6	surfeit gene 6	ENSMUSG00000036160	14252	14,43
		ENSMUSG00000014245	55958	14,43
Mon1b	MON1 homolog b (yeast)	ENSMUSG00000078908	9593	14,43
Rap2c	RAP2C, member of RAS oncogene family	ENSMUSG00000050029	14107	14,43
Tm2d1	TM2 domain containing 1	ENSMUSG00000028563	27937	14,43
Cbx7	chromobox 7	ENSMUSG00000053411	55313	14,43
Dpp6	dipeptidylpeptidase 6	ENSMUSG00000061576	910303	14,43
2700062C07Rik	RIKEN cDNA 2700062C07 gene	ENSMUSG00000024273	6905	14,35
Pik3cd	phosphatidylinositol 3-kinase catalytic delta polypeptide	ENSMUSG00000039936	53404	14,35

Mns1	meiosis-specific nuclear structural protein 1	ENSMUSG00000032221	20046	14,35
Ptgr2	prostaglandin reductase 2	ENSMUSG00000072946	30601	14,35
Decr2	2-4-dienoyl-Coenzyme A reductase 2, peroxisomal	ENSMUSG00000036775	9119	14,35
Zfp74	zinc finger protein 74	ENSMUSG00000059975	23275	14,35
Ormdl1	ORM1-like 1 (S. cerevisiae)	ENSMUSG00000026097	13215	14,35
AI597479	expressed sequence AI597479	ENSMUSG00000010290	17238	14,35
Pomk	protein-O-mannose kinase	ENSMUSG00000037251	13518	14,35
Rab40b	Rab40b, member RAS oncogene family	ENSMUSG00000025170	32127	14,35
Pdcd2l	programmed cell death 2-like	ENSMUSG00000002635	12163	14,35
Gm12942	predicted gene 12942	ENSMUSG00000070737	5954	14,35
		ENSMUSG00000053399	151490	14,35
Hace1	HECT domain and ankyrin repeat containing, E3 ubiquitin protein ligase 1	ENSMUSG00000038822	134517	14,35
Orai3	ORAI calcium release-activated calcium modulator 3	ENSMUSG00000043964	5336	14,28
Nr2f6	nuclear receptor subfamily 2, group F, member 6	ENSMUSG00000002393	7838	14,28
Ndufaf2	NADH dehydrogenase (ubiquinone) 1 alpha subcomplex, assembly factor 2	ENSMUSG00000068184	106036	14,28
Fam124a	family with sequence similarity 124, member A	ENSMUSG00000035184	52750	14,28
		ENSMUSG00000035228	4634	14,28
Fuz	fuzzy homolog (Drosophila)	ENSMUSG00000011658	6553	14,28
Smim19	small integral membrane protein 19	ENSMUSG00000031534	14265	14,28
Pafah2	platelet-activating factor acetylhydrolase 2	ENSMUSG00000037366	31094	14,28
Spns2	spinster homolog 2	ENSMUSG00000040447	38267	14,28
Mina	myc induced nuclear antigen	ENSMUSG00000022724	20687	14,28
		ENSMUSG00000092367	4771	14,28
Zc3h3	zinc finger CCCH type containing 3	ENSMUSG00000075600	87484	14,28
Nme9	NME/NM23 family member 9	ENSMUSG00000046242	18509	14,28
Isl1	ISL1 transcription factor, LIM/homeodomain	ENSMUSG00000042258	11408	14,28
Lage3	L antigen family, member 3	ENSMUSG00000015289	2343	14,21
Tvp23a	trans-golgi network vesicle protein 23A	ENSMUSG00000050908	26806	14,21
Casp9	caspase 9	ENSMUSG00000028914	22365	14,21
Tnk2	tyrosine kinase, non-receptor, 2	ENSMUSG00000022791	39620	14,21
Cdkn2aip	CDKN2A interacting protein	ENSMUSG00000038069	4588	14,21
Sc5d	sterol-C5-desaturase (fungal ERG3, delta-5-desaturase) homolog (S. cerevisae)	ENSMUSG00000032018	10124	14,21
Tab1	TGF-beta activated kinase 1/MAP3K7 binding protein 1	ENSMUSG00000022414	28548	14,21
Telo2	TEL2, telomere maintenance 2, homolog (S. cerevisiae)	ENSMUSG00000024170	16398	14,21
Zfp408	zinc finger protein 408	ENSMUSG00000075040	6123	14,21
Tubgcp5	tubulin, gamma complex associated protein 5	ENSMUSG00000033790	37300	14,21
		ENSMUSG00000044835	26416	14,21

Gabrb3	gamma-aminobutyric acid (GABA) A receptor, subunit beta 3	ENSMUSG00000033676	238285	14,21
Sh3d21	SH3 domain containing 21	ENSMUSG00000073758	12890	14,21
Mylk	myosin, light polypeptide kinase	ENSMUSG00000022836	257211	14,14
Tbc1d24	TBC1 domain family, member 24	ENSMUSG00000036473	30132	14,14
Bap1	Brca1 associated protein 1	ENSMUSG00000021901	8441	14,14
Zfp830	zinc finger protein 830	ENSMUSG00000046010	3278	14,14
Apip	APAF1 interacting protein	ENSMUSG00000010911	18970	14,14
Tchp	trichoplein, keratin filament binding	ENSMUSG00000002486	14568	14,14
Eef1e1	eukaryotic translation elongation factor 1 epsilon 1	ENSMUSG00000001707	13331	14,14
9130023H24Rik	RIKEN cDNA 9130023H24 gene	ENSMUSG00000062944	3581	14,14
Pigc	phosphatidylinositol glycan anchor biosynthesis, class C	ENSMUSG00000026698	57921	14,14
Phf7	PHD finger protein 7	ENSMUSG00000021902	13523	14,14
Cadm4	cell adhesion molecule 4	ENSMUSG00000054793	22517	14,14
D8Ertd738e	DNA segment, Chr 8, ERATO Doi 738, expressed	ENSMUSG00000019362	3525	14,14
Traf6	TNF receptor-associated factor 6	ENSMUSG00000027164	23241	14,14
Dnajc17	DnaJ (Hsp40) homolog, subfamily C, member 17	ENSMUSG00000034278	36296	14,14
Alg1	asparagine-linked glycosylation 1 (beta-1,4-mannosyltransferase)	ENSMUSG00000039427	11292	14,14
Ilvbl	ilvB (bacterial acetolactate synthase)-like	ENSMUSG00000032763	9999	14,14
Alg6	asparagine-linked glycosylation 6 (alpha-1,3,-glucosyltransferase)	ENSMUSG00000073792	47797	14,14
Klhdc8a	kelch domain containing 8A	ENSMUSG00000042115	8732	14,14
Egln3	EGL nine homolog 3 (C. elegans)	ENSMUSG00000035105	24880	14,14
Polr2f	polymerase (RNA) II (DNA directed) polypeptide F	ENSMUSG00000033020	10424	14,06
		ENSMUSG00000096951	32134	14,06
		ENSMUSG00000043162	5503	14,06
4930432K21Rik	RIKEN cDNA 4930432K21 gene	ENSMUSG00000008129	24566	14,06
Pigm	phosphatidylinositol glycan anchor biosynthesis, class M	ENSMUSG00000050229	7569	14,06
		ENSMUSG00000043090	29853	14,06
Cth	cystathionase (cystathionine gamma-lyase)	ENSMUSG00000028179	30830	14,06
Snx15	sorting nexin 15	ENSMUSG00000024787	8906	14,06
Rbks	ribokinase	ENSMUSG00000029136	73189	14,06
Spry1	sprouty homolog 1 (Drosophila)	ENSMUSG00000037211	4652	14,06
Timd2	T cell immunoglobulin and mucin domain containing 2	ENSMUSG00000040413	38102	14,06
Gsto1	glutathione S-transferase omega 1	ENSMUSG00000025068	9821	14,06
1700001C02Rik	RIKEN cDNA 1700001C02 gene	ENSMUSG00000029182	18042	14,06
Cldn8	claudin 8	ENSMUSG00000050520	2356	14,06
Pygm	muscle glycogen phosphorylase	ENSMUSG00000032648	14061	14,06
Tmem147	transmembrane protein 147	ENSMUSG00000006315	1834	13,99
Kazald1	Kazal-type serine peptidase inhibitor domain 1	ENSMUSG00000025213	4049	13,99
Fblim1	filamin binding LIM protein 1	ENSMUSG00000006219	30035	13,99
Tmem17	transmembrane protein 17	ENSMUSG00000049904	7147	13,99
Kat2a	K(lysine) acetyltransferase 2A	ENSMUSG00000020918	7720	13,99
Clspn	claspin	ENSMUSG00000042489	36969	13,99
		ENSMUSG00000052419	3784	13,99

D030056L22Rik	RIKEN cDNA D030056L22 gene	ENSMUSG00000047044	5236	13,99
Pih1d1	PIH1 domain containing 1	ENSMUSG00000003423	5767	13,99
		ENSMUSG00000004393	8854	13,99
Vps39	vacuolar protein sorting 39 (yeast)	ENSMUSG00000027291	36677	13,99
		ENSMUSG00000040563	11868	13,99
Dbf4	DBF4 homolog (S. cerevisiae)	ENSMUSG00000002297	25744	13,99
Aifm3	apoptosis-inducing factor, mitochondrion-associated 3	ENSMUSG00000022763	17875	13,99
C1qbp	complement component 1, q subcomponent binding protein	ENSMUSG00000018446	5191	13,92
Prim1	DNA primase, p49 subunit	ENSMUSG00000025395	14870	13,92
Caskin1	CASK interacting protein 1	ENSMUSG00000033597	20123	13,92
Vmn2r59	vomeronasal 2, receptor 59	ENSMUSG00000092032	47190	13,92
AW209491	expressed sequence AW209491	ENSMUSG00000039182	7958	13,92
Esrra	estrogen related receptor, alpha	ENSMUSG00000024955	10837	13,92
Commd6	COMM domain containing 6	ENSMUSG00000075486	6921	13,92
Sardh	sarcosine dehydrogenase	ENSMUSG00000009614	59945	13,92
Slc6a17	solute carrier family 6 (neurotransmitter transporter), member 17	ENSMUSG00000027894	50476	13,92
6330408A02Rik	RIKEN cDNA 6330408A02 gene	ENSMUSG00000070814	19755	13,92
		ENSMUSG00000022861	188761	13,92
Rnf123	ring finger protein 123	ENSMUSG00000041528	31813	13,92
Zfp622	zinc finger protein 622	ENSMUSG00000052253	14117	13,92
Ints12	integrator complex subunit 12	ENSMUSG00000028016	19149	13,92
Pdzk1ip1	PDZK1 interacting protein 1	ENSMUSG00000028716	5192	13,92
Trim68	tripartite motif-containing 68	ENSMUSG00000073968	9747	13,92
Slc9a3	solute carrier family 9 (sodium/hydrogen exchanger), member 3	ENSMUSG00000036123	44550	13,92
Mpi	mannose phosphate isomerase	ENSMUSG00000032306	8508	13,85
Trim59	tripartite motif-containing 59	ENSMUSG00000034317	9468	13,85
Hist2h3c1	histone cluster 2, H3c1	ENSMUSG00000093769	1825	13,85
Gmppa	GDP-mannose pyrophosphorylase A	ENSMUSG00000033021	7250	13,85
Tmem150b	transmembrane protein 150B	ENSMUSG00000046456	18418	13,85
		ENSMUSG00000039810	5812	13,85
Lonrf2	LON peptidase N-terminal domain and ring finger 2	ENSMUSG00000048814	42422	13,85
		ENSMUSG00000043257	12261	13,85
		ENSMUSG00000079109	27346	13,85
Sytl2	synaptotagmin-like 2	ENSMUSG00000030616	61740	13,85
Tfam	transcription factor A, mitochondrial	ENSMUSG00000003923	12817	13,85
Ccdc93	coiled-coil domain containing 93	ENSMUSG00000026339	75412	13,85
Opcml	opioid binding protein/cell adhesion molecule-like	ENSMUSG00000062257	1134636	13,85
Qpctl	glutaminyl-peptide cyclotransferase-like	ENSMUSG00000030407	8980	13,85
Pitpnm2	phosphatidylinositol transfer protein, membrane-associated 2	ENSMUSG00000029406	131071	13,85
		ENSMUSG00000062093	2244	13,85
		ENSMUSG00000090486	14158	13,85
Acadsb	acyl-Coenzyme A dehydrogenase, short/branched chain	ENSMUSG00000030861	35611	13,85

Kcnj3	potassium inwardly-rectifying channel, subfamily J, member 3	ENSMUSG00000026824	162176	13,85
Chchd4	coiled-coil-helix-coiled-coil-helix domain containing 4	ENSMUSG00000034203	9148	13,77
		ENSMUSG00000031302	26796	13,77
Fkrp	fukutin related protein	ENSMUSG00000048920	7463	13,77
Kctd7	potassium channel tetramerisation domain containing 7	ENSMUSG00000034110	10946	13,77
Ccna2	cyclin A2	ENSMUSG00000027715	7285	13,77
Ccm2	cerebral cavernous malformation 2	ENSMUSG00000000378	49858	13,77
Cenph	centromere protein H	ENSMUSG00000045273	16226	13,77
Hepacam2	HEPACAM family member 2	ENSMUSG00000044156	37403	13,77
A430005L14Rik	RIKEN cDNA A430005L14 gene	ENSMUSG00000047613	4689	13,77
		ENSMUSG00000098140	18430	13,77
Gm10643	predicted gene 10643	ENSMUSG00000074215	1577	13,77
		ENSMUSG00000091041	19951	13,77
Tsen2	tRNA splicing endonuclease 2 homolog (S. cerevisiae)	ENSMUSG00000042389	33687	13,77
Klc4	kinesin light chain 4	ENSMUSG00000003546	14514	13,77
Atpaf1	ATP synthase mitochondrial F1 complex assembly factor 1	ENSMUSG00000028710	34017	13,77
Vmn2r29	vomeronasal 2, receptor 29	ENSMUSG00000095730	46963	13,77
Zfp280c	zinc finger protein 280C	ENSMUSG00000036916	52880	13,77
Fam131a	family with sequence similarity 131, member A	ENSMUSG00000050821	9767	13,70
Ogfod2	2-oxoglutarate and iron-dependent oxygenase domain containing 2	ENSMUSG00000023707	3187	13,70
C77080	expressed sequence C77080	ENSMUSG00000050390	41827	13,70
		ENSMUSG00000024654	25769	13,70
Dis3l	DIS3 mitotic control homolog (S. cerevisiae)-like	ENSMUSG00000032396	34533	13,70
Ly6h	lymphocyte antigen 6 complex, locus H	ENSMUSG00000022577	15988	13,70
Mrp63	mitochondrial ribosomal protein 63	ENSMUSG00000021967	2507	13,70
Sema4g	sema domain, immunoglobulin domain (Ig), transmembrane domain (TM) and short cytoplasmic domain, (semaphorin) 4G	ENSMUSG00000025207	14297	13,70
Ddx39	DEAD (Asp-Glu-Ala-Asp) box polypeptide 39	ENSMUSG00000005481	11716	13,70
Pacsin1	protein kinase C and casein kinase substrate in neurons 1	ENSMUSG00000040276	55598	13,70
Kif23	kinesin family member 23	ENSMUSG00000032254	29520	13,70
Pus1	pseudouridine synthase 1	ENSMUSG00000029507	6993	13,70
		ENSMUSG00000039483	7237	13,70
Vwa9	von Willebrand factor A domain containing 9	ENSMUSG00000034263	26143	13,70
Mtfp1	mitochondrial fission process 1	ENSMUSG00000004748	3966	13,70
Miip	migration and invasion inhibitory protein	ENSMUSG00000029022	8039	13,70
Cdpf1	cysteine rich, DPF motif domain containing 1	ENSMUSG00000064284	4726	13,70
Sox13	SRY-box containing gene 13	ENSMUSG00000070643	42075	13,70
Parp12	poly (ADP-ribose) polymerase family, member 12	ENSMUSG00000038507	31940	13,70

Tvp23b	trans-golgi network vesicle protein 23B	ENSMUSG00000014177	37326	13,70
		ENSMUSG00000094103	14544	13,70
Rab15	RAB15, member RAS oncogene family	ENSMUSG00000021062	24949	13,70
Acpp	acid phosphatase, prostate	ENSMUSG00000032561	49478	13,70
Slc16a4	solute carrier family 16 (monocarboxylic acid transporters), member 4	ENSMUSG00000027896	20886	13,70
		ENSMUSG00000003746	171009	13,70
Atcay	ataxia, cerebellar, Cayman type homolog (human)	ENSMUSG00000034958	26326	13,63
Nxph3	neurexophilin 3	ENSMUSG00000046719	4726	13,63
Vdac3-ps1	voltage-dependent anion channel 3, pseudogene 1	ENSMUSG00000075053	1306	13,63
Orc6	origin recognition complex, subunit 6	ENSMUSG00000031697	8647	13,63
		ENSMUSG00000091405	1608	13,63
Cdca2	cell division cycle associated 2	ENSMUSG00000048922	39511	13,63
Pard6g	par-6 partitioning defective 6 homolog gamma (C. elegans)	ENSMUSG00000056214	72746	13,63
		ENSMUSG00000045854	1307	13,63
Tprn	taperin	ENSMUSG00000048707	7268	13,63
Nol6	nucleolar protein family 6 (RNA-associated)	ENSMUSG00000028430	10029	13,63
Heatr5b	HEAT repeat containing 5B	ENSMUSG00000039414	82476	13,63
		ENSMUSG00000094143	979	13,63
Tspan33	tetraspanin 33	ENSMUSG00000001763	24338	13,63
BC051070	cDNA sequence BC051070	ENSMUSG00000027902	18795	13,56
Gorasp1	golgi reassembly stacking protein 1	ENSMUSG00000032513	11886	13,56
Nob1	NIN1/RPN12 binding protein 1 homolog (S. cerevisiae)	ENSMUSG00000003848	12566	13,56
Taf9	TAF9 RNA polymerase II, TATA box binding protein (TBP)-associated factor	ENSMUSG00000052293	4717	13,56
Actl6a	actin-like 6A	ENSMUSG00000027671	18423	13,56
Polr3g	polymerase (RNA) III (DNA directed) polypeptide G	ENSMUSG00000035834	37171	13,56
Exosc7	exosome component 7	ENSMUSG00000025785	22915	13,56
Lrp2bp	Lrp2 binding protein	ENSMUSG00000031637	30175	13,56
Jam3	junction adhesion molecule 3	ENSMUSG00000031990	58037	13,56
Slmo1	slowmo homolog 1 (Drosophila)	ENSMUSG00000024530	15733	13,56
Tmem194b	transmembrane protein 194B	ENSMUSG00000043015	21215	13,56
Mboat7	membrane bound O-acyltransferase domain containing 7	ENSMUSG00000035596	15734	13,56
Tead4	TEA domain family member 4	ENSMUSG00000030353	76536	13,56
Prrg1	proline rich Gla (G-carboxyglutamic acid) 1	ENSMUSG00000047996	134284	13,56
		ENSMUSG00000023505	4155	13,48
Cxx1b	CAAX box 1B	ENSMUSG00000067924	1231	13,48
Dlc1	deleted in liver cancer 1	ENSMUSG00000031523	385393	13,48
Lmnb2	lamin B2	ENSMUSG00000062075	17043	13,48
Pip4k2c	phosphatidylinositol-5-phosphate 4-kinase, type II, gamma	ENSMUSG00000025417	14556	13,48

Chchd5	coiled-coil-helix-coiled-coil-helix domain containing 5	ENSMUSG00000037938	4435	13,48
Pde2a	phosphodiesterase 2A, cGMP-stimulated	ENSMUSG00000030653	91139	13,48
Rbbp5	retinoblastoma binding protein 5	ENSMUSG00000026439	28299	13,48
Fam214b	family with sequence similarity 214, member B	ENSMUSG00000036002	13807	13,48
Inpp5b	inositol polyphosphate-5-phosphatase B	ENSMUSG00000028894	59662	13,48
Adamts1	a disintegrin-like and metallopeptidase (reprolysin type) with thrombospondin type 1 motif, 1	ENSMUSG00000022893	9287	13,48
Spink10	serine peptidase inhibitor, Kazal type 10	ENSMUSG00000044176	112456	13,48
Khnyn	KH and NYN domain containing	ENSMUSG00000047153	11813	13,48
Stat6	signal transducer and activator of transcription 6	ENSMUSG00000002147	17972	13,48
Fan1	FANCD2/FANCI-associated nuclease 1	ENSMUSG00000033458	27338	13,48
4931428L18Rik	RIKEN cDNA 4931428L18 gene	ENSMUSG00000086727	81584	13,48
Hdac11	histone deacetylase 11	ENSMUSG00000034245	18028	13,41
Imp4	IMP4, U3 small nucleolar ribonucleoprotein, homolog (yeast)	ENSMUSG00000026127	5886	13,41
Supv3l1	suppressor of var1, 3-like 1 (S. cerevisiae)	ENSMUSG00000020079	20530	13,41
Sco2	SCO cytochrome oxidase deficient homolog 2 (yeast)	ENSMUSG00000091780	2180	13,41
Cetn3	centrin 3	ENSMUSG00000021537	13852	13,41
Mitd1	MIT, microtubule interacting and transport, domain containing 1	ENSMUSG00000026088	15611	13,41
Gdpd3	glycerophosphodiester phosphodiesterase domain containing 3	ENSMUSG00000030703	9230	13,41
Arhgap28	Rho GTPase activating protein 28	ENSMUSG00000024043	161408	13,41
Coa6	cytochrome c oxidase assembly factor 6	ENSMUSG00000051671	2934	13,41
Sep 06	septin 6	ENSMUSG00000050379	80469	13,41
Ccdc101	coiled-coil domain containing 101	ENSMUSG00000030714	23617	13,41
		ENSMUSG00000042997	14736	13,41
Irf3	interferon regulatory factor 3	ENSMUSG00000003184	5201	13,41
Helq	helicase, POLQ-like	ENSMUSG00000035266	36454	13,41
Apaf1	apoptotic peptidase activating factor 1	ENSMUSG00000019979	93460	13,41
Cnksr1	connector enhancer of kinase suppressor of Ras 1	ENSMUSG00000028841	10359	13,41
Gpr68	G protein-coupled receptor 68	ENSMUSG00000047415	31517	13,41
Abracl	ABRA C-terminal like	ENSMUSG00000078453	11990	13,34
Fam110a	family with sequence similarity 110, member A	ENSMUSG00000027459	10822	13,34
Syce2	synaptonemal complex central element protein 2	ENSMUSG00000003824	16111	13,34
Pomt1	protein-O-mannosyltransferase 1	ENSMUSG00000039254	18416	13,34
Grb7	growth factor receptor bound protein 7	ENSMUSG00000019312	8980	13,34

Gnpda2	glucosamine-6-phosphate deaminase 2	ENSMUSG00000029209	19230	13,34
Lgals8	lectin, galactose binding, soluble 8	ENSMUSG00000057554	25530	13,34
Cog2	component of oligomeric golgi complex 2	ENSMUSG00000031979	31242	13,34
Vav3	vav 3 oncogene	ENSMUSG00000033721	345046	13,34
Ahr	aryl-hydrocarbon receptor	ENSMUSG00000019256	37065	13,34
Slc30a1	solute carrier family 30 (zinc transporter), member 1	ENSMUSG00000037434	6481	13,34
Dnaic1	dynein, axonemal, intermediate chain 1	ENSMUSG00000061322	68384	13,34
Pam16	presequence translocase-asssociated motor 16 homolog (S. cerevisiae)	ENSMUSG00000014301	8525	13,34
Crkl	v-crk sarcoma virus CT10 oncogene homolog (avian)-like	ENSMUSG00000006134	34269	13,34
Tcirg1	T cell, immune regulator 1, ATPase, H+ transporting, lysosomal V0 protein A3	ENSMUSG00000001750	11084	13,34
Fam196a	family with sequence similarity 196, member A	ENSMUSG00000073805	56505	13,34
E130309D14Rik	RIKEN cDNA E130309D14 gene	ENSMUSG00000069814	21912	13,27
Runx2	runt related transcription factor 2	ENSMUSG00000039153	318811	13,27
Ung	uracil DNA glycosylase	ENSMUSG00000029591	8938	13,27
Doc2a	double C2, alpha	ENSMUSG00000052301	5290	13,27
Dyrk4	dual-specificity tyrosine-(Y)-phosphorylation regulated kinase 4	ENSMUSG00000030345	45820	13,27
U2af1l4	U2 small nuclear RNA auxiliary factor 1-like 4	ENSMUSG00000078765	4660	13,27
Nlrx1	NLR family member X1	ENSMUSG00000032109	15887	13,27
Git1	G protein-coupled receptor kinase-interactor 1	ENSMUSG00000011877	14225	13,27
Epdr1	ependymin related protein 1 (zebrafish)	ENSMUSG00000002808	28122	13,27
Ipo13	importin 13	ENSMUSG00000033365	20514	13,27
Pnpo	pyridoxine 5'-phosphate oxidase	ENSMUSG00000018659	6162	13,27
Tgfbrap1	transforming growth factor, beta receptor associated protein 1	ENSMUSG00000070939	51354	13,27
Gmip	Gem-interacting protein	ENSMUSG00000036246	13192	13,27
Slc16a13	solute carrier family 16 (monocarboxylic acid transporters), member 13	ENSMUSG00000044367	4206	13,27
Stard3	START domain containing 3	ENSMUSG00000018167	22745	13,27
Spag5	sperm associated antigen 5	ENSMUSG00000002055	20929	13,27
Gm4944	predicted gene 4944	ENSMUSG00000096433	28350	13,27
Pstk	phosphoseryl-tRNA kinase	ENSMUSG00000063179	16699	13,27
Ccdc158	coiled-coil domain containing 158	ENSMUSG00000050050	67318	13,27
Usp43	ubiquitin specific peptidase 43	ENSMUSG00000020905	67631	13,27
BC024978	cDNA sequence BC024978	ENSMUSG00000078786	9606	13,27
Col20a1	collagen, type XX, alpha 1	ENSMUSG00000016356	31006	13,27
Ccnd2	cyclin D2	ENSMUSG00000000184	25270	13,19
Zfp651	zinc finger protein 651	ENSMUSG00000013419	11710	13,19
Nkd2	naked cuticle 2 homolog (Drosophila)	ENSMUSG00000021567	29098	13,19
Mettl14	methyltransferase like 14	ENSMUSG00000028114	21346	13,19
Cln8	ceroid-lipofuscinosis, neuronal 8	ENSMUSG00000026317	20386	13,19

Maf1	MAF1 homolog (S. cerevisiae)	ENSMUSG00000022553	3087	13,19
Slc24a3	solute carrier family 24 (sodium/potassium/calcium exchanger), member 3	ENSMUSG00000063873	474413	13,19
Rbm45	RNA binding motif protein 45	ENSMUSG00000042369	13785	13,19
Tyw5	tRNA-yW synthesizing protein 5	ENSMUSG00000048495	18865	13,19
Scn2a1	sodium channel, voltage-gated, type II, alpha 1	ENSMUSG00000075318	146591	13,19
Ptpn23	protein tyrosine phosphatase, non-receptor type 23	ENSMUSG00000036057	23122	13,19
Pfkl	phosphofructokinase, liver, B-type	ENSMUSG00000020277	23137	13,19
Slc12a9	solute carrier family 12 (potassium/chloride transporters), member 9	ENSMUSG00000037344	19040	13,19
Foxp2	forkhead box P2	ENSMUSG00000029563	540629	13,19
		ENSMUSG00000092360	9504	13,19
Gin1	gypsy retrotransposon integrase 1	ENSMUSG00000026333	23538	13,19
Deptor	DEP domain containing MTOR-interacting protein	ENSMUSG00000022419	146955	13,19
Ripk2		ENSMUSG00000041135	40915	13,19
Adam5	a disintegrin and metallopeptidase domain 5	ENSMUSG00000031554	97277	13,19
		ENSMUSG00000046532	173495	13,19
Dgkb	diacylglycerol kinase, beta	ENSMUSG00000036095	752706	13,19
St8sia2	ST8 alpha-N-acetyl-neuraminide alpha-2,8-sialyltransferase 2	ENSMUSG00000025789	74564	13,19
Syne1	spectrin repeat containing, nuclear envelope 1	ENSMUSG00000096054	213744	13,19
Ccdc32	coiled-coil domain containing 32	ENSMUSG00000039983	11615	13,12
Enkd1	enkurin domain containing 1	ENSMUSG00000013155	4279	13,12
Kdelc2	KDEL (Lys-Asp-Glu-Leu) containing 2	ENSMUSG00000034487	17845	13,12
Foxm1	forkhead box M1	ENSMUSG00000001517	12927	13,12
Il18	interleukin 18	ENSMUSG00000039217	6565	13,12
Hmmr	hyaluronan mediated motility receptor (RHAMM)	ENSMUSG00000020330	32028	13,12
Zfp354c	zinc finger protein 354C	ENSMUSG00000044807	16639	13,12
Efnb1	ephrin B1	ENSMUSG00000031217	12862	13,12
Tceb1	transcription elongation factor B (SIII), polypeptide 1	ENSMUSG00000079658	14100	13,12
Cecr5	cat eye syndrome chromosome region, candidate 5	ENSMUSG00000058979	21826	13,12
Lrriq3	leucine-rich repeats and IQ motif containing 3	ENSMUSG00000028182	100845	13,12
Acbd4	acyl-Coenzyme A binding domain containing 4	ENSMUSG00000056938	10519	13,12
		ENSMUSG00000079733	25215	13,12
Aox3	aldehyde oxidase 3	ENSMUSG00000064294	87569	13,12
Zfp141	zinc finger protein 141	ENSMUSG00000092416	32341	13,12
Acbd7	acyl-Coenzyme A binding domain containing 7	ENSMUSG00000026644	4826	13,05
Sfxn2	sideroflexin 2	ENSMUSG00000025036	23534	13,05
		ENSMUSG00000025578	4665	13,05
Ap1m1	adaptor-related protein complex AP-1, mu subunit 1	ENSMUSG00000003033	17368	13,05
Hectd3	HECT domain containing 3	ENSMUSG00000046861	9961	13,05

Thyn1	thymocyte nuclear protein 1	ENSMUSG00000035443	7654	13,05
Emc4	ER membrane protein complex subunit 4	ENSMUSG00000027131	5017	13,05
Esd	esterase D/formylglutathione hydrolase	ENSMUSG00000021996	18469	13,05
Zfyve19	zinc finger, FYVE domain containing 19	ENSMUSG00000068580	8433	13,05
1110001A16Rik	RIKEN cDNA 1110001A16 gene	ENSMUSG00000062691	4539	13,05
Cpt1c	carnitine palmitoyltransferase 1c	ENSMUSG00000007783	15479	13,05
Nbn	nibrin	ENSMUSG00000028224	34665	13,05
Ncmap	noncompact myelin associated protein	ENSMUSG00000043924	28652	13,05
Glyatl3	glycine-N-acyltransferase-like 3	ENSMUSG00000091043	9610	12,98
Spryd3	SPRY domain containing 3	ENSMUSG00000036966	19707	12,98
Trat1	T cell receptor associated transmembrane adaptor 1	ENSMUSG00000030775	37267	12,98
		ENSMUSG00000025321	80781	12,98
Tnks1bp1	tankyrase 1 binding protein 1	ENSMUSG00000033955	25027	12,98
Drc1	dynein regulatory complex subunit 1	ENSMUSG00000073102	85321	12,98
Prex2	phosphatidylinositol-3,4,5-trisphosphate-dependent Rac exchange factor 2	ENSMUSG00000048960	310218	12,98
Mrps27	mitochondrial ribosomal protein S27	ENSMUSG00000041632	70776	12,98
Ciita	class II transactivator	ENSMUSG00000022504	39387	12,98
Smoc2	SPARC related modular calcium binding 2	ENSMUSG00000023886	125285	12,90
Tmem80	transmembrane protein 80	ENSMUSG00000025505	9418	12,90
		ENSMUSG00000029518	15808	12,90
Fam159a	family with sequence similarity 159, member A	ENSMUSG00000059816	15573	12,90
6330403A02Rik	RIKEN cDNA 6330403A02 gene	ENSMUSG00000053963	51118	12,90
Stx4a	syntaxin 4A (placental)	ENSMUSG00000030805	24726	12,90
Ltbp4	latent transforming growth factor beta binding protein 4	ENSMUSG00000040488	32557	12,90
Dcaf4	DDB1 and CUL4 associated factor 4	ENSMUSG00000021222	21523	12,90
Cbr4	carbonyl reductase 4	ENSMUSG00000031641	15749	12,90
Tmem184a	transmembrane protein 184a	ENSMUSG00000036687	17433	12,90
Trim9	tripartite motif-containing 9	ENSMUSG00000021071	103076	12,90
Syce1l	synaptonemal complex central element protein 1 like	ENSMUSG00000033409	12321	12,90
Ctps	cytidine 5'-triphosphate synthase	ENSMUSG00000028633	30409	12,90
Slc5a3	solute carrier family 5 (inositol transporters), member 3	ENSMUSG00000089774	29152	12,90
Birc3	baculoviral IAP repeat-containing 3	ENSMUSG00000032000	24488	12,90
Arvcf	armadillo repeat gene deleted in velo-cardio-facial syndrome	ENSMUSG00000000325	58895	12,83
Trim62	tripartite motif-containing 62	ENSMUSG00000041000	27749	12,83
		ENSMUSG00000090544	822	12,83
Rpf2	ribosome production factor 2 homolog (S. cerevisiae)	ENSMUSG00000038510	23791	12,83
Arhgap29	Rho GTPase activating protein 29	ENSMUSG00000039831	63449	12,83
4931428F04Rik	RIKEN cDNA 4931428F04 gene	ENSMUSG00000014837	9119	12,83
Sirt2	sirtuin 2	ENSMUSG00000015149	21931	12,83

Fahd1	fumarylacetoacetate hydrolase domain containing 1	ENSMUSG00000045316	1407	12,83
Trmt61a	tRNA methyltransferase 61A	ENSMUSG00000060950	5798	12,83
Mrpl38	mitochondrial ribosomal protein L38	ENSMUSG00000020775	7052	12,83
Traf7	TNF receptor-associated factor 7	ENSMUSG00000052752	19377	12,83
Pigg	phosphatidylinositol glycan anchor biosynthesis, class G	ENSMUSG00000029263	36747	12,83
Mios	missing oocyte, meiosis regulator, homolog (Drosophila)	ENSMUSG00000042447	27053	12,83
Cyb5d2	cytochrome b5 domain containing 2	ENSMUSG00000057778	18911	12,83
Gca	grancalcin	ENSMUSG00000026893	29825	12,83
Sacs	sacsin	ENSMUSG00000048279	102239	12,83
Cnih2	cornichon homolog 2 (Drosophila)	ENSMUSG00000024873	5548	12,76
Plekhj1	pleckstrin homology domain containing, family J member 1	ENSMUSG00000035278	2527	12,76
Gltp	glycolipid transfer protein	ENSMUSG00000011884	21582	12,76
		ENSMUSG00000005267	31550	12,76
Sgcb	sarcoglycan, beta (dystrophin-associated glycoprotein)	ENSMUSG00000029156	14965	12,76
Sart1	squamous cell carcinoma antigen recognized by T cells 1	ENSMUSG00000039148	11181	12,76
Fcgrt	Fc receptor, IgG, alpha chain transporter	ENSMUSG00000003420	10830	12,76
Kcnc1	potassium voltage gated channel, Shaw-related subfamily, member 1	ENSMUSG00000058975	41879	12,76
Acyp1	acylphosphatase 1, erythrocyte (common) type	ENSMUSG00000008822	16041	12,76
Lrig3	leucine-rich repeats and immunoglobulin-like domains 3	ENSMUSG00000020105	49141	12,76
Etfdh	electron transferring flavoprotein, dehydrogenase	ENSMUSG00000027809	25076	12,76
Nqo2	NAD(P)H dehydrogenase, quinone 2	ENSMUSG00000046949	23807	12,76
Stac	src homology three (SH3) and cysteine rich domain	ENSMUSG00000032502	128912	12,76
Prelid2	PRELI domain containing 2	ENSMUSG00000056671	75499	12,76
		ENSMUSG00000095719	123596	12,76
Znrd1	zinc ribbon domain containing, 1	ENSMUSG00000036315	4202	12,69
Kcnn1	potassium intermediate/small conductance calcium-activated channel, subfamily N, member 1	ENSMUSG00000002908	14960	12,69
Dolpp1	dolichyl pyrophosphate phosphatase 1	ENSMUSG00000026856	8276	12,69
Slc41a1	solute carrier family 41, member 1	ENSMUSG00000013275	21373	12,69
Slc35e4	solute carrier family 35, member E4	ENSMUSG00000048807	7644	12,69
Ifngr1	interferon gamma receptor 1	ENSMUSG00000020009	18281	12,69
Wfs1	Wolfram syndrome 1 homolog (human)	ENSMUSG00000039474	22879	12,69
Adnp2	ADNP homeobox 2	ENSMUSG00000053950	25145	12,69
Dyrk2	dual-specificity tyrosine-(Y)-phosphorylation regulated kinase 2	ENSMUSG00000028630	9555	12,69
4931414P19Rik	RIKEN cDNA 4931414P19 gene	ENSMUSG00000022179	22246	12,69
Lnx2	ligand of numb-protein X 2	ENSMUSG00000016520	59932	12,69

		ENSMUSG00000041261	95675	12,69
Ankrd39	ankyrin repeat domain 39	ENSMUSG00000079610	9029	12,61
Nol3	nucleolar protein 3 (apoptosis repressor with CARD domain)	ENSMUSG00000014776	5493	12,61
Arhgap25	Rho GTPase activating protein 25	ENSMUSG00000030047	74715	12,61
Pdia5	protein disulfide isomerase associated 5	ENSMUSG00000022844	93561	12,61
Cnep1r1	CTD nuclear envelope phosphatase 1 regulatory subunit 1	ENSMUSG00000036810	16342	12,61
		ENSMUSG00000018334	132968	12,61
Nap1l3	nucleosome assembly protein 1-like 3	ENSMUSG00000055733	2821	12,61
Bfsp2	beaded filament structural protein 2, phakinin	ENSMUSG00000032556	55409	12,61
Ncapg	non-SMC condensin I complex, subunit G	ENSMUSG00000015880	30623	12,61
Fam154b	family with sequence similarity 154, member B	ENSMUSG00000038570	15569	12,61
Tdp1	tyrosyl-DNA phosphodiesterase 1	ENSMUSG00000021177	70703	12,61
Zdhhc11	zinc finger, DHHC domain containing 11	ENSMUSG00000069189	29139	12,61
Cacna1a	calcium channel, voltage-dependent, P/Q type, alpha 1A subunit	ENSMUSG00000034656	251807	12,61
Atl1	atlastin GTPase 1	ENSMUSG00000021066	70981	12,61
Alpk1	alpha-kinase 1	ENSMUSG00000028028	110129	12,61
		ENSMUSG00000078306	3758	12,61
Dpp10	dipeptidylpeptidase 10	ENSMUSG00000036815	713418	12,61
Sorcs3	sortilin-related VPS10 domain containing receptor 3	ENSMUSG00000063434	599481	12,61
Lrrc7	leucine rich repeat containing 7	ENSMUSG00000028176	477783	12,61
Lgr6	leucine-rich repeat-containing G protein-coupled receptor 6	ENSMUSG00000042793	118929	12,61
Arhgap19	Rho GTPase activating protein 19	ENSMUSG00000025154	35497	12,54
		ENSMUSG00000071035	1968	12,54
		ENSMUSG00000078139	1924	12,54
Mavs	mitochondrial antiviral signaling protein	ENSMUSG00000037523	13963	12,54
Rab39b	RAB39B, member RAS oncogene family	ENSMUSG00000031202	6186	12,54
Sep 10	septin 10	ENSMUSG00000019917	57735	12,54
Ghdc	GH3 domain containing	ENSMUSG00000017747	4926	12,54
Rasgef1b	RasGEF domain family, member 1B	ENSMUSG00000029333	35502	12,54
		ENSMUSG00000006215	23277	12,54
Csrnp3	cysteine-serine-rich nuclear protein 3	ENSMUSG00000044647	185780	12,54
Irf9	interferon regulatory factor 9	ENSMUSG00000002325	6460	12,54
Dgkh	diacylglycerol kinase, eta	ENSMUSG00000034731	155481	12,54
N4bp2l1	NEDD4 binding protein 2-like 1	ENSMUSG00000041132	22883	12,54
Cyp4f13	cytochrome P450, family 4, subfamily f, polypeptide 13	ENSMUSG00000024055	22715	12,54
Gpr4	G protein-coupled receptor 4	ENSMUSG00000044317	11639	12,54
		ENSMUSG00000090491	62761	12,54
Txndc5	thioredoxin domain containing 5	ENSMUSG00000038991	28552	12,47
Gm4737	predicted gene 4737	ENSMUSG00000048087	2081	12,47

Zcchc3	zinc finger, CCHC domain containing 3	ENSMUSG00000074682	3089	12,47
		ENSMUSG00000030888	5170	12,47
Mrpl53	mitochondrial ribosomal protein L53	ENSMUSG00000030037	845	12,47
Tti2	TELO2 interacting protein 2	ENSMUSG00000031577	14387	12,47
Alkbh4	alkB, alkylation repair homolog 4 (E. coli)	ENSMUSG00000039754	5470	12,47
Med31	mediator of RNA polymerase II transcription, subunit 31 homolog (yeast)	ENSMUSG00000020801	3869	12,47
Hsd17b10	hydroxysteroid (17-beta) dehydrogenase 10	ENSMUSG00000025260	2598	12,47
Gstm7	glutathione S-transferase, mu 7	ENSMUSG00000004035	5484	12,47
Tmem176a	transmembrane protein 176A	ENSMUSG00000023367	3882	12,47
Rcan2	regulator of calcineurin 2	ENSMUSG00000039601	237666	12,47
Ccdc8	coiled-coil domain containing 8	ENSMUSG00000041117	4809	12,47
Gm6878	predicted gene 6878	ENSMUSG00000075549	9824	12,47
		ENSMUSG00000092592	100040	12,47
Dennd3	DENN/MADD domain containing 3	ENSMUSG00000036661	59683	12,47
Lyrm1	LYR motif containing 1	ENSMUSG00000030922	20896	12,47
Rel	reticuloendotheliosis oncogene	ENSMUSG00000020275	29457	12,47
Rqcd1	rcd1 (required for cell differentiation) homolog 1 (S. pombe)	ENSMUSG00000026174	24787	12,40
Nt5m	5',3'-nucleotidase, mitochondrial	ENSMUSG00000032615	41522	12,40
Hspa13	heat shock protein 70 family, member 13	ENSMUSG00000032932	11632	12,40
Nr1i3	nuclear receptor subfamily 1, group I, member 3	ENSMUSG00000005677	6732	12,40
Eif2s1	eukaryotic translation initiation factor 2, subunit 1 alpha	ENSMUSG00000021116	25192	12,40
Ebpl	emopamil binding protein-like	ENSMUSG00000021928	20683	12,40
Gm2a	GM2 ganglioside activator protein	ENSMUSG00000000594	14915	12,40
Slc25a16	solute carrier family 25 (mitochondrial carrier, Graves disease autoantigen), member 16	ENSMUSG00000071253	25866	12,40
Uba3	ubiquitin-like modifier activating enzyme 3	ENSMUSG00000030061	21973	12,40
Gtf3c3	general transcription factor IIIC, polypeptide 3	ENSMUSG00000041303	41391	12,40
Lrrc56	leucine rich repeat containing 56	ENSMUSG00000038637	15899	12,40
Slc25a40	solute carrier family 25, member 40	ENSMUSG00000054099	31941	12,40
Pin1	protein (peptidyl-prolyl cis/trans isomerase) NIMA-interacting 1	ENSMUSG00000032171	14455	12,40
Prkab1	protein kinase, AMP-activated, beta 1 non-catalytic subunit	ENSMUSG00000029513	10923	12,40
Unc93b1	unc-93 homolog B1 (C. elegans)	ENSMUSG00000036908	14155	12,40
		ENSMUSG00000002578	15149	12,40
Kit	kit oncogene	ENSMUSG00000005672	81807	12,40
Arg2	arginase type II	ENSMUSG00000021125	25525	12,40
Cd46	CD46 antigen, complement regulatory protein	ENSMUSG00000016493	50964	12,40
Rsph10b	radial spoke head 10 homolog B (Chlamydomonas)	ENSMUSG00000075569	52685	12,40

Eef1a2	eukaryotic translation elongation factor 1 alpha 2	ENSMUSG00000016349	9362	12,32
Rpl23a	ribosomal protein L23A	ENSMUSG00000058546	2653	12,32
Ifitm2	interferon induced transmembrane protein 2	ENSMUSG00000060591	1123	12,32
Tipin	timeless interacting protein	ENSMUSG00000032397	23186	12,32
Psmg1	proteasome (prosome, macropain) assembly chaperone 1	ENSMUSG00000022913	11028	12,32
Veph1	ventricular zone expressed PH domain-containing 1	ENSMUSG00000027831	243280	12,32
Mphosph10	M-phase phosphoprotein 10 (U3 small nucleolar ribonucleoprotein)	ENSMUSG00000030521	15693	12,32
Chd1l	chromodomain helicase DNA binding protein 1-like	ENSMUSG00000028089	49462	12,32
Mzf1	myeloid zinc finger 1	ENSMUSG00000030380	12462	12,32
		ENSMUSG00000022537	38988	12,32
		ENSMUSG00000028701	18933	12,32
Brms1l	breast cancer metastasis-suppressor 1-like	ENSMUSG00000012076	33370	12,32
Zfp169	zinc finger protein 169	ENSMUSG00000050954	25805	12,32
Slc13a2	solute carrier family 13 (sodium-dependent dicarboxylate transporter), member 2	ENSMUSG00000001095	25131	12,32
Fam216b	family with sequence similarity 216, member B	ENSMUSG00000045655	7987	12,32
		ENSMUSG00000074529	22274	12,32
Ugt2b38	UDP glucuronosyltransferase 2 family, polypeptide B38	ENSMUSG00000061906	14262	12,32
Arsa	arylsulfatase A	ENSMUSG00000022620	4950	12,25
Mbl2	mannose-binding lectin (protein C) 2	ENSMUSG00000024863	6758	12,25
Trpv2	transient receptor potential cation channel, subfamily V, member 2	ENSMUSG00000018507	26030	12,25
		ENSMUSG00000029173	29643	12,25
Tmem43	transmembrane protein 43	ENSMUSG00000030095	14757	12,25
4930558K02Rik	RIKEN cDNA 4930558K02 gene	ENSMUSG00000086277	37551	12,25
Yipf3	Yip1 domain family, member 3	ENSMUSG00000071074	4458	12,25
Chtf8	CTF8, chromosome transmission fidelity factor 8	ENSMUSG00000046691	9739	12,25
Mib2	mindbomb homolog 2 (Drosophila)	ENSMUSG00000029060	14522	12,25
AA987161	expressed sequence AA987161	ENSMUSG00000058093	20265	12,25
Supt20	suppressor of Ty 20	ENSMUSG00000027751	23733	12,25
Polh	polymerase (DNA directed), eta (RAD 30 related)	ENSMUSG00000023953	30622	12,25
		ENSMUSG00000041565	504165	12,25
		ENSMUSG00000020491	16073	12,25
Slc7a3	solute carrier family 7 (cationic amino acid transporter, y+ system), member 3	ENSMUSG00000031297	6811	12,25
		ENSMUSG00000003123	19184	12,25
Uggt2	UDP-glucose glycoprotein glucosyltransferase 2	ENSMUSG00000042104	114392	12,25
Cd83	CD83 antigen	ENSMUSG00000015396	18024	12,25
Dach2	dachshund 2 (Drosophila)	ENSMUSG00000025592	538877	12,25
Ldhd	lactate dehydrogenase D	ENSMUSG00000031958	6590	12,18

Slc1a2	solute carrier family 1 (glial high affinity glutamate transporter), member 2	ENSMUSG00000005089	132126	12,18
Maneal	mannosidase, endo-alpha-like	ENSMUSG00000042763	6933	12,18
Trp53rk	transformation related protein 53 regulating kinase	ENSMUSG00000042854	6975	12,18
Bcat2	branched chain aminotransferase 2, mitochondrial	ENSMUSG00000030826	19558	12,18
Dapk3	death-associated protein kinase 3	ENSMUSG00000034974	10191	12,18
Rbbp9	retinoblastoma binding protein 9	ENSMUSG00000027428	8605	12,18
Bcl2l12	BCL2-like 12 (proline rich)	ENSMUSG00000003190	7491	12,18
Dhps	deoxyhypusine synthase	ENSMUSG00000060038	3406	12,18
Cdh23	cadherin 23 (otocadherin)	ENSMUSG00000012819	393743	12,18
Adam15	a disintegrin and metallopeptidase domain 15 (metargidin)	ENSMUSG00000028041	10357	12,18
A4galt	alpha 1,4-galactosyltransferase	ENSMUSG00000047878	25049	12,18
Cldn6	claudin 6	ENSMUSG00000023906	3082	12,18
Ccdc108	coiled-coil domain containing 108	ENSMUSG00000047021	33529	12,18
Usp2	ubiquitin specific peptidase 2	ENSMUSG00000032010	28607	12,18
BC027231	cDNA sequence BC027231	ENSMUSG00000036208	12984	12,18
Ephb4	Eph receptor B4	ENSMUSG00000029710	28561	12,18
Nphp4	nephronophthisis 4 (juvenile) homolog (human)	ENSMUSG00000039577	86478	12,18
Glmn	glomulin, FKBP associated protein	ENSMUSG00000029276	48922	12,18
Gabra3	gamma-aminobutyric acid (GABA) A receptor, subunit alpha 3	ENSMUSG00000031343	224168	12,18
Fbn2	fibrillin 2	ENSMUSG00000024598	201304	12,11
Scamp5	secretory carrier membrane protein 5	ENSMUSG00000040722	26697	12,11
Bbs1	Bardet-Biedl syndrome 1 (human)	ENSMUSG00000006464	19730	12,11
Cenpl	centromere protein L	ENSMUSG00000026708	15958	12,11
Sharpin	SHANK-associated RH domain interacting protein	ENSMUSG00000022552	4072	12,11
Pomgnt2	protein O-linked mannose beta 1,4-N-acetylglucosaminyltransferase 2	ENSMUSG00000066235	14421	12,11
Tspan31	tetraspanin 31	ENSMUSG00000006736	2945	12,11
Scd3	stearoyl-coenzyme A desaturase 3	ENSMUSG00000025202	40729	12,11
Bora	bora, aurora kinase A activator	ENSMUSG00000022070	28111	12,11
Cdc23	CDC23 cell division cycle 23	ENSMUSG00000024370	20785	12,11
9430038I01Rik	RIKEN cDNA 9430038I01 gene	ENSMUSG00000040139	35189	12,11
Urb1	URB1 ribosome biogenesis 1 homolog (S. cerevisiae)	ENSMUSG00000039929	58887	12,11
Enpp4	ectonucleotide pyrophosphatase/phosphodiesterase 4	ENSMUSG00000023961	9502	12,11
Arhgef33	Rho guanine nucleotide exchange factor (GEF) 33	ENSMUSG00000054901	81282	12,11
Cnnm4	cyclin M4	ENSMUSG00000037408	37145	12,11
Exd2	exonuclease 3'-5' domain containing 2	ENSMUSG00000032705	35041	12,11
Mycbpap	MYCBP associated protein	ENSMUSG00000039110	20396	12,11
2410076I21Rik	RIKEN cDNA 2410076I21 gene	ENSMUSG00000074269	88704	12,11
Pla2g12a	phospholipase A2, group XIIA	ENSMUSG00000027999	17200	12,03
Aurka	aurora kinase A	ENSMUSG00000027496	14346	12,03
Nop9	NOP9 nucleolar protein	ENSMUSG00000019297	9808	12,03

Rcl1	RNA terminal phosphate cyclase-like 1	ENSMUSG00000024785	42469	12,03
Stk17b	serine/threonine kinase 17b (apoptosis-inducing)	ENSMUSG00000026094	29719	12,03
Exog	endo/exonuclease (5'-3'), endonuclease G-like	ENSMUSG00000042787	20595	12,03
Bak1	BCL2-antagonist/killer 1	ENSMUSG00000057789	9200	12,03
Slc30a6	solute carrier family 30 (zinc transporter), member 6	ENSMUSG00000024069	28622	12,03
Utp18	UTP18, small subunit (SSU) processome component, homolog (yeast)	ENSMUSG00000054079	26524	12,03
Fam45a	family with sequence similarity 45, member A	ENSMUSG00000024993	24643	12,03
Retsat	retinol saturase (all trans retinol 13,14 reductase)	ENSMUSG00000056666	9951	12,03
Fgf20	fibroblast growth factor 20	ENSMUSG00000031603	29166	12,03
		ENSMUSG00000045435	4680	12,03
Dennd1c	DENN/MADD domain containing 1C	ENSMUSG00000002668	12455	12,03
Dtwd2	DTW domain containing 2	ENSMUSG00000024505	59457	12,03
Eml3	echinoderm microtubule associated protein like 3	ENSMUSG00000071647	11870	12,03
		ENSMUSG00000089901	8022	12,03
		ENSMUSG00000055493	116241	12,03
Bid	BH3 interacting domain death agonist	ENSMUSG00000004446	24924	12,03
		ENSMUSG00000074527	35301	12,03
Man2b1	mannosidase 2, alpha B1	ENSMUSG00000005142	15471	12,03
Sdcbp2	syndecan binding protein (syntenin) 2	ENSMUSG00000027456	17384	12,03
Qars	glutaminyl-tRNA synthetase	ENSMUSG00000032604	7937	11,96
Tamm41	TAM41, mitochondrial translocator assembly and maintenance protein, homolog (S. cerevisiae)	ENSMUSG00000030316	33496	11,96
Pard6a	par-6 (partitioning defective 6,) homolog alpha (C. elegans)	ENSMUSG00000005699	2340	11,96
Abcb9	ATP-binding cassette, sub-family B (MDR/TAP), member 9	ENSMUSG00000029408	34269	11,96
Cyb561d1	cytochrome b-561 domain containing 1	ENSMUSG00000048796	5526	11,96
Slc7a7	solute carrier family 7 (cationic amino acid transporter, y+ system), member 7	ENSMUSG00000000958	40029	11,96
Zfp770	zinc finger protein 770	ENSMUSG00000040321	8009	11,96
Fbxo22	F-box protein 22	ENSMUSG00000032309	15509	11,96
Fpgs	folylpolyglutamyl synthetase	ENSMUSG00000009566	21529	11,96
Slc39a14	solute carrier family 39 (zinc transporter), member 14	ENSMUSG00000022094	47957	11,96
Cxcl17	chemokine (C-X-C motif) ligand 17	ENSMUSG00000060188	12834	11,96
Dnah8	dynein, axonemal, heavy chain 8	ENSMUSG00000033826	250911	11,96
Rfx5	regulatory factor X, 5 (influences HLA class II expression)	ENSMUSG00000005774	7487	11,96
		ENSMUSG00000031016	19763	11,96
Piezo1	piezo-type mechanosensitive ion channel component 1	ENSMUSG00000014444	69632	11,96

Lrrc69	leucine rich repeat containing 69	ENSMUSG00000023151	172441	11,96
Il20rb	interleukin 20 receptor beta	ENSMUSG00000044244	29070	11,96
Ascl3	achaete-scute complex homolog 3 (Drosophila)	ENSMUSG00000035951	4268	11,89
Myo1c	myosin IC	ENSMUSG00000017774	23407	11,89
Tmub1	transmembrane and ubiquitin-like domain containing 1	ENSMUSG00000028958	2397	11,89
Smpd3	sphingomyelin phosphodiesterase 3, neutral	ENSMUSG00000031906	85441	11,89
G6pc3	glucose 6 phosphatase, catalytic, 3	ENSMUSG00000034793	4462	11,89
Dcaf15	DDB1 and CUL4 associated factor 15	ENSMUSG00000037103	7699	11,89
Hn1l	hematological and neurological expressed 1-like	ENSMUSG00000024165	18481	11,89
Slc25a29	solute carrier family 25 (mitochondrial carrier, palmitoylcarnitine transporter), member 29	ENSMUSG00000021265	10011	11,89
Gcdh	glutaryl-Coenzyme A dehydrogenase	ENSMUSG00000003809	7529	11,89
Tpst1	protein-tyrosine sulfotransferase 1	ENSMUSG00000034118	62404	11,89
Psmd5	proteasome (prosome, macropain) 26S subunit, non-ATPase, 5	ENSMUSG00000026869	18852	11,89
Ddx28	DEAD (Asp-Glu-Ala-Asp) box polypeptide 28	ENSMUSG00000045538	1871	11,89
Rbm15	RNA binding motif protein 15	ENSMUSG00000048109	7180	11,89
Siae	sialic acid acetylesterase	ENSMUSG00000001942	34472	11,89
Ercc8	excision repaiross-complementing rodent repair deficiency, complementation group 8	ENSMUSG00000021694	36634	11,89
Slc15a3	solute carrier family 15, member 3	ENSMUSG00000024737	19636	11,89
Zkscan5	zinc finger with KRAB and SCAN domains 5	ENSMUSG00000055991	17189	11,89
Mettl18	methyltransferase like 18	ENSMUSG00000041396	2355	11,89
Nup133	nucleoporin 133	ENSMUSG00000039509	52143	11,89
Klhl25	kelch-like 25	ENSMUSG00000055652	25793	11,89
Mettl3	methyltransferase like 3	ENSMUSG00000022160	10461	11,89
Pank4	pantothenate kinase 4	ENSMUSG00000029056	16816	11,89
Cd151	CD151 antigen	ENSMUSG00000025510	4118	11,89
Abhd3	abhydrolase domain containing 3	ENSMUSG00000002475	62361	11,89
Ccno	cyclin O	ENSMUSG00000042417	2977	11,89
Xxylt1	xyloside xylosyltransferase 1	ENSMUSG00000047434	125806	11,89
Top1mt	DNA topoisomerase 1, mitochondrial	ENSMUSG00000000934	21766	11,89
Zfp868	zinc finger protein 868	ENSMUSG00000060427	14692	11,89
Tmem120b	transmembrane protein 120B	ENSMUSG00000054434	49335	11,89
Grik2	glutamate receptor, ionotropic, kainate 2 (beta 2)	ENSMUSG00000056073	689307	11,89
Apoo	apolipoprotein O	ENSMUSG00000079508	49977	11,89
Chrna9	cholinergic receptor, nicotinic, alpha polypeptide 9	ENSMUSG00000029205	42406	11,89
Snx22	sorting nexin 22	ENSMUSG00000039452	4556	11,82
Ndufa1	NADH dehydrogenase (ubiquinone) 1 alpha subcomplex, 1	ENSMUSG00000016427	3576	11,82
		ENSMUSG00000039838	23603	11,82

Mad2l1bp	MAD2L1 binding protein	ENSMUSG00000034509	6164	11,82
Calcoco1	calcium binding and coiled coil domain 1	ENSMUSG00000023055	15400	11,82
Wipf1	WAS/WASL interacting protein family, member 1	ENSMUSG00000075284	100125	11,82
Ttc33	tetratricopeptide repeat domain 33	ENSMUSG00000022151	32812	11,82
Kctd11	potassium channel tetramerisation domain containing 11	ENSMUSG00000046731	2721	11,82
Hsd3b7	hydroxy-delta-5-steroid dehydrogenase, 3 beta- and steroid delta-isomerase 7	ENSMUSG00000042289	18195	11,82
Zbtb42	zinc finger and BTB domain containing 42	ENSMUSG00000037638	3920	11,82
Appl2	adaptor protein, phosphotyrosine interaction, PH domain and leucine zipper containing 2	ENSMUSG00000020263	48706	11,82
Fam98c	family with sequence similarity 98, member C	ENSMUSG00000030590	3724	11,74
Ino80b	INO80 complex subunit B	ENSMUSG00000030034	3667	11,74
Rusc2	RUN and SH3 domain containing 2	ENSMUSG00000035969	45110	11,74
Tmem177	transmembrane protein 177	ENSMUSG00000036975	5319	11,74
		ENSMUSG00000030545	7175	11,74
Ubiad1	UbiA prenyltransferase domain containing 1	ENSMUSG00000047719	10277	11,74
4930481A15Rik	RIKEN cDNA 4930481A15 gene	ENSMUSG00000086938	16108	11,74
Fam174b	family with sequence similarity 174, member B	ENSMUSG00000078670	36613	11,74
Ptpre	protein tyrosine phosphatase, receptor type, E	ENSMUSG00000041836	148471	11,74
Lrrc24	leucine rich repeat containing 24	ENSMUSG00000033707	6898	11,74
Asl	argininosuccinate lyase	ENSMUSG00000025533	13875	11,74
Zfp395	zinc finger protein 395	ENSMUSG00000034522	40255	11,74
Inpp4b	inositol polyphosphate-4-phosphatase, type II	ENSMUSG00000037940	782481	11,74
1300017J02Rik	RIKEN cDNA 1300017J02 gene	ENSMUSG00000033688	54561	11,74
		ENSMUSG00000001506	16819	11,67
Tmem101	transmembrane protein 101	ENSMUSG00000020921	3859	11,67
Enho	energy homeostasis associated	ENSMUSG00000028445	2181	11,67
Sema6c	sema domain, transmembrane domain (TM), and cytoplasmic domain, (semaphorin) 6C	ENSMUSG00000038777	13568	11,67
Bloc1s4	biogenesis of organelles complex-1, subunit 4, cappuccino	ENSMUSG00000060708	1306	11,67
		ENSMUSG00000030256	7316	11,67
Ccng1	cyclin G1	ENSMUSG00000020326	6760	11,67
Aars2	alanyl-tRNA synthetase 2, mitochondrial (putative)	ENSMUSG00000023938	14002	11,67
Rhog	ras homolog gene family, member G	ENSMUSG00000073982	11001	11,67
Apold1	apolipoprotein L domain containing 1	ENSMUSG00000090698	4836	11,67
Pfkm	phosphofructokinase, muscle	ENSMUSG00000033065	39859	11,67
		ENSMUSG00000031290	83707	11,67
Zfp101	zinc finger protein 101	ENSMUSG00000055240	34135	11,67
Mthfs	5, 10-methenyltetrahydrofolate synthetase	ENSMUSG00000066442	40848	11,67

Sema7a	sema domain, immunoglobulin domain (Ig), and GPI membrane anchor, (semaphorin) 7A	ENSMUSG00000038264	22752	11,67
Pdzd7	PDZ domain containing 7	ENSMUSG00000074818	19709	11,67
Casp8	caspase 8	ENSMUSG00000026029	52130	11,67
Chkb	choline kinase beta	ENSMUSG00000022617	13504	11,67
Cntnap5a	contactin associated protein-like 5A	ENSMUSG00000070695	897271	11,67
Dnaic2	dynein, axonemal, intermediate chain 2	ENSMUSG00000034706	30482	11,67
Csrnp1	cysteine-serine-rich nuclear protein 1	ENSMUSG00000032515	6085	11,67
Dmap1	DNA methyltransferase 1-associated protein 1	ENSMUSG00000009640	7593	11,60
Rab9	RAB9, member RAS oncogene family	ENSMUSG00000079316	22615	11,60
Samd11	sterile alpha motif domain containing 11	ENSMUSG00000096351	8366	11,60
Ccdc13	coiled-coil domain containing 13	ENSMUSG00000079235	41835	11,60
Glipr1	GLI pathogenesis-related 1 (glioma)	ENSMUSG00000056888	17184	11,60
Rrp36	ribosomal RNA processing 36 homolog (S. cerevisiae)	ENSMUSG00000023971	6798	11,60
		ENSMUSG00000033961	6493	11,60
		ENSMUSG00000020534	23615	11,60
		ENSMUSG00000093898	290299	11,60
Cpne2	copine II	ENSMUSG00000034361	37540	11,60
Tfpt	TCF3 (E2A) fusion partner	ENSMUSG00000006335	9606	11,60
Naprt1	nicotinate phosphoribosyltransferase domain containing 1	ENSMUSG00000022574	3518	11,60
Cirh1a	cirrhosis, autosomal recessive 1A (human)	ENSMUSG00000041438	29454	11,60
Dnah1	dynein, axonemal, heavy chain 1	ENSMUSG00000019027	63522	11,60
Gm13157	predicted gene 13157	ENSMUSG00000078495	55815	11,60
Serpinb7	serine (or cysteine) peptidase inhibitor, clade B, member 7	ENSMUSG00000067001	53035	11,60
AF529169	cDNA sequence AF529169	ENSMUSG00000039313	32949	11,60
Dhrs11	dehydrogenase/reductase (SDR family) member 11	ENSMUSG00000034449	8139	11,60
		ENSMUSG00000047854	9671	11,60
Ndrg2	N-myc downstream regulated gene 2	ENSMUSG00000004558	8218	11,53
Sh3bgrl3	SH3 domain binding glutamic acid-rich protein-like 3	ENSMUSG00000028843	1384	11,53
Nubp1	nucleotide binding protein 1	ENSMUSG00000022503	12481	11,53
Kif22	kinesin family member 22	ENSMUSG00000030677	14689	11,53
Fbxl15	F-box and leucine-rich repeat protein 15	ENSMUSG00000025226	2263	11,53
Zfp956	zinc finger protein 956	ENSMUSG00000045466	11911	11,53
1600002K03Rik	RIKEN cDNA 1600002K03 gene	ENSMUSG00000035595	2203	11,53
Zmynd10	zinc finger, MYND domain containing 10	ENSMUSG00000010044	4010	11,53
Micall1	microtubule associated monooxygenase, calponin and LIM domain containing -like 1	ENSMUSG00000033039	27926	11,53

Fam78a	family with sequence similarity 78, member A	ENSMUSG00000050592	34803	11,53
Tmem132a	transmembrane protein 132A	ENSMUSG00000024736	12119	11,53
Cyp2a4	cytochrome P450, family 2, subfamily a, polypeptide 4	ENSMUSG00000074254	7899	11,53
Lhfpl2	lipoma HMGIC fusion partner-like 2	ENSMUSG00000045312	137614	11,53
Zfp341	zinc finger protein 341	ENSMUSG00000059842	33525	11,53
Adpgk	ADP-dependent glucokinase	ENSMUSG00000025236	24628	11,53
Scml4	sex comb on midleg-like 4 (Drosophila)	ENSMUSG00000044770	100411	11,53
Ninj2	ninjurin 2	ENSMUSG00000041377	106990	11,53
Pms1	postmeiotic segregation increased 1 (S. cerevisiae)	ENSMUSG00000026098	107832	11,53
Nek4	NIMA (never in mitosis gene a)-related expressed kinase 4	ENSMUSG00000021918	35954	11,53
Syne1	spectrin repeat containing, nuclear envelope 1	ENSMUSG00000019769	48861	11,53
Ndst3	N-deacetylase/N-sulfotransferase (heparan glucosaminyl) 3	ENSMUSG00000027977	164687	11,53
Sntn	sentan, cilia apical structure protein	ENSMUSG00000044772	12273	11,53
		ENSMUSG00000050490	952	11,45
Umps	uridine monophosphate synthetase	ENSMUSG00000022814	12257	11,45
		ENSMUSG00000021974	41791	11,45
Efna4	ephrin A4	ENSMUSG00000028040	4639	11,45
Bloc1s2	biogenesis of lysosome-related organelles complex-1, subunit 2	ENSMUSG00000057506	7181	11,45
Prr24	proline rich 24	ENSMUSG00000091811	1679	11,45
Mrpl39	mitochondrial ribosomal protein L39	ENSMUSG00000022889	18167	11,45
A230083G16Rik	RIKEN cDNA A230083G16 gene	ENSMUSG00000055818	8982	11,45
Eps8l3	EPS8-like 3	ENSMUSG00000040600	15670	11,45
Znhit6	zinc finger, HIT type 6	ENSMUSG00000074182	29038	11,45
Six2	sine oculis-related homeobox 2	ENSMUSG00000024134	4007	11,45
		ENSMUSG00000094886	240	11,45
Pml	promyelocytic leukemia	ENSMUSG00000036986	31711	11,45
Lamb3	laminin, beta 3	ENSMUSG00000026639	41922	11,45
		ENSMUSG00000032579	10659	11,45
Gtf3c5	general transcription factor IIIC, polypeptide 5	ENSMUSG00000026816	17441	11,45
Pak6	p21 protein (Cdc42/Rac)-activated kinase 6	ENSMUSG00000074923	34718	11,45
Alox12	arachidonate 12-lipoxygenase	ENSMUSG00000000320	13897	11,45
Ttc16	tetratricopeptide repeat domain 16	ENSMUSG00000039021	18608	11,45
Nek5	NIMA (never in mitosis gene a)-related expressed kinase 5	ENSMUSG00000037738	51438	11,45
Unc80	unc-80 homolog (C. elegans)	ENSMUSG00000055567	231500	11,45
Gm7694	predicted gene 7694	ENSMUSG00000096603	8140	11,45
Ifltd1	intermediate filament tail domain containing 1	ENSMUSG00000054966	249186	11,45
Nfe2	nuclear factor, erythroid derived 2	ENSMUSG00000058794	10192	11,45
Slc22a20	solute carrier family 22 (organic anion transporter), member 20	ENSMUSG00000037451	15910	11,38
Col11a1	collagen, type XI, alpha 1	ENSMUSG00000027966	190179	11,38
Xylt2	xylosyltransferase II	ENSMUSG00000020868	13665	11,38

Sdccag3	serologically defined colon cancer antigen 3	ENSMUSG00000026927	6515	11,38
Csf1r	colony stimulating factor 1 receptor	ENSMUSG00000024621	26578	11,38
Usf1	upstream transcription factor 1	ENSMUSG00000026641	7830	11,38
Nfu1	NFU1 iron-sulfur cluster scaffold homolog (S. cerevisiae)	ENSMUSG00000029993	19226	11,38
Homer3	homer homolog 3 (Drosophila)	ENSMUSG00000003573	11535	11,38
		ENSMUSG00000073374	1471	11,38
Wnk4	WNK lysine deficient protein kinase 4	ENSMUSG00000035112	16843	11,38
Tmem18	transmembrane protein 18	ENSMUSG00000043061	6771	11,38
Cox17	cytochrome c oxidase assembly protein 17	ENSMUSG00000046516	5773	11,38
		ENSMUSG00000070972	30512	11,38
Aplf	aprataxin and PNKP like factor	ENSMUSG00000030051	43740	11,38
Ndufaf6	NADH dehydrogenase (ubiquinone) complex I, assembly factor 6	ENSMUSG00000050323	25161	11,38
Spg7	spastic paraplegia 7 homolog (human)	ENSMUSG00000000738	34819	11,38
Cat	catalase	ENSMUSG00000027187	31312	11,38
Sh3bp1	SH3-domain binding protein 1	ENSMUSG00000022436	19366	11,38
Mnd1	meiotic nuclear divisions 1 homolog (S. cerevisiae)	ENSMUSG00000033752	67853	11,38
Slc35g1	solute carrier family 35, member G1	ENSMUSG00000044026	9628	11,38
Gltscr1	glioma tumor suppressor candidate region gene 1	ENSMUSG00000070808	28234	11,38
Kcnmb3	potassium large conductance calcium-activated channel, subfamily M, beta member 3	ENSMUSG00000091091	19649	11,38
Igf2	insulin-like growth factor 2	ENSMUSG00000048583	16051	11,31
Ramp2		ENSMUSG00000001240	13519	11,31
Cd276	CD276 antigen	ENSMUSG00000035914	30833	11,31
Rps6kl1	ribosomal protein S6 kinase-like 1	ENSMUSG00000019235	15669	11,31
Psmb10	proteasome (prosome, macropain) subunit, beta type 10	ENSMUSG00000031897	2665	11,31
Kctd6	potassium channel tetramerisation domain containing 6	ENSMUSG00000021752	9420	11,31
1110017D15Rik	RIKEN cDNA 1110017D15 gene	ENSMUSG00000028441	12325	11,31
Adrbk1	adrenergic receptor kinase, beta 1	ENSMUSG00000024858	20222	11,31
		ENSMUSG00000032652	28778	11,31
Idi1	isopentenyl-diphosphate delta isomerase	ENSMUSG00000058258	6951	11,31
Slc25a20	solute carrier family 25 (mitochondrial carnitine/acylcarnitine translocase), member 20	ENSMUSG00000032602	22544	11,31
Zfp654	zinc finger protein 654	ENSMUSG00000047141	5971	11,31
Pwwp2b	PWWP domain containing 2B	ENSMUSG00000060260	18772	11,31
Grk4	G protein-coupled receptor kinase 4	ENSMUSG00000052783	94927	11,31
Uchl3	ubiquitin carboxyl-terminal esterase L3 (ubiquitin thiolesterase)	ENSMUSG00000022111	42158	11,31
Col4a5	collagen, type IV, alpha 5	ENSMUSG00000031274	213850	11,31
Cldn4	claudin 4	ENSMUSG00000047501	1809	11,31

Tctex1d4	Tctex1 domain containing 4	ENSMUSG00000047671	1945	11,31
Wnt2b	wingless related MMTV integration site 2b	ENSMUSG00000027840	16905	11,31
		ENSMUSG00000091191	907	11,31
Gm11992	predicted gene 11992	ENSMUSG00000040978	20763	11,24
Plp1	proteolipid protein (myelin) 1	ENSMUSG00000031425	17063	11,24
		ENSMUSG00000022820	7284	11,24
Lrrn2	leucine rich repeat protein 2, neuronal	ENSMUSG00000026443	59733	11,24
Aasdhppt	aminoadipate-semialdehyde dehydrogenase-phosphopantetheinyl transferase	ENSMUSG00000025894	14702	11,24
Klhl22	kelch-like 22	ENSMUSG00000022750	33765	11,24
Gad2	glutamic acid decarboxylase 2	ENSMUSG00000026787	71670	11,24
Zbtb45	zinc finger and BTB domain containing 45	ENSMUSG00000049600	4887	11,24
Ehbp1l1	EH domain binding protein 1-like 1	ENSMUSG00000024937	18942	11,24
Enthd2	ENTH domain containing 2	ENSMUSG00000025377	8201	11,24
Tmem240	transmembrane protein 240	ENSMUSG00000084845	5765	11,24
Smim1	small integral membrane protein 1	ENSMUSG00000078350	5761	11,24
Spdef	SAM pointed domain containing ets transcription factor	ENSMUSG00000024215	14600	11,24
Spon2	spondin 2, extracellular matrix protein	ENSMUSG00000037379	4721	11,24
Ccdc71l	coiled-coil domain containing 71 like	ENSMUSG00000090946	4155	11,24
Npm3	nucleoplasmin 3	ENSMUSG00000056209	1858	11,24
Haus3	HAUS augmin-like complex, subunit 3	ENSMUSG00000079555	15525	11,24
Ctgf	connective tissue growth factor	ENSMUSG00000019997	3242	11,24
Tmem68	transmembrane protein 68	ENSMUSG00000028232	25813	11,24
Ift27	intraflagellar transport 27	ENSMUSG00000016637	14644	11,16
Paqr4	progestin and adipoQ receptor family member IV	ENSMUSG00000023909	4145	11,16
Taf9b	TAF9B RNA polymerase II, TATA box binding protein (TBP)-associated factor	ENSMUSG00000047242	14276	11,16
1700021F05Rik	RIKEN cDNA 1700021F05 gene	ENSMUSG00000019797	15874	11,16
Dhcr7	7-dehydrocholesterol reductase	ENSMUSG00000058454	25266	11,16
Rbm7	RNA binding motif protein 7	ENSMUSG00000042396	6622	11,16
Jmjd4	jumonji domain containing 4	ENSMUSG00000036819	8523	11,16
Lhfp	lipoma HMGIC fusion partner	ENSMUSG00000048332	220152	11,16
Exosc9	exosome component 9	ENSMUSG00000027714	13124	11,16
Abca2	ATP-binding cassette, sub-family A (ABC1), member 2	ENSMUSG00000026944	19838	11,16
Rps2	ribosomal protein S2	ENSMUSG00000044533	3814	11,16
Mrps10	mitochondrial ribosomal protein S10	ENSMUSG00000034729	12531	11,16
Atad3a	ATPase family, AAA domain containing 3A	ENSMUSG00000029036	20453	11,16
Nudt7	nudix (nucleoside diphosphate linked moiety X)-type motif 7	ENSMUSG00000031767	18756	11,16
Acsf3	acyl-CoA synthetase family member 3	ENSMUSG00000015016	42376	11,16
Cyp2j9	cytochrome P450, family 2, subfamily j, polypeptide 9	ENSMUSG00000015224	23150	11,16

Vps33b	vacuolar protein sorting 33B (yeast)	ENSMUSG00000030534	21931	11,16
Ttc39c	tetratricopeptide repeat domain 39C	ENSMUSG00000024424	137125	11,16
Fut11	fucosyltransferase 11	ENSMUSG00000039357	5230	11,16
Mtmr9	myotubularin related protein 9	ENSMUSG00000035078	20338	11,16
Catsper2	cation channel, sperm associated 2	ENSMUSG00000033486	21162	11,16
Hspa1a	heat shock protein 1A	ENSMUSG00000091971	2956	11,16
Tdrd9	tudor domain containing 9	ENSMUSG00000054003	97296	11,16
Trhde	TRH-degrading enzyme	ENSMUSG00000050663	403485	11,16
Prrg4	proline rich Gla (G-carboxyglutamic acid) 4 (transmembrane)	ENSMUSG00000027171	19136	11,16
Pvr	poliovirus receptor	ENSMUSG00000040511	17583	11,16
Ddx60	DEAD (Asp-Glu-Ala-Asp) box polypeptide 60	ENSMUSG00000037921	109615	11,16
Lzic	leucine zipper and CTNNBIP1 domain containing	ENSMUSG00000028990	11440	11,09
Vps25	vacuolar protein sorting 25 (yeast)	ENSMUSG00000078656	5843	11,09
Spryd4	SPRY domain containing 4	ENSMUSG00000051346	1887	11,09
Ulk3	unc-51-like kinase 3	ENSMUSG00000032308	6782	11,09
Slc36a4	solute carrier family 36 (proton/amino acid symporter), member 4	ENSMUSG00000043885	32647	11,09
Txnl4b	thioredoxin-like 4B	ENSMUSG00000031723	8160	11,09
Rdh13	retinol dehydrogenase 13 (all-trans and 9-cis)	ENSMUSG00000008435	20880	11,09
		ENSMUSG00000050619	12853	11,09
Cops7b	COP9 (constitutive photomorphogenic) homolog, subunit 7b (Arabidopsis thaliana)	ENSMUSG00000026240	24075	11,09
Tctex1d1	Tctex1 domain containing 1	ENSMUSG00000028523	19216	11,09
Nup155	nucleoporin 155	ENSMUSG00000022142	50545	11,09
Lgals12	lectin, galactose binding, soluble 12	ENSMUSG00000024972	10534	11,09
Ramp1	receptor (calcitonin) activity modifying protein 1	ENSMUSG00000034353	45375	11,09
Lrrc29	leucine rich repeat containing 29	ENSMUSG00000041679	13936	11,09
Prox2	prospero homeobox 2	ENSMUSG00000042320	20618	11,09
Xpo5	exportin 5	ENSMUSG00000067150	39445	11,09
Morc1	microrchidia 1	ENSMUSG00000022652	199669	11,09
Adig	adipogenin	ENSMUSG00000044405	5587	11,09
		ENSMUSG00000000579	8104	11,02
Nabp2	nucleic acid binding protein 2	ENSMUSG00000025374	10098	11,02
Gas6	growth arrest specific 6	ENSMUSG00000031451	29162	11,02
Nprl2	nitrogen permease regulator-like 2	ENSMUSG00000010057	3496	11,02
		ENSMUSG00000073174	3773	11,02
Amigo1	adhesion molecule with Ig like domain 1	ENSMUSG00000050947	5952	11,02
Lrrc47	leucine rich repeat containing 47	ENSMUSG00000029028	9783	11,02
Zfp9	zinc finger protein 9	ENSMUSG00000072623	17371	11,02
Pih1d2	PIH1 domain containing 2	ENSMUSG00000000167	7680	11,02
Nme6	NME/NM23 nucleoside diphosphate kinase 6	ENSMUSG00000032478	10368	11,02
Ccdc9	coiled-coil domain containing 9	ENSMUSG00000041375	12754	11,02
Chaf1b	chromatin assembly factor 1, subunit B (p60)	ENSMUSG00000022945	22215	11,02

2410066E13Rik	RIKEN cDNA 2410066E13 gene	ENSMUSG00000038065	18635	11,02
Apoh	apolipoprotein H	ENSMUSG00000000049	71043	11,02
Tm9sf1	transmembrane 9 superfamily member 1	ENSMUSG00000002320	7842	11,02
AB124611	cDNA sequence AB124611	ENSMUSG00000057191	19158	11,02
Wdr27	WD repeat domain 27	ENSMUSG00000046991	105323	11,02
Dcst1	DC-STAMP domain containing 1	ENSMUSG00000042672	15035	10,95
Snrnp25	small nuclear ribonucleoprotein 25 (U11/U12)	ENSMUSG00000040767	3570	10,95
Zfp764	zinc finger protein 764	ENSMUSG00000045757	3155	10,95
		ENSMUSG00000020211	6727	10,95
Slc7a4	solute carrier family 7 (cationic amino acid transporter, y+ system), member 4	ENSMUSG00000022756	4981	10,95
Ankrd36	ankyrin repeat domain 36	ENSMUSG00000020481	119654	10,95
Ccdc114	coiled-coil domain containing 114	ENSMUSG00000040189	24885	10,95
Kif14	kinesin family member 14	ENSMUSG00000041498	63554	10,95
Rfxank	regulatory factor X-associated ankyrin-containing protein	ENSMUSG00000036120	8386	10,95
Entpd7	ectonucleoside triphosphate diphosphohydrolase 7	ENSMUSG00000025192	44026	10,95
Mss51	MSS51 mitochondrial translational activator	ENSMUSG00000021815	14038	10,95
Zdhhc13	zinc finger, DHHC domain containing 13	ENSMUSG00000030471	38438	10,95
E330013P04Rik	RIKEN cDNA E330013P04 gene	ENSMUSG00000053117	17572	10,95
Kank2	KN motif and ankyrin repeat domains 2	ENSMUSG00000032194	31774	10,95
Gm13247	predicted gene 13247	ENSMUSG00000056300	37369	10,95
Ankrd26	ankyrin repeat domain 26	ENSMUSG00000007827	59693	10,95
Plekha4	pleckstrin homology domain containing, family A (phosphoinositide binding specific) member 4	ENSMUSG00000040428	27900	10,95
March10	membrane-associated ring finger (C3HC4) 10	ENSMUSG00000078627	95938	10,95
Ppp5c	protein phosphatase 5, catalytic subunit	ENSMUSG00000003099	23285	10,87
Icmt	isoprenylcysteine carboxyl methyltransferase	ENSMUSG00000039662	9895	10,87
Morn2	MORN repeat containing 2	ENSMUSG00000045257	7262	10,87
Col12a1	collagen, type XII, alpha 1	ENSMUSG00000032332	119841	10,87
Fam109a	family with sequence similarity 109, member A	ENSMUSG00000044134	5572	10,87
Atg4a	autophagy related 4A, cysteine peptidase	ENSMUSG00000079418	207364	10,87
Dhx35	DEAH (Asp-Glu-Ala-His) box polypeptide 35	ENSMUSG00000027655	63408	10,87
Ttc21b	tetratricopeptide repeat domain 21B	ENSMUSG00000034848	72291	10,87
Jag2	jagged 2	ENSMUSG00000002799	20906	10,87
Tcn2	transcobalamin 2	ENSMUSG00000020432	14968	10,87
Ptpn14	protein tyrosine phosphatase, non-receptor type 14	ENSMUSG00000026604	148428	10,87
Gm960	predicted gene 960	ENSMUSG00000071691	72828	10,87
Tti1	TELO2 interacting protein 1	ENSMUSG00000027650	46631	10,87

Dis3	DIS3 mitotic control homolog (S. cerevisiae)	ENSMUSG00000033166	23137	10,87
Trp73	transformation related protein 73	ENSMUSG00000029026	83956	10,87
Khdrbs2	KH domain containing, RNA binding, signal transduction associated 2	ENSMUSG00000026058	484933	10,87
		ENSMUSG00000091294	37953	10,87
Asphd2	aspartate beta-hydroxylase domain containing 2	ENSMUSG00000029348	6766	10,80
Slco4a1	solute carrier organic anion transporter family, member 4a1	ENSMUSG00000038963	18623	10,80
Rpf1	ribosome production factor 1 homolog (S. cerevisiae)	ENSMUSG00000028187	15076	10,80
0610037L13Rik	RIKEN cDNA 0610037L13 gene	ENSMUSG00000028608	9572	10,80
Alg11	asparagine-linked glycosylation 11 (alpha-1,2-mannosyltransferase)	ENSMUSG00000063362	10907	10,80
Kif20a	kinesin family member 20A	ENSMUSG00000003779	8642	10,80
Nrarp	Notch-regulated ankyrin repeat protein	ENSMUSG00000078202	2573	10,80
Haus8	4HAUS augmin-like complex, subunit 8	ENSMUSG00000035439	24374	10,80
Armc7	armadillo repeat containing 7	ENSMUSG00000057219	14801	10,80
Spg20	spastic paraplegia 20, spartin (Troyer syndrome) homolog (human)	ENSMUSG00000036580	25215	10,80
Aaas	achalasia, adrenocortical insufficiency, alacrimia	ENSMUSG00000036678	12513	10,80
Pdcd11	programmed cell death 11	ENSMUSG00000025047	40378	10,80
Tmem125	transmembrane protein 125	ENSMUSG00000050854	3104	10,80
Myo19	myosin XIX	ENSMUSG00000020527	31079	10,80
Spata16	spermatogenesis associated 16	ENSMUSG00000039335	345593	10,80
		ENSMUSG00000027667	5391	10,73
Tmem236	transmembrane protein 236	ENSMUSG00000061531	47448	10,73
Tomm40l	translocase of outer mitochondrial membrane 40 homolog-like (yeast)	ENSMUSG00000005674	4713	10,73
Bckdk	branched chain ketoacid dehydrogenase kinase	ENSMUSG00000030802	5583	10,73
Riiad1	regulatory subunit of type II PKA R-subunit (RIIa) domain containing 1	ENSMUSG00000028139	8607	10,73
Ppp1r42	protein phosphatase 1, regulatory subunit 42	ENSMUSG00000025916	40513	10,73
Pcid2	PCI domain containing 2	ENSMUSG00000038542	28271	10,73
Crip1	cysteine-rich protein 1 (intestinal)	ENSMUSG00000006360	1866	10,73
Lyplal1	lysophospholipase-like 1	ENSMUSG00000039246	29580	10,73
		ENSMUSG00000095669	13135	10,73
Adcy7	adenylate cyclase 7	ENSMUSG00000031659	57560	10,73
		ENSMUSG00000017688	151746	10,73
1700028K03Rik	RIKEN cDNA 1700028K03 gene	ENSMUSG00000089798	42606	10,66
Mpg	N-methylpurine-DNA glycosylase	ENSMUSG00000020287	6196	10,66
		ENSMUSG00000079043	16188	10,66
		ENSMUSG00000003438	6557	10,66
Gtf3a	general transcription factor III A	ENSMUSG00000016503	6958	10,66
Utp15	UTP15, U3 small nucleolar ribonucleoprotein, homolog (yeast)	ENSMUSG00000041747	16148	10,66
Pwp1	PWP1 homolog (S. cerevisiae)	ENSMUSG00000001785	17266	10,66
Fads2	fatty acid desaturase 2	ENSMUSG00000024665	37340	10,66

Foxn2	forkhead box N2	ENSMUSG00000034998	49823	10,66
Zfr2	zinc finger RNA binding protein 2	ENSMUSG00000034949	18969	10,66
		ENSMUSG00000020268	34051	10,66
Klhl11	kelch-like 11	ENSMUSG00000048732	10128	10,66
Nmd3	NMD3 homolog (S. cerevisiae)	ENSMUSG00000027787	27058	10,66
Adap1	ArfGAP with dual PH domains 1	ENSMUSG00000056413	53747	10,66
Taf4a	TAF4A RNA polymerase II, TATA box binding protein (TBP)-associated factor	ENSMUSG00000039117	64501	10,66
Pdgfd	platelet-derived growth factor, D polypeptide	ENSMUSG00000032006	210232	10,66
		ENSMUSG00000026141	325583	10,66
Slpi	secretory leukocyte peptidase inhibitor	ENSMUSG00000017002	35026	10,66
Esr1	estrogen receptor 1 (alpha)	ENSMUSG00000019768	394022	10,66
		ENSMUSG00000043913	164345	10,66
Has2	hyaluronan synthase 2	ENSMUSG00000022367	28913	10,66
Ttc29	tetratricopeptide repeat domain 29	ENSMUSG00000037101	181030	10,66
Ccdc80	coiled-coil domain containing 80	ENSMUSG00000022665	34523	10,58
		ENSMUSG00000030032	7037	10,58
Fscn1	fascin homolog 1, actin bundling protein (Strongylocentrotus purpuratus)	ENSMUSG00000029581	12843	10,58
Exo5	exonuclease 5	ENSMUSG00000028629	3822	10,58
Map7d2	MAP7 domain containing 2	ENSMUSG00000041020	84186	10,58
Sgol2	shugoshin-like 2 (S. pombe)	ENSMUSG00000026039	29929	10,58
Tnfrsf12a	tumor necrosis factor receptor superfamily, member 12a	ENSMUSG00000023905	2003	10,58
Dnajc28	DnaJ (Hsp40) homolog, subfamily C, member 28	ENSMUSG00000039763	4770	10,58
Rusc1	RUN and SH3 domain containing 1	ENSMUSG00000041263	9383	10,58
Angel1	angel homolog 1 (Drosophila)	ENSMUSG00000021257	25959	10,58
Znf512b	zinc finger protein 512B	ENSMUSG00000000823	10701	10,58
Zc3hc1	zinc finger, C3HC type 1	ENSMUSG00000039130	24649	10,58
Prepl	prolyl endopeptidase-like	ENSMUSG00000024127	26791	10,58
Hexb	hexosaminidase B	ENSMUSG00000021665	22026	10,58
Tmtc3	transmembrane and tetratricopeptide repeat containing 3	ENSMUSG00000036676	43449	10,58
Shisa2	shisa homolog 2 (Xenopus laevis)	ENSMUSG00000044461	6378	10,58
Itga11	integrin alpha 11	ENSMUSG00000032243	106157	10,58
Nwd1	NACHT and WD repeat domain containing 1	ENSMUSG00000048148	68036	10,58
Sema3c	sema domain, immunoglobulin domain (Ig), short basic domain, secreted, (semaphorin) 3C	ENSMUSG00000028780	155988	10,58
Zfp142	zinc finger protein 142	ENSMUSG00000026135	23120	10,58
Asah2	N-acylsphingosine amidohydrolase 2	ENSMUSG00000024887	76816	10,58
Capn3	calpain 3	ENSMUSG00000079110	48895	10,58
Nudt18	nudix (nucleoside diphosphate linked moiety X)-type motif 18	ENSMUSG00000045211	4725	10,51
Ifit1	interferon-induced protein with tetratricopeptide repeats 1	ENSMUSG00000034459	9139	10,51
Pcdhb16	protocadherin beta 16	ENSMUSG00000047910	5268	10,51
		ENSMUSG00000027099	52629	10,51

Fbxo31	F-box protein 31	ENSMUSG00000052934	29367	10,51
Cpsf4l	cleavage and polyadenylation specific factor 4-like	ENSMUSG00000018727	11846	10,51
Hspbp1	HSPA (heat shock 70kDa) binding protein, cytoplasmic cochaperone 1	ENSMUSG00000063802	24443	10,51
		ENSMUSG00000029447	58382	10,51
Wdr24	WD repeat domain 24	ENSMUSG00000025737	5104	10,51
Acot9	acyl-CoA thioesterase 9	ENSMUSG00000025287	35212	10,51
C330027C09Rik	RIKEN cDNA C330027C09 gene	ENSMUSG00000033031	25525	10,51
Klhl21	kelch-like 21	ENSMUSG00000073700	8878	10,51
Kif18a	kinesin family member 18A	ENSMUSG00000027115	61010	10,51
1700029J07Rik	RIKEN cDNA 1700029J07 gene	ENSMUSG00000071103	21647	10,51
B230325K18Rik	RIKEN cDNA B230325K18 gene	ENSMUSG00000063254	2623	10,51
Slfn9	schlafen 9	ENSMUSG00000069793	12202	10,51
Slco6b1	solute carrier organic anion transporter family, member 6b1	ENSMUSG00000045463	85707	10,51
		ENSMUSG00000000794	147598	10,51
Dynlt1f	dynein light chain Tctex-type 1F	ENSMUSG00000095677	6999	10,44
Bmyc	brain expressed myelocytomatosis oncogene	ENSMUSG00000049086	839	10,44
Gins4	GINS complex subunit 4 (Sld5 homolog)	ENSMUSG00000031546	11059	10,44
BC048502	cDNA sequence BC048502	ENSMUSG00000053508	9495	10,44
Tbc1d7	TBC1 domain family, member 7	ENSMUSG00000021368	19758	10,44
Gprin1	G protein-regulated inducer of neurite outgrowth 1	ENSMUSG00000069227	13204	10,44
Plbd2	phospholipase B domain containing 2	ENSMUSG00000029598	20344	10,44
Ssbp4	single stranded DNA binding protein 4	ENSMUSG00000070003	10824	10,44
		ENSMUSG00000095779	123187	10,44
Fgf18	fibroblast growth factor 18	ENSMUSG00000057967	29971	10,44
Prss57	protease, serine 57	ENSMUSG00000020323	9488	10,44
2410004P03Rik	RIKEN cDNA 2410004P03 gene	ENSMUSG00000071398	6770	10,44
Acap3	ArfGAP with coiled-coil, ankyrin repeat and PH domains 3	ENSMUSG00000029033	15430	10,44
Capn9	calpain 9	ENSMUSG00000031981	42621	10,44
1700111E14Rik	RIKEN cDNA 1700111E14 gene	ENSMUSG00000029837	48730	10,44
Rhobtb1	Rho-related BTB domain containing 1	ENSMUSG00000019944	140358	10,44
Tsnaxip1	translin-associated factor X (Tsnax) interacting protein 1	ENSMUSG00000031893	16933	10,44
1110034G24Rik	RIKEN cDNA 1110034G24 gene	ENSMUSG00000044991	64125	10,44
Fam83f	family with sequence similarity 83, member F	ENSMUSG00000022408	28574	10,44
Fam47e	family with sequence similarity 47, member E	ENSMUSG00000057068	36211	10,44
4921513I03Rik	RIKEN cDNA 4921513I03 gene	ENSMUSG00000044544	13113	10,44
Creb3l1	cAMP responsive element binding protein 3-like 1	ENSMUSG00000027230	42175	10,37
Hsd17b6	hydroxysteroid (17-beta) dehydrogenase 6	ENSMUSG00000025396	9738	10,37
Mpdz	multiple PDZ domain protein	ENSMUSG00000028402	164316	10,37
Noa1	nitric oxide associated 1	ENSMUSG00000036285	15903	10,37
Psat1	phosphoserine aminotransferase 1	ENSMUSG00000024640	42660	10,37

Atpaf2	ATP synthase mitochondrial F1 complex assembly factor 2	ENSMUSG00000042709	17832	10,37
Abhd4	abhydrolase domain containing 4	ENSMUSG00000040997	15041	10,37
Tmem50b	transmembrane protein 50B	ENSMUSG00000022964	23298	10,37
Smarcd3	SWI/SNF related, matrix associated, actin dependent regulator of chromatin, subfamily d, member 3	ENSMUSG00000028949	11195	10,37
Ipo4	importin 4	ENSMUSG00000002319	12496	10,37
		ENSMUSG00000069844	14401	10,37
Ccdc136	coiled-coil domain containing 136	ENSMUSG00000029769	30686	10,37
Cd84	CD84 antigen	ENSMUSG00000038147	51022	10,37
Tbc1d2	TBC1 domain family, member 2	ENSMUSG00000039813	45820	10,37
Dusp12	dual specificity phosphatase 12	ENSMUSG00000026659	12043	10,37
Srl	sarcalumenin	ENSMUSG00000022519	61601	10,37
Slc27a1	solute carrier family 27 (fatty acid transporter), member 1	ENSMUSG00000031808	17782	10,37
Slc44a5	solute carrier family 44, member 5	ENSMUSG00000028360	298287	10,37
Pgm1	phosphoglucomutase 1	ENSMUSG00000029171	35420	10,37
Mbip	MAP3K12 binding inhibitory protein 1	ENSMUSG00000021028	17589	10,37
Wtip	WT1-interacting protein	ENSMUSG00000036459	23726	10,37
		ENSMUSG00000035861	18732	10,37
Apol7a	apolipoprotein L 7a	ENSMUSG00000010601	10890	10,37
Ccdc28b	coiled coil domain containing 28B	ENSMUSG00000028795	4674	10,29
Gng3	guanine nucleotide binding protein (G protein), gamma 3	ENSMUSG00000071658	2318	10,29
Ephx4	epoxide hydrolase 4	ENSMUSG00000033805	27300	10,29
Blvra	biliverdin reductase A	ENSMUSG00000001999	26420	10,29
Pofut2	protein O-fucosyltransferase 2	ENSMUSG00000020260	10287	10,29
Asns	asparagine synthetase	ENSMUSG00000029752	18086	10,29
Nudt2	nudix (nucleoside diphosphate linked moiety X)-type motif 2	ENSMUSG00000028443	15776	10,29
Myg1	melanocyte proliferating gene 1	ENSMUSG00000001285	6431	10,29
Vit	vitrin	ENSMUSG00000024076	119347	10,29
Rassf7	Ras association (RalGDS/AF-6) domain family (N-terminal) member 7	ENSMUSG00000038618	2799	10,29
1810043G02Rik	RIKEN cDNA 1810043G02 gene	ENSMUSG00000020284	8382	10,29
Fahd2a	fumarylacetoacetate hydrolase domain containing 2A	ENSMUSG00000027371	8351	10,29
		ENSMUSG00000068859	12782	10,29
Dnmt3b	DNA methyltransferase 3B	ENSMUSG00000027478	38281	10,29
Ctf1	cardiotrophin 1	ENSMUSG00000042340	5450	10,29
Zbtb25	zinc finger and BTB domain containing 25	ENSMUSG00000056459	21821	10,29
Camsap3	calmodulin regulated spectrin-associated protein family, member 3	ENSMUSG00000044433	21625	10,29
Cdca4	cell division cycle associated 4	ENSMUSG00000047832	9153	10,29
Pycrl	pyrroline-5-carboxylate reductase-like	ENSMUSG00000022571	5098	10,29
Neil3	nei like 3 (E. coli)	ENSMUSG00000039396	52199	10,29
		ENSMUSG00000030671	123971	10,29
Rab32	RAB32, member RAS oncogene family	ENSMUSG00000019832	13223	10,29

Eno3	enolase 3, beta muscle	ENSMUSG00000060600	5312	10,29
Kcnk12	potassium channel, subfamily K, member 12	ENSMUSG00000050138	52158	10,29
Zufsp	zinc finger with UFM1-specific peptidase domain	ENSMUSG00000039531	24277	10,29
Tnip2	TNFAIP3 interacting protein 2	ENSMUSG00000059866	17911	10,29
Bmp8a	bone morphogenetic protein 8a	ENSMUSG00000032726	30608	10,29
Ccdc17	coiled-coil domain containing 17	ENSMUSG00000034035	3613	10,29
Grip2	glutamate receptor interacting protein 2	ENSMUSG00000030098	65742	10,29
Gdap1l1	ganglioside-induced differentiation-associated protein 1-like 1	ENSMUSG00000017943	16849	10,22
Tor1a	torsin family 1, member A (torsin A)	ENSMUSG00000026849	7307	10,22
		ENSMUSG00000095464	15258	10,22
Sars2	seryl-aminoacyl-tRNA synthetase 2	ENSMUSG00000070699	11912	10,22
		ENSMUSG00000096115	35146	10,22
		ENSMUSG00000043629	40937	10,22
Pdk2	pyruvate dehydrogenase kinase, isoenzyme 2	ENSMUSG00000038967	15097	10,22
Ccdc65	coiled-coil domain containing 65	ENSMUSG00000003354	15120	10,22
Rpap1	RNA polymerase II associated protein 1	ENSMUSG00000034032	24234	10,22
Klf10	Kruppel-like factor 10	ENSMUSG00000037465	6295	10,22
Tmem132c	transmembrane protein 132C	ENSMUSG00000034324	323986	10,22
		ENSMUSG00000021763	62628	10,22
Porcn	porcupine homolog (Drosophila)	ENSMUSG00000031169	12678	10,22
Apeh	acylpeptide hydrolase	ENSMUSG00000032590	9064	10,22
Tspan9	tetraspanin 9	ENSMUSG00000030352	182199	10,22
Papolg	poly(A) polymerase gamma	ENSMUSG00000020273	32608	10,22
Setd1a	SET domain containing 1A	ENSMUSG00000042308	23453	10,22
Casp7	caspase 7	ENSMUSG00000025076	45216	10,22
BC107364	cDNA sequence BC107364	ENSMUSG00000046317	18519	10,22
Mfsd7b	major facilitator superfamily domain containing 7B	ENSMUSG00000066595	20359	10,22
Hsf3	heat shock transcription factor 3	ENSMUSG00000045802	150117	10,22
		ENSMUSG00000061864	484134	10,22
Gpx6	glutathione peroxidase 6	ENSMUSG00000004341	7408	10,15
Ptges2	prostaglandin E synthase 2	ENSMUSG00000026820	6844	10,15
Rabggta	Rab geranylgeranyl transferase, a subunit	ENSMUSG00000040472	6385	10,15
1110065P20Rik	RIKEN cDNA 1110065P20 gene	ENSMUSG00000078570	1246	10,15
Ccdc103	coiled-coil domain containing 103	ENSMUSG00000020930	4013	10,15
Mpnd	MPN domain containing	ENSMUSG00000003199	11080	10,15
Cdr2	cerebellar degeneration-related 2	ENSMUSG00000030878	25277	10,15
Zyx	zyxin	ENSMUSG00000029860	10319	10,15
Mterfd1	MTERF domain containing 1	ENSMUSG00000021519	26121	10,15
		ENSMUSG00000071180	2723	10,15
Dclre1a	DNA cross-link repair 1A, PSO2 homolog (S. cerevisiae)	ENSMUSG00000025077	19062	10,15
Rbck1	RanBP-type and C3HC4-type zinc finger containing 1	ENSMUSG00000027466	16320	10,15
Zfp809	zinc finger protein 809	ENSMUSG00000057982	17484	10,15
Ccdc134	coiled-coil domain containing 134	ENSMUSG00000068114	14279	10,15
Zfp846	zinc finger protein 846	ENSMUSG00000058192	13870	10,15

Zdhhc15	zinc finger, DHHC domain containing 15	ENSMUSG00000033906	134096	10,15
Med12	mediator of RNA polymerase II transcription, subunit 12 homolog (yeast)	ENSMUSG00000079487	23436	10,15
Myo3b	myosin IIIB	ENSMUSG00000042064	390073	10,15
Oasl2	2'-5' oligoadenylate synthetase-like 2	ENSMUSG00000029561	15299	10,15
Scn4b	sodium channel, type IV, beta	ENSMUSG00000046480	15716	10,08
Lfng	LFNG O-fucosylpeptide 3-beta-N-acetylglucosaminyltransferase	ENSMUSG00000029570	8203	10,08
Tmem120a	transmembrane protein 120A	ENSMUSG00000039886	8787	10,08
		ENSMUSG00000078184	1338	10,08
Bivm	basic, immunoglobulin-like variable motif containing	ENSMUSG00000041684	25814	10,08
		ENSMUSG00000069808	35157	10,08
Dbr1	debranching enzyme homolog 1 (S. cerevisiae)	ENSMUSG00000032469	8665	10,08
Wdr45	WD repeat domain 45	ENSMUSG00000039382	6229	10,08
Lmf1	lipase maturation factor 1	ENSMUSG00000002279	83653	10,08
Dnaaf1	dynein, axonemal assembly factor 1	ENSMUSG00000031831	23220	10,08
Mief2	mitochondrial elongation factor 2	ENSMUSG00000018599	4554	10,08
		ENSMUSG00000071584	390	10,08
Rgs11	regulator of G-protein signaling 11	ENSMUSG00000024186	8374	10,08
Fbxo6	F-box protein 6	ENSMUSG00000055401	6425	10,08
Cwf19l1	CWF19-like 1, cell cycle control (S. pombe)	ENSMUSG00000025200	27233	10,08
Pecam1	platelet/endothelial cell adhesion molecule 1	ENSMUSG00000020717	96412	10,08
Gnpda1	glucosamine-6-phosphate deaminase 1	ENSMUSG00000052102	11446	10,08
Igsf11	immunoglobulin superfamily, member 11	ENSMUSG00000022790	125689	10,08
Colgalt2	collagen beta(1-O)galactosyltransferase 2	ENSMUSG00000032649	110866	10,08
		ENSMUSG00000096375	745	10,08
Cyp2c69	cytochrome P450, family 2, subfamily c, polypeptide 69	ENSMUSG00000092008	44110	10,08
Mex3d	mex3 homolog D (C. elegans)	ENSMUSG00000048696	7305	10,00
Arl4c	ADP-ribosylation factor-like 4C	ENSMUSG00000049866	29097	10,00
Pex11b	peroxisomal biogenesis factor 11 beta	ENSMUSG00000028102	17726	10,00
Rad1	RAD1 homolog (S. pombe)	ENSMUSG00000022248	13046	10,00
Zfp112	zinc finger protein 112	ENSMUSG00000052675	15639	10,00
Lmo2	LIM domain only 2	ENSMUSG00000032698	23893	10,00
Homez	homeodomain leucine zipper-encoding gene	ENSMUSG00000057156	18226	10,00
Fastkd3	FAST kinase domains 3	ENSMUSG00000021532	10032	10,00
Zfpl1	zinc finger like protein 1	ENSMUSG00000024792	8458	10,00
Ccdc125	coiled-coil domain containing 125	ENSMUSG00000048924	27760	10,00
Gba2	glucosidase beta 2	ENSMUSG00000028467	11946	10,00
Cyba	cytochrome b-245, alpha polypeptide	ENSMUSG00000006519	8169	10,00
St3gal1	ST3 beta-galactoside alpha-2,3-sialyltransferase 1	ENSMUSG00000013846	11118	10,00

Oas1c	2'-5' oligoadenylate synthetase 1C	ENSMUSG00000001166	12321	10,00
Itga4	integrin alpha 4	ENSMUSG00000027009	77698	10,00
Polq	polymerase (DNA directed), theta	ENSMUSG00000034206	83632	10,00
Prkar1b	protein kinase, cAMP dependent regulatory, type I beta	ENSMUSG00000025855	132696	10,00
Cntnap3	contactin associated protein-like 3	ENSMUSG00000033063	166298	10,00
Gramd2	GRAM domain containing 2	ENSMUSG00000074259	38731	10,00
Ica1l	islet cell autoantigen 1-like	ENSMUSG00000026018	54018	10,00
		ENSMUSG00000027068	24089	10,00
1700012B09Rik	RIKEN cDNA 1700012B09 gene	ENSMUSG00000031927	12831	9,93
Mrpl15	mitochondrial ribosomal protein L15	ENSMUSG00000033845	12534	9,93
Hspg2	perlecan (heparan sulfate proteoglycan 2)	ENSMUSG00000028763	101862	9,93
Tnc	tenascin C	ENSMUSG00000028364	87231	9,93
Hcn2	hyperpolarization-activated, cyclic nucleotide-gated K+ 2	ENSMUSG00000020331	19475	9,93
Slc39a3	solute carrier family 39 (zinc transporter), member 3	ENSMUSG00000046822	8889	9,93
Ccng2	cyclin G2	ENSMUSG00000029385	8975	9,93
Ovca2	candidate tumor suppressor in ovarian cancer 2	ENSMUSG00000038268	2894	9,93
Fam111a	family with sequence similarity 111, member A	ENSMUSG00000024691	44029	9,93
Zik1	zinc finger protein interacting with K protein 1	ENSMUSG00000030393	8166	9,93
Pask	PAS domain containing serine/threonine kinase	ENSMUSG00000026274	34713	9,93
Scara5	scavenger receptor class A, member 5 (putative)	ENSMUSG00000022032	98424	9,93
Espl1	extra spindle poles-like 1 (S. cerevisiae)	ENSMUSG00000058290	28064	9,93
Gstt3	glutathione S-transferase, theta 3	ENSMUSG00000001665	7300	9,93
Ndc80	NDC80 homolog, kinetochore complex component (S. cerevisiae)	ENSMUSG00000024056	30758	9,93
Vps37c	vacuolar protein sorting 37C (yeast)	ENSMUSG00000048832	25605	9,93
Nmi	N-myc (and STAT) interactor	ENSMUSG00000026946	25008	9,93
Zc2hc1c	zinc finger, C2HC-type containing 1C	ENSMUSG00000045064	10768	9,93
Kctd15	potassium channel tetramerisation domain containing 15	ENSMUSG00000030499	16496	9,93
Pla2g6	phospholipase A2, group VI	ENSMUSG00000042632	42163	9,93
		ENSMUSG00000036995	38852	9,93
Nmb	neuromedin B	ENSMUSG00000025723	2850	9,93
Zfp40	zinc finger protein 40	ENSMUSG00000002617	19384	9,93
Clnk	cytokine-dependent hematopoietic cell linker	ENSMUSG00000039315	170351	9,93
Atp2a1	ATPase, Ca++ transporting, cardiac muscle, fast twitch 1	ENSMUSG00000030730	17251	9,93
Hsf5	heat shock transcription factor family member 5	ENSMUSG00000070345	42379	9,93
		ENSMUSG00000074500	22694	9,93
E2f1	E2F transcription factor 1	ENSMUSG00000027490	10486	9,86
Adam23	a disintegrin and metallopeptidase domain 23	ENSMUSG00000025964	150625	9,86
Tcp11l2	t-complex 11 (mouse) like 2	ENSMUSG00000020034	37734	9,86

B3galt6	UDP-Gal:betaGal beta 1,3-galactosyltransferase, polypeptide 6	ENSMUSG00000050796	3213	9,86
Phldb1	pleckstrin homology-like domain, family B, member 1	ENSMUSG00000048537	48895	9,86
A830031A19Rik	RIKEN cDNA A830031A19 gene	ENSMUSG00000055010	26100	9,86
Kbtbd11	kelch repeat and BTB (POZ) domain containing 11	ENSMUSG00000055675	22306	9,86
Snapin	SNAP-associated protein	ENSMUSG00000001018	3008	9,86
Alyref2	Aly/REF export factor 2	ENSMUSG00000060244	1273	9,86
Rab26	RAB26, member RAS oncogene family	ENSMUSG00000079657	6970	9,86
Tsta3	tissue specific transplantation antigen P35B	ENSMUSG00000022570	5047	9,86
Mapk13	mitogen-activated protein kinase 13	ENSMUSG00000004864	9392	9,86
Acer2	alkaline ceramidase 2	ENSMUSG00000038007	60427	9,86
Polg	polymerase (DNA directed), gamma	ENSMUSG00000039176	17572	9,86
Vps11	vacuolar protein sorting 11 (yeast)	ENSMUSG00000032127	13565	9,86
Vegfa	vascular endothelial growth factor A	ENSMUSG00000023951	15385	9,86
		ENSMUSG00000003233	15539	9,86
Sh3bgrl2	SH3 domain binding glutamic acid-rich protein like 2	ENSMUSG00000032261	51955	9,86
Tex26	testis expressed 26	ENSMUSG00000029660	30915	9,86
D630044L22Rik	RIKEN cDNA gene D630044L22 gene	ENSMUSG00000097405	12139	9,86
Hmgcs2	3-hydroxy-3-methylglutaryl-Coenzyme A synthase 2	ENSMUSG00000027875	30304	9,86
4930422G04Rik	RIKEN cDNA 4930422G04 gene	ENSMUSG00000051278	64533	9,86
Rs1	retinoschisis (X-linked, juvenile) 1 (human)	ENSMUSG00000031293	31651	9,86
Neb	nebulin	ENSMUSG00000026950	202152	9,86
		ENSMUSG00000078493	11610	9,79
Dok4	docking protein 4	ENSMUSG00000040631	12485	9,79
Phldb2	pleckstrin homology-like domain, family B, member 2	ENSMUSG00000033149	207356	9,79
Armcx2	armadillo repeat containing, X-linked 2	ENSMUSG00000033436	5077	9,79
		ENSMUSG00000000876	17951	9,79
		ENSMUSG00000060121	15078	9,79
Ctrc	chymotrypsin C (caldecrin)	ENSMUSG00000062478	31398	9,79
Bccip	BRCA2 and CDKN1A interacting protein	ENSMUSG00000030983	11813	9,79
Fam96a	family with sequence similarity 96, member A	ENSMUSG00000032381	12345	9,79
Fbxw17	F-box and WD-40 domain protein 17	ENSMUSG00000037816	15917	9,79
Myo7a	myosin VIIA	ENSMUSG00000030761	68465	9,79
Bcl7b	B cell CLL/lymphoma 7B	ENSMUSG00000029681	13573	9,79
Stmnd1	stathmin domain containing 1	ENSMUSG00000063529	26395	9,79
Mafk	v-maf musculoaponeurotic fibrosarcoma oncogene family, protein K (avian)	ENSMUSG00000018143	11141	9,79
Paqr5	progestin and adipoQ receptor family member V	ENSMUSG00000032278	73376	9,79

Nrip2	nuclear receptor interacting protein 2	ENSMUSG00000001520	9637	9,79
Rabl3	RAB, member of RAS oncogene family-like 3	ENSMUSG00000022827	32501	9,79
1810024B03Rik	RIKEN cDNA 1810024B03 gene	ENSMUSG00000044145	21926	9,79
Tshr	thyroid stimulating hormone receptor	ENSMUSG00000020963	139517	9,79
Ccdc79	coiled-coil domain containing 79	ENSMUSG00000052616	63192	9,79
		ENSMUSG00000074829	18760	9,79
		ENSMUSG00000097490	57574	9,79
Stpg1	sperm tail PG rich repeat containing 1	ENSMUSG00000028801	43014	9,79
		ENSMUSG00000078435	13759	9,79
Txk	TXK tyrosine kinase	ENSMUSG00000054892	56796	9,79
Casp4	caspase 4, apoptosis-related cysteine peptidase	ENSMUSG00000033538	27956	9,79
Cxx1c	CAAX box 1C	ENSMUSG00000051851	1211	9,71
Laptm5	lysosomal-associated protein transmembrane 5	ENSMUSG00000028581	23017	9,71
Pkmyt1	protein kinase, membrane associated tyrosine/threonine 1	ENSMUSG00000023908	10394	9,71
		ENSMUSG00000059278	1194	9,71
Ccdc147	coiled-coil domain containing 147	ENSMUSG00000046585	97668	9,71
Svop	SV2 related protein	ENSMUSG00000042078	64661	9,71
Hmgcl	3-hydroxy-3-methylglutaryl-Coenzyme A lyase	ENSMUSG00000028672	16170	9,71
Sumf2	sulfatase modifying factor 2	ENSMUSG00000025538	17061	9,71
Map3k11	mitogen-activated protein kinase kinase kinase 11	ENSMUSG00000004054	13732	9,71
Mis18bp1	MIS18 binding protein 1	ENSMUSG00000047534	39871	9,71
		ENSMUSG00000063180	186	9,71
Tmem201	transmembrane protein 201	ENSMUSG00000044700	22670	9,71
Plekha8	pleckstrin homology domain containing, family A (phosphoinositide binding specific) member 8	ENSMUSG00000005225	50729	9,71
Bahd1	bromo adjacent homology domain containing 1	ENSMUSG00000040007	24152	9,71
AI464131	expressed sequence AI464131	ENSMUSG00000046312	7473	9,71
Etaa1	Ewing's tumor-associated antigen 1	ENSMUSG00000016984	15120	9,71
Plcg2	phospholipase C, gamma 2	ENSMUSG00000034330	136850	9,71
Spsb2	splA/ryanodine receptor domain and SOCS box containing 2	ENSMUSG00000038451	1959	9,71
Zfp944	zinc finger protein 944	ENSMUSG00000033972	23412	9,71
		ENSMUSG00000034912	752982	9,71
Narg2	NMDA receptor-regulated gene 2	ENSMUSG00000032235	35217	9,71
Rab17	RAB17, member RAS oncogene family	ENSMUSG00000026304	11529	9,71
Ccdc78	coiled-coil domain containing 78	ENSMUSG00000071202	3934	9,71
Nphp3	nephronophthisis 3 (adolescent)	ENSMUSG00000032558	41187	9,71
Rfpl3s	RIKEN cDNA 4930563M21 gene	ENSMUSG00000050702	18326	9,71
Cox4i2	cytochrome c oxidase subunit IV isoform 2	ENSMUSG00000009876	10865	9,71
		ENSMUSG00000094973	1236	9,64

Shmt2	serine hydroxymethyltransferase 2 (mitochondrial)	ENSMUSG00000025403	5322	9,64
Cdk20	cyclin-dependent kinase 20	ENSMUSG00000021483	7168	9,64
Me1	malic enzyme 1, NADP(+)-dependent, cytosolic	ENSMUSG00000032418	114552	9,64
Trim13	tripartite motif-containing 13	ENSMUSG00000035235	7721	9,64
Fdxr	ferredoxin reductase	ENSMUSG00000018861	9027	9,64
F8a	factor 8-associated gene A	ENSMUSG00000078317	2490	9,64
Inpp5e	inositol polyphosphate-5-phosphatase E	ENSMUSG00000026925	12955	9,64
Nudt8	nudix (nucleoside diphosphate linked moiety X)-type motif 8	ENSMUSG00000024869	5237	9,64
		ENSMUSG00000022543	10027	9,64
		ENSMUSG00000025962	24042	9,64
Kctd10	potassium channel tetramerisation domain containing 10	ENSMUSG00000001098	16949	9,64
Pmf1	polyamine-modulated factor 1	ENSMUSG00000028066	16174	9,64
Pttg1	pituitary tumor-transforming gene 1	ENSMUSG00000020415	6002	9,64
		ENSMUSG00000063108	27191	9,64
Edem2	ER degradation enhancer, mannosidase alpha-like 2	ENSMUSG00000038312	27799	9,64
		ENSMUSG00000025937	35702	9,64
Elac1	elaC homolog 1 (E. coli)	ENSMUSG00000036941	19442	9,64
Acot12	acyl-CoA thioesterase 12	ENSMUSG00000021620	44637	9,64
		ENSMUSG00000025762	18296	9,64
4930451C15Rik	RIKEN cDNA 4930451C15 gene	ENSMUSG00000022759	16783	9,64
2010107G23Rik	RIKEN cDNA 2010107G23 gene	ENSMUSG00000020083	36266	9,57
Jrk	jerky	ENSMUSG00000046380	7124	9,57
Adamtsl1	ADAMTS-like 1	ENSMUSG00000066113	374471	9,57
Oxsm	3-oxoacyl-ACP synthase, mitochondrial	ENSMUSG00000021786	11153	9,57
		ENSMUSG00000070284	3533	9,57
Ccdc71	coiled-coil domain containing 71	ENSMUSG00000049305	5428	9,57
Yae1d1	Yae1 domain containing 1	ENSMUSG00000075054	6712	9,57
Cela2a	chymotrypsin-like elastase family, member 2A	ENSMUSG00000058579	11199	9,57
Poll	polymerase (DNA directed), lambda	ENSMUSG00000025218	8257	9,57
Irak1bp1	interleukin-1 receptor-associated kinase 1 binding protein 1	ENSMUSG00000032251	17883	9,57
		ENSMUSG00000056679	25107	9,57
Axin2	axin2	ENSMUSG00000000142	30435	9,57
		ENSMUSG00000004996	7421	9,57
Ssbp1	single-stranded DNA binding protein 1	ENSMUSG00000029911	10472	9,57
Klhl20	kelch-like 20	ENSMUSG00000026705	43137	9,57
Clec2g	C-type lectin domain family 2, member g	ENSMUSG00000000248	50327	9,57
Tmem38b	transmembrane protein 38B	ENSMUSG00000028420	35975	9,57
Wipf3	WAS/WASL interacting protein family, member 3	ENSMUSG00000086040	74166	9,57
Kdm5d	lysine (K)-specific demethylase 5D	ENSMUSG00000056673	46024	9,57
Cflar	CASP8 and FADD-like apoptosis regulator	ENSMUSG00000072980	8958	9,57
Ptpru	protein tyrosine phosphatase, receptor type, U	ENSMUSG00000028909	69832	9,57

Pcgf6	polycomb group ring finger 6	ENSMUSG00000025050	17227	9,57
Cndp1	carnosine dipeptidase 1 (metallopeptidase M20 family)	ENSMUSG00000056162	39576	9,57
Oas1a	2'-5' oligoadenylate synthetase 1A	ENSMUSG00000052776	11266	9,57
Lama5	laminin, alpha 5	ENSMUSG00000015647	49487	9,57
		ENSMUSG00000068399	208227	9,57
Itgb7	integrin beta 7	ENSMUSG00000001281	15941	9,57
A730049H05Rik	RIKEN cDNA A730049H05 gene	ENSMUSG00000048636	30697	9,57
Chrm1	cholinergic receptor, muscarinic 1, CNS	ENSMUSG00000032773	19799	9,57
Abhd8	abhydrolase domain containing 8	ENSMUSG00000007950	6958	9,50
Kbtbd8	kelch repeat and BTB (POZ) domain containing 8	ENSMUSG00000030031	12479	9,50
Lig4	ligase IV, DNA, ATP-dependent	ENSMUSG00000049717	7667	9,50
Bet1	blocked early in transport 1 homolog (S. cerevisiae)	ENSMUSG00000032757	10074	9,50
Dgkz	diacylglycerol kinase zeta	ENSMUSG00000040479	43041	9,50
Def6	differentially expressed in FDCP 6	ENSMUSG00000002257	20831	9,50
Mtrf1l	mitochondrial translational release factor 1-like	ENSMUSG00000019774	12024	9,50
Nagpa	N-acetylglucosamine-1-phosphodiester alpha-N-acetylglucosaminidase	ENSMUSG00000023143	8724	9,50
Slain1	SLAIN motif family, member 1	ENSMUSG00000055717	54680	9,50
Rapgefl1	Rap guanine nucleotide exchange factor (GEF)-like 1	ENSMUSG00000038020	16913	9,50
Pnpla6	patatin-like phospholipase domain containing 6	ENSMUSG00000004565	28883	9,50
Gm13139	predicted gene 13139	ENSMUSG00000067916	48542	9,50
Egfem1	EGF-like and EMI domain containing 1	ENSMUSG00000063600	609182	9,50
Nabp1	nucleic acid binding protein 1	ENSMUSG00000026107	8902	9,50
		ENSMUSG00000067158	134292	9,50
Epsti1	epithelial stromal interaction 1 (breast)	ENSMUSG00000022014	98418	9,50
P2ry14	purinergic receptor P2Y, G-protein coupled, 14	ENSMUSG00000036381	15999	9,50
AI848285	expressed sequence AI848285	ENSMUSG00000096883	5864	9,50
Ntrk2	neurotrophic tyrosine kinase, receptor, type 2	ENSMUSG00000055254	327375	9,42
Rbfa	ribosome binding factor A	ENSMUSG00000024570	8394	9,42
1700067K01Rik	RIKEN cDNA 1700067K01 gene	ENSMUSG00000046408	3269	9,42
Dkk3	dickkopf homolog 3 (Xenopus laevis)	ENSMUSG00000030772	43039	9,42
Myl4	myosin, light polypeptide 4	ENSMUSG00000061086	45091	9,42
Slc37a4	solute carrier family 37 (glucose-6-phosphate transporter), member 4	ENSMUSG00000032114	4788	9,42
Pgbd5	piggyBac transposable element derived 5	ENSMUSG00000050751	70610	9,42
Lix1l	Lix1-like	ENSMUSG00000049288	24220	9,42
4931440P22Rik	RIKEN cDNA 4931440P22 gene	ENSMUSG00000074580	22123	9,42
Aim2	absent in melanoma 2	ENSMUSG00000037860	115162	9,42
Ranbp6	RAN binding protein 6	ENSMUSG00000074909	4867	9,42
Dimt1	DIM1 dimethyladenosine transferase 1-like (S. cerevisiae)	ENSMUSG00000021692	13096	9,42

Mbtps2	membrane-bound transcription factor peptidase, site 2	ENSMUSG00000046873	63345	9,42
		ENSMUSG00000067424	16894	9,42
Ube2cbp	ubiquitin-conjugating enzyme E2C binding protein	ENSMUSG00000032415	157717	9,42
Itga3	integrin alpha 3	ENSMUSG00000001507	32328	9,42
		ENSMUSG00000024228	13755	9,42
Atg14	autophagy related 14	ENSMUSG00000037526	27541	9,42
Styxl1	serine/threonine/tyrosine interacting-like 1	ENSMUSG00000019178	31166	9,42
Cmbl	carboxymethylenebutenolidase-like (Pseudomonas)	ENSMUSG00000022235	21329	9,42
6820408C15Rik	RIKEN cDNA 6820408C15 gene	ENSMUSG00000032680	28744	9,42
Nupl2	nucleoporin like 2	ENSMUSG00000048439	19051	9,42
Ppp4r4	protein phosphatase 4, regulatory subunit 4	ENSMUSG00000021209	81268	9,42
Pim2	proviral integration site 2	ENSMUSG00000031155	5171	9,42
Trpc5	transient receptor potential cation channel, subfamily C, member 5	ENSMUSG00000041710	306510	9,42
Dpp4	dipeptidylpeptidase 4	ENSMUSG00000035000	82159	9,42
Abcb5	ATP-binding cassette, sub-family B (MDR/TAP), member 5	ENSMUSG00000072791	98598	9,42
Angptl3	angiopoietin-like 3	ENSMUSG00000028553	15158	9,42
Gm7534	predicted gene 7534	ENSMUSG00000073747	12201	9,35
Ikbip	IKBKB interacting protein	ENSMUSG00000019975	19668	9,35
Ptges	prostaglandin E synthase	ENSMUSG00000050737	40393	9,35
Pigo	phosphatidylinositol glycan anchor biosynthesis, class O	ENSMUSG00000028454	8185	9,35
Naglu	alpha-N-acetylglucosaminidase (Sanfilippo disease IIIB)	ENSMUSG00000001751	7661	9,35
Mfsd9	major facilitator superfamily domain containing 9	ENSMUSG00000041945	18650	9,35
Mfsd2a	major facilitator superfamily domain containing 2A	ENSMUSG00000028655	14339	9,35
Taf6l	TAF6-like RNA polymerase II, p300/CBP-associated factor (PCAF)-associated factor	ENSMUSG00000003680	12064	9,35
Lmod1	leiomodin 1 (smooth muscle)	ENSMUSG00000048096	43259	9,35
Fmnl3	formin-like 3	ENSMUSG00000023008	53258	9,35
Mob3a	MOB kinase activator 3A	ENSMUSG00000003348	16565	9,35
Rpp30	ribonuclease P/MRP 30 subunit	ENSMUSG00000024800	21057	9,35
3110040N11Rik	RIKEN cDNA 3110040N11 gene	ENSMUSG00000025102	7297	9,35
Lin7a	lin-7 homolog A (C. elegans)	ENSMUSG00000019906	153301	9,35
Chl1	cell adhesion molecule with homology to L1CAM	ENSMUSG00000030077	222160	9,35
Gzmm	granzyme M (lymphocyte met-ase 1)	ENSMUSG00000054206	6242	9,35
Zfp385a	zinc finger protein 385A	ENSMUSG00000000552	26192	9,35
Gan	giant axonal neuropathy	ENSMUSG00000052557	47700	9,35
Lrrc39	leucine rich repeat containing 39	ENSMUSG00000027961	20162	9,35
Bmp6	bone morphogenetic protein 6	ENSMUSG00000039004	154013	9,35
		ENSMUSG00000031504	25247	9,35
Rragb	Ras-related GTP binding B	ENSMUSG00000041658	31963	9,35
Pgpep1l	pyroglutamyl-peptidase I-like	ENSMUSG00000030553	27626	9,35
		ENSMUSG00000083193	24642	9,35
		ENSMUSG00000094920	422658	9,35

		ENSMUSG00000051074	652	9,28
Mrm1	mitochondrial rRNA methyltransferase 1 homolog (S. cerevisiae)	ENSMUSG00000018405	6455	9,28
Peo1	progressive external ophthalmoplegia 1 (human)	ENSMUSG00000025209	6205	9,28
Qrsl1	glutaminyl-tRNA synthase (glutamine-hydrolyzing)-like 1	ENSMUSG00000019863	27558	9,28
Nxpe3	neurexophilin and PC-esterase domain family, member 3	ENSMUSG00000075033	55327	9,28
Gins1	GINS complex subunit 1 (Psf1 homolog)	ENSMUSG00000027454	25881	9,28
		ENSMUSG00000024831	22094	9,28
Fgd1	FYVE, RhoGEF and PH domain containing 1	ENSMUSG00000025265	43372	9,28
Abca1	ATP-binding cassette, sub-family A (ABC1), member 1	ENSMUSG00000015243	129109	9,28
Ccdc113	coiled-coil domain containing 113	ENSMUSG00000036598	24789	9,28
F13a1	coagulation factor XIII, A1 subunit	ENSMUSG00000039109	183067	9,28
Gm5901	predicted gene 5901	ENSMUSG00000078611	3190	9,28
Tbc1d31	TBC1 domain family, member 31	ENSMUSG00000022364	57869	9,28
		ENSMUSG00000044676	10418	9,28
Tubd1	tubulin, delta 1	ENSMUSG00000020513	22370	9,28
Efhc1	EF-hand domain (C-terminal) containing 1	ENSMUSG00000041809	39213	9,28
Tnnt1	troponin T1, skeletal, slow	ENSMUSG00000064179	11813	9,28
4933408B17Rik	RIKEN cDNA 4933408B17 gene	ENSMUSG00000049357	17623	9,28
Stab2	stabilin 2	ENSMUSG00000035459	166828	9,28
Ccdc67	coiled-coil domain containing 67	ENSMUSG00000039977	68070	9,28
Srgn	serglycin	ENSMUSG00000020077	33619	9,28
Pde4c	phosphodiesterase 4C, cAMP specific	ENSMUSG00000031842	27467	9,28
Sparc	secreted acidic cysteine rich glycoprotein	ENSMUSG00000018593	25581	9,21
Col4a2	collagen, type IV, alpha 2	ENSMUSG00000031503	136483	9,21
Tefm	transcription elongation factor, mitochondrial	ENSMUSG00000046909	5498	9,21
Fam114a1	family with sequence similarity 114, member A1	ENSMUSG00000029185	71804	9,21
Nrtn	neurturin	ENSMUSG00000039481	6206	9,21
Brsk2	BR serine/threonine kinase 2	ENSMUSG00000053046	54497	9,21
Rps12	ribosomal protein S12	ENSMUSG00000061983	1985	9,21
Endov	endonuclease V	ENSMUSG00000039850	20091	9,21
		ENSMUSG00000041592	290673	9,21
Shkbp1	Sh3kbp1 binding protein 1	ENSMUSG00000089832	13887	9,21
Dnajc30	DnaJ (Hsp40) homolog, subfamily C, member 30	ENSMUSG00000061118	1160	9,21
Zc3h8	zinc finger CCCH type containing 8	ENSMUSG00000027387	17810	9,21
Ap5m1	adaptor-related protein complex 5, mu 1 subunit	ENSMUSG00000036291	21229	9,21
Mrgprx2	MAS-related GPR, member X2	ENSMUSG00000074109	20652	9,21
Mrrf	mitochondrial ribosome recycling factor	ENSMUSG00000026887	54259	9,21
Fam178b	family with sequence similarity 178, member B	ENSMUSG00000046337	120490	9,21
Zfp384	zinc finger protein 384	ENSMUSG00000038346	28726	9,21

Tk1	thymidine kinase 1	ENSMUSG00000025574	10567	9,21
Sag	S-antigen, retina and pineal gland (arrestin)	ENSMUSG00000056055	41479	9,21
Dhtkd1	dehydrogenase E1 and transketolase domain containing 1	ENSMUSG00000025815	46678	9,21
Trpc3	transient receptor potential cation channel, subfamily C, member 3	ENSMUSG00000027716	69686	9,21
		ENSMUSG00000063929	20634	9,21
Mab21l3	mab-21-like 3 (C. elegans)	ENSMUSG00000044313	35884	9,21
Ccdc150	coiled-coil domain containing 150	ENSMUSG00000025983	118045	9,21
Piezo2	piezo-type mechanosensitive ion channel component 2	ENSMUSG00000041482	376971	9,13
Ppp1r35	protein phosphatase 1, regulatory subunit 35	ENSMUSG00000029725	1262	9,13
Cenpw	centromere protein W	ENSMUSG00000075266	4530	9,13
Pus3	pseudouridine synthase 3	ENSMUSG00000032103	8589	9,13
Cdk10	cyclin-dependent kinase 10	ENSMUSG00000033862	7410	9,13
Tapbpl	TAP binding protein-like	ENSMUSG00000038213	7928	9,13
Armcx4	armadillo repeat containing, X-linked 4	ENSMUSG00000049804	10239	9,13
		ENSMUSG00000026027	22194	9,13
Pias3	protein inhibitor of activated STAT 3	ENSMUSG00000028101	9687	9,13
Zfp536	zinc finger protein 536	ENSMUSG00000043456	301507	9,13
Asf1b	ASF1 anti-silencing function 1 homolog B (S. cerevisiae)	ENSMUSG00000005470	14691	9,13
Mmachc	methylmalonic aciduria cblC type, with homocystinuria	ENSMUSG00000028690	6128	9,13
Tmem141	transmembrane protein 141	ENSMUSG00000026939	1939	9,13
Psme2	proteasome (prosome, macropain) activator subunit 2 (PA28 beta)	ENSMUSG00000079197	3673	9,13
Sh3gl1	SH3-domain GRB2-like 1	ENSMUSG00000003200	19885	9,13
Man2c1	mannosidase, alpha, class 2C, member 1	ENSMUSG00000032295	12033	9,13
		ENSMUSG00000041779	82934	9,13
Mbd3l1	methyl-CpG binding domain protein 3-like 1	ENSMUSG00000038691	6914	9,13
Fstl1	follistatin-like 1	ENSMUSG00000022816	59642	9,06
		ENSMUSG00000037493	15425	9,06
Rpusd1	RNA pseudouridylate synthase domain containing 1	ENSMUSG00000041199	3706	9,06
Toe1	target of EGR1, member 1 (nuclear)	ENSMUSG00000028688	13331	9,06
1110008J03Rik	RIKEN cDNA 1110008J03 gene	ENSMUSG00000029600	18285	9,06
2900011O08Rik	RIKEN cDNA 2900011O08 gene	ENSMUSG00000044117	114897	9,06
Anln	anillin, actin binding protein	ENSMUSG00000036777	57993	9,06
S100a16	S100 calcium binding protein A16	ENSMUSG00000074457	5898	9,06
Gm2058	predicted gene 2058	ENSMUSG00000074903	787	9,06
Thop1	thimet oligopeptidase 1	ENSMUSG00000004929	12525	9,06
Tspyl2	TSPY-like 2	ENSMUSG00000041096	5574	9,06
Ap4m1	adaptor-related protein complex AP-4, mu 1	ENSMUSG00000019518	6707	9,06
Atp10a	ATPase, class V, type 10A	ENSMUSG00000025324	171225	9,06
Spag8	sperm associated antigen 8	ENSMUSG00000066196	2260	9,06
Fosl1	fos-like antigen 1	ENSMUSG00000024912	8243	9,06
Xrra1	X-ray radiation resistance associated 1	ENSMUSG00000035211	58607	9,06

Phldb3	pleckstrin homology-like domain, family B, member 3	ENSMUSG00000074277	17961	9,06
Tmem154	transmembrane protein 154	ENSMUSG00000056498	38384	9,06
Ly6a	lymphocyte antigen 6 complex, locus A	ENSMUSG00000075602	2757	9,06
Muc6	mucin 6, gastric	ENSMUSG00000048191	21348	9,06
4930415O20Rik	RIKEN cDNA 4930415O20 gene	ENSMUSG00000022993	18585	9,06
Hp	haptoglobin	ENSMUSG00000031722	4043	9,06
Ryr2	ryanodine receptor 2, cardiac	ENSMUSG00000021313	553843	9,06
1700026D08Rik	RIKEN cDNA 1700026D08 gene	ENSMUSG00000011154	20780	9,06
Ttc9b	tetratricopeptide repeat domain 9B	ENSMUSG00000007944	2294	8,99
Ubxn11	UBX domain protein 11	ENSMUSG00000012126	25024	8,99
Asf1a	ASF1 anti-silencing function 1 homolog A (S. cerevisiae)	ENSMUSG00000019857	12253	8,99
Repin1	replication initiator 1	ENSMUSG00000052751	5200	8,99
Dtwd1	DTW domain containing 1	ENSMUSG00000023330	13139	8,99
Slc52a2	solute carrier protein 52, member 2	ENSMUSG00000022560	3188	8,99
		ENSMUSG00000026694	16425	8,99
		ENSMUSG00000078903	15853	8,99
Zfp760	zinc finger protein 760	ENSMUSG00000067928	17896	8,99
		ENSMUSG00000027669	36254	8,99
Cc2d1a	coiled-coil and C2 domain containing 1A	ENSMUSG00000036686	15109	8,99
Fam122b	family with sequence similarity 122, member B	ENSMUSG00000036022	26391	8,99
Rnf139	ring finger protein 139	ENSMUSG00000037075	13162	8,99
C1galt1	core 1 synthase, glycoprotein-N-acetylgalactosamine 3-beta-galactosyltransferase, 1	ENSMUSG00000042460	27206	8,99
Oca2	oculocutaneous albinism II	ENSMUSG00000030450	296758	8,99
Ptrf	polymerase I and transcript release factor	ENSMUSG00000004044	14155	8,92
Fbxl6	F-box and leucine-rich repeat protein 6	ENSMUSG00000022559	3017	8,92
Smox	spermine oxidase	ENSMUSG00000027333	34427	8,92
Hilpda	hypoxia inducible lipid droplet associated	ENSMUSG00000043421	2959	8,92
Tagln	transgelin	ENSMUSG00000032085	6431	8,92
Rpusd4	RNA pseudouridylate synthase domain containing 4	ENSMUSG00000032044	9867	8,92
Fbxl14	F-box and leucine-rich repeat protein 14	ENSMUSG00000030019	4219	8,92
Zbtb24	zinc finger and BTB domain containing 24	ENSMUSG00000019826	15176	8,92
Gspt2	G1 to S phase transition 2	ENSMUSG00000071723	7176	8,92
Fdx1	ferredoxin 1	ENSMUSG00000032051	20227	8,92
D11Wsu47e	DNA segment, Chr 11, Wayne State University 47, expressed	ENSMUSG00000041623	10236	8,92
Nr1h2	nuclear receptor subfamily 1, group H, member 2	ENSMUSG00000060601	4336	8,92
Ubqln4	ubiquilin 4	ENSMUSG00000008604	16010	8,92
Coro1b	coronin, actin binding protein 1B	ENSMUSG00000024835	5417	8,92
Anapc15	anaphase prompoting complex C subunit 15	ENSMUSG00000030649	20521	8,92
Acap1	ArfGAP with coiled-coil, ankyrin repeat and PH domains 1	ENSMUSG00000001588	13973	8,92

		ENSMUSG00000010175	52643	8,92
Fanca	Fanconi anemia, complementation group A	ENSMUSG00000032815	50277	8,92
Clp1	CLP1, cleavage and polyadenylation factor I subunit	ENSMUSG00000027079	5247	8,92
Camkk1	calcium/calmodulin-dependent protein kinase kinase 1, alpha	ENSMUSG00000020785	23066	8,92
Ctu2	cytosolic thiouridylase subunit 2 homolog (S. pombe)	ENSMUSG00000049482	7996	8,92
Arhgef10	Rho guanine nucleotide exchange factor (GEF) 10	ENSMUSG00000071176	89423	8,92
Bace2	beta-site APP-cleaving enzyme 2	ENSMUSG00000040605	82285	8,92
Tmem173	transmembrane protein 173	ENSMUSG00000024349	6877	8,92
Mtfr2	mitochondrial fission regulator 2	ENSMUSG00000019992	13851	8,92
Lrfn5	leucine rich repeat and fibronectin type III domain containing 5	ENSMUSG00000035653	334779	8,92
Acox2	acyl-Coenzyme A oxidase 2, branched chain	ENSMUSG00000021751	33843	8,92
Bcl3	B cell leukemia/lymphoma 3	ENSMUSG00000053175	14309	8,92
Selenbp2	selenium binding protein 2	ENSMUSG00000068877	10858	8,92
Cdkl1	cyclin-dependent kinase-like 1 (CDC2-related kinase)	ENSMUSG00000020990	43860	8,92
Mpz	myelin protein zero	ENSMUSG00000056569	10420	8,84
Fbln5	fibulin 5	ENSMUSG00000021186	72491	8,84
Zfp275	zinc finger protein 275	ENSMUSG00000031365	16460	8,84
Zfp874b	zinc finger protein 874b	ENSMUSG00000059839	12712	8,84
Ier5l	immediate early response 5-like	ENSMUSG00000089762	1552	8,84
1700017D01Rik	RIKEN cDNA 1700017D01 gene	ENSMUSG00000024729	34063	8,84
Pced1a	PC-esterase domain containing 1A	ENSMUSG00000037773	7455	8,84
Rgmb	RGM domain family, member B	ENSMUSG00000048027	20334	8,84
Has3	hyaluronan synthase 3	ENSMUSG00000031910	14325	8,84
Cdk18	cyclin-dependent kinase 18	ENSMUSG00000026437	26136	8,84
Zfpm2	zinc finger protein, multitype 2	ENSMUSG00000022306	449551	8,84
Nynrin	NYN domain and retroviral integrase containing	ENSMUSG00000075592	20700	8,84
Wdr25	WD repeat domain 25	ENSMUSG00000040877	134181	8,84
		ENSMUSG00000027358	10727	8,84
Trmt10b	tRNA methyltransferase 10B	ENSMUSG00000035601	19005	8,84
		ENSMUSG00000022184	14129	8,84
Zswim7	zinc finger SWIM-type containing 7	ENSMUSG00000014243	14152	8,84
2810459M11Rik	RIKEN cDNA 2810459M11 gene	ENSMUSG00000026227	9594	8,84
Unc5d	unc-5 homolog D (C. elegans)	ENSMUSG00000063626	572622	8,84
Ush2a	Usher syndrome 2A (autosomal recessive, mild)	ENSMUSG00000026609	703019	8,84
Cygb	cytoglobin	ENSMUSG00000020810	8719	8,77
Jkamp	JNK1/MAPK8-associated membrane protein	ENSMUSG00000005078	15873	8,77
Rabl5	RAB, member of RAS oncogene family-like 5	ENSMUSG00000007987	5095	8,77
Ubl7	ubiquitin-like 7 (bone marrow stromal cell-derived)	ENSMUSG00000055720	18983	8,77
Fbxo41	F-box protein 41	ENSMUSG00000047013	33421	8,77
Det1	de-etiolated homolog 1 (Arabidopsis)	ENSMUSG00000030610	24275	8,77
Zdhhc7	zinc finger, DHHC domain containing 7	ENSMUSG00000031823	20388	8,77

Zfp961	zinc finger protein 961	ENSMUSG00000052446	42371	8,77
Rab4b	RAB4B, member RAS oncogene family	ENSMUSG00000053291	10473	8,77
Inf2	inverted formin, FH2 and WH2 domain containing	ENSMUSG00000037679	26773	8,77
Osbpl7	oxysterol binding protein-like 7	ENSMUSG00000038534	18277	8,77
		ENSMUSG00000008496	68014	8,77
Ccdc84	coiled-coil domain containing 84	ENSMUSG00000043923	7844	8,77
Cul7	cullin 7	ENSMUSG00000038545	14028	8,77
		ENSMUSG00000026344	45480	8,77
Abcd4	ATP-binding cassette, sub-family D (ALD), member 4	ENSMUSG00000021240	14936	8,77
AA415398	expressed sequence AA415398	ENSMUSG00000087385	9222	8,77
Tead3	TEA domain family member 3	ENSMUSG00000002249	19135	8,77
Zbtb16	zinc finger and BTB domain containing 16	ENSMUSG00000066687	181635	8,77
Tmem243	transmembrane protein 243, mitochondrial	ENSMUSG00000079659	18247	8,77
Fam19a2	family with sequence similarity 19, member A2	ENSMUSG00000044071	477129	8,77
Niacr1	niacin receptor 1	ENSMUSG00000045502	1947	8,77
		ENSMUSG00000095162	54951	8,77
Spata21	spermatogenesis associated 21	ENSMUSG00000045004	24428	8,77
BC030476	cDNA sequence BC030476	ENSMUSG00000044726	20581	8,77
Srxn1	sulfiredoxin 1 homolog (S. cerevisiae)	ENSMUSG00000032802	5861	8,70
Aldh5a1	aldhehyde dehydrogenase family 5, subfamily A1	ENSMUSG00000035936	30083	8,70
Gyg	glycogenin	ENSMUSG00000019528	33234	8,70
Trmt12	tRNA methyltranferase 12	ENSMUSG00000037085	4133	8,70
		ENSMUSG00000036873	6816	8,70
		ENSMUSG00000046463	2122	8,70
Mak16	MAK16 homolog (S. cerevisiae)	ENSMUSG00000031578	9257	8,70
Zfp551	zinc fingr protein 551	ENSMUSG00000034071	7599	8,70
		ENSMUSG00000095253	6746	8,70
Suv39h1	suppressor of variegation 3-9 homolog 1 (Drosophila)	ENSMUSG00000039231	13590	8,70
Mybpc1	myosin binding protein C, slow-type	ENSMUSG00000020061	86874	8,70
Plk5	polo-like kinase 5	ENSMUSG00000035486	9031	8,70
BC053749	cDNA sequence BC053749	ENSMUSG00000036864	13163	8,70
Kdm4d	lysine (K)-specific demethylase 4D	ENSMUSG00000053914	37935	8,70
Tgm4	transglutaminase 4 (prostate)	ENSMUSG00000025787	32823	8,70
		ENSMUSG00000031465	51300	8,70
Zdbf2	zinc finger, DBF-type containing 2	ENSMUSG00000027520	41312	8,70
Trf	transferrin	ENSMUSG00000032554	26444	8,70
Ttn	titin	ENSMUSG00000051747	278568	8,70
A630023A22Rik	RIKEN cDNA A630023A22 gene	ENSMUSG00000095493	51623	8,70
Htr1f	5-hydroxytryptamine (serotonin) receptor 1F	ENSMUSG00000050783	181056	8,70
Plod1	procollagen-lysine, 2-oxoglutarate 5-dioxygenase 1	ENSMUSG00000019055	27015	8,63
Rad9a	RAD9 homolog A	ENSMUSG00000024824	6406	8,63
2810408M09Rik	RIKEN cDNA 2810408M09 gene	ENSMUSG00000039725	3215	8,63
Mrps22	mitochondrial ribosomal protein S22	ENSMUSG00000032459	12947	8,63

Traf3ip3	TRAF3 interacting protein 3	ENSMUSG00000037318	26198	8,63
Srrd	SRR1 domain containing	ENSMUSG00000029346	5649	8,63
Zfp784	zinc finger protein 784	ENSMUSG00000043290	4008	8,63
Iffo1	intermediate filament family orphan 1	ENSMUSG00000038271	16542	8,63
		ENSMUSG00000095538	12657	8,63
Ercc1	excision repair cross-complementing rodent repair deficiency, complementation group 1	ENSMUSG00000003549	11747	8,63
Akr1b10	aldo-keto reductase family 1, member B10 (aldose reductase)	ENSMUSG00000061758	12733	8,63
Gins3	GINS complex subunit 3 (Psf3 homolog)	ENSMUSG00000031669	11501	8,63
Ano8	anoctamin 8	ENSMUSG00000034863	10045	8,63
		ENSMUSG00000074501	1264	8,63
Nme4	NME/NM23 nucleoside diphosphate kinase 4	ENSMUSG00000024177	3775	8,63
Disp2	dispatched homolog 2 (Drosophila)	ENSMUSG00000040035	31575	8,63
Uros	uroporphyrinogen III synthase	ENSMUSG00000030979	23830	8,63
Gm1661	predicted gene 1661	ENSMUSG00000060268	38793	8,63
Ccdc163	coiled-coil domain containing 163	ENSMUSG00000028689	42698	8,63
Acsf2	acyl-CoA synthetase family member 2	ENSMUSG00000076435	44770	8,63
Mcm8	minichromosome maintenance deficient 8 (S. cerevisiae)	ENSMUSG00000027353	28057	8,63
Klhl3	kelch-like 3	ENSMUSG00000014164	109372	8,63
Arhgap27	Rho GTPase activating protein 27	ENSMUSG00000034255	32196	8,63
Klhl1	kelch-like 1	ENSMUSG00000022076	413844	8,63
Mettl24	methyltransferase like 24	ENSMUSG00000045555	127802	8,63
Emr1	EGF-like module containing, mucin-like, hormone receptor-like sequence 1	ENSMUSG00000004730	124844	8,63
Serpinf1	serine (or cysteine) peptidase inhibitor, clade F, member 1	ENSMUSG00000000753	12933	8,55
6430548M08Rik	RIKEN cDNA 6430548M08 gene	ENSMUSG00000031824	51155	8,55
Foxi1	forkhead box I1	ENSMUSG00000047861	3752	8,55
		ENSMUSG00000019303	3614	8,55
Isg20l2	interferon stimulated exonuclease gene 20-like 2	ENSMUSG00000048039	10373	8,55
Mpp3	membrane protein, palmitoylated 3 (MAGUK p55 subfamily member 3)	ENSMUSG00000052373	28810	8,55
Gstp1	glutathione S-transferase, pi 1	ENSMUSG00000060803	2502	8,55
Col27a1	collagen, type XXVII, alpha 1	ENSMUSG00000045672	120988	8,55
Sult1a1	sulfotransferase family 1A, phenol-preferring, member 1	ENSMUSG00000030711	3568	8,55
Rarres2	retinoic acid receptor responder (tazarotene induced) 2	ENSMUSG00000009281	2973	8,55
Dxo	decapping exoribonuclease	ENSMUSG00000040482	2215	8,55
Zfp574	zinc finger protein 574	ENSMUSG00000045252	6288	8,55
Eif2d	eukaryotic translation initiation factor 2D	ENSMUSG00000026427	34478	8,55
Fam71e1	family with sequence similarity 71, member E1	ENSMUSG00000051113	4547	8,55
Rad54b	RAD54 homolog B (S. cerevisiae)	ENSMUSG00000078773	56886	8,55
		ENSMUSG00000006576	15907	8,55

Pdzk1	PDZ domain containing 1	ENSMUSG00000038298	41643	8,55
Slc38a7	solute carrier family 38, member 7	ENSMUSG00000036534	17620	8,55
		ENSMUSG00000022965	18098	8,55
		ENSMUSG00000093862	2710	8,55
Gm7120	predicted gene 7120	ENSMUSG00000074634	122518	8,55
Raver1	ribonucleoprotein, PTB-binding 1	ENSMUSG00000010205	17821	8,55
Elmo3	engulfment and cell motility 3	ENSMUSG00000014791	5023	8,55
		ENSMUSG00000032443	138161	8,55
Htr2c	5-hydroxytryptamine (serotonin) receptor 2C	ENSMUSG00000041380	234765	8,55
1700003E16Rik	RIKEN cDNA 1700003E16 gene	ENSMUSG00000030030	6572	8,48
Serp2	stress-associated endoplasmic reticulum protein family member 2	ENSMUSG00000052584	23874	8,48
Ece2	endothelin converting enzyme 2	ENSMUSG00000022842	34893	8,48
Nlgn2	neuroligin 2	ENSMUSG00000051790	14663	8,48
Kifc1	kinesin family member C1	ENSMUSG00000079553	14996	8,48
Zbtb34	zinc finger and BTB domain containing 34	ENSMUSG00000068966	25217	8,48
Tex21	testis expressed gene 21	ENSMUSG00000021056	48055	8,48
Dclre1b	DNA cross-link repair 1B, PSO2 homolog (S. cerevisiae)	ENSMUSG00000027845	8840	8,48
Hs3st6	heparan sulfate (glucosamine) 3-O-sulfotransferase 6	ENSMUSG00000039628	5681	8,48
Setd6	SET domain containing 6	ENSMUSG00000031671	3096	8,48
Slco2a1	solute carrier organic anion transporter family, member 2a1	ENSMUSG00000032548	79361	8,48
Fcf1	FCF1 small subunit (SSU) processome component homolog (S. cerevisiae)	ENSMUSG00000021243	12407	8,48
Tbx20	T-box 20	ENSMUSG00000031965	53492	8,48
Zfp948	zinc finger protein 948	ENSMUSG00000067931	21652	8,48
Trap1	TNF receptor-associated protein 1	ENSMUSG00000005981	37857	8,48
Cdca7l	cell division cycle associated 7 like	ENSMUSG00000021175	74418	8,48
Bnipl	BCL2/adenovirus E1B 19kD interacting protein like	ENSMUSG00000028115	9923	8,48
Gpr171	G protein-coupled receptor 171	ENSMUSG00000050075	5374	8,48
Nlrp9c	NLR family, pyrin domain containing 9C	ENSMUSG00000040614	81228	8,48
Reep4	receptor accessory protein 4	ENSMUSG00000033589	3683	8,41
Dak	dihydroxyacetone kinase 2 homolog (yeast)	ENSMUSG00000034371	12059	8,41
S100z	S100 calcium binding protein, zeta	ENSMUSG00000021679	1355	8,41
Plekho2	pleckstrin homology domain containing, family O member 2	ENSMUSG00000050721	25655	8,41
Efs	embryonal Fyn-associated substrate	ENSMUSG00000022203	10246	8,41
		ENSMUSG00000040688	9712	8,41
Pnpla2	patatin-like phospholipase domain containing 2	ENSMUSG00000025509	5546	8,41
Haus7	HAUS augmin-like complex, subunit 7	ENSMUSG00000031371	21730	8,41
3110062M04Rik	RIKEN cDNA 3110062M04 gene	ENSMUSG00000046806	6290	8,41
Il4	interleukin 4	ENSMUSG00000000869	15688	8,41
Tmem140	transmembrane protein 140	ENSMUSG00000057137	11801	8,41
Ndufa3	NADH dehydrogenase (ubiquinone) 1 alpha subcomplex, 3	ENSMUSG00000035674	2791	8,41

Clvs1	clavesin 1	ENSMUSG00000041216	182399	8,41
Brinp3	bone morphogenetic protein/retinoic acid inducible neural specific 3	ENSMUSG00000035131	406844	8,41
Mroh2a	maestro heat-like repeat family member 2A	ENSMUSG00000079429	35304	8,41
Mmp19	matrix metallopeptidase 19	ENSMUSG00000025355	9872	8,41
Med20	mediator complex subunit 20	ENSMUSG00000092558	12837	8,41
		ENSMUSG00000063652	30368	8,41
Nup54	nucleoporin 54	ENSMUSG00000034826	19680	8,41
Adamtsl4	ADAMTS-like 4	ENSMUSG00000015850	11717	8,41
Lepr	leptin receptor	ENSMUSG00000057722	97949	8,41
Gm10704	predicted pseudogene 10704	ENSMUSG00000074479	964	8,34
Ogfod3	2-oxoglutarate and iron-dependent oxygenase domain containing 3	ENSMUSG00000025169	27121	8,34
Ror2	receptor tyrosine kinase-like orphan receptor 2	ENSMUSG00000021464	176813	8,34
Zswim1	zinc finger SWIM-type containing 1	ENSMUSG00000017764	4186	8,34
		ENSMUSG00000096364	77643	8,34
1700023E05Rik	RIKEN cDNA 1700023E05 gene	ENSMUSG00000029248	45507	8,34
Galnt12	UDP-N-acetyl-alpha-D-galactosamine:polypeptide N-acetylgalactosaminyltransferase 12	ENSMUSG00000039774	31162	8,34
Fam166a	family with sequence similarity 166, member A	ENSMUSG00000026969	3536	8,34
1110007C09Rik	RIKEN cDNA 1110007C09 gene	ENSMUSG00000037960	13076	8,34
		ENSMUSG00000073987	24699	8,34
Pfkfb4	6-phosphofructo-2-kinase/fructose-2,6-biphosphatase 4	ENSMUSG00000025648	40322	8,34
Akap5	A kinase (PRKA) anchor protein 5	ENSMUSG00000021057	9263	8,34
Rai2	retinoic acid induced 2	ENSMUSG00000043518	62428	8,34
Zfp248	zinc finger protein 248	ENSMUSG00000030145	28210	8,34
Pnkp	polynucleotide kinase 3'- phosphatase	ENSMUSG00000002963	5787	8,34
Tgfb3	transforming growth factor, beta 3	ENSMUSG00000021253	22298	8,34
Lgi1	leucine-rich repeat LGI family, member 1	ENSMUSG00000067242	44158	8,34
Ddx11	DEAD/H (Asp-Glu-Ala-Asp/His) box helicase 11	ENSMUSG00000035842	28648	8,34
Calr3	calreticulin 3	ENSMUSG00000019732	19695	8,34
Tmem97	transmembrane protein 97	ENSMUSG00000037278	8961	8,34
Stxbp2	syntaxin binding protein 2	ENSMUSG00000004626	12690	8,34
Dlec1	deleted in lung and esophageal cancer 1	ENSMUSG00000038060	45769	8,34
Gm6086	predicted gene 6086	ENSMUSG00000073608	21033	8,34
2900092C05Rik	RIKEN cDNA 2900092C05 gene	ENSMUSG00000030385	43807	8,34
Ccdc178	coiled coil domain containing 178	ENSMUSG00000024306	360500	8,34
Akap14	A kinase (PRKA) anchor protein 14	ENSMUSG00000036551	18145	8,34
Slc18a1	solute carrier family 18 (vesicular monoamine), member 1	ENSMUSG00000036330	51525	8,34
Serping1	serine (or cysteine) peptidase inhibitor, clade G, member 1	ENSMUSG00000023224	10058	8,26
BC022687	cDNA sequence BC022687	ENSMUSG00000037594	7271	8,26
Jph3	junctophilin 3	ENSMUSG00000025318	64654	8,26
Ppif	peptidylprolyl isomerase F (cyclophilin F)	ENSMUSG00000021868	6315	8,26

Abhd14a	abhydrolase domain containing 14A	ENSMUSG00000042210	7628	8,26
Mvd	mevalonate (diphospho) decarboxylase	ENSMUSG00000006517	9827	8,26
Ttc30b	tetratricopeptide repeat domain 30B	ENSMUSG00000075273	2600	8,26
Gpr137	G protein-coupled receptor 137	ENSMUSG00000024958	4382	8,26
Isca2	iron-sulfur cluster assembly 2 homolog (S. cerevisiae)	ENSMUSG00000021241	1820	8,26
BC068157	cDNA sequence BC068157	ENSMUSG00000064125	7917	8,26
Dtd2	D-tyrosyl-tRNA deacylase 2	ENSMUSG00000020956	18190	8,26
Klhdc4	kelch domain containing 4	ENSMUSG00000040263	33257	8,26
Cenpt	centromere protein T	ENSMUSG00000036672	7331	8,26
Tmem106a	transmembrane protein 106A	ENSMUSG00000034947	9547	8,26
Tmem200a	transmembrane protein 200A	ENSMUSG00000049420	87867	8,26
5730508B09Rik	RIKEN cDNA 5730508B09 gene	ENSMUSG00000050549	26636	8,26
Ngef	neuronal guanine nucleotide exchange factor	ENSMUSG00000026259	97037	8,26
Acad10	acyl-Coenzyme A dehydrogenase family, member 10	ENSMUSG00000029456	39489	8,26
Emilin2	elastin microfibril interfacer 2	ENSMUSG00000024053	58790	8,26
		ENSMUSG00000091908	56431	8,26
Ralyl	RALY RNA binding protein-like	ENSMUSG00000039717	235916	8,26
Casc1	cancer susceptibility candidate 1	ENSMUSG00000043541	36137	8,26
Oas1g	2'-5' oligoadenylate synthetase 1G	ENSMUSG00000066861	11472	8,26
Tmem126a	transmembrane protein 126A	ENSMUSG00000030615	6530	8,19
Ankdd1b	ankyrin repeat and death domain containing 1B	ENSMUSG00000047117	55065	8,19
Acaa1b	acetyl-Coenzyme A acyltransferase 1B	ENSMUSG00000010651	9071	8,19
Dennd6b	DENN/MADD domain containing 6B	ENSMUSG00000015377	14258	8,19
Fam175b	family with sequence similarity 175, member B	ENSMUSG00000030965	25890	8,19
Katnb1	katanin p80 (WD40-containing) subunit B 1	ENSMUSG00000031787	18674	8,19
Srd5a3	steroid 5 alpha-reductase 3	ENSMUSG00000029233	15234	8,19
Extl2	exostoses (multiple)-like 2	ENSMUSG00000027963	21555	8,19
Rps6ka4	ribosomal protein S6 kinase, polypeptide 4	ENSMUSG00000024952	11517	8,19
Kbtbd3	kelch repeat and BTB (POZ) domain containing 3	ENSMUSG00000025893	21979	8,19
Rhof	ras homolog gene family, member f	ENSMUSG00000029449	29649	8,19
Ccne2	cyclin E2	ENSMUSG00000028212	13429	8,19
LOC101055764	transmembrane protein C5orf28 homolog	ENSMUSG00000094114	3809	8,19
Atp5s	ATP synthase, H+ transporting, mitochondrial F0 complex, subunit s	ENSMUSG00000054894	19697	8,19
Vpreb3	pre-B lymphocyte gene 3	ENSMUSG00000000903	6601	8,19
Rnft1	ring finger protein, transmembrane 1	ENSMUSG00000020521	14369	8,19
Ntng1	netrin G1	ENSMUSG00000059857	363189	8,19
		ENSMUSG00000005364	33135	8,19
Cyp2j8	cytochrome P450, family 2, subfamily j, polypeptide 8	ENSMUSG00000082932	62791	8,19

Tktl1	transketolase-like 1	ENSMUSG00000031397	31242	8,19
Tpgs1	tubulin polyglutamylase complex subunit 1	ENSMUSG00000020308	6717	8,12
Aprt	adenine phosphoribosyl transferase	ENSMUSG00000006589	2271	8,12
Haus1	HAUS augmin-like complex, subunit 1	ENSMUSG00000041840	10214	8,12
Tbc1d25	TBC1 domain family, member 25	ENSMUSG00000039201	21710	8,12
Gnai1	guanine nucleotide binding protein (G protein), alpha inhibiting 1	ENSMUSG00000057614	95279	8,12
Vegfb	vascular endothelial growth factor B	ENSMUSG00000024962	5179	8,12
Hebp2	heme binding protein 2	ENSMUSG00000019853	5954	8,12
Pbx4	pre B cell leukemia homeobox 4	ENSMUSG00000031860	40540	8,12
Mthfsd	methenyltetrahydrofolate synthetase domain containing	ENSMUSG00000031816	13381	8,12
Adck5	aarF domain containing kinase 5	ENSMUSG00000022550	19459	8,12
Ddx51	DEAD (Asp-Glu-Ala-Asp) box polypeptide 51	ENSMUSG00000029504	7046	8,12
Gm4793	predicted gene 4793	ENSMUSG00000054412	15178	8,12
Txnrd2	thioredoxin reductase 2	ENSMUSG00000075704	52657	8,12
Gtpbp10	GTP-binding protein 10 (putative)	ENSMUSG00000040464	22085	8,12
		ENSMUSG00000091020	1869	8,12
Hps3	Hermansky-Pudlak syndrome 3 homolog (human)	ENSMUSG00000027615	39371	8,12
		ENSMUSG00000071724	2463	8,12
Gng7	guanine nucleotide binding protein (G protein), gamma 7	ENSMUSG00000048240	66322	8,12
Nmrk1	nicotinamide riboside kinase 1	ENSMUSG00000037847	20245	8,12
Heatr1	HEAT repeat containing 1	ENSMUSG00000050244	43280	8,12
Smc1b	structural maintenance of chromosomes 1B	ENSMUSG00000022432	67267	8,12
		ENSMUSG00000045231	4713	8,12
Helb	helicase (DNA) B	ENSMUSG00000020228	29380	8,12
Adal	adenosine deaminase-like	ENSMUSG00000027259	16253	8,12
Whrn	whirlin	ENSMUSG00000039137	81082	8,12
Kcnip2	Kv channel-interacting protein 2	ENSMUSG00000025221	24223	8,12
Srsf12	serine/arginine-rich splicing factor 12	ENSMUSG00000054679	24350	8,12
		ENSMUSG00000041889	4181	8,05
Hps6	Hermansky-Pudlak syndrome 6	ENSMUSG00000074811	2696	8,05
		ENSMUSG00000018999	23732	8,05
Fam73b	family with sequence similarity 73, member B	ENSMUSG00000026858	21289	8,05
Pif1	PIF1 5'-to-3' DNA helicase homolog (S. cerevisiae)	ENSMUSG00000041064	8808	8,05
1700030J22Rik	RIKEN cDNA 1700030J22 gene	ENSMUSG00000031847	9345	8,05
Gpr45	G protein-coupled receptor 45	ENSMUSG00000041907	82578	8,05
Gcnt7	glucosaminyl (N-acetyl) transferase family member 7	ENSMUSG00000074569	8284	8,05
Chpf2	chondroitin polymerizing factor 2	ENSMUSG00000038181	7655	8,05
AI837181	expressed sequence AI837181	ENSMUSG00000047423	2157	8,05
		ENSMUSG00000020544	6759	8,05
		ENSMUSG00000097771	5417	8,05
Gabrp	gamma-aminobutyric acid (GABA) A receptor, pi	ENSMUSG00000020159	28179	8,05
Abat	4-aminobutyrate aminotransferase	ENSMUSG00000057880	108140	8,05
Dock10	dedicator of cytokinesis 10	ENSMUSG00000038608	257481	8,05

Smim22	small integral membrane protein 22	ENSMUSG00000096215	991	8,05
Yif1b	Yip1 interacting factor homolog B (S. cerevisiae)	ENSMUSG00000030588	9265	8,05
Pigh	phosphatidylinositol glycan anchor biosynthesis, class H	ENSMUSG00000021120	8998	8,05
Gm3417	predicted gene 3417	ENSMUSG00000036648	14624	8,05
Stk32b	serine/threonine kinase 32B	ENSMUSG00000029123	270326	8,05
Cnnm1	cyclin M1	ENSMUSG00000025189	56777	8,05
Klhl17	kelch-like 17	ENSMUSG00000078484	5814	8,05
Gbp8	guanylate-binding protein 8	ENSMUSG00000034438	125388	8,05
		ENSMUSG00000052565	3821	8,05
1700013F07Rik	RIKEN cDNA 1700013F07 gene	ENSMUSG00000027886	10574	8,05
Hyls1	hydrolethalus syndrome 1	ENSMUSG00000050555	9284	7,97
Dolk	dolichol kinase	ENSMUSG00000075419	2126	7,97
Fyb	FYN binding protein	ENSMUSG00000022148	83443	7,97
		ENSMUSG00000061482	393	7,97
Cdc6	cell division cycle 6	ENSMUSG00000017499	16140	7,97
Dyrk3	dual-specificity tyrosine-(Y)-phosphorylation regulated kinase 3	ENSMUSG00000016526	9794	7,97
Optn	optineurin	ENSMUSG00000026672	43410	7,97
Krtcap3	keratinocyte associated protein 3	ENSMUSG00000029149	1512	7,97
		ENSMUSG00000073617	2392	7,97
Tmem29	transmembrane protein 29	ENSMUSG00000090483	32322	7,97
4930519G04Rik	RIKEN cDNA 4930519G04 gene	ENSMUSG00000029564	29996	7,97
Trim32	tripartite motif-containing 32	ENSMUSG00000051675	11253	7,97
Wdhd1	WD repeat and HMG-box DNA binding protein 1	ENSMUSG00000037572	35889	7,97
Ccdc94	coiled-coil domain containing 94	ENSMUSG00000003208	8763	7,97
Rhbdl3	rhomboid, veinlet-like 3 (Drosophila)	ENSMUSG00000017692	55044	7,97
Trcg1	taste receptor cell gene 1	ENSMUSG00000070298	13308	7,97
Shisa3	shisa homolog 3 (Xenopus laevis)	ENSMUSG00000050010	15670	7,97
Gchfr	GTP cyclohydrolase I feedback regulator	ENSMUSG00000046814	4618	7,97
Fbp1	fructose bisphosphatase 1	ENSMUSG00000069805	23530	7,97
		ENSMUSG00000074156	11834	7,97
A630010A05Rik	RIKEN cDNA A630010A05 gene	ENSMUSG00000075395	58985	7,97
Flt3l	FMS-like tyrosine kinase 3 ligand	ENSMUSG00000089989	5245	7,97
Fam71d	family with sequence similarity 71, member D	ENSMUSG00000056987	42979	7,97
Fn1	fibronectin 1	ENSMUSG00000026193	67649	7,90
Ddit4	DNA-damage-inducible transcript 4	ENSMUSG00000020108	2096	7,90
Plk1	polo-like kinase 1	ENSMUSG00000030867	10439	7,90
Slc12a4	solute carrier family 12, member 4	ENSMUSG00000017765	22526	7,90
Pacrgl	PARK2 co-regulated-like	ENSMUSG00000029089	16365	7,90
		ENSMUSG00000097909	6406	7,90
		ENSMUSG00000098004	39538	7,90
Zcchc18	zinc finger, CCHC domain containing 18	ENSMUSG00000031428	5318	7,90
		ENSMUSG00000058447	34377	7,90
Ruvbl2	RuvB-like protein 2	ENSMUSG00000003868	16245	7,90
Mgme1	mitochondrial genome maintainance exonuclease 1	ENSMUSG00000027424	10565	7,90
Pros1	protein S (alpha)	ENSMUSG00000022912	75040	7,90
Kifc2	kinesin family member C2	ENSMUSG00000004187	7556	7,90

Trappc2l	trafficking protein particle complex 2-like	ENSMUSG00000015013	3963	7,90
Ech1	enoyl coenzyme A hydratase 1, peroxisomal	ENSMUSG00000053898	7031	7,90
Ptprz1	protein tyrosine phosphatase, receptor type Z, polypeptide 1	ENSMUSG00000068748	177415	7,90
Zswim3	zinc finger SWIM-type containing 3	ENSMUSG00000045822	17033	7,90
Elf4	E74-like factor 4 (ets domain transcription factor)	ENSMUSG00000031103	52087	7,90
Mfsd7c	major facilitator superfamily domain containing 7C	ENSMUSG00000034258	67047	7,90
Trpm1	transient receptor potential cation channel, subfamily M, member 1	ENSMUSG00000030523	115941	7,90
Tekt4	tektin 4	ENSMUSG00000024175	5005	7,90
6430531B16Rik	RIKEN cDNA 6430531B16 gene	ENSMUSG00000073795	6453	7,90
Cacna2d3	calcium channel, voltage-dependent, alpha2/delta subunit 3	ENSMUSG00000021991	816918	7,90
Abca14	ATP-binding cassette, sub-family A (ABC1), member 14	ENSMUSG00000062017	121392	7,90
Adamts20	a disintegrin-like and metallopeptidase (reprolysin type) with thrombospondin type 1 motif, 20	ENSMUSG00000022449	195256	7,90
		ENSMUSG00000004988	3607	7,90
		ENSMUSG00000067608	1257	7,83
Zfp286	zinc finger protein 286	ENSMUSG00000047342	36086	7,83
Cnpy4	canopy 4 homolog (zebrafish)	ENSMUSG00000036968	6434	7,83
Fam132b	family with sequence similarity 132, member B	ENSMUSG00000047443	7787	7,83
2610524H06Rik	RIKEN cDNA 2610524H06 gene	ENSMUSG00000092486	1534	7,83
Lrrc61	leucine rich repeat containing 61	ENSMUSG00000073096	15927	7,83
Gldc	glycine decarboxylase	ENSMUSG00000024827	76970	7,83
		ENSMUSG00000048747	209946	7,83
Tlcd1	TLC domain containing 1	ENSMUSG00000019437	5199	7,83
Flywch2	FLYWCH family member 2	ENSMUSG00000023911	9166	7,83
Efna2	ephrin A2	ENSMUSG00000003070	10529	7,83
Bcl10	B cell leukemia/lymphoma 10	ENSMUSG00000028191	9906	7,83
Ccdc137	coiled-coil domain containing 137	ENSMUSG00000049957	6244	7,83
Srd5a1	steroid 5 alpha-reductase 1	ENSMUSG00000021594	37994	7,83
Polr1b	polymerase (RNA) I polypeptide B	ENSMUSG00000027395	25600	7,83
Gab3	growth factor receptor bound protein 2-associated protein 3	ENSMUSG00000032750	118616	7,83
Rtkn2	rhotekin 2	ENSMUSG00000037846	80171	7,83
4930404A10Rik	RIKEN cDNA 4930404A10 gene	ENSMUSG00000020332	56139	7,83
Camk1g	calcium/calmodulin-dependent protein kinase I gamma	ENSMUSG00000016179	23952	7,83
Ccdc33	coiled-coil domain containing 33	ENSMUSG00000037716	90147	7,83
Vps9d1	VPS9 domain containing 1	ENSMUSG00000001062	11993	7,83
Hps1	Hermansky-Pudlak syndrome 1 homolog (human)	ENSMUSG00000025188	24874	7,83
		ENSMUSG00000068860	4679	7,83
Prr18	proline rich region 18	ENSMUSG00000055945	3707	7,83
Il6ra	interleukin 6 receptor, alpha	ENSMUSG00000027947	43839	7,83
Pzp	pregnancy zone protein	ENSMUSG00000030359	43154	7,83
Trim12c	tripartite motif-containing 12C	ENSMUSG00000057143	14609	7,83

Aldh3a1	aldehyde dehydrogenase family 3, subfamily A1	ENSMUSG00000019102	10885	7,83
2410016O06Rik	RIKEN cDNA 2410016O06 gene	ENSMUSG00000046791	2346	7,76
Gm9755	predicted pseudogene 9755	ENSMUSG00000030735	1607	7,76
		ENSMUSG00000036061	4696	7,76
Lmtk3	lemur tyrosine kinase 3	ENSMUSG00000062044	20407	7,76
		ENSMUSG00000063757	1129	7,76
Prrg3	proline rich Gla (G-carboxyglutamic acid) 3 (transmembrane)	ENSMUSG00000033361	10099	7,76
Gal3st4	galactose-3-O-sulfotransferase 4	ENSMUSG00000075593	7920	7,76
Mrc1	mannose receptor, C type 1	ENSMUSG00000026712	102644	7,76
Nek2	NIMA (never in mitosis gene a)-related expressed kinase 2	ENSMUSG00000026622	11607	7,76
Lrfn4	leucine rich repeat and fibronectin type III domain containing 4	ENSMUSG00000045045	3883	7,76
D6Wsu163e	DNA segment, Chr 6, Wayne State University 163, expressed	ENSMUSG00000030347	35739	7,76
Atxn1l	ataxin 1-like	ENSMUSG00000069895	11289	7,76
Zfp692	zinc finger protein 692	ENSMUSG00000037243	7559	7,76
Npr3	natriuretic peptide receptor 3	ENSMUSG00000022206	65779	7,76
Pex5	peroxisomal biogenesis factor 5	ENSMUSG00000005069	18252	7,76
		ENSMUSG00000046178	300602	7,76
Zfp750	zinc finger protein 750	ENSMUSG00000039238	8356	7,76
Zbtb48	zinc finger and BTB domain containing 48	ENSMUSG00000028952	7898	7,76
Afmid	arylformamidase	ENSMUSG00000017718	13985	7,76
Il16	interleukin 16	ENSMUSG00000001741	102879	7,76
Dydc1	DPY30 domain containing 1	ENSMUSG00000021790	19283	7,76
Vmn2r7	vomeronasal 2, receptor 7	ENSMUSG00000062200	154106	7,76
Slc6a15	solute carrier family 6 (neurotransmitter transporter), member 15	ENSMUSG00000019894	51571	7,76
		ENSMUSG00000051076	70221	7,76
Slc6a20b	solute carrier family 6 (neurotransmitter transporter), member 20B	ENSMUSG00000025243	38746	7,76
Rrm2	ribonucleotide reductase M2	ENSMUSG00000020649	5906	7,68
Bex1	brain expressed gene 1	ENSMUSG00000050071	1542	7,68
Fam89b	family with sequence similarity 89, member B	ENSMUSG00000024939	1567	7,68
Pdcd2	programmed cell death 2	ENSMUSG00000014771	8094	7,68
Morc4	microrchidia 4	ENSMUSG00000031434	50046	7,68
4933415A04Rik	RIKEN cDNA 4933415A04 gene	ENSMUSG00000045877	2009	7,68
Hopx	HOP homeobox	ENSMUSG00000059325	28719	7,68
Pop1	processing of precursor 1, ribonuclease P/MRP family, (S. cerevisiae)	ENSMUSG00000022325	35345	7,68
Gdpgp1	GDP-D-glucose phosphorylase 1	ENSMUSG00000050973	8140	7,68
		ENSMUSG00000030929	25380	7,68
		ENSMUSG00000059974	968392	7,68
Arhgef19	Rho guanine nucleotide exchange factor (GEF) 19	ENSMUSG00000028919	17990	7,68
Sarm1	sterile alpha and HEAT/Armadillo motif containing 1	ENSMUSG00000050132	25425	7,68
Rfesd	Rieske (Fe-S) domain containing	ENSMUSG00000043190	17178	7,68
9030612E09Rik	RIKEN cDNA 9030612E09 gene	ENSMUSG00000045008	1859	7,68

Ccdc24	coiled-coil domain containing 24	ENSMUSG00000078588	6034	7,68
Ccdc14	coiled-coil domain containing 14	ENSMUSG00000022833	34579	7,68
Cklf	chemokine-like factor	ENSMUSG00000054400	14034	7,68
Hacl1	2-hydroxyacyl-CoA lyase 1	ENSMUSG00000021884	88382	7,68
Ccdc120	coiled-coil domain containing 120	ENSMUSG00000031150	19192	7,68
Tctn1	tectonic family member 1	ENSMUSG00000038593	23174	7,68
Siah3	seven in absentia homolog 3 (Drosophila)	ENSMUSG00000091722	70160	7,68
		ENSMUSG00000073079	23690	7,68
Apol8	apolipoprotein L 8	ENSMUSG00000056656	7431	7,68
Il1b	interleukin 1 beta	ENSMUSG00000027398	6570	7,68
		ENSMUSG00000066487	882	7,61
		ENSMUSG00000050856	1205	7,61
Slit3	slit homolog 3 (Drosophila)	ENSMUSG00000056427	587284	7,61
Mcm2	minichromosome maintenance deficient 2 mitotin (S. cerevisiae)	ENSMUSG00000002870	15306	7,61
Dsn1	DSN1, MIND kinetochore complex component, homolog (S. cerevisiae)	ENSMUSG00000027635	11890	7,61
		ENSMUSG00000070520	5946	7,61
Hs1bp3	HCLS1 binding protein 3	ENSMUSG00000020605	30392	7,61
Zfp420	zinc finger protein 420	ENSMUSG00000058402	17314	7,61
Sorcs2	sortilin-related VPS10 domain containing receptor 2	ENSMUSG00000029093	380960	7,61
Tro	trophinin	ENSMUSG00000025272	12280	7,61
Zbtb12	zinc finger and BTB domain containing 12	ENSMUSG00000049823	17360	7,61
Gpr39	G protein-coupled receptor 39	ENSMUSG00000026343	196868	7,61
Dpp7	dipeptidylpeptidase 7	ENSMUSG00000026958	4084	7,61
Lipa	lysosomal acid lipase A	ENSMUSG00000024781	35157	7,61
Fhod1	formin homology 2 domain containing 1	ENSMUSG00000014778	18791	7,61
Phf15	PHD finger protein 15	ENSMUSG00000020387	44199	7,61
Setd4	SET domain containing 4	ENSMUSG00000022948	20607	7,61
Ackr4	atypical chemokine receptor 4	ENSMUSG00000079355	28506	7,61
Alox5	arachidonate 5-lipoxygenase	ENSMUSG00000025701	51102	7,61
Chgb	chromogranin B	ENSMUSG00000027350	13802	7,54
Pelo	pelota homolog (Drosophila)	ENSMUSG00000042275	1832	7,54
Psmg3	proteasome (prosome, macropain) assembly chaperone 3	ENSMUSG00000029551	3294	7,54
Celf6	CUGBP, Elav-like family member 6	ENSMUSG00000032297	29376	7,54
Mtfmt	mitochondrial methionyl-tRNA formyltransferase	ENSMUSG00000059183	17273	7,54
Lmf2	lipase maturation factor 2	ENSMUSG00000022614	4656	7,54
Alg8	asparagine-linked glycosylation 8 (alpha-1,3-glucosyltransferase)	ENSMUSG00000035704	20548	7,54
Nubp2	nucleotide binding protein 2	ENSMUSG00000039183	3740	7,54
		ENSMUSG00000091609	4130	7,54
Melk	maternal embryonic leucine zipper kinase	ENSMUSG00000035683	63800	7,54
Cyp7b1	cytochrome P450, family 7, subfamily b, polypeptide 1	ENSMUSG00000039519	171389	7,54
Orc1	origin recognition complex, subunit 1	ENSMUSG00000028587	35411	7,54
Mob3c	MOB kinase activator 3C	ENSMUSG00000028709	8094	7,54
Usp18	ubiquitin specific peptidase 18	ENSMUSG00000030107	25011	7,54

Catsperg1	catsper channel auxiliary subunit gamma 1	ENSMUSG00000049676	32715	7,54
		ENSMUSG00000026162	79501	7,54
Cyp4b1	cytochrome P450, family 4, subfamily b, polypeptide 1	ENSMUSG00000028713	22985	7,54
Best1	bestrophin 1	ENSMUSG00000037418	16460	7,54
Mx2	myxovirus (influenza virus) resistance 2	ENSMUSG00000023341	24817	7,54
Cxcl2	chemokine (C-X-C motif) ligand 2	ENSMUSG00000058427	2011	7,54
Praf2	PRA1 domain family 2	ENSMUSG00000031149	2626	7,47
Dhx34	DEAH (Asp-Glu-Ala-His) box polypeptide 34	ENSMUSG00000006019	24891	7,47
Ncan	neurocan	ENSMUSG00000002341	27789	7,47
Tbx19	T-box 19	ENSMUSG00000026572	22919	7,47
Dbndd1	dysbindin (dystrobrevin binding protein 1) domain containing 1	ENSMUSG00000031970	10746	7,47
Mtcp1	mature T cell proliferation 1	ENSMUSG00000090110	11739	7,47
Nudt1	nudix (nucleoside diphosphate linked moiety X)-type motif 1	ENSMUSG00000036639	6278	7,47
Il17d	interleukin 17D	ENSMUSG00000050222	18337	7,47
Calhm2	calcium homeostasis modulator 2	ENSMUSG00000033033	32942	7,47
		ENSMUSG00000078899	16243	7,47
Oat	ornithine aminotransferase	ENSMUSG00000030934	18924	7,47
Kifc5b	kinesin family member C5B	ENSMUSG00000024301	15489	7,47
Zfp28	zinc finger protein 28	ENSMUSG00000062861	13316	7,47
Tnfrsf11a	tumor necrosis factor receptor superfamily, member 11a	ENSMUSG00000026321	67259	7,47
Trim12a	tripartite motif-containing 12A	ENSMUSG00000066258	15573	7,47
Cntnap4	contactin associated protein-like 4	ENSMUSG00000031772	312675	7,47
Skint5	selection and upkeep of intraepithelial T cells 5	ENSMUSG00000078598	521613	7,47
Gm17296	predicted gene, 17296	ENSMUSG00000090290	48414	7,47
Morn3	MORN repeat containing 3	ENSMUSG00000029477	11248	7,47
Cpne9	copine family member IX	ENSMUSG00000030270	23321	7,47
Smagp	small cell adhesion glycoprotein	ENSMUSG00000053559	15524	7,47
S100a9	S100 calcium binding protein A9 (calgranulin B)	ENSMUSG00000056071	3090	7,39
Rap2b	RAP2B, member of RAS oncogene family	ENSMUSG00000036894	4197	7,39
Mtg1	mitochondrial GTPase 1 homolog (S. cerevisiae)	ENSMUSG00000039018	13223	7,39
Fstl3	follistatin-like 3	ENSMUSG00000020325	5359	7,39
Pik3r2	phosphatidylinositol 3-kinase, regulatory subunit, polypeptide 2 (p85 beta)	ENSMUSG00000031834	8538	7,39
Cisd3	CDGSH iron sulfur domain 3	ENSMUSG00000078695	2796	7,39
Tfap4	transcription factor AP4	ENSMUSG00000005718	15060	7,39
Zfp3	zinc finger protein 3	ENSMUSG00000043602	8720	7,39
Ccdc130	coiled-coil domain containing 130	ENSMUSG00000004994	12586	7,39
Iqcc	IQ motif containing C	ENSMUSG00000040795	3974	7,39
Gas2l3	growth arrest-specific 2 like 3	ENSMUSG00000074802	35145	7,39
Tceanc	transcription elongation factor A (SII) N-terminal and central domain containing	ENSMUSG00000051224	18756	7,39
BC026590	cDNA sequence BC026590	ENSMUSG00000038827	7257	7,39

Pcsk4	proprotein convertase subtilisin/kexin type 4	ENSMUSG00000020131	8216	7,39
Rdh5	retinol dehydrogenase 5	ENSMUSG00000025350	9296	7,39
Ddx41	DEAD (Asp-Glu-Ala-Asp) box polypeptide 41	ENSMUSG00000021494	6249	7,39
		ENSMUSG00000075359	2374	7,39
Cldn2	claudin 2	ENSMUSG00000047230	10559	7,39
Fkbp11	FK506 binding protein 11	ENSMUSG00000003355	3826	7,39
Zfp808	zinc finger protein 80	ENSMUSG00000074867	43504	7,39
		ENSMUSG00000078185	1866	7,39
Fshr	follicle stimulating hormone receptor	ENSMUSG00000032937	215443	7,39
Cd300lg	CD300 antigen like family member G	ENSMUSG00000017309	14112	7,39
Nap1l5	nucleosome assembly protein 1-like 5	ENSMUSG00000055430	1893	7,32
Atp8b3	ATPase, class I, type 8B, member 3	ENSMUSG00000003341	19540	7,32
Enpp1	ectonucleotide pyrophosphatase/phosphodiesterase 1	ENSMUSG00000037370	74246	7,32
Tekt2	tektin 2	ENSMUSG00000028845	3568	7,32
Fam229b	family with sequence similarity 229, member B	ENSMUSG00000051736	15111	7,32
Tspan4	tetraspanin 4	ENSMUSG00000025511	18188	7,32
Smco4	single-pass membrane protein with coiled-coil domains 4	ENSMUSG00000058173	39774	7,32
Slmo2	slowmo homolog 2 (Drosophila)	ENSMUSG00000016257	8015	7,32
Cdc25c	cell division cycle 25C	ENSMUSG00000044201	18537	7,32
Nacad	NAC alpha domain containing	ENSMUSG00000041073	8231	7,32
C920021L13Rik	RIKEN cDNA C920021L13 gene	ENSMUSG00000080727	17572	7,32
Ost4	oligosaccharyltransferase 4 homolog (S. cerevisiae)	ENSMUSG00000038803	1904	7,32
Cnpy3	canopy 3 homolog (zebrafish)	ENSMUSG00000023973	16510	7,32
Ginm1	glycoprotein integral membrane 1	ENSMUSG00000040006	24854	7,32
Ufsp1	UFM1-specific peptidase 1	ENSMUSG00000051502	996	7,32
Gm5617	predicted gene 5617	ENSMUSG00000042293	620	7,32
Tmem14a	transmembrane protein 14A	ENSMUSG00000025933	11593	7,32
Gck	glucokinase	ENSMUSG00000041798	49262	7,32
Trpc4	transient receptor potential cation channel, subfamily C, member 4	ENSMUSG00000027748	162414	7,32
Trim30d	tripartite motif-containing 30D	ENSMUSG00000057596	37836	7,32
Nkrf	NF-kappaB repressing factor	ENSMUSG00000044149	15360	7,32
Zfp251	zinc finger protein 251	ENSMUSG00000022526	19288	7,32
Hmgcll1	3-hydroxymethyl-3-methylglutaryl-Coenzyme A lyase-like 1	ENSMUSG00000007908	121496	7,32
Figf	c-fos induced growth factor	ENSMUSG00000031380	29273	7,32
Serpina1b	serine (or cysteine) preptidase inhibitor, clade A, member 1B	ENSMUSG00000071178	102218	7,32
Pik3ap1	phosphoinositide-3-kinase adaptor protein 1	ENSMUSG00000025017	110853	7,32
Rnf113a1	ring finger protein 113A1	ENSMUSG00000036537	1218	7,25
Tmem200c	transmembrane protein 200C	ENSMUSG00000095407	6003	7,25
Atp6v1g2	ATPase, H+ transporting, lysosomal V1 subunit G2	ENSMUSG00000024403	5108	7,25

Upf3a	UPF3 regulator of nonsense transcripts homolog A (yeast)	ENSMUSG00000038398	12924	7,25
1190007I07Rik	RIKEN cDNA 1190007I07 gene	ENSMUSG00000063320	3346	7,25
Tmem115	transmembrane protein 115	ENSMUSG00000010045	4712	7,25
Acsm3	acyl-CoA synthetase medium-chain family member 3	ENSMUSG00000030935	26591	7,25
Slx4	SLX4 structure-specific endonuclease subunit homolog (S. cerevisiae)	ENSMUSG00000039738	24666	7,25
Fibp	fibroblast growth factor (acidic) intracellular binding protein	ENSMUSG00000024911	4446	7,25
Spire2	spire homolog 2 (Drosophila)	ENSMUSG00000010154	36803	7,25
Alas2	aminolevulinic acid synthase 2, erythroid	ENSMUSG00000025270	23264	7,25
Rasl10b	RAS-like, family 10, member B	ENSMUSG00000020684	11903	7,25
D2hgdh	D-2-hydroxyglutarate dehydrogenase	ENSMUSG00000073609	27109	7,25
Rbm20	RNA binding motif protein 20	ENSMUSG00000043639	189775	7,25
Slc2a10	solute carrier family 2 (facilitated glucose transporter), member 10	ENSMUSG00000027661	16131	7,25
Onecut2	one cut domain, family member 2	ENSMUSG00000045991	58123	7,25
Serpine3	serpin peptidase inhibitor, clade E (nexin, plasminogen activator inhibitor type 1), member 3	ENSMUSG00000091155	28577	7,25
Lhfpl1	lipoma HMGIC fusion partner-like 1	ENSMUSG00000041700	58731	7,25
Zfp442	zinc finger protein 442	ENSMUSG00000068130	44346	7,25
Gem	GTP binding protein (gene overexpressed in skeletal muscle)	ENSMUSG00000028214	10296	7,25
Mdfi	MyoD family inhibitor	ENSMUSG00000032717	19364	7,25
Abi3bp	ABI gene family, member 3 (NESH) binding protein	ENSMUSG00000035258	212283	7,18
Ccbl2	cysteine conjugate-beta lyase 2	ENSMUSG00000040213	43860	7,18
0610009B22Rik	RIKEN cDNA 0610009B22 gene	ENSMUSG00000007777	3489	7,18
Cmpk2	cytidine monophosphate (UMP-CMP) kinase 2, mitochondrial	ENSMUSG00000020638	10632	7,18
9130019O22Rik	RIKEN cDNA 9130019O22 gene	ENSMUSG00000030823	4907	7,18
Zfp513	zinc finger protein 513	ENSMUSG00000043059	3323	7,18
Cep55	centrosomal protein 55	ENSMUSG00000024989	19401	7,18
Fzd5	frizzled homolog 5 (Drosophila)	ENSMUSG00000045005	7191	7,18
Suv39h2	suppressor of variegation 3-9 homolog 2 (Drosophila)	ENSMUSG00000026646	19217	7,18
Cpsf1	cleavage and polyadenylation specific factor 1	ENSMUSG00000034022	11783	7,18
Mtrr	5-methyltetrahydrofolate-homocysteine methyltransferase reductase	ENSMUSG00000034617	21342	7,18
Acads	acyl-Coenzyme A dehydrogenase, short chain	ENSMUSG00000029545	9048	7,18
Pold1	polymerase (DNA directed), delta 1, catalytic subunit	ENSMUSG00000038644	16104	7,18
Galnt4	UDP-N-acetyl-alpha-D-galactosamine:polypeptide N-acetylgalactosaminyltransferase 4	ENSMUSG00000090035	5111	7,18
Slc29a1	solute carrier family 29 (nucleoside transporters), member 1	ENSMUSG00000023942	14407	7,18

Ly6g6e	lymphocyte antigen 6 complex, locus G6E	ENSMUSG00000013766	1903	7,18
		ENSMUSG00000020870	1233	7,18
Ccnb2	cyclin B2	ENSMUSG00000032218	13866	7,18
Mat1a	methionine adenosyltransferase I, alpha	ENSMUSG00000037798	19032	7,18
Rmi2	RMI2, RecQ mediated genome instability 2, homolog (S. cerevisiae)	ENSMUSG00000037991	57908	7,18
		ENSMUSG00000078784	4902	7,18
Prx	periaxin	ENSMUSG00000053198	20891	7,18
Flrt3	fibronectin leucine rich transmembrane protein 3	ENSMUSG00000051379	20556	7,18
		ENSMUSG00000091144	14549	7,18
Zfp711	zinc finger protein 711	ENSMUSG00000025529	34545	7,18
1700113H08Rik	RIKEN cDNA 1700113H08 gene	ENSMUSG00000047129	172554	7,18
Gdpd4	glycerophosphodiester phosphodiesterase domain containing 4	ENSMUSG00000035582	129709	7,18
Pmel	premelanosome protein	ENSMUSG00000025359	13981	7,18
Gpr124	G protein-coupled receptor 124	ENSMUSG00000031486	37854	7,10
Mafb	v-maf musculoaponeurotic fibrosarcoma oncogene family, protein B (avian)	ENSMUSG00000074622	3389	7,10
Elovl2	elongation of very long chain fatty acids (FEN1/Elo2, SUR4/Elo3, yeast)-like 2	ENSMUSG00000021364	37911	7,10
Rnmtl1	RNA methyltransferase like 1	ENSMUSG00000038046	6905	7,10
9630033F20Rik	RIKEN cDNA 9630033F20 gene	ENSMUSG00000038028	24435	7,10
Dkkl1	dickkopf-like 1	ENSMUSG00000030792	4359	7,10
		ENSMUSG00000097830	2502	7,10
Pi16	peptidase inhibitor 16	ENSMUSG00000024011	10537	7,10
Mocs3	molybdenum cofactor synthesis 3	ENSMUSG00000074576	1681	7,10
Shcbp1	Shc SH2-domain binding protein 1	ENSMUSG00000022322	43555	7,10
Pno1	partner of NOB1 homolog (S. cerevisiae)	ENSMUSG00000020116	8371	7,10
Lipg	lipase, endothelial	ENSMUSG00000053846	21942	7,10
Csgalnact2	chondroitin sulfate N-acetylgalactosaminyltransferase 2	ENSMUSG00000042042	31689	7,10
		ENSMUSG00000072809	2766	7,10
L3mbtl2	l(3)mbt-like 2 (Drosophila)	ENSMUSG00000022394	24427	7,10
		ENSMUSG00000039579	184134	7,10
Shisa7	shisa homolog 7 (Xenopus laevis)	ENSMUSG00000053550	19145	7,10
		ENSMUSG00000027384	33041	7,10
		ENSMUSG00000057409	18509	7,10
Nfatc2	nuclear factor of activated T cells, cytoplasmic, calcineurin dependent 2	ENSMUSG00000027544	125248	7,10
1700034H15Rik	RIKEN cDNA 1700034H15 gene	ENSMUSG00000055833	17295	7,10
Mc2r	melanocortin 2 receptor	ENSMUSG00000045569	22345	7,10
Myl6b	myosin, light polypeptide 6B	ENSMUSG00000039824	4529	7,10
		ENSMUSG00000094958	62256	7,10
		ENSMUSG00000079021	20535	7,10
		ENSMUSG00000068617	19243	7,10
Tmprss11f	transmembrane protease, serine 11f	ENSMUSG00000048764	110527	7,10

Col1a2	collagen, type I, alpha 2	ENSMUSG00000029661	36730	7,03
Mlx	MAX-like protein X	ENSMUSG00000017801	4931	7,03
Tmem143	transmembrane protein 143	ENSMUSG00000002781	20473	7,03
Siah1b	seven in absentia 1B	ENSMUSG00000040749	5789	7,03
Phf21b	PHD finger protein 21B	ENSMUSG00000016624	70669	7,03
Mrps11	mitochondrial ribosomal protein S11	ENSMUSG00000030611	9871	7,03
Cdt1	chromatin licensing and DNA replication factor 1	ENSMUSG00000006585	5114	7,03
Ntf3	neurotrophin 3	ENSMUSG00000049107	65332	7,03
Armcx3	armadillo repeat containing, X-linked 3	ENSMUSG00000049047	4861	7,03
Ckap2	cytoskeleton associated protein 2	ENSMUSG00000037725	17668	7,03
Polr3a	polymerase (RNA) III (DNA directed) polypeptide A	ENSMUSG00000025280	38353	7,03
Ribc1	RIB43A domain with coiled-coils 1	ENSMUSG00000025257	11714	7,03
Gm3383	predicted gene 3383	ENSMUSG00000096629	200363	7,03
Syde2	synapse defective 1, Rho GTPase, homolog 2 (C. elegans)	ENSMUSG00000036863	33851	7,03
A530054K11Rik	RIKEN cDNA A530054K11 gene	ENSMUSG00000021510	20791	7,03
Xk	Kell blood group precursor (McLeod phenotype) homolog	ENSMUSG00000015342	40495	7,03
Lpxn	leupaxin	ENSMUSG00000024696	35202	7,03
		ENSMUSG00000041748	18404	7,03
Lpar3	lysophosphatidic acid receptor 3	ENSMUSG00000036832	65254	7,03
Pstpip1	proline-serine-threonine phosphatase-interacting protein 1	ENSMUSG00000032322	38927	7,03
Slc15a5	solute carrier family 15, member 5	ENSMUSG00000044378	96331	7,03
Wdr93	WD repeat domain 93	ENSMUSG00000039099	42788	7,03
Efnb3	ephrin B3	ENSMUSG00000003934	6114	6,96
Fkbpl	FK506 binding protein-like	ENSMUSG00000033739	1561	6,96
Zfp280b	zinc finger protein 280B	ENSMUSG00000049764	10581	6,96
Ppp1r26	protein phosphatase 1, regulatory subunit 26	ENSMUSG00000035829	8709	6,96
Rtn4ip1	reticulon 4 interacting protein 1	ENSMUSG00000019864	46056	6,96
Rbm12b2	RNA binding motif protein 12 B2	ENSMUSG00000052137	6805	6,96
Stard5	StAR-related lipid transfer (START) domain containing 5	ENSMUSG00000046027	21169	6,96
Zfp521	zinc finger protein 521	ENSMUSG00000024420	285721	6,96
		ENSMUSG00000030804	9228	6,96
Exosc8	exosome component 8	ENSMUSG00000027752	6716	6,96
Tbx1	T-box 1	ENSMUSG00000009097	5257	6,96
Nr2c2ap	nuclear receptor 2C2-associated protein	ENSMUSG00000071078	2407	6,96
Cutc	cutC copper transporter homolog (E.coli)	ENSMUSG00000025193	15643	6,96
Tymp	thymidine phosphorylase	ENSMUSG00000022615	5109	6,96
Enpp5	ectonucleotide pyrophosphatase/phosphodiesterase 5	ENSMUSG00000023960	7755	6,96
Nrip3	nuclear receptor interacting protein 3	ENSMUSG00000034825	23971	6,96
Card10	caspase recruitment domain family, member 10	ENSMUSG00000033170	27905	6,96
Zfp51	zinc finger protein 51	ENSMUSG00000023892	15240	6,96

Echdc3	enoyl Coenzyme A hydratase domain containing 3	ENSMUSG00000039063	24569	6,96
Tas1r1	taste receptor, type 1, member 1	ENSMUSG00000028950	10655	6,96
Tmem132b	transmembrane protein 132B	ENSMUSG00000070498	260166	6,96
Shank3	SH3/ankyrin domain gene 3	ENSMUSG00000022623	60639	6,96
Ttll9	tubulin tyrosine ligase-like family, member 9	ENSMUSG00000074673	45998	6,96
		ENSMUSG00000074968	295723	6,96
Wibg	within bgcn homolog (Drosophila)	ENSMUSG00000064030	18690	6,96
Zc3h12a	zinc finger CCCH type containing 12A	ENSMUSG00000042677	9418	6,96
Nckap5l	NCK-associated protein 5-like	ENSMUSG00000023009	35714	6,96
Pipox	pipecolic acid oxidase	ENSMUSG00000017453	13482	6,96
Crybb3	crystallin, beta B3	ENSMUSG00000029352	5746	6,96
Mmp9	matrix metallopeptidase 9	ENSMUSG00000017737	15071	6,89
Dio2	deiodinase, iodothyronine, type II	ENSMUSG00000007682	13885	6,89
Gemin6	gem (nuclear organelle) associated protein 6	ENSMUSG00000055760	4057	6,89
Rab13	RAB13, member RAS oncogene family	ENSMUSG00000027935	12691	6,89
Mettl21a	methyltransferase like 21A	ENSMUSG00000025956	10770	6,89
2310039H08Rik	RIKEN cDNA 2310039H08 gene	ENSMUSG00000062619	773	6,89
Soat1	sterol O-acyltransferase 1	ENSMUSG00000026600	46221	6,89
Ttf2	transcription termination factor, RNA polymerase II	ENSMUSG00000033222	30804	6,89
		ENSMUSG00000074589	60517	6,89
Zfp772	zinc finger protein 772	ENSMUSG00000066838	7877	6,89
Taco1	translational activator of mitochondrially encoded cytochrome c oxidase I	ENSMUSG00000001983	7552	6,89
Fignl1	fidgetin-like 1	ENSMUSG00000035455	21532	6,89
Zfp11	zinc finger protein 11	ENSMUSG00000051034	15507	6,89
Wdr5b	WD repeat domain 5B	ENSMUSG00000034379	1784	6,89
Aim1	absent in melanoma 1	ENSMUSG00000019866	54540	6,89
Fam102b	family with sequence similarity 102, member B	ENSMUSG00000040339	56611	6,89
Actl6b	actin-like 6B	ENSMUSG00000029712	16066	6,89
5730559C18Rik	RIKEN cDNA 5730559C18 gene	ENSMUSG00000041605	20759	6,89
Vstm2a	V-set and transmembrane domain containing 2A	ENSMUSG00000048834	169587	6,89
Thsd1	thrombospondin, type I, domain 1	ENSMUSG00000031480	34022	6,89
Il17rc	interleukin 17 receptor C	ENSMUSG00000030281	11714	6,89
Ppil6	peptidylprolyl isomerase (cyclophilin)-like 6	ENSMUSG00000078451	23846	6,89
Gm14459	predicted gene 14459	ENSMUSG00000079704	10903	6,89
Plcd4	phospholipase C, delta 4	ENSMUSG00000026173	24907	6,89
Arhgap30	Rho GTPase activating protein 30	ENSMUSG00000048865	21345	6,89
		ENSMUSG00000025754	448538	6,89
Gm16432	predicted gene 16432	ENSMUSG00000091476	56857	6,89
Npsr1	neuropeptide S receptor 1	ENSMUSG00000043659	218403	6,89
LOC101055656	uncharacterized LOC101055656	ENSMUSG00000084085	2056	6,89
Lrrtm2	leucine rich repeat transmembrane neuronal 2	ENSMUSG00000071862	6014	6,89
Sftpc	surfactant associated protein C	ENSMUSG00000022097	3141	6,89

Chst7	carbohydrate (N-acetylglucosamino) sulfotransferase 7	ENSMUSG00000037347	37962	6,89
Ampd1	adenosine monophosphate deaminase 1	ENSMUSG00000070385	25701	6,89
Dcpp1	demilune cell and parotid protein 1	ENSMUSG00000096445	1978	6,81
Arhgdib	Rho, GDP dissociation inhibitor (GDI) beta	ENSMUSG00000030220	18377	6,81
Lrp1	low density lipoprotein receptor-related protein 1	ENSMUSG00000040249	82988	6,81
1700001L19Rik	RIKEN cDNA 1700001L19 gene	ENSMUSG00000021534	16793	6,81
Ptgis	prostaglandin I2 (prostacyclin) synthase	ENSMUSG00000017969	48800	6,81
Foxred1	FAD-dependent oxidoreductase domain containing 1	ENSMUSG00000039048	6850	6,81
Zcchc12	zinc finger, CCHC domain containing 12	ENSMUSG00000036699	3255	6,81
Ddx59	DEAD (Asp-Glu-Ala-Asp) box polypeptide 59	ENSMUSG00000026404	24888	6,81
Rpain	RPA interacting protein	ENSMUSG00000018449	7621	6,81
Ring1	ring finger protein 1	ENSMUSG00000024325	3889	6,81
Neu2	neuraminidase 2	ENSMUSG00000079434	87957	6,81
Syngr3	synaptogyrin 3	ENSMUSG00000007021	4864	6,81
1700010I14Rik	RIKEN cDNA 1700010I14 gene	ENSMUSG00000023873	19987	6,81
Gpn3	GPN-loop GTPase 3	ENSMUSG00000029464	11027	6,81
Mast3	microtubule associated serine/threonine kinase 3	ENSMUSG00000031833	14317	6,81
Ap5s1	adaptor-related protein 5 complex, sigma 1 subunit	ENSMUSG00000068264	6437	6,81
Zfp276	zinc finger protein (C2H2 type) 276	ENSMUSG00000001065	15551	6,81
		ENSMUSG00000016833	5845	6,81
4930523C07Rik	RIKEN cDNA 4930523C07 gene	ENSMUSG00000090394	34045	6,81
Catsperd	catsper channel auxiliary subunit delta	ENSMUSG00000040828	36314	6,81
Fancd2	Fanconi anemia, complementation group D2	ENSMUSG00000034023	65336	6,81
Clec12a	C-type lectin domain family 12, member a	ENSMUSG00000053063	22613	6,81
		ENSMUSG00000034551	127149	6,81
Msh4	mutS homolog 4 (E. coli)	ENSMUSG00000005493	48992	6,81
1700061G19Rik	RIKEN cDNA 1700061G19 gene	ENSMUSG00000024209	13428	6,81
		ENSMUSG00000037336	11920	6,81
Neurog1	neurogenin 1	ENSMUSG00000048904	1659	6,74
Gpn2	GPN-loop GTPase 2	ENSMUSG00000028848	7374	6,74
Rnf112	ring finger protein 112	ENSMUSG00000010086	5690	6,74
		ENSMUSG00000079615	8231	6,74
Lsm3	LSM3 homolog, U6 small nuclear RNA associated (S. cerevisiae)	ENSMUSG00000034192	6698	6,74
5830403L16Rik	RIKEN cDNA 5830403L16 gene	ENSMUSG00000045968	50667	6,74
Ptgs1	prostaglandin-endoperoxide synthase 1	ENSMUSG00000047250	21847	6,74
Churc1	churchill domain containing 1	ENSMUSG00000090258	17643	6,74
Wdr74	WD repeat domain 74	ENSMUSG00000042729	4785	6,74
Mpp4	membrane protein, palmitoylated 4 (MAGUK p55 subfamily member 4)	ENSMUSG00000079550	42454	6,74

Larp6	La ribonucleoprotein domain family, member 6	ENSMUSG00000034839	25813	6,74
C030006K11Rik	RIKEN cDNA C030006K11 gene	ENSMUSG00000079002	2380	6,74
Vcan	versican	ENSMUSG00000021614	87198	6,74
Tmem79	transmembrane protein 79	ENSMUSG00000001420	5850	6,74
Akr1c14	aldo-keto reductase family 1, member C14	ENSMUSG00000033715	41412	6,74
Napepld	N-acyl phosphatidylethanolamine phospholipase D	ENSMUSG00000044968	38496	6,74
Entpd6	ectonucleoside triphosphate diphosphohydrolase 6	ENSMUSG00000033068	22634	6,74
B3gnt7	UDP-GlcNAc:betaGal beta-1,3-N-acetylglucosaminyltransferase 7	ENSMUSG00000079445	4083	6,74
E030030I06Rik	RIKEN cDNA E030030I06 gene	ENSMUSG00000055657	36221	6,74
Pole2	polymerase (DNA directed), epsilon 2 (p59 subunit)	ENSMUSG00000020974	26412	6,74
Tmem62	transmembrane protein 62	ENSMUSG00000054484	30836	6,74
Kcnj13	potassium inwardly-rectifying channel, subfamily J, member 13	ENSMUSG00000079436	8367	6,74
Nckipsd	NCK interacting protein with SH3 domain	ENSMUSG00000032598	9987	6,74
F830045P16Rik	RIKEN cDNA F830045P16 gene	ENSMUSG00000043727	78244	6,74
		ENSMUSG00000057058	294538	6,74
Mgat4c	mannosyl (alpha-1,3-)-glycoprotein beta-1,4-N-acetylglucosaminyltransferase, isozyme C (putative)	ENSMUSG00000019888	709983	6,74
Pate2	prostate and testis expressed 2	ENSMUSG00000074452	120035	6,74
Cngb3	cyclic nucleotide gated channel beta 3	ENSMUSG00000056494	229774	6,74
Lancl3	LanC lantibiotic synthetase component C-like 3 (bacterial)	ENSMUSG00000047344	68184	6,74
Rxra	retinoid X receptor alpha	ENSMUSG00000015846	86518	6,67
Clip4	CAP-GLY domain containing linker protein family, member 4	ENSMUSG00000024059	76405	6,67
Ptgdr	prostaglandin D receptor	ENSMUSG00000071489	8141	6,67
Men1	multiple endocrine neoplasia 1	ENSMUSG00000024947	5913	6,67
Cenpa	centromere protein A	ENSMUSG00000029177	8051	6,67
Zc4h2	zinc finger, C4H2 domain containing	ENSMUSG00000035062	19317	6,67
Gm9958	predicted gene 9958	ENSMUSG00000054945	1284	6,67
Zfp27	zinc finger protein 27	ENSMUSG00000062040	13240	6,67
M1ap	meiosis 1 associated protein	ENSMUSG00000030041	83282	6,67
Ppfia4	protein tyrosine phosphatase, receptor type, f polypeptide (PTPRF), interacting protein (liprin), alpha 4	ENSMUSG00000026458	36146	6,67
Obsl1	obscurin-like 1	ENSMUSG00000026211	27143	6,67
Fsip1	fibrous sheath-interacting protein 1	ENSMUSG00000027344	52079	6,67
		ENSMUSG00000091277	5790	6,67
Kank3	KN motif and ankyrin repeat domains 3	ENSMUSG00000042099	12399	6,67
Stc1	stanniocalcin 1	ENSMUSG00000014813	12113	6,67
Mypn	myopalladin	ENSMUSG00000020067	88158	6,67
Trub1	TruB pseudouridine (psi) synthase homolog 1 (E. coli)	ENSMUSG00000025086	38100	6,67

Zkscan14	zinc finger with KRAB and SCAN domains 14	ENSMUSG00000029627	6923	6,67
Dguok	deoxyguanosine kinase	ENSMUSG00000014554	26753	6,67
Zcchc10	zinc finger, CCHC domain containing 10	ENSMUSG00000018239	8623	6,67
Ppm1f	protein phosphatase 1F (PP2C domain containing)	ENSMUSG00000026181	30896	6,67
Trdn	triadin	ENSMUSG00000019787	393227	6,67
		ENSMUSG00000062808	1249	6,67
		ENSMUSG00000042360	65936	6,67
Adamts19	a disintegrin-like and metallopeptidase (reprolysin type) with thrombospondin type 1 motif, 19	ENSMUSG00000053441	216915	6,67
Serpinc1	serine (or cysteine) peptidase inhibitor, clade C (antithrombin), member 1	ENSMUSG00000026715	24405	6,67
1700011E24Rik	RIKEN cDNA 1700011E24 gene	ENSMUSG00000036557	38171	6,67
Olfr78	olfactory receptor 78	ENSMUSG00000043366	18751	6,67
Nrm	nurim (nuclear envelope membrane protein)	ENSMUSG00000059791	4085	6,60
Ceacam1	carcinoembryonic antigen-related cell adhesion molecule 1	ENSMUSG00000074272	15924	6,60
Hspa12a	heat shock protein 12A	ENSMUSG00000025092	65234	6,60
Gltscr2	glioma tumor suppressor candidate region gene 2	ENSMUSG00000041560	8271	6,60
Zkscan4	zinc finger with KRAB and SCAN domains 4	ENSMUSG00000054931	6601	6,60
Isoc2b	isochorismatase domain containing 2b	ENSMUSG00000052605	21235	6,60
Clstn3	calsyntenin 3	ENSMUSG00000008153	34036	6,60
Mad2l1	MAD2 mitotic arrest deficient-like 1	ENSMUSG00000029910	5644	6,60
Smcr8	Smith-Magenis syndrome chromosome region, candidate 8 homolog (human)	ENSMUSG00000049323	10763	6,60
Msrb1	methionine sulfoxide reductase B1	ENSMUSG00000075705	6137	6,60
Zbtb33	zinc finger and BTB domain containing 33	ENSMUSG00000048047	7254	6,60
Fjx1	four jointed box 1 (Drosophila)	ENSMUSG00000075012	2425	6,60
Sirt4	sirtuin 4	ENSMUSG00000029524	6716	6,60
		ENSMUSG00000026163	154062	6,60
Klhl31	kelch-like 31	ENSMUSG00000044938	23387	6,60
Atic	5-aminoimidazole-4-carboxamide ribonucleotide formyltransferase/IMP cyclohydrolase	ENSMUSG00000026192	22482	6,60
Lysmd3	LysM, putative peptidoglycan-binding, domain containing 3	ENSMUSG00000035840	14083	6,60
		ENSMUSG00000089922	22173	6,60
		ENSMUSG00000079386	88461	6,60
Zfp938	zinc finger protein 938	ENSMUSG00000062931	16431	6,60
Neil2	nei like 2 (E. coli)	ENSMUSG00000035121	11099	6,60
Cav2	caveolin 2	ENSMUSG00000000058	7931	6,60
Mettl4	methyltransferase like 4	ENSMUSG00000055660	22813	6,60
Gla	galactosidase, alpha	ENSMUSG00000031266	12977	6,60
		ENSMUSG00000069518	15786	6,60

Dpy19l2	dpy-19-like 2 (C. elegans)	ENSMUSG00000085576	139246	6,60
Kcne3	potassium voltage-gated channel, Isk-related subfamily, gene 3	ENSMUSG00000035165	8200	6,60
Olfr138	olfactory receptor 138	ENSMUSG00000057443	14645	6,60
Ces1a	carboxylesterase 1A	ENSMUSG00000071047	27979	6,52
Col8a1	collagen, type VIII, alpha 1	ENSMUSG00000068196	130480	6,52
Cdh11	cadherin 11	ENSMUSG00000031673	152117	6,52
		ENSMUSG00000000202	4729	6,52
Tspan6	tetraspanin 6	ENSMUSG00000067377	7362	6,52
		ENSMUSG00000048106	1077	6,52
Matn4	matrilin 4	ENSMUSG00000016995	15768	6,52
Ccdc135	coiled-coil domain containing 135	ENSMUSG00000031786	23039	6,52
Psph	phosphoserine phosphatase	ENSMUSG00000029446	21695	6,52
		ENSMUSG00000078580	3957	6,52
Rnase4	ribonuclease, RNase A family 4	ENSMUSG00000021876	15075	6,52
		ENSMUSG00000091854	1715	6,52
		ENSMUSG00000062753	2175	6,52
Nudt11	nudix (nucleoside diphosphate linked moiety X)-type motif 11	ENSMUSG00000073295	7658	6,52
Slc39a13	solute carrier family 39 (metal ion transporter), member 13	ENSMUSG00000002105	8627	6,52
Zfp37	zinc finger protein 37	ENSMUSG00000028389	18907	6,52
		ENSMUSG00000072829	995	6,52
Zfp69	zinc finger protein 69	ENSMUSG00000064141	21563	6,52
Nbeal2	neurobeachin-like 2	ENSMUSG00000056724	29373	6,52
Dr1	down-regulator of transcription 1	ENSMUSG00000029265	11630	6,52
Thtpa	thiamine triphosphatase	ENSMUSG00000045691	4152	6,52
Zbtb8a	zinc finger and BTB domain containing 8a	ENSMUSG00000028807	24489	6,52
Mndal	myeloid nuclear differentiation antigen like	ENSMUSG00000090272	85271	6,52
Rapgef4	Rap guanine nucleotide exchange factor (GEF) 4	ENSMUSG00000049044	276235	6,52
Lypd6b	LY6/PLAUR domain containing 6B	ENSMUSG00000026765	161162	6,52
Lrrc71	leucine rich repeat containing 71	ENSMUSG00000023084	11703	6,52
		ENSMUSG00000040350	26070	6,52
		ENSMUSG00000063245	47200	6,52
C9	complement component 9	ENSMUSG00000022149	53425	6,52
Lox	lysyl oxidase	ENSMUSG00000024529	13799	6,45
Fam64a	family with sequence similarity 64, member A	ENSMUSG00000020808	5339	6,45
B930041F14Rik	RIKEN cDNA B930041F14 gene	ENSMUSG00000074738	2140	6,45
Hfe	hemochromatosis	ENSMUSG00000006611	8821	6,45
Sema3b	sema domain, immunoglobulin domain (Ig), short basic domain, secreted, (semaphorin) 3B	ENSMUSG00000057969	11556	6,45
		ENSMUSG00000028992	17631	6,45
Serf1	small EDRK-rich factor 1	ENSMUSG00000021643	6575	6,45
Syngr2	synaptogyrin 2	ENSMUSG00000048277	4616	6,45
		ENSMUSG00000039253	9888	6,45
Ccnj	cyclin J	ENSMUSG00000025010	17294	6,45
Tmem220	transmembrane protein 220	ENSMUSG00000050270	10159	6,45
		ENSMUSG00000036504	1795	6,45
Lrrc27	leucine rich repeat containing 27	ENSMUSG00000015980	29992	6,45
Pnma2	paraneoplastic antigen MA2	ENSMUSG00000046204	8885	6,45

Tek	endothelial-specific receptor tyrosine kinase	ENSMUSG00000006386	135688	6,45
Bcam	basal cell adhesion molecule	ENSMUSG00000002980	14886	6,45
		ENSMUSG00000039345	19897	6,45
Actr6	ARP6 actin-related protein 6	ENSMUSG00000019948	20323	6,45
Cenpk	centromere protein K	ENSMUSG00000021714	20661	6,45
Eps8l2	EPS8-like 2	ENSMUSG00000025504	24141	6,45
		ENSMUSG00000024446	2425	6,45
Rgs17	regulator of G-protein signaling 17	ENSMUSG00000019775	96738	6,45
Cmah	cytidine monophospho-N-acetylneuraminic acid hydroxylase	ENSMUSG00000016756	149866	6,45
Oacyl	O-acyltransferase like	ENSMUSG00000046610	53334	6,45
Trim47	tripartite motif-containing 47	ENSMUSG00000020773	21459	6,45
Susd3	sushi domain containing 3	ENSMUSG00000021384	18017	6,45
Grm5	glutamate receptor, metabotropic 5	ENSMUSG00000049583	532364	6,45
Cyp4f16	cytochrome P450, family 4, subfamily f, polypeptide 16	ENSMUSG00000048440	15241	6,45
Myo15	myosin XV	ENSMUSG00000042678	59031	6,45
Htr2b	5-hydroxytryptamine (serotonin) receptor 2B	ENSMUSG00000026228	12945	6,45
Scgb3a1	secretoglobin, family 3A, member 1	ENSMUSG00000064057	1509	6,45
Prdx4	peroxiredoxin 4	ENSMUSG00000025289	16837	6,38
Paqr7	progestin and adipoQ receptor family member VII	ENSMUSG00000037348	13539	6,38
Tacc3	transforming, acidic coiled-coil containing protein 3	ENSMUSG00000037313	20868	6,38
Dpm3	dolichyl-phosphate mannosyltransferase polypeptide 3	ENSMUSG00000042737	7722	6,38
Gm17359	predicted gene, 17359	ENSMUSG00000091685	118754	6,38
Tcte3	t-complex-associated testis expressed 3	ENSMUSG00000079707	14403	6,38
Zfp932	zinc finger protein 932	ENSMUSG00000066613	13891	6,38
Tmem63c	transmembrane protein 63c	ENSMUSG00000034145	68704	6,38
Cetn4	centrin 4	ENSMUSG00000045031	4960	6,38
		ENSMUSG00000072258	20373	6,38
Gata4	GATA binding protein 4	ENSMUSG00000021944	72771	6,38
9530053A07Rik	RIKEN cDNA 9530053A07 gene	ENSMUSG00000078776	35346	6,38
Slc35f4	solute carrier family 35, member F4	ENSMUSG00000021852	227528	6,38
Arg1	arginase, liver	ENSMUSG00000019987	12263	6,38
Spock3	sparc/osteonectin, cwcv and kazal-like domains proteoglycan 3	ENSMUSG00000054162	406095	6,38
Dmc1	DMC1 dosage suppressor of mck1 homolog, meiosis-specific homologous recombination	ENSMUSG00000022429	43585	6,38
Shcbp1l	Shc SH2-domain binding protein 1-like	ENSMUSG00000042708	27413	6,38
Aass	aminoadipate-semialdehyde synthase	ENSMUSG00000029695	60814	6,38
Myrfl	myelin regulatory factor-like	ENSMUSG00000034057	120335	6,38
Slc25a30	solute carrier family 25, member 30	ENSMUSG00000022003	25039	6,38
Slc44a4	solute carrier family 44, member 4	ENSMUSG00000007034	15971	6,31
Tyw3	tRNA-yW synthesizing protein 3 homolog (S. cerevisiae)	ENSMUSG00000047583	20585	6,31

Gria3	glutamate receptor, ionotropic, AMPA3 (alpha 3)	ENSMUSG00000001986	277748	6,31
Nuf2	NUF2, NDC80 kinetochore complex component, homolog (S. cerevisiae)	ENSMUSG00000026683	33531	6,31
Tut1	terminal uridylyl transferase 1, U6 snRNA-specific	ENSMUSG00000071645	12358	6,31
Fn3k	fructosamine 3 kinase	ENSMUSG00000025175	15579	6,31
		ENSMUSG00000057594	2742	6,31
Surf1	surfeit gene 1	ENSMUSG00000015790	3148	6,31
Def8	differentially expressed in FDCP 8	ENSMUSG00000001482	20285	6,31
Scamp3	secretory carrier membrane protein 3	ENSMUSG00000028049	5293	6,31
Armcx5	armadillo repeat containing, X-linked 5	ENSMUSG00000072969	4590	6,31
Fastkd1	FAST kinase domains 1	ENSMUSG00000027086	26702	6,31
Cenpi	centromere protein I	ENSMUSG00000031262	54556	6,31
BC068281	cDNA sequence BC068281	ENSMUSG00000051721	13623	6,31
Gas8	growth arrest specific 8	ENSMUSG00000040220	17815	6,31
1700052N19Rik	RIKEN cDNA 1700052N19 gene	ENSMUSG00000061759	22675	6,31
		ENSMUSG00000097829	9843	6,31
Rwdd3	RWD domain containing 3	ENSMUSG00000028133	16296	6,31
Rhbdf1	rhomboid family 1 (Drosophila)	ENSMUSG00000020282	12716	6,31
4921507P07Rik	RIKEN cDNA 4921507P07 gene	ENSMUSG00000029828	23331	6,31
1700026L06Rik	RIKEN cDNA 1700026L06 gene	ENSMUSG00000026809	7648	6,31
		ENSMUSG00000074591	26053	6,31
Rtbdn	retbindin	ENSMUSG00000048617	9613	6,31
6030498E09Rik	RIKEN cDNA 6030498E09 gene	ENSMUSG00000051361	190016	6,31
Emr4	EGF-like module containing, mucin-like, hormone receptor-like sequence 4	ENSMUSG00000032915	103679	6,31
Slc22a22	solute carrier family 22 (organic cation transporter), member 22	ENSMUSG00000022366	233859	6,31
Slc7a8	solute carrier family 7 (cationic amino acid transporter, y+ system), member 8	ENSMUSG00000022180	59671	6,23
Cidea	cell death-inducing DNA fragmentation factor, alpha subunit-like effector A	ENSMUSG00000024526	24231	6,23
Rxrb	retinoid X receptor beta	ENSMUSG00000039656	6582	6,23
Nop2	NOP2 nucleolar protein	ENSMUSG00000038279	12871	6,23
Pglyrp3	peptidoglycan recognition protein 3	ENSMUSG00000042244	17000	6,23
Rad51ap1	RAD51 associated protein 1	ENSMUSG00000030346	16538	6,23
Iba57	IBA57, iron-sulfur cluster assembly homolog (S. cerevisiae)	ENSMUSG00000049287	8371	6,23
Hcn4	hyperpolarization-activated, cyclic nucleotide-gated K+ 4	ENSMUSG00000032338	37444	6,23
Mier2	mesoderm induction early response 1, family member 2	ENSMUSG00000042570	14955	6,23
Snapc2	small nuclear RNA activating complex, polypeptide 2	ENSMUSG00000011837	3118	6,23
Ndufaf3	NADH dehydrogenase (ubiquinone) 1 alpha subcomplex, assembly factor 3	ENSMUSG00000070283	1477	6,23
Ctsf	cathepsin F	ENSMUSG00000083282	5784	6,23

Ppapdc2	phosphatidic acid phosphatase type 2 domain containing 2	ENSMUSG00000040105	2879	6,23
Drg2	developmentally regulated GTP binding protein 2	ENSMUSG00000020537	14164	6,23
		ENSMUSG00000074873	2796	6,23
		ENSMUSG00000026975	9764	6,23
Asb13	ankyrin repeat and SOCS box-containing 13	ENSMUSG00000033781	17743	6,23
Ddo	D-aspartate oxidase	ENSMUSG00000063428	19921	6,23
Tor4a	torsin family 4, member A	ENSMUSG00000059555	3921	6,23
Ttc12	tetratricopeptide repeat domain 12	ENSMUSG00000040219	49263	6,23
Mlxipl	MLX interacting protein-like	ENSMUSG00000005373	31492	6,23
Jdp2	Jun dimerization protein 2	ENSMUSG00000034271	40774	6,23
Unc5cl	unc-5 homolog C (C. elegans)-like	ENSMUSG00000043592	84814	6,23
Tbxas1	thromboxane A synthase 1, platelet	ENSMUSG00000029925	209182	6,23
Ctbs	chitobiase, di-N-acetyl-	ENSMUSG00000028189	15380	6,23
Defb41	defensin beta 41	ENSMUSG00000067773	14159	6,23
Kctd21	potassium channel tetramerisation domain containing 21	ENSMUSG00000044952	17887	6,23
Pla2g4a	phospholipase A2, group IVA (cytosolic, calcium-dependent)	ENSMUSG00000056220	131673	6,23
A3galt2	alpha 1,3-galactosyltransferase 2 (isoglobotriaosylceramide synthase)	ENSMUSG00000028794	13935	6,23
Grm3	glutamate receptor, metabotropic 3	ENSMUSG00000003974	240117	6,23
Dusp26	dual specificity phosphatase 26 (putative)	ENSMUSG00000039661	7577	6,16
Alpl	alkaline phosphatase, liver/bone/kidney	ENSMUSG00000028766	54652	6,16
Atp1b2	ATPase, Na+/K+ transporting, beta 2 polypeptide	ENSMUSG00000041329	6207	6,16
Mfsd6l	major facilitator superfamily domain containing 6-like	ENSMUSG00000048329	2059	6,16
Prmt6	protein arginine N-methyltransferase 6	ENSMUSG00000049300	4887	6,16
Serpinb1a	serine (or cysteine) peptidase inhibitor, clade B, member 1a	ENSMUSG00000044734	9094	6,16
Ttc30a1	tetratricopeptide repeat domain 30A1	ENSMUSG00000075271	2862	6,16
		ENSMUSG00000097565	35000	6,16
BC025920	cDNA sequence BC025920	ENSMUSG00000074862	14434	6,16
Gpatch3	G patch domain containing 3	ENSMUSG00000028850	9499	6,16
Guk1	guanylate kinase 1	ENSMUSG00000020444	8338	6,16
B3galt1	UDP-Gal:betaGlcNAc beta 1,3-galactosyltransferase, polypeptide 1	ENSMUSG00000034780	374478	6,16
Bik	BCL2-interacting killer	ENSMUSG00000016758	17774	6,16
Slc29a4	solute carrier family 29 (nucleoside transporters), member 4	ENSMUSG00000050822	20390	6,16
Zfp708	zinc finger protein 708	ENSMUSG00000058883	28577	6,16
		ENSMUSG00000052544	519658	6,16
Uxt	ubiquitously expressed transcript	ENSMUSG00000001134	20296	6,16
2310057M21Rik	RIKEN cDNA 2310057M21 gene	ENSMUSG00000040177	19981	6,16
Yy2	Yy2 transcription factor	ENSMUSG00000091736	32704	6,16
		ENSMUSG00000019791	10339	6,16

		ENSMUSG00000096793	156445	6,16
Avil	advillin	ENSMUSG00000025432	20286	6,16
		ENSMUSG00000031340	18805	6,16
Capsl	calcyphosine-like	ENSMUSG00000039676	30008	6,16
		ENSMUSG00000096330	27268	6,16
Hist1h1b	histone cluster 1, H1b	ENSMUSG00000058773	743	6,09
Crlf1	cytokine receptor-like factor 1	ENSMUSG00000007888	10924	6,09
Il17b	interleukin 17B	ENSMUSG00000024578	4603	6,09
Bcl2l2	BCL2-like 2	ENSMUSG00000089682	4858	6,09
Uchl4	ubiquitin carboxyl-terminal esterase L4	ENSMUSG00000035337	1162	6,09
Oaz3	ornithine decarboxylase antizyme 3	ENSMUSG00000028141	3227	6,09
Noxo1	NADPH oxidase organizer 1	ENSMUSG00000019320	4296	6,09
Zscan20	zinc finger and SCAN domains 20	ENSMUSG00000061894	26560	6,09
		ENSMUSG00000011254	8652	6,09
		ENSMUSG00000043313	7131	6,09
Pwp2	PWP2 periodic tryptophan protein homolog (yeast)	ENSMUSG00000032834	14240	6,09
Pgbd1	piggyBac transposable element derived 1	ENSMUSG00000055313	19784	6,09
Hepacam	hepatocyte cell adhesion molecule	ENSMUSG00000046240	19205	6,09
Ssr4	signal sequence receptor, delta	ENSMUSG00000002014	3803	6,09
Evpl	envoplakin	ENSMUSG00000034282	17519	6,09
Cybb	cytochrome b-245, beta polypeptide	ENSMUSG00000015340	52520	6,09
Stambpl1	STAM binding protein like 1	ENSMUSG00000024776	48105	6,09
Pde3a	phosphodiesterase 3A, cGMP inhibited	ENSMUSG00000041741	250083	6,09
3110001I22Rik	RIKEN cDNA 3110001I22 gene	ENSMUSG00000079737	6477	6,09
Aoah	acyloxyacyl hydrolase	ENSMUSG00000021322	230134	6,09
Fam135b	family with sequence similarity 135, member B	ENSMUSG00000036800	282161	6,09
Tex11	testis expressed gene 11	ENSMUSG00000009670	221020	6,09
Gm9990	predicted gene 9990	ENSMUSG00000056106	2086	6,09
B3galt2	UDP-Gal:betaGlcNAc beta 1,3-galactosyltransferase, polypeptide 2	ENSMUSG00000033849	9743	6,09
Acsm1	acyl-CoA synthetase medium-chain family member 1	ENSMUSG00000033533	55490	6,09
Olfr56	olfactory receptor 56	ENSMUSG00000040328	156499	6,09
Col8a2	collagen, type VIII, alpha 2	ENSMUSG00000056174	27538	6,02
Tspan18	tetraspanin 18	ENSMUSG00000027217	132746	6,02
Them6	thioesterase superfamily member 6	ENSMUSG00000056665	3140	6,02
Cpxm2	carboxypeptidase X 2 (M14 family)	ENSMUSG00000030862	111924	6,02
Mypop	Myb-related transcription factor, partner of profilin	ENSMUSG00000048481	10522	6,02
1600002H07Rik	RIKEN cDNA 1600002H07 gene	ENSMUSG00000024118	5798	6,02
Gprasp2	G protein-coupled receptor associated sorting protein 2	ENSMUSG00000072966	5697	6,02
Notch4	notch 4	ENSMUSG00000015468	24236	6,02
Sema4f	sema domain, immunoglobulin domain (Ig), TM domain, and short cytoplasmic domain	ENSMUSG00000000627	27885	6,02
Zfp239	zinc finger protein 239	ENSMUSG00000042097	11192	6,02
Gm10681	predicted gene 10681	ENSMUSG00000095388	36255	6,02

Ddit4l	DNA-damage-inducible transcript 4-like	ENSMUSG00000046818	6722	6,02
Hus1	Hus1 homolog (S. pombe)	ENSMUSG00000020413	18055	6,02
Kcnc3	potassium voltage gated channel, Shaw-related subfamily, member 3	ENSMUSG00000062785	14091	6,02
Ttyh2	tweety homolog 2 (Drosophila)	ENSMUSG00000034714	45547	6,02
Zfp449	zinc finger protein 449	ENSMUSG00000073176	19275	6,02
Zfp697	zinc finger protein 697	ENSMUSG00000050064	49468	6,02
Naalad2	N-acetylated alpha-linked acidic dipeptidase 2	ENSMUSG00000043943	81045	6,02
Zrsr1	zinc finger (CCCH type), RNA binding motif and serine/arginine rich 1	ENSMUSG00000044068	4492	6,02
Gria4	glutamate receptor, ionotropic, AMPA4 (alpha 4)	ENSMUSG00000025892	378336	6,02
Slc10a5	solute carrier family 10 (sodium/bile acid cotransporter family), member 5	ENSMUSG00000058921	3923	6,02
Tor2a	torsin family 2, member A	ENSMUSG00000009563	5011	6,02
A430033K04Rik	RIKEN cDNA A430033K04 gene	ENSMUSG00000056014	26045	6,02
Lin37	lin-37 homolog (C. elegans)	ENSMUSG00000036845	4398	6,02
2610528J11Rik	RIKEN cDNA 2610528J11 gene	ENSMUSG00000028536	3230	6,02
1810062G17Rik	RIKEN cDNA 1810062G17 gene	ENSMUSG00000027713	6363	6,02
Ticrr	TOPBP1-interacting checkpoint and replication regulator	ENSMUSG00000046591	37939	6,02
Liph	lipase, member H	ENSMUSG00000044626	41717	6,02
Ppil3	peptidylprolyl isomerase (cyclophilin)-like 3	ENSMUSG00000026035	14493	6,02
Pkd1l2	polycystic kidney disease 1 like 2	ENSMUSG00000034416	86771	6,02
Apbb3	amyloid beta (A4) precursor protein-binding, family B, member 3	ENSMUSG00000001379	8207	6,02
Zfp54	zinc finger protein 54	ENSMUSG00000023882	12414	6,02
Tekt3	tektin 3	ENSMUSG00000042189	33311	6,02
Rassf5	Ras association (RalGDS/AF-6) domain family member 5	ENSMUSG00000026430	68849	6,02
Kcnf1	potassium voltage-gated channel, subfamily F, member 1	ENSMUSG00000051726	4787	6,02
Slc7a11	solute carrier family 7 (cationic amino acid transporter, y+ system), member 11	ENSMUSG00000027737	78678	6,02
Gprin3	GPRIN family member 3	ENSMUSG00000045441	73830	6,02
Ccdc69	coiled-coil domain containing 69	ENSMUSG00000049588	28401	6,02
Ros1	Ros1 proto-oncogene	ENSMUSG00000019893	149524	6,02
Nfkbie	nuclear factor of kappa light polypeptide gene enhancer in B cells inhibitor, epsilon	ENSMUSG00000023947	7454	6,02
Sap25	sin3 associated polypeptide	ENSMUSG00000079165	1569	6,02
Klb	klotho beta	ENSMUSG00000029195	35618	6,02
Cdca5	cell division cycle associated 5	ENSMUSG00000024791	6795	5,94
Rpusd3	RNA pseudouridylate synthase domain containing 3	ENSMUSG00000051169	4030	5,94
Fbxo44	F-box protein 44	ENSMUSG00000029001	7683	5,94
Gm10259	predicted pseudogene 10259	ENSMUSG00000069083	1957	5,94
Caskin2	CASK-interacting protein 2	ENSMUSG00000034471	14457	5,94

H6pd	hexose-6-phosphate dehydrogenase (glucose 1-dehydrogenase)	ENSMUSG00000028980	29549	5,94
Zfp473	zinc finger protein 473	ENSMUSG00000048012	19570	5,94
Lrrc73	leucine rich repeat containing 73	ENSMUSG00000071073	3152	5,94
Gemin4	gem (nuclear organelle) associated protein 4	ENSMUSG00000049396	7094	5,94
Zfp458	zinc finger protein 458	ENSMUSG00000055480	14151	5,94
Eif4ebp1	eukaryotic translation initiation factor 4E binding protein 1	ENSMUSG00000031490	15330	5,94
Nudt6	nudix (nucleoside diphosphate linked moiety X)-type motif 6	ENSMUSG00000050174	15302	5,94
Ank1	ankyrin 1, erythroid	ENSMUSG00000031543	175654	5,94
Pigt	phosphatidylinositol glycan anchor biosynthesis, class T	ENSMUSG00000017721	10782	5,94
Cep76	centrosomal protein 76	ENSMUSG00000073542	23940	5,94
Rrp15	ribosomal RNA processing 15 homolog (S. cerevisiae)	ENSMUSG00000001305	28381	5,94
Dnph1	2'-deoxynucleoside 5'-phosphate N-hydrolase 1	ENSMUSG00000040658	2830	5,94
		ENSMUSG00000062797	23671	5,94
Vmn1r4	vomeronasal 1 receptor 4	ENSMUSG00000071893	34079	5,94
Tmem258	transmembrane protein 258	ENSMUSG00000036372	3811	5,94
AW146154	expressed sequence AW146154	ENSMUSG00000074166	21011	5,94
Tlr3	toll-like receptor 3	ENSMUSG00000031639	14944	5,94
Pdgfrl	platelet-derived growth factor receptor-like	ENSMUSG00000031595	64574	5,94
		ENSMUSG00000049536	3497	5,94
Itpka	inositol 1,4,5-trisphosphate 3-kinase A	ENSMUSG00000027296	8927	5,94
Wdr16	WD repeat domain 16	ENSMUSG00000020904	40846	5,94
		ENSMUSG00000048334	914	5,94
Isg15	ISG15 ubiquitin-like modifier	ENSMUSG00000035692	1395	5,94
Gm15446	predicted gene 15446	ENSMUSG00000090015	20537	5,94
Raet1e	retinoic acid early transcript 1E	ENSMUSG00000053219	215571	5,94
		ENSMUSG00000094441	13522	5,94
Nt5e	5' nucleotidase, ecto	ENSMUSG00000032420	44481	5,94
Cblc	Casitas B-lineage lymphoma c	ENSMUSG00000040525	17929	5,94
Vill	villin-like	ENSMUSG00000038775	18748	5,94
		ENSMUSG00000051107	15513	5,94
Tigit	T cell immunoreceptor with Ig and ITIM domains	ENSMUSG00000071552	15324	5,94
Lgals4	lectin, galactose binding, soluble 4	ENSMUSG00000053964	7729	5,94
Samsn1	SAM domain, SH3 domain and nuclear localization signals, 1	ENSMUSG00000022876	163478	5,94
Gm1123	predicted gene 1123	ENSMUSG00000044860	28726	5,94
Adh1	alcohol dehydrogenase 1 (class I)	ENSMUSG00000074207	29708	5,87
		ENSMUSG00000019464	5921	5,87
Tstd1	thiosulfate sulfurtransferase (rhodanese)-like domain containing 1	ENSMUSG00000091166	1320	5,87
Gltpd1	glycolipid transfer protein domain containing 1	ENSMUSG00000029073	4718	5,87
		ENSMUSG00000039904	22123	5,87
Kpna2	karyopherin (importin) alpha 2	ENSMUSG00000018362	10913	5,87
		ENSMUSG00000030587	1872	5,87

Ifi30	interferon gamma inducible protein 30	ENSMUSG00000031838	3890	5,87
Zfp870	zinc finger protein 870	ENSMUSG00000095325	6856	5,87
Pla2g15	phospholipase A2, group XV	ENSMUSG00000031903	14315	5,87
		ENSMUSG00000091754	139471	5,87
Kng2	kininogen 2	ENSMUSG00000060459	43248	5,87
Kbtbd12	kelch repeat and BTB (POZ) domain containing 12	ENSMUSG00000033182	79724	5,87
Bag2	BCL2-associated athanogene 2	ENSMUSG00000042215	12311	5,87
Sprr1a	small proline-rich protein 1A	ENSMUSG00000050359	1944	5,87
LOC100861615	alpha takusan-like	ENSMUSG00000079396	196274	5,87
Rhd	Rh blood group, D antigen	ENSMUSG00000028825	31637	5,87
Zfp758	zinc finger protein 758	ENSMUSG00000044501	15829	5,87
1700102P08Rik	RIKEN cDNA 1700102P08 gene	ENSMUSG00000032611	4930	5,87
		ENSMUSG00000031574	6250	5,87
Grm1	glutamate receptor, metabotropic 1	ENSMUSG00000019828	396298	5,87
		ENSMUSG00000060034	7511	5,87
Sbspon	somatomedin B and thrombospondin, type 1 domain containing	ENSMUSG00000032719	38861	5,87
Cyp11a1	cytochrome P450, family 11, subfamily a, polypeptide 1	ENSMUSG00000032323	12008	5,87
Aox4	aldehyde oxidase 4	ENSMUSG00000038242	58201	5,87
Col5a2	collagen, type V, alpha 2	ENSMUSG00000026042	128962	5,80
Ube2c	ubiquitin-conjugating enzyme E2C	ENSMUSG00000001403	8925	5,80
E130012A19Rik	RIKEN cDNA E130012A19 gene	ENSMUSG00000043439	2324	5,80
Kif2c	kinesin family member 2C	ENSMUSG00000028678	23001	5,80
Art5	ADP-ribosyltransferase 5	ENSMUSG00000070424	5967	5,80
Dmpk	dystrophia myotonica-protein kinase	ENSMUSG00000030409	9973	5,80
Abcf2	ATP-binding cassette, sub-family F (GCN20), member 2	ENSMUSG00000028953	12127	5,80
Ankrd37	ankyrin repeat domain 37	ENSMUSG00000050914	2966	5,80
Fgf10	fibroblast growth factor 10	ENSMUSG00000021732	122325	5,80
Scara3	scavenger receptor class A, member 3	ENSMUSG00000034463	34350	5,80
		ENSMUSG00000043192	1413	5,80
Spata25	spermatogenesis associated 25	ENSMUSG00000017767	2149	5,80
Tpra1	transmembrane protein, adipocyte asscociated 1	ENSMUSG00000002871	9988	5,80
Srfbp1	serum response factor binding protein 1	ENSMUSG00000024528	25046	5,80
AU023871	expressed sequence AU023871	ENSMUSG00000073414	3492	5,80
		ENSMUSG00000040612	62685	5,80
Phf1	PHD finger protein 1	ENSMUSG00000024193	4764	5,80
Stat4	signal transducer and activator of transcription 4	ENSMUSG00000062939	120041	5,80
Tmppe	transmembrane protein with metallophosphoesterase domain	ENSMUSG00000079260	10106	5,80
Fam83d	family with sequence similarity 83, member D	ENSMUSG00000027654	18545	5,80
Tmem8b	transmembrane protein 8B	ENSMUSG00000078716	23698	5,80
Rpp38	ribonuclease P/MRP 38 subunit	ENSMUSG00000049950	3695	5,80
Arid3b	AT rich interactive domain 3B (BRIGHT-like)	ENSMUSG00000004661	46441	5,80
Sh3bp2	SH3-domain binding protein 2	ENSMUSG00000054520	37801	5,80

		ENSMUSG00000089923	4557	5,80
Matk	megakaryocyte-associated tyrosine kinase	ENSMUSG00000004933	10431	5,80
Rbfox1	RNA binding protein, fox-1 homolog (C. elegans) 1	ENSMUSG00000008658	603258	5,80
Gm16223	predicted gene 16223	ENSMUSG00000067285	148839	5,80
Mfng	MFNG O-fucosylpeptide 3-beta-N-acetylglucosaminyltransferase	ENSMUSG00000018169	17594	5,73
Car2	carbonic anhydrase 2	ENSMUSG00000027562	14344	5,73
Paqr9	progestin and adipoQ receptor family member IX	ENSMUSG00000064225	2305	5,73
Nxpe4	neurexophilin and PC-esterase domain family, member 4	ENSMUSG00000044229	37985	5,73
		ENSMUSG00000032346	2617	5,73
Zfp719	zinc finger protein 719	ENSMUSG00000030469	13626	5,73
Nhsl2	NHS-like 2	ENSMUSG00000079481	242671	5,73
Skida1	SKI/DACH domain containing 1	ENSMUSG00000054074	8376	5,73
Ttk	Ttk protein kinase	ENSMUSG00000038379	37702	5,73
Bmper	BMP-binding endothelial regulator	ENSMUSG00000031963	262127	5,73
Dlgap5	discs, large (Drosophila) homolog-associated protein 5	ENSMUSG00000037544	30629	5,73
Npas1	neuronal PAS domain protein 1	ENSMUSG00000001988	21060	5,73
Ncaph	non-SMC condensin I complex, subunit H	ENSMUSG00000034906	30146	5,73
Cage1	cancer antigen 1	ENSMUSG00000044566	31018	5,73
Tepp	testis, prostate and placenta expressed	ENSMUSG00000090206	15758	5,73
Rnf135	ring finger protein 135	ENSMUSG00000020707	15907	5,73
		ENSMUSG00000051890	43457	5,73
Vmn2r35	vomeronasal 2, receptor 35	ENSMUSG00000096399	33717	5,73
		ENSMUSG00000057835	13659	5,73
Mcidas	multiciliate differentiation and DNA synthesis associated cell cycle protein	ENSMUSG00000074651	6527	5,73
Cd86	CD86 antigen	ENSMUSG00000022901	62213	5,73
Fcgbp	Fc fragment of IgG binding protein	ENSMUSG00000047730	49627	5,73
Anxa8	annexin A8	ENSMUSG00000021950	14591	5,73
Chmp4c	charged multivesicular body protein 4C	ENSMUSG00000027536	24099	5,73
		ENSMUSG00000067795	27347	5,73
Atp6v1g3	ATPase, H+ transporting, lysosomal V1 subunit G3	ENSMUSG00000026394	15723	5,65
		ENSMUSG00000090460	345	5,65
Jph4	junctophilin 4	ENSMUSG00000022208	10106	5,65
Apitd1	apoptosis-inducing, TAF9-like domain 1	ENSMUSG00000073705	10509	5,65
Spata33	spermatogenesis associated 33	ENSMUSG00000048478	9188	5,65
Aldh1l1	aldehyde dehydrogenase 1 family, member L1	ENSMUSG00000030088	113777	5,65
		ENSMUSG00000026956	5794	5,65
Nfya	nuclear transcription factor-Y alpha	ENSMUSG00000023994	23022	5,65
Ndst2	N-deacetylase/N-sulfotransferase (heparan glucosaminyl) 2	ENSMUSG00000039308	6589	5,65
Rab7l1	RAB7, member RAS oncogene family-like 1	ENSMUSG00000026433	5664	5,65
Pgap3	post-GPI attachment to proteins 3	ENSMUSG00000038208	11814	5,65

Vegfc	vascular endothelial growth factor C	ENSMUSG00000031520	108923	5,65
Doc2b	double C2, beta	ENSMUSG00000020848	27084	5,65
Samd10	sterile alpha motif domain containing 10	ENSMUSG00000038605	3995	5,65
Mlf1ip	myeloid leukemia factor 1 interacting protein	ENSMUSG00000031629	27953	5,65
Gm3086	predicted gene 3086	ENSMUSG00000079076	6249	5,65
Osmr	oncostatin M receptor	ENSMUSG00000022146	61393	5,65
Mb	myoglobin	ENSMUSG00000018893	35182	5,65
Krt15	keratin 15	ENSMUSG00000054146	4171	5,65
Ak5	adenylate kinase 5	ENSMUSG00000039058	205326	5,65
		ENSMUSG00000096910	13548	5,65
Plbd1	phospholipase B domain containing 1	ENSMUSG00000030214	49859	5,65
		ENSMUSG00000003444	6563	5,65
Tll2	tolloid-like 2	ENSMUSG00000025013	122794	5,65
Itgam	integrin alpha M	ENSMUSG00000030786	65521	5,65
Il34	interleukin 34	ENSMUSG00000031750	64096	5,65
Vwc2	von Willebrand factor C domain containing 2	ENSMUSG00000050830	154709	5,65
Caps2	calcyphosphine 2	ENSMUSG00000035694	52935	5,65
Impg1	interphotoreceptor matrix proteoglycan 1	ENSMUSG00000032343	152109	5,65
Higd1c	HIG1 domain family, member 1C	ENSMUSG00000093550	19423	5,65
Apod	apolipoprotein D	ENSMUSG00000022548	18617	5,58
0610010K14Rik	RIKEN cDNA 0610010K14 gene	ENSMUSG00000020831	2709	5,58
Sulf2	sulfatase 2	ENSMUSG00000006800	82575	5,58
Serpina3n	serine (or cysteine) peptidase inhibitor, clade A, member 3N	ENSMUSG00000021091	7601	5,58
Rom1	rod outer segment membrane protein 1	ENSMUSG00000071648	1975	5,58
Ppan	peter pan homolog (Drosophila)	ENSMUSG00000004100	4004	5,58
		ENSMUSG00000044881	3270	5,58
Eno2	enolase 2, gamma neuronal	ENSMUSG00000004267	9621	5,58
Xkr7	X Kell blood group precursor related family member 7 homolog	ENSMUSG00000042631	23924	5,58
		ENSMUSG00000066592	537	5,58
Fpgt	fucose-1-phosphate guanylyltransferase	ENSMUSG00000053870	8458	5,58
Iqcd	IQ motif containing D	ENSMUSG00000029601	18099	5,58
Trip13	thyroid hormone receptor interactor 13	ENSMUSG00000021569	25306	5,58
Adamts7	a disintegrin-like and metallopeptidase (reprolysin type) with thrombospondin type 1 motif, 7	ENSMUSG00000032363	45094	5,58
Vps37d	vacuolar protein sorting 37D (yeast)	ENSMUSG00000043614	5367	5,58
Hdhd3	haloacid dehalogenase-like hydrolase domain containing 3	ENSMUSG00000038422	4460	5,58
Sh3rf3	SH3 domain containing ring finger 3	ENSMUSG00000037990	325558	5,58
		ENSMUSG00000079834	174537	5,58
Cdhr4	cadherin-related family member 4	ENSMUSG00000032595	16398	5,58
Acoxl	acyl-Coenzyme A oxidase-like	ENSMUSG00000027380	284998	5,58
		ENSMUSG00000079104	9471	5,58
Reg1	regenerating islet-derived 1	ENSMUSG00000059654	2684	5,58

1700057G04Rik	RIKEN cDNA 1700057G04 gene	ENSMUSG00000074139	48500	5,58
Igfbp3	insulin-like growth factor binding protein 3	ENSMUSG00000020427	7838	5,51
A930004D18Rik	RIKEN cDNA A930004D18 gene	ENSMUSG00000054057	12545	5,51
		ENSMUSG00000042579	5730	5,51
Tgm2	transglutaminase 2, C polypeptide	ENSMUSG00000037820	30035	5,51
Eif3m	eukaryotic translation initiation factor 3, subunit M	ENSMUSG00000027170	17425	5,51
Daam2	dishevelled associated activator of morphogenesis 2	ENSMUSG00000040260	108316	5,51
Nfkbib	nuclear factor of kappa light polypeptide gene enhancer in B cells inhibitor, beta	ENSMUSG00000030595	9262	5,51
Klf16	Kruppel-like factor 16	ENSMUSG00000035397	10198	5,51
Kcnj2	potassium inwardly-rectifying channel, subfamily J, member 2	ENSMUSG00000041695	10658	5,51
Adamts15	a disintegrin-like and metallopeptidase (reprolysin type) with thrombospondin type 1 motif, 15	ENSMUSG00000033453	23298	5,51
Brat1	BRCA1-associated ATM activator 1	ENSMUSG00000000148	14369	5,51
Fam203a	family with sequence similarity 203, member A	ENSMUSG00000022554	2506	5,51
Xrcc6bp1	XRCC6 binding protein 1	ENSMUSG00000025436	32942	5,51
Zwilch	zwilch kinetochore protein	ENSMUSG00000032400	35961	5,51
Vmn2r60	vomeronasal 2, receptor 60	ENSMUSG00000090619	79306	5,51
Hrg	histidine-rich glycoprotein	ENSMUSG00000022877	10585	5,51
Paqr8	progestin and adipoQ receptor family member VIII	ENSMUSG00000025931	48134	5,51
Cd59b	CD59b antigen	ENSMUSG00000068686	21339	5,51
Stap2	signal transducing adaptor family member 2	ENSMUSG00000038781	8490	5,51
Slc16a3	solute carrier family 16 (monocarboxylic acid transporters), member 3	ENSMUSG00000025161	12389	5,51
Lrrc63	leucine rich repeat containing 63	ENSMUSG00000021997	46579	5,51
Slc8a2	solute carrier family 8 (sodium/calcium exchanger), member 2	ENSMUSG00000030376	30210	5,51
Rbpjl	recombination signal binding protein for immunoglobulin kappa J region-like	ENSMUSG00000017007	12308	5,51
Fam131c	family with sequence similarity 131, member C	ENSMUSG00000006218	15956	5,44
Mmp2	matrix metallopeptidase 2	ENSMUSG00000031740	26090	5,44
		ENSMUSG00000095334	10779	5,44
		ENSMUSG00000062683	4736	5,44
Dynlt1b	dynein light chain Tctex-type 1B	ENSMUSG00000096255	6182	5,44
Fat4	FAT tumor suppressor homolog 4 (Drosophila)	ENSMUSG00000046743	125046	5,44
Rsg1	REM2 and RAB-like small GTPase 1	ENSMUSG00000073733	12801	5,44
Rab34	RAB34, member of RAS oncogene family	ENSMUSG00000002059	3764	5,44

Slc16a10	solute carrier family 16 (monocarboxylic acid transporters), member 10	ENSMUSG00000019838	108720	5,44
Etv4	ets variant gene 4 (E1A enhancer binding protein, E1AF)	ENSMUSG00000017724	15630	5,44
Tradd	TNFRSF1A-associated via death domain	ENSMUSG00000031887	6324	5,44
Parp10	poly (ADP-ribose) polymerase family, member 10	ENSMUSG00000063268	12268	5,44
Accs	1-aminocyclopropane-1-carboxylate synthase (non-functional)	ENSMUSG00000040272	16477	5,44
Piwil2	piwi-like RNA-mediated gene silencing 2	ENSMUSG00000033644	56615	5,44
Ccdc141	coiled-coil domain containing 141	ENSMUSG00000044033	160735	5,44
Sycp1	synaptonemal complex protein 1	ENSMUSG00000027855	117602	5,44
Pigr	polymeric immunoglobulin receptor	ENSMUSG00000026417	25566	5,44
Nxnl2	nucleoredoxin-like 2	ENSMUSG00000021396	4163	5,36
Exo1	exonuclease 1	ENSMUSG00000039748	30619	5,36
Mfi2	antigen p97 (melanoma associated) identified by monoclonal antibodies 133.2 and 96.5	ENSMUSG00000022780	20211	5,36
Pdxp	pyridoxal (pyridoxine, vitamin B6) phosphatase	ENSMUSG00000068221	5599	5,36
Fah	fumarylacetoacetate hydrolase	ENSMUSG00000030630	21564	5,36
Nipsnap3b	nipsnap homolog 3B (C. elegans)	ENSMUSG00000015247	10181	5,36
Nfatc4	nuclear factor of activated T cells, cytoplasmic, calcineurin dependent 4	ENSMUSG00000023411	9149	5,36
Trank1	tetratricopeptide repeat and ankyrin repeat containing 1	ENSMUSG00000062296	84036	5,36
		ENSMUSG00000094634	88012	5,36
Bop1	block of proliferation 1	ENSMUSG00000022557	24274	5,36
Pak4	p21 protein (Cdc42/Rac)-activated kinase 4	ENSMUSG00000030602	39367	5,36
Znhit3	zinc finger, HIT type 3	ENSMUSG00000020526	5417	5,36
Fbn1	fibrillin 1	ENSMUSG00000027204	207400	5,36
AI118078	expressed sequence AI118078	ENSMUSG00000032313	111433	5,36
		ENSMUSG00000039208	14763	5,36
Cacnb1	calcium channel, voltage-dependent, beta 1 subunit	ENSMUSG00000020882	21527	5,36
Pik3c2g	phosphatidylinositol 3-kinase, C2 domain containing, gamma polypeptide	ENSMUSG00000030228	232390	5,36
Nadsyn1	NAD synthetase 1	ENSMUSG00000031090	27266	5,36
Ctso	cathepsin O	ENSMUSG00000028015	24125	5,36
Pabpc1l	poly(A) binding protein, cytoplasmic 1-like	ENSMUSG00000054582	25089	5,36
1700008F21Rik	RIKEN cDNA 1700008F21 gene	ENSMUSG00000056018	116599	5,36
Frem3	Fras1 related extracellular matrix protein 3	ENSMUSG00000042353	84277	5,36
Papln	papilin, proteoglycan-like sulfated glycoprotein	ENSMUSG00000021223	28749	5,36
Fer1l6	fer-1-like 6 (C. elegans)	ENSMUSG00000037106	155045	5,36
Fzd4	frizzled homolog 4 (Drosophila)	ENSMUSG00000049791	5745	5,36

Gsto2	glutathione S-transferase omega 2	ENSMUSG00000025069	20780	5,36
Sytl5	synaptotagmin-like 5	ENSMUSG00000054453	108922	5,36
Gm6792	predicted gene 6792	ENSMUSG00000053367	29357	5,36
		ENSMUSG00000080717	12678	5,36
Mal	myelin and lymphocyte protein, T cell differentiation protein	ENSMUSG00000027375	23470	5,29
Itpripl2	inositol 1,4,5-triphosphate receptor interacting protein-like 2	ENSMUSG00000095115	6862	5,29
		ENSMUSG00000054676	14352	5,29
Nkd1	naked cuticle 1 homolog (Drosophila)	ENSMUSG00000031661	73544	5,29
Rbm43	RNA binding motif protein 43	ENSMUSG00000036249	10716	5,29
1700016D06Rik	RIKEN cDNA 1700016D06 gene	ENSMUSG00000031509	23830	5,29
		ENSMUSG00000038677	32537	5,29
Chac2	ChaC, cation transport regulator 2	ENSMUSG00000020309	9644	5,29
Yars2	tyrosyl-tRNA synthetase 2 (mitochondrial)	ENSMUSG00000022792	6676	5,29
Fndc7	fibronectin type III domain containing 7	ENSMUSG00000045326	36331	5,29
Zfp953	zinc finger protein 953	ENSMUSG00000056552	42664	5,29
Bloc1s1	biogenesis of lysosome-related organelles complex-1, subunit 1	ENSMUSG00000090247	6154	5,29
Fancg	Fanconi anemia, complementation group G	ENSMUSG00000028453	8164	5,29
Arsk	arylsulfatase K	ENSMUSG00000021592	38239	5,29
Casp2	caspase 2	ENSMUSG00000029863	17524	5,29
Vamp3	vesicle-associated membrane protein 3	ENSMUSG00000028955	10664	5,29
Cln5	ceroid-lipofuscinosis, neuronal 5	ENSMUSG00000022125	7364	5,29
Cntn3	contactin 3	ENSMUSG00000030075	301362	5,29
Ttll8	tubulin tyrosine ligase-like family, member 8	ENSMUSG00000022388	63786	5,29
Cd14	CD14 antigen	ENSMUSG00000051439	1663	5,29
Zfp97	zinc finger protein 97	ENSMUSG00000095990	25417	5,29
Cd274	CD274 antigen	ENSMUSG00000016496	20641	5,29
Ankrd13d	ankyrin repeat domain 13 family, member D	ENSMUSG00000005986	12958	5,29
Cdh20	cadherin 20	ENSMUSG00000050840	226953	5,29
Msln	mesothelin	ENSMUSG00000063011	5714	5,22
Hpgd	hydroxyprostaglandin dehydrogenase 15 (NAD)	ENSMUSG00000031613	26495	5,22
N4bp3	NEDD4 binding protein 3	ENSMUSG00000001053	7780	5,22
Fkbp14	FK506 binding protein 14	ENSMUSG00000038074	19741	5,22
Ficd	FIC domain containing	ENSMUSG00000053334	4819	5,22
		ENSMUSG00000057551	6825	5,22
Swsap1	SWIM type zinc finger 7 associated protein 1	ENSMUSG00000051238	2518	5,22
Rps19bp1	ribosomal protein S19 binding protein 1	ENSMUSG00000051518	3693	5,22
Cp	ceruloplasmin	ENSMUSG00000003617	52092	5,22
Chadl	chondroadherin-like	ENSMUSG00000063765	18992	5,22
Ccnf	cyclin F	ENSMUSG00000072082	28178	5,22
		ENSMUSG00000097319	11413	5,22
		ENSMUSG00000090733	891	5,22
Alg10b	asparagine-linked glycosylation 10B (alpha-1,2-glucosyltransferase)	ENSMUSG00000075470	6244	5,22

Neu3	neuraminidase 3	ENSMUSG00000035239	16979	5,22
L2hgdh	L-2-hydroxyglutarate dehydrogenase	ENSMUSG00000020988	34439	5,22
Rab38	RAB38, member of RAS oncogene family	ENSMUSG00000030559	61194	5,22
2310042D19Rik	RIKEN cDNA 2310042D19 gene	ENSMUSG00000078486	5381	5,22
Odf3	outer dense fiber of sperm tails 3	ENSMUSG00000025482	3214	5,22
1700056E22Rik	RIKEN cDNA 1700056E22 gene	ENSMUSG00000044854	967	5,22
Edaradd	EDAR (ectodysplasin-A receptor)-associated death domain	ENSMUSG00000095105	49242	5,22
Kdelr3	KDEL (Lys-Asp-Glu-Leu) endoplasmic reticulum protein retention receptor 3	ENSMUSG00000010830	11332	5,22
Lair1	leukocyte-associated Ig-like receptor 1	ENSMUSG00000055541	56132	5,22
Herc6	hect domain and RLD 6	ENSMUSG00000029798	84145	5,22
Mcmdc2	minichromosome maintenance domain containing 2	ENSMUSG00000046101	33448	5,22
Glis1	GLIS family zinc finger 1	ENSMUSG00000034762	200471	5,22
		ENSMUSG00000079353	48185	5,22
Pcolce	procollagen C-endopeptidase enhancer protein	ENSMUSG00000029718	8682	5,15
Ldb2	LIM domain binding 2	ENSMUSG00000039706	327575	5,15
9330151L19Rik	RIKEN cDNA 9330151L19 gene	ENSMUSG00000097061	2658	5,15
Slc25a1	solute carrier family 25 (mitochondrial carrier, citrate transporter), member 1	ENSMUSG00000003528	3009	5,15
Oxld1	oxidoreductase like domain containing 1	ENSMUSG00000039670	1463	5,15
Zfp111	zinc finger protein 111	ENSMUSG00000087598	17497	5,15
		ENSMUSG00000044763	5211	5,15
Mcm10	minichromosome maintenance deficient 10 (S. cerevisiae)	ENSMUSG00000026669	23078	5,15
Sytl1	synaptotagmin-like 1	ENSMUSG00000028860	10024	5,15
Sertad4	SERTA domain containing 4	ENSMUSG00000016262	11322	5,15
Rassf10	Ras association (RalGDS/AF-6) domain family (N-terminal) member 10	ENSMUSG00000098132	3496	5,15
C230052I12Rik	RIKEN cDNA C230052I12 gene	ENSMUSG00000030493	4543	5,15
Glyctk	glycerate kinase	ENSMUSG00000020258	5282	5,15
Mks1	Meckel syndrome, type 1	ENSMUSG00000034121	10589	5,15
Klhdc9	kelch domain containing 9	ENSMUSG00000045259	2353	5,15
Pcdhb7	protocadherin beta 7	ENSMUSG00000045062	3506	5,15
AA474408	expressed sequence AA474408	ENSMUSG00000073867	3339	5,15
1190002N15Rik	RIKEN cDNA 1190002N15 gene	ENSMUSG00000045414	20218	5,15
Ankrd24	ankyrin repeat domain 24	ENSMUSG00000054708	19071	5,15
		ENSMUSG00000032578	6759	5,15
Entpd3	ectonucleoside triphosphate diphosphohydrolase 3	ENSMUSG00000041608	28501	5,15
Alg12	asparagine-linked glycosylation 12 (alpha-1,6-mannosyltransferase)	ENSMUSG00000035845	14075	5,15
Hspb11	heat shock protein family B (small), member 11	ENSMUSG00000063172	26346	5,15
Ache	acetylcholinesterase	ENSMUSG00000023328	6948	5,15
BC096441	cDNA sequence BC096441	ENSMUSG00000018752	13522	5,15
Podxl2	podocalyxin-like 2	ENSMUSG00000033152	32487	5,15

H2-Ke6	H2-K region expressed gene 6	ENSMUSG00000073422	2028	5,15
Zfp229	zinc finger protein 229	ENSMUSG00000061544	38548	5,15
		ENSMUSG00000007646	28310	5,15
Atp2b2	ATPase, Ca++ transporting, plasma membrane 2	ENSMUSG00000030302	296945	5,15
A430105I19Rik	RIKEN cDNA A430105I19 gene	ENSMUSG00000045838	8504	5,15
Stap1	signal transducing adaptor family member 1	ENSMUSG00000029254	32248	5,15
Lrrc34	leucine rich repeat containing 34	ENSMUSG00000027702	23603	5,15
Mybph	myosin binding protein H	ENSMUSG00000042451	7784	5,15
Sh3gl3	SH3-domain GRB2-like 3	ENSMUSG00000030638	133580	5,15
		ENSMUSG00000025185	18832	5,15
Trpv4	transient receptor potential cation channel, subfamily V, member 4	ENSMUSG00000014158	36270	5,15
Tmprss15	transmembrane protease, serine 15	ENSMUSG00000022857	138090	5,15
Tnfaip3	tumor necrosis factor, alpha-induced protein 3	ENSMUSG00000019850	14748	5,15
Tmem74b	transmembrane protein 74b	ENSMUSG00000044364	10150	5,07
Colec12	collectin sub-family member 12	ENSMUSG00000036103	170348	5,07
Zfp511	zinc finger protein 511	ENSMUSG00000025470	4215	5,07
Batf2	basic leucine zipper transcription factor, ATF-like 2	ENSMUSG00000039699	31494	5,07
Msrb2	methionine sulfoxide reductase B2	ENSMUSG00000023094	23537	5,07
Rsad1	radical S-adenosyl methionine domain containing 1	ENSMUSG00000039096	9458	5,07
Mapk7	mitogen-activated protein kinase 7	ENSMUSG00000001034	5595	5,07
C330011M18Rik	RIKEN cDNA C330011M18 gene	ENSMUSG00000056753	2052	5,07
Tmem198b	transmembrane protein 198b	ENSMUSG00000047090	4335	5,07
Tespa1	thymocyte expressed, positive selection associated 1	ENSMUSG00000034833	39791	5,07
Fbxo17	F-box protein 17	ENSMUSG00000030598	21341	5,07
Fli1	Friend leukemia integration 1	ENSMUSG00000016087	119182	5,07
Cdh19	cadherin 19, type 2	ENSMUSG00000047216	88178	5,07
Car5b	carbonic anhydrase 5b, mitochondrial	ENSMUSG00000031373	51176	5,07
Plvap	plasmalemma vesicle associated protein	ENSMUSG00000034845	14016	5,07
Mlip	muscular LMNA-interacting protein	ENSMUSG00000032355	245787	5,07
Tcerg1l	transcription elongation regulator 1-like	ENSMUSG00000091002	188757	5,07
Dhx37	DEAH (Asp-Glu-Ala-His) box polypeptide 37	ENSMUSG00000029480	19643	5,07
		ENSMUSG00000030806	20650	5,07
A930011G23Rik	RIKEN cDNA A930011G23 gene	ENSMUSG00000089809	431822	5,07
Dyx1c1	dyslexia susceptibility 1 candidate 1 homolog (human)	ENSMUSG00000092192	14280	5,07
Slc19a3	solute carrier family 19, member 3	ENSMUSG00000038496	25926	5,07
Kdm1b	lysine (K)-specific demethylase 1B	ENSMUSG00000038080	41115	5,07
Gli2	GLI-Kruppel family member GLI2	ENSMUSG00000048402	219488	5,07
Slc4a5	solute carrier family 4, sodium bicarbonate cotransporter, member 5	ENSMUSG00000068323	85118	5,07
Etnk2	ethanolamine kinase 2	ENSMUSG00000070644	16765	5,07
Cyp2j13	cytochrome P450, family 2, subfamily j, polypeptide 13	ENSMUSG00000028571	50013	5,07

LOC101055670	HEAT repeat-containing protein 4-like	ENSMUSG00000090843	30354	5,07
Gm11175	predicted gene 11175	ENSMUSG00000080058	543	5,07
Erich2	glutamate rich 2	ENSMUSG00000075302	32066	5,07
Abcb4	ATP-binding cassette, sub-family B (MDR/TAP), member 4	ENSMUSG00000042476	65505	5,07
Kcnk18	potassium channel, subfamily K, member 18	ENSMUSG00000040901	17723	5,07
Plaur	plasminogen activator, urokinase receptor	ENSMUSG00000046223	13374	5,07
Sepn1	selenoprotein N, 1	ENSMUSG00000050989	14275	5,00
Kctd13	potassium channel tetramerisation domain containing 13	ENSMUSG00000030685	16753	5,00
Impa2	inositol (myo)-1(or 4)-monophosphatase 2	ENSMUSG00000024525	29894	5,00
Slc25a19	solute carrier family 25 (mitochondrial thiamine pyrophosphate carrier), member 19	ENSMUSG00000020744	14118	5,00
Slc25a22	solute carrier family 25 (mitochondrial carrier, glutamate), member 22	ENSMUSG00000019082	8154	5,00
Cenpn	centromere protein N	ENSMUSG00000031756	19764	5,00
AI317395	expressed sequence AI317395	ENSMUSG00000038522	23694	5,00
Celf5	CUGBP, Elav-like family member 5	ENSMUSG00000034818	23483	5,00
		ENSMUSG00000002797	10371	5,00
Sp6	trans-acting transcription factor 6	ENSMUSG00000038560	11341	5,00
Fgfrl1	fibroblast growth factor receptor-like 1	ENSMUSG00000008090	12722	5,00
Fbxo15	F-box protein 15	ENSMUSG00000034391	46219	5,00
Tmem169	transmembrane protein 169	ENSMUSG00000026188	18736	5,00
Inpp1	inositol polyphosphate-1-phosphatase	ENSMUSG00000026102	28276	5,00
Hvcn1	hydrogen voltage-gated channel 1	ENSMUSG00000064267	35494	5,00
Nek3	NIMA (never in mitosis gene a)-related expressed kinase 3	ENSMUSG00000031478	38153	5,00
Lama3	laminin, alpha 3	ENSMUSG00000024421	248990	5,00
Wdfy4	WD repeat and FYVE domain containing 4	ENSMUSG00000051506	225962	5,00
Zfyve28	zinc finger, FYVE domain containing 28	ENSMUSG00000037224	93576	5,00
Scml2	sex comb on midleg-like 2 (Drosophila)	ENSMUSG00000000037	141021	5,00
Gm7173	predicted gene 7173	ENSMUSG00000073077	34718	5,00
Vwf	Von Willebrand factor homolog	ENSMUSG00000001930	139906	5,00
Hmha1	histocompatibility (minor) HA-1	ENSMUSG00000035697	14820	5,00
Art3	ADP-ribosyltransferase 3	ENSMUSG00000034842	82802	5,00
Gm216	predicted gene 216	ENSMUSG00000073650	7214	5,00
Icam2	intercellular adhesion molecule 2	ENSMUSG00000001029	10420	5,00
Tusc5	tumor suppressor candidate 5	ENSMUSG00000046275	18857	4,93
Neurod1	neurogenic differentiation 1	ENSMUSG00000034701	4231	4,93
Arxes1	adipocyte-related X-chromosome expressed sequence 1	ENSMUSG00000048355	1580	4,93
Ccnb1	cyclin B1	ENSMUSG00000041431	7921	4,93
Tarbp2	TAR (HIV) RNA binding protein 2	ENSMUSG00000023051	5485	4,93
		ENSMUSG00000097573	2307	4,93
Leprel4	leprecan-like 4	ENSMUSG00000006931	6381	4,93

Kcnk5	potassium channel, subfamily K, member 5	ENSMUSG00000023243	41725	4,93
Eng	endoglin	ENSMUSG00000026814	36075	4,93
		ENSMUSG00000068855	520	4,93
Cd74	CD74 antigen (invariant polypeptide of major histocompatibility complex, class II antigen-associated)	ENSMUSG00000024610	8799	4,93
Dusp23	dual specificity phosphatase 23	ENSMUSG00000026544	2206	4,93
Fermt3	fermitin family homolog 3 (Drosophila)	ENSMUSG00000024965	20512	4,93
Tex30	testis expressed 30	ENSMUSG00000026049	15829	4,93
Sipa1	signal-induced proliferation associated gene 1	ENSMUSG00000056917	12523	4,93
Pts	6-pyruvoyl-tetrahydropterin synthase	ENSMUSG00000032067	7024	4,93
Tfb1m	transcription factor B1, mitochondrial	ENSMUSG00000036983	38451	4,93
Syne4	spectrin repeat containing, nuclear envelope family member 4	ENSMUSG00000019737	4240	4,93
Dyrk1b	dual-specificity tyrosine-(Y)-phosphorylation regulated kinase 1b	ENSMUSG00000002409	7826	4,93
		ENSMUSG00000078546	28523	4,93
		ENSMUSG00000095195	166118	4,93
Foxg1	forkhead box G1	ENSMUSG00000020950	4175	4,93
Smarca1	SWI/SNF related, matrix associated, actin dependent regulator of chromatin, subfamily a, member 1	ENSMUSG00000031099	83607	4,93
Hsbp1l1	heat shock factor binding protein 1-like 1	ENSMUSG00000078963	17345	4,93
		ENSMUSG00000073719	2061	4,93
		ENSMUSG00000074771	60182	4,93
		ENSMUSG00000026203	8340	4,93
Nfkbid	nuclear factor of kappa light polypeptide gene enhancer in B cells inhibitor, delta	ENSMUSG00000036931	7015	4,93
		ENSMUSG00000068631	798	4,93
Pter	phosphotriesterase related	ENSMUSG00000026730	79413	4,93
Chi3l1	chitinase 3-like 1	ENSMUSG00000064246	8006	4,93
Zcwpw1	zinc finger, CW type with PWWP domain 1	ENSMUSG00000037108	34824	4,93
Daf2	decay accelerating factor 2	ENSMUSG00000026401	34473	4,93
Ppef1	protein phosphatase with EF hand calcium-binding domain 1	ENSMUSG00000062168	112672	4,93
		ENSMUSG00000095134	7829	4,93
Gm10545	predicted gene 10545	ENSMUSG00000073594	11811	4,93
Ikbke	inhibitor of kappaB kinase epsilon	ENSMUSG00000042349	25264	4,93
Padi6	peptidyl arginine deiminase, type VI	ENSMUSG00000040935	15289	4,93
		ENSMUSG00000047735	27312	4,93
BC016579	cDNA sequence, BC016579	ENSMUSG00000033187	27271	4,93
		ENSMUSG00000091028	1409	4,93
Tnfrsf19	tumor necrosis factor receptor superfamily, member 19	ENSMUSG00000060548	83022	4,86

Azgp1	alpha-2-glycoprotein 1, zinc	ENSMUSG00000037053	8713	4,86
Col14a1	collagen, type XIV, alpha 1	ENSMUSG00000022371	213054	4,86
		ENSMUSG00000027985	113886	4,86
Zfp580	zinc finger protein 580	ENSMUSG00000055633	2191	4,86
		ENSMUSG00000097509	1569	4,86
Wisp1	WNT1 inducible signaling pathway protein 1	ENSMUSG00000005124	31882	4,86
Bmp1	bone morphogenetic protein 1	ENSMUSG00000022098	45704	4,86
Nell2	NEL-like 2	ENSMUSG00000022454	309256	4,86
Rasa3	RAS p21 protein activator 3	ENSMUSG00000031453	110654	4,86
St8sia4	ST8 alpha-N-acetyl-neuraminide alpha-2,8-sialyltransferase 4	ENSMUSG00000040710	79911	4,86
Mrc2	mannose receptor, C type 2	ENSMUSG00000020695	58497	4,86
Pltp	phospholipid transfer protein	ENSMUSG00000017754	18194	4,86
Alg3	asparagine-linked glycosylation 3 (alpha-1,3-mannosyltransferase)	ENSMUSG00000033809	5318	4,86
Mxd3	Max dimerization protein 3	ENSMUSG00000021485	4591	4,86
Stard8	START domain containing 8	ENSMUSG00000031216	71481	4,86
Tmem136	transmembrane protein 136	ENSMUSG00000048503	7918	4,86
		ENSMUSG00000022840	150861	4,86
Arhgap20	Rho GTPase activating protein 20	ENSMUSG00000053199	88520	4,86
Josd2	Josephin domain containing 2	ENSMUSG00000038695	3673	4,86
Chek2	checkpoint kinase 2	ENSMUSG00000029521	34117	4,86
Abcd1	ATP-binding cassette, sub-family D (ALD), member 1	ENSMUSG00000031378	21938	4,86
Nova1	neuro-oncological ventral antigen 1	ENSMUSG00000021047	124259	4,86
		ENSMUSG00000094685	1266	4,86
Rab27a	RAB27A, member RAS oncogene family	ENSMUSG00000032202	52728	4,86
P4ha3	procollagen-proline, 2-oxoglutarate 4-dioxygenase (proline 4-hydroxylase), alpha polypeptide III	ENSMUSG00000051048	34180	4,86
Kdelc1	KDEL (Lys-Asp-Glu-Leu) containing 1	ENSMUSG00000026047	27346	4,86
Meis3	Meis homeobox 3	ENSMUSG00000041420	11421	4,86
		ENSMUSG00000095362	8546	4,86
Kcnc2	potassium voltage gated channel, Shaw-related subfamily, member 2	ENSMUSG00000035681	195182	4,86
Gm867	predicted gene 867	ENSMUSG00000050157	2952	4,86
1700084C01Rik	RIKEN cDNA 1700084C01 gene	ENSMUSG00000070532	6006	4,86
Kctd4	potassium channel tetramerisation domain containing 4	ENSMUSG00000046523	10215	4,86
		ENSMUSG00000037613	20064	4,86
Cyp2c38	cytochrome P450, family 2, subfamily c, polypeptide 38	ENSMUSG00000032808	73520	4,86
Rfx4	regulatory factor X, 4 (influences HLA class II expression)	ENSMUSG00000020037	150477	4,78
Slc25a24	solute carrier family 25 (mitochondrial carrier, phosphate carrier), member 24	ENSMUSG00000040322	45309	4,78
LOC101056010	uncharacterized LOC101056010	ENSMUSG00000090399	81659	4,78
Aip	aryl-hydrocarbon receptor-interacting protein	ENSMUSG00000024847	11413	4,78
C1galt1c1	C1GALT1-specific chaperone 1	ENSMUSG00000048970	4303	4,78
Tmco6	transmembrane and coiled-coil domains 6	ENSMUSG00000006850	7324	4,78

Ccdc23	coiled-coil domain containing 23	ENSMUSG00000028643	5993	4,78
Myadm	myeloid-associated differentiation marker	ENSMUSG00000068566	10308	4,78
Fmnl1	formin-like 1	ENSMUSG00000055805	27795	4,78
P4ha2	procollagen-proline, 2-oxoglutarate 4-dioxygenase (proline 4-hydroxylase), alpha II polypeptide	ENSMUSG00000018906	31571	4,78
Rtp4	receptor transporter protein 4	ENSMUSG00000033355	4304	4,78
Nup37	nucleoporin 37	ENSMUSG00000035351	31397	4,78
		ENSMUSG00000073791	83591	4,78
Cyp2s1	cytochrome P450, family 2, subfamily s, polypeptide 1	ENSMUSG00000040703	14439	4,78
		ENSMUSG00000078284	1447	4,78
Fat2	FAT tumor suppressor homolog 2 (Drosophila)	ENSMUSG00000055333	85956	4,78
Steap4	STEAP family member 4	ENSMUSG00000012428	21742	4,78
		ENSMUSG00000089996	20233	4,78
Dnase1l1	deoxyribonuclease 1-like 1	ENSMUSG00000019088	9121	4,78
Msh5	mutS homolog 5 (E. coli)	ENSMUSG00000007035	18141	4,78
Dnajc5b	DnaJ (Hsp40) homolog, subfamily C, member 5 beta	ENSMUSG00000027606	102268	4,78
Slitrk6	SLIT and NTRK-like family, member 6	ENSMUSG00000045871	6572	4,78
Ankrd23	ankyrin repeat domain 23	ENSMUSG00000067653	5196	4,78
Ccdc85c	coiled-coil domain containing 85C	ENSMUSG00000084883	69073	4,78
Spata18	spermatogenesis associated 18	ENSMUSG00000029155	28105	4,78
Qrich2	glutamine rich 2	ENSMUSG00000070331	13913	4,78
Synpo	synaptopodin	ENSMUSG00000043079	66170	4,71
Dynlt1a	dynein light chain Tctex-type 1A	ENSMUSG00000092074	6928	4,71
		ENSMUSG00000079242	1280	4,71
Nkapl	NFKB activating protein-like	ENSMUSG00000059395	1455	4,71
		ENSMUSG00000091732	525	4,71
Traip	TRAF-interacting protein	ENSMUSG00000032586	21306	4,71
Ptprb	protein tyrosine phosphatase, receptor type, B	ENSMUSG00000020154	88165	4,71
Zfp13	zinc finger protein 13	ENSMUSG00000062012	23643	4,71
Shpk	sedoheptulokinase	ENSMUSG00000005951	25052	4,71
Acpt	acid phosphatase, testicular	ENSMUSG00000012777	5231	4,71
Adamts13	a disintegrin-like and metallopeptidase (reprolysin type) with thrombospondin type 1 motif, 13	ENSMUSG00000014852	36213	4,71
Gm9797	predicted pseudogene 9797	ENSMUSG00000045455	1182	4,71
Dph2	DPH2 homolog (S. cerevisiae)	ENSMUSG00000028540	3390	4,71
Glp2r	glucagon-like peptide 2 receptor	ENSMUSG00000049928	109652	4,71
Fam72a	family with sequence similarity 72, member A	ENSMUSG00000055184	11970	4,71
Gm5546	predicted gene 5546	ENSMUSG00000022187	15979	4,71
Aste1	asteroid homolog 1 (Drosophila)	ENSMUSG00000032567	10367	4,71
Bend3	BEN domain containing 3	ENSMUSG00000038214	36257	4,71
Itgae	integrin alpha E, epithelial-associated	ENSMUSG00000005947	56864	4,71
BC037034	cDNA sequence BC037034	ENSMUSG00000036948	4391	4,71
Gtf2h3	general transcription factor IIH, polypeptide 3	ENSMUSG00000029387	18541	4,71
Wdr62	WD repeat domain 62	ENSMUSG00000037020	40282	4,71

A730017C20Rik	RIKEN cDNA A730017C20 gene	ENSMUSG00000050875	14715	4,71
		ENSMUSG00000032377	36133	4,71
Gm9994	predicted gene 9994	ENSMUSG00000093805	22803	4,71
Bard1	BRCA1 associated RING domain 1	ENSMUSG00000026196	75649	4,71
Dnaaf3	dynein, axonemal assembly factor 3	ENSMUSG00000055809	9521	4,71
Drd2	dopamine receptor D2	ENSMUSG00000032259	66553	4,71
Cyp2c66	cytochrome P450, family 2, subfamily c, polypeptide 66	ENSMUSG00000067229	73175	4,71
Adam32	a disintegrin and metallopeptidase domain 32	ENSMUSG00000037437	112665	4,71
Slc4a10	solute carrier family 4, sodium bicarbonate cotransporter-like, member 10	ENSMUSG00000026904	280269	4,71
Itgal	integrin alpha L	ENSMUSG00000030830	38878	4,71
Actn2	actinin alpha 2	ENSMUSG00000052374	71335	4,71
		ENSMUSG00000091556	68230	4,71
Smok4a	sperm motility kinase 4A	ENSMUSG00000079711	6978	4,71
Il7	interleukin 7	ENSMUSG00000040329	41351	4,71
Chia	chitinase, acidic	ENSMUSG00000062778	18892	4,71
Olfr481	olfactory receptor 481	ENSMUSG00000054236	939	4,71
1500015O10Rik	RIKEN cDNA 1500015O10 gene	ENSMUSG00000026051	11977	4,64
S100a8	S100 calcium binding protein A8 (calgranulin A)	ENSMUSG00000056054	964	4,64
Syde1	synapse defective 1, Rho GTPase, homolog 1 (C. elegans)	ENSMUSG00000032714	7462	4,64
Mpv17l2	MPV17 mitochondrial membrane protein-like 2	ENSMUSG00000035559	2273	4,64
Gm10615	predicted gene 10615	ENSMUSG00000074056	2214	4,64
Zfp566	zinc finger protein 566	ENSMUSG00000078768	13191	4,64
Ska1	spindle and kinetochore associated complex subunit 1	ENSMUSG00000036223	12520	4,64
Fgfbp3	fibroblast growth factor binding protein 3	ENSMUSG00000047632	2066	4,64
Parvb	parvin, beta	ENSMUSG00000022438	83646	4,64
Rras	Harvey rat sarcoma oncogene, subgroup R	ENSMUSG00000038387	3669	4,64
Dchs1	dachsous 1 (Drosophila)	ENSMUSG00000036862	34666	4,64
H2-T22	histocompatibility 2, T region locus 22	ENSMUSG00000056116	5620	4,64
Xrcc2	X-ray repair complementing defective repair in Chinese hamster cells 2	ENSMUSG00000028933	16014	4,64
Iqgap3	IQ motif containing GTPase activating protein 3	ENSMUSG00000028068	38998	4,64
		ENSMUSG00000027379	30744	4,64
Rassf6	Ras association (RalGDS/AF-6) domain family member 6	ENSMUSG00000029370	37412	4,64
Lrrc46	leucine rich repeat containing 46	ENSMUSG00000020878	6806	4,64
Pde1b	phosphodiesterase 1B, Ca2+-calmodulin dependent	ENSMUSG00000022489	26755	4,64
Myom1	myomesin 1	ENSMUSG00000024049	107336	4,64
Obox3	oocyte specific homeobox 3	ENSMUSG00000066772	160078	4,64
		ENSMUSG00000092004	31952	4,64
Alpk2	alpha-kinase 2	ENSMUSG00000032845	128360	4,64

Muc5ac	mucin 5, subtypes A and C, tracheobronchial/gastric	ENSMUSG00000037974	30260	4,64
Gm9999	predicted gene 9999	ENSMUSG00000056509	4175	4,64
Bpifb6	BPI fold containing family B, member 6	ENSMUSG00000068009	12408	4,57
Gm10642	predicted gene 10642	ENSMUSG00000074213	1610	4,57
Necab3	N-terminal EF-hand calcium binding protein 3	ENSMUSG00000027489	14492	4,57
Dact3	dapper homolog 3, antagonist of beta-catenin (xenopus)	ENSMUSG00000078794	11985	4,57
Cd200	CD200 antigen	ENSMUSG00000022661	26919	4,57
Zfp688	zinc finger protein 688	ENSMUSG00000045251	3102	4,57
Tnfaip8l1	tumor necrosis factor, alpha-induced protein 8-like 1	ENSMUSG00000044469	11479	4,57
Nfatc2ip	nuclear factor of activated T cells, cytoplasmic, calcineurin dependent 2 interacting protein	ENSMUSG00000030722	13884	4,57
Pnma1	paraneoplastic antigen MA1	ENSMUSG00000054383	2359	4,57
Zfp623	zinc finger protein 623	ENSMUSG00000050846	8426	4,57
		ENSMUSG00000036376	2328	4,57
Plxnd1	plexin D1	ENSMUSG00000030123	40195	4,57
Polr3gl	polymerase (RNA) III (DNA directed) polypeptide G like	ENSMUSG00000028104	16310	4,57
Slc2a8	solute carrier family 2, (facilitated glucose transporter), member 8	ENSMUSG00000026791	9094	4,57
9430015G10Rik	RIKEN cDNA 9430015G10 gene	ENSMUSG00000059939	17284	4,57
		ENSMUSG00000030793	1765	4,57
		ENSMUSG00000097328	13522	4,57
Dhodh	dihydroorotate dehydrogenase	ENSMUSG00000031730	17331	4,57
Hspa1l	heat shock protein 1-like	ENSMUSG00000007033	6582	4,57
Ube2t	ubiquitin-conjugating enzyme E2T (putative)	ENSMUSG00000026429	11598	4,57
		ENSMUSG00000090264	1858	4,57
Btbd11	BTB (POZ) domain containing 11	ENSMUSG00000020042	273479	4,57
Ppp1r13l	protein phosphatase 1, regulatory (inhibitor) subunit 13 like	ENSMUSG00000040734	18785	4,57
E130311K13Rik	RIKEN cDNA E130311K13 gene	ENSMUSG00000048581	14789	4,57
Slc16a9	solute carrier family 16 (monocarboxylic acid transporters), member 9	ENSMUSG00000037762	40869	4,57
Clcn2	chloride channel 2	ENSMUSG00000022843	14783	4,57
Rnf113a2	ring finger protein 113A2	ENSMUSG00000098134	1383	4,57
Dennd4b	DENN/MADD domain containing 4B	ENSMUSG00000042404	15485	4,57
Eral1	Era (G-protein)-like 1 (E. coli)	ENSMUSG00000020832	7008	4,57
Cys1	cystin 1	ENSMUSG00000062563	15976	4,57
Lims2	LIM and senescent cell antigen like domains 2	ENSMUSG00000024395	27113	4,57
Fgf1	fibroblast growth factor 1	ENSMUSG00000036585	90732	4,57
As3mt	arsenic (+3 oxidation state) methyltransferase	ENSMUSG00000003559	33653	4,57
		ENSMUSG00000095304	14860	4,57
Tmem91	transmembrane protein 91	ENSMUSG00000061702	6028	4,57
Gm266	predicted gene 266	ENSMUSG00000010529	1213	4,57
Gpr82	G protein-coupled receptor 82	ENSMUSG00000047678	6071	4,57
		ENSMUSG00000043648	3749	4,57

Vil1	villin 1	ENSMUSG00000026175	26184	4,57
Gm10184	predicted pseudogene 10184	ENSMUSG00000066878	1939	4,49
Rftn2	raftlin family member 2	ENSMUSG00000025978	56624	4,49
Coro1a	coronin, actin binding protein 1A	ENSMUSG00000030707	8015	4,49
Nhlrc1	NHL repeat containing 1	ENSMUSG00000044231	2293	4,49
Mrps36	mitochondrial ribosomal protein S36	ENSMUSG00000061474	8705	4,49
Zfp628	zinc finger protein 628	ENSMUSG00000074406	6787	4,49
Gm9833	predicted gene 9833	ENSMUSG00000049230	4392	4,49
Slc46a1	solute carrier family 46, member 1	ENSMUSG00000020829	6363	4,49
Cox6b2	cytochrome c oxidase subunit VIb polypeptide 2	ENSMUSG00000051811	1303	4,49
Cdk15	cyclin-dependent kinase 15	ENSMUSG00000026023	96088	4,49
Sema4a	sema domain, immunoglobulin domain (Ig), transmembrane domain (TM) and short cytoplasmic domain, (semaphorin) 4A	ENSMUSG00000028064	25224	4,49
Mrpl22	mitochondrial ribosomal protein L22	ENSMUSG00000020514	7905	4,49
Rbm12b1	RNA binding motif protein 12 B1	ENSMUSG00000046667	6468	4,49
Nthl1	nth (endonuclease III)-like 1 (E.coli)	ENSMUSG00000041429	6157	4,49
Ppih	peptidyl prolyl isomerase H	ENSMUSG00000060288	20537	4,49
Cep72	centrosomal protein 72	ENSMUSG00000021572	25791	4,49
Tmem86b	transmembrane protein 86B	ENSMUSG00000045282	2441	4,49
Cyb5rl	cytochrome b5 reductase-like	ENSMUSG00000028621	21281	4,49
Lcp2	lymphocyte cytosolic protein 2	ENSMUSG00000002699	45376	4,49
Bcs1l	BCS1-like (yeast)	ENSMUSG00000026172	4155	4,49
Slc16a7	solute carrier family 16 (monocarboxylic acid transporters), member 7	ENSMUSG00000020102	102500	4,49
Fbxo2	F-box protein 2	ENSMUSG00000041556	5804	4,49
Trim65	tripartite motif-containing 65	ENSMUSG00000054517	9283	4,49
		ENSMUSG00000090673	4167	4,49
Cdc42ep1	CDC42 effector protein (Rho GTPase binding) 1	ENSMUSG00000049521	8256	4,49
Disc1	disrupted in schizophrenia 1	ENSMUSG00000043051	207664	4,49
		ENSMUSG00000056900	120526	4,49
		ENSMUSG00000070336	30329	4,49
Murc	muscle-related coiled-coil protein	ENSMUSG00000028348	9989	4,49
Slitrk5	SLIT and NTRK-like family, member 5	ENSMUSG00000033214	8020	4,49
Gm10373	predicted gene 10373	ENSMUSG00000072592	46094	4,49
Gprc5a	G protein-coupled receptor, family C, group 5, member A	ENSMUSG00000046733	19059	4,49
Rasgrp1	RAS guanyl releasing protein 1	ENSMUSG00000027347	63009	4,49
		ENSMUSG00000029636	87427	4,49
		ENSMUSG00000095186	1672	4,49
Cd160	CD160 antigen	ENSMUSG00000038304	30589	4,49
Cd177	CD177 antigen	ENSMUSG00000052212	16327	4,49
Ptpn20	protein tyrosine phosphatase, non-receptor type 20	ENSMUSG00000021940	51485	4,49
Ces1f	carboxylesterase 1F	ENSMUSG00000031725	23512	4,42
Ddr2	discoidin domain receptor family, member 2	ENSMUSG00000026674	138194	4,42
		ENSMUSG00000071265	7211	4,42

Clec11a	C-type lectin domain family 11, member a	ENSMUSG00000004473	3190	4,42
Panx2	pannexin 2	ENSMUSG00000058441	13834	4,42
Zbtb9	zinc finger and BTB domain containing 9	ENSMUSG00000079605	3039	4,42
C1qa	complement component 1, q subcomponent, alpha polypeptide	ENSMUSG00000036887	2887	4,42
Cdkn3	cyclin-dependent kinase inhibitor 3	ENSMUSG00000037628	10985	4,42
Med30	mediator complex subunit 30	ENSMUSG00000038622	17987	4,42
Card14	caspase recruitment domain family, member 14	ENSMUSG00000013483	37608	4,42
		ENSMUSG00000048647	31123	4,42
Efcab10	EF-hand calcium binding domain 10	ENSMUSG00000020562	6416	4,42
Ceacam2	carcinoembryonic antigen-related cell adhesion molecule 2	ENSMUSG00000054385	23963	4,42
Gstm4	glutathione S-transferase, mu 4	ENSMUSG00000027890	4487	4,42
Tert	telomerase reverse transcriptase	ENSMUSG00000021611	22041	4,42
Tfdp1	transcription factor Dp 1	ENSMUSG00000038482	38029	4,42
Fam167a	family with sequence similarity 167, member A	ENSMUSG00000035095	29105	4,42
		ENSMUSG00000026723	35651	4,42
Nod2	nucleotide-binding oligomerization domain containing 2	ENSMUSG00000055994	41160	4,42
Fam194a	family with sequence similarity 194, member A	ENSMUSG00000070471	20908	4,42
Ovgp1	oviductal glycoprotein 1	ENSMUSG00000074340	13713	4,42
Egf	epidermal growth factor	ENSMUSG00000028017	77748	4,42
Nlrp1b	NLR family, pyrin domain containing 1B	ENSMUSG00000070390	77632	4,42
Btg4	B cell translocation gene 4	ENSMUSG00000032056	3700	4,42
		ENSMUSG00000073991	262073	4,42
Apex1	apurinic/apyrimidinic endonuclease 1	ENSMUSG00000035960	2172	4,35
Arxes2	adipocyte-related X-chromosome expressed sequence 2	ENSMUSG00000048040	1540	4,35
Slc39a8	solute carrier family 39 (metal ion transporter), member 8	ENSMUSG00000053897	63294	4,35
Gpr116	G protein-coupled receptor 116	ENSMUSG00000056492	70092	4,35
Tubb6	tubulin, beta 6 class V	ENSMUSG00000001473	12032	4,35
		ENSMUSG00000095710	97144	4,35
Vasn	vasorin	ENSMUSG00000039646	10862	4,35
Sgsh	N-sulfoglucosamine sulfohydrolase (sulfamidase)	ENSMUSG00000005043	12112	4,35
9230110C19Rik	RIKEN cDNA 9230110C19 gene	ENSMUSG00000053070	21152	4,35
BC017158	cDNA sequence BC017158	ENSMUSG00000030780	26792	4,35
Ankrd33b	ankyrin repeat domain 33B	ENSMUSG00000022237	76281	4,35
Ppp1r3b	protein phosphatase 1, regulatory (inhibitor) subunit 3B	ENSMUSG00000046794	12384	4,35
		ENSMUSG00000058152	235046	4,35
4930546H06Rik	RIKEN cDNA 4930546H06 gene	ENSMUSG00000080316	12291	4,35
Agtr1a	angiotensin II receptor, type 1a	ENSMUSG00000049115	46427	4,35
Cd68	CD68 antigen	ENSMUSG00000018774	1941	4,35
Zfp39	zinc finger protein 39	ENSMUSG00000037001	16073	4,35
Cand2	cullin-associated and neddylation-dissociated 2 (putative)	ENSMUSG00000030319	30356	4,35

		ENSMUSG00000021256	13392	4,35
Ptprn	protein tyrosine phosphatase, receptor type, N	ENSMUSG00000026204	17168	4,35
Fam49a	family with sequence similarity 49, member A	ENSMUSG00000020589	114221	4,35
C3ar1	complement component 3a receptor 1	ENSMUSG00000040552	9024	4,35
Scn7a	sodium channel, voltage-gated, type VII, alpha	ENSMUSG00000034810	111490	4,35
Prdm5	PR domain containing 5	ENSMUSG00000029913	157386	4,35
Top3a	topoisomerase (DNA) III alpha	ENSMUSG00000002814	37308	4,35
Slc12a5	solute carrier family 12, member 5	ENSMUSG00000017740	38912	4,35
Gm4788	predicted gene 4788	ENSMUSG00000070594	83605	4,35
Ccdc81	coiled-coil domain containing 81	ENSMUSG00000039391	37482	4,35
		ENSMUSG00000069873	19491	4,35
Scn1a	sodium channel, voltage-gated, type I, alpha	ENSMUSG00000064329	170060	4,35
		ENSMUSG00000097198	3711	4,35
Cd209c	CD209c antigen	ENSMUSG00000040165	14525	4,35
Slc9b1	solute carrier family 9, subfamily B (NHA1, cation proton antiporter 1), member 1	ENSMUSG00000050150	49799	4,35
Chst5	carbohydrate (N-acetylglucosamine 6-O) sulfotransferase 5	ENSMUSG00000031952	21065	4,35
Serpinb3b	serine (or cysteine) peptidase inhibitor, clade B (ovalbumin), member 3B	ENSMUSG00000073602	124422	4,35
Atp13a4	ATPase type 13A4	ENSMUSG00000038094	149012	4,35
Mybphl	myosin binding protein H-like	ENSMUSG00000068745	15146	4,35
Slc39a4	solute carrier family 39 (zinc transporter), member 4	ENSMUSG00000063354	4468	4,35
Ccdc83	coiled-coil domain containing 83	ENSMUSG00000030617	41555	4,35
Aspg	asparaginase homolog (S. cerevisiae)	ENSMUSG00000037686	20891	4,28
4732456N10Rik	RIKEN cDNA 4732456N10 gene	ENSMUSG00000048699	10595	4,28
Tmprss4	transmembrane protease, serine 4	ENSMUSG00000032091	31367	4,28
Eif3g	eukaryotic translation initiation factor 3, subunit G	ENSMUSG00000070319	4249	4,28
2410018M08Rik	RIKEN cDNA 2410018M08 gene	ENSMUSG00000034173	7901	4,28
Atp2c2	ATPase, Ca++ transporting, type 2C, member 2	ENSMUSG00000034112	57710	4,28
Heyl	hairy/enhancer-of-split related with YRPW motif-like	ENSMUSG00000032744	16320	4,28
Cmtr2	cap methyltransferase 2	ENSMUSG00000046441	6530	4,28
1500011B03Rik	RIKEN cDNA 1500011B03 gene	ENSMUSG00000092279	1153	4,28
Polr2d	polymerase (RNA) II (DNA directed) polypeptide D	ENSMUSG00000024258	7484	4,28
Fam136a	family with sequence similarity 136, member A	ENSMUSG00000057497	4375	4,28
Clec10a	C-type lectin domain family 10, member A	ENSMUSG00000000318	14638	4,28
Bst2	bone marrow stromal cell antigen 2	ENSMUSG00000046718	3174	4,28
Timm8a1	translocase of inner mitochondrial membrane 8A1	ENSMUSG00000048007	4610	4,28
Zfp94	zinc finger protein 94	ENSMUSG00000074282	14963	4,28
Adcy1	adenylate cyclase 1	ENSMUSG00000020431	115018	4,28

Nid2	nidogen 2	ENSMUSG00000021806	60531	4,28
Stra6	stimulated by retinoic acid gene 6	ENSMUSG00000032327	90209	4,28
Pcdhb20	protocadherin beta 20	ENSMUSG00000046191	3395	4,28
Gm10655	predicted gene 10655	ENSMUSG00000074256	1397	4,28
Bub1b	budding uninhibited by benzimidazoles 1 homolog, beta (S. cerevisiae)	ENSMUSG00000040084	43381	4,28
Hist4h4	histone cluster 4, H4	ENSMUSG00000096010	2879	4,28
Spdya	speedy homolog A (Xenopus laevis)	ENSMUSG00000052525	37473	4,28
Sidt1	SID1 transmembrane family, member 1	ENSMUSG00000022696	93017	4,28
Inpp5d	inositol polyphosphate-5-phosphatase D	ENSMUSG00000026288	100196	4,28
Gpr19	G protein-coupled receptor 19	ENSMUSG00000032641	28833	4,28
		ENSMUSG00000078500	31182	4,28
Gpam	glycerol-3-phosphate acyltransferase, mitochondrial	ENSMUSG00000024978	29718	4,28
D130062J21Rik	RIKEN cDNA D130062J21 gene	ENSMUSG00000090278	2743	4,28
Rab19	RAB19, member RAS oncogene family	ENSMUSG00000029923	9206	4,28
4930505A04Rik	RIKEN cDNA 4930505A04 gene	ENSMUSG00000040919	45822	4,28
		ENSMUSG00000025955	20071	4,28
Cr2	complement receptor 2	ENSMUSG00000026616	39905	4,28
Otogl	otogelin-like	ENSMUSG00000091455	149912	4,28
Gal3st3	galactose-3-O-sulfotransferase 3	ENSMUSG00000047658	10408	4,20
Gm561	predicted gene 561	ENSMUSG00000074754	1313	4,20
Vkorc1	vitamin K epoxide reductase complex, subunit 1	ENSMUSG00000096145	2551	4,20
Lrrc3	leucine rich repeat containing 3	ENSMUSG00000051652	4962	4,20
Sigmar1	sigma non-opioid intracellular receptor 1	ENSMUSG00000036078	17665	4,20
Snx20	sorting nexin 20	ENSMUSG00000031662	9301	4,20
Dph1	DPH1 homolog (S. cerevisiae)	ENSMUSG00000078789	13599	4,20
Ggta1	glycoprotein galactosyltransferase alpha 1, 3	ENSMUSG00000035778	63053	4,20
Nxt1	NTF2-related export protein 1	ENSMUSG00000036992	3427	4,20
Exoc3l	exocyst complex component 3-like	ENSMUSG00000043251	6175	4,20
Gm9897	predicted gene 9897	ENSMUSG00000053178	5285	4,20
		ENSMUSG00000026575	35303	4,20
Nrg2	neuregulin 2	ENSMUSG00000060275	179674	4,20
Gsdmd	gasdermin D	ENSMUSG00000022575	5066	4,20
Rimkla	ribosomal modification protein rimK-like family member A	ENSMUSG00000048899	27346	4,20
Rhpn1	rhophilin, Rho GTPase binding protein 1	ENSMUSG00000022580	10131	4,20
3110009E18Rik	RIKEN cDNA 3110009E18 gene	ENSMUSG00000026388	67003	4,20
Inpp5j	inositol polyphosphate 5-phosphatase J	ENSMUSG00000034570	10447	4,20
		ENSMUSG00000038168	146497	4,20
Apobec1	apolipoprotein B mRNA editing enzyme, catalytic polypeptide 1	ENSMUSG00000040613	24653	4,20
Prrt3	proline-rich transmembrane protein 3	ENSMUSG00000045009	7724	4,20
Dpf1	D4, zinc and double PHD fingers family 1	ENSMUSG00000030584	13638	4,20

Dzip1	DAZ interacting protein 1	ENSMUSG00000042156	49795	4,20
Nle1	notchless homolog 1 (Drosophila)	ENSMUSG00000020692	7644	4,20
Zfp493	zinc finger protein 493	ENSMUSG00000090659	12759	4,20
Hinfp	histone H4 transcription factor	ENSMUSG00000032119	9999	4,20
Gm13251	predicted gene 13251	ENSMUSG00000070605	21270	4,20
Podnl1	podocan-like 1	ENSMUSG00000012889	6539	4,20
A330008L17Rik	RIKEN cDNA A330008L17 gene	ENSMUSG00000052479	316797	4,20
Gm5934	predicted gene 5934	ENSMUSG00000084063	26453	4,20
Stard6	StAR-related lipid transfer (START) domain containing 6	ENSMUSG00000079608	28613	4,20
Lamb1	laminin B1	ENSMUSG00000002900	64411	4,13
Trp63	transformation related protein 63	ENSMUSG00000022510	208340	4,13
Kcna6	potassium voltage-gated channel, shaker-related, subfamily, member 6	ENSMUSG00000038077	32334	4,13
		ENSMUSG00000063698	24203	4,13
Adtrp	androgen dependent TFPI regulating protein	ENSMUSG00000058022	84537	4,13
Lmo1	LIM domain only 1	ENSMUSG00000036111	31737	4,13
Zfp593	zinc finger protein 593	ENSMUSG00000028840	2304	4,13
		ENSMUSG00000092356	48996	4,13
ENSMUSG00000	predicted gene, ENSMUSG00000022591	ENSMUSG00000022591	2822	4,13
Cdk2	cyclin-dependent kinase 2	ENSMUSG00000025358	7113	4,13
Cpsf4	cleavage and polyadenylation specific factor 4	ENSMUSG00000029625	14829	4,13
Lgals9	lectin, galactose binding, soluble 9	ENSMUSG00000001123	21973	4,13
Mmab	methylmalonic aciduria (cobalamin deficiency) type B homolog (human)	ENSMUSG00000029575	13026	4,13
Pfkfb3	6-phosphofructo-2-kinase/fructose-2,6-biphosphatase 3	ENSMUSG00000026773	30672	4,13
Lynx1	Ly6/neurotoxin 1	ENSMUSG00000022594	5124	4,13
		ENSMUSG00000055900	4304	4,13
Akr1b3	aldo-keto reductase family 1, member B3 (aldose reductase)	ENSMUSG00000001642	13564	4,13
Mcts2	malignant T cell amplified sequence 2	ENSMUSG00000042814	793	4,13
Acacb	acetyl-Coenzyme A carboxylase beta	ENSMUSG00000042010	104227	4,13
Rsad2	radical S-adenosyl methionine domain containing 2	ENSMUSG00000020641	13700	4,13
Lyz2	lysozyme 2	ENSMUSG00000069516	4941	4,13
Smyd5	SET and MYND domain containing 5	ENSMUSG00000033706	14447	4,13
Bst1	bone marrow stromal cell antigen 1	ENSMUSG00000029082	24362	4,13
Gm9996	predicted gene 9996	ENSMUSG00000056316	913	4,13
Arv1	ARV1 homolog (yeast)	ENSMUSG00000031982	11985	4,13
Upp1	uridine phosphorylase 1	ENSMUSG00000020407	18068	4,13
Ly96	lymphocyte antigen 96	ENSMUSG00000025779	21150	4,13
Hnmt	histamine N-methyltransferase	ENSMUSG00000026986	46485	4,13
Mybpc2	myosin binding protein C, fast-type	ENSMUSG00000038670	22971	4,13
Bcas1	breast carcinoma amplified sequence 1	ENSMUSG00000013523	80855	4,13
Mus81	MUS81 endonuclease homolog (yeast)	ENSMUSG00000024906	6058	4,13

Peli3	pellino 3	ENSMUSG00000024901	12477	4,13
Cntnap1	contactin associated protein-like 1	ENSMUSG00000017167	20202	4,13
		ENSMUSG00000061259	71204	4,13
Dffb	DNA fragmentation factor, beta subunit	ENSMUSG00000029027	10678	4,13
Hfm1	HFM1, ATP-dependent DNA helicase homolog (S. cerevisiae)	ENSMUSG00000043410	86130	4,13
		ENSMUSG00000074519	10739	4,13
		ENSMUSG00000050808	18188	4,13
Ggt5	gamma-glutamyltransferase 5	ENSMUSG00000006344	27588	4,13
		ENSMUSG00000050883	26926	4,13
Myo16	myosin XVI	ENSMUSG00000039057	362170	4,13
Zc3h12d	zinc finger CCCH type containing 12D	ENSMUSG00000039981	37928	4,13
Ttc23l	tetratricopeptide repeat domain 23-like	ENSMUSG00000022249	58567	4,13
Tm4sf4	transmembrane 4 superfamily member 4	ENSMUSG00000027801	16364	4,13
Col15a1	collagen, type XV, alpha 1	ENSMUSG00000028339	105007	4,06
5730409E04Rik	RIKEN cDNA 5730409E04Rik gene	ENSMUSG00000073755	4554	4,06
2310022B05Rik	RIKEN cDNA 2310022B05 gene	ENSMUSG00000031983	27604	4,06
		ENSMUSG00000097687	4185	4,06
Gm10401	predicted gene 10401	ENSMUSG00000072693	607	4,06
		ENSMUSG00000090305	813	4,06
Bud13	BUD13 homolog (yeast)	ENSMUSG00000032077	15776	4,06
Khk	ketohexokinase	ENSMUSG00000029162	9691	4,06
Sirt6	sirtuin 6	ENSMUSG00000034748	6013	4,06
Nup43	nucleoporin 43	ENSMUSG00000040034	11379	4,06
Cml1	camello-like 1	ENSMUSG00000057103	5534	4,06
		ENSMUSG00000091418	28392	4,06
Mybl2	myeloblastosis oncogene-like 2	ENSMUSG00000017861	30002	4,06
Wdr55	WD repeat domain 55	ENSMUSG00000042660	3470	4,06
Zfp941	zinc finger protein 941	ENSMUSG00000060314	14730	4,06
Mtrf1	mitochondrial translational release factor 1	ENSMUSG00000022022	25879	4,06
Nradd	neurotrophin receptor associated death domain	ENSMUSG00000032491	3257	4,06
BC037034	cDNA sequence BC037034	ENSMUSG00000091964	4389	4,06
Npr2	natriuretic peptide receptor 2	ENSMUSG00000028469	19310	4,06
		ENSMUSG00000021418	12888	4,06
4933427G17Rik	RIKEN cDNA 4933427G17 gene	ENSMUSG00000030877	32279	4,06
Slc25a42	solute carrier family 25, member 42	ENSMUSG00000002346	27966	4,06
Zc3hav1l	zinc finger CCCH-type, antiviral 1-like	ENSMUSG00000047749	11864	4,06
Phactr3	phosphatase and actin regulator 3	ENSMUSG00000027525	219518	4,06
Inca1	inhibitor of CDK, cyclin A1 interacting protein 1	ENSMUSG00000057054	11795	4,06
Tulp1	tubby like protein 1	ENSMUSG00000037446	13668	4,06
Ifitm10	interferon induced transmembrane protein 10	ENSMUSG00000045777	47917	4,06
Gtpbp6	GTP binding protein 6 (putative)	ENSMUSG00000033434	4223	4,06
Dennd2c	DENN/MADD domain containing 2C	ENSMUSG00000007379	67166	4,06
Gen1	Gen homolog 1, endonuclease (Drosophila)	ENSMUSG00000051235	24862	4,06

Pcbd1	pterin 4 alpha carbinolamine dehydratase/dimerization cofactor of hepatocyte nuclear factor 1 alpha (TCF1) 1	ENSMUSG00000020098	4986	4,06
3110047P20Rik	RIKEN cDNA 3110047P20 gene	ENSMUSG00000090061	161444	4,06
C230029F24Rik	RIKEN cDNA C230029F24 gene	ENSMUSG00000051616	93631	4,06
Fam163a	family with sequence similarity 163, member A	ENSMUSG00000015484	129061	4,06
Gm1604A	predicted gene 1604A	ENSMUSG00000094083	62719	4,06
		ENSMUSG00000050936	9281	4,06
		ENSMUSG00000094393	2190	4,06
		ENSMUSG00000037593	5965	4,06
Fam228a	family with sequence similarity 228, member A	ENSMUSG00000079177	24567	4,06
Il6	interleukin 6	ENSMUSG00000025746	6808	4,06
Fam96b	family with sequence similarity 96, member B	ENSMUSG00000031879	2127	3,99
Cd80	CD80 antigen	ENSMUSG00000075122	28001	3,99
Tomt	transmembrane O-methyltransferase	ENSMUSG00000078630	7987	3,99
Gyltl1b	glycosyltransferase-like 1B	ENSMUSG00000040434	9824	3,99
Zfp87	zinc finger protein 87	ENSMUSG00000097333	10397	3,99
Lect1	leukocyte cell derived chemotaxin 1	ENSMUSG00000022025	24481	3,99
Six5	sine oculis-related homeobox 5	ENSMUSG00000040841	3956	3,99
Zmat5	zinc finger, matrin type 5	ENSMUSG00000009076	32992	3,99
Tatdn3	TatD DNase domain containing 3	ENSMUSG00000026632	17107	3,99
E2f8	E2F transcription factor 8	ENSMUSG00000046179	15168	3,99
Adamts12	a disintegrin-like and metallopeptidase (reprolysin type) with thrombospondin type 1 motif, 12	ENSMUSG00000047497	282078	3,99
Zfp273	zinc finger protein 273	ENSMUSG00000030446	13263	3,99
Drd3	dopamine receptor D3	ENSMUSG00000022705	60712	3,99
Kif18b	kinesin family member 18B	ENSMUSG00000051378	19596	3,99
Kcnu1	potassium channel, subfamily U, member 1	ENSMUSG00000031576	88311	3,99
Ces2h	carboxylesterase 2H	ENSMUSG00000091813	19558	3,99
Tcfl5	transcription factor-like 5 (basic helix-loop-helix)	ENSMUSG00000038932	20753	3,99
		ENSMUSG00000078886	14973	3,99
		ENSMUSG00000060336	22653	3,99
Dus4l	dihydrouridine synthase 4-like (S. cerevisiae)	ENSMUSG00000020648	14772	3,99
Gm5464	predicted gene 5464	ENSMUSG00000075553	2156	3,99
Spsb4	splA/ryanodine receptor domain and SOCS box containing 4	ENSMUSG00000046997	75362	3,99
Dmrt2	doublesex and mab-3 related transcription factor 2	ENSMUSG00000048138	6591	3,99
Icam1	intercellular adhesion molecule 1	ENSMUSG00000037405	12838	3,99
Col4a3	collagen, type IV, alpha 3	ENSMUSG00000079465	135139	3,99
Bmx	BMX non-receptor tyrosine kinase	ENSMUSG00000031377	65352	3,99
Ecm2	extracellular matrix protein 2, female organ and adipocyte specific	ENSMUSG00000043631	27980	3,99
4933428G20Rik	RIKEN cDNA 4933428G20 gene	ENSMUSG00000047988	9558	3,99

B130006D01Rik	RIKEN cDNA B130006D01 gene	ENSMUSG00000075596	3124	3,99
Serpinb1b	serine (or cysteine) peptidase inhibitor, clade B, member 1b	ENSMUSG00000051029	10256	3,99
4932414N04Rik	RIKEN cDNA 4932414N04 gene	ENSMUSG00000079324	91980	3,99
Gm5709	predicted gene 5709	ENSMUSG00000095128	33281	3,99
		ENSMUSG00000027848	15780	3,99
Atp1a2	ATPase, Na+/K+ transporting, alpha 2 polypeptide	ENSMUSG00000007097	26356	3,91
Cyp26b1	cytochrome P450, family 26, subfamily b, polypeptide 1	ENSMUSG00000063415	22495	3,91
Slc2a4	solute carrier family 2 (facilitated glucose transporter), member 4	ENSMUSG00000018566	5650	3,91
Rrs1	RRS1 ribosome biogenesis regulator homolog (S. cerevisiae)	ENSMUSG00000061024	2047	3,91
Chmp6	charged multivesicular body protein 6	ENSMUSG00000025371	6108	3,91
Wscd1	WSC domain containing 1	ENSMUSG00000020811	39728	3,91
		ENSMUSG00000097708	6690	3,91
Cox15	cytochrome c oxidase assembly protein 15	ENSMUSG00000040018	19747	3,91
Nnt	nicotinamide nucleotide transhydrogenase	ENSMUSG00000025453	73550	3,91
Car10	carbonic anhydrase 10	ENSMUSG00000056158	503722	3,91
Naga	N-acetyl galactosaminidase, alpha	ENSMUSG00000022453	9295	3,91
		ENSMUSG00000091900	1773	3,91
Thap8	THAP domain containing 8	ENSMUSG00000013928	10144	3,91
Zfp960	zinc finger protein 960	ENSMUSG00000096696	25516	3,91
		ENSMUSG00000063047	12661	3,91
Fam150b	family with sequence similarity 150, member B	ENSMUSG00000054204	9533	3,91
1810032O08Rik	RIKEN cDNA 1810032O08 gene	ENSMUSG00000020812	4140	3,91
Grin2b	glutamate receptor, ionotropic, NMDA2B (epsilon 2)	ENSMUSG00000030209	443686	3,91
Nox4	NADPH oxidase 4	ENSMUSG00000030562	152615	3,91
Gpr75	G protein-coupled receptor 75	ENSMUSG00000043999	8372	3,91
Unc13d	unc-13 homolog D (C. elegans)	ENSMUSG00000057948	15867	3,91
Gpr1	G protein-coupled receptor 1	ENSMUSG00000046856	31853	3,91
		ENSMUSG00000024274	10581	3,91
Klrb1f	killer cell lectin-like receptor subfamily B member 1F	ENSMUSG00000030154	11571	3,91
Rfpl4	ret finger protein-like 4	ENSMUSG00000035191	7125	3,91
Myl9	myosin, light polypeptide 9, regulatory	ENSMUSG00000067818	6239	3,84
Extl1	exostoses (multiple)-like 1	ENSMUSG00000028838	27479	3,84
B3gnt8	UDP-GlcNAc:betaGal beta-1,3-N-acetylglucosaminyltransferase 8	ENSMUSG00000059479	1867	3,84
Pomc	pro-opiomelanocortin-alpha	ENSMUSG00000020660	5668	3,84
Zfp414	zinc finger protein 414	ENSMUSG00000073423	2690	3,84
Ctsk	cathepsin K	ENSMUSG00000028111	10077	3,84
		ENSMUSG00000095224	1498	3,84
Tsen54	tRNA splicing endonuclease 54 homolog (S. cerevisiae)	ENSMUSG00000020781	8371	3,84
Kcnip3	Kv channel interacting protein 3, calsenilin	ENSMUSG00000079056	65597	3,84
Smtnl2	smoothelin-like 2	ENSMUSG00000045667	22550	3,84

Msl3l2	male-specific lethal 3-like 2 (Drosophila)	ENSMUSG00000047669	9964	3,84
Gpd1	glycerol-3-phosphate dehydrogenase 1 (soluble)	ENSMUSG00000023019	7491	3,84
Osgin2	oxidative stress induced growth inhibitor family member 2	ENSMUSG00000041153	16768	3,84
Megf6	multiple EGF-like-domains 6	ENSMUSG00000057751	104984	3,84
Dnajb5	DnaJ (Hsp40) homolog, subfamily B, member 5	ENSMUSG00000036052	9612	3,84
Slc17a7	solute carrier family 17 (sodium-dependent inorganic phosphate cotransporter), member 7	ENSMUSG00000070570	12218	3,84
Polr2i	polymerase (RNA) II (DNA directed) polypeptide I	ENSMUSG00000019738	1443	3,84
Prmt1	protein arginine N-methyltransferase 1	ENSMUSG00000052429	10504	3,84
Irak3	interleukin-1 receptor-associated kinase 3	ENSMUSG00000020227	60483	3,84
		ENSMUSG00000087236	49889	3,84
Ada	adenosine deaminase	ENSMUSG00000017697	23656	3,84
		ENSMUSG00000095474	20435	3,84
Iqcj	IQ motif containing J	ENSMUSG00000051777	164374	3,84
Ace2	angiotensin I converting enzyme (peptidyl-dipeptidase A) 2	ENSMUSG00000015405	49089	3,84
1190005I06Rik	RIKEN cDNA 1190005I06 gene	ENSMUSG00000043687	39031	3,84
Glt8d2	glycosyltransferase 8 domain containing 2	ENSMUSG00000020251	40218	3,84
Rit2	Ras-like without CAAX 2	ENSMUSG00000057455	343802	3,84
Radil	Ras association and DIL domains	ENSMUSG00000029576	66260	3,84
Proz	protein Z, vitamin K-dependent plasma glycoprotein	ENSMUSG00000031445	14099	3,84
Cox7b2	cytochrome c oxidase subunit VIIb2	ENSMUSG00000049387	105382	3,84
Fam83b	family with sequence similarity 83, member B	ENSMUSG00000032358	55100	3,84
Sel1l2	sel-1 suppressor of lin-12-like 2 (C. elegans)	ENSMUSG00000074764	159852	3,84
Impg2	interphotoreceptor matrix proteoglycan 2	ENSMUSG00000035270	69444	3,84
Gm5087	predicted gene 5087	ENSMUSG00000051729	127251	3,84
Nlrp4c	NLR family, pyrin domain containing 4C	ENSMUSG00000034690	59989	3,84
Ccdc154	coiled-coil domain containing 154	ENSMUSG00000059562	9453	3,84
Ppm1n	protein phosphatase, Mg2+/Mn2+ dependent, 1N (putative)	ENSMUSG00000030402	3243	3,84
Ppt2	palmitoyl-protein thioesterase 2	ENSMUSG00000015474	11849	3,77
Camk2n2	calcium/calmodulin-dependent protein kinase II inhibitor 2	ENSMUSG00000051146	2073	3,77
Rtkn	rhotekin	ENSMUSG00000034930	17117	3,77
1700019G17Rik	RIKEN cDNA 1700019G17 gene	ENSMUSG00000068299	5834	3,77
		ENSMUSG00000040705	2606	3,77
		ENSMUSG00000039164	6497	3,77
Btd	biotinidase	ENSMUSG00000021900	27552	3,77
Adamtsl3	ADAMTS-like 3	ENSMUSG00000070469	278757	3,77
		ENSMUSG00000046675	3802	3,77
		ENSMUSG00000090451	948	3,77

Muc13	mucin 13, epithelial transmembrane	ENSMUSG00000022824	25898	3,77
Gtf2h4	general transcription factor II H, polypeptide 4	ENSMUSG00000001524	6010	3,77
		ENSMUSG00000010051	4784	3,77
Capn6	calpain 6	ENSMUSG00000067276	25184	3,77
Dctpp1	dCTP pyrophosphatase 1	ENSMUSG00000042462	3751	3,77
6430573F11Rik	RIKEN cDNA 6430573F11 gene	ENSMUSG00000039620	56773	3,77
Acadvl	acyl-Coenzyme A dehydrogenase, very long chain	ENSMUSG00000018574	5229	3,77
Rasgrp2	RAS, guanyl releasing protein 2	ENSMUSG00000032946	15877	3,77
		ENSMUSG00000069476	17338	3,77
Tnni3	troponin I, cardiac 3	ENSMUSG00000035458	5925	3,77
		ENSMUSG00000097096	13968	3,77
Folr2	folate receptor 2 (fetal)	ENSMUSG00000032725	5342	3,77
Gm5127	predicted gene 5127	ENSMUSG00000073010	127372	3,77
Fscn2	fascin homolog 2, actin-bundling protein, retinal (Strongylocentrotus purpuratus)	ENSMUSG00000025380	6635	3,77
Samd15	sterile alpha motif domain containing 15	ENSMUSG00000090812	13819	3,77
Kdm8	lysine (K)-specific demethylase 8	ENSMUSG00000030752	17590	3,77
Gm101	predicted gene 101	ENSMUSG00000036962	73894	3,77
		ENSMUSG00000043441	76064	3,77
Catsper3	cation channel, sperm associated 3	ENSMUSG00000021499	24431	3,77
Anxa9	annexin A9	ENSMUSG00000015702	11081	3,77
4930595M18Rik	RIKEN cDNA 4930595M18 gene	ENSMUSG00000060673	38547	3,77
Tac4	tachykinin 4	ENSMUSG00000020872	7737	3,70
Lepre1	leprecan 1	ENSMUSG00000028641	16061	3,70
Bex4	brain expressed gene 4	ENSMUSG00000047844	1448	3,70
Chst15	carbohydrate (N-acetylgalactosamine 4-sulfate 6-O) sulfotransferase 15	ENSMUSG00000030930	81449	3,70
Cd320	CD320 antigen	ENSMUSG00000002308	6684	3,70
Ccdc87	coiled-coil domain containing 87	ENSMUSG00000067872	3163	3,70
Elk1	ELK1, member of ETS oncogene family	ENSMUSG00000009406	17214	3,70
Dna2	DNA replication helicase 2 homolog (yeast)	ENSMUSG00000036875	27160	3,70
		ENSMUSG00000029360	4351	3,70
Tbc1d10c	TBC1 domain family, member 10c	ENSMUSG00000040247	6690	3,70
Tmem203	transmembrane protein 203	ENSMUSG00000078201	914	3,70
		ENSMUSG00000052658	727	3,70
Nit2	nitrilase family, member 2	ENSMUSG00000022751	10662	3,70
Rnf208	ring finger protein 208	ENSMUSG00000044628	2008	3,70
Slco6d1	solute carrier organic anion transporter family, member 6d1	ENSMUSG00000026336	95865	3,70
Pcdhb14	protocadherin beta 14	ENSMUSG00000044043	3438	3,70
Tspan2	tetraspanin 2	ENSMUSG00000027858	37764	3,70
Tpsg1	tryptase gamma 1	ENSMUSG00000033200	5170	3,70
Clcnka	chloride channel Ka	ENSMUSG00000033770	14115	3,70
Tube1	epsilon-tubulin 1	ENSMUSG00000019845	17036	3,70
Tpo	thyroid peroxidase	ENSMUSG00000020673	77966	3,70
Acot11	acyl-CoA thioesterase 11	ENSMUSG00000034853	60444	3,70

Usmg5	upregulated during skeletal muscle growth 5	ENSMUSG00000071528	7155	3,70
		ENSMUSG00000034445	11095	3,70
Prss22	protease, serine, 22	ENSMUSG00000045027	4566	3,70
Gm10113	predicted gene 10113	ENSMUSG00000062282	15621	3,70
Leng9	leukocyte receptor cluster (LRC) member 9	ENSMUSG00000043432	1690	3,70
Il1a	interleukin 1 alpha	ENSMUSG00000027399	10363	3,70
Nlrp3	NLR family, pyrin domain containing 3	ENSMUSG00000032691	25389	3,70
Ncf1	neutrophil cytosolic factor 1	ENSMUSG00000015950	9573	3,70
Olfr786	olfactory receptor 786	ENSMUSG00000095696	939	3,70
Olfr558	olfactory receptor 558	ENSMUSG00000070423	9724	3,70
		ENSMUSG00000028487	400181	3,62
Leprel2	leprecan-like 2	ENSMUSG00000023191	16664	3,62
Sdpr	serum deprivation response	ENSMUSG00000045954	13834	3,62
Smtn	smoothelin	ENSMUSG00000020439	23090	3,62
		ENSMUSG00000054885	110704	3,62
Rgcc	regulator of cell cycle	ENSMUSG00000022018	12890	3,62
Chrnb2	cholinergic receptor, nicotinic, beta polypeptide 2 (neuronal)	ENSMUSG00000027950	11185	3,62
Rbm38	RNA binding motif protein 38	ENSMUSG00000027510	14237	3,62
Clcn1	chloride channel 1	ENSMUSG00000029862	29072	3,62
Cebpa	CCAAT/enhancer binding protein (C/EBP), alpha	ENSMUSG00000034957	2634	3,62
Snta1	syntrophin, acidic 1	ENSMUSG00000027488	31787	3,62
Smoc1	SPARC related modular calcium binding 1	ENSMUSG00000021136	159607	3,62
Cad	carbamoyl-phosphate synthetase 2, aspartate transcarbamylase, and dihydroorotase	ENSMUSG00000013629	23700	3,62
Dcaf12l1	DDB1 and CUL4 associated factor 12-like 1	ENSMUSG00000045284	3628	3,62
Cecr6	cat eye syndrome chromosome region, candidate 6	ENSMUSG00000094626	4869	3,62
Bmp8b	bone morphogenetic protein 8b	ENSMUSG00000002384	26935	3,62
Hspa1b	heat shock protein 1B	ENSMUSG00000090877	2803	3,62
Zfp940	zinc finger protein 940	ENSMUSG00000050855	20050	3,62
Dlk2	delta-like 2 homolog (Drosophila)	ENSMUSG00000047428	5851	3,62
		ENSMUSG00000072714	441	3,62
Sox30	SRY-box containing gene 30	ENSMUSG00000040489	37685	3,62
Tmc1	transmembrane channel-like gene family 1	ENSMUSG00000024749	170745	3,62
Zbtb49	zinc finger and BTB domain containing 49	ENSMUSG00000029127	30723	3,62
Hoga1	4-hydroxy-2-oxoglutarate aldolase 1	ENSMUSG00000025176	25344	3,62
Prlr	prolactin receptor	ENSMUSG00000005268	171943	3,62
		ENSMUSG00000092036	17063	3,62
Mamld1	mastermind-like domain containing 1	ENSMUSG00000059401	105801	3,62
		ENSMUSG00000074357	1665	3,62
Zfp600	zinc finger protein 600	ENSMUSG00000066007	41932	3,62
Vash2	vasohibin 2	ENSMUSG00000037568	31649	3,62
Gorab	golgin, RAB6-interacting	ENSMUSG00000040124	18733	3,62
Arhgap8	Rho GTPase activating protein 8	ENSMUSG00000078954	52156	3,62

Atg16l2	autophagy related 16-like 2 (S. cerevisiae)	ENSMUSG00000047767	12860	3,62
Slc24a1	solute carrier family 24 (sodium/potassium/calcium exchanger), member 1	ENSMUSG00000034452	28747	3,62
		ENSMUSG00000070354	17175	3,62
Obox1	oocyte specific homeobox 1	ENSMUSG00000054310	167919	3,62
Ucma	upper zone of growth plate and cartilage matrix associated	ENSMUSG00000026668	9627	3,62
		ENSMUSG00000095720	61631	3,62
Mtl5	metallothionein-like 5, testis-specific (tesmin)	ENSMUSG00000024905	18967	3,62
		ENSMUSG00000091844	5985	3,62
Olfm3	olfactomedin 3	ENSMUSG00000027965	221645	3,62
Ankrd9	ankyrin repeat domain 9	ENSMUSG00000037904	3688	3,55
Kcna1	potassium voltage-gated channel, shaker-related subfamily, member 1	ENSMUSG00000047976	9339	3,55
Zfp672	zinc finger protein 672	ENSMUSG00000049755	8233	3,55
Fam195a	family with sequence similarity 195, member A	ENSMUSG00000025732	5041	3,55
Cyb5d1	cytochrome b5 domain containing 1	ENSMUSG00000044795	3707	3,55
Fuom	fucose mutarotase	ENSMUSG00000025466	5672	3,55
Emp1	epithelial membrane protein 1	ENSMUSG00000030208	20243	3,55
Pars2	prolyl-tRNA synthetase (mitochondrial)(putative)	ENSMUSG00000043572	4214	3,55
Slc27a3	solute carrier family 27 (fatty acid transporter), member 3	ENSMUSG00000027932	4700	3,55
Zfp202	zinc finger protein 202	ENSMUSG00000025602	21289	3,55
Sez6l2	seizure related 6 homolog like 2	ENSMUSG00000030683	20044	3,55
Gm10762	predicted gene 10762	ENSMUSG00000074807	1133	3,55
Nanp	N-acetylneuraminic acid phosphatase	ENSMUSG00000053916	9714	3,55
Osgepl1	O-sialoglycoprotein endopeptidase-like 1	ENSMUSG00000026096	12720	3,55
Parpbp	PARP1 binding protein	ENSMUSG00000035365	55510	3,55
		ENSMUSG00000074922	855	3,55
Dmkn	dermokine	ENSMUSG00000060962	17311	3,55
		ENSMUSG00000072955	21882	3,55
Dusp4	dual specificity phosphatase 4	ENSMUSG00000031530	12598	3,55
		ENSMUSG00000026922	11361	3,55
		ENSMUSG00000034116	48932	3,55
Ryr1	ryanodine receptor 1, skeletal muscle	ENSMUSG00000030592	121812	3,55
Inhbc	inhibin beta-C	ENSMUSG00000025405	14217	3,55
Shank1	SH3/ankyrin domain gene 1	ENSMUSG00000038738	48099	3,55
Fam101a	family with sequence similarity 101, member A	ENSMUSG00000037962	9101	3,55
Dram1	DNA-damage regulated autophagy modulator 1	ENSMUSG00000020057	56277	3,55
1700019L03Rik	RIKEN cDNA 1700019L03 gene	ENSMUSG00000038987	7048	3,55
Gnrh1	gonadotropin releasing hormone 1	ENSMUSG00000015812	4207	3,55
Fzd7	frizzled homolog 7 (Drosophila)	ENSMUSG00000041075	4801	3,55

Slc6a2	solute carrier family 6 (neurotransmitter transporter, noradrenalin), member 2	ENSMUSG00000055368	41589	3,55
A630095N17Rik	RIKEN cDNA A630095N17 gene	ENSMUSG00000096094	11999	3,55
		ENSMUSG00000098078	59696	3,55
		ENSMUSG00000075014	755	3,55
		ENSMUSG00000096385	3603	3,55
		ENSMUSG00000058260	19013	3,55
		ENSMUSG00000095935	49411	3,55
Col16a1	collagen, type XVI, alpha 1	ENSMUSG00000040690	51444	3,48
Fgfr3	fibroblast growth factor receptor 3	ENSMUSG00000054252	15345	3,48
Aatk	apoptosis-associated tyrosine kinase	ENSMUSG00000025375	39855	3,48
		ENSMUSG00000055676	3581	3,48
Acpl2	acid phosphatase-like 2	ENSMUSG00000043587	69326	3,48
Grhpr	glyoxylate reductase/hydroxypyruvate reductase	ENSMUSG00000035637	9340	3,48
		ENSMUSG00000023919	11997	3,48
Lypd6	LY6/PLAUR domain containing 6	ENSMUSG00000050447	127141	3,48
Plekhs1	pleckstrin homology domain containing, family S member 1	ENSMUSG00000035818	25093	3,48
Chrna10	cholinergic receptor, nicotinic, alpha polypeptide 10	ENSMUSG00000066279	5448	3,48
Aldh1b1	aldehyde dehydrogenase 1 family, member B1	ENSMUSG00000035561	5583	3,48
Rslcan18	regulator of sex-limitation candidate 18	ENSMUSG00000074824	17416	3,48
Ltk	leukocyte tyrosine kinase	ENSMUSG00000027297	9112	3,48
Lix1	limb expression 1 homolog (chicken)	ENSMUSG00000047786	56716	3,48
Gsta2	glutathione S-transferase, alpha 2 (Yc2)	ENSMUSG00000057933	24771	3,48
Spdl1	spindle apparatus coiled-coil protein 1	ENSMUSG00000069910	24452	3,48
Mdh1b	malate dehydrogenase 1B, NAD (soluble)	ENSMUSG00000025963	31500	3,48
Pcp4	Purkinje cell protein 4	ENSMUSG00000090223	58188	3,48
		ENSMUSG00000026452	43850	3,48
Pigz	phosphatidylinositol glycan anchor biosynthesis, class Z	ENSMUSG00000045625	12196	3,48
		ENSMUSG00000037845	5113	3,48
4930518I15Rik	RIKEN cDNA 4930518I15 gene	ENSMUSG00000074629	1331	3,48
Nkx6-2	NK6 homeobox 2	ENSMUSG00000041309	3421	3,48
4632434I11Rik	RIKEN cDNA 4632434I11 gene	ENSMUSG00000030641	16685	3,48
Setmar	SET domain without mariner transposase fusion	ENSMUSG00000034639	12078	3,48
Pla2g5	phospholipase A2, group V	ENSMUSG00000041193	64239	3,48
Gtse1	G two S phase expressed protein 1	ENSMUSG00000022385	16866	3,48
2610034B18Rik	RIKEN cDNA 2610034B18 gene	ENSMUSG00000039043	9999	3,48
Fam228b	family with sequence similarity 228, member B	ENSMUSG00000050545	23052	3,48
		ENSMUSG00000078590	6291	3,48
Tmem40	transmembrane protein 40	ENSMUSG00000059900	33336	3,48
Corin	corin	ENSMUSG00000005220	204449	3,48
Isg20	interferon-stimulated protein	ENSMUSG00000039236	6939	3,48

Ccnb3	cyclin B3	ENSMUSG00000051592	61968	3,48
Tha1	threonine aldolase 1	ENSMUSG00000017713	5530	3,48
Pram1	PML-RAR alpha-regulated adaptor molecule 1	ENSMUSG00000032739	7651	3,48
Popdc3	popeye domain containing 3	ENSMUSG00000019848	140355	3,48
Ugt3a1	UDP glycosyltransferases 3 family, polypeptide A1	ENSMUSG00000072664	41714	3,48
Lrrc19	leucine rich repeat containing 19	ENSMUSG00000049799	13492	3,48
		ENSMUSG00000078161	5631	3,48
Clvs2	clavesin 2	ENSMUSG00000019785	112484	3,48
		ENSMUSG00000079716	38704	3,48
Spink13	serine peptidase inhibitor, Kazal type 13	ENSMUSG00000073551	133848	3,48
Il1rn	interleukin 1 receptor antagonist	ENSMUSG00000026981	14642	3,48
		ENSMUSG00000095547	4186	3,48
Il1r2	interleukin 1 receptor, type II	ENSMUSG00000026073	40452	3,48
		ENSMUSG00000075217	35431	3,48
Nmrk2	nicotinamide riboside kinase 2	ENSMUSG00000004939	3868	3,48
Cyp2b19	cytochrome P450, family 2, subfamily b, polypeptide 19	ENSMUSG00000066704	15468	3,48
Spp2	secreted phosphoprotein 2	ENSMUSG00000026295	19478	3,48
Fabp7	fatty acid binding protein 7, brain	ENSMUSG00000019874	3570	3,41
Acot1	acyl-CoA thioesterase 1	ENSMUSG00000072949	8169	3,41
Diras1	DIRAS family, GTP-binding RAS-like 1	ENSMUSG00000043670	6074	3,41
Padi1	peptidyl arginine deiminase, type I	ENSMUSG00000025329	32796	3,41
		ENSMUSG00000052504	320621	3,41
		ENSMUSG00000043487	10696	3,41
Timm21	tranlocase of inner mitochondrial membrane 21	ENSMUSG00000024645	4231	3,41
Zfp457	zinc finger protein 457	ENSMUSG00000055341	13963	3,41
Tubg2	tubulin, gamma 2	ENSMUSG00000045007	5881	3,41
Atp6v0d2	ATPase, H+ transporting, lysosomal V0 subunit D2	ENSMUSG00000028238	45765	3,41
		ENSMUSG00000097778	8666	3,41
Fpr3	formyl peptide receptor 3	ENSMUSG00000079700	83826	3,41
Pnma3	paraneoplastic antigen MA3	ENSMUSG00000046287	3404	3,41
		ENSMUSG00000063447	1090	3,41
Zfp946	zinc finger protein 946	ENSMUSG00000071266	32468	3,41
Gjb2	gap junction protein, beta 2	ENSMUSG00000046352	6103	3,41
Zfp146	zinc finger protein 146	ENSMUSG00000037029	8454	3,41
Otoa	otoancorin	ENSMUSG00000034990	79776	3,41
		ENSMUSG00000078632	52643	3,41
Tyr	tyrosinase	ENSMUSG00000004651	66007	3,41
Ism2	isthmin 2 homolog (zebrafish)	ENSMUSG00000050671	21068	3,41
Rinl	Ras and Rab interactor-like	ENSMUSG00000051735	9995	3,41
Kcnd3	potassium voltage-gated channel, Shal-related family, member 3	ENSMUSG00000040896	221673	3,41
Acad12	acyl-Coenzyme A dehydrogenase family, member 12	ENSMUSG00000042647	20655	3,41
Il17rb	interleukin 17 receptor B	ENSMUSG00000015966	12756	3,41
		ENSMUSG00000090697	19474	3,41
Mcf2	mcf.2 transforming sequence	ENSMUSG00000031139	123134	3,41
St8sia1	ST8 alpha-N-acetyl-neuraminide alpha-2,8-sialyltransferase 1	ENSMUSG00000030283	142908	3,41
Tacr3	tachykinin receptor 3	ENSMUSG00000028172	105575	3,41

Foxq1	forkhead box Q1	ENSMUSG00000038415	2801	3,41
Rarres1	retinoic acid receptor responder (tazarotene induced) 1	ENSMUSG00000049404	36638	3,41
		ENSMUSG00000091575	1425	3,41
Zp3r	zona pellucida 3 receptor	ENSMUSG00000042554	52909	3,41
Hsh2d	hematopoietic SH2 domain containing	ENSMUSG00000062007	11328	3,41
Atp1a4	ATPase, Na+/K+ transporting, alpha 4 polypeptide	ENSMUSG00000007107	34902	3,41
Olfr658	olfactory receptor 658	ENSMUSG00000070421	4426	3,41
Cyp1a1	cytochrome P450, family 1, subfamily a, polypeptide 1	ENSMUSG00000032315	6210	3,33
Gpt	glutamic pyruvic transaminase, soluble	ENSMUSG00000022546	2911	3,33
Mfap2	microfibrillar-associated protein 2	ENSMUSG00000060572	5567	3,33
		ENSMUSG00000054850	3190	3,33
		ENSMUSG00000056856	143247	3,33
Nyap1	neuronal tyrosine-phosphorylated phosphoinositide 3-kinase adaptor 1	ENSMUSG00000045348	10725	3,33
		ENSMUSG00000062588	1468	3,33
Ptgfrn	prostaglandin F2 receptor negative regulator	ENSMUSG00000027864	70047	3,33
Magohb	mago-nashi homolog B (Drosophila)	ENSMUSG00000030188	8857	3,33
A230050P20Rik	RIKEN cDNA A230050P20 gene	ENSMUSG00000038884	5666	3,33
Dnajc12	DnaJ (Hsp40) homolog, subfamily C, member 12	ENSMUSG00000036764	26398	3,33
Gm11696	predicted gene 11696	ENSMUSG00000056687	8877	3,33
Ccne1	cyclin E1	ENSMUSG00000002068	9551	3,33
Pax3	paired box gene 3	ENSMUSG00000004872	95868	3,33
Dok3	docking protein 3	ENSMUSG00000035711	5304	3,33
Cpne5	copine V	ENSMUSG00000024008	81248	3,33
Nckap1l	NCK associated protein 1 like	ENSMUSG00000022488	44976	3,33
Adat2	adenosine deaminase, tRNA-specific 2	ENSMUSG00000019808	10483	3,33
Glipr2	GLI pathogenesis-related 2	ENSMUSG00000028480	21718	3,33
Tmem255a	transmembrane protein 255A	ENSMUSG00000036502	55867	3,33
Polm	polymerase (DNA directed), mu	ENSMUSG00000020474	10157	3,33
Vmn2r38	vomeronasal 2, receptor 38	ENSMUSG00000095773	23086	3,33
Gm19426	predicted gene, 19426	ENSMUSG00000097187	1487	3,33
Opn1mw	opsin 1 (cone pigments), medium-wave-sensitive (color blindness, deutan)	ENSMUSG00000031394	23297	3,33
Lpin3	lipin 3	ENSMUSG00000027412	25333	3,33
Ptprq	protein tyrosine phosphatase, receptor type, Q	ENSMUSG00000035916	202668	3,33
Rasa4	RAS p21 protein activator 4	ENSMUSG00000004952	27945	3,33
Enpp6	ectonucleotide pyrophosphatase/phosphodiesterase 6	ENSMUSG00000038173	107971	3,33
Mab21l1	mab-21-like 1 (C. elegans)	ENSMUSG00000056947	2776	3,33
9630014M24Rik	RIKEN cDNA 9630014M24 gene	ENSMUSG00000073576	44389	3,33
H60b	histocompatibility 60b	ENSMUSG00000075297	130264	3,33
Ephb6	Eph receptor B6	ENSMUSG00000029869	15028	3,33
Gm11627	predicted gene 11627	ENSMUSG00000078640	3064	3,33

4932418E24Rik	RIKEN cDNA 4932418E24 gene	ENSMUSG00000048038	22912	3,33
C030039L03Rik	RIKEN cDNA C030039L03 gene	ENSMUSG00000057093	17145	3,33
Lat2	linker for activation of T cells family, member 2	ENSMUSG00000040751	14756	3,33
Slc26a8	solute carrier family 26, member 8	ENSMUSG00000036196	52205	3,33
Upk2	uroplakin 2	ENSMUSG00000041523	2052	3,33
Gm5724	predicted gene 5724	ENSMUSG00000084927	65693	3,33
Abcb11	ATP-binding cassette, sub-family B (MDR/TAP), member 11	ENSMUSG00000027048	104335	3,33
Hao1	hydroxyacid oxidase 1, liver	ENSMUSG00000027261	57008	3,33
Mrgpra2b	MAS-related GPR, member A2B	ENSMUSG00000096719	65057	3,33
3930402G23Rik	RIKEN cDNA 3930402G23 gene	ENSMUSG00000038917	4031	3,33
Fmod	fibromodulin	ENSMUSG00000041559	11024	3,26
		ENSMUSG00000037548	8247	3,26
Metrn	meteorin, glial cell differentiation regulator	ENSMUSG00000002274	2566	3,26
Rhoj	ras homolog gene family, member J	ENSMUSG00000046768	93135	3,26
Susd2	sushi domain containing 2	ENSMUSG00000006342	7390	3,26
		ENSMUSG00000055305	6408	3,26
C1ra	complement component 1, r subcomponent A	ENSMUSG00000055172	10809	3,26
Ftsj2	FtsJ homolog 2 (E. coli)	ENSMUSG00000029557	4225	3,26
Arhgef39	Rho guanine nucleotide exchange factor (GEF) 39	ENSMUSG00000051517	3554	3,26
Zfp213	zinc finger protein 213	ENSMUSG00000071256	7458	3,26
Sowahb	sosondowah ankyrin repeat domain family member B	ENSMUSG00000045314	3900	3,26
Spata9	spermatogenesis associated 9	ENSMUSG00000021590	31230	3,26
Apobec2	apolipoprotein B mRNA editing enzyme, catalytic polypeptide 2	ENSMUSG00000040694	13498	3,26
Nefh	neurofilament, heavy polypeptide	ENSMUSG00000020396	9311	3,26
Slc16a1	solute carrier family 16 (monocarboxylic acid transporters), member 1	ENSMUSG00000032902	19795	3,26
		ENSMUSG00000094130	477	3,26
Angptl2	angiopoietin-like 2	ENSMUSG00000004105	31649	3,26
Pramef8	PRAME family member 8	ENSMUSG00000046862	8666	3,26
Ppie	peptidylprolyl isomerase E (cyclophilin E)	ENSMUSG00000028651	12837	3,26
		ENSMUSG00000042918	6545	3,26
Samd1	sterile alpha motif domain containing 1	ENSMUSG00000079003	2715	3,26
P2rx7	purinergic receptor P2X, ligand-gated ion channel, 7	ENSMUSG00000029468	47522	3,26
Rltpr	RGD motif, leucine rich repeats, tropomodulin domain and proline-rich containing	ENSMUSG00000050357	7273	3,26
Rfc4	replication factor C (activator 1) 4	ENSMUSG00000022881	13795	3,26
Ly86	lymphocyte antigen 86	ENSMUSG00000021423	73692	3,26
Slc5a2	solute carrier family 5 (sodium/glucose cotransporter), member 2	ENSMUSG00000030781	6774	3,26
Slc14a2	solute carrier family 14 (urea transporter), member 2	ENSMUSG00000024552	62155	3,26
		ENSMUSG00000020226	53468	3,26

Grik4	glutamate receptor, ionotropic, kainate 4	ENSMUSG00000032017	426359	3,26
Cct6b	chaperonin containing Tcp1, subunit 6b (zeta)	ENSMUSG00000020698	45072	3,26
Gm884	predicted gene 884	ENSMUSG00000034239	86564	3,26
2010109A12Rik	RIKEN cDNA 2010109A12 gene	ENSMUSG00000029384	6958	3,26
1700123I01Rik	RIKEN cDNA 1700123I01 gene	ENSMUSG00000024786	39838	3,26
Bpifb9b	BPI fold containing family B, member 9B	ENSMUSG00000067996	13420	3,26
Gm9869	predicted gene 9869	ENSMUSG00000052143	16346	3,26
Gtsf1	gametocyte specific factor 1	ENSMUSG00000022487	28066	3,26
Gm5627	predicted gene 5627	ENSMUSG00000093812	17038	3,26
Ifi27l2a	interferon, alpha-inducible protein 27 like 2A	ENSMUSG00000079017	1514	3,19
Adora2b	adenosine A2b receptor	ENSMUSG00000018500	17470	3,19
Frem1	Fras1 related extracellular matrix protein 1	ENSMUSG00000059049	154420	3,19
Amacr	alpha-methylacyl-CoA racemase	ENSMUSG00000022244	14869	3,19
Ribc2	RIB43A domain with coiled-coils 2	ENSMUSG00000022431	12471	3,19
Bglap3	bone gamma-carboxyglutamate protein 3	ENSMUSG00000074489	4128	3,19
Tgds	TDP-glucose 4,6-dehydratase	ENSMUSG00000022130	20855	3,19
Kcnab3	potassium voltage-gated channel, shaker-related subfamily, beta member 3	ENSMUSG00000018470	6785	3,19
Pex10	peroxisomal biogenesis factor 10	ENSMUSG00000029047	5418	3,19
Cercam	cerebral endothelial cell adhesion molecule	ENSMUSG00000039787	13677	3,19
Tex29	testis expressed 29	ENSMUSG00000031512	15288	3,19
Scnn1g	sodium channel, nonvoltage-gated 1 gamma	ENSMUSG00000000216	33997	3,19
Abca17	ATP-binding cassette, sub-family A (ABC1), member 17	ENSMUSG00000035435	86771	3,19
		ENSMUSG00000049233	903	3,19
Igsf21	immunoglobulin superfamily, member 21	ENSMUSG00000040972	219939	3,19
		ENSMUSG00000092137	112941	3,19
Zfp82	zinc finger protein 82	ENSMUSG00000098022	18412	3,19
		ENSMUSG00000064247	5985	3,19
Trmt5	TRM5 tRNA methyltransferase 5	ENSMUSG00000034442	6302	3,19
Hist1h4i	histone cluster 1, H4i	ENSMUSG00000060639	537	3,19
		ENSMUSG00000059897	2638	3,19
Fbxl8	F-box and leucine-rich repeat protein 8	ENSMUSG00000033313	4679	3,19
Zkscan2	zinc finger with KRAB and SCAN domains 2	ENSMUSG00000030757	20934	3,19
Thumpd2	THUMP domain containing 2	ENSMUSG00000024246	38759	3,19
Slc5a6	solute carrier family 5 (sodium-dependent vitamin transporter), member 6	ENSMUSG00000006641	12889	3,19
BC051142	cDNA sequence BC051142	ENSMUSG00000057246	61915	3,19
Cntn6	contactin 6	ENSMUSG00000030092	370617	3,19
Tcea3	transcription elongation factor A (SII), 3	ENSMUSG00000001604	27170	3,19
		ENSMUSG00000078866	13397	3,19

Tmeff2	transmembrane protein with EGF-like and two follistatin-like domains 2	ENSMUSG00000026109	286624	3,19
		ENSMUSG00000094620	20101	3,19
Cyp2b10	cytochrome P450, family 2, subfamily b, polypeptide 10	ENSMUSG00000030483	29005	3,19
		ENSMUSG00000060441	24709	3,19
Rep15	RAB15 effector protein	ENSMUSG00000040121	982	3,19
Zan	zonadhesin	ENSMUSG00000079173	98428	3,19
Helz2	helicase with zinc finger 2, transcriptional coactivator	ENSMUSG00000027580	14413	3,19
Myo15b	myosin XVB	ENSMUSG00000034427	30992	3,19
S100a4	S100 calcium binding protein A4	ENSMUSG00000001020	2276	3,12
Aoc3	amine oxidase, copper containing 3	ENSMUSG00000019326	11334	3,12
Lama4	laminin, alpha 4	ENSMUSG00000019846	144674	3,12
Cdh15	cadherin 15	ENSMUSG00000031962	19024	3,12
Copz2	coatomer protein complex, subunit zeta 2	ENSMUSG00000018672	11334	3,12
Cabp4	calcium binding protein 4	ENSMUSG00000024842	4185	3,12
		ENSMUSG00000094430	5718	3,12
Paox	polyamine oxidase (exo-N4-amino)	ENSMUSG00000025464	8678	3,12
		ENSMUSG00000044022	4662	3,12
Ldb1	LIM domain binding 1	ENSMUSG00000025223	12622	3,12
Ugt1a7c	UDP glucuronosyltransferase 1 family, polypeptide A7C	ENSMUSG00000090124	124941	3,12
Nab2	Ngfi-A binding protein 2	ENSMUSG00000025402	7651	3,12
		ENSMUSG00000096687	9221	3,12
		ENSMUSG00000089675	131192	3,12
Tle2	transducin-like enhancer of split 2, homolog of Drosophila E(spl)	ENSMUSG00000034771	16285	3,12
Cdnf	cerebral dopamine neurotrophic factor	ENSMUSG00000039496	13347	3,12
Ppp1r3d	protein phosphatase 1, regulatory subunit 3D	ENSMUSG00000049999	3267	3,12
Nanos1	nanos homolog 1 (Drosophila)	ENSMUSG00000072437	3927	3,12
Mterf	mitochondrial transcription termination factor	ENSMUSG00000040429	3353	3,12
Hexim2	hexamethylene bis-acetamide inducible 2	ENSMUSG00000043372	7448	3,12
Vax2	ventral anterior homeobox containing gene 2	ENSMUSG00000034777	27041	3,12
Zfp691	zinc finger protein 691	ENSMUSG00000045268	4683	3,12
Chst10	carbohydrate sulfotransferase 10	ENSMUSG00000026080	34284	3,12
Asb5	ankyrin repeat and SOCs box-containing 5	ENSMUSG00000031519	37506	3,12
Napsa	napsin A aspartic peptidase	ENSMUSG00000002204	14431	3,12
Ltbp2	latent transforming growth factor beta binding protein 2	ENSMUSG00000002020	93321	3,12
Clic3	chloride intracellular channel 3	ENSMUSG00000015093	1939	3,12
Gm9845	predicted pseudogene 9845	ENSMUSG00000050533	1735	3,12
AI429214	expressed sequence AI429214	ENSMUSG00000074384	1957	3,12
		ENSMUSG00000024339	12795	3,12
Lxn	latexin	ENSMUSG00000047557	5929	3,12

Pkdrej	polycystic kidney disease (polycystin) and REJ (sperm receptor for egg jelly homolog, sea urchin)	ENSMUSG00000052496	7058	3,12
Chek1	checkpoint kinase 1	ENSMUSG00000032113	18584	3,12
		ENSMUSG00000029594	81470	3,12
Gpr146	G protein-coupled receptor 146	ENSMUSG00000044197	18674	3,12
Zswim2	zinc finger SWIM-type containing 2	ENSMUSG00000034552	26150	3,12
		ENSMUSG00000078896	12187	3,12
Tbkbp1	TBK1 binding protein 1	ENSMUSG00000038517	15325	3,12
Gas2l2	growth arrest-specific 2 like 2	ENSMUSG00000020686	7554	3,12
Spred3	sprouty-related, EVH1 domain containing 3	ENSMUSG00000037239	11013	3,12
Ptprh	protein tyrosine phosphatase, receptor type, H	ENSMUSG00000035429	55430	3,12
Sult2a2	sulfotransferase family 2A, dehydroepiandrosterone (DHEA)-preferring, member 2	ENSMUSG00000070811	46132	3,12
Grxcr1	glutaredoxin, cysteine rich 1	ENSMUSG00000068082	134564	3,12
Mas1	MAS1 oncogene	ENSMUSG00000068037	27065	3,12
Tmem196	transmembrane protein 196	ENSMUSG00000048004	72745	3,12
F2	coagulation factor II	ENSMUSG00000027249	11095	3,12
Syt10	synaptotagmin X	ENSMUSG00000063260	59468	3,12
Vmn2r56	vomeronasal 2, receptor 56	ENSMUSG00000090762	39108	3,12
Cpxm1	carboxypeptidase X 1 (M14 family)	ENSMUSG00000027408	6800	3,04
Mme	membrane metallo endopeptidase	ENSMUSG00000027820	86360	3,04
Stbd1	starch binding domain 1	ENSMUSG00000047963	3529	3,04
Dact1	dapper homolog 1, antagonist of beta-catenin (xenopus)	ENSMUSG00000044548	10224	3,04
		ENSMUSG00000090165	163617	3,04
Kti12	KTI12 homolog, chromatin associated (S. cerevisiae)	ENSMUSG00000073775	1556	3,04
Dleu7	deleted in lymphocytic leukemia, 7	ENSMUSG00000048281	16749	3,04
Shf	Src homology 2 domain containing F	ENSMUSG00000033256	20271	3,04
Apcdd1	adenomatosis polyposis coli down-regulated 1	ENSMUSG00000071847	30869	3,04
Zxdb	zinc finger, X-linked, duplicated B	ENSMUSG00000073062	5623	3,04
Pald1	phosphatase domain containing, paladin 1	ENSMUSG00000020092	63867	3,04
		ENSMUSG00000017667	13996	3,04
Lrrc2	leucine rich repeat containing 2	ENSMUSG00000032495	32522	3,04
Nudt10	nudix (nucleoside diphosphate linked moiety X)-type motif 10	ENSMUSG00000073293	4320	3,04
Crtac1	cartilage acidic protein 1	ENSMUSG00000042401	148747	3,04
Exoc3l4	exocyst complex component 3-like 4	ENSMUSG00000021280	13753	3,04
Tmem25	transmembrane protein 25	ENSMUSG00000002032	5532	3,04
Diras2	DIRAS family, GTP-binding RAS-like 2	ENSMUSG00000047842	26462	3,04
Fsd1	fibronectin type 3 and SPRY domain-containing protein	ENSMUSG00000011589	10371	3,04
Usp35	ubiquitin specific peptidase 35	ENSMUSG00000035713	22641	3,04
Epha2	Eph receptor A2	ENSMUSG00000006445	28145	3,04
Zfp874a	zinc finger protein 874a	ENSMUSG00000069206	27076	3,04
BC117090	cDNA sequence BC1179090	ENSMUSG00000079594	12668	3,04

		ENSMUSG00000093979	21335	3,04
Hdac10	histone deacetylase 10	ENSMUSG00000062906	5394	3,04
Ybx2	Y box protein 2	ENSMUSG00000018554	5810	3,04
Tectb	tectorin beta	ENSMUSG00000024979	15581	3,04
		ENSMUSG00000004371	9803	3,04
Anpep	alanyl (membrane) aminopeptidase	ENSMUSG00000039062	26408	3,04
Gm3252	predicted gene 3252	ENSMUSG00000094628	20719	3,04
		ENSMUSG00000027071	38880	3,04
Frmpd1	FERM and PDZ domain containing 1	ENSMUSG00000035615	101062	3,04
		ENSMUSG00000028631	52475	3,04
Mov10l1	Moloney leukemia virus 10-like 1	ENSMUSG00000015365	72244	3,04
Gabrg1	gamma-aminobutyric acid (GABA) A receptor, subunit gamma 1	ENSMUSG00000001260	91571	3,04
Rnd1	Rho family GTPase 1	ENSMUSG00000054855	14041	3,04
Clec3b	C-type lectin domain family 3, member b	ENSMUSG00000025784	6485	3,04
Skint11	selection and upkeep of intraepithelial T cells 11	ENSMUSG00000057977	81645	3,04
Lipm	lipase, family member M	ENSMUSG00000056078	21745	3,04
Plac9b	placenta specific 9b	ENSMUSG00000072674	14486	3,04
Pkhd1l1	polycystic kidney and hepatic disease 1-like 1	ENSMUSG00000038725	139591	3,04
Serpinf2	serine (or cysteine) peptidase inhibitor, clade F, member 2	ENSMUSG00000038224	7860	3,04
D830014E11Rik	RIKEN cDNA D830014E11 gene	ENSMUSG00000045750	7924	3,04
Ascl1	achaete-scute complex homolog 1 (Drosophila)	ENSMUSG00000020052	2842	2,97
Ppp1r18	protein phosphatase 1, regulatory subunit 18	ENSMUSG00000034595	10004	2,97
Rnf183	ring finger protein 183	ENSMUSG00000063851	7713	2,97
Maged2	melanoma antigen, family D, 2	ENSMUSG00000025268	7976	2,97
Wfdc3	WAP four-disulfide core domain 3	ENSMUSG00000076434	14752	2,97
Echdc2	enoyl Coenzyme A hydratase domain containing 2	ENSMUSG00000028601	13872	2,97
Nr1h3	nuclear receptor subfamily 1, group H, member 3	ENSMUSG00000002108	18774	2,97
Zfp748	zinc finger protein 748	ENSMUSG00000095432	14498	2,97
Ube2l6	ubiquitin-conjugating enzyme E2L 6	ENSMUSG00000027078	11508	2,97
Reck	reversion-inducing-cysteine-rich protein with kazal motifs	ENSMUSG00000028476	69277	2,97
Prdm1	PR domain containing 1, with ZNF domain	ENSMUSG00000038151	91325	2,97
Fam65a	family with sequence similarity 65, member A	ENSMUSG00000038604	16966	2,97
Itprip	inositol 1,4,5-triphosphate receptor interacting protein	ENSMUSG00000079254	24702	2,97
Hr	hairless	ENSMUSG00000022096	21337	2,97
Depdc1b	DEP domain containing 1B	ENSMUSG00000021697	91451	2,97
		ENSMUSG00000097585	1100	2,97
Mageb4	melanoma antigen, family B, 4	ENSMUSG00000035427	54840	2,97
Slc8b1	solute carrier family 8 (sodium/lithium/calcium exchanger), member B1	ENSMUSG00000032754	22857	2,97
Hck	hemopoietic cell kinase	ENSMUSG00000003283	42974	2,97

Zfp85-rs1	zinc finger protein 85, related sequence 1	ENSMUSG00000058331	7429	2,97
Spaca5	sperm acrosome associated 5	ENSMUSG00000037167	9558	2,97
Ldhc	lactate dehydrogenase C	ENSMUSG00000030851	16940	2,97
Rex2	reduced expression 2	ENSMUSG00000067919	38945	2,97
Zfp174	zinc finger protein 174	ENSMUSG00000054939	11613	2,97
Pfkfb1	6-phosphofructo-2-kinase/fructose-2,6-biphosphatase 1	ENSMUSG00000025271	55650	2,97
Stac2	SH3 and cysteine rich domain 2	ENSMUSG00000017400	16840	2,97
		ENSMUSG00000093938	17205	2,97
Vmn2r118	vomeronasal 2, receptor 118	ENSMUSG00000091504	32332	2,97
Dmbx1	diencephalon/mesencephalon homeobox 1	ENSMUSG00000028707	25383	2,97
Zmynd15	zinc finger, MYND-type containing 15	ENSMUSG00000040829	6770	2,97
Gdf9	growth differentiation factor 9	ENSMUSG00000018238	6882	2,97
		ENSMUSG00000049109	215459	2,97
Cxcl10	chemokine (C-X-C motif) ligand 10	ENSMUSG00000034855	2252	2,97
Ephx3	epoxide hydrolase 3	ENSMUSG00000037577	5780	2,97
		ENSMUSG00000073962	10059	2,97
Lgals1	lectin, galactose binding, soluble 1	ENSMUSG00000068220	3741	2,90
Tmem86a	transmembrane protein 86A	ENSMUSG00000010307	4137	2,90
Ptgr1	prostaglandin reductase 1	ENSMUSG00000028378	21681	2,90
Ccl20	chemokine (C-C motif) ligand 20	ENSMUSG00000026166	2402	2,90
Caly	calcyon neuron-specific vesicular protein	ENSMUSG00000025468	12669	2,90
Sult6b1	sulfotransferase family, cytosolic, 6B, member 1	ENSMUSG00000038045	23055	2,90
Nsl1	NSL1, MIND kinetochore complex component, homolog (S. cerevisiae)	ENSMUSG00000062510	21547	2,90
Arrdc2	arrestin domain containing 2	ENSMUSG00000002910	4566	2,90
Gal3st1	galactose-3-O-sulfotransferase 1	ENSMUSG00000049721	15691	2,90
Gm12253	predicted gene 12253	ENSMUSG00000058287	9064	2,90
Gapdhs	glyceraldehyde-3-phosphate dehydrogenase, spermatogenic	ENSMUSG00000061099	13907	2,90
Angptl6	angiopoietin-like 6	ENSMUSG00000038742	5919	2,90
Stab1	stabilin 1	ENSMUSG00000042286	29629	2,90
Psrc1	proline/serine-rich coiled-coil 1	ENSMUSG00000068744	4393	2,90
Nxt2	nuclear transport factor 2-like export factor 2	ENSMUSG00000042271	12923	2,90
Ssc5d	scavenger receptor cysteine rich domain containing (5 domains)	ENSMUSG00000035279	18978	2,90
		ENSMUSG00000051220	15376	2,90
BC030867	cDNA sequence BC030867	ENSMUSG00000034773	16306	2,90
Pabpc4l	poly(A) binding protein, cytoplasmic 4-like	ENSMUSG00000090919	5745	2,90
Calr4	calreticulin 4	ENSMUSG00000028558	20087	2,90
Adamts16	a disintegrin-like and metallopeptidase (reprolysin type) with thrombospondin type 1 motif, 16	ENSMUSG00000049538	114010	2,90
Samd5	sterile alpha motif domain containing 5	ENSMUSG00000060487	47950	2,90
Cbr3	carbonyl reductase 3	ENSMUSG00000022947	7776	2,90
Mtap7d3	MAP7 domain containing 3	ENSMUSG00000067878	24469	2,90

Gsg1l	GSG1-like	ENSMUSG00000046182	203993	2,90
2210418O10Rik	RIKEN cDNA 2210418O10 gene	ENSMUSG00000078894	13760	2,90
Ccdc89	coiled-coil domain containing 89	ENSMUSG00000044362	2349	2,90
Cacna2d4	calcium channel, voltage-dependent, alpha 2/delta subunit 4	ENSMUSG00000041460	115882	2,90
		ENSMUSG00000073771	6507	2,90
Pex16	peroxisomal biogenesis factor 16	ENSMUSG00000027222	6542	2,90
Irgm2	immunity-related GTPase family M member 2	ENSMUSG00000069874	23162	2,90
B3galnt1	UDP-GalNAc:betaGlcNAc beta 1,3-galactosaminyltransferase, polypeptide 1	ENSMUSG00000043300	24676	2,90
Ccdc110	coiled-coil domain containing 110	ENSMUSG00000071104	9527	2,90
Fads3	fatty acid desaturase 3	ENSMUSG00000024664	18124	2,90
Sirpb1b	signal-regulatory protein beta 1B	ENSMUSG00000095028	79312	2,90
		ENSMUSG00000079697	6498	2,90
Pappa2	pappalysin 2	ENSMUSG00000073530	268764	2,90
Acr	acrosin prepropeptide	ENSMUSG00000022622	6260	2,90
Trim34b	tripartite motif-containing 34B	ENSMUSG00000090215	36680	2,90
		ENSMUSG00000097570	140191	2,90
Lct	lactase	ENSMUSG00000026354	43563	2,90
Btk	Bruton agammaglobulinemia tyrosine kinase	ENSMUSG00000031264	41235	2,90
Fam154a	family with sequence similarity 154, member A	ENSMUSG00000028492	113688	2,90
Cyp2t4	cytochrome P450, family 2, subfamily t, polypeptide 4	ENSMUSG00000078787	4955	2,90
Fabp12	fatty acid binding protein 12	ENSMUSG00000027530	56975	2,90
Psg16	pregnancy specific glycoprotein 16	ENSMUSG00000066760	59411	2,90
Plac8l1	PLAC8-like 1	ENSMUSG00000059455	18035	2,90
A930009A15Rik	RIKEN cDNA A930009A15 gene	ENSMUSG00000092210	12463	2,90
Casq2	calsequestrin 2	ENSMUSG00000027861	60100	2,90
Dupd1	dual specificity phosphatase and pro isomerase domain containing 1	ENSMUSG00000063821	37995	2,90
Tomm20l	translocase of outer mitochondrial membrane 20 homolog (yeast)-like	ENSMUSG00000021078	11795	2,90
Hsd17b13	hydroxysteroid (17-beta) dehydrogenase 13	ENSMUSG00000034528	21971	2,90
Ces1b	carboxylesterase 1B	ENSMUSG00000078964	23291	2,90
Reg4	regenerating islet-derived family, member 4	ENSMUSG00000027876	14593	2,90
L3mbtl1	l(3)mbt-like (Drosophila)	ENSMUSG00000035576	31051	2,90
Olfr69	olfactory receptor 69	ENSMUSG00000058662	4318	2,90
Igfbp7	insulin-like growth factor binding protein 7	ENSMUSG00000036256	58806	2,83
Col9a3	collagen, type IX, alpha 3	ENSMUSG00000027570	24400	2,83
Pdlim2	PDZ and LIM domain 2	ENSMUSG00000022090	13464	2,83
		ENSMUSG00000079442	12604	2,83
Pth1r	parathyroid hormone 1 receptor	ENSMUSG00000032492	21599	2,83
Palmd	palmdelphin	ENSMUSG00000033377	50732	2,83
Cox8b	cytochrome c oxidase subunit VIIIb	ENSMUSG00000025488	1504	2,83
Cyp2u1	cytochrome P450, family 2, subfamily u, polypeptide 1	ENSMUSG00000027983	12737	2,83
		ENSMUSG00000039476	46270	2,83
		ENSMUSG00000090175	148198	2,83
		ENSMUSG00000021504	10548	2,83

Elovl7	ELOVL family member 7, elongation of long chain fatty acids (yeast)	ENSMUSG00000021696	72702	2,83
Lrrtm1	leucine rich repeat transmembrane neuronal 1	ENSMUSG00000060780	15103	2,83
Ccdc74a	coiled-coil domain containing 74A	ENSMUSG00000041617	4269	2,83
Syndig1	synapse differentiation inducing 1	ENSMUSG00000074736	175182	2,83
Urb2	URB2 ribosome biogenesis 2 homolog (S. cerevisiae)	ENSMUSG00000031976	26998	2,83
B230217C12Rik	RIKEN cDNA B230217C12 gene	ENSMUSG00000089676	2264	2,83
Grin3b	glutamate receptor, ionotropic, NMDA3B	ENSMUSG00000035745	6476	2,83
Hectd2	HECT domain containing 2	ENSMUSG00000041180	66497	2,83
Fgr	Gardner-Rasheed feline sarcoma viral (Fgr) oncogene homolog	ENSMUSG00000028874	27816	2,83
		ENSMUSG00000049091	1359	2,83
		ENSMUSG00000024136	3025	2,83
Oprd1	opioid receptor, delta 1	ENSMUSG00000050511	33761	2,83
Fam151b	family with sequence similarity 151, member B	ENSMUSG00000034334	34410	2,83
Sytl4	synaptotagmin-like 4	ENSMUSG00000031255	45428	2,83
Gria2	glutamate receptor, ionotropic, AMPA2 (alpha 2)	ENSMUSG00000033981	117856	2,83
Edar	ectodysplasin-A receptor	ENSMUSG00000003227	74866	2,83
Megf10	multiple EGF-like-domains 10	ENSMUSG00000024593	164378	2,83
4930579F01Rik	RIKEN cDNA 4930579F01 gene	ENSMUSG00000012042	22579	2,83
Aldh1l2	aldehyde dehydrogenase 1 family, member L2	ENSMUSG00000020256	46691	2,83
Gpr156	G protein-coupled receptor 156	ENSMUSG00000046961	91032	2,83
		ENSMUSG00000039070	24050	2,83
Aqp2	aquaporin 2	ENSMUSG00000023013	5490	2,83
		ENSMUSG00000089951	12168	2,83
Cxcr5	chemokine (C-X-C motif) receptor 5	ENSMUSG00000047880	44604	2,83
		ENSMUSG00000031230	10661	2,83
Map3k8	mitogen-activated protein kinase kinase kinase 8	ENSMUSG00000024235	21689	2,83
Neil1	nei endonuclease VIII-like 1 (E. coli)	ENSMUSG00000032298	3779	2,83
Mylk3	myosin light chain kinase 3	ENSMUSG00000031698	62043	2,83
Sspo	SCO-spondin	ENSMUSG00000029797	53022	2,83
		ENSMUSG00000041660	51864	2,83
		ENSMUSG00000095556	18838	2,83
		ENSMUSG00000091558	6871	2,83
Tlr8	toll-like receptor 8	ENSMUSG00000040522	21634	2,83
4921539E11Rik	RIKEN cDNA 4921539E11 gene	ENSMUSG00000028520	60419	2,83
		ENSMUSG00000026220	29228	2,83
Gm15401	predicted gene 15401	ENSMUSG00000072999	8430	2,83
		ENSMUSG00000000263	93961	2,83
1700034E13Rik	RIKEN cDNA 1700034E13 gene	ENSMUSG00000024532	17525	2,83
		ENSMUSG00000017493	13149	2,75
Ttyh1	tweety homolog 1 (Drosophila)	ENSMUSG00000030428	17299	2,75
		ENSMUSG00000028597	6572	2,75
Paqr6	progestin and adipoQ receptor family member VI	ENSMUSG00000041423	3958	2,75
Zfp365	zinc finger protein 365	ENSMUSG00000037855	26560	2,75

Man1c1	mannosidase, alpha, class 1C, member 1	ENSMUSG00000037306	142601	2,75
Kcnq2	potassium voltage-gated channel, subfamily Q, member 2	ENSMUSG00000016346	59713	2,75
Crtap	cartilage associated protein	ENSMUSG00000032431	15542	2,75
		ENSMUSG00000025481	2949	2,75
Pla2g3	phospholipase A2, group III	ENSMUSG00000034579	11067	2,75
Acd	adrenocortical dysplasia	ENSMUSG00000038000	2937	2,75
		ENSMUSG00000048921	5407	2,75
Asb4	ankyrin repeat and SOCS box-containing 4	ENSMUSG00000042607	49637	2,75
Eif5a2	eukaryotic translation initiation factor 5A2	ENSMUSG00000050192	17536	2,75
Gm9933	predicted gene 9933	ENSMUSG00000054044	1718	2,75
LOC101056064	uncharacterized LOC101056064	ENSMUSG00000090363	21023	2,75
Il10ra	interleukin 10 receptor, alpha	ENSMUSG00000032089	15313	2,75
Inhba	inhibin beta-A	ENSMUSG00000041324	15361	2,75
4933417A18Rik	RIKEN cDNA 4933417A18 gene	ENSMUSG00000021415	31467	2,75
Scrn2	secernin 2	ENSMUSG00000020877	4021	2,75
Lpar6	lysophosphatidic acid receptor 6	ENSMUSG00000033446	2468	2,75
Pdp2	pyruvate dehyrogenase phosphatase catalytic subunit 2	ENSMUSG00000048371	5369	2,75
AW551984	expressed sequence AW551984	ENSMUSG00000038112	17008	2,75
Gabrr3	gamma-aminobutyric acid (GABA) receptor, rho 3	ENSMUSG00000074991	54558	2,75
Emc9	ER membrane protein complex subunit 9	ENSMUSG00000022217	3787	2,75
		ENSMUSG00000091604	3315	2,75
Tmem198	transmembrane protein 198	ENSMUSG00000051703	6174	2,75
Tstd3	thiosulfate sulfurtransferase (rhodanese)-like domain containing 3	ENSMUSG00000028251	9831	2,75
Ampd3	adenosine monophosphate deaminase 3	ENSMUSG00000005686	44200	2,75
		ENSMUSG00000039330	4353	2,75
		ENSMUSG00000073494	20860	2,75
Bcl6b	B cell CLL/lymphoma 6, member B	ENSMUSG00000000317	5671	2,75
Btc	betacellulin, epidermal growth factor family member	ENSMUSG00000082361	45734	2,75
Hif3a	hypoxia inducible factor 3, alpha subunit	ENSMUSG00000004328	30921	2,75
Slurp1	secreted Ly6/Plaur domain containing 1	ENSMUSG00000022596	1383	2,75
		ENSMUSG00000039037	165166	2,75
Lilrb4	leukocyte immunoglobulin-like receptor, subfamily B, member 4	ENSMUSG00000062593	15974	2,75
Angptl1	angiopoietin-like 1	ENSMUSG00000033544	22517	2,75
Dock2	dedicator of cyto-kinesis 2	ENSMUSG00000020143	557078	2,75
4930556J24Rik	RIKEN cDNA 4930556J24 gene	ENSMUSG00000034493	39238	2,75
Gm10115	predicted gene 10115	ENSMUSG00000062319	1228	2,75
Klhdc7a	kelch domain containing 7A	ENSMUSG00000078234	5854	2,75
5430401F13Rik	RIKEN cDNA 5430401F13 gene	ENSMUSG00000094113	67358	2,75
		ENSMUSG00000094800	14017	2,75
Abcc12	ATP-binding cassette, sub-family C (CFTR/MRP), member 12	ENSMUSG00000036872	98427	2,75

Lrrd1	leucine rich repeats and death domain containing 1	ENSMUSG00000040367	21424	2,75
Clic5	chloride intracellular channel 5	ENSMUSG00000023959	91597	2,75
Klrg2	killer cell lectin-like receptor subfamily G, member 2	ENSMUSG00000071537	10580	2,75
		ENSMUSG00000038257	184970	2,75
3222401L13Rik	RIKEN cDNA 3222401L13 gene	ENSMUSG00000073590	2831	2,75
Cd300a	CD300A antigen	ENSMUSG00000034652	14614	2,75
A1cf	APOBEC1 complementation factor	ENSMUSG00000052595	80646	2,75
		ENSMUSG00000055114	22275	2,75
Opn1sw	opsin 1 (cone pigments), short-wave-sensitive (color blindness, tritan)	ENSMUSG00000058831	11798	2,75
Krt80	keratin 80	ENSMUSG00000037185	20555	2,75
Chi3l3	chitinase 3-like 3	ENSMUSG00000040809	20011	2,75
Ccl4	chemokine (C-C motif) ligand 4	ENSMUSG00000018930	2100	2,75
Hes5	hairy and enhancer of split 5 (Drosophila)	ENSMUSG00000048001	1449	2,68
Igf1	insulin-like growth factor 1	ENSMUSG00000020053	78778	2,68
Pla2g7	phospholipase A2, group VII (platelet-activating factor acetylhydrolase, plasma)	ENSMUSG00000023913	44104	2,68
Col9a1	collagen, type IX, alpha 1	ENSMUSG00000026147	75075	2,68
Flrt2	fibronectin leucine rich transmembrane protein 2	ENSMUSG00000047414	92990	2,68
Tram1l1	translocation associated membrane protein 1-like 1	ENSMUSG00000044528	2224	2,68
Ccdc166	coiled-coil domain containing 166	ENSMUSG00000098176	2584	2,68
Ugt1a6b	UDP glucuronosyltransferase 1 family, polypeptide A6B	ENSMUSG00000090145	115752	2,68
		ENSMUSG00000089943	52986	2,68
Etv1	ets variant gene 1	ENSMUSG00000004151	88836	2,68
Sh2d5	SH2 domain containing 5	ENSMUSG00000045349	10930	2,68
Rgl3	ral guanine nucleotide dissociation stimulator-like 3	ENSMUSG00000040146	17927	2,68
		ENSMUSG00000059146	386047	2,68
Fcrla	Fc receptor-like A	ENSMUSG00000038421	10008	2,68
Dnajc4	DnaJ (Hsp40) homolog, subfamily C, member 4	ENSMUSG00000024963	4593	2,68
Klhl36	kelch-like 36	ENSMUSG00000031828	14684	2,68
Lgi4	leucine-rich repeat LGI family, member 4	ENSMUSG00000036560	11594	2,68
Casr	calcium-sensing receptor	ENSMUSG00000051980	68446	2,68
Fchsd1	FCH and double SH3 domains 1	ENSMUSG00000038524	12344	2,68
Gm9936	predicted gene 9936	ENSMUSG00000054123	2925	2,68
Zbtb26	zinc finger and BTB domain containing 26	ENSMUSG00000050714	10954	2,68
Zbtb39	zinc finger and BTB domain containing 39	ENSMUSG00000044617	7798	2,68
Tmem54	transmembrane protein 54	ENSMUSG00000028786	6079	2,68
Col4a6	collagen, type IV, alpha 6	ENSMUSG00000031273	308674	2,68
Pold2	polymerase (DNA directed), delta 2, regulatory subunit	ENSMUSG00000020471	6113	2,68
Tm4sf5	transmembrane 4 superfamily member 5	ENSMUSG00000018919	5935	2,68
Zfp354a	zinc finger protein 354A	ENSMUSG00000020364	13543	2,68

Foxi2	forkhead box I2	ENSMUSG00000048377	3249	2,68
Slc41a3	solute carrier family 41, member 3	ENSMUSG00000030089	41688	2,68
		ENSMUSG00000035226	58933	2,68
Wisp3	WNT1 inducible signaling pathway protein 3	ENSMUSG00000062074	12824	2,68
4930503L19Rik	RIKEN cDNA 4930503L19 gene	ENSMUSG00000044906	19341	2,68
Tmprss5	transmembrane protease, serine 5 (spinesin)	ENSMUSG00000032268	36328	2,68
Rin1	Ras and Rab interactor 1	ENSMUSG00000024883	6264	2,68
Ppapdc1b	phosphatidic acid phosphatase type 2 domain containing 1B	ENSMUSG00000031570	4827	2,68
		ENSMUSG00000014198	64913	2,68
		ENSMUSG00000021376	19568	2,68
P2ry2	purinergic receptor P2Y, G-protein coupled 2	ENSMUSG00000032860	15420	2,68
		ENSMUSG00000049871	31626	2,68
Ms4a4c	membrane-spanning 4-domains, subfamily A, member 4C	ENSMUSG00000024675	22477	2,68
Ttpa	tocopherol (alpha) transfer protein	ENSMUSG00000073988	22848	2,68
		ENSMUSG00000021708	251257	2,68
		ENSMUSG00000055228	12387	2,68
Tgfb1	transforming growth factor, beta 1	ENSMUSG00000002603	18076	2,68
Syk	spleen tyrosine kinase	ENSMUSG00000021457	65620	2,68
Dmrt1	doublesex and mab-3 related transcription factor 1	ENSMUSG00000024837	98712	2,68
Acvr1c	activin A receptor, type IC	ENSMUSG00000026834	90443	2,68
Adamts8	a disintegrin-like and metallopeptidase (reprolysin type) with thrombospondin type 1 motif, 8	ENSMUSG00000031994	21277	2,68
4930550C14Rik	RIKEN cDNA 4930550C14 gene	ENSMUSG00000005131	27518	2,68
Slc1a6	solute carrier family 1 (high affinity aspartate/glutamate transporter), member 6	ENSMUSG00000005357	34330	2,68
Pcsk9	proprotein convertase subtilisin/kexin type 9	ENSMUSG00000044254	22001	2,68
Il18r1	interleukin 18 receptor 1	ENSMUSG00000026070	35303	2,68
Nxph2	neurexophilin 2	ENSMUSG00000069132	80728	2,68
Smok3b	sperm motility kinase 3B	ENSMUSG00000079156	29208	2,68
Slc22a4	solute carrier family 22 (organic cation transporter), member 4	ENSMUSG00000020334	44968	2,68
Trim72	tripartite motif-containing 72	ENSMUSG00000042828	7405	2,68
Nrn1l	neuritin 1-like	ENSMUSG00000044287	1457	2,61
		ENSMUSG00000047603	8093	2,61
Suox	sulfite oxidase	ENSMUSG00000049858	4032	2,61
		ENSMUSG00000069899	968	2,61
		ENSMUSG00000062961	6496	2,61
Pear1	platelet endothelial aggregation receptor 1	ENSMUSG00000028073	76417	2,61
Cnn1	calponin 1	ENSMUSG00000001349	10350	2,61
Rnf217	ring finger protein 217	ENSMUSG00000063760	107298	2,61
Ccdc102a	coiled-coil domain containing 102A	ENSMUSG00000063605	15230	2,61
Zfp658	zinc finger protein 658	ENSMUSG00000056592	13206	2,61
Ccdc126	coiled-coil domain containing 126	ENSMUSG00000050786	22309	2,61
Zfp58	zinc finger protein 58	ENSMUSG00000071291	10389	2,61

Dus1l	dihydrouridine synthase 1-like (S. cerevisiae)	ENSMUSG00000025155	7203	2,61
		ENSMUSG00000074140	2250	2,61
Zfp583	zinc finger protein 583	ENSMUSG00000030443	15626	2,61
Ak4	adenylate kinase 4	ENSMUSG00000028527	47719	2,61
Acy3	aspartoacylase (aminoacylase) 3	ENSMUSG00000024866	3347	2,61
5830415F09Rik	RIKEN cDNA 5830415F09 gene	ENSMUSG00000028331	12933	2,61
Dscc1	defective in sister chromatid cohesion 1 homolog (S. cerevisiae)	ENSMUSG00000022422	14393	2,61
Duxbl1	double homeobox B-like 1	ENSMUSG00000048502	11512	2,61
Fbxo27	F-box protein 27	ENSMUSG00000037463	6490	2,61
Pitpnm1	phosphatidylinositol transfer protein, membrane-associated 1	ENSMUSG00000024851	13968	2,61
Arl5c	ADP-ribosylation factor-like 5C	ENSMUSG00000038352	6604	2,61
Gm2897	predicted gene 2897	ENSMUSG00000079410	27556	2,61
Cdh6	cadherin 6	ENSMUSG00000039385	139440	2,61
Cpb2	carboxypeptidase B2 (plasma)	ENSMUSG00000021999	41265	2,61
Esyt3	extended synaptotagmin-like protein 3	ENSMUSG00000037681	48564	2,61
Nrap	nebulin-related anchoring protein	ENSMUSG00000049134	70003	2,61
Cers3	ceramide synthase 3	ENSMUSG00000030510	80190	2,61
Rasd1	RAS, dexamethasone-induced 1	ENSMUSG00000049892	1764	2,61
Trim43c	tripartite motif-containing 43C	ENSMUSG00000067399	9027	2,61
4932438H23Rik	RIKEN cDNA 4932438H23 gene	ENSMUSG00000039851	41188	2,61
Grin2d	glutamate receptor, ionotropic, NMDA2D (epsilon 4)	ENSMUSG00000002771	39046	2,61
		ENSMUSG00000078898	11766	2,61
Neurl3	neuralized homolog 3 homolog (Drosophila)	ENSMUSG00000047180	8824	2,61
Izumo1	izumo sperm-egg fusion 1	ENSMUSG00000064158	7013	2,61
Pamr1	peptidase domain containing associated with muscle regeneration 1	ENSMUSG00000027188	93030	2,61
		ENSMUSG00000098133	309	2,61
Tgm6	transglutaminase 6	ENSMUSG00000027403	41817	2,61
Apol6	apolipoprotein L 6	ENSMUSG00000033576	12379	2,61
Ssh3	slingshot homolog 3 (Drosophila)	ENSMUSG00000034616	7505	2,61
1700110I01Rik	RIKEN cDNA 1700110I01 gene	ENSMUSG00000079407	43492	2,61
Nlrp9b	NLR family, pyrin domain containing 9B	ENSMUSG00000060508	65284	2,61
1700015G11Rik	RIKEN cDNA 1700015G11 gene	ENSMUSG00000094445	4037	2,61
Mgam	maltase-glucoamylase	ENSMUSG00000068587	140293	2,61
Myh7b	myosin, heavy chain 7B, cardiac muscle, beta	ENSMUSG00000074652	23096	2,61
Aoc1	amine oxidase, copper-containing 1	ENSMUSG00000029811	13935	2,61
Gpbar1	G protein-coupled bile acid receptor 1	ENSMUSG00000064272	1075	2,61
9230112D13Rik	RIKEN cDNA 9230112D13 gene	ENSMUSG00000021797	11183	2,61
Lmod3	leiomodin 3 (fetal)	ENSMUSG00000044086	14253	2,61
Ccrl2	chemokine (C-C motif) receptor-like 2	ENSMUSG00000043953	2436	2,61
		ENSMUSG00000046618	31776	2,61
Olfr613	olfactory receptor 613	ENSMUSG00000078624	5137	2,61
Ggt1	gamma-glutamyltransferase 1	ENSMUSG00000006345	24597	2,54
Cxcl5	chemokine (C-X-C motif) ligand 5	ENSMUSG00000029371	2327	2,54

Lpl	lipoprotein lipase	ENSMUSG00000015568	26958	2,54
Ogn	osteoglycin	ENSMUSG00000021390	16430	2,54
Svep1	sushi, von Willebrand factor type A, EGF and pentraxin domain containing 1	ENSMUSG00000028369	164418	2,54
		ENSMUSG00000074364	20591	2,54
Rpa3	replication protein A3	ENSMUSG00000012483	3238	2,54
Pparg	peroxisome proliferator activated receptor gamma	ENSMUSG00000000440	129181	2,54
B3galt4	UDP-Gal:betaGlcNAc beta 1,3-galactosyltransferase, polypeptide 4	ENSMUSG00000067370	1575	2,54
Fbln7	fibulin 7	ENSMUSG00000027386	34054	2,54
Ugt1a6a	UDP glucuronosyltransferase 1 family, polypeptide A6A	ENSMUSG00000054545	116751	2,54
Syt13	synaptotagmin XIII	ENSMUSG00000027220	40961	2,54
S1pr2	sphingosine-1-phosphate receptor 2	ENSMUSG00000043895	10842	2,54
		ENSMUSG00000033287	10346	2,54
		ENSMUSG00000066107	1006	2,54
Lenep	lens epithelial protein	ENSMUSG00000078173	1806	2,54
		ENSMUSG00000091568	21451	2,54
		ENSMUSG00000043346	1330	2,54
Zfp575	zinc finger protein 575	ENSMUSG00000066721	3804	2,54
D930048N14Rik	RIKEN cDNA D930048N14 gene	ENSMUSG00000052563	6728	2,54
Duxbl2	doubl homeobox B-like 2	ENSMUSG00000072675	11439	2,54
Apba3	amyloid beta (A4) precursor protein-binding, family A, member 3	ENSMUSG00000004931	5075	2,54
Dok5	docking protein 5	ENSMUSG00000027560	147963	2,54
Cyp4f17	cytochrome P450, family 4, subfamily f, polypeptide 17	ENSMUSG00000091586	22433	2,54
Asgr1	asialoglycoprotein receptor 1	ENSMUSG00000020884	3810	2,54
		ENSMUSG00000038540	40688	2,54
Slc26a11	solute carrier family 26, member 11	ENSMUSG00000039908	25523	2,54
Fam110c	family with sequence similarity 110, member C	ENSMUSG00000036136	5970	2,54
Spatc1	spermatogenesis and centriole associated 1	ENSMUSG00000049653	24484	2,54
Clec4n	C-type lectin domain family 4, member n	ENSMUSG00000023349	17179	2,54
Pld4	phospholipase D family, member 4	ENSMUSG00000052160	8332	2,54
Dhx58	DEXH (Asp-Glu-X-His) box polypeptide 58	ENSMUSG00000017830	9388	2,54
Orm3	orosomucoid 3	ENSMUSG00000028359	3350	2,54
Shc3	src homology 2 domain-containing transforming protein C3	ENSMUSG00000021448	136044	2,54
Apobr	apolipoprotein B receptor	ENSMUSG00000042759	4171	2,54
Vmn2r27	vomeronasal 2, receptor27	ENSMUSG00000072778	40189	2,54
Adra1b	adrenergic receptor, alpha 1b	ENSMUSG00000050541	126605	2,54
Inhbb	inhibin beta-B	ENSMUSG00000037035	6784	2,54
		ENSMUSG00000063305	12079	2,54
C2cd4b	C2 calcium-dependent domain containing 4B	ENSMUSG00000091956	1493	2,54
Strip2	striatin interacting protein 2	ENSMUSG00000039629	42669	2,54
Daw1	dynein assembly factor with WDR repeat domains 1	ENSMUSG00000053161	50823	2,54

Alpk3	alpha-kinase 3	ENSMUSG00000038763	48013	2,54
Ifi204	interferon activated gene 204	ENSMUSG00000073489	19584	2,54
Foxc1	forkhead box C1	ENSMUSG00000050295	3990	2,46
Adra2a	adrenergic receptor, alpha 2a	ENSMUSG00000033717	3801	2,46
S100b	S100 protein, beta polypeptide, neural	ENSMUSG00000033208	7307	2,46
Pgf	placental growth factor	ENSMUSG00000004791	10658	2,46
Tusc1	tumor suppressor candidate 1	ENSMUSG00000054000	1364	2,46
Ppapdc3	phosphatidic acid phosphatase type 2 domain containing 3	ENSMUSG00000051373	15293	2,46
Pcgf1	polycomb group ring finger 1	ENSMUSG00000069678	2987	2,46
Tbr1	T-box brain gene 1	ENSMUSG00000035033	11185	2,46
		ENSMUSG00000031077	5146	2,46
Emcn	endomucin	ENSMUSG00000054690	91119	2,46
Gm3558	predicted gene 3558	ENSMUSG00000079364	23416	2,46
Ttc39d	tetratricopeptide repeat domain 39D	ENSMUSG00000046196	10477	2,46
		ENSMUSG00000074881	1609	2,46
Cdk2ap2	CDK2-associated protein 2	ENSMUSG00000024856	1838	2,46
Ddc	dopa decarboxylase	ENSMUSG00000020182	84044	2,46
Prr7	proline rich 7 (synaptic)	ENSMUSG00000034686	8889	2,46
Car11	carbonic anhydrase 11	ENSMUSG00000003273	4840	2,46
Mycn	v-myc myelocytomatosis viral related oncogene, neuroblastoma derived (avian)	ENSMUSG00000037169	5819	2,46
Pigw	phosphatidylinositol glycan anchor biosynthesis, class W	ENSMUSG00000045140	3971	2,46
Lrrc14b	leucine rich repeat containing 14B	ENSMUSG00000021579	4428	2,46
Lamp3	lysosomal-associated membrane protein 3	ENSMUSG00000041247	52985	2,46
Cabp1	calcium binding protein 1	ENSMUSG00000029544	25693	2,46
Srcrb4d	scavenger receptor cysteine rich domain containing, group B (4 domains)	ENSMUSG00000029699	14321	2,46
Rps21	ribosomal protein S21	ENSMUSG00000039001	1069	2,46
Gm9961	predicted gene 9961	ENSMUSG00000055015	29238	2,46
Sectm1a	secreted and transmembrane 1A	ENSMUSG00000025165	13814	2,46
D630039A03Rik	RIKEN cDNA D630039A03 gene	ENSMUSG00000052117	7815	2,46
		ENSMUSG00000048424	98126	2,46
Gbp9	guanylate-binding protein 9	ENSMUSG00000029298	61146	2,46
Gm14305	predicted gene 14305	ENSMUSG00000078878	13486	2,46
Prdm6	PR domain containing 6	ENSMUSG00000069378	111362	2,46
Adam11	a disintegrin and metallopeptidase domain 11	ENSMUSG00000020926	18824	2,46
		ENSMUSG00000022847	9354	2,46
Kcnh5	potassium voltage-gated channel, subfamily H (eag-related), member 5	ENSMUSG00000034402	280116	2,46
Gm5475	predicted gene 5475	ENSMUSG00000087444	6004	2,46
Gm15097	predicted gene 15097	ENSMUSG00000094378	42222	2,46
1700024B05Rik	RIKEN cDNA 1700024B05 gene	ENSMUSG00000052334	59827	2,46
Elfn1	leucine rich repeat and fibronectin type III, extracellular 1	ENSMUSG00000048988	66769	2,46
Slc22a30	solute carrier family 22, member 30	ENSMUSG00000052562	69741	2,46
		ENSMUSG00000073197	19454	2,46

Naip2	NLR family, apoptosis inhibitory protein 2	ENSMUSG00000078945	58030	2,46
Mst1	macrophage stimulating 1 (hepatocyte growth factor-like)	ENSMUSG00000032591	4592	2,46
Abcc6	ATP-binding cassette, sub-family C (CFTR/MRP), member 6	ENSMUSG00000030834	53923	2,46
		ENSMUSG00000003198	6401	2,46
H1foo	H1 histone family, member O, oocyte-specific	ENSMUSG00000042279	5299	2,46
4930486L24Rik	RIKEN cDNA 4930486L24 gene	ENSMUSG00000050345	21796	2,46
		ENSMUSG00000053211	72203	2,46
Rgsl1	regulator of G-protein signaling like 1	ENSMUSG00000042641	64762	2,46
		ENSMUSG00000079537	5798	2,46
Gsdmc	gasdermin C	ENSMUSG00000079025	32789	2,46
Aanat	arylalkylamine N-acetyltransferase	ENSMUSG00000020804	11249	2,46
		ENSMUSG00000095891	7873	2,46
Slc22a2	solute carrier family 22 (organic cation transporter), member 2	ENSMUSG00000040966	44277	2,39
Slc7a2	solute carrier family 7 (cationic amino acid transporter, y+ system), member 2	ENSMUSG00000031596	59913	2,39
Fam131b	family with sequence similarity 131, member B	ENSMUSG00000029861	9332	2,39
Slc52a3	solute carrier protein family 52, member 3	ENSMUSG00000027463	12748	2,39
Crtc2	CREB regulated transcription coactivator 2	ENSMUSG00000027936	9963	2,39
		ENSMUSG00000087610	3680	2,39
Comtd1	catechol-O-methyltransferase domain containing 1	ENSMUSG00000021773	3117	2,39
Ms4a6c	membrane-spanning 4-domains, subfamily A, member 6C	ENSMUSG00000079419	12830	2,39
Efcc1	EF hand and coiled-coil domain containing 1	ENSMUSG00000068263	25040	2,39
Gm9776	predicted gene 9776	ENSMUSG00000042857	2980	2,39
Stfa1	stefin A1	ENSMUSG00000071562	8224	2,39
Cda	cytidine deaminase	ENSMUSG00000028755	29569	2,39
1700066B19Rik	RIKEN cDNA 1700066B19 gene	ENSMUSG00000073598	27645	2,39
		ENSMUSG00000097485	1838	2,39
Pus7l	pseudouridylate synthase 7 homolog (S. cerevisiae)-like	ENSMUSG00000033356	20860	2,39
Clhc1	clathrin heavy chain linker domain containing 1	ENSMUSG00000078521	12929	2,39
		ENSMUSG00000009075	9887	2,39
Sh2d6	SH2 domain containing 6	ENSMUSG00000052631	7991	2,39
Abca8b	ATP-binding cassette, sub-family A (ABC1), member 8b	ENSMUSG00000020620	63656	2,39
Zfp319	zinc finger protein 319	ENSMUSG00000046556	5814	2,39
		ENSMUSG00000092511	25631	2,39
		ENSMUSG00000020253	5575	2,39
B4galnt4	beta-1,4-N-acetyl-galactosaminyl transferase 4	ENSMUSG00000055629	10846	2,39
Rbfox3	RNA binding protein, fox-1 homolog (C. elegans) 3	ENSMUSG00000025576	421836	2,39
Xlr3a	X-linked lymphocyte-regulated 3A	ENSMUSG00000057836	10803	2,39

A330017A19Rik	RIKEN cDNA A330017A19 gene	ENSMUSG00000073388	1190	2,39
Tnfrsf10b	tumor necrosis factor receptor superfamily, member 10b	ENSMUSG00000022074	16932	2,39
Reep2	receptor accessory protein 2	ENSMUSG00000038555	6875	2,39
Acmsd	amino carboxymuconate semialdehyde decarboxylase	ENSMUSG00000026348	38152	2,39
Gm10338	predicted gene 10338	ENSMUSG00000094706	14976	2,39
Pcsk1	proprotein convertase subtilisin/kexin type 1	ENSMUSG00000021587	42673	2,39
Eva1a	eva-1 homolog A (C. elegans)	ENSMUSG00000035104	52057	2,39
Vmn2r21	vomeronasal 2, receptor 21	ENSMUSG00000093820	40899	2,39
Ppm1j	protein phosphatase 1J	ENSMUSG00000002228	4963	2,39
Plcxd2	phosphatidylinositol-specific phospholipase C, X domain containing 2	ENSMUSG00000087141	50956	2,39
1700125H20Rik	RIKEN cDNA 1700125H20 gene	ENSMUSG00000018479	10742	2,39
Gucy2f	guanylate cyclase 2f	ENSMUSG00000042282	115772	2,39
Ppp1r36	protein phosphatase 1, regulatory subunit 36	ENSMUSG00000052221	21875	2,39
		ENSMUSG00000078889	11737	2,39
Zfp641	zinc finger protein 641	ENSMUSG00000022987	10543	2,39
Trip10	thyroid hormone receptor interactor 10	ENSMUSG00000019487	14247	2,39
Gm8898	predicted gene 8898	ENSMUSG00000089756	12220	2,39
Slc22a28	solute carrier family 22, member 28	ENSMUSG00000063590	69774	2,39
Slc11a1	solute carrier family 11 (proton-coupled divalent metal ion transporters), member 1	ENSMUSG00000026177	10860	2,39
Pde6b	phosphodiesterase 6B, cGMP, rod receptor, beta polypeptide	ENSMUSG00000029491	44007	2,39
Tpte	transmembrane phosphatase with tensin homology	ENSMUSG00000031481	87978	2,39
Ldlrad1	low density lipoprotein receptor class A domain containing 1	ENSMUSG00000070877	8881	2,39
Gm6994	predicted gene 6994	ENSMUSG00000067973	27386	2,39
1700019B03Rik	RIKEN cDNA 1700019B03 gene	ENSMUSG00000040340	16320	2,39
Cldn15	claudin 15	ENSMUSG00000001739	9243	2,39
Rhox2e	reproductive homeobox 2E	ENSMUSG00000096788	11358	2,39
Igsf6	immunoglobulin superfamily, member 6	ENSMUSG00000035004	10464	2,39
		ENSMUSG00000062511	38613	2,39
Afm	afamin	ENSMUSG00000029369	34612	2,39
		ENSMUSG00000020420	17011	2,39
Ghrh	growth hormone releasing hormone	ENSMUSG00000027643	18010	2,39
Mup6	major urinary protein 6	ENSMUSG00000078689	42981	2,39
Rnf17	ring finger protein 17	ENSMUSG00000000365	122336	2,39
Gm21704	predicted gene, 21704	ENSMUSG00000093987	43018	2,39
Aqp7	aquaporin 7	ENSMUSG00000028427	15066	2,39
Zfp619	zinc finger protein 619	ENSMUSG00000068959	22655	2,39
2010107G12Rik	RIKEN cDNA 2010107G12 gene	ENSMUSG00000029847	33777	2,39
Ttc34	tetratricopeptide repeat domain 34	ENSMUSG00000046637	10926	2,39
Rasgef1c	RasGEF domain family, member 1C	ENSMUSG00000020374	79160	2,39
Loxl1	lysyl oxidase-like 1	ENSMUSG00000032334	25490	2,32
S100a10	S100 calcium binding protein A10 (calpactin)	ENSMUSG00000041959	9564	2,32

Tspan15	tetraspanin 15	ENSMUSG00000037031	45823	2,32
Myh11	myosin, heavy polypeptide 11, smooth muscle	ENSMUSG00000018830	96882	2,32
Nrgn	neurogranin	ENSMUSG00000053310	8413	2,32
LOC101055924	uncharacterized LOC101055924	ENSMUSG00000039951	2367	2,32
Plekha2	pleckstrin homology domain-containing, family A (phosphoinositide binding specific) member 2	ENSMUSG00000031557	63051	2,32
Hnf1a	HNF1 homeobox A	ENSMUSG00000029556	22707	2,32
Chac1	ChaC, cation transport regulator 1	ENSMUSG00000027313	3153	2,32
Pdia2	protein disulfide isomerase associated 2	ENSMUSG00000024184	3089	2,32
Zfp105	zinc finger protein 105	ENSMUSG00000057895	7957	2,32
Amt	aminomethyltransferase	ENSMUSG00000032607	4719	2,32
Csf2ra	colony stimulating factor 2 receptor, alpha, low-affinity (granulocyte-macrophage)	ENSMUSG00000059326	4017	2,32
		ENSMUSG00000035364	603	2,32
4921530D09Rik	RIKEN cDNA 4921530D09 gene	ENSMUSG00000026940	9044	2,32
1700030F18Rik	RIKEN cDNA 1700030F18 gene	ENSMUSG00000045350	48728	2,32
Fhl3	four and a half LIM domains 3	ENSMUSG00000032643	7911	2,32
Lpcat4	lysophosphatidylcholine acyltransferase 4	ENSMUSG00000027134	7644	2,32
Ccdc159	coiled-coil domain containing 159	ENSMUSG00000006241	8402	2,32
Trpm8	transient receptor potential cation channel, subfamily M, member 8	ENSMUSG00000036251	111633	2,32
		ENSMUSG00000085601	16818	2,32
Adam2	a disintegrin and metallopeptidase domain 2	ENSMUSG00000022039	50405	2,32
Gm3494	predicted gene 3494	ENSMUSG00000095295	21254	2,32
Tex22	testis expressed gene 22	ENSMUSG00000012211	14416	2,32
Man2b2	mannosidase 2, alpha B2	ENSMUSG00000029119	23733	2,32
Vmn2r22	vomeronasal 2, receptor 22	ENSMUSG00000095486	40878	2,32
Cd28	CD28 antigen	ENSMUSG00000026012	56560	2,32
BC035044	cDNA sequence BC035044	ENSMUSG00000090164	43083	2,32
		ENSMUSG00000013418	48949	2,32
1700001O22Rik	RIKEN cDNA 1700001O22 gene	ENSMUSG00000044320	8893	2,32
Gdpd2	glycerophosphodiester phosphodiesterase domain containing 2	ENSMUSG00000019359	9023	2,32
Upk1a	uroplakin 1A	ENSMUSG00000006313	9643	2,32
Ptpro	protein tyrosine phosphatase, receptor type, O	ENSMUSG00000030223	212331	2,32
Sqrdl	sulfide quinone reductase-like (yeast)	ENSMUSG00000005803	44333	2,32
Kcnk6	potassium inwardly-rectifying channel, subfamily K, member 6	ENSMUSG00000046410	10595	2,32
Fancb	Fanconi anemia, complementation group B	ENSMUSG00000047757	16681	2,32
Gm6034	predicted gene 6034	ENSMUSG00000073407	15685	2,32
B4galnt1	beta-1,4-N-acetyl-galactosaminyl transferase 1	ENSMUSG00000006731	7185	2,32
Igflr1	IGF-like family receptor 1	ENSMUSG00000036826	2536	2,32
Gm14440	predicted gene 14440	ENSMUSG00000078901	11681	2,32
Styk1	serine/threonine/tyrosine kinase 1	ENSMUSG00000032899	54454	2,32

Abca12	ATP-binding cassette, sub-family A (ABC1), member 12	ENSMUSG00000050296	171821	2,32
Hsd17b14	hydroxysteroid (17-beta) dehydrogenase 14	ENSMUSG00000030825	12860	2,32
AA986860	expressed sequence AA986860	ENSMUSG00000042510	12646	2,32
Gpr132	G protein-coupled receptor 132	ENSMUSG00000021298	10041	2,32
		ENSMUSG00000060791	10787	2,32
AI607873	expressed sequence AI607873	ENSMUSG00000073490	18380	2,32
2310007L24Rik	RIKEN cDNA 2310007L24 gene	ENSMUSG00000009210	11641	2,32
Slc39a12	solute carrier family 39 (zinc transporter), member 12	ENSMUSG00000036949	106660	2,32
Prkcg	protein kinase C, gamma	ENSMUSG00000078816	27563	2,32
Wnt2	wingless-related MMTV integration site 2	ENSMUSG00000010797	41646	2,32
Gabrr2	gamma-aminobutyric acid (GABA) C receptor, subunit rho 2	ENSMUSG00000023267	32867	2,32
Serpina10	serine (or cysteine) peptidase inhibitor, clade A (alpha-1 antiproteinase, antitrypsin), member 10	ENSMUSG00000061947	14770	2,32
Gm11435	predicted gene 11435	ENSMUSG00000018925	10440	2,32
Ppp1r1c	protein phosphatase 1, regulatory (inhibitor) subunit 1C	ENSMUSG00000034683	110717	2,32
Col6a1	collagen, type VI, alpha 1	ENSMUSG00000001119	17377	2,25
Fbln2	fibulin 2	ENSMUSG00000064080	60086	2,25
Spta1	spectrin alpha, erythrocytic 1	ENSMUSG00000026532	75675	2,25
Prss23	protease, serine, 23	ENSMUSG00000039405	9802	2,25
Odf3b	outer dense fiber of sperm tails 3B	ENSMUSG00000047394	1805	2,25
Lrdd	leucine-rich and death domain containing	ENSMUSG00000025507	5913	2,25
Col24a1	collagen, type XXIV, alpha 1	ENSMUSG00000028197	259540	2,25
Tcf19	transcription factor 19	ENSMUSG00000050410	4091	2,25
Fam221a	family with sequence similarity 221, member A	ENSMUSG00000047115	22167	2,25
Loxl3	lysyl oxidase-like 3	ENSMUSG00000000693	18390	2,25
		ENSMUSG00000090957	909	2,25
		ENSMUSG00000063902	1453	2,25
B3gnt9	UDP-GlcNAc:betaGal beta-1,3-N-acetylglucosaminyltransferase 9	ENSMUSG00000069920	2516	2,25
Krt222	keratin 222	ENSMUSG00000035849	11325	2,25
Gm10503	predicted gene 10503	ENSMUSG00000073427	2645	2,25
Pcdhb17	protocadherin beta 17	ENSMUSG00000046387	3269	2,25
Col17a1	collagen, type XVII, alpha 1	ENSMUSG00000025064	45751	2,25
Wnt9a	wingless-type MMTV integration site 9A	ENSMUSG00000000126	26625	2,25
Irf7	interferon regulatory factor 7	ENSMUSG00000025498	3776	2,25
Cdca7	cell division cycle associated 7	ENSMUSG00000055612	10735	2,25
Gm9847	predicted pseudogene 9847	ENSMUSG00000050974	597	2,25
Xlr3b	X-linked lymphocyte-regulated 3B	ENSMUSG00000073125	10724	2,25
Ikzf1	IKAROS family zinc finger 1	ENSMUSG00000018654	87924	2,25
Ms4a6d	membrane-spanning 4-domains, subfamily A, member 6D	ENSMUSG00000024679	18246	2,25
Gsg1	germ cell-specific gene 1	ENSMUSG00000030206	17008	2,25
		ENSMUSG00000095199	11424	2,25
Ptch2	patched homolog 2	ENSMUSG00000028681	20027	2,25
		ENSMUSG00000073413	6623	2,25

Zfp185	zinc finger protein 185	ENSMUSG00000031351	44205	2,25
S100g	S100 calcium binding protein G	ENSMUSG00000040808	2608	2,25
Sntg2	syntrophin, gamma 2	ENSMUSG00000020672	198894	2,25
C5ar1	complement component 5a receptor 1	ENSMUSG00000049130	12798	2,25
Lag3	lymphocyte-activation gene 3	ENSMUSG00000030124	7345	2,25
Gm10621	predicted gene 10621	ENSMUSG00000074075	2114	2,25
Sprtn	SprT-like N-terminal domain	ENSMUSG00000031986	5928	2,25
Lrrk2	leucine-rich repeat kinase 2	ENSMUSG00000036273	142946	2,25
		ENSMUSG00000075520	108681	2,25
0610012H03Rik	RIKEN cDNA 0610012H03 gene	ENSMUSG00000055312	155477	2,25
Mr1	major histocompatibility complex, class I-related	ENSMUSG00000026471	18903	2,25
		ENSMUSG00000044021	98063	2,25
		ENSMUSG00000079800	49759	2,25
1700011L22Rik	RIKEN cDNA 1700011L22 gene	ENSMUSG00000031682	38154	2,25
Il1rl2	interleukin 1 receptor-like 2	ENSMUSG00000070942	40845	2,25
Vmn2r13	vomeronasal 2, receptor 13	ENSMUSG00000091635	36040	2,25
Bpifb5	BPI fold containing family B, member 5	ENSMUSG00000038572	17161	2,25
Clrn1	clarin 1	ENSMUSG00000043850	41313	2,25
Ankrd66	ankyrin repeat domain 66	ENSMUSG00000096140	9466	2,25
Olfr631	olfactory receptor 631	ENSMUSG00000042219	14850	2,25
Bpifb3	BPI fold containing family B, member 3	ENSMUSG00000068008	14767	2,17
Acy1	aminoacylase 1	ENSMUSG00000023262	5241	2,17
Prmt8	protein arginine N-methyltransferase 8	ENSMUSG00000030350	80462	2,17
Zcchc24	zinc finger, CCHC domain containing 24	ENSMUSG00000055538	57217	2,17
Rgs10	regulator of G-protein signalling 10	ENSMUSG00000030844	45138	2,17
		ENSMUSG00000069713	1789	2,17
Csf1	colony stimulating factor 1 (macrophage)	ENSMUSG00000014599	19422	2,17
Ddx25	DEAD (Asp-Glu-Ala-Asp) box polypeptide 25	ENSMUSG00000032101	16623	2,17
Izumo4	IZUMO family member 4	ENSMUSG00000055862	2690	2,17
Plac8	placenta-specific 8	ENSMUSG00000029322	18521	2,17
		ENSMUSG00000042268	27483	2,17
Fes	feline sarcoma oncogene	ENSMUSG00000053158	10189	2,17
Gpx3	glutathione peroxidase 3	ENSMUSG00000018339	7925	2,17
		ENSMUSG00000055235	18990	2,17
Rcn3	reticulocalbin 3, EF-hand calcium binding domain	ENSMUSG00000019539	9309	2,17
		ENSMUSG00000097808	1815	2,17
Cdh22	cadherin 22	ENSMUSG00000053166	123347	2,17
Masp1	mannan-binding lectin serine peptidase 1	ENSMUSG00000022887	68806	2,17
Hspb2	heat shock protein 2	ENSMUSG00000038086	1283	2,17
		ENSMUSG00000041849	12559	2,17
		ENSMUSG00000032609	4720	2,17
Nrp	neural regeneration protein	ENSMUSG00000021041	1335	2,17
Trim15	tripartite motif-containing 15	ENSMUSG00000050747	6518	2,17
Sep 04	septin 4	ENSMUSG00000020486	21637	2,17
Gm10638	predicted gene 10638	ENSMUSG00000074178	905	2,17
Pkn3	protein kinase N3	ENSMUSG00000026785	13339	2,17

Stk32c	serine/threonine kinase 32C	ENSMUSG00000015981	109670	2,17
Hist1h1a	histone cluster 1, H1a	ENSMUSG00000049539	747	2,17
Trim16	tripartite motif-containing 16	ENSMUSG00000047821	30578	2,17
Usp51	ubiquitin specific protease 51	ENSMUSG00000067215	2948	2,17
Bfsp1	beaded filament structural protein 1, in lens-CP94	ENSMUSG00000027420	36646	2,17
Slc10a3	solute carrier family 10 (sodium/bile acid cotransporter family), member 3	ENSMUSG00000032806	4133	2,17
		ENSMUSG00000090441	1664	2,17
Scel	sciellin	ENSMUSG00000022123	100006	2,17
Npl	N-acetylneuraminate pyruvate lyase	ENSMUSG00000042684	46699	2,17
Htra3	HtrA serine peptidase 3	ENSMUSG00000029096	27742	2,17
Cutal	cutA divalent cation tolerance homolog-like	ENSMUSG00000026870	17737	2,17
		ENSMUSG00000072919	12302	2,17
Pnldc1	poly(A)-specific ribonuclease (PARN)-like domain containing 1	ENSMUSG00000073460	21099	2,17
Txlnb	taxilin beta	ENSMUSG00000039891	49440	2,17
Clca4	chloride channel calcium activated 4	ENSMUSG00000037033	26735	2,17
Gpr97	G protein-coupled receptor 97	ENSMUSG00000060470	27556	2,17
Gpr110	G protein-coupled receptor 110	ENSMUSG00000041293	54409	2,17
Gatsl3	GATS protein-like 3	ENSMUSG00000020424	4185	2,17
Naip5	NLR family, apoptosis inhibitory protein 5	ENSMUSG00000071203	34585	2,17
Gm8787	predicted gene 8787	ENSMUSG00000079517	27556	2,17
Sh2d1b1	SH2 domain protein 1B1	ENSMUSG00000096334	8287	2,17
Ankle1	ankyrin repeat and LEM domain containing 1	ENSMUSG00000046295	3895	2,17
Plb1	phospholipase B1	ENSMUSG00000029134	133813	2,17
		ENSMUSG00000097192	31164	2,17
Gm2012	predicted gene 2012	ENSMUSG00000082639	30678	2,17
		ENSMUSG00000024806	10528	2,17
		ENSMUSG00000004842	14494	2,17
4932411E22Rik	RIKEN cDNA 4932411E22 gene	ENSMUSG00000047490	28726	2,17
Prrx1	paired related homeobox 1	ENSMUSG00000026586	68595	2,10
Gnmt	glycine N-methyltransferase	ENSMUSG00000002769	3505	2,10
Dse	dermatan sulfate epimerase	ENSMUSG00000039497	56158	2,10
Fam105a	family with sequence similarity 105, member A	ENSMUSG00000056069	26472	2,10
Fam212b	family with sequence similarity 212, member B	ENSMUSG00000048458	16244	2,10
Prss41	protease, serine, 41	ENSMUSG00000024114	7388	2,10
Gm10108	predicted pseudogene 10108	ENSMUSG00000062038	318	2,10
Abca4	ATP-binding cassette, sub-family A (ABC1), member 4	ENSMUSG00000028125	135619	2,10
		ENSMUSG00000036599	20175	2,10
Trpt1	tRNA phosphotransferase 1	ENSMUSG00000047656	2914	2,10
Tpbg	trophoblast glycoprotein	ENSMUSG00000035274	4676	2,10
Tmem204	transmembrane protein 204	ENSMUSG00000024168	23480	2,10
Mfsd10	major facilitator superfamily domain containing 10	ENSMUSG00000001082	3571	2,10
Myocd	myocardin	ENSMUSG00000020542	93429	2,10
Gpr85	G protein-coupled receptor 85	ENSMUSG00000048216	5485	2,10

		ENSMUSG00000032179	124947	2,10
Cyth4	cytohesin 4	ENSMUSG00000018008	24973	2,10
Pdzd9	PDZ domain containing 9	ENSMUSG00000030887	11048	2,10
4921536K21Rik	RIKEN cDNA 4921536K21 gene	ENSMUSG00000020434	9011	2,10
Gm514	predicted gene 514	ENSMUSG00000086228	19318	2,10
Cgref1	cell growth regulator with EF hand domain 1	ENSMUSG00000029161	12335	2,10
Lrfn2	leucine rich repeat and fibronectin type III domain containing 2	ENSMUSG00000040490	165210	2,10
Dysf	dysferlin	ENSMUSG00000033788	202471	2,10
Irgm1	immunity-related GTPase family M member 1	ENSMUSG00000046879	9716	2,10
Fgf21	fibroblast growth factor 21	ENSMUSG00000030827	1584	2,10
Itpr3	inositol 1,4,5-triphosphate receptor 3	ENSMUSG00000042644	64920	2,10
Bend4	BEN domain containing 4	ENSMUSG00000092060	35653	2,10
Afap1l1	actin filament associated protein 1-like 1	ENSMUSG00000033032	56442	2,10
Qpct	glutaminyl-peptide cyclotransferase (glutaminyl cyclase)	ENSMUSG00000024084	38338	2,10
Zfp595	zinc finger protein 595	ENSMUSG00000057842	19563	2,10
2310002J15Rik	RIKEN cDNA 2310002J15 gene	ENSMUSG00000036731	3626	2,10
Nod1	nucleotide-binding oligomerization domain containing 1	ENSMUSG00000038058	48671	2,10
Abca6	ATP-binding cassette, sub-family A (ABC1), member 6	ENSMUSG00000044749	74957	2,10
Hscb	HscB iron-sulfur cluster co-chaperone homolog (E. coli)	ENSMUSG00000043510	10708	2,10
		ENSMUSG00000039095	7917	2,10
		ENSMUSG00000053985	6682	2,10
Myo1f	myosin IF	ENSMUSG00000024300	52046	2,10
		ENSMUSG00000074300	43102	2,10
Lck	lymphocyte protein tyrosine kinase	ENSMUSG00000000409	25298	2,10
		ENSMUSG00000074521	8689	2,10
Sec16b	SEC16 homolog B (S. cerevisiae)	ENSMUSG00000026589	61698	2,10
Sgk2	serum/glucocorticoid regulated kinase 2	ENSMUSG00000017868	26798	2,10
Timp4	tissue inhibitor of metalloproteinase 4	ENSMUSG00000030317	6234	2,10
Zfp712	zinc finger protein 712	ENSMUSG00000090641	22577	2,10
Sycp2	synaptonemal complex protein 2	ENSMUSG00000060445	62393	2,10
Agmat	agmatine ureohydrolase (agmatinase)	ENSMUSG00000040706	12592	2,10
Ankrd2	ankyrin repeat domain 2 (stretch responsive muscle)	ENSMUSG00000025172	9073	2,10
Bdh2	3-hydroxybutyrate dehydrogenase, type 2	ENSMUSG00000028167	23206	2,10
		ENSMUSG00000022935	159981	2,10
Ccdc7	coiled-coil domain containing 7	ENSMUSG00000025808	85499	2,10
		ENSMUSG00000090093	12520	2,10
Trim43a	tripartite motif-containing 43A	ENSMUSG00000090693	7784	2,10
Gpr112	G protein-coupled receptor 112	ENSMUSG00000053852	131396	2,10
Psma8	proteasome (prosome, macropain) subunit, alpha type, 8	ENSMUSG00000036743	56149	2,10
Capn13	calpain 13	ENSMUSG00000043705	92833	2,10

Nlrc5	NLR family, CARD domain containing 5	ENSMUSG00000074151	54510	2,10
		ENSMUSG00000040113	22976	2,10
Cyp2j11	cytochrome P450, family 2, subfamily j, polypeptide 11	ENSMUSG00000066097	54155	2,10
4921528I07Rik	RIKEN cDNA 4921528I07 gene	ENSMUSG00000064299	40346	2,10
Cyp2c54	cytochrome P450, family 2, subfamily c, polypeptide 54	ENSMUSG00000067225	35871	2,10
		ENSMUSG00000062783	51440	2,10
Tinag	tubulointerstitial nephritis antigen	ENSMUSG00000032357	94084	2,10
		ENSMUSG00000087303	42276	2,10
Crisp2	cysteine-rich secretory protein 2	ENSMUSG00000023930	42270	2,10
		ENSMUSG00000080115	10332	2,10
Slc9c1	solute carrier family 9, subfamily C (Na+-transporting carboxylic acid decarboxylase), member 1	ENSMUSG00000033210	71736	2,10
Rp1	retinitis pigmentosa 1 (human)	ENSMUSG00000025900	16808	2,10
Fbxw21	F-box and WD-40 domain protein 21	ENSMUSG00000047237	22588	2,10
A430093F15Rik	RIKEN cDNA A430093F15 gene	ENSMUSG00000067577	45097	2,10
		ENSMUSG00000054362	26333	2,10
Gfral	GDNF family receptor alpha like	ENSMUSG00000059383	49556	2,10
Bgn	biglycan	ENSMUSG00000031375	12332	2,03
Col4a1	collagen, type IV, alpha 1	ENSMUSG00000031502	114404	2,03
Nid1	nidogen 1	ENSMUSG00000005397	74674	2,03
Pxdc1	PX domain containing 1	ENSMUSG00000021411	24842	2,03
Car12	carbonic anyhydrase 12	ENSMUSG00000032373	53160	2,03
H2-DMb1	histocompatibility 2, class II, locus Mb1	ENSMUSG00000079547	7152	2,03
Tceal5	transcription elongation factor A (SII)-like 5	ENSMUSG00000054034	2929	2,03
		ENSMUSG00000079317	12567	2,03
Ube2ql1	ubiquitin-conjugating enzyme E2Q family-like 1	ENSMUSG00000052981	37063	2,03
		ENSMUSG00000041731	178840	2,03
Darc	Duffy blood group, chemokine receptor	ENSMUSG00000037872	1618	2,03
Slc23a3	solute carrier family 23 (nucleobase transporters), member 3	ENSMUSG00000026205	8347	2,03
Tmem170	transmembrane protein 170	ENSMUSG00000031953	11778	2,03
		ENSMUSG00000097508	1733	2,03
Myrip	myosin VIIA and Rab interacting protein	ENSMUSG00000041794	170906	2,03
Gstt2	glutathione S-transferase, theta 2	ENSMUSG00000033318	3037	2,03
Fam150a	family with sequence similarity 150, member A	ENSMUSG00000087247	35401	2,03
Col28a1	collagen, type XXVIII, alpha 1	ENSMUSG00000068794	194810	2,03
Kcnt2	potassium channel, subfamily T, member 2	ENSMUSG00000052726	365848	2,03
B230317F23Rik	RIKEN cDNA B230317F23 gene	ENSMUSG00000045746	2472	2,03
Plekhn1	pleckstrin homology domain containing, family N member 1	ENSMUSG00000078485	7087	2,03
Amica1	adhesion molecule, interacts with CXADR antigen 1	ENSMUSG00000048534	29348	2,03

Ablim3	actin binding LIM protein family, member 3	ENSMUSG00000032735	112458	2,03
Mars2	methionine-tRNA synthetase 2 (mitochondrial)	ENSMUSG00000046994	2882	2,03
Strc	stereocilin	ENSMUSG00000033498	23441	2,03
Plek2	pleckstrin 2	ENSMUSG00000021118	18274	2,03
Myh15	myosin, heavy chain 15	ENSMUSG00000092009	141619	2,03
Crhbp	corticotropin releasing hormone binding protein	ENSMUSG00000021680	13461	2,03
Gm5595	predicted gene 5595	ENSMUSG00000069727	32614	2,03
AI481877	expressed sequence AI481877	ENSMUSG00000038598	95231	2,03
		ENSMUSG00000054659	15508	2,03
Ndst4	N-deacetylase/N-sulfotransferase (heparin glucosaminyl) 4	ENSMUSG00000027971	320893	2,03
Relt	RELT tumor necrosis factor receptor	ENSMUSG00000008318	17599	2,03
Gvin1	GTPase, very large interferon inducible 1	ENSMUSG00000045868	58806	2,03
Klra17	killer cell lectin-like receptor, subfamily A, member 17	ENSMUSG00000014543	45519	2,03
Pnp2	purine-nucleoside phosphorylase 2	ENSMUSG00000068417	8758	2,03
F10	coagulation factor X	ENSMUSG00000031444	19369	2,03
Cyp2ab1	cytochrome P450, family 2, subfamily ab, polypeptide 1	ENSMUSG00000022818	14679	2,03
Olfr309	olfactory receptor 309	ENSMUSG00000054054	7325	2,03
Bai2	brain-specific angiogenesis inhibitor 2	ENSMUSG00000028782	37764	1,96
Nes	nestin	ENSMUSG00000004891	9374	1,96
Rab33a	RAB33A, member of RAS oncogene family	ENSMUSG00000031104	10948	1,96
Hspa12b	heat shock protein 12B	ENSMUSG00000074793	19042	1,96
Bean1	brain expressed, associated with Nedd4, 1	ENSMUSG00000031872	48580	1,96
Haus5	HAUS augmin-like complex, subunit 5	ENSMUSG00000078762	11284	1,96
Tbx15	T-box 15	ENSMUSG00000027868	113879	1,96
Erg	avian erythroblastosis virus E-26 (v-ets) oncogene related	ENSMUSG00000040732	227425	1,96
Bsnd	Bartter syndrome, infantile, with sensorineural deafness (Barttin)	ENSMUSG00000025418	8828	1,96
Dbpht2	DNA binding protein with his-thr domain	ENSMUSG00000029878	2995	1,96
BC028528	cDNA sequence BC028528	ENSMUSG00000038543	8052	1,96
5430435G22Rik	RIKEN cDNA 5430435G22 gene	ENSMUSG00000052688	24848	1,96
Dusp15	dual specificity phosphatase-like 15	ENSMUSG00000042662	10704	1,96
Emid1	EMI domain containing 1	ENSMUSG00000034164	45993	1,96
Acp6	acid phosphatase 6, lysophosphatidic	ENSMUSG00000028093	17798	1,96
		ENSMUSG00000097273	1682	1,96
Gm5919	predicted gene 5919	ENSMUSG00000090522	3837	1,96
Gpr157	G protein-coupled receptor 157	ENSMUSG00000047875	18563	1,96
		ENSMUSG00000070522	1131	1,96
Car7	carbonic anhydrase 7	ENSMUSG00000031883	15644	1,96
1700066M21Rik	RIKEN cDNA 1700066M21 gene	ENSMUSG00000038323	7804	1,96
Gm5113	predicted gene 5113	ENSMUSG00000066647	10335	1,96

Prr16	proline rich 16	ENSMUSG00000073565	186744	1,96
Zfp189	zinc finger protein 189	ENSMUSG00000039634	10342	1,96
Foxp3	forkhead box P3	ENSMUSG00000039521	15568	1,96
Pthlh	parathyroid hormone-like peptide	ENSMUSG00000048776	12067	1,96
Rrp9	RRP9, small subunit (SSU) processome component, homolog (yeast)	ENSMUSG00000041506	9462	1,96
		ENSMUSG00000069806	35262	1,96
2310015B20Rik	RIKEN cDNA 2310015B20 gene	ENSMUSG00000019933	15027	1,96
Acer1	alkaline ceramidase 1	ENSMUSG00000045019	28637	1,96
Slc38a11	solute carrier family 38, member 11	ENSMUSG00000061171	47605	1,96
		ENSMUSG00000090498	4065	1,96
Tox2	TOX high mobility group box family member 2	ENSMUSG00000074607	121046	1,96
Tldc1	TBC/LysM associated domain containing 1	ENSMUSG00000034105	17830	1,96
Cntf	ciliary neurotrophic factor	ENSMUSG00000079415	1973	1,96
		ENSMUSG00000030226	219051	1,96
Kctd19	potassium channel tetramerisation domain containing 19	ENSMUSG00000051648	30696	1,96
Stx11	syntaxin 11	ENSMUSG00000039232	24277	1,96
Colec10	collectin sub-family member 10	ENSMUSG00000038591	55585	1,96
4930404N11Rik	RIKEN cDNA 4930404N11 gene	ENSMUSG00000020234	2750	1,96
		ENSMUSG00000036144	73245	1,96
1700028J19Rik	RIKEN cDNA 1700028J19 gene	ENSMUSG00000038782	6190	1,96
Nhlrc4	NHL repeat containing 4	ENSMUSG00000090113	2729	1,96
Crb2	crumbs homolog 2 (Drosophila)	ENSMUSG00000035403	22855	1,96
Fgf6	fibroblast growth factor 6	ENSMUSG00000000183	9177	1,96
Klhl41	kelch-like 41	ENSMUSG00000075307	14111	1,96
		ENSMUSG00000041620	19809	1,96
		ENSMUSG00000096828	28803	1,96
		ENSMUSG00000078648	5614	1,96
4930412D23Rik	RIKEN cDNA 4930412D23 gene	ENSMUSG00000043569	15373	1,96
		ENSMUSG00000028011	18661	1,96
Ccdc36	coiled-coil domain containing 36	ENSMUSG00000047220	24843	1,96
Mmp25	matrix metallopeptidase 25	ENSMUSG00000023903	15812	1,96
Wdr64	WD repeat domain 64	ENSMUSG00000026523	117142	1,96
Lipk	lipase, family member K	ENSMUSG00000024771	39650	1,96
		ENSMUSG00000095795	28960	1,96
Cyp4f40	cytochrome P450, family 4, subfamily f, polypeptide 40	ENSMUSG00000090700	17278	1,96
Lingo4	leucine rich repeat and Ig domain containing 4	ENSMUSG00000044505	5985	1,96
Tecta	tectorin alpha	ENSMUSG00000037705	70311	1,96
Ctrb1	chymotrypsinogen B1	ENSMUSG00000031957	4501	1,96
Crtam	cytotoxic and regulatory T cell molecule	ENSMUSG00000032021	31876	1,96
Hrh4	histamine receptor H4	ENSMUSG00000037346	15893	1,96
Irg1	immunoresponsive gene 1	ENSMUSG00000022126	9597	1,96
Olfr1333	olfactory receptor 1333	ENSMUSG00000073770	6789	1,96
Olfr1031	olfactory receptor 1031	ENSMUSG00000043267	1011	1,96
Acta2	actin, alpha 2, smooth muscle, aorta	ENSMUSG00000035783	14247	1,88
Sox10	SRY-box containing gene 10	ENSMUSG00000033006	9578	1,88
Eva1b	eva-1 homolog B (C. elegans)	ENSMUSG00000050212	2132	1,88

Dkk2	dickkopf homolog 2 (Xenopus laevis)	ENSMUSG00000028031	95002	1,88
		ENSMUSG00000030725	2089	1,88
AI467606	expressed sequence AI467606	ENSMUSG00000045165	2614	1,88
Alx4	aristaless-like homeobox 4	ENSMUSG00000040310	38956	1,88
Apln	apelin	ENSMUSG00000037010	9708	1,88
Bbs10	Bardet-Biedl syndrome 10 (human)	ENSMUSG00000035759	3058	1,88
Gstk1	glutathione S-transferase kappa 1	ENSMUSG00000029864	4507	1,88
A230052G05Rik	RIKEN cDNA A230052G05 gene	ENSMUSG00000020887	2302	1,88
C3	complement component 3	ENSMUSG00000024164	24167	1,88
Itgad	integrin, alpha D	ENSMUSG00000070369	69441	1,88
Tmem88b	transmembrane protein 88B	ENSMUSG00000073680	4284	1,88
Otos	otospiralin	ENSMUSG00000044055	4624	1,88
Prss32	protease, serine, 32	ENSMUSG00000048992	15922	1,88
Ajuba	ajuba LIM protein	ENSMUSG00000022178	10193	1,88
Mmel1	membrane metallo-endopeptidase-like 1	ENSMUSG00000058183	25944	1,88
Htra2	HtrA serine peptidase 2	ENSMUSG00000068329	4008	1,88
Capg	capping protein (actin filament), gelsolin-like	ENSMUSG00000056737	18593	1,88
Csl	citrate synthase like	ENSMUSG00000046934	1954	1,88
Kcnj6	potassium inwardly-rectifying channel, subfamily J, member 6	ENSMUSG00000043301	248431	1,88
Pik3cg	phosphoinositide-3-kinase, catalytic, gamma polypeptide	ENSMUSG00000020573	35187	1,88
Ptgir	prostaglandin I receptor (IP)	ENSMUSG00000043017	4416	1,88
Pkp1	plakophilin 1	ENSMUSG00000026413	47813	1,88
Gmnc	geminin coiled-coil domain containing	ENSMUSG00000068428	32739	1,88
Fbxo40	F-box protein 40	ENSMUSG00000047746	27008	1,88
		ENSMUSG00000094814	48370	1,88
		ENSMUSG00000028186	35229	1,88
		ENSMUSG00000052760	61561	1,88
Ptrh1	peptidyl-tRNA hydrolase 1 homolog (S. cerevisiae)	ENSMUSG00000053746	1812	1,88
Nfkb2	nuclear factor of kappa light polypeptide gene enhancer in B cells 2, p49/p100	ENSMUSG00000025225	7354	1,88
Gm9817	predicted gene 9817	ENSMUSG00000047061	2531	1,88
Slc47a2	solute carrier family 47, member 2	ENSMUSG00000069855	41230	1,88
Zscan2	zinc finger and SCAN domain containing 2	ENSMUSG00000038797	15597	1,88
Car6	carbonic anhydrase 6	ENSMUSG00000028972	14318	1,88
2410017I17Rik	RIKEN cDNA 2410017I17 gene	ENSMUSG00000038311	17556	1,88
Hist1h4h	histone cluster 1, H4h	ENSMUSG00000060981	476	1,88
Pou2af1	POU domain, class 2, associating factor 1	ENSMUSG00000032053	26390	1,88
Spns3	spinster homolog 3	ENSMUSG00000020798	55588	1,88
Gjb6	gap junction protein, beta 6	ENSMUSG00000040055	10309	1,88
Gm5150	predicted gene 5150	ENSMUSG00000078780	58263	1,88
Gckr	glucokinase regulatory protein	ENSMUSG00000059434	29720	1,88
Ofcc1	orofacial cleft 1 candidate 1	ENSMUSG00000047094	286130	1,88
Poteg	POTE ankyrin domain family, member G	ENSMUSG00000063932	47503	1,88
Layn	layilin	ENSMUSG00000060594	20315	1,88
4930447C04Rik	RIKEN cDNA 4930447C04 gene	ENSMUSG00000021098	59725	1,88

Fas	Fas (TNF receptor superfamily member 6)	ENSMUSG00000024778	37112	1,88
Casp12	caspase 12	ENSMUSG00000025887	27603	1,88
AA467197	expressed sequence AA467197	ENSMUSG00000033213	4206	1,88
		ENSMUSG00000096916	19705	1,88
		ENSMUSG00000073623	351	1,88
		ENSMUSG00000097856	46737	1,88
Sult2a4	sulfotransferase family 2A, dehydroepiandrosterone (DHEA)-preferring, member 4	ENSMUSG00000074377	79949	1,88
Gm5444	predicted gene 5444	ENSMUSG00000053499	64837	1,88
P2rx5	purinergic receptor P2X, ligand-gated ion channel, 5	ENSMUSG00000005950	12265	1,88
Skint8	selection and upkeep of intraepithelial T cells 8	ENSMUSG00000078599	30967	1,88
Xpnpep2	X-prolyl aminopeptidase (aminopeptidase P) 2, membrane-bound	ENSMUSG00000037005	28257	1,88
Ankmy1	ankyrin repeat and MYND domain containing 1	ENSMUSG00000034212	32778	1,88
Zfp804b	zinc finger protein 804B	ENSMUSG00000092094	107494	1,88
		ENSMUSG00000038194	3502	1,88
Hormad2	HORMA domain containing 2	ENSMUSG00000020419	95292	1,88
Clec4d	C-type lectin domain family 4, member d	ENSMUSG00000030144	13162	1,88
Cdh24	cadherin-like 24	ENSMUSG00000059674	9373	1,88
4921504E06Rik	RIKEN cDNA 4921504E06 gene	ENSMUSG00000026734	91078	1,88
Gm10057	predicted gene 10057	ENSMUSG00000096867	11054	1,88
Gpr142	G protein-coupled receptor 142	ENSMUSG00000034677	7822	1,88
		ENSMUSG00000095280	3074	1,88
Olfr745	olfactory receptor 745	ENSMUSG00000050028	1175	1,88
Flt1	FMS-like tyrosine kinase 1	ENSMUSG00000029648	164408	1,81
Acp5	acid phosphatase 5, tartrate resistant	ENSMUSG00000001348	9016	1,81
Scd1	stearoyl-Coenzyme A desaturase 1	ENSMUSG00000037071	13259	1,81
9930013L23Rik	RIKEN cDNA 9930013L23 gene	ENSMUSG00000052353	153646	1,81
Arhgef15	Rho guanine nucleotide exchange factor (GEF) 15	ENSMUSG00000052921	14326	1,81
Ppp1r3c	protein phosphatase 1, regulatory (inhibitor) subunit 3C	ENSMUSG00000067279	4917	1,81
Dmrtc1a	DMRT-like family C1a	ENSMUSG00000031323	5472	1,81
Aldh1a3	aldehyde dehydrogenase family 1, subfamily A3	ENSMUSG00000015134	36626	1,81
Polrmt	polymerase (RNA) mitochondrial (DNA directed)	ENSMUSG00000020329	10459	1,81
Gstp2	glutathione S-transferase, pi 2	ENSMUSG00000038155	1815	1,81
Slc4a1	solute carrier family 4 (anion exchanger), member 1	ENSMUSG00000006574	17380	1,81
Csf2rb	colony stimulating factor 2 receptor, beta, low-affinity (granulocyte-macrophage)	ENSMUSG00000071713	25011	1,81
Csdc2	cold shock domain containing C2, RNA binding	ENSMUSG00000042109	14183	1,81
Itgb3	integrin beta 3	ENSMUSG00000020689	62477	1,81
		ENSMUSG00000057092	8533	1,81

Zbtb8b	zinc finger and BTB domain containing 8b	ENSMUSG00000048485	15089	1,81
C1qb	complement component 1, q subcomponent, beta polypeptide	ENSMUSG00000036905	6059	1,81
Tspan17	tetraspanin 17	ENSMUSG00000025875	7400	1,81
Zfp707	zinc finger protein 707	ENSMUSG00000034429	6746	1,81
Kcnh2	potassium voltage-gated channel, subfamily H (eag-related), member 2	ENSMUSG00000038319	32016	1,81
Vsig1	V-set and immunoglobulin domain containing 1	ENSMUSG00000031430	31865	1,81
Rdh9	retinol dehydrogenase 9	ENSMUSG00000056148	32911	1,81
Psd4	pleckstrin and Sec7 domain containing 4	ENSMUSG00000026979	41603	1,81
		ENSMUSG00000078532	42572	1,81
Fut4	fucosyltransferase 4	ENSMUSG00000049307	3664	1,81
Fam84a	family with sequence similarity 84, member A	ENSMUSG00000020607	4440	1,81
Lhpp	phospholysine phosphohistidine inorganic pyrophosphate phosphatase	ENSMUSG00000030946	95783	1,81
B3gat1	beta-1,3-glucuronyltransferase 1 (glucuronosyltransferase P)	ENSMUSG00000045994	29374	1,81
Gsg2	germ cell-specific gene 2	ENSMUSG00000050107	2810	1,81
		ENSMUSG00000031712	71586	1,81
Zfp939	zinc finger protein 939	ENSMUSG00000030424	24818	1,81
Lrrc17	leucine rich repeat containing 17	ENSMUSG00000039883	32374	1,81
Lctl	lactase-like	ENSMUSG00000032401	20972	1,81
Dgkk	diacylglycerol kinase kappa	ENSMUSG00000062393	169058	1,81
		ENSMUSG00000058748	3541	1,81
Lrmp	lymphoid-restricted membrane protein	ENSMUSG00000030263	59282	1,81
Atf7ip2	activating transcription factor 7 interacting protein 2	ENSMUSG00000039200	50340	1,81
Mpeg1	macrophage expressed gene 1	ENSMUSG00000046805	4506	1,81
Hs3st4	heparan sulfate (glucosamine) 3-O-sulfotransferase 4	ENSMUSG00000078591	416121	1,81
Ccdc42b	coiled-coil domain containing 42B	ENSMUSG00000094282	5901	1,81
Cdc45	cell division cycle 45	ENSMUSG00000000028	31541	1,81
Sema6b	sema domain, transmembrane domain (TM), and cytoplasmic domain, (semaphorin) 6B	ENSMUSG00000001227	17259	1,81
		ENSMUSG00000019762	14638	1,81
Serpinb8	serine (or cysteine) peptidase inhibitor, clade B, member 8	ENSMUSG00000026315	18904	1,81
Slc25a34	solute carrier family 25, member 34	ENSMUSG00000040740	4998	1,81
BC049715	cDNA sequence BC049715	ENSMUSG00000047515	11694	1,81
Satl1	spermidine/spermine N1-acetyl transferase-like 1	ENSMUSG00000025527	22477	1,81
Magea2	melanoma antigen, family A, 2	ENSMUSG00000055746	66466	1,81
Lrrc18	leucine rich repeat containing 18	ENSMUSG00000041673	23911	1,81
4833403I15Rik	RIKEN cDNA 4833403I15 gene	ENSMUSG00000024481	55408	1,81
Nlrp2	NLR family, pyrin domain containing 2	ENSMUSG00000035177	52482	1,81
C1rl	complement component 1, r subcomponent-like	ENSMUSG00000038527	17531	1,81

Acnat2	acyl-coenzyme A amino acid N-acyltransferase 2	ENSMUSG00000060317	28312	1,81
Cdrt4	CMT1A duplicated region transcript 4	ENSMUSG00000042200	41903	1,81
Gipc3	GIPC PDZ domain containing family, member 3	ENSMUSG00000034872	5505	1,81
Vmn2r87	vomeronasal 2, receptor 87	ENSMUSG00000091511	26048	1,81
Klhl10	kelch-like 10	ENSMUSG00000001558	15106	1,81
Gm527	predicted gene 527	ENSMUSG00000047227	6681	1,81
Zdhhc23	zinc finger, DHHC domain containing 23	ENSMUSG00000036304	10016	1,81
Tfap2e	transcription factor AP-2, epsilon	ENSMUSG00000042477	20235	1,81
Bpifb9a	BPI fold containing family B, member 9A	ENSMUSG00000067998	13392	1,81
		ENSMUSG00000045613	140331	1,81
Gp2	glycoprotein 2 (zymogen granule membrane)	ENSMUSG00000030954	16729	1,81
Klhl38	kelch-like 38	ENSMUSG00000022357	9597	1,81
Gm6471	predicted gene 6471	ENSMUSG00000073781	21040	1,81
		ENSMUSG00000079058	49053	1,81
Mrgpra2a	MAS-related GPR, member A2A	ENSMUSG00000093973	25812	1,81
Tmprss11a	transmembrane protease, serine 11a	ENSMUSG00000072845	58581	1,81
Ces1c	carboxylesterase 1C	ENSMUSG00000057400	32269	1,81
Gm5142	predicted gene 5142	ENSMUSG00000053868	20247	1,81
Gm10309	predicted gene 10309	ENSMUSG00000071036	7501	1,81
Samt1	spermatogenesis associated multipass transmembrane protein 1	ENSMUSG00000094802	11051	1,81
Trim69	tripartite motif-containing 69	ENSMUSG00000033368	18328	1,81
Lrrc72	leucine rich repeat containing 72	ENSMUSG00000020545	45054	1,81
Musk	muscle, skeletal, receptor tyrosine kinase	ENSMUSG00000057280	88344	1,81
		ENSMUSG00000044701	5932	1,81
Olfr1030	olfactory receptor 1030	ENSMUSG00000044923	5487	1,81
Sparcl1	SPARC-like 1	ENSMUSG00000029309	34978	1,74
Pdgfra	platelet derived growth factor receptor, alpha polypeptide	ENSMUSG00000029231	45914	1,74
Axl	AXL receptor tyrosine kinase	ENSMUSG00000002602	31433	1,74
Rasgef1a	RasGEF domain family, member 1A	ENSMUSG00000030134	25162	1,74
Rem2	rad and gem related GTP binding protein 2	ENSMUSG00000022176	4332	1,74
Lrfn3	leucine rich repeat and fibronectin type III domain containing 3	ENSMUSG00000036957	7259	1,74
Shc2	SHC (Src homology 2 domain containing) transforming protein 2	ENSMUSG00000020312	19979	1,74
C130074G19Rik	RIKEN cDNA C130074G19 gene	ENSMUSG00000039349	11293	1,74
Srm	spermidine synthase	ENSMUSG00000006442	3491	1,74
Cfh	complement component factor h	ENSMUSG00000026365	97557	1,74
Hrh3	histamine receptor H3	ENSMUSG00000039059	5024	1,74
Fcgr3	Fc receptor, IgG, low affinity III	ENSMUSG00000059498	13762	1,74
Calcrl	calcitonin receptor-like	ENSMUSG00000059588	94786	1,74
		ENSMUSG00000063166	357	1,74
Slc8a3	solute carrier family 8 (sodium/calcium exchanger), member 3	ENSMUSG00000079055	135266	1,74

Gm9791	predicted pseudogene 9791	ENSMUSG00000044434	733	1,74
Rapsn	receptor-associated protein of the synapse	ENSMUSG00000002104	10110	1,74
Sh2d4a	SH2 domain containing 4A	ENSMUSG00000053886	71128	1,74
Lrrc57	leucine rich repeat containing 57	ENSMUSG00000027286	5283	1,74
Pcdhb18	protocadherin beta 18	ENSMUSG00000048347	5039	1,74
		ENSMUSG00000091472	21402	1,74
		ENSMUSG00000031112	52051	1,74
Ccdc68	coiled-coil domain containing 68	ENSMUSG00000038903	44059	1,74
Dok2	docking protein 2	ENSMUSG00000022102	12460	1,74
Ccr2	chemokine (C-C motif) receptor 2	ENSMUSG00000049103	11608	1,74
		ENSMUSG00000094370	23242	1,74
Kpna7	karyopherin alpha 7 (importin alpha 8)	ENSMUSG00000038770	100512	1,74
Zfp456	zinc finger protein 456	ENSMUSG00000078995	30565	1,74
Cdh8	cadherin 8	ENSMUSG00000036510	392001	1,74
Zfp128	zinc finger protein 128	ENSMUSG00000060397	12246	1,74
9130008F23Rik	RIKEN cDNA 9130008F23 gene	ENSMUSG00000054951	5077	1,74
Trim21	tripartite motif-containing 21	ENSMUSG00000030966	7548	1,74
Glipr1l2	GLI pathogenesis-related 1 like 2	ENSMUSG00000020214	24745	1,74
Vsig10l	ZV-set and immunoglobulin domain containing 10 like	ENSMUSG00000070604	8763	1,74
Smim18	small integral membrane protein 18	ENSMUSG00000094500	5657	1,74
Rgag4	retrotransposon gag domain containing 4	ENSMUSG00000049191	4761	1,74
Pitpnm3	PITPNM family member 3	ENSMUSG00000040543	88251	1,74
		ENSMUSG00000056529	18617	1,74
Zfp786	zinc finger protein 786	ENSMUSG00000051499	11602	1,74
Yod1	YOD1 OTU deubiquitinating enzyme 1 homologue (S. cerevisiae)	ENSMUSG00000046404	4729	1,74
		ENSMUSG00000093803	1533	1,74
Itih3	inter-alpha trypsin inhibitor, heavy chain 3	ENSMUSG00000006522	15188	1,74
Abcg4	ATP-binding cassette, sub-family G (WHITE), member 4	ENSMUSG00000032131	15428	1,74
Lrit2	leucine-rich repeat, immunoglobulin-like and transmembrane domains 2	ENSMUSG00000043418	5687	1,74
Fndc8	fibronectin type III domain containing 8	ENSMUSG00000018844	8678	1,74
Tmem145	transmembrane protein 145	ENSMUSG00000043843	10090	1,74
		ENSMUSG00000069755	1046	1,74
Vipr1	vasoactive intestinal peptide receptor 1	ENSMUSG00000032528	30239	1,74
Ang	angiogenin, ribonuclease, RNase A family, 5	ENSMUSG00000072115	10931	1,74
Aim1l	absent in melanoma 1-like	ENSMUSG00000012123	26593	1,74
		ENSMUSG00000091007	940	1,74
Padi4	peptidyl arginine deiminase, type IV	ENSMUSG00000025330	28372	1,74
		ENSMUSG00000094230	140191	1,74
Vnn3	vanin 3	ENSMUSG00000020010	18382	1,74
Glrb	glycine receptor, beta subunit	ENSMUSG00000028020	70062	1,74
C6	complement component 6	ENSMUSG00000022181	88282	1,74
1600016N20Rik	RIKEN cDNA 1600016N20 gene	ENSMUSG00000025500	4040	1,74

Rspo2	R-spondin 2 homolog (Xenopus laevis)	ENSMUSG00000051920	150008	1,74
Cfhr2	complement factor H-related 2	ENSMUSG00000033898	48397	1,74
Havcr1	hepatitis A virus cellular receptor 1	ENSMUSG00000040405	44499	1,74
BC100451	cDNA sequence BC100451	ENSMUSG00000076433	10141	1,74
Lif	leukemia inhibitory factor	ENSMUSG00000034394	14958	1,74
		ENSMUSG00000029219	7780	1,74
Gdnf	glial cell line derived neurotrophic factor	ENSMUSG00000022144	26565	1,74
Kl	klotho	ENSMUSG00000058488	41203	1,74
Sult2a3	sulfotransferase family 2A, dehydroepiandrosterone (DHEA)-preferring, member 3	ENSMUSG00000074375	55593	1,74
Dok7	docking protein 7	ENSMUSG00000044716	31027	1,74
F2rl2	coagulation factor II (thrombin) receptor-like 2	ENSMUSG00000021675	5887	1,74
Vwce	von Willebrand factor C and EGF domains	ENSMUSG00000043789	30978	1,74
Hhatl	hedgehog acyltransferase-like	ENSMUSG00000032523	8492	1,74
Has1	hyaluronan synthase1	ENSMUSG00000003665	11861	1,74
Gm12689	predicted gene 12689	ENSMUSG00000070891	792	1,74
Cd164l2	CD164 sialomucin-like 2	ENSMUSG00000028865	12425	1,74
Anxa10	annexin A10	ENSMUSG00000031635	66077	1,74
1700011H14Rik	RIKEN cDNA 1700011H14 gene	ENSMUSG00000021850	19071	1,74
Gm10339	predicted gene 10339	ENSMUSG00000071591	37709	1,74
2310007B03Rik	RIKEN cDNA 2310007B03 gene	ENSMUSG00000034159	9600	1,74
Olfr399	olfactory receptor 399	ENSMUSG00000043692	4343	1,74
Inmt	indolethylamine N-methyltransferase	ENSMUSG00000003477	4418	1,67
Itih5	inter-alpha (globulin) inhibitor H5	ENSMUSG00000025780	102959	1,67
		ENSMUSG00000036545	205490	1,67
Scgn	secretagogin, EF-hand calcium binding protein	ENSMUSG00000021337	37759	1,67
		ENSMUSG00000073538	3603	1,67
5430419D17Rik	RIKEN cDNA 5430419D17 gene	ENSMUSG00000006204	76544	1,67
Fxyd2	FXYD domain-containing ion transport regulator 2	ENSMUSG00000059412	7141	1,67
		ENSMUSG00000090171	18397	1,67
Rnf5	ring finger protein 5	ENSMUSG00000015478	2600	1,67
Ccl9	chemokine (C-C motif) ligand 9	ENSMUSG00000019122	5718	1,67
Plekhh2	pleckstrin homology domain containing, family H (with MyTH4 domain) member 2	ENSMUSG00000040852	110248	1,67
		ENSMUSG00000037214	4729	1,67
6330409D20Rik	RIKEN cDNA 6330409D20 gene	ENSMUSG00000009551	8397	1,67
Eci3	enoyl-Coenzyme A delta isomerase 3	ENSMUSG00000021416	17197	1,67
Cdkn2c	cyclin-dependent kinase inhibitor 2C (p18, inhibits CDK4)	ENSMUSG00000028551	6314	1,67
Pnck	pregnancy upregulated non-ubiquitously expressed CaM kinase	ENSMUSG00000002012	4124	1,67
Zfp429	zinc finger protein 429	ENSMUSG00000078994	10459	1,67
		ENSMUSG00000029097	17865	1,67
		ENSMUSG00000067455	776	1,67
		ENSMUSG00000095681	22279	1,67

Map3k6	mitogen-activated protein kinase kinase kinase 6	ENSMUSG00000028862	12112	1,67
B3gnt4	UDP-GlcNAc:betaGal beta-1,3-N-acetylglucosaminyltransferase 4	ENSMUSG00000029431	1423	1,67
Ptpn22	protein tyrosine phosphatase, non-receptor type 22 (lymphoid)	ENSMUSG00000027843	52453	1,67
Hpn	hepsin	ENSMUSG00000001249	16566	1,67
Ccdc22	coiled-coil domain containing 22	ENSMUSG00000031143	11709	1,67
		ENSMUSG00000096923	1615	1,67
Pemt	phosphatidylethanolamine N-methyltransferase	ENSMUSG00000000301	75876	1,67
Rell2	RELT-like 2	ENSMUSG00000044024	4101	1,67
Tbxa2r	thromboxane A2 receptor	ENSMUSG00000034881	6440	1,67
Tonsl	tonsoku-like, DNA repair protein	ENSMUSG00000059323	13957	1,67
		ENSMUSG00000094437	360	1,67
		ENSMUSG00000095545	11424	1,67
Klhl4	kelch-like 4	ENSMUSG00000025597	86497	1,67
4930415F15Rik	RIKEN cDNA 4930415F15 gene	ENSMUSG00000020191	53099	1,67
Rsph6a	radial spoke head 6 homolog A (Chlamydomonas)	ENSMUSG00000040866	19758	1,67
Cpt1b	carnitine palmitoyltransferase 1b, muscle	ENSMUSG00000078937	9459	1,67
Zbtb32	zinc finger and BTB domain containing 32	ENSMUSG00000006310	9229	1,67
Alox12e	arachidonate lipoxygenase, epidermal	ENSMUSG00000018907	7019	1,67
Als2cl	ALS2 C-terminal like	ENSMUSG00000044037	20661	1,67
4933427D06Rik	RIKEN cDNA 4933427D06 gene	ENSMUSG00000055403	159348	1,67
5430403G16Rik	RIKEN cDNA 5430403G16 gene	ENSMUSG00000072763	16497	1,67
Rgs13	regulator of G-protein signaling 13	ENSMUSG00000051079	38706	1,67
Gp6	glycoprotein 6 (platelet)	ENSMUSG00000078810	29032	1,67
Clca1	chloride channel calcium activated 1	ENSMUSG00000056025	31301	1,67
Rdh16	retinol dehydrogenase 16	ENSMUSG00000069456	45414	1,67
Vmn2r57	vomeronasal 2, receptor 57	ENSMUSG00000066537	48910	1,67
Cyp2c68	cytochrome P450, family 2, subfamily c, polypeptide 68	ENSMUSG00000074882	52221	1,67
Tex40	testis expressed 40	ENSMUSG00000050623	2955	1,67
1700015F17Rik	RIKEN cDNA 1700015F17 gene	ENSMUSG00000079666	41317	1,67
Gm10283	predicted gene 10283	ENSMUSG00000070023	73175	1,67
Sis	sucrase isomaltase (alpha-glucosidase)	ENSMUSG00000027790	79307	1,67
Gm595	predicted gene 595	ENSMUSG00000079606	36248	1,67
Wdr20b	WD repeat domain 20b	ENSMUSG00000035560	2938	1,67
4930468A15Rik	RIKEN cDNA 4930468A15 gene	ENSMUSG00000079525	20315	1,67
Eqtn	equatorin, sperm acrosome associated	ENSMUSG00000028575	21577	1,67
Gbp5	guanylate binding protein 5	ENSMUSG00000040264	25411	1,67
Slc51a	solute carrier family 51, alpha subunit	ENSMUSG00000035699	12358	1,67
Clec4b1	C-type lectin domain family 4, member b1	ENSMUSG00000030147	21594	1,67
Spaca7	sperm acrosome associated 7	ENSMUSG00000010435	27690	1,67
Olfr1437	olfactory receptor 1437	ENSMUSG00000096436	939	1,67
Olfr974	olfactory receptor 974	ENSMUSG00000051493	1080	1,67
Cldn11	claudin 11	ENSMUSG00000037625	14379	1,59

Fbln1	fibulin 1	ENSMUSG00000006369	80587	1,59
Afap1l2	actin filament associated protein 1-like 2	ENSMUSG00000025083	95868	1,59
Spry4	sprouty homolog 4 (Drosophila)	ENSMUSG00000024427	15001	1,59
Sult2a6	sulfotransferase family 2A, dehydroepiandrosterone (DHEA)-preferring, member 6	ENSMUSG00000070810	32487	1,59
Abcb1a	ATP-binding cassette, sub-family B (MDR/TAP), member 1A	ENSMUSG00000040584	88499	1,59
Duxbl3	double homeobox B-like 3	ENSMUSG00000072672	11475	1,59
Igdcc4	immunoglobulin superfamily, DCC subclass, member 4	ENSMUSG00000032816	36455	1,59
		ENSMUSG00000078936	4718	1,59
Prdm9	PR domain containing 9	ENSMUSG00000051977	21276	1,59
Pcdhb22	protocadherin beta 22	ENSMUSG00000073591	3067	1,59
Tnfrsf9	tumor necrosis factor receptor superfamily, member 9	ENSMUSG00000028965	31541	1,59
Cbln2	cerebellin 2 precursor protein	ENSMUSG00000024647	7174	1,59
Gbp3	guanylate binding protein 3	ENSMUSG00000028268	13158	1,59
Gbp7	guanylate binding protein 7	ENSMUSG00000040253	19746	1,59
Slc38a4	solute carrier family 38, member 4	ENSMUSG00000022464	61134	1,59
Tmem221	transmembrane protein 221	ENSMUSG00000043664	4569	1,59
4930426L09Rik	RIKEN cDNA 4930426L09 gene	ENSMUSG00000026736	1459	1,59
Henmt1	HEN1 methyltransferase homolog 1 (Arabidopsis)	ENSMUSG00000045662	20690	1,59
Zfp773	zinc finger protein 773	ENSMUSG00000063535	6078	1,59
Serpina1e	serine (or cysteine) peptidase inhibitor, clade A, member 1E	ENSMUSG00000072849	12045	1,59
Arl10	ADP-ribosylation factor-like 10	ENSMUSG00000025870	6114	1,59
Ret	ret proto-oncogene	ENSMUSG00000030110	45997	1,59
Itga2	integrin alpha 2	ENSMUSG00000015533	96126	1,59
Cav1	caveolin 1, caveolae protein	ENSMUSG00000007655	35118	1,59
Cyp46a1	cytochrome P450, family 46, subfamily a, polypeptide 1	ENSMUSG00000021259	27855	1,59
Fam151a	family with sequence simliarity 151, member A	ENSMUSG00000034871	14404	1,59
		ENSMUSG00000035868	4959	1,59
D330045A20Rik	RIKEN cDNA D330045A20 gene	ENSMUSG00000042498	74216	1,59
Syngr4	synaptogyrin 4	ENSMUSG00000040231	11493	1,59
6030419C18Rik	RIKEN cDNA 6030419C18 gene	ENSMUSG00000066607	11140	1,59
Nostrin	nitric oxide synthase trafficker	ENSMUSG00000034738	53531	1,59
Rimbp2	RIMS binding protein 2	ENSMUSG00000029420	192962	1,59
Gna15	guanine nucleotide binding protein, alpha 15	ENSMUSG00000034792	21920	1,59
Csf3r	colony stimulating factor 3 receptor (granulocyte)	ENSMUSG00000028859	19891	1,59
Cd97	CD97 antigen	ENSMUSG00000002885	18076	1,59
Cfp	complement factor properdin	ENSMUSG00000001128	6102	1,59
Sfmbt2	Scm-like with four mbt domains 2	ENSMUSG00000061186	224744	1,59
Sall4	sal-like 4 (Drosophila)	ENSMUSG00000027547	19612	1,59
Gm5134	predicted gene 5134	ENSMUSG00000033255	55078	1,59
Rasgrp3	RAS, guanyl releasing protein 3	ENSMUSG00000071042	93139	1,59
		ENSMUSG00000074224	18853	1,59
Hsd11b1	hydroxysteroid 11-beta dehydrogenase 1	ENSMUSG00000016194	42425	1,59

Ifit3	interferon-induced protein with tetratricopeptide repeats 3	ENSMUSG00000074896	5201	1,59
Insm2	insulinoma-associated 2	ENSMUSG00000045440	3102	1,59
Galnt9	UDP-N-acetyl-alpha-D-galactosamine:polypeptide N-acetylgalactosaminyltransferase 9	ENSMUSG00000033316	77038	1,59
Cyp2a22	cytochrome P450, family 2, subfamily a, polypeptide 22	ENSMUSG00000091867	7756	1,59
Pebp4	phosphatidylethanolamine binding protein 4	ENSMUSG00000022085	219467	1,59
		ENSMUSG00000041544	47702	1,59
Gpr34	G protein-coupled receptor 34	ENSMUSG00000040229	8770	1,59
Gm5916	predicted gene 5916	ENSMUSG00000091411	8846	1,59
4921524L21Rik	RIKEN cDNA 4921524L21 gene	ENSMUSG00000039540	35334	1,59
Vmn2r58	vomeronasal 2, receptor 58	ENSMUSG00000090383	35790	1,59
Morn5	MORN repeat containing 5	ENSMUSG00000026894	30261	1,59
Frmpd3	FERM and PDZ domain containing 3	ENSMUSG00000042425	27047	1,59
Crb1	crumbs homolog 1 (Drosophila)	ENSMUSG00000063681	180045	1,59
Foxa3	forkhead box A3	ENSMUSG00000040891	10257	1,59
Ano7	anoctamin 7	ENSMUSG00000034107	29607	1,59
Cplx1	complexin 1	ENSMUSG00000033615	31471	1,59
Klhl40	kelch-like 40	ENSMUSG00000074001	6213	1,59
Grap2	GRB2-related adaptor protein 2	ENSMUSG00000042351	27055	1,59
Gm15319	predicted gene 15319	ENSMUSG00000074449	25547	1,59
Barhl1	BarH-like 1 (Drosophila)	ENSMUSG00000026805	8990	1,59
		ENSMUSG00000069273	836	1,59
Fcamr	Fc receptor, IgA, IgM, high affinity	ENSMUSG00000026415	13839	1,59
Upk3b	uroplakin 3B	ENSMUSG00000042985	6808	1,59
Defb48	defensin beta 48	ENSMUSG00000075574	6987	1,59
Olfml2b	olfactomedin-like 2B	ENSMUSG00000038463	38258	1,59
Olfr299	olfactory receptor 299	ENSMUSG00000020168	993	1,59
Col2a1	collagen, type II, alpha 1	ENSMUSG00000022483	29094	1,52
Lum	lumican	ENSMUSG00000036446	7203	1,52
Gata6	GATA binding protein 6	ENSMUSG00000005836	33126	1,52
Tnni3k	TNNI3 interacting kinase	ENSMUSG00000040086	269106	1,52
Cd52	CD52 antigen	ENSMUSG00000000682	12635	1,52
		ENSMUSG00000090330	1571	1,52
Hpdl	4-hydroxyphenylpyruvate dioxygenase-like	ENSMUSG00000043155	1602	1,52
Fxyd5	FXYD domain-containing ion transport regulator 5	ENSMUSG00000009687	9760	1,52
Cubn	cubilin (intrinsic factor-cobalamin receptor)	ENSMUSG00000026726	215587	1,52
Bloc1s3	biogenesis of lysosome-related organelles complex-1, subunit 3	ENSMUSG00000057667	2518	1,52
Fam129c	family with sequence similarity 129, member C	ENSMUSG00000043243	10289	1,52
Prr11	proline rich 11	ENSMUSG00000020493	19556	1,52
		ENSMUSG00000036073	3468	1,52
Gm9938	predicted gene 9938	ENSMUSG00000054178	1756	1,52
Pcdhb9	protocadherin beta 9	ENSMUSG00000051242	3055	1,52
		ENSMUSG00000078722	74844	1,52
		ENSMUSG00000091423	2375	1,52
Gm10406	predicted gene 10406	ENSMUSG00000093985	21335	1,52

Ampd2	adenosine monophosphate deaminase 2	ENSMUSG00000027889	12590	1,52
Adamtsl5	ADAMTS-like 5	ENSMUSG00000043822	9303	1,52
		ENSMUSG00000037649	3920	1,52
Foxred2	FAD-dependent oxidoreductase domain containing 2	ENSMUSG00000016552	16201	1,52
Ap5b1	adaptor-related protein complex 5, beta 1 subunit	ENSMUSG00000049562	3188	1,52
Gm1673	predicted gene 1673	ENSMUSG00000070858	1577	1,52
Chd5	chromodomain helicase DNA binding protein 5	ENSMUSG00000005045	51544	1,52
		ENSMUSG00000021101	6195	1,52
Mdga1	MAM domain containing glycosylphosphatidylinositol anchor 1	ENSMUSG00000043557	142132	1,52
Cpvl	carboxypeptidase, vitellogenic-like	ENSMUSG00000052955	105284	1,52
		ENSMUSG00000072774	14615	1,52
Gm7102	predicted gene 7102	ENSMUSG00000094649	1624	1,52
Serpinb9	serine (or cysteine) peptidase inhibitor, clade B, member 9	ENSMUSG00000045827	14708	1,52
Fam115c	family with sequence similarity 115, member C	ENSMUSG00000029851	22236	1,52
Actg2	actin, gamma 2, smooth muscle, enteric	ENSMUSG00000059430	23361	1,52
9930012K11Rik	RIKEN cDNA 9930012K11 gene	ENSMUSG00000044551	5098	1,52
		ENSMUSG00000069581	57555	1,52
Mfsd3	major facilitator superfamily domain containing 3	ENSMUSG00000019080	2698	1,52
		ENSMUSG00000096914	186340	1,52
Efcab9	EF-hand calcium binding domain 9	ENSMUSG00000044056	9331	1,52
Kcnk13	potassium channel, subfamily K, member 13	ENSMUSG00000045404	98184	1,52
		ENSMUSG00000068962	6728	1,52
Il7r	interleukin 7 receptor	ENSMUSG00000003882	23716	1,52
Spata2l	spermatogenesis associated 2-like	ENSMUSG00000033594	3955	1,52
		ENSMUSG00000022683	15927	1,52
Selplg	selectin, platelet (p-selectin) ligand	ENSMUSG00000048163	12704	1,52
Cass4	Cas scaffolding protein family member 4	ENSMUSG00000074570	39964	1,52
Syngap1	synaptic Ras GTPase activating protein 1 homolog (rat)	ENSMUSG00000067629	29194	1,52
Sh2d7	SH2 domain containing 7	ENSMUSG00000046460	6037	1,52
Ces5a	carboxylesterase 5A	ENSMUSG00000058019	36495	1,52
Slc25a45	solute carrier family 25, member 45	ENSMUSG00000024818	7959	1,52
		ENSMUSG00000074158	33063	1,52
Ccr4	chemokine (C-C motif) receptor 4	ENSMUSG00000047898	6229	1,52
Adm	adrenomedullin	ENSMUSG00000030790	2151	1,52
Slc16a12	solute carrier family 16 (monocarboxylic acid transporters), member 12	ENSMUSG00000009378	78887	1,52
		ENSMUSG00000095294	24316	1,52
Vmn2r3	vomeronasal 2, receptor 3	ENSMUSG00000091572	28535	1,52
		ENSMUSG00000031497	28599	1,52
Cyp19a1	cytochrome P450, family 19, subfamily a, polypeptide 1	ENSMUSG00000032274	27506	1,52
		ENSMUSG00000049971	76493	1,52

		ENSMUSG00000070708	746	1,52
Pdzd3	PDZ domain containing 3	ENSMUSG00000032105	4153	1,52
Snx31	sorting nexin 31	ENSMUSG00000013611	51512	1,52
Myom2	myomesin 2	ENSMUSG00000031461	75889	1,52
LOC100038947	signal-regulatory protein beta 1-like	ENSMUSG00000074677	53384	1,52
Cacna1f	calcium channel, voltage-dependent, alpha 1F subunit	ENSMUSG00000031142	28114	1,52
Tmem247	transmembrane protein 247	ENSMUSG00000037689	5019	1,52
Adora2a	adenosine A2a receptor	ENSMUSG00000020178	17846	1,52
		ENSMUSG00000074414	7446	1,52
Cd200r2	Cd200 receptor 2	ENSMUSG00000090176	48744	1,52
		ENSMUSG00000028599	33408	1,52
Cyp4a12a	cytochrome P450, family 4, subfamily a, polypeptide 12a	ENSMUSG00000066071	33770	1,52
Ppapdc1a	phosphatidic acid phosphatase type 2 domain containing 1A	ENSMUSG00000070366	134214	1,52
Slco1b2	solute carrier organic anion transporter family, member 1b2	ENSMUSG00000030236	57129	1,52
Asb11	ankyrin repeat and SOCS box-containing 11	ENSMUSG00000031382	22177	1,52
Haao	3-hydroxyanthranilate 3,4-dioxygenase	ENSMUSG00000000673	15435	1,52
		ENSMUSG00000091115	874	1,52
		ENSMUSG00000050241	7932	1,52
Tas1r2	taste receptor, type 1, member 2	ENSMUSG00000028738	16743	1,52
4930447A16Rik	RIKEN cDNA 4930447A16 gene	ENSMUSG00000022288	15091	1,52
Serpind1	serine (or cysteine) peptidase inhibitor, clade D, member 1	ENSMUSG00000022766	12205	1,52
5430421N21Rik	RIKEN cDNA 5430421N21 gene	ENSMUSG00000067613	6382	1,52
Ccdc121	coiled-coil domain containing 121	ENSMUSG00000050625	1819	1,52
4930511M11Rik	RIKEN cDNA 4930511M11 gene	ENSMUSG00000014529	23322	1,52
Rln1	relaxin 1	ENSMUSG00000039097	3501	1,52
Ubqlnl	ubiquilin-like	ENSMUSG00000051437	2298	1,52
F11	coagulation factor XI	ENSMUSG00000031645	20862	1,52
Ern2	endoplasmic reticulum (ER) to nucleus signalling 2	ENSMUSG00000030866	16324	1,52
Slco6c1	solute carrier organic anion transporter family, member 6c1	ENSMUSG00000026331	69216	1,52
Olfr1362	olfactory receptor 1362	ENSMUSG00000051258	985	1,52
Omd	osteomodulin	ENSMUSG00000048368	9844	1,45
		ENSMUSG00000001497	21056	1,45
Lcn2	lipocalin 2	ENSMUSG00000026822	3165	1,45
Sprn	shadow of prion protein	ENSMUSG00000045733	4250	1,45
Ace	angiotensin I converting enzyme (peptidyl-dipeptidase A) 1	ENSMUSG00000020681	22020	1,45
Sapcd2	suppressor APC domain containing 2	ENSMUSG00000026955	5893	1,45
C1qc	complement component 1, q subcomponent, C chain	ENSMUSG00000036896	3262	1,45
Tfap2c	transcription factor AP-2, gamma	ENSMUSG00000028640	9030	1,45
Foxn4	forkhead box N4	ENSMUSG00000042002	19644	1,45
Ephx2	epoxide hydrolase 2, cytoplasmic	ENSMUSG00000022040	40124	1,45
Slc29a2	solute carrier family 29 (nucleoside transporters), member 2	ENSMUSG00000024891	7966	1,45
		ENSMUSG00000079022	238433	1,45
Slc47a1	solute carrier family 47, member 1	ENSMUSG00000010122	34945	1,45

4632428N05Rik	RIKEN cDNA 4632428N05 gene	ENSMUSG00000020101	25834	1,45
Enpep	glutamyl aminopeptidase	ENSMUSG00000028024	63546	1,45
Lrrc4b	leucine rich repeat containing 4B	ENSMUSG00000047085	34334	1,45
Actn3	actinin alpha 3	ENSMUSG00000006457	16687	1,45
Gstt1	glutathione S-transferase, theta 1	ENSMUSG00000001663	14772	1,45
Gm10483	predicted gene 10483	ENSMUSG00000073231	2746	1,45
Itga5	integrin alpha 5 (fibronectin receptor alpha)	ENSMUSG00000000555	22475	1,45
Vmn2r97	vomeronasal 2, receptor 97	ENSMUSG00000091491	33750	1,45
		ENSMUSG00000089857	7747	1,45
Jak3	Janus kinase 3	ENSMUSG00000031805	14280	1,45
Zbtbd6	kelch repeat and BTB (POZ) domain containing 6	ENSMUSG00000075502	2982	1,45
Cdhr2	cadherin-related family member 2	ENSMUSG00000034918	35202	1,45
Cmtm1	CKLF-like MARVEL transmembrane domain containing 1	ENSMUSG00000031876	28799	1,45
Baiap2l2	BAI1-associated protein 2-like 2	ENSMUSG00000018126	27343	1,45
Themis2	thymocyte selection associated family member 2	ENSMUSG00000037731	14545	1,45
1700023F06Rik	RIKEN cDNA 1700023F06 gene	ENSMUSG00000020940	9613	1,45
Ptprc	protein tyrosine phosphatase, receptor type, C	ENSMUSG00000026395	112846	1,45
Slc6a4	solute carrier family 6 (neurotransmitter transporter, serotonin), member 4	ENSMUSG00000020838	33738	1,45
Cfb	complement factor B	ENSMUSG00000090231	6145	1,45
Usp27x	ubiquitin specific peptidase 27, X chromosome	ENSMUSG00000046269	4555	1,45
Fgl2	fibrinogen-like protein 2	ENSMUSG00000039899	5733	1,45
Apbb1ip	amyloid beta (A4) precursor protein-binding, family B, member 1 interacting protein	ENSMUSG00000026786	101560	1,45
Vmn2r20	vomeronasal 2, receptor 20	ENSMUSG00000094145	32800	1,45
1700001C19Rik	RIKEN cDNA 1700001C19 gene	ENSMUSG00000047150	24643	1,45
		ENSMUSG00000037216	5650	1,45
		ENSMUSG00000007653	209272	1,45
Dfnb59	deafness, autosomal recessive 59 (human)	ENSMUSG00000075267	10081	1,45
Gm10874	predicted gene 10874	ENSMUSG00000075591	24569	1,45
Gm8052	predicted gene 8052	ENSMUSG00000079391	22320	1,45
Il1rl1	interleukin 1 receptor-like 1	ENSMUSG00000026069	35846	1,45
Gm4070	predicted gene 4070	ENSMUSG00000078606	58829	1,45
Sh3bgr	SH3-binding domain glutamic acid-rich protein	ENSMUSG00000040666	28464	1,45
Flt3	FMS-like tyrosine kinase 3	ENSMUSG00000042817	69749	1,45
Tfr2	transferrin receptor 2	ENSMUSG00000029716	17631	1,45
Tmem30c	transmembrane protein 30C	ENSMUSG00000022753	26727	1,45
Aadat	aminoadipate aminotransferase	ENSMUSG00000057228	39554	1,45
P2rx2	purinergic receptor P2X, ligand-gated ion channel, 2	ENSMUSG00000029503	3224	1,45
		ENSMUSG00000097449	27563	1,45
Irf8	interferon regulatory factor 8	ENSMUSG00000041515	20337	1,45
Slc13a4	solute carrier family 13 (sodium/sulfate symporters), member 4	ENSMUSG00000029843	40175	1,45

Asz1	ankyrin repeat, SAM and basic leucine zipper domain containing 1	ENSMUSG00000010796	58098	1,45
Asphd1	aspartate beta-hydroxylase domain containing 1	ENSMUSG00000046378	4016	1,45
Vmn2r83	vomeronasal 2, receptor 83	ENSMUSG00000091381	23212	1,45
Lingo3	leucine rich repeat and Ig domain containing 3	ENSMUSG00000051067	11233	1,45
Npffr1	neuropeptide FF receptor 1	ENSMUSG00000020090	30902	1,45
Slc28a2	solute carrier family 28 (sodium-coupled nucleoside transporter), member 2	ENSMUSG00000027219	34661	1,45
Kcnt1	potassium channel, subfamily T, member 1	ENSMUSG00000058740	54479	1,45
Ppp1r14d	protein phosphatase 1, regulatory (inhibitor) subunit 14D	ENSMUSG00000027317	11788	1,45
Ido2	indoleamine 2,3-dioxygenase 2	ENSMUSG00000031549	44442	1,45
Gpr183	G protein-coupled receptor 183	ENSMUSG00000051212	12863	1,45
Fam46d	family with sequence similarity 46, member D	ENSMUSG00000073007	80320	1,45
Rbm44	RNA binding motif protein 44	ENSMUSG00000070732	25693	1,45
		ENSMUSG00000093903	126356	1,45
Lyzl4	lysozyme-like 4	ENSMUSG00000032530	64254	1,45
Vwc2l	von Willebrand factor C domain-containing protein 2-like	ENSMUSG00000045648	159683	1,45
Gm10277	predicted gene 10277	ENSMUSG00000069804	2295	1,45
Vmn2r34	vomeronasal 2, receptor 34	ENSMUSG00000070841	18627	1,45
Zc2hc1b	zinc finger, C2HC-type containing 1B	ENSMUSG00000019815	28452	1,45
Dio1	deiodinase, iodothyronine, type I	ENSMUSG00000034785	15705	1,45
Ubqln3	ubiquilin 3	ENSMUSG00000051618	2657	1,45
Ccl3	chemokine (C-C motif) ligand 3	ENSMUSG00000000982	1512	1,45
Olfr976	olfactory receptor 976	ENSMUSG00000047352	2813	1,45
Olfr368	olfactory receptor 368	ENSMUSG00000049018	985	1,45
Mfap4	microfibrillar-associated protein 4	ENSMUSG00000042436	3470	1,38
Mcam	melanoma cell adhesion molecule	ENSMUSG00000032135	8259	1,38
Cxcl14	chemokine (C-X-C motif) ligand 14	ENSMUSG00000021508	7905	1,38
Vgll3	vestigial like 3 (Drosophila)	ENSMUSG00000091243	47425	1,38
Tyrobp	TYRO protein tyrosine kinase binding protein	ENSMUSG00000030579	3790	1,38
Flt4	FMS-like tyrosine kinase 4	ENSMUSG00000020357	43477	1,38
Btbd6	BTB (POZ) domain containing 6	ENSMUSG00000002803	2449	1,38
Scube2	signal peptide, CUB domain, EGF-like 2	ENSMUSG00000007279	67004	1,38
		ENSMUSG00000052821	29334	1,38
Akr1b8	aldo-keto reductase family 1, member B8	ENSMUSG00000029762	14345	1,38
Lpar2	lysophosphatidic acid receptor 2	ENSMUSG00000031861	8674	1,38
Aif1	allograft inflammatory factor 1	ENSMUSG00000024397	5078	1,38
Zfp454	zinc finger protein 454	ENSMUSG00000048728	14932	1,38
Mical1	microtubule associated monooxygenase, calponin and LIM domain containing 1	ENSMUSG00000019823	10719	1,38
Spata4	spermatogenesis associated 4	ENSMUSG00000031518	9318	1,38
		ENSMUSG00000094374	1119	1,38
Gm3696	predicted gene 3696	ENSMUSG00000092167	17414	1,38
Kif7	kinesin family member 7	ENSMUSG00000050382	16088	1,38

Rgs19	regulator of G-protein signaling 19	ENSMUSG00000002458	5559	1,38
Gp1ba	glycoprotein 1b, alpha polypeptide	ENSMUSG00000050675	2915	1,38
Tpcn2	two pore segment channel 2	ENSMUSG00000048677	30005	1,38
		ENSMUSG00000091275	22521	1,38
Mboat4	membrane bound O-acyltransferase domain containing 4	ENSMUSG00000071113	10156	1,38
		ENSMUSG00000060586	10317	1,38
Trim67	tripartite motif-containing 67	ENSMUSG00000036913	41695	1,38
Map4k1	mitogen-activated protein kinase kinase kinase kinase 1	ENSMUSG00000037337	20426	1,38
Slc25a47	solute carrier family 25, member 47	ENSMUSG00000048856	5686	1,38
		ENSMUSG00000094900	32794	1,38
Nxpe2	neurexophilin and PC-esterase domain family, member 2	ENSMUSG00000032028	35449	1,38
Zfp352	zinc finger protein 352	ENSMUSG00000070902	6883	1,38
		ENSMUSG00000091617	17401	1,38
Pik3r6	phosphoinositide-3-kinase, regulatory subunit 6	ENSMUSG00000046207	49680	1,38
Adam21	a disintegrin and metallopeptidase domain 21	ENSMUSG00000008438	9891	1,38
		ENSMUSG00000045110	1131	1,38
Cbs	cystathionine beta-synthase	ENSMUSG00000024039	24577	1,38
Mtcp1	mature T cell proliferation 1	ENSMUSG00000031200	6145	1,38
		ENSMUSG00000053164	4272	1,38
Zfp819	zinc finger protein 819	ENSMUSG00000055102	11117	1,38
Mep1b	meprin 1 beta	ENSMUSG00000024313	27856	1,38
		ENSMUSG00000074899	39616	1,38
Sez6l	seizure related 6 homolog like	ENSMUSG00000058153	158048	1,38
		ENSMUSG00000078897	13508	1,38
Ccdc175	coiled-coil domain containing 175	ENSMUSG00000021086	83738	1,38
		ENSMUSG00000006056	12639	1,38
Slc22a1	solute carrier family 22 (organic cation transporter), member 1	ENSMUSG00000023829	26965	1,38
Igtp	interferon gamma induced GTPase	ENSMUSG00000078853	8036	1,38
Robo3	roundabout homolog 3 (Drosophila)	ENSMUSG00000032128	17495	1,38
Slc17a1	solute carrier family 17 (sodium phosphate), member 1	ENSMUSG00000021335	27981	1,38
3425401B19Rik	RIKEN cDNA 3425401B19 gene	ENSMUSG00000071540	26154	1,38
F830002L21Rik	RIKEN cDNA F830002L21 gene	ENSMUSG00000075184	37490	1,38
Tmprss9	transmembrane protease, serine 9	ENSMUSG00000059406	19722	1,38
Dmgdh	dimethylglycine dehydrogenase precursor	ENSMUSG00000042102	78399	1,38
Catsperg2	catsper channel auxiliary subunit gamma 2	ENSMUSG00000049123	29797	1,38
Xaf1	XIAP associated factor 1	ENSMUSG00000040483	12105	1,38
		ENSMUSG00000025279	30041	1,38
Chrm4	cholinergic receptor, muscarinic 4	ENSMUSG00000040495	7641	1,38
Insl5	insulin-like 5	ENSMUSG00000066090	8971	1,38
C87414	expressed sequence C87414	ENSMUSG00000070686	36559	1,38
		ENSMUSG00000079265	57253	1,38
Glt6d1	glycosyltransferase 6 domain containing 1	ENSMUSG00000036401	21990	1,38
Rad51ap2	RAD51 associated protein 2	ENSMUSG00000086022	6850	1,38
Tgm1	transglutaminase 1, K polypeptide	ENSMUSG00000022218	13484	1,38
Gpr111	G protein-coupled receptor 111	ENSMUSG00000057899	33244	1,38

Npffr2	neuropeptide FF receptor 2	ENSMUSG00000035528	56312	1,38
Fam71e2	family with sequence similarity 71, member E2	ENSMUSG00000092518	18077	1,38
Vmn2r55	vomeronasal 2, receptor 55	ENSMUSG00000091045	33286	1,38
Catsper4	cation channel, sperm associated 4	ENSMUSG00000048003	15414	1,38
Renbp	renin binding protein	ENSMUSG00000031387	8730	1,38
		ENSMUSG00000091204	32254	1,38
Trem3	triggering receptor expressed on myeloid cells 3	ENSMUSG00000041754	11065	1,38
Foxf1	forkhead box F1	ENSMUSG00000042812	3759	1,38
Gc	group specific component	ENSMUSG00000035540	40388	1,38
Slc22a19	solute carrier family 22 (organic anion transporter), member 19	ENSMUSG00000024757	38250	1,38
4931440F15Rik	RIKEN cDNA 4931440F15 gene	ENSMUSG00000078157	3274	1,38
Lgals2	lectin, galactose-binding, soluble 2	ENSMUSG00000043501	4668	1,38
		ENSMUSG00000037406	13512	1,38
Mx1	myxovirus (influenza virus) resistance 1	ENSMUSG00000000386	15873	1,38
Tdrd6	tudor domain containing 6	ENSMUSG00000040140	14965	1,38
		ENSMUSG00000067724	24520	1,38
		ENSMUSG00000096519	1205	1,38
Tmc8	transmembrane channel-like gene family 8	ENSMUSG00000050106	11035	1,38
Vmn2r17	vomeronasal 2, receptor 17	ENSMUSG00000091879	33375	1,38
Zar1l	zygote arrest 1-like	ENSMUSG00000056586	11110	1,38
Naip6	NLR family, apoptosis inhibitory protein 6	ENSMUSG00000078942	36554	1,38
Olfr360	olfactory receptor 360	ENSMUSG00000083361	3515	1,38
Olfr411	olfactory receptor 411	ENSMUSG00000054406	5998	1,38
Olfr598	olfactory receptor 598	ENSMUSG00000073951	960	1,38
Olfr1511	olfactory receptor 1511	ENSMUSG00000063867	957	1,38
Fkbp10	FK506 binding protein 10	ENSMUSG00000001555	9128	1,30
Rasl11a	RAS-like, family 11, member A	ENSMUSG00000029641	2656	1,30
Gng11	guanine nucleotide binding protein (G protein), gamma 11	ENSMUSG00000032766	4503	1,30
Acot10	acyl-CoA thioesterase 10	ENSMUSG00000047565	1532	1,30
Klf15	Kruppel-like factor 15	ENSMUSG00000030087	12663	1,30
Kctd14	potassium channel tetramerisation domain containing 14	ENSMUSG00000051727	8220	1,30
4930579G24Rik	RIKEN cDNA 4930579G24 gene	ENSMUSG00000027811	3739	1,30
		ENSMUSG00000023336	21700	1,30
Fcgr1	Fc receptor, IgG, high affinity I	ENSMUSG00000015947	11061	1,30
Cilp	cartilage intermediate layer protein, nucleotide pyrophosphohydrolase	ENSMUSG00000042254	15426	1,30
Tmem26	transmembrane protein 26	ENSMUSG00000060044	58909	1,30
Krt26	keratin 26	ENSMUSG00000075570	9417	1,30
Gpr61	G protein-coupled receptor 61	ENSMUSG00000046793	6562	1,30
Rab3il1	RAB3A interacting protein (rabin3)-like 1	ENSMUSG00000024663	36712	1,30
BC048403	cDNA sequence BC048403	ENSMUSG00000053684	12942	1,30
Zfp661	zinc finger protein 661	ENSMUSG00000034800	12433	1,30
Zfp418	zinc finger protein 418	ENSMUSG00000034538	12233	1,30
Ehhadh	enoyl-Coenzyme A, hydratase/3-hydroxyacyl Coenzyme A dehydrogenase	ENSMUSG00000022853	26558	1,30

Aaed1	AhpC/TSA antioxidant enzyme domain containing 1	ENSMUSG00000021482	20875	1,30
Tmprss7	transmembrane serine protease 7	ENSMUSG00000033177	37344	1,30
		ENSMUSG00000096998	35262	1,30
Fez1	fasciculation and elongation protein zeta 1 (zygin I)	ENSMUSG00000032118	56986	1,30
Gm14085	predicted gene 14085	ENSMUSG00000079071	43100	1,30
Ahsg	alpha-2-HS-glycoprotein	ENSMUSG00000022868	7397	1,30
		ENSMUSG00000068604	956	1,30
		ENSMUSG00000091890	2025	1,30
Twist2	twist basic helix-loop-helix transcription factor 2	ENSMUSG00000007805	46568	1,30
Plekhd1	pleckstrin homology domain containing, family D (with coiled-coil domains) member 1	ENSMUSG00000066438	31624	1,30
Lamc3	laminin gamma 3	ENSMUSG00000026840	59249	1,30
		ENSMUSG00000026468	44085	1,30
Fsbp	fibrinogen silencer binding protein	ENSMUSG00000094595	7927	1,30
Arhgef37	Rho guanine nucleotide exchange factor (GEF) 37	ENSMUSG00000045094	42743	1,30
Pmch	pro-melanin-concentrating hormone	ENSMUSG00000035383	1304	1,30
		ENSMUSG00000016239	33989	1,30
		ENSMUSG00000094157	15185	1,30
Adssl1	adenylosuccinate synthetase like 1	ENSMUSG00000011148	21310	1,30
Tg	thyroglobulin	ENSMUSG00000053469	179969	1,30
Tcam1	testicular cell adhesion molecule 1	ENSMUSG00000020712	12074	1,30
Gm10408	predicted gene 10408	ENSMUSG00000072739	9909	1,30
Ccr5	chemokine (C-C motif) receptor 5	ENSMUSG00000079227	26157	1,30
		ENSMUSG00000034009	96270	1,30
Shisa9	shisa homolog 9 (Xenopus laevis)	ENSMUSG00000022494	286790	1,30
Gls2	glutaminase 2 (liver, mitochondrial)	ENSMUSG00000044005	15548	1,30
Ifi205	interferon activated gene 205	ENSMUSG00000054203	19758	1,30
Hgd	homogentisate 1, 2-dioxygenase	ENSMUSG00000022821	51868	1,30
Kcns1	K+ voltage-gated channel, subfamily S, 1	ENSMUSG00000040164	7495	1,30
Pcp2	Purkinje cell protein 2 (L7)	ENSMUSG00000004630	2175	1,30
Sfta2	surfactant associated 2	ENSMUSG00000090509	49023	1,30
Cstad	CSA-conditional, T cell activation-dependent protein	ENSMUSG00000047363	13904	1,30
		ENSMUSG00000022296	16264	1,30
Gfra3	glial cell line derived neurotrophic factor family receptor alpha 3	ENSMUSG00000024366	30485	1,30
Serpina1a	serine (or cysteine) peptidase inhibitor, clade A, member 1A	ENSMUSG00000066366	9974	1,30
Igsf9b	immunoglobulin superfamily, member 9B	ENSMUSG00000034275	35536	1,30
Zfp804a	zinc finger protein 804A	ENSMUSG00000070866	206658	1,30
1700093K21Rik	RIKEN cDNA 1700093K21 gene	ENSMUSG00000020286	4953	1,30
Pcdhb3	protocadherin beta 3	ENSMUSG00000045498	3787	1,30
		ENSMUSG00000072762	15897	1,30
Gabrr1	gamma-aminobutyric acid (GABA) C receptor, subunit rho 1	ENSMUSG00000028280	31068	1,30
Gm10647	predicted gene 10647	ENSMUSG00000074232	2036	1,30
Kcng3	potassium voltage-gated channel, subfamily G, member 3	ENSMUSG00000045053	45939	1,30
Duox1	dual oxidase 1	ENSMUSG00000033268	32301	1,30

Medag	mesenteric estrogen dependent adipogenesis	ENSMUSG00000029659	63248	1,30
Hipk4	homeodomain interacting protein kinase 4	ENSMUSG00000040424	7909	1,30
		ENSMUSG00000015957	20084	1,30
Pnoc	prepronociceptin	ENSMUSG00000045731	24800	1,30
Gpr35	G protein-coupled receptor 35	ENSMUSG00000026271	13273	1,30
Ankrd61	ankyrin repeat domain 61	ENSMUSG00000029607	6945	1,30
Chit1	chitinase 1 (chitotriosidase)	ENSMUSG00000026450	40299	1,30
Htr6	5-hydroxytryptamine (serotonin) receptor 6	ENSMUSG00000028747	14463	1,30
		ENSMUSG00000079049	16329	1,30
Plk3	polo-like kinase 3	ENSMUSG00000028680	5309	1,30
Ccdc116	coiled-coil domain containing 116	ENSMUSG00000022768	8166	1,30
Gm10258	predicted gene 10258	ENSMUSG00000069074	2123	1,30
		ENSMUSG00000056031	23045	1,30
Rhox3c	reproductive homeobox 3C	ENSMUSG00000079636	15380	1,30
		ENSMUSG00000009734	260941	1,30
Bcl2a1d	B cell leukemia/lymphoma 2 related protein A1d	ENSMUSG00000096581	8563	1,30
Emilin3	elastin microfibril interfacer 3	ENSMUSG00000050700	5892	1,30
Mroh7	maestro heat-like repeat family member 7	ENSMUSG00000047502	50509	1,30
		ENSMUSG00000016498	60239	1,30
		ENSMUSG00000030137	16121	1,30
I830012O16Rik	RIKEN cDNA I830012O16 gene	ENSMUSG00000062488	5432	1,30
Grm2	glutamate receptor, metabotropic 2	ENSMUSG00000023192	11573	1,30
		ENSMUSG00000081362	41463	1,30
Ccl7	chemokine (C-C motif) ligand 7	ENSMUSG00000035373	1814	1,30
7530416G11Rik	RIKEN cDNA 7530416G11 gene	ENSMUSG00000096607	10492	1,30
Spint3	serine peptidase inhibitor, Kunitz type, 3	ENSMUSG00000074596	3765	1,30
Mgp	matrix Gla protein	ENSMUSG00000030218	3370	1,23
Acsbg1	acyl-CoA synthetase bubblegum family member 1	ENSMUSG00000032281	56994	1,23
Col5a1	collagen, type V, alpha 1	ENSMUSG00000026837	153090	1,23
Adamts5	a disintegrin-like and metallopeptidase (reprolysin type) with thrombospondin type 1 motif, 5 (aggrecanase-2)	ENSMUSG00000022894	42956	1,23
Krt14	keratin 14	ENSMUSG00000045545	4387	1,23
Tfap2a	transcription factor AP-2, alpha	ENSMUSG00000021359	18149	1,23
Kcng1	potassium voltage-gated channel, subfamily G, member 1	ENSMUSG00000074575	21620	1,23
Aif1l	allograft inflammatory factor 1-like	ENSMUSG00000001864	23183	1,23
Ism1	isthmin 1 homolog (zebrafish)	ENSMUSG00000074766	80404	1,23
Mmrn1	multimerin 1	ENSMUSG00000054641	64403	1,23
		ENSMUSG00000017446	21761	1,23
Dand5	DAN domain family, member 5	ENSMUSG00000053226	7419	1,23
Trabd2b	TraB domain containing 2B	ENSMUSG00000070867	208375	1,23
Hapln1	hyaluronan and proteoglycan link protein 1	ENSMUSG00000021613	71197	1,23
Caln1	calneuron 1	ENSMUSG00000060371	471170	1,23
Mip	major intrinsic protein of eye lens fiber	ENSMUSG00000025389	5975	1,23
Bdnf	brain derived neurotrophic factor	ENSMUSG00000048482	52308	1,23

1700048O20Rik	RIKEN cDNA 1700048O20 gene	ENSMUSG00000043773	8880	1,23
Scarf1	scavenger receptor class F, member 1	ENSMUSG00000038188	13043	1,23
Grm4	glutamate receptor, metabotropic 4	ENSMUSG00000063239	90955	1,23
Slc22a3	solute carrier family 22 (organic cation transporter), member 3	ENSMUSG00000023828	87733	1,23
Sdsl	serine dehydratase-like	ENSMUSG00000029596	14625	1,23
2610318N02Rik	RIKEN cDNA 2610318N02 gene	ENSMUSG00000049916	11708	1,23
Batf	basic leucine zipper transcription factor, ATF-like	ENSMUSG00000034266	22419	1,23
		ENSMUSG00000042826	5790	1,23
Zfp300	zinc finger protein 300	ENSMUSG00000031079	10354	1,23
Gm10684	predicted gene 10684	ENSMUSG00000074385	28431	1,23
Usp17lc	ubiquitin specific peptidase 17-like C	ENSMUSG00000058976	4809	1,23
Rnaseh1	ribonuclease H1	ENSMUSG00000020630	9990	1,23
H2-T23	histocompatibility 2, T region locus 23	ENSMUSG00000067212	3083	1,23
Ptchd4	patched domain containing 4	ENSMUSG00000042256	191795	1,23
Upk3bl	uroplakin 3B-like	ENSMUSG00000006143	9835	1,23
Fndc4	fibronectin type III domain containing 4	ENSMUSG00000038552	3623	1,23
Cpn1	carboxypeptidase N, polypeptide 1	ENSMUSG00000025196	30250	1,23
Gm10654	predicted gene 10654	ENSMUSG00000074252	1633	1,23
Nutm1	NUT midline carcinoma, family member 1	ENSMUSG00000041358	11344	1,23
Zscan25	zinc finger and SCAN domain containing 25	ENSMUSG00000070420	8127	1,23
Arntl2	aryl hydrocarbon receptor nuclear translocator-like 2	ENSMUSG00000040187	37475	1,23
Zfp382	zinc finger protein 382	ENSMUSG00000074220	12724	1,23
Prss35	protease, serine, 35	ENSMUSG00000033491	14640	1,23
Apol7e	apolipoprotein L 7e	ENSMUSG00000071716	20400	1,23
Agap2	ArfGAP with GTPase domain, ankyrin repeat and PH domain 2	ENSMUSG00000025422	14263	1,23
Rimbp3	RIMS binding protein 3	ENSMUSG00000071636	5837	1,23
Slc19a2	solute carrier family 19 (thiamine transporter), member 2	ENSMUSG00000040918	16340	1,23
Esrrb	estrogen related receptor, beta	ENSMUSG00000021255	160512	1,23
Amigo2	adhesion molecule with Ig like domain 2	ENSMUSG00000048218	3214	1,23
Ogdhl	oxoglutarate dehydrogenase-like	ENSMUSG00000021913	25802	1,23
		ENSMUSG00000078502	40180	1,23
		ENSMUSG00000093536	33379	1,23
Glb1l3	galactosidase, beta 1 like 3	ENSMUSG00000031966	42871	1,23
Lrrtm3	leucine rich repeat transmembrane neuronal 3	ENSMUSG00000042846	161806	1,23
Sp140	Sp140 nuclear body protein	ENSMUSG00000070031	44660	1,23
Zfp820	zinc finger protein 820	ENSMUSG00000069743	28884	1,23
Mgst2	microsomal glutathione S-transferase 2	ENSMUSG00000074604	22316	1,23
Themis3	thymocyte selection associated family member 3	ENSMUSG00000024105	39370	1,23
Clec4a3	C-type lectin domain family 4, member a3	ENSMUSG00000043832	17361	1,23
Aknad1	AKNA domain containing 1	ENSMUSG00000049565	42639	1,23

Gm9881	predicted gene 9881	ENSMUSG00000052471	3205	1,23
Myo18b	myosin XVIIIb	ENSMUSG00000072720	207487	1,23
Nxnl1	nucleoredoxin-like 1	ENSMUSG00000034829	6094	1,23
Tnni1	troponin I, skeletal, slow 1	ENSMUSG00000026418	27925	1,23
Cel	carboxyl ester lipase	ENSMUSG00000026818	7609	1,23
Skint4	selection and upkeep of intraepithelial T cells 4	ENSMUSG00000055960	96061	1,23
Ntf5	neurotrophin 5	ENSMUSG00000074121	3484	1,23
Cyp3a44	cytochrome P450, family 3, subfamily a, polypeptide 44	ENSMUSG00000054417	31892	1,23
Gm10416	predicted pseudogene 10416	ENSMUSG00000072761	3434	1,23
Rrh	retinal pigment epithelium derived rhodopsin homolog	ENSMUSG00000028012	18098	1,23
4930402F06Rik	RIKEN cDNA 4930402F06 gene	ENSMUSG00000079421	21614	1,23
Vmn2r65	vomeronasal 2, receptor 65	ENSMUSG00000066372	23841	1,23
Awat2	acyl-CoA wax alcohol acyltransferase 2	ENSMUSG00000031220	40497	1,23
Tnfsf18	tumor necrosis factor (ligand) superfamily, member 18	ENSMUSG00000066755	10636	1,23
Hsf2bp	heat shock transcription factor 2 binding protein	ENSMUSG00000002076	89740	1,23
Gm9733	predicted gene 9733	ENSMUSG00000078783	35752	1,23
C030017K20Rik	RIKEN cDNA C030017K20 gene	ENSMUSG00000078490	14961	1,23
Scn4a	sodium channel, voltage-gated, type IV, alpha	ENSMUSG00000001027	34697	1,23
Smok3a	sperm motility kinase 3A	ENSMUSG00000075599	13390	1,23
Pmaip1	phorbol-12-myristate-13-acetate-induced protein 1	ENSMUSG00000024521	6947	1,23
Gulo	gulonolactone (L-) oxidase	ENSMUSG00000034450	22424	1,23
C8a	complement component 8, alpha polypeptide	ENSMUSG00000035031	60720	1,23
		ENSMUSG00000079030	6697	1,23
Mettl7a3	methyltransferase like 7A3	ENSMUSG00000058057	5413	1,23
		ENSMUSG00000096808	1500	1,23
Pla2g12b	phospholipase A2, group XIIB	ENSMUSG00000009646	18317	1,23
Mettl21c	methyltransferase like 21C	ENSMUSG00000047343	10627	1,23
		ENSMUSG00000071192	2676	1,23
		ENSMUSG00000059645	6618	1,23
Nr2e3	nuclear receptor subfamily 2, group E, member 3	ENSMUSG00000032292	17889	1,23
2410124H12Rik	RIKEN cDNA 2410124H12 gene	ENSMUSG00000051297	18623	1,23
Slc38a8	solute carrier family 38, member 8	ENSMUSG00000034224	22097	1,23
Il2ra	interleukin 2 receptor, alpha chain	ENSMUSG00000026770	50400	1,23
Olfr18	olfactory receptor 18	ENSMUSG00000066896	22203	1,23
Gfra2	glial cell line derived neurotrophic factor family receptor alpha 2	ENSMUSG00000022103	89709	1,16
Tfap2b	transcription factor AP-2 beta	ENSMUSG00000025927	29821	1,16
Rgr	retinal G protein coupled receptor	ENSMUSG00000021804	11475	1,16
Msx1	msh homeobox 1	ENSMUSG00000048450	4099	1,16
Slc43a1	solute carrier family 43, member 1	ENSMUSG00000027075	24745	1,16
Pcsk1n	proprotein convertase subtilisin/kexin type 1 inhibitor	ENSMUSG00000039278	4589	1,16
Gpr162	G protein-coupled receptor 162	ENSMUSG00000038390	5474	1,16

Adamts10	a disintegrin-like and metallopeptidase (reprolysin type) with thrombospondin type 1 motif, 10	ENSMUSG00000024299	29579	1,16
Prkcq	protein kinase C, theta	ENSMUSG00000026778	129114	1,16
Tbx2	T-box 2	ENSMUSG00000000093	9398	1,16
Srd5a2	steroid 5 alpha-reductase 2	ENSMUSG00000038541	30211	1,16
4930562F07Rik	RIKEN cDNA 4930562F07 gene	ENSMUSG00000026729	3251	1,16
Nrsn1	neurensin 1	ENSMUSG00000048978	18463	1,16
Cmtm3	CKLF-like MARVEL transmembrane domain containing 3	ENSMUSG00000031875	7079	1,16
Art4	ADP-ribosyltransferase 4	ENSMUSG00000030217	9283	1,16
Bai1	brain-specific angiogenesis inhibitor 1	ENSMUSG00000034730	73248	1,16
Psg17	pregnancy specific glycoprotein 17	ENSMUSG00000004540	7671	1,16
Rab37	RAB37, member of RAS oncogene family	ENSMUSG00000020732	70806	1,16
Prss53	protease, serine, 53	ENSMUSG00000044139	5130	1,16
Alkbh2	alkB, alkylation repair homolog 2 (E. coli)	ENSMUSG00000044339	4293	1,16
Cyp2j12	cytochrome P450, family 2, subfamily j, polypeptide 12	ENSMUSG00000081225	41835	1,16
Lrr1	leucine rich repeat protein 1	ENSMUSG00000034883	10197	1,16
Thrsp	thyroid hormone responsive	ENSMUSG00000035686	4793	1,16
Card9	caspase recruitment domain family, member 9	ENSMUSG00000026928	8743	1,16
Zfp963	zinc finger protein 963	ENSMUSG00000092260	8332	1,16
Ccdc88b	coiled-coil domain containing 88B	ENSMUSG00000047810	13589	1,16
Lmx1a	LIM homeobox transcription factor 1 alpha	ENSMUSG00000026686	159503	1,16
Gm3500	predicted gene 3500	ENSMUSG00000096003	23256	1,16
		ENSMUSG00000091159	7773	1,16
Nat2	N-acetyltransferase 2 (arylamine N-acetyltransferase)	ENSMUSG00000051147	7714	1,16
Frs3	fibroblast growth factor receptor substrate 3	ENSMUSG00000023266	15257	1,16
		ENSMUSG00000090707	22465	1,16
Gm10010	predicted gene 10010	ENSMUSG00000056771	12778	1,16
Htr3a	5-hydroxytryptamine (serotonin) receptor 3A	ENSMUSG00000032269	11886	1,16
Nat8l	N-acetyltransferase 8-like	ENSMUSG00000048142	9933	1,16
Neurl2	neuralized-like 2 (Drosophila)	ENSMUSG00000039873	2725	1,16
Glra2	glycine receptor, alpha 2 subunit	ENSMUSG00000018589	198377	1,16
LOC100862024	uncharacterized LOC100862024	ENSMUSG00000002111	33367	1,16
		ENSMUSG00000063543	387	1,16
		ENSMUSG00000040724	5313	1,16
Clec4a2	C-type lectin domain family 4, member a2	ENSMUSG00000030148	37572	1,16
		ENSMUSG00000090691	21461	1,16
1700018C11Rik	RIKEN cDNA 1700018C11 gene	ENSMUSG00000058935	15340	1,16
Fam209	family with sequence similarity 209	ENSMUSG00000027505	1812	1,16
Chad	chondroadherin	ENSMUSG00000039084	4081	1,16
Nccrp1	non-specific cytotoxic cell receptor protein 1 homolog (zebrafish)	ENSMUSG00000047586	3659	1,16
Ano5	anoctamin 5	ENSMUSG00000055489	87681	1,16

Kcne2	potassium voltage-gated channel, Isk-related subfamily, gene 2	ENSMUSG00000039672	5741	1,16
		ENSMUSG00000078779	12232	1,16
Kcnrg	potassium channel regulator	ENSMUSG00000046168	4435	1,16
Prdx6b	peroxiredoxin 6B	ENSMUSG00000050114	2889	1,16
Mill2	MHC I like leukocyte 2	ENSMUSG00000040987	18689	1,16
Doc2g	double C2, gamma	ENSMUSG00000024871	3685	1,16
Abcg3	ATP-binding cassette, sub-family G (WHITE), member 3	ENSMUSG00000029299	47662	1,16
1700019A02Rik	RIKEN cDNA 1700019A02 gene	ENSMUSG00000060715	29041	1,16
		ENSMUSG00000096626	27859	1,16
Il1f5	interleukin 1 family, member 5 (delta)	ENSMUSG00000026983	6473	1,16
Tsks	testis-specific serine kinase substrate	ENSMUSG00000059891	14814	1,16
Gm9982	predicted gene 9982	ENSMUSG00000055831	1193	1,16
Ahrr	aryl-hydrocarbon receptor repressor	ENSMUSG00000021575	81214	1,16
Lrrc31	leucine rich repeat containing 31	ENSMUSG00000074653	20786	1,16
Gm13769	predicted gene 13769	ENSMUSG00000089859	2987	1,16
		ENSMUSG00000028417	9008	1,16
Gm1110	predicted gene 1110	ENSMUSG00000079644	43515	1,16
Irf4	interferon regulatory factor 4	ENSMUSG00000021356	17702	1,16
Aqp9	aquaporin 9	ENSMUSG00000032204	58024	1,16
Abcb1b	ATP-binding cassette, sub-family B (MDR/TAP), member 1B	ENSMUSG00000028970	68168	1,16
Serpinb6c	serine (or cysteine) peptidase inhibitor, clade B, member 6c	ENSMUSG00000052180	25893	1,16
Ceacam5	carcinoembryonic antigen-related cell adhesion molecule 5	ENSMUSG00000008789	47870	1,16
		ENSMUSG00000053216	11182	1,16
Fndc9	fibronectin type III domain containing 9	ENSMUSG00000048721	4342	1,16
		ENSMUSG00000055385	17788	1,16
		ENSMUSG00000059639	33739	1,16
Lama1	laminin, alpha 1	ENSMUSG00000032796	125381	1,16
G430095P16Rik	RIKEN cDNA G430095P16 gene	ENSMUSG00000074203	3838	1,16
Cib4	calcium and integrin binding family member 4	ENSMUSG00000053194	60254	1,16
Il23r	interleukin 23 receptor	ENSMUSG00000049093	68924	1,16
Tbx6	T-box 6	ENSMUSG00000030699	4078	1,16
Fcer2a	Fc receptor, IgE, low affinity II, alpha polypeptide	ENSMUSG00000005540	12438	1,16
Tdrd12	tudor domain containing 12	ENSMUSG00000030491	44134	1,16
Nxpe5	neurexophilin and PC-esterase domain family, member 5	ENSMUSG00000047592	27466	1,16
Pyroxd2	pyridine nucleotide-disulphide oxidoreductase domain 2	ENSMUSG00000060224	26918	1,16
E230019M04Rik	RIKEN cDNA E230019M04 gene	ENSMUSG00000042433	43919	1,16
Onecut3	one cut domain, family member 3	ENSMUSG00000045518	22442	1,16
Npff	neuropeptide FF-amide peptide precursor	ENSMUSG00000023052	779	1,16
Ankar	ankyrin and armadillo repeat containing	ENSMUSG00000039342	57585	1,16
Tnf	tumor necrosis factor	ENSMUSG00000024401	2627	1,16
1700128F08Rik	RIKEN cDNA 1700128F08 gene	ENSMUSG00000031998	20099	1,16

Gm711	predicted gene 711	ENSMUSG00000049897	19450	1,16
Galp	galanin-like peptide	ENSMUSG00000034660	19048	1,16
Yipf7	Yip1 domain family, member 7	ENSMUSG00000029158	25973	1,16
Tsx	testis specific X-linked gene	ENSMUSG00000031329	10114	1,16
Mtnr1a	melatonin receptor 1A	ENSMUSG00000054764	19220	1,16
C1qtnf9	C1q and tumor necrosis factor related protein 9	ENSMUSG00000071347	12736	1,16
Uts2	urotensin 2	ENSMUSG00000028963	4714	1,16
		ENSMUSG00000020279	12739	1,16
1700024G13Rik	RIKEN cDNA 1700024G13 gene	ENSMUSG00000072473	11872	1,16
Vsig4	V-set and immunoglobulin domain containing 4	ENSMUSG00000044206	46236	1,16
Pde6c	phosphodiesterase 6C, cGMP specific, cone, alpha prime	ENSMUSG00000024992	51167	1,16
Lman1l	lectin, mannose-binding 1 like	ENSMUSG00000056271	13690	1,16
Cd93	CD93 antigen	ENSMUSG00000027435	6924	1,09
Aebp1	AE binding protein 1	ENSMUSG00000020473	10142	1,09
Tril	TLR4 interactor with leucine-rich repeats	ENSMUSG00000043496	5355	1,09
Emp3	epithelial membrane protein 3	ENSMUSG00000040212	3404	1,09
Aspn	asporin	ENSMUSG00000021388	23115	1,09
Slc9b2	solute carrier family 9, subfamily B (NHA2, cation proton antiporter 2), member 2	ENSMUSG00000037994	35068	1,09
Thnsl2	threonine synthase-like 2 (bacterial)	ENSMUSG00000054474	16181	1,09
Nos3	nitric oxide synthase 3, endothelial cell	ENSMUSG00000028978	19665	1,09
Ly6c1	lymphocyte antigen 6 complex, locus C1	ENSMUSG00000079018	3821	1,09
Pold4	polymerase (DNA-directed), delta 4	ENSMUSG00000024854	1733	1,09
B630019K06Rik	RIKEN cDNA B630019K06 gene	ENSMUSG00000052364	2582	1,09
Slc7a10	solute carrier family 7 (cationic amino acid transporter, y+ system), member 10	ENSMUSG00000030495	18514	1,09
D3Bwg0562e	DNA segment, Chr 3, Brigham & Women's Genetics 0562 expressed	ENSMUSG00000044667	41732	1,09
		ENSMUSG00000095789	1829	1,09
Mamdc2	MAM domain containing 2	ENSMUSG00000033207	145714	1,09
Gm10657	predicted gene 10657	ENSMUSG00000074273	1031	1,09
Jph2	junctophilin 2	ENSMUSG00000017817	61752	1,09
Jam2	junction adhesion molecule 2	ENSMUSG00000053062	51806	1,09
Tmem150a	transmembrane protein 150A	ENSMUSG00000055912	4316	1,09
Zfp599	zinc finger protein 599	ENSMUSG00000062794	12466	1,09
		ENSMUSG00000079323	11950	1,09
Itga10	integrin, alpha 10	ENSMUSG00000090210	18936	1,09
Tnfsf10	tumor necrosis factor (ligand) superfamily, member 10	ENSMUSG00000039304	25400	1,09
Slc2a5	solute carrier family 2 (facilitated glucose transporter), member 5	ENSMUSG00000028976	24887	1,09
Begain	brain-enriched guanylate kinase-associated	ENSMUSG00000040867	36031	1,09
Fcrls	Fc receptor-like S, scavenger receptor	ENSMUSG00000015852	12981	1,09

Trem2	triggering receptor expressed on myeloid cells 2	ENSMUSG00000023992	7747	1,09
		ENSMUSG00000045589	33219	1,09
Cacna1s	calcium channel, voltage-dependent, L type, alpha 1S subunit	ENSMUSG00000026407	67073	1,09
C2cd4c	C2 calcium-dependent domain containing 4C	ENSMUSG00000045912	7169	1,09
Krt1	keratin 1	ENSMUSG00000046834	5359	1,09
Mei1	meiosis defective 1	ENSMUSG00000068117	56819	1,09
		ENSMUSG00000056089	653	1,09
Il2	interleukin 2	ENSMUSG00000027720	5437	1,09
Nap1l2	nucleosome assembly protein 1-like 2	ENSMUSG00000082229	2465	1,09
Creb3l3	cAMP responsive element binding protein 3-like 3	ENSMUSG00000035041	14551	1,09
Egfl6	EGF-like-domain, multiple 6	ENSMUSG00000000402	62714	1,09
Col5a3	collagen, type V, alpha 3	ENSMUSG00000004098	45018	1,09
1700055D18Rik	RIKEN cDNA 1700055D18 gene	ENSMUSG00000070997	1570	1,09
Rdh12	retinol dehydrogenase 12	ENSMUSG00000021123	13752	1,09
Dhrs2	dehydrogenase/reductase member 2	ENSMUSG00000022209	19429	1,09
		ENSMUSG00000078503	28430	1,09
Ntng2	netrin G2	ENSMUSG00000035513	58212	1,09
Cnih3	cornichon homolog 3 (Drosophila)	ENSMUSG00000026514	108014	1,09
Myzap	myocardial zonula adherens protein	ENSMUSG00000041361	88014	1,09
		ENSMUSG00000027324	4980	1,09
		ENSMUSG00000096904	6656	1,09
Gm10244	predicted gene 10244	ENSMUSG00000068601	1408	1,09
Pla2g2c	phospholipase A2, group IIC	ENSMUSG00000028750	21341	1,09
Tnip3	TNFAIP3 interacting protein 3	ENSMUSG00000044162	43643	1,09
Rasgrf1	RAS protein-specific guanine nucleotide-releasing factor 1	ENSMUSG00000032356	117202	1,09
Itgb2	integrin beta 2	ENSMUSG00000000290	35457	1,09
Bambi	BMP and activin membrane-bound inhibitor	ENSMUSG00000024232	8448	1,09
Pth2r	parathyroid hormone 2 receptor	ENSMUSG00000025946	107189	1,09
Col6a6	collagen, type VI, alpha 6	ENSMUSG00000043719	138669	1,09
		ENSMUSG00000078861	10782	1,09
Ankrd35	ankyrin repeat domain 35	ENSMUSG00000038354	20879	1,09
Tnfsf13	tumor necrosis factor (ligand) superfamily, member 13	ENSMUSG00000089669	2939	1,09
Plcd1	phospholipase C, delta 1	ENSMUSG00000010660	21970	1,09
BC021891	cDNA sequence BC021891	ENSMUSG00000031853	36990	1,09
Smim6	small integral membrane protein 6	ENSMUSG00000075420	1904	1,09
Galntl5	UDP-N-acetyl-alpha-D-galactosamine:polypeptide N-acetylgalactosaminyltransferase-like 5	ENSMUSG00000028938	38838	1,09
Srms	src-related kinase lacking C-terminal regulatory tyrosine and N-terminal myristylation sites	ENSMUSG00000027579	7624	1,09
Card11	caspase recruitment domain family, member 11	ENSMUSG00000036526	127598	1,09
3110070M22Rik	RIKEN cDNA 3110070M22 gene	ENSMUSG00000074635	1123	1,09

Pnpla1	patatin-like phospholipase domain containing 1	ENSMUSG00000043286	31898	1,09
Xkr8	X Kell blood group precursor related family member 8 homolog	ENSMUSG00000037752	7650	1,09
Ttc36	tetratricopeptide repeat domain 36	ENSMUSG00000039438	3552	1,09
		ENSMUSG00000041287	1414	1,09
Traf1	TNF receptor-associated factor 1	ENSMUSG00000026875	20023	1,09
		ENSMUSG00000078872	13028	1,09
Pabpc5	poly(A) binding protein, cytoplasmic 5	ENSMUSG00000034732	2970	1,09
Il21r	interleukin 21 receptor	ENSMUSG00000030745	30142	1,09
4931429P17Rik	RIKEN cDNA 4931429P17 gene	ENSMUSG00000047324	58279	1,09
Adamts14	a disintegrin-like and metallopeptidase (reprolysin type) with thrombospondin type 1 motif, 14	ENSMUSG00000059901	76327	1,09
Gabrq	gamma-aminobutyric acid (GABA) A receptor, subunit theta	ENSMUSG00000031344	17425	1,09
Slc25a43	solute carrier family 25, member 43	ENSMUSG00000037636	33544	1,09
Obscn	obscurin, cytoskeletal calmodulin and titin-interacting RhoGEF	ENSMUSG00000061462	142147	1,09
		ENSMUSG00000094590	45751	1,09
Wnt3	wingless-related MMTV integration site 3	ENSMUSG00000000125	43808	1,09
Abra	actin-binding Rho activating protein	ENSMUSG00000042895	4428	1,09
Cldn22	claudin 22	ENSMUSG00000038064	994	1,09
Tbx21	T-box 21	ENSMUSG00000001444	17261	1,09
Cyp2c39	cytochrome P450, family 2, subfamily c, polypeptide 39	ENSMUSG00000025003	57668	1,09
Klra2	killer cell lectin-like receptor, subfamily A, member 2	ENSMUSG00000030187	28140	1,09
		ENSMUSG00000095852	42595	1,09
		ENSMUSG00000051228	23204	1,09
Fhl5	four and a half LIM domains 5	ENSMUSG00000028259	42969	1,09
Syce3	synaptonemal complex central element protein 3	ENSMUSG00000078938	20338	1,09
Ly6g5b	lymphocyte antigen 6 complex, locus G5B	ENSMUSG00000043807	1481	1,09
		ENSMUSG00000069208	2427	1,09
Slc26a5	solute carrier family 26, member 5	ENSMUSG00000029015	54950	1,09
Prl3a1	prolactin family 3, subfamily a, member 1	ENSMUSG00000038883	17225	1,09
Pdilt	protein disulfide isomerase-like, testis expressed	ENSMUSG00000030968	36896	1,09
Spem1	sperm maturation 1	ENSMUSG00000041165	1305	1,09
Mylpf	myosin light chain, phosphorylatable, fast skeletal muscle	ENSMUSG00000030672	2691	1,09
Abcc8	ATP-binding cassette, sub-family C (CFTR/MRP), member 8	ENSMUSG00000040136	75511	1,09
		ENSMUSG00000078960	1123	1,09
Stac3	SH3 and cysteine rich domain 3	ENSMUSG00000040287	7138	1,09
B230216G23Rik	RIKEN cDNA B230216G23 gene	ENSMUSG00000071112	6290	1,09
Loxhd1	lipoxygenase homology domains 1	ENSMUSG00000032818	160384	1,09
4921511M17Rik	RIKEN cDNA 4921511M17 gene	ENSMUSG00000094577	11048	1,09

Tm4sf20	transmembrane 4 L six family member 20	ENSMUSG00000026149	11813	1,09
		ENSMUSG00000040836	21068	1,09
		ENSMUSG00000074146	25809	1,09
4930510E17Rik	RIKEN cDNA 4930510E17 gene	ENSMUSG00000032048	16371	1,09
Prdm14	PR domain containing 14	ENSMUSG00000042414	13707	1,09
Tbata	thymus, brain and testes associated	ENSMUSG00000020096	16888	1,09
Hrh2	histamine receptor H2	ENSMUSG00000034987	30304	1,09
Ppp1r32	protein phosphatase 1, regulatory subunit 32	ENSMUSG00000035179	8641	1,09
Gm3115	predicted gene 3115	ENSMUSG00000091022	15187	1,09
Hhipl2	hedgehog interacting protein-like 2	ENSMUSG00000053461	18551	1,09
Cyp2c67	cytochrome P450, family 2, subfamily c, polypeptide 67	ENSMUSG00000062624	40210	1,09
Gast	gastrin	ENSMUSG00000017165	2590	1,09
		ENSMUSG00000028386	9931	1,09
Olfr1045	olfactory receptor 1045	ENSMUSG00000075198	948	1,09
Olfr1037	olfactory receptor 1037	ENSMUSG00000075205	1125	1,09
Olfr293	olfactory receptor 293	ENSMUSG00000063394	1011	1,09
Olfr543	olfactory receptor 543	ENSMUSG00000044814	1130	1,09
Olfr52	olfactory receptor 52	ENSMUSG00000075199	960	1,09
Olfr1357	olfactory receptor 1357	ENSMUSG00000071185	6406	1,09
		ENSMUSG00000092413	17325	1,09
Olfr875	olfactory receptor 875	ENSMUSG00000058628	1004	1,09
Cyp3a13	cytochrome P450, family 3, subfamily a, polypeptide 13	ENSMUSG00000029727	28688	1,01
Fam211b	family with sequence similarity 211, member B	ENSMUSG00000046807	10206	1,01
Kank4	KN motif and ankyrin repeat domains 4	ENSMUSG00000035407	62640	1,01
Col10a1	collagen, type X, alpha 1	ENSMUSG00000039462	7105	1,01
		ENSMUSG00000059830	480	1,01
Rapgef3	Rap guanine nucleotide exchange factor (GEF) 3	ENSMUSG00000022469	23203	1,01
Rasl11b	RAS-like, family 11, member B	ENSMUSG00000049907	4183	1,01
Bbs12	Bardet-Biedl syndrome 12 (human)	ENSMUSG00000051444	8900	1,01
		ENSMUSG00000045775	11925	1,01
Wee2	WEE1 homolog 2 (S. pombe)	ENSMUSG00000037159	23951	1,01
Prom2	prominin 2	ENSMUSG00000027376	14995	1,01
Cthrc1	collagen triple helix repeat containing 1	ENSMUSG00000054196	10190	1,01
Zscan18	zinc finger and SCAN domain containing 18	ENSMUSG00000070822	7567	1,01
Aurkb	aurora kinase B	ENSMUSG00000020897	6018	1,01
Gria1	glutamate receptor, ionotropic, AMPA1 (alpha 1)	ENSMUSG00000020524	318858	1,01
Greb1	gene regulated by estrogen in breast cancer protein	ENSMUSG00000036523	130272	1,01
Fkbp7	FK506 binding protein 7	ENSMUSG00000002732	10073	1,01
Gm10273	predicted pseudogene 10273	ENSMUSG00000069622	1126	1,01
Tmem121	transmembrane protein 121	ENSMUSG00000049036	3618	1,01
Gm5093	predicted gene 5093	ENSMUSG00000091742	522	1,01
Tagln3	transgelin 3	ENSMUSG00000022658	13379	1,01
Ifit2	interferon-induced protein with tetratricopeptide repeats 2	ENSMUSG00000045932	25726	1,01

Chst3	carbohydrate (chondroitin 6/keratan) sulfotransferase 3	ENSMUSG00000057337	37729	1,01
		ENSMUSG00000020303	12768	1,01
Mb21d1	Mab-21 domain containing 1	ENSMUSG00000032344	12712	1,01
		ENSMUSG00000084897	8839	1,01
		ENSMUSG00000024338	3734	1,01
Snhg11	small nucleolar RNA host gene 11	ENSMUSG00000044349	10508	1,01
Zfp759	zinc finger protein 759	ENSMUSG00000057396	13560	1,01
		ENSMUSG00000038135	7006	1,01
		ENSMUSG00000071181	1711	1,01
Slc26a3	solute carrier family 26, member 3	ENSMUSG00000001225	83047	1,01
Baiap3	BAI1-associated protein 3	ENSMUSG00000047507	13706	1,01
Scd4	stearoyl-coenzyme A desaturase 4	ENSMUSG00000050195	13652	1,01
Tmem156	transmembrane protein 156	ENSMUSG00000037913	28348	1,01
Ms4a1	membrane-spanning 4-domains, subfamily A, member 1	ENSMUSG00000024673	15549	1,01
Popdc2	popeye domain containing 2	ENSMUSG00000022803	16008	1,01
		ENSMUSG00000053706	3702	1,01
Zkscan7	zinc finger with KRAB and SCAN domains 7	ENSMUSG00000063488	7654	1,01
Cdh7	cadherin 7, type 2	ENSMUSG00000026312	157727	1,01
Tceal3	transcription elongation factor A (SII)-like 3	ENSMUSG00000044550	77537	1,01
Cfi	complement component factor i	ENSMUSG00000058952	38590	1,01
Rnase6	ribonuclease, RNase A family, 6	ENSMUSG00000021880	2055	1,01
		ENSMUSG00000073016	24673	1,01
Xlr4a	X-linked lymphocyte-regulated 4A	ENSMUSG00000079845	8134	1,01
Ctsw	cathepsin W	ENSMUSG00000024910	3259	1,01
Mroh8	maestro heat-like repeat family member 8	ENSMUSG00000074627	71000	1,01
		ENSMUSG00000026068	36344	1,01
Gm13547	predicted gene 13547	ENSMUSG00000075425	9687	1,01
Ccdc160	coiled-coil domain containing 160	ENSMUSG00000073207	8269	1,01
Mutyh	mutY homolog (E. coli)	ENSMUSG00000028687	11718	1,01
Slco1c1	solute carrier organic anion transporter family, member 1c1	ENSMUSG00000030235	45792	1,01
Cytip	cytohesin 1 interacting protein	ENSMUSG00000026832	66396	1,01
Trim50	tripartite motif-containing 50	ENSMUSG00000053388	14711	1,01
Phyhip	phytanoyl-CoA hydroxylase interacting protein	ENSMUSG00000003469	11357	1,01
		ENSMUSG00000078881	13506	1,01
Ky	kyphoscoliosis peptidase	ENSMUSG00000035606	40101	1,01
Gm9923	predicted pseudogene 9923	ENSMUSG00000053830	2624	1,01
		ENSMUSG00000035296	37432	1,01
		ENSMUSG00000094929	6674	1,01
1700012P22Rik	RIKEN cDNA 1700012P22 gene	ENSMUSG00000028589	20584	1,01
Gm2381	predicted gene 2381	ENSMUSG00000092225	50406	1,01
		ENSMUSG00000098164	11566	1,01
Bdkrb2	bradykinin receptor, beta 2	ENSMUSG00000021070	29900	1,01
Hsd17b2	hydroxysteroid (17-beta) dehydrogenase 2	ENSMUSG00000031844	57084	1,01
Adam18	a disintegrin and metallopeptidase domain 18	ENSMUSG00000031552	72510	1,01
Erv3	endogenous retroviral sequence 3	ENSMUSG00000037482	6070	1,01
Nuggc	nuclear GTPase, germinal center associated	ENSMUSG00000061356	49986	1,01

Angptl7	angiopoietin-like 7	ENSMUSG00000028989	5278	1,01
		ENSMUSG00000079051	10028	1,01
		ENSMUSG00000097905	5940	1,01
Scrg1	scrapie responsive gene 1	ENSMUSG00000031610	21663	1,01
E230025N22Rik	Riken cDNA E230025N22 gene	ENSMUSG00000044719	11003	1,01
D7Ertd443e	DNA segment, Chr 7, ERATO Doi 443, expressed	ENSMUSG00000030994	119400	1,01
Pnliprp1	pancreatic lipase related protein 1	ENSMUSG00000042179	15283	1,01
CN725425	cDNA sequence CN725425	ENSMUSG00000078932	29308	1,01
Nr1i2	nuclear receptor subfamily 1, group I, member 2	ENSMUSG00000022809	46502	1,01
		ENSMUSG00000091561	552	1,01
Gm11437	predicted gene 11437	ENSMUSG00000051452	19126	1,01
4932411N23Rik	RIKEN cDNA 4932411N23 gene	ENSMUSG00000058670	21543	1,01
		ENSMUSG00000063656	8895	1,01
Skint9	selection and upkeep of intraepithelial T cells 9	ENSMUSG00000049972	48017	1,01
Lamp5	lysosomal-associated membrane protein family, member 5	ENSMUSG00000027270	17679	1,01
Adam29	a disintegrin and metallopeptidase domain 29	ENSMUSG00000046258	36037	1,01
		ENSMUSG00000093867	71867	1,01
Apobec4	apolipoprotein B mRNA editing enzyme, catalytic polypeptide-like 4 (putative)	ENSMUSG00000055547	6994	1,01
		ENSMUSG00000098137	23770	1,01
Gpr113	G protein-coupled receptor 113	ENSMUSG00000067642	12292	1,01
Oosp1	oocyte secreted protein 1	ENSMUSG00000041857	23691	1,01
		ENSMUSG00000090891	7426	1,01
6030468B19Rik	RIKEN cDNA 6030468B19 gene	ENSMUSG00000025573	9642	1,01
Mybpc3	myosin binding protein C, cardiac	ENSMUSG00000002100	18373	1,01
Olfml1	olfactomedin-like 1	ENSMUSG00000051041	23649	1,01
Olfr77	olfactory receptor 77	ENSMUSG00000051118	5282	1,01
Aqp1	aquaporin 1	ENSMUSG00000004655	12124	0,94
Thbs2	thrombospondin 2	ENSMUSG00000023885	28763	0,94
G0s2	G0/G1 switch gene 2	ENSMUSG00000009633	1028	0,94
Tmem59l	transmembrane protein 59-like	ENSMUSG00000035964	3483	0,94
Kcnn4	potassium intermediate/small conductance calcium-activated channel, subfamily N, member 4	ENSMUSG00000054342	14947	0,94
Vdr	vitamin D receptor	ENSMUSG00000022479	56206	0,94
Smpdl3b	sphingomyelin phosphodiesterase, acid-like 3B	ENSMUSG00000028885	24287	0,94
Cdr1	cerebellar degeneration related antigen 1	ENSMUSG00000090546	2313	0,94
Nrn1	neuritin 1	ENSMUSG00000039114	8853	0,94
Gprc5b	G protein-coupled receptor, family C, group 5, member B	ENSMUSG00000008734	23172	0,94
Tgfbi	transforming growth factor, beta induced	ENSMUSG00000035493	29737	0,94
Derl3	Der1-like domain family, member 3	ENSMUSG00000009092	2544	0,94
Marc1	mitochondrial amidoxime reducing component 1	ENSMUSG00000026621	24538	0,94
Fgd3	FYVE, RhoGEF and PH domain containing 3	ENSMUSG00000037946	58758	0,94
Acot2	acyl-CoA thioesterase 2	ENSMUSG00000021226	6013	0,94

Sertm1	serine rich and transmembrane domain containing 1	ENSMUSG00000056306	18820	0,94
Slc45a3	solute carrier family 45, member 3	ENSMUSG00000026435	19997	0,94
		ENSMUSG00000092310	14192	0,94
Aoc2	amine oxidase, copper containing 2 (retina-specific)	ENSMUSG00000078651	4640	0,94
		ENSMUSG00000097678	599	0,94
Alox5ap	arachidonate 5-lipoxygenase activating protein	ENSMUSG00000060063	22988	0,94
Zfp455	zinc finger protein 455	ENSMUSG00000051037	14793	0,94
Slc35f2	solute carrier family 35, member F2	ENSMUSG00000042195	46627	0,94
Gypa	glycophorin A	ENSMUSG00000051839	16741	0,94
Rtn4r	reticulon 4 receptor	ENSMUSG00000043811	24767	0,94
Ifrd2	interferon-related developmental regulator 2	ENSMUSG00000010048	5321	0,94
Ces2g	carboxylesterase 2G	ENSMUSG00000031877	7820	0,94
Pdgfb	platelet derived growth factor, B polypeptide	ENSMUSG00000000489	18909	0,94
Artn	artemin	ENSMUSG00000028539	3602	0,94
Armcx6	armadillo repeat containing, X-linked 6	ENSMUSG00000050394	2957	0,94
Fam69b	family with sequence similarity 69, member B	ENSMUSG00000036186	8041	0,94
Hcn3	hyperpolarization-activated, cyclic nucleotide-gated K+ 3	ENSMUSG00000028051	14123	0,94
Gm10767	predicted gene 10767	ENSMUSG00000074826	4323	0,94
Gm14393	predicted gene 14393	ENSMUSG00000078905	6233	0,94
Myom3	myomesin family, member 3	ENSMUSG00000037139	55850	0,94
Amigo3	adhesion molecule with Ig like domain 3	ENSMUSG00000032593	2543	0,94
Hmox1	heme oxygenase (decycling) 1	ENSMUSG00000005413	7006	0,94
Pbld1	phenazine biosynthesis-like protein domain containing 1	ENSMUSG00000020072	16381	0,94
Fam57b	family with sequence similarity 57, member B	ENSMUSG00000058966	13335	0,94
Hist1h2bp	histone cluster 1, H2bp	ENSMUSG00000069308	1753	0,94
Hmcn2	hemicentin 2	ENSMUSG00000055632	146324	0,94
Folr1	folate receptor 1 (adult)	ENSMUSG00000001827	12458	0,94
Fxyd7	FXYD domain-containing ion transport regulator 7	ENSMUSG00000036578	8946	0,94
		ENSMUSG00000030600	8826	0,94
Tssk4	testis-specific serine kinase 4	ENSMUSG00000007591	2358	0,94
Gnb3	guanine nucleotide binding protein (G protein), beta 3	ENSMUSG00000023439	6036	0,94
Arsj	arylsulfatase J	ENSMUSG00000046561	76548	0,94
Cckbr	cholecystokinin B receptor	ENSMUSG00000030898	45082	0,94
Atp2b3	ATPase, Ca++ transporting, plasma membrane 3	ENSMUSG00000031376	67920	0,94
AY358078	cDNA sequence AY358078	ENSMUSG00000050961	26314	0,94
Tdrd5	tudor domain containing 5	ENSMUSG00000060985	48369	0,94
Colq	collagen-like tail subunit (single strand of homotrimer) of asymmetric acetylcholinesterase	ENSMUSG00000057606	68262	0,94
Ass1	argininosuccinate synthetase 1	ENSMUSG00000076441	50466	0,94
Tns4	tensin 4	ENSMUSG00000017607	23629	0,94

		ENSMUSG00000096574	22173	0,94
H2-Q4	histocompatibility 2, Q region locus 4	ENSMUSG00000035929	5674	0,94
Prickle4	prickle homolog 4 (Drosophila)	ENSMUSG00000096549	6220	0,94
Gm3020	predicted gene 3020	ENSMUSG00000079402	21186	0,94
Skint2	selection and upkeep of intraepithelial T cells 2	ENSMUSG00000034359	90647	0,94
		ENSMUSG00000094475	9883	0,94
Ccdc109b	coiled-coil domain containing 109B	ENSMUSG00000027994	55247	0,94
Il18bp	interleukin 18 binding protein	ENSMUSG00000070427	3077	0,94
		ENSMUSG00000090592	2720	0,94
Cd96	CD96 antigen	ENSMUSG00000022657	84595	0,94
Slfn8	schlafen 8	ENSMUSG00000035208	18653	0,94
Hs3st2	heparan sulfate (glucosamine) 3-O-sulfotransferase 2	ENSMUSG00000046321	109473	0,94
Vmn1r32	vomeronasal 1 receptor 32	ENSMUSG00000062905	7526	0,94
Tgtp1	T cell specific GTPase 1	ENSMUSG00000078922	8846	0,94
Glod5	glyoxalase domain containing 5	ENSMUSG00000031163	14293	0,94
		ENSMUSG00000072944	55908	0,94
Pkd2l1	polycystic kidney disease 2-like 1	ENSMUSG00000037578	44806	0,94
Vpreb1	pre-B lymphocyte gene 1	ENSMUSG00000059305	2442	0,94
Enthd1	ENTH domain containing 1	ENSMUSG00000050439	108231	0,94
Cnga3	cyclic nucleotide gated channel alpha 3	ENSMUSG00000026114	45049	0,94
Pla2g1b	phospholipase A2, group IB, pancreas	ENSMUSG00000029522	8461	0,94
Hist1h2bq	histone cluster 1, H2bq	ENSMUSG00000069307	3788	0,94
Gm156	predicted gene 156	ENSMUSG00000071158	17703	0,94
		ENSMUSG00000078875	11832	0,94
Als2cr12	amyotrophic lateral sclerosis 2 (juvenile) chromosome region, candidate 12 (human)	ENSMUSG00000047528	37873	0,94
		ENSMUSG00000092421	15807	0,94
		ENSMUSG00000095648	8235	0,94
Gpr119	G-protein coupled receptor 119	ENSMUSG00000051209	6500	0,94
Hist1h2bg	histone cluster 1, H2bg	ENSMUSG00000058385	618	0,94
Amdhd1	amidohydrolase domain containing 1	ENSMUSG00000015890	16696	0,94
		ENSMUSG00000060678	395	0,94
Efcab4b	EF-hand calcium binding domain 4B	ENSMUSG00000061414	51964	0,94
		ENSMUSG00000062727	479	0,94
Mixl1	Mix1 homeobox-like 1 (Xenopus laevis)	ENSMUSG00000026497	3992	0,94
Nox3	NADPH oxidase 3	ENSMUSG00000023802	61022	0,94
Usp26	ubiquitin specific peptidase 26	ENSMUSG00000055780	47275	0,94
Tgm3	transglutaminase 3, E polypeptide	ENSMUSG00000027401	38051	0,94
Fbxw18	F-box and WD-40 domain protein 18	ENSMUSG00000074059	25967	0,94
Btbd16	BTB (POZ) domain containing 16	ENSMUSG00000040298	51829	0,94
Tlr13	toll-like receptor 13	ENSMUSG00000033777	17290	0,94
4930590J08Rik	RIKEN cDNA 4930590J08 gene	ENSMUSG00000034063	35882	0,94
Slc9a5	solute carrier family 9 (sodium/hydrogen exchanger), member 5	ENSMUSG00000014786	21624	0,94
Tekt5	tektin 5	ENSMUSG00000039179	37543	0,94

		ENSMUSG00000090121	29746	0,94
Gabrd	gamma-aminobutyric acid (GABA) A receptor, subunit delta	ENSMUSG00000029054	13133	0,94
Aloxe3	arachidonate lipoxygenase 3	ENSMUSG00000020892	23220	0,94
Iqcf4	IQ motif containing F4	ENSMUSG00000041009	2647	0,94
		ENSMUSG00000096218	37469	0,94
Olah	oleoyl-ACP hydrolase	ENSMUSG00000026645	24610	0,94
Nkpd1	NTPase, KAP family P-loop domain containing 1	ENSMUSG00000060621	6320	0,94
Ppp1r3g	protein phosphatase 1, regulatory (inhibitor) subunit 3G	ENSMUSG00000050423	11550	0,94
Fam186b	family with sequence similarity 186, member B	ENSMUSG00000078907	16163	0,94
Ckmt2	creatine kinase, mitochondrial 2	ENSMUSG00000021622	23499	0,94
Grpr	gastrin releasing peptide receptor	ENSMUSG00000031364	35774	0,94
Defb39	defensin beta 39	ENSMUSG00000061847	11985	0,94
Scin	scinderin	ENSMUSG00000002565	74460	0,94
Tex35	testis expressed 35	ENSMUSG00000026592	9533	0,94
Slc10a6	solute carrier family 10 (sodium/bile acid cotransporter family), member 6	ENSMUSG00000029321	23693	0,94
Zp3	zona pellucida glycoprotein 3	ENSMUSG00000004948	8526	0,94
		ENSMUSG00000069830	52469	0,94
Ppef2	protein phosphatase, EF hand calcium-binding domain 2	ENSMUSG00000029410	26186	0,94
Tnfsf4	tumor necrosis factor (ligand) superfamily, member 4	ENSMUSG00000026700	23002	0,94
Grid2ip	glutamate receptor, ionotropic, delta 2 (Grid2) interacting protein 1	ENSMUSG00000010825	34815	0,94
Hyal5	hyaluronoglucosaminidase 5	ENSMUSG00000029678	15897	0,94
Vwde	von Willebrand factor D and EGF domains	ENSMUSG00000079679	39353	0,94
Dgat2l6	diacylglycerol O-acyltransferase 2-like 6	ENSMUSG00000067597	21307	0,94
Vmn2r117	vomeronasal 2, receptor 117	ENSMUSG00000091407	19923	0,94
		ENSMUSG00000047508	2857	0,94
		ENSMUSG00000096084	6589	0,94
Fbxw25	F-box and WD-40 domain protein 25	ENSMUSG00000094992	19531	0,94
Bco2	beta-carotene oxygenase 2	ENSMUSG00000032066	22095	0,94
Csta	cystatin A	ENSMUSG00000034362	11296	0,94
Aldh8a1	aldehyde dehydrogenase 8 family, member A1	ENSMUSG00000037542	19295	0,94
Gm15140	predicted gene 15140	ENSMUSG00000094592	11053	0,94
Olfr619	olfactory receptor 619	ENSMUSG00000073944	1124	0,94
Olfr1043	olfactory receptor 1043	ENSMUSG00000075201	945	0,94
Col6a2	collagen, type VI, alpha 2	ENSMUSG00000020241	27869	0,87
Mxra8	matrix-remodelling associated 8	ENSMUSG00000029070	4409	0,87
Postn	periostin, osteoblast specific factor	ENSMUSG00000027750	29926	0,87
		ENSMUSG00000069171	10785	0,87
Arhgef25	Rho guanine nucleotide exchange factor (GEF) 25	ENSMUSG00000019467	7534	0,87
Krt5	keratin 5	ENSMUSG00000061527	5822	0,87
Crispld1	cysteine-rich secretory protein LCCL domain containing 1	ENSMUSG00000025776	39300	0,87

Rspo3	R-spondin 3 homolog (Xenopus laevis)	ENSMUSG00000019880	82759	0,87
Esam	endothelial cell-specific adhesion molecule	ENSMUSG00000001946	10242	0,87
Itm2a	integral membrane protein 2A	ENSMUSG00000031239	6278	0,87
Ednra	endothelin receptor type A	ENSMUSG00000031616	61434	0,87
Chst2	carbohydrate sulfotransferase 2	ENSMUSG00000033350	6342	0,87
Oaf	OAF homolog (Drosophila)	ENSMUSG00000032014	18539	0,87
Mn1	meningioma 1	ENSMUSG00000070576	39672	0,87
Vwa1	von Willebrand factor A domain containing 1	ENSMUSG00000042116	6550	0,87
Abcg2	ATP-binding cassette, sub-family G (WHITE), member 2	ENSMUSG00000029802	108347	0,87
Rlbp1	retinaldehyde binding protein 1	ENSMUSG00000039194	12157	0,87
Aspa	aspartoacylase	ENSMUSG00000020774	21816	0,87
Scube1	signal peptide, CUB domain, EGF-like 1	ENSMUSG00000016763	122439	0,87
Chst14	carbohydrate (N-acetylgalactosamine 4-0) sulfotransferase 14	ENSMUSG00000074916	2089	0,87
C1qtnf7	C1q and tumor necrosis factor related protein 7	ENSMUSG00000061535	103249	0,87
Dll3	delta-like 3 (Drosophila)	ENSMUSG00000003436	8686	0,87
Gamt	guanidinoacetate methyltransferase	ENSMUSG00000020150	2862	0,87
Tmem178	transmembrane protein 178	ENSMUSG00000024245	57185	0,87
Rac2	RAS-related C3 botulinum substrate 2	ENSMUSG00000033220	13615	0,87
5033430I15Rik	RIKEN cDNA 5033430I15 gene	ENSMUSG00000038152	1248	0,87
Spata22	spermatogenesis associated 22	ENSMUSG00000069825	26173	0,87
Mansc1	MANSC domain containing 1	ENSMUSG00000032718	23279	0,87
Fndc1	fibronectin type III domain containing 1	ENSMUSG00000071984	88734	0,87
		ENSMUSG00000095159	1335	0,87
Cd109	CD109 antigen	ENSMUSG00000046186	100708	0,87
Sphk1	sphingosine kinase 1	ENSMUSG00000061878	5750	0,87
Lor	loricrin	ENSMUSG00000043165	2868	0,87
Hebp1	heme binding protein 1	ENSMUSG00000042770	30697	0,87
6430571L13Rik	RIKEN cDNA 6430571L13 gene	ENSMUSG00000037977	11821	0,87
Clec2d	C-type lectin domain family 2, member d	ENSMUSG00000030157	5920	0,87
Gjb3	gap junction protein, beta 3	ENSMUSG00000042367	5610	0,87
Chrd	chordin	ENSMUSG00000006958	9258	0,87
Ninj1	ninjurin 1	ENSMUSG00000037966	8760	0,87
Arhgap36	Rho GTPase activating protein 36	ENSMUSG00000036198	36300	0,87
Fcgr2b	Fc receptor, IgG, low affinity IIb	ENSMUSG00000026656	15990	0,87
Myot	myotilin	ENSMUSG00000024471	21651	0,87
		ENSMUSG00000092130	2364	0,87
Tigd3	tigger transposable element derived 3	ENSMUSG00000044390	2969	0,87
Fancd2os	Fancd2 opposite strand	ENSMUSG00000033963	3955	0,87
2600006K01Rik	RIKEN cDNA 2600006K01 gene	ENSMUSG00000039798	1060	0,87
Slc13a1	solute carrier family 13 (sodium/sulfate symporters), member 1	ENSMUSG00000029700	79810	0,87
Mrgpre	MAS-related GPR, member E	ENSMUSG00000048965	6136	0,87

Ptges3l	prostaglandin E synthase 3 (cytosolic)-like	ENSMUSG00000097487	6522	0,87
Tgm5	transglutaminase 5	ENSMUSG00000053675	39731	0,87
Xkr5	X Kell blood group precursor-related family, member 5	ENSMUSG00000039814	18247	0,87
Ermap	erythroblast membrane-associated protein	ENSMUSG00000028644	14555	0,87
		ENSMUSG00000030636	7887	0,87
Gm9934	predicted gene 9934	ENSMUSG00000054061	28947	0,87
Gm5622	predicted gene 5622	ENSMUSG00000079244	110164	0,87
		ENSMUSG00000082766	18310	0,87
Lst1	leukocyte specific transcript 1	ENSMUSG00000073412	3345	0,87
Tmem81	transmembrane protein 81	ENSMUSG00000048174	2408	0,87
Gm8369	predicted gene 8369	ENSMUSG00000058470	20540	0,87
Spink5	serine peptidase inhibitor, Kazal type 5	ENSMUSG00000055561	59267	0,87
Arr3	arrestin 3, retinal	ENSMUSG00000060890	12997	0,87
A130051J06Rik	RIKEN cDNA A130051J0 gene	ENSMUSG00000097643	3787	0,87
Gm5796	predicted gene 5796	ENSMUSG00000096775	17430	0,87
Itgb1bp2	integrin beta 1 binding protein 2	ENSMUSG00000031312	4454	0,87
Hist1h3a	histone cluster 1, H3a	ENSMUSG00000069265	534	0,87
Zfp459	zinc finger protein 459	ENSMUSG00000055560	15697	0,87
Gm14295	predicted gene 14295	ENSMUSG00000078877	12612	0,87
Gm3264	predicted gene 3264	ENSMUSG00000094132	20276	0,87
		ENSMUSG00000079190	29033	0,87
		ENSMUSG00000069303	3788	0,87
Tmc2	transmembrane channel-like gene family 2	ENSMUSG00000060332	69252	0,87
Slc17a3	solute carrier family 17 (sodium phosphate), member 3	ENSMUSG00000036083	21281	0,87
C8g	complement component 8, gamma polypeptide	ENSMUSG00000015083	3069	0,87
Agxt2	alanine-glyoxylate aminotransferase 2	ENSMUSG00000089678	51622	0,87
Gm765	predicted gene 765	ENSMUSG00000090667	104741	0,87
Sash3	SAM and SH3 domain containing 3	ENSMUSG00000031101	15130	0,87
Topaz1	testis and ovary specific PAZ domain containing 1	ENSMUSG00000094985	54729	0,87
		ENSMUSG00000019146	128281	0,87
AI182371	expressed sequence AI182371	ENSMUSG00000035875	17498	0,87
Msr1	macrophage scavenger receptor 1	ENSMUSG00000025044	60979	0,87
		ENSMUSG00000048652	40398	0,87
Syt6	synaptotagmin VI	ENSMUSG00000027849	70337	0,87
Oasl1	2'-5' oligoadenylate synthetase-like 1	ENSMUSG00000041827	14676	0,87
		ENSMUSG00000054648	4393	0,87
Gucy1b2	guanylate cyclase 1, soluble, beta 2	ENSMUSG00000021933	63614	0,87
Zpbp2	zona pellucida binding protein 2	ENSMUSG00000017195	7569	0,87
Gm13154	predicted gene 13154	ENSMUSG00000065999	31922	0,87
		ENSMUSG00000073497	45262	0,87
Gm6772	predicted gene 6772	ENSMUSG00000059114	29413	0,87
Ubash3a	ubiquitin associated and SH3 domain containing, A	ENSMUSG00000042345	34330	0,87
1700022P22Rik	RIKEN cDNA 1700022P22 gene	ENSMUSG00000057072	6120	0,87
F830016B08Rik	RIKEN cDNA F830016B08 gene	ENSMUSG00000090942	9637	0,87

Prodh2	proline dehydrogenase (oxidase) 2	ENSMUSG00000036892	19781	0,87
Fgfr4	fibroblast growth factor receptor 4	ENSMUSG00000005320	16120	0,87
Jsrp1	junctional sarcoplasmic reticulum protein 1	ENSMUSG00000020216	5003	0,87
Kcnk7	potassium channel, subfamily K, member 7	ENSMUSG00000024936	2735	0,87
Gcsam	germinal center associated, signaling and motility	ENSMUSG00000022659	12488	0,87
Lin7b	lin-7 homolog B (C. elegans)	ENSMUSG00000003872	2693	0,87
Wbp2nl	WBP2 N-terminal like	ENSMUSG00000022455	15575	0,87
Dqx1	DEAQ RNA-dependent ATPase	ENSMUSG00000009145	9367	0,87
		ENSMUSG00000032473	27807	0,87
Siglec15	sialic acid binding Ig-like lectin 15	ENSMUSG00000091055	13782	0,87
Kcp	kielin/chordin-like protein	ENSMUSG00000059022	34791	0,87
Zap70	zeta-chain (TCR) associated protein kinase	ENSMUSG00000026117	20956	0,87
Clec7a	C-type lectin domain family 7, member a	ENSMUSG00000079293	11187	0,87
Hpse	heparanase	ENSMUSG00000035273	40233	0,87
Tm6sf2	transmembrane 6 superfamily member 2	ENSMUSG00000036151	7143	0,87
Fam196b	family with sequence similarity 196, member B	ENSMUSG00000069911	107819	0,87
E030019B06Rik	RIKEN cDNA E030019B06 gene	ENSMUSG00000070357	15379	0,87
Gm4884	predicted gene 4884	ENSMUSG00000048312	12584	0,87
		ENSMUSG00000050709	2717	0,87
Fgf8	fibroblast growth factor 8	ENSMUSG00000025219	6118	0,87
Amn	amnionless	ENSMUSG00000021278	5316	0,87
2610034M16Rik	RIKEN cDNA 2610034M16 gene	ENSMUSG00000024227	112568	0,87
		ENSMUSG00000049173	15473	0,87
Ankdd1a	ankyrin repeat and death domain containing 1A	ENSMUSG00000066510	15547	0,87
Gm10610	predicted gene 10610	ENSMUSG00000094841	7459	0,87
9930111J21Rik2	RIKEN cDNA 9930111J21 gene 2	ENSMUSG00000069892	35369	0,87
		ENSMUSG00000061371	2913	0,87
Mmp21	matrix metallopeptidase 21	ENSMUSG00000030981	5792	0,87
Gpr123	G protein-coupled receptor 123	ENSMUSG00000025475	43915	0,87
		ENSMUSG00000069041	16739	0,87
Pax5	paired box gene 5	ENSMUSG00000014030	180302	0,87
Ccdc63	coiled-coil domain containing 63	ENSMUSG00000043036	30918	0,87
Oc90	otoconin 90	ENSMUSG00000015001	36345	0,87
3110021A11Rik	RIKEN cDNA 3110021A11 gene	ENSMUSG00000050604	821	0,87
Lrrc9	leucine rich repeat containing 9	ENSMUSG00000021090	72469	0,87
Pou3f1	POU domain, class 3, transcription factor 1	ENSMUSG00000090125	3010	0,87
Tacr2	tachykinin receptor 2	ENSMUSG00000020081	13553	0,87
Vmn2r18	vomeronasal 2, receptor 18	ENSMUSG00000091794	25264	0,87
Cdh9	cadherin 9	ENSMUSG00000025370	78994	0,87
		ENSMUSG00000030604	7857	0,87
Gm996	predicted gene 996	ENSMUSG00000029419	4684	0,87
Tecrl	trans-2,3-enoyl-CoA reductase-like	ENSMUSG00000049537	77051	0,87
Slc22a29	solute carrier family 22. member 29	ENSMUSG00000075044	58736	0,87
4933425L06Rik	RIKEN cDNA 4933425L06 gene	ENSMUSG00000021718	39661	0,87
Scgb1b30	secretoglobin, family 1B, member 30	ENSMUSG00000078752	5407	0,87
		ENSMUSG00000087230	25624	0,87

Gstm3	glutathione S-transferase, mu 3	ENSMUSG00000004038	26951	0,87
Prss27	protease, serine, 27	ENSMUSG00000050762	7811	0,87
Olfr1384	olfactory receptor 1384	ENSMUSG00000044170	1034	0,87
Olfr131	olfactory receptor 131	ENSMUSG00000043312	6724	0,87
Olfr749	olfactory receptor 749	ENSMUSG00000059069	8106	0,87
Adra2c	adrenergic receptor, alpha 2c	ENSMUSG00000045318	3196	0,80
Spp1	secreted phosphoprotein 1	ENSMUSG00000029304	5933	0,80
Col6a3	collagen, type VI, alpha 3	ENSMUSG00000048126	78049	0,80
Prss33	protease, serine, 33	ENSMUSG00000049620	2697	0,80
Fam198b	family with sequence similarity 198, member B	ENSMUSG00000027955	61748	0,80
Lysmd2	LysM, putative peptidoglycan-binding, domain containing 2	ENSMUSG00000032184	12042	0,80
9430020K01Rik	RIKEN cDNA 9430020K01 gene	ENSMUSG00000033960	47940	0,80
Grp	gastrin releasing peptide	ENSMUSG00000024517	13759	0,80
Itih2	inter-alpha trypsin inhibitor, heavy chain 2	ENSMUSG00000037254	36804	0,80
Snai1	snail homolog 1 (Drosophila)	ENSMUSG00000042821	4620	0,80
Dll4	delta-like 4 (Drosophila)	ENSMUSG00000027314	10179	0,80
Gm6578	predicted gene 6578	ENSMUSG00000057223	10066	0,80
Foxd3	forkhead box D3	ENSMUSG00000067261	2373	0,80
Lrriq4	leucine-rich repeats and IQ motif containing 4	ENSMUSG00000027703	27925	0,80
Gm9945	predicted gene 9945	ENSMUSG00000054450	932	0,80
Nog	noggin	ENSMUSG00000048616	1922	0,80
Magee2	melanoma antigen, family E, 2	ENSMUSG00000031224	2313	0,80
Tmem45a	transmembrane protein 45a	ENSMUSG00000022754	81006	0,80
Grem2	gremlin 2 homolog, cysteine knot superfamily (Xenopus laevis)	ENSMUSG00000050069	88035	0,80
Lrp3	low density lipoprotein receptor-related protein 3	ENSMUSG00000001802	15344	0,80
Flnc	filamin C, gamma	ENSMUSG00000068699	28736	0,80
Efhd1	EF hand domain containing 1	ENSMUSG00000026255	46477	0,80
Sat2	spermidine/spermine N1-acetyl transferase 2	ENSMUSG00000069835	1847	0,80
Gm4477	predicted gene 4477	ENSMUSG00000089694	6479	0,80
Iigp1	interferon inducible GTPase 1	ENSMUSG00000054072	16599	0,80
Mmp17	matrix metallopeptidase 17	ENSMUSG00000029436	24707	0,80
2610042L04Rik	RIKEN cDNA 2610042L04 gene	ENSMUSG00000079388	19925	0,80
Capn8	calpain 8	ENSMUSG00000038599	67346	0,80
Gm10382	predicted gene 10382	ENSMUSG00000072612	1233	0,80
Mlkl	mixed lineage kinase domain-like	ENSMUSG00000012519	26381	0,80
Csn1s1	casein alpha s1	ENSMUSG00000070702	16350	0,80
		ENSMUSG00000071273	837	0,80
Ano9	anoctamin 9	ENSMUSG00000054662	16588	0,80
Smad9	SMAD family member 9	ENSMUSG00000027796	45676	0,80
		ENSMUSG00000093844	222	0,80
Nudt17	nudix (nucleoside diphosphate linked moiety X)-type motif 17	ENSMUSG00000028100	2669	0,80
Nefm	neurofilament, medium polypeptide	ENSMUSG00000022054	42257	0,80
		ENSMUSG00000069310	630	0,80
Wfikkn2	WAP, follistatin/kazal, immunoglobulin, kunitz and netrin domain containing 2	ENSMUSG00000044177	10050	0,80
Rwdd2b	RWD domain containing 2B	ENSMUSG00000041079	7167	0,80

Plekhf1	pleckstrin homology domain containing, family F (with FYVE domain) member 1	ENSMUSG00000074170	7341	0,80
		ENSMUSG00000026222	60011	0,80
Gm11128	predicted gene 11128	ENSMUSG00000079495	12931	0,80
Fcer1a	Fc receptor, IgE, high affinity I, alpha polypeptide	ENSMUSG00000005339	5959	0,80
Ager	advanced glycosylation end product-specific receptor	ENSMUSG00000015452	3073	0,80
		ENSMUSG00000042385	54301	0,80
		ENSMUSG00000097481	1069	0,80
Atp4b	ATPase, H+/K+ exchanging, beta polypeptide	ENSMUSG00000031449	10570	0,80
Fam71b	family with sequence similarity 71, member B	ENSMUSG00000020401	11424	0,80
Zfp677	zinc finger protein 677	ENSMUSG00000062743	15518	0,80
Apol7b	apolipoprotein L 7b	ENSMUSG00000068252	25283	0,80
Grin2c	glutamate receptor, ionotropic, NMDA2C (epsilon 3)	ENSMUSG00000020734	18075	0,80
Gabra2	gamma-aminobutyric acid (GABA) A receptor, subunit alpha 2	ENSMUSG00000000560	134793	0,80
Aard	alanine and arginine rich domain containing protein	ENSMUSG00000068522	5616	0,80
Spag4	sperm associated antigen 4	ENSMUSG00000038180	5443	0,80
Akr1d1	aldo-keto reductase family 1, member D1	ENSMUSG00000038641	38643	0,80
Ly6g6c	lymphocyte antigen 6 complex, locus G6C	ENSMUSG00000092586	4663	0,80
Zfp879	zinc finger protein 879	ENSMUSG00000044296	9522	0,80
Ptgs2	prostaglandin-endoperoxide synthase 2	ENSMUSG00000032487	7887	0,80
Tac1	tachykinin 1	ENSMUSG00000061762	7903	0,80
Tnfrsf17	tumor necrosis factor receptor superfamily, member 17	ENSMUSG00000022496	6263	0,80
Pcdhb1	protocadherin beta 1	ENSMUSG00000051663	2457	0,80
Zpld1	zona pellucida like domain containing 1	ENSMUSG00000064310	72812	0,80
		ENSMUSG00000097022	303	0,80
		ENSMUSG00000074780	461	0,80
Slc2a6	solute carrier family 2 (facilitated glucose transporter), member 6	ENSMUSG00000036067	6636	0,80
Car15	carbonic anhydrase 15	ENSMUSG00000090236	3089	0,80
Espnl	espin-like	ENSMUSG00000049515	26232	0,80
Spib	Spi-B transcription factor (Spi-1/PU.1 related)	ENSMUSG00000008193	6077	0,80
Enpp3	ectonucleotide pyrophosphatase/phosphodiesterase 3	ENSMUSG00000019989	62382	0,80
Gata1	GATA binding protein 1	ENSMUSG00000031162	18812	0,80
Ttc22	tetratricopeptide repeat domain 22	ENSMUSG00000034919	17758	0,80
Dnase1	deoxyribonuclease I	ENSMUSG00000005980	3083	0,80
Pcdhb5	protocadherin beta 5	ENSMUSG00000063687	3533	0,80
Hnf4a	hepatic nuclear factor 4, alpha	ENSMUSG00000017950	66103	0,80
Psors1c2	psoriasis susceptibility 1 candidate 2 (human)	ENSMUSG00000024409	1447	0,80
Trim6	tripartite motif-containing 6	ENSMUSG00000072244	16360	0,80

Zfp296	zinc finger protein 296	ENSMUSG00000011267	3370	0,80
Bhmt2	betaine-homocysteine methyltransferase 2	ENSMUSG00000042118	18583	0,80
		ENSMUSG00000046636	1116	0,80
Gpat2	glycerol-3-phosphate acyltransferase 2, mitochondrial	ENSMUSG00000046338	10894	0,80
1700008O03Rik	RIKEN cDNA 1700008O03 gene	ENSMUSG00000008028	25778	0,80
B3gnt5	UDP-GlcNAc:betaGal beta-1,3-N-acetylglucosaminyltransferase 5	ENSMUSG00000022686	12546	0,80
BC021785	cDNA sequence BC021785	ENSMUSG00000038528	28586	0,80
Gm7008	predicted gene 7008	ENSMUSG00000035983	6427	0,80
Fgf7	fibroblast growth factor 7	ENSMUSG00000027208	56528	0,80
Gm1564	predicted gene 1564	ENSMUSG00000051455	18522	0,80
Tspo2	translocator protein 2	ENSMUSG00000023995	3567	0,80
Gm4841	predicted gene 4841	ENSMUSG00000068606	4967	0,80
Vmn2r26	vomeronasal 2, receptor 26	ENSMUSG00000096630	37278	0,80
Hist1h4k	histone cluster 1, H4k	ENSMUSG00000064288	312	0,80
Fa2h	fatty acid 2-hydroxylase	ENSMUSG00000033579	48690	0,80
		ENSMUSG00000020548	7982	0,80
		ENSMUSG00000087346	2039	0,80
Gpr44	G protein-coupled receptor 44	ENSMUSG00000034117	5352	0,80
Tnnc1	troponin C, cardiac/slow skeletal	ENSMUSG00000091898	3418	0,80
Clec1a	C-type lectin domain family 1, member a	ENSMUSG00000033082	25317	0,80
Mtnr1b	melatonin receptor 1B	ENSMUSG00000050901	50029	0,80
Cyct	cytochrome c, testis	ENSMUSG00000056436	6509	0,80
Gm14548	predicted gene 14548	ENSMUSG00000074417	13879	0,80
Lypd3	Ly6/Plaur domain containing 3	ENSMUSG00000057454	4547	0,80
Pfn4	profilin family, member 4	ENSMUSG00000020639	8972	0,80
Rasl10a	RAS-like, family 10, member A	ENSMUSG00000034209	2258	0,80
Zyg11a	zyg-11 family member A, cell cycle regulator	ENSMUSG00000034645	35989	0,80
Gnat1	guanine nucleotide binding protein, alpha transducing 1	ENSMUSG00000034837	5116	0,80
4933402D24Rik	RIKEN cDNA 4933402D24 gene	ENSMUSG00000025961	14361	0,80
Abcc2	ATP-binding cassette, sub-family C (CFTR/MRP), member 2	ENSMUSG00000025194	55760	0,80
H2-M2	histocompatibility 2, M region locus 2	ENSMUSG00000016283	2702	0,80
Ddx4	DEAD (Asp-Glu-Ala-Asp) box polypeptide 4	ENSMUSG00000021758	53978	0,80
Abca15	ATP-binding cassette, sub-family A (ABC1), member 15	ENSMUSG00000054746	79004	0,80
Hormad1	HORMA domain containing 1	ENSMUSG00000028109	27995	0,80
Gys2	glycogen synthase 2	ENSMUSG00000030244	50497	0,80
		ENSMUSG00000090353	1113	0,80
Bcl2a1b	B cell leukemia/lymphoma 2 related protein A1b	ENSMUSG00000089929	8619	0,80
		ENSMUSG00000094447	7246	0,80
Rpl3l	ribosomal protein L3-like	ENSMUSG00000002500	8324	0,80
Arl9	ADP-ribosylation factor-like 9	ENSMUSG00000063820	6552	0,80
Spo11	SPO11 meiotic protein covalently bound to DSB homolog (S. cerevisiae)	ENSMUSG00000005883	15877	0,80
Cryaa	crystallin, alpha A	ENSMUSG00000024041	3790	0,80
		ENSMUSG00000096265	22491	0,80

Nr5a1	nuclear receptor subfamily 5, group A, member 1	ENSMUSG00000026751	21887	0,80
Clec1b	C-type lectin domain family 1, member b	ENSMUSG00000030159	12039	0,80
Tlr7	toll-like receptor 7	ENSMUSG00000044583	25630	0,80
Tph2	tryptophan hydroxylase 2	ENSMUSG00000006764	106382	0,80
		ENSMUSG00000038663	33637	0,80
Phf11d	PHD finger protein 11D	ENSMUSG00000068245	18064	0,80
Ms4a15	membrane-spanning 4-domains, subfamily A, member 15	ENSMUSG00000067571	14944	0,80
Cckar	cholecystokinin A receptor	ENSMUSG00000029193	9220	0,80
Krt78	keratin 78	ENSMUSG00000050463	8284	0,80
Myo1g	myosin IG	ENSMUSG00000020437	14418	0,80
		ENSMUSG00000096736	1207	0,80
Tshb	thyroid stimulating hormone, beta subunit	ENSMUSG00000027857	5317	0,80
Cyp3a16	cytochrome P450, family 3, subfamily a, polypeptide 16	ENSMUSG00000038656	33415	0,80
Hamp	hepcidin antimicrobial peptide	ENSMUSG00000050440	1647	0,80
Dkk4	dickkopf homolog 4 (Xenopus laevis)	ENSMUSG00000031535	3494	0,80
Iapp	islet amyloid polypeptide	ENSMUSG00000041681	5539	0,80
Adcyap1r1	adenylate cyclase activating polypeptide 1 receptor 1	ENSMUSG00000029778	49474	0,80
Slc7a12	solute carrier family 7 (cationic amino acid transporter, y+ system), member 12	ENSMUSG00000039710	25134	0,80
4931440L10Rik	RIKEN cDNA 4931440L10 gene	ENSMUSG00000046367	8742	0,80
Hist1h2bn	histone cluster 1, H2bn	ENSMUSG00000095217	381	0,80
Olfr1496	olfactory receptor 1496	ENSMUSG00000048356	1067	0,80
Olfr470	olfactory receptor 470	ENSMUSG00000094289	945	0,80
Olfr1373	olfactory receptor 1373	ENSMUSG00000062204	936	0,80
Olfr920	olfactory receptor 920	ENSMUSG00000061039	4068	0,80
Olfr578	olfactory receptor 578	ENSMUSG00000045792	942	0,80
		ENSMUSG00000045392	10044	0,80
Olfr1250	olfactory receptor 1250	ENSMUSG00000075078	4822	0,80
Olfr1086	olfactory receptor 1086	ENSMUSG00000075175	933	0,80
Olfr1406	olfactory receptor 1406	ENSMUSG00000058981	1063	0,80
Tpm2	tropomyosin 2, beta	ENSMUSG00000028464	9055	0,72
Kcnj10	potassium inwardly-rectifying channel, subfamily J, member 10	ENSMUSG00000044708	32876	0,72
Kdr	kinase insert domain protein receptor	ENSMUSG00000062960	45632	0,72
Ets1	E26 avian leukemia oncogene 1, 5' domain	ENSMUSG00000032035	61779	0,72
Ecm1	extracellular matrix protein 1	ENSMUSG00000028108	5423	0,72
Pth2	parathyroid hormone 2	ENSMUSG00000038300	844	0,72
Sun3	Sad1 and UNC84 domain containing 3	ENSMUSG00000040985	32938	0,72
Gm10047	predicted gene 10047	ENSMUSG00000058736	1330	0,72
Gstm6	glutathione S-transferase, mu 6	ENSMUSG00000068762	4903	0,72
Helt	helt bHLH transcription factor	ENSMUSG00000047171	2622	0,72
Hyal2	hyaluronoglucosaminidase 2	ENSMUSG00000010047	3660	0,72
Tmod4	tropomodulin 4	ENSMUSG00000005628	4734	0,72
Iglon5	IgLON family member 5	ENSMUSG00000013367	17169	0,72

Ppp1r14a	protein phosphatase 1, regulatory (inhibitor) subunit 14A	ENSMUSG00000037166	4071	0,72
Foxs1	forkhead box S1	ENSMUSG00000074676	1311	0,72
Med18	mediator of RNA polymerase II transcription, subunit 18 homolog (yeast)	ENSMUSG00000066042	5191	0,72
Nrl	neural retina leucine zipper gene	ENSMUSG00000040632	6004	0,72
		ENSMUSG00000097070	1502	0,72
Dctd	dCMP deaminase	ENSMUSG00000031562	54142	0,72
Cntfr	ciliary neurotrophic factor receptor	ENSMUSG00000028444	39592	0,72
Cryz	crystallin, zeta	ENSMUSG00000028199	25986	0,72
Gm10324	predicted gene 10324	ENSMUSG00000071302	4227	0,72
Slc36a2	solute carrier family 36 (proton/amino acid symporter), member 2	ENSMUSG00000020264	26608	0,72
Socs1	suppressor of cytokine signaling 1	ENSMUSG00000038037	1729	0,72
Tmem217	transmembrane protein 217	ENSMUSG00000079580	23561	0,72
Zdhhc19	zinc finger, DHHC domain containing 19	ENSMUSG00000052363	65702	0,72
Scn1b	sodium channel, voltage-gated, type I, beta	ENSMUSG00000019194	10421	0,72
Slc13a3	solute carrier family 13 (sodium-dependent dicarboxylate transporter), member 3	ENSMUSG00000018459	68203	0,72
Troap	trophinin associated protein	ENSMUSG00000032783	8435	0,72
Gm10430	predicted gene 10430	ENSMUSG00000072855	1844	0,72
Tnfrsf13b	tumor necrosis factor receptor superfamily, member 13b	ENSMUSG00000010142	22618	0,72
Cml3	camello-like 3	ENSMUSG00000051262	5096	0,72
4933406M09Rik	RIKEN cDNA 4933406M09 gene	ENSMUSG00000050526	5042	0,72
Gm10549	predicted gene 10549	ENSMUSG00000073610	8286	0,72
Glp1r	glucagon-like peptide 1 receptor	ENSMUSG00000024027	34644	0,72
Aldoart1	aldolase 1 A, retrogene 1	ENSMUSG00000059343	2052	0,72
Hoxa3	homeobox A3	ENSMUSG00000079560	49981	0,72
Gpr176	G protein-coupled receptor 176	ENSMUSG00000040133	96310	0,72
Tnr	tenascin R	ENSMUSG00000015829	186888	0,72
Asprv1	aspartic peptidase, retroviral-like 1	ENSMUSG00000033508	1529	0,72
		ENSMUSG00000078887	12030	0,72
		ENSMUSG00000050087	5238	0,72
		ENSMUSG00000096718	5827	0,72
F12	coagulation factor XII (Hageman factor)	ENSMUSG00000021492	8836	0,72
Lcat	lecithin cholesterol acyltransferase	ENSMUSG00000035237	3832	0,72
Tmem144	transmembrane protein 144	ENSMUSG00000027956	29515	0,72
Edn1	endothelin 1	ENSMUSG00000021367	6720	0,72
		ENSMUSG00000094932	9883	0,72
Casq1	calsequestrin 1	ENSMUSG00000007122	9975	0,72
Asb15	ankyrin repeat and SOCS box-containing 15	ENSMUSG00000029685	45021	0,72
Pf4	platelet factor 4	ENSMUSG00000029373	947	0,72
Gm13306	predicted gene 13306	ENSMUSG00000073877	5404	0,72
Gm129	predicted gene 129	ENSMUSG00000038550	3749	0,72
Gucy2e	guanylate cyclase 2e	ENSMUSG00000020890	18920	0,72
Plac1	placental specific protein 1	ENSMUSG00000061082	170117	0,72
Ccdc144b	coiled-coil domain containing 144B	ENSMUSG00000047696	46301	0,72

Slco2b1	solute carrier organic anion transporter family, member 2b1	ENSMUSG00000030737	53537	0,72
Fgfbp1	fibroblast growth factor binding protein 1	ENSMUSG00000048373	2898	0,72
		ENSMUSG00000027460	34128	0,72
Fignl2	fidgetin-like 2	ENSMUSG00000095440	4206	0,72
Kmo	kynurenine 3-monooxygenase (kynurenine 3-hydroxylase)	ENSMUSG00000039783	41736	0,72
Amhr2	anti-Mullerian hormone type 2 receptor	ENSMUSG00000023047	9264	0,72
Pcdhb4	protocadherin beta 4	ENSMUSG00000045689	3719	0,72
Map6d1	MAP6 domain containing 1	ENSMUSG00000041205	8049	0,72
		ENSMUSG00000096727	5777	0,72
Tcea2	transcription elongation factor A (SII), 2	ENSMUSG00000059540	7762	0,72
Mzb1	marginal zone B and B1 cell-specific protein 1	ENSMUSG00000024353	2102	0,72
		ENSMUSG00000092463	7619	0,72
1300002K09Rik	RIKEN cDNA 1300002K09 gene	ENSMUSG00000028327	38345	0,72
Krt79	keratin 79	ENSMUSG00000061397	10993	0,72
Serpinb9e	serine (or cysteine) peptidase inhibitor, clade B, member 9e	ENSMUSG00000062342	11235	0,72
AI747448	expressed sequence AI747448	ENSMUSG00000074195	21609	0,72
Nepn	nephrocan	ENSMUSG00000038624	15636	0,72
Cd53	CD53 antigen	ENSMUSG00000040747	31289	0,72
Ceacam10	carcinoembryonic antigen-related cell adhesion molecule 10	ENSMUSG00000054169	7446	0,72
Gm12302	predicted gene 12302	ENSMUSG00000097886	15291	0,72
Fam19a3	family with sequence similarity 19, member A3	ENSMUSG00000055865	14435	0,72
Clec4a1	C-type lectin domain family 4, member a1	ENSMUSG00000049037	12772	0,72
Myo7b	myosin VIIB	ENSMUSG00000024388	77728	0,72
Cdh17	cadherin 17	ENSMUSG00000028217	59749	0,72
Slamf9	SLAM family member 9	ENSMUSG00000026548	3218	0,72
Cst7	cystatin F (leukocystatin)	ENSMUSG00000068129	8530	0,72
Dazl	deleted in azoospermia-like	ENSMUSG00000010592	14206	0,72
Isl2	insulin related protein 2 (islet 2)	ENSMUSG00000032318	7509	0,72
Pla1a	phospholipase A1 member A	ENSMUSG00000002847	37027	0,72
		ENSMUSG00000069267	620	0,72
Uba7	ubiquitin-like modifier activating enzyme 7	ENSMUSG00000032596	8556	0,72
Ccna1	cyclin A1	ENSMUSG00000027793	9587	0,72
		ENSMUSG00000032948	45543	0,72
		ENSMUSG00000040663	9253	0,72
Chrna3	cholinergic receptor, nicotinic, alpha polypeptide 3	ENSMUSG00000032303	15217	0,72
Tnnt2	troponin T2, cardiac	ENSMUSG00000026414	15875	0,72
		ENSMUSG00000064081	315	0,72
		ENSMUSG00000090622	3337	0,72
Hist1h3i	histone cluster 1, H3i	ENSMUSG00000075032	483	0,72
Lax1	lymphocyte transmembrane adaptor 1	ENSMUSG00000051998	11080	0,72
Areg	amphiregulin	ENSMUSG00000029378	8818	0,72
		ENSMUSG00000036062	28093	0,72
		ENSMUSG00000094296	120303	0,72

Cd226	CD226 antigen	ENSMUSG00000034028	72771	0,72
		ENSMUSG00000079259	57237	0,72
Hist1h1t	histone cluster 1, H1t	ENSMUSG00000036211	909	0,72
Zfp947	zinc finger protein 947	ENSMUSG00000063383	21832	0,72
		ENSMUSG00000093668	3384	0,72
H2-Q2	histocompatibility 2, Q region locus 2	ENSMUSG00000091705	4521	0,72
Ifi202b	interferon activated gene 202B	ENSMUSG00000026535	20276	0,72
Kcnk4	potassium channel, subfamily K, member 4	ENSMUSG00000024957	8806	0,72
2310005G13Rik	RIKEN cDNA 2310005G13 gene	ENSMUSG00000046748	34380	0,72
Prph2	peripherin 2	ENSMUSG00000023978	14475	0,72
Meiob	meiosis specific with OB domains	ENSMUSG00000024155	35406	0,72
Gm15128	predicted gene 15128	ENSMUSG00000095872	67466	0,72
D130052B06Rik	RIKEN cDNA D130052B06 gene	ENSMUSG00000073052	26317	0,72
Allc	allantoicase	ENSMUSG00000020636	28769	0,72
Slc4a9	solute carrier family 4, sodium bicarbonate cotransporter, member 9	ENSMUSG00000024485	13137	0,72
		ENSMUSG00000037390	14397	0,72
		ENSMUSG00000075015	1847	0,72
		ENSMUSG00000081650	3301	0,72
Tnp1	transition protein 1	ENSMUSG00000026182	817	0,72
Usp9y	ubiquitin specific peptidase 9, Y chromosome	ENSMUSG00000069044	160826	0,72
Gm14525	predicted gene 14525	ENSMUSG00000071788	29862	0,72
Mug2	murinoglobulin 2	ENSMUSG00000030131	79170	0,72
Cidec	cell death-inducing DFFA-like effector c	ENSMUSG00000030278	11127	0,72
1700029H14Rik	RIKEN cDNA 1700029H14 gene	ENSMUSG00000031452	11729	0,72
Ivl	involucrin	ENSMUSG00000049128	2804	0,72
Hsd3b3	hydroxy-delta-5-steroid dehydrogenase, 3 beta- and steroid delta-isomerase 3	ENSMUSG00000062410	21605	0,72
Gcgr	glucagon receptor	ENSMUSG00000025127	8288	0,72
Il23a	interleukin 23, alpha subunit p19	ENSMUSG00000025383	1945	0,72
Htr5b	5-hydroxytryptamine (serotonin) receptor 5B	ENSMUSG00000050534	18624	0,72
Clec2l	C-type lectin domain family 2, member L	ENSMUSG00000079598	17796	0,72
Als2cr11	amyotrophic lateral sclerosis 2 (juvenile) chromosome region, candidate 11 (human)	ENSMUSG00000072295	44395	0,72
Fbll1	fibrillarin-like 1	ENSMUSG00000051062	1504	0,72
Ucp3	uncoupling protein 3 (mitochondrial, proton carrier)	ENSMUSG00000032942	13443	0,72
Fibcd1	fibrinogen C domain containing 1	ENSMUSG00000026841	32716	0,72
9130409I23Rik	RIKEN cDNA 9130409I23 gene	ENSMUSG00000038768	9431	0,72
Tmem95	transmembrane protein 95	ENSMUSG00000094845	1335	0,72
Htr2a	5-hydroxytryptamine (serotonin) receptor 2A	ENSMUSG00000034997	66020	0,72
Rab9b	RAB9B, member RAS oncogene family	ENSMUSG00000043463	10609	0,72
5031410I06Rik	RIKEN cDNA 5031410I06 gene	ENSMUSG00000051940	6695	0,72
Rasal1	RAS protein activator like 1 (GAP1 like)	ENSMUSG00000029602	30786	0,72

Spaca1	sperm acrosome associated 1	ENSMUSG00000028264	25318	0,72
Gm6289	predicted gene 6289	ENSMUSG00000074196	16690	0,72
		ENSMUSG00000079010	1632	0,72
Fitm1	fat storage-inducing transmembrane protein 1	ENSMUSG00000022215	1336	0,72
Atp12a	ATPase, H+/K+ transporting, nongastric, alpha polypeptide	ENSMUSG00000022229	23484	0,72
		ENSMUSG00000023781	3652	0,72
Ankrd53	ankyrin repeat domain 53	ENSMUSG00000014747	5683	0,72
Rgs1	regulator of G-protein signaling 1	ENSMUSG00000026358	5271	0,72
1700011A15Rik	RIKEN cDNA 1700011A15 gene	ENSMUSG00000063971	6165	0,72
Defb45	defensin beta 45	ENSMUSG00000062124	6209	0,72
Egr3	early growth response 3	ENSMUSG00000033730	2713	0,72
Klk11	kallikrein related-peptidase 11	ENSMUSG00000067616	4642	0,72
1700001P01Rik	RIKEN cDNA 1700001P01 gene	ENSMUSG00000018543	4438	0,72
Ceacam20	carcinoembryonic antigen-related cell adhesion molecule 20	ENSMUSG00000070777	25702	0,72
Ins2	insulin II	ENSMUSG00000000215	20855	0,72
		ENSMUSG00000095861	17151	0,72
		ENSMUSG00000094840	3577	0,72
Bcl2a1a	B cell leukemia/lymphoma 2 related protein A1a	ENSMUSG00000093809	5497	0,72
Gm15091	predicted gene 15091	ENSMUSG00000095082	43328	0,72
Ttr	transthyretin	ENSMUSG00000061808	9075	0,72
		ENSMUSG00000096795	2550	0,72
Cmtm2b	CKLF-like MARVEL transmembrane domain containing 2B	ENSMUSG00000035785	8520	0,72
Olfr225	olfactory receptor 225	ENSMUSG00000044061	1260	0,72
Olfr212	olfactory receptor 212	ENSMUSG00000053251	11450	0,72
Olfr934	olfactory receptor 934	ENSMUSG00000057424	933	0,72
Olfr23	olfactory receptor 23	ENSMUSG00000069816	978	0,72
Olfr968	olfactory receptor 968	ENSMUSG00000095903	1027	0,72
Olfr448	olfactory receptor 448	ENSMUSG00000043119	933	0,72
Olfr27	olfactory receptor 27	ENSMUSG00000049708	16906	0,72
Olfr39	olfactory receptor 39	ENSMUSG00000059623	4298	0,72
